# Supplementary material for: Data-mining unveils structure–property–activity correlation of viral infectivity enhancing self-assembling peptides
Source: Nat Commun. 2023 Aug 23;14:5121. doi: 10.1038/s41467-023-40663-6 (PMC10447463; doi:10.1038/s41467-023-40663-6)
Supplement: Supplementary file 1 — Supporting Information [file 41467_2023_40663_MOESM1_ESM.pdf]

Supplementary Information for

Data-Mining Unveils Structure–Property–Activity Correlation of Viral Infectivity Enhancing Self-Assembling Peptides

Kübra Kaygisiz, Lena Rauch-Wirth, Arghya Dutta, Xiaoqing Yu, Yuki Nagata, Tristan Bereau, Jan Münch, Christopher V. Synatschke, and Tanja Weil**

K. Kaygisiz, Dr. C. V. Synatschke, Prof. T. Weil
Department Synthesis of Macromolecules
Max Planck Institute for Polymer Research
Ackermannweg 10, 55128 Mainz, Germany
Email: synatschke@mpip-mainz.mpg.de, weil@mpip-mainz.mpg.de

L. Rauch-Wirth, Prof. J. Münch
Institute of Molecular Virology
Ulm University Medical Center
Meyerhofstraße 1, 89081 Ulm, Germany
Email: jan.muench@uni-ulm.de

Dr. A. Dutta, Prof. T. Bereau
Department Polymer Theory
Max Planck Institute for Polymer Research
Ackermannweg 10, 55128 Mainz, Germany

Dr. A. Dutta (*present address*)
Institute of Biochemistry II, Faculty of Medicine
Goethe University
Theodor-Stern-Kai 7, 60590 Frankfurt, Germany

X. Yu, Dr. Y. Nagata
Department Molecular Spectroscopy
Max Planck Institute for Polymer Research
Ackermannweg 10, 55128 Mainz, Germany

Prof. T. Bereau (*present address*)
Institute for Theoretical Physics
Heidelberg University
Philosophenweg 19, 69120 Heidelberg, Germany

*Corresponding authors: Christopher V. Synatschke, synatschke@mpip-mainz.mpg.de and Tanja Weil, weil@mpip-mainz.mpg.de

Content

1. REMARKS ON EXPERIMENTAL PARAMETERS AND BIOINFORMATIC DESCRIPTORS 3

1.1 EXPERIMENTAL PARAMETERS 3
1.2 BIOINFORMATIC PARAMETERS 5

2. CELL VIABILITY OF REPRESENTATIVELY SELECTED PEPTIDES 7

3. TIMESCALE OF PEPTIDE AGGREGATION 8

4. SINGLE PARAMETER CORRELATIONS 9

4.1 CORRELATION MATRIX 9
4.2 CORRELATION OF INFECTION RATES WITH HYDROPHOBICITY AND FORMATION OF β-SHEET RICH NANOSTRUCTURES 10
4.3 CORRELATION OF INFECTION RATES AND COUNT RATE WITH μM-SIZED AGGREGATE FORMATION 11

5. MULTIPARAMETER CORRELATIONS 14

6. SELECTED EXAMPLES SHOWCASING STRUCTURE–PROPERTY–ACTIVITY RELATIONSHIP 17

6.1 *N*-TERMINAL CYSTEINE LEADS TO MORE AGGREGATION AND INCREASES INFECTIVITY ENHANCEMENT 17
6.2 PEPTIDES WITH HIGH HYDROPHOBICITY CAN ENHANCE INFECTIVITY EVEN IF THEY HAVE NEGATIVE ZETA-POTENTIAL OR DO NOT FORM FIBRILS 19
6.3 PEPTIDES SUBSTITUTED WITH LOW HYDROPHOBIC AMINO ACID ALANINE DO NOT ENHANCE INFECTIVITY 20
6.4 *C*-TERMINAL SEQUENCE EXTENSIONS WITH HYDROPHOBIC AMINO ACIDS INCREASE INFECTIVITY ENHANCEMENT 22
6.5 THE CHAOTROPIC SUBSTANCE EGCG CHANGES PHYSICOCHEMICAL PROPERTIES AND HIGHLIGHTS IMPORTANCE OF SURFACE CHARGE AND INTERNAL ORDER 23

7. FURTHER INFORMATION ON PATTERN AND AMINO ACID ANALYSIS 24

IMPACT OF SEQUENCE ORDER IS GREATER FOR SHORTER SEQUENCES COMPARED TO LONG SEQUENCES 29

8. AMYLOID PEPTIDES FROM FUNCTIONAL AND PATHOGENIC CONTEXTS 30

PREPARATION OF PEPTIDE FIBRILS DESCRIBED IN LITERATURE 31

9. PEPTIDE LIBRARY: TABLES 34

BIOINFORMATIC DESCRIPTORS 40
EXPERIMENTAL DESCRIPTORS 44
MICROSCOPY IMAGES OF μM-SIZED PEPTIDE AGGREGATION 51
TEM MICROGRAPHS OF PEPTIDE LIBRARY 52
FT-IR OF PEPTIDE LIBRARY 55
LC-MS MEASUREMENT OF SYNTHESIZED PEPTIDES 61
TEM OF LITERATURE DERIVED AMYLOID PEPTIDES 62
FT-IR OF PEPTIDES DESIGNED VIA PATTERN ANALYSIS 64
FT-IR OF LITERATURE DERIVED PEPTIDES 66

SUPPLEMENTARY REFERENCES 69

1. Remarks on experimental parameters and bioinformatic descriptors

1.1 Experimental parameters

Transmission electron microscopy (TEM) measurements. TEM measurements were used to control if peptides assemble. The assemblies were categorized in non-fibrillar structures (no assembly, amorphous and micellar aggregates) and fibrillar structures (fibrils, bundles, sheets) as shown in **Figure S1**. A peptide was defined as non-fibrillar if no fibrillar structure was observed once on the entire TEM-grid but only amorphous aggregates. In contrast, peptides defined as fibrillar may also include amorphous structures. Amongst nonfibrillar assemblies also amorphous aggregates are summarized. Amorphous aggregates are defined as structures with high β-sheet content which lack long-range order, that is crystallinity or fibril formation. It is generally assumed that these structures might be oligomers from on or off pathway amyloid assembly.[1–4]

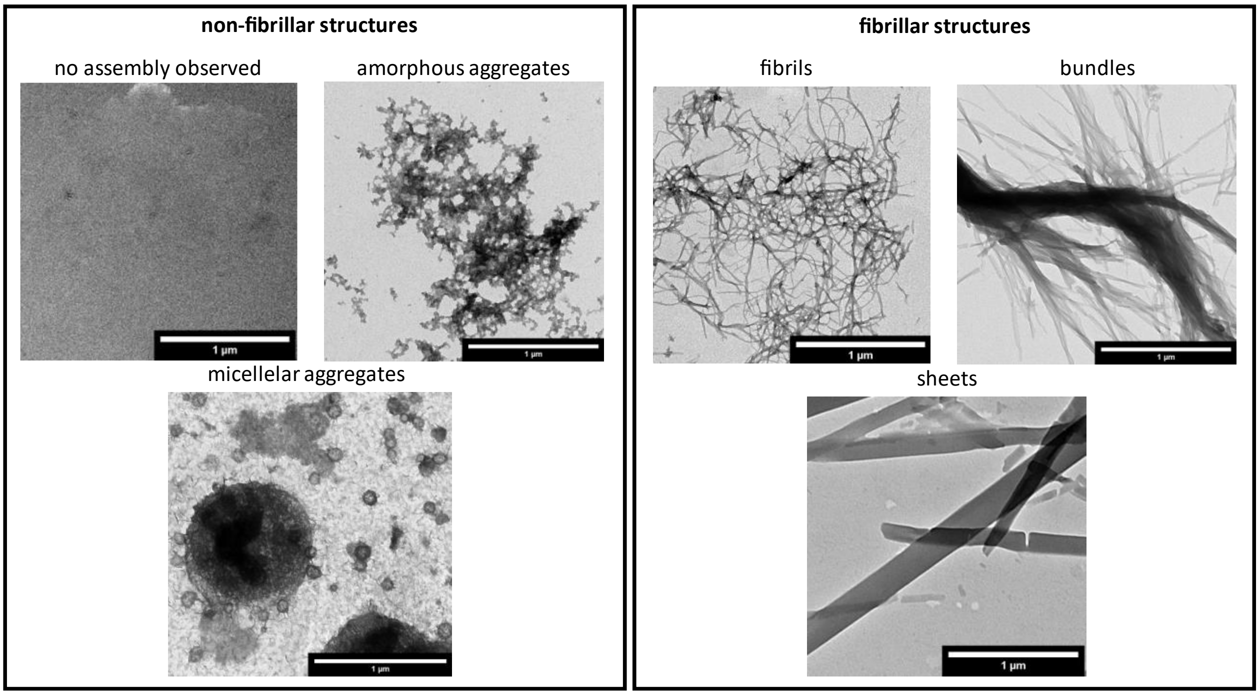

Figure S1 Overview of observed morphologies using representative examples and categorization into non-fibrillar structures (no assembly, amorphous aggregates, micellar aggregates) and fibrillar structures (fibrils, bundles, sheets).

Thioflavin T (ThT) fluorescence measurement. The ThT-fluorescence assay is used as a standard method to show the presence of amyloidal secondary structure. ThT binds to β-sheet rich structures, which immobilizes molecular rotation and enables excitation at 450 nm and emission at 482 nm.[5,6] However, it has to be kept in mind, that this assay is prone to show large variations in fluorescence intensity depending on structural polymorphism,[7] electrostatic interactions with positive charged particles,[8] and in the presence of aromatic amino acid such as phenylalanine (F), tryptophane (W) and tyrosine (Y).[5,9] Consequently, in this study a classification into ThT-active (> 2 fold compared to control without peptide) and non-active peptides will be considered rather than comparing exact ThT fluorescence intensities (**Table S5, S6, S7**).

Turbidity measurements via light scattering. Most of the peptide fibrils investigated in this study aggregate into microscopically large clusters with visible turbidity as can be also observed in bright field and fluorescence microscopy (**Figure 2**, **Figure S31**, **Figure S32**). In general, there are two main

ways to determine turbidity of samples, this can be via transmittance and via scattering. Transmittance is typically measured above 400 nm where the peptide itself is not absorbing and is frequently applied as it is an easy and fast method.[10,11] However, transmittance contributes not only from µm-sized aggregates but also nm-sized aggregates such as distinct fibrils, which makes hard to distinguish between fibril formation and micrometer sized aggregation. Although dynamic light scattering is a frequently applied method for determination of particle size, it cannot be conducted in solutions with anisotropic aggregates in micrometer size range, since the Stokes-Einstein relation is only applicable for homogeneous anisotropic spherical particles.[12] Further models describing scattering intensity and particles sizes, such as Mie or Rayleigh cannot be applied to these self-assembling peptides containing heterogeneous mixture of aggregates ranging from nano- to micrometer in size.[10] Since the fibrils formed by the short-self assembling sequences are highly polymorphic (TEM, **Figure S33**) and form heterogenous aggregate size (microscopy, **Figure S32**), it is not straightforward to apply DLS to determine polydispersity of aggregates. We assume that the heterogeneity of fibril aggregation in our peptide library originates from fast self-assembly kinetics and the resulting turbidity is observed already by bare eye within seconds after inducing assembly. Therefore, we used the derived count rate of scattered light to gain information on turbidity of fibrils solutions.[1,13] Light scattering rates detected in a backscattering setup at 173° is suitable for evaluating concentrated samples.[12] Still this method bears the risk that insoluble particles (other than assembled and aggregated) are contributing to the scattering events. Therefore, the scattering measurement is accompaniment by complementary characterization methods such as TEM (for nanostructure verification, **Figure S33**) and microscopy (for macroscopic verification, **Figure S32**).

Zeta Potential. The zeta-potential describes the electrophoretic mobility of a particle in an electric field. For the case of supramolecularly assembled peptides, this method provides information about the surface charge of peptide fibrils and aggregates as well as information about aggregate size because this also plays a role for the final mobility. The net charge of a peptide does not necessarily correlate with the measured zeta-potential of the molecularly dissolved peptide or an aggregate of peptide. It is known that peptides with similar sequence and same net charge can differ significantly in their zeta-potential depending on the pH and aggregation state of the fibrils.[14–16]

Infectivity Data. All infectivity data discussed in this study are referring to an *in vitro* peptide concentration of 1.3 µM on cells (**Table S5**). The virus stock concentration is same for all peptides within one experiment. The infectivity enhancement of a peptide was determined via a luminescence assay, which detects β galactosidase expressed by TZM-bl cells upon infection of HIV-1. To evaluate the infectivity enhancement capability of a peptide, first the background luminescence (cell-only without virus or peptide) was subtracted and set relative the corresponding virus-only infectivity, which was simultaneously measured on each well plate. Then the infectivity was calculated relative to EF-C infectivity at 1.3 µM, which was applied as a reference peptide to ensure comparability of infectivity data among different experiments. Noteworthy, the logarithm of the infection enhancement relative to EF-C (Log Infection Rel. EF-C) correlated with a higher Pearson *R* with peptide properties compared to absolute infection enhancement values (**Figure S5**). To enable linear regression, we therefore applied logarithmic scale on infectivity relative to EF-C for all the correlations. The asymmetric error bars for the logarithmic plot were calculated according to eq (S1) and eq (S2) for positive σ^+ and negative σ^-errors, respectively.

$$\sigma^+ = \log_{10}(\text{Infection Rel EF-C} + \Delta\ \text{Infection Rel EF-C}) - \log_{10}\text{Infection Rel EF-C} \quad \text{eq (S1)}$$

$$\sigma^{-} = \log_{10} \text{Infection Rel EF-C} - \log_{10} (\text{Infection Rel EF-C - } \Delta \text{ Infection Rel EF-C}) \quad - \text{eq (S2)}$$

1.2 Bioinformatic parameters

A selection of bioinformatic parameters were calculated for all peptides of the peptide library according to previously reported protocols.[17,18] The descriptors include peptide net charge, hydrophobicity, isoelectric point, aliphatic index, hydrophobic moment index, boman index, instability index, graph shape index, upsilon steric parameter, smoothed upsilon steric parameter, polarizability, normalized van der Waals volume (**Table S4**). It must be noted that the bioinformatic descriptors only evaluate the primary sequence of peptides and proteins and not aggregation propensities. **Net charge.** The sum of the charges of amino acid side chains at a certain pH is the net charge. The net charge was calculated (pH = 7.4, pKscale = EMBOSS) with the "peptides" package in R.[18] **Hydrophobicity.** The hydrophobicity gives information about average hydrophobicity of the peptide calculated by hydrophobicity of amino acid side chains. The calculation of the hydrophobicity was applied according to Fauchère[17] as an experimental based on water–octanol partition coefficients and Kyte-Doolittle[19] as a calculation based hydrophobicity scales. The calculation of hydrophobicity was conducted via the "peptides" package in R.[18] **Isoelectric point (pI)** The pI describes the pH at which all negative and positive charged side chains of a peptide are statistically balanced (total net charge neutral). Since all peptides are incubated at pH 7.4 in phosphate buffer the side chains of the peptides with pI higher than 7.4 are positively charged. Peptides with pI lower than 7.4 are accordingly negative charged. The pI was calculated with the pKscale EMBOSS via "peptides" package in R.[18] **Aliphatic index.** The aliphatic index indicates the thermostability by evaluating the amount of aliphatic amino (alanine (A), isoleucine (I), leucine (L) and valine (V)) acids in a protein.[20] The aliphatic index was calculated via the "peptides" package in R.[18] **Hydrophobic moment index.** The hydrophobic moment index is proposed to measure amphiphilicity perpendicular to the axis of a peptide structure.[21] The calculate of the hydrophobic moment index (angle = 100, window = 11) was conducted with the "peptides" package in R.[18] **Boman index.** The sum of solubility for amino acid side chains normalized by the number of amino acids gives an estimation of the peptide-protein or peptide-membrane interaction. A boman index, which is negative or nearly 0, is assumed to be connected to high membrane interaction, e.g. for antimicrobial activity.[22] The boman index is calculated with the "peptides" package in R.[18] **Instability index.** The *in vivo* stability of a protein is estimated by prevalence of pairs of amino acid in a peptide. Thereby the direct proximity of certain amino acids in stable and unstable peptides is evaluated and gives back information on composition in a simplified way. A peptide with a instability index below 40 is considered to be stable.[23] The instability index is calculated with the "peptides" package in R.[18] **Graph shape index.** The graph shape index measures the sterical demand (branching, symmetry, complexity) of the amino acid side chains. The index for a peptide was calculated by dividing through number of amino acids after adding up the respective single amino acid graph shape descriptors.[17] **Upsilon steric parameter.** The upsilon steric parameter measures steric effects by considering the minimal van der Waals radius of amino acid side chains. The index for a peptide was calculated by dividing through number of amino acids after adding up the respective single amino acid descriptors.[17] **Polarizability.** The polarizability is a bulk parameter describing the molecular volume and models dispersion forces. It is related to molar refractivity, which gives information on electrical properties of a molecule. The index for the peptide was calculated by dividing through number of amino acids after adding up the respective single amino acid descriptors.[17] **Normalized van der Waals volume.** The van der Waals volume is another bulk parameter correlating with polarizability and was calculated for a peptide by

dividing through number of amino acids after adding up the respective single amino acid side chain van der Waals volume.[17]

Linear Regression. For conducting multiple linear regression, the constrained multiple linear regression tool (v.1.10) of OriginPro, Version 2021 was applied. As a dependent variable (y) the Log Infection Rel EF-C was selected, the independent variables (A) were the features of interest. The selected model for regression analysis is constrained linear least-squares problems solution with the following general equation: y = A0+A1*x1+A2*x2+[…].

2. Cell Viability of Representatively Selected Peptides

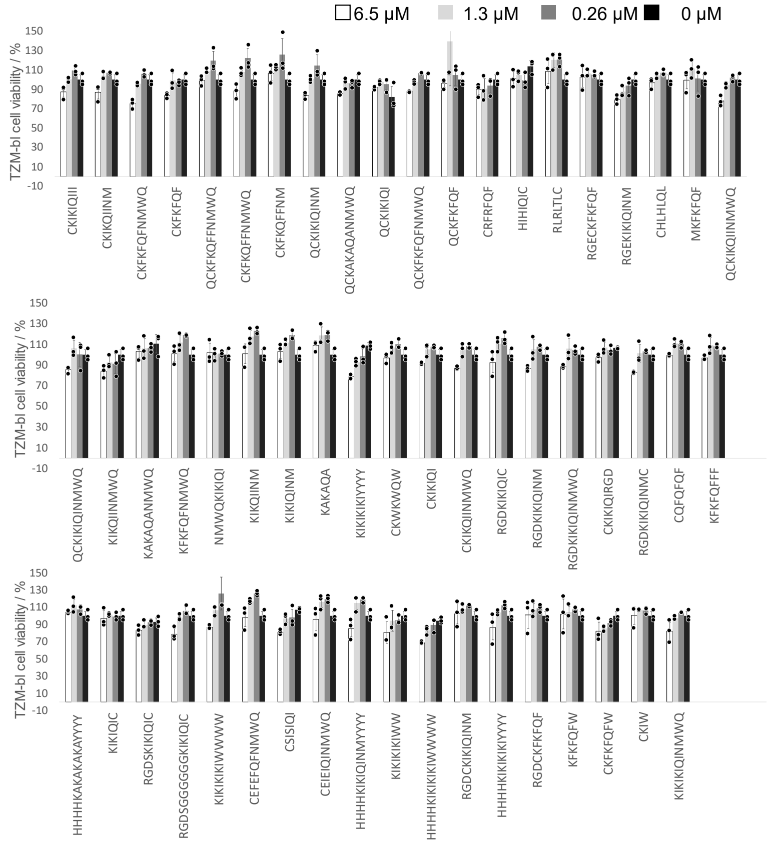

Figure S2 Cell Viability normalized to 100% metabolic activity as determined by CellTiterGlo Assay of 57 representatively selected peptides from the library. The selection represents infectivity enhancing and not enhancing peptides (**Table S5**) and show that cell viability (> 80%) is maintained for all peptides at 1.3 µM concentration. Error bars indicate standard deviation from triplicate measurements. Source data for Figure S2 is provided.

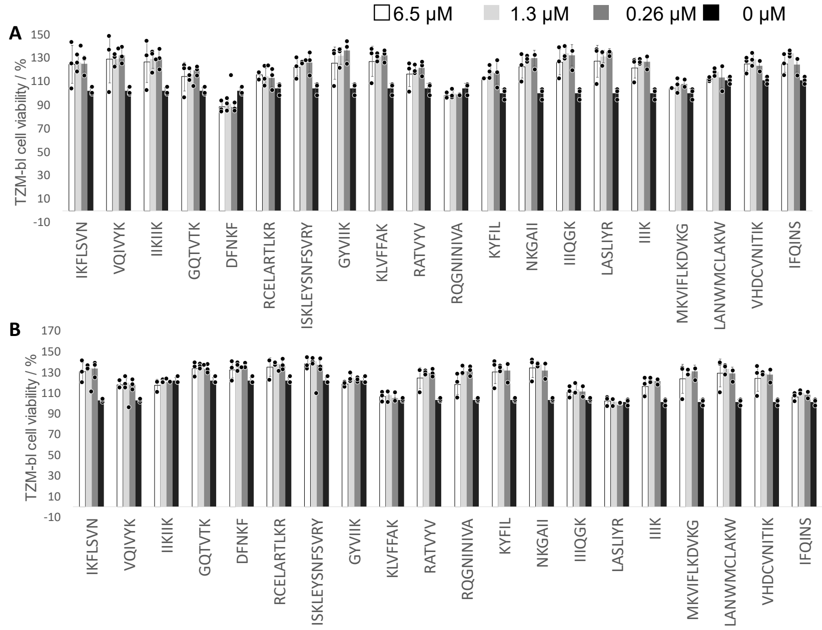

Figure S3 Cell Viability normalized to 100% metabolic activity determined by CellTiterGlo Assay of 20 peptides reported in literature.[33,34,43–48,35–42] Cell viability (> 80%) is maintained for all peptides at 1.3 µM concentration regardless of the preparation method, which is **A** PNF formation method by dilution of 10 mg/mL peptide stock in DMSO in PBS to 1 mg/mL or **B** preparation according to literature reported protocol (**SI Section 8**). Error bars indicate standard deviation from triplicate measurements. Source data for Figure S3 is provided.

3. Timescale of peptide aggregation

Fibril formation and µm-sized aggregation occur immediately upon incubation of peptides in PBS as demonstrated exemplary for CKFKFQF with TEM micrographs (**Figure S4**A, B) and by turbidity as observed via bare eye (data not shown), and light scattering exemplary shown for CKFKFQF (**Figure S4**C) and QCIKIQINMWQ (**Figure S4**D). No significant change in light scattering count rate is observed regardless of measurement directly after sample preparation (T_0) or after incubation for 1 day (T_{1d}) or 2 months (T_{2m}), indicating that the fibril formation and aggregation is completed within the timeframe of sample preparation (within seconds).

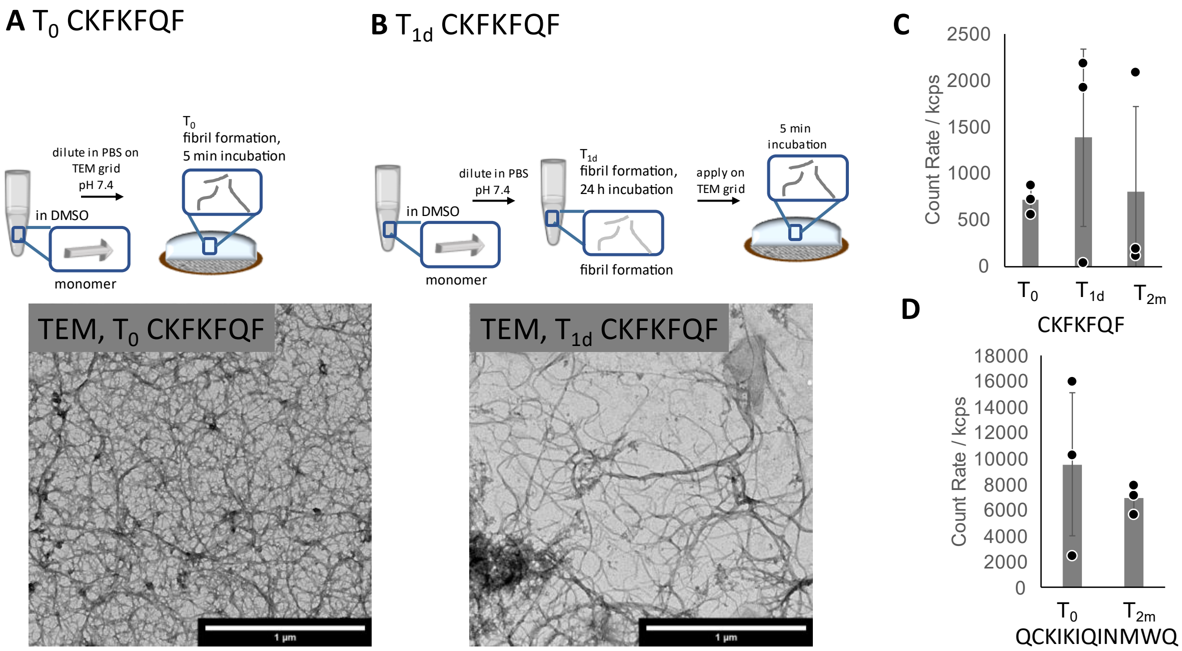

Figure S4 Time dependent fibril-formation and aggregation of selected peptides CKFKFQF and QCIKIQINMWQ. **A** Schematic illustration and TEM micrograph of T_0 incubation condition. The peptide monomer, dissolved in DMSO (10 mg/mL), was added to a PBS droplet on a TEM grid and incubated at 1 mg/mL concentration for 5 min. Fibril formation is observed via TEM (scale bar 1 µm). **B** Schematic illustration and TEM micrograph of T_{1d} incubation condition. The peptide monomer, dissolved in DMSO (10 mg/mL), was added to PBS and incubated at 1 mg/mL concentration for 24 h. Then, the peptide fibrils were incubated on a TEM grid for 5 min. Fibril formation was observed via TEM (scale bar 1 µm). **C** The count rate of scattered light was determined from Zeta-Potential measurements for CKFKFQF (0.1 mg/mL) after T_0, T_{1d}, and T_{2m}. For T_0 incubation condition, the peptide was diluted from DMSO (10 mg/mL) in PBS (1 mg/mL) and immediately further diluted in 1 mM KCl to 0.1 mg/mL for measurement, which tookapproximately 3 min. For T_{1d} and T_{2m} measurements, the sample was incubated at 1 mg/mL concentration for one day and two months, respectively, and diluted in 1 mM KCl to 0.1 mg/mL just before measurement. Error bars indicate standard deviation from triplicate measurements. **D** The count rate of scattered light was determined from Zeta-Potential measurements for QCIKIQINMWQ (0.1 mg/mL) after T_0, and T_{2m}. Created with BioRender.com. Error bars indicate standard deviation from triplicate measurements. Source data for Figure S4 C, D is provided.

4. Single Parameter Correlations

4.1 Correlation matrix

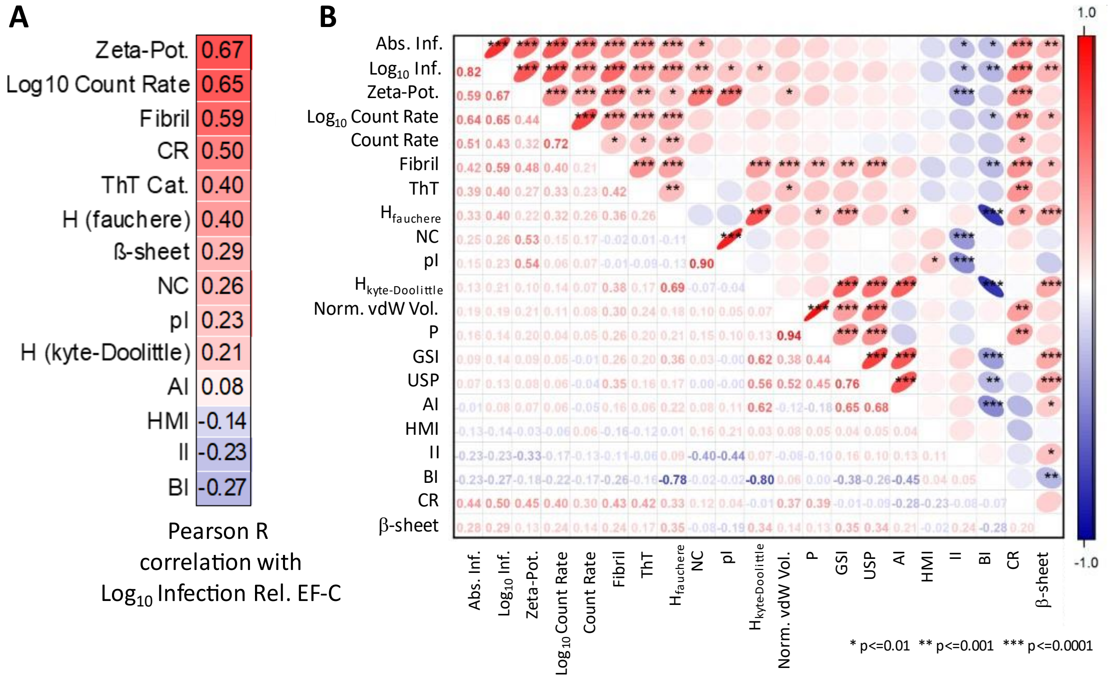

Figure S5 A Pearson *R* correlation factor of Log_{10} Infection Rel EF-C values with experimental and calculated properties of 163 peptides of an EF-C based library. **B** Correlation matrix of physicochemical and bioinformatic properties of a peptide library based on EF-C (**Table S4**, **Table S5**). All columns except of Conversion Rate (CR) contain data of 163 peptides. Conversion rate was determined for 80 representative subset of peptides (**Table S5**). Abbreviations: Absolute Infectivity at 1.3 µM peptide concentration (Abs. Inf.), and the logarithmic infectivity relative to EF-C (Log_{10} Inf.), the derived count rate of scattered light from zeta-potential measurements (Log_{10} Count Rate), zeta potential (Zeta-Pot.), fibril formation (Fibril) as evaluated qualitatively from TEM measurements (**Figure S33**), conversion of monomers to assembled structure (Conversion Rate, CR), percentual β–sheet content from fourier transform infrared spectroscopy (FT-IR, **Figure S34**) measurements (β–sheet), categorical ThT fluorescence (a peptide is ThT-active, if *n*-fold ThT fluorescence is >2, **Table S5**), Net charge (NC), hydrophobicity according to Fauchère scale ($H_{fauchere}$) and Kyte-Doolittle scale ($H_{Kyte\text{-}Doolittle}$), isoelectric point (pI), aliphatic index (AI), hydrophobic moment index (HMI), boman index (BI), instability index (II), graph shape index (GSI), upsilon steric parameter (USP), polarizability (P), and normalized van der Waals Volume (Norm vdW Vol.) **Table S4**. Significance level is determined via Pearson two-sided t-distribution test and indicated by p-values *p<= 0.01, **p<= 0.001, ***p<= 0.0001. Source data for Figure S5 is provided.

4.2 Correlation of infection rates with hydrophobicity and formation of β-sheet rich nanostructures

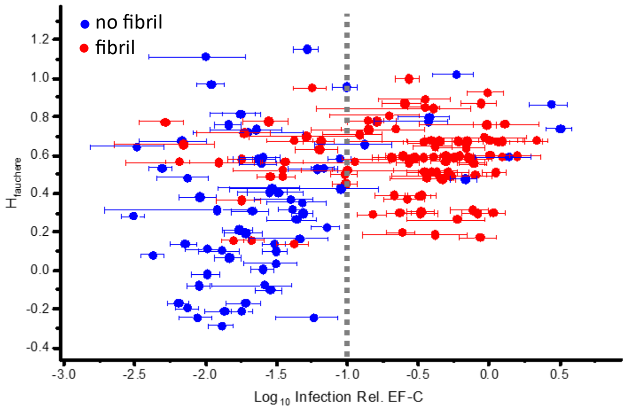

Figure S6 Graphical representation of calculated hydrophobicity (**Table S4**) plotted versus $\log_{10}$ Infection Rel. EF-C (**Table S5**). The color code represents fibril formation (red) and no fibril formation (blue) as observed via TEM (**Figure S33**, **Table S5**). Interestingly, there are no active peptides (dashed line, Infection Rel EF-C <10% or Log Infection Rel EF-C <-1) with a hydrophobicity lower than 0.17. From these active peptides 90% are forming fibrils. The majority (69%) of peptides with a hydrophobicity higher than 0.17 are also forming fibrils, illustrating a positive correlation between hydrophobicity, fibril formation and infectivity enhancement. Error bars indicate standard deviation from triplicate measurements. Source data for Figure S6 is provided.

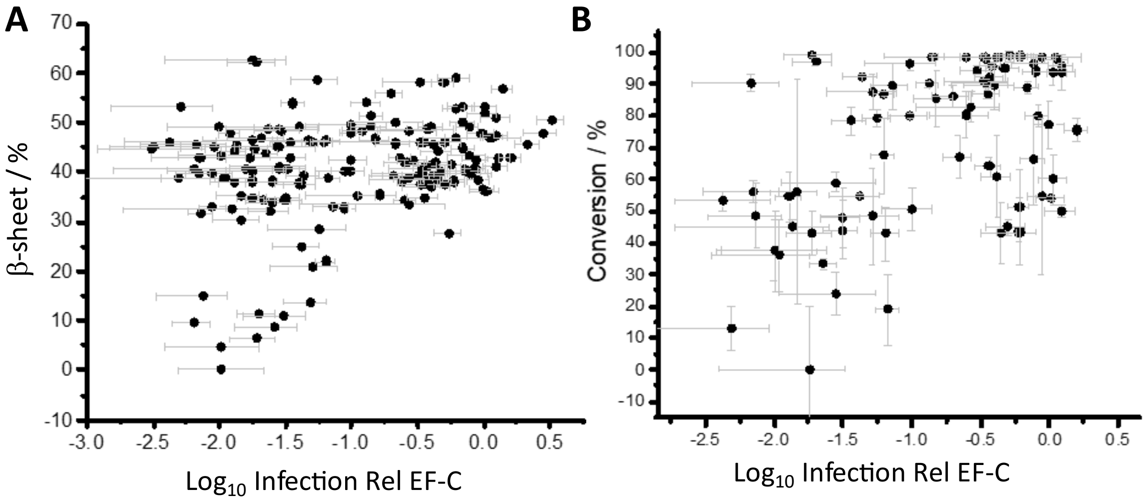

Figure S7 Characterization of β-sheet and monomer to assembly conversion of the peptide library (**Figure S34**, **Table S5**). Error bars indicate standard deviation from triplicate measurements. **A** β-sheet content obtained from attenuated total reflection (ATR)-FT-IR measurements plotted against $\log_{10}$ infection relative to EF-C. **B** Conversion of monomers to assembled structure contain data of 80 peptides plotted against $\log_{10}$ infection relative to EF-C. Source data for Figure S7 is provided.

4.3 Correlation of infection rates and count rate with µm-sized aggregate formation

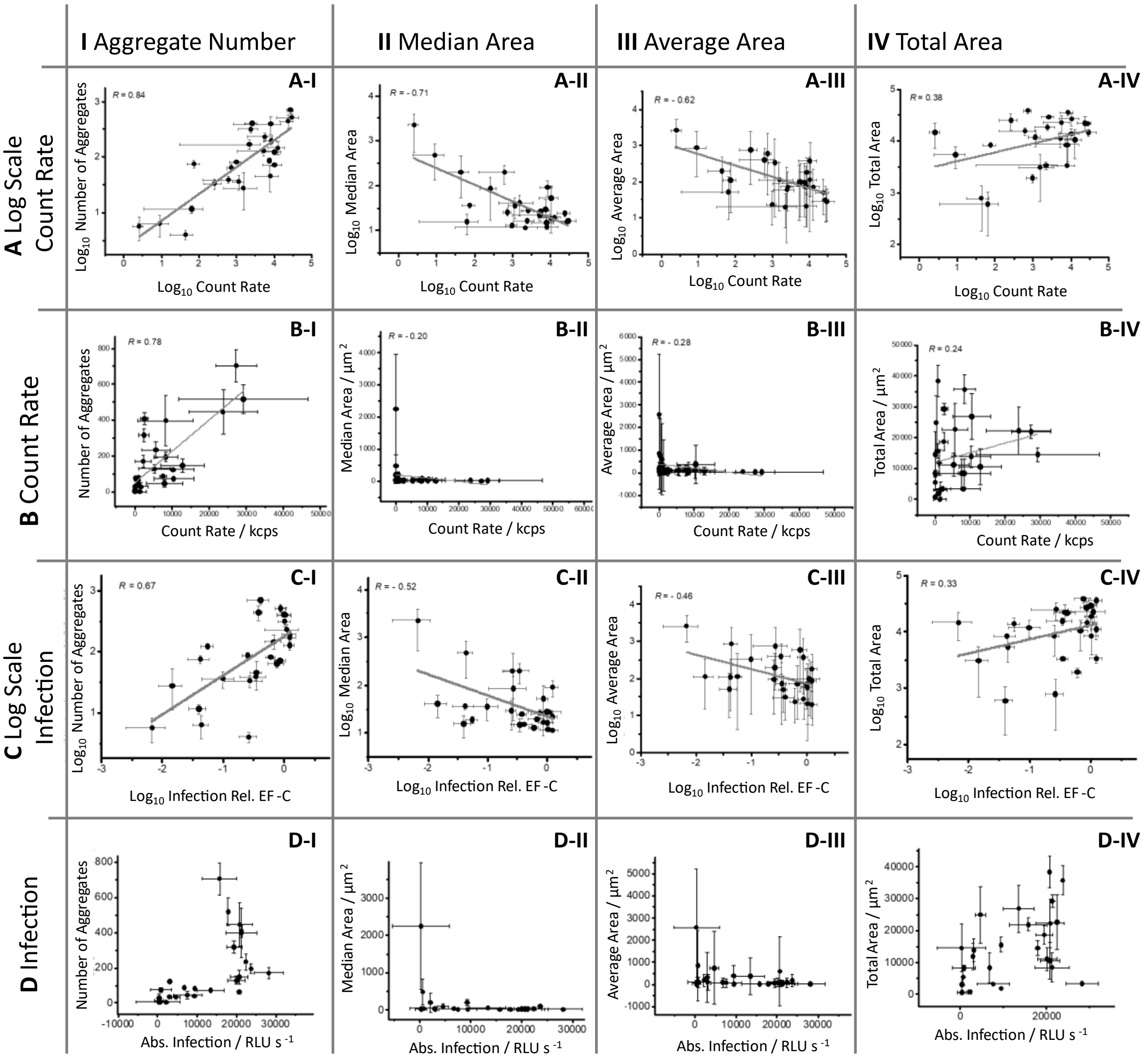

Figure S8 Graphical summary of aggregate size and number analysis as correlation plots with count rate and infection rate. **A-I** Log_{10} number of aggregates plotted against Log_{10} count rate of scattered light display a high correlation coefficient R = 0.84. **A-II** Log_{10} median area and **A-III** Log_{10} average area of aggregates plotted against Log_{10} count rate of scattered light display a negative correlation (R = – 0.71 and – 0.62, respectively) indicating that smaller aggregates are contributing stronger to count rate than larger ones. **A-IV** Plotting the Log_{10} total area against the Log_{10} count rate of scattered light show no good correlation (R = 0.38). **B-I** Number of aggregates plotted against count rate of scattered light display a high correlation coefficient R = 0.78. **B-II** Median area and **B-III** average area of aggregates plotted against the count rate of scattered light display no linear relationship and thus no good correlation (R = – 0.20 and – 0.28, respectively) indicating that log_{10} scale is a better measure to evaluate aggregates. **B-IV** Plotting the total area of aggregates against the count rate of scattered light show no good correlation (R = 0.24). **C-I** Log_{10} number of aggregates plotted against Log_{10} Infection Rel. EF-C display a good correlation coefficient R = 0.67. **C-II** Log_{10} median area and **C-III** Log_{10} average area of aggregates plotted against Log_{10} Infection Rel. EF-C display a negative correlation (R = – 0.52 and – 0.46, respectively) indicating that smaller aggregates are more active than larger ones. **C-IV** Plotting the Log_{10} total area against the Log_{10} Infection Rel. EF-C show no good correlation (R = 0.33). **D-I** Number of aggregates **D-II** median area and **D-III** average area of aggregates plotted against Absolute Infection (RLU, relative light units) display a non-linear relationship indicating that log_{10} scale is a better measure to evaluate aggregates. **D-IV** Plotting the total area of aggregates against the count rate of scattered light show no correlation. All aggregate analyses were performed in triplicate measurements (Std. Dev.) by automated 3D objects counting function (ImageJ) from widefield microscopy images (**Figure S32**), area 1330 µm × 1330 µm. The count rate and infection rate data are retrieved from **Table S8**. Mean values are displayed with error bars indicating standard deviation from triplicate measurements. Source data for Figure S8 is provided.

The light scattering count rate as well as the infectivity enhancement correlate with increasing number of visible aggregates (**Figure S8**I, *R* = 0.84 for Log_{10} Count Rate correlation with Log_{10} Aggregate Number (**Figure S8**AI). In contrast, having few larger aggregates (> 100 μm^2) does not enhance infectivity as can be observed from the negative correlation between infection rates and count rates in **Figure S8**II, III. We hypothesize that either too few virions can associate with the larger fibril aggregates or that many smaller aggregates can transduces many cells more efficiently than few larger aggregates. This is supported by the correlation of the cumulative surface area of the particles that, instead of the full distribution, only considers total surface area (**Figure S8**IV), and shows no correlation with the count rate or infectivity.

Mechanical downsizing of µm-sized aggregates can be achieved by applying pressure on a peptide solution which is entrapped between two glass slides (**Figure S9**AII, BII, C). However, upon releasing the physical pressure, the initial aggregate size is recovered within seconds (**Figure S9**AIII, BIII, C). This fast recovery likely originates from the fast non-covalent self-assembly of the peptide structures. This is emphasized also by ultrasonication experiments, which show comparable aggregation before and after ultrasonication (**Figure S9**D) and similar infectivity values for peptides treated and not treated with ultrasonication (**Figure S9**E). Thus, it is not possible to tune the aggregate sizes under comparable conditions for the same peptide sequence by simple mechanical forces.

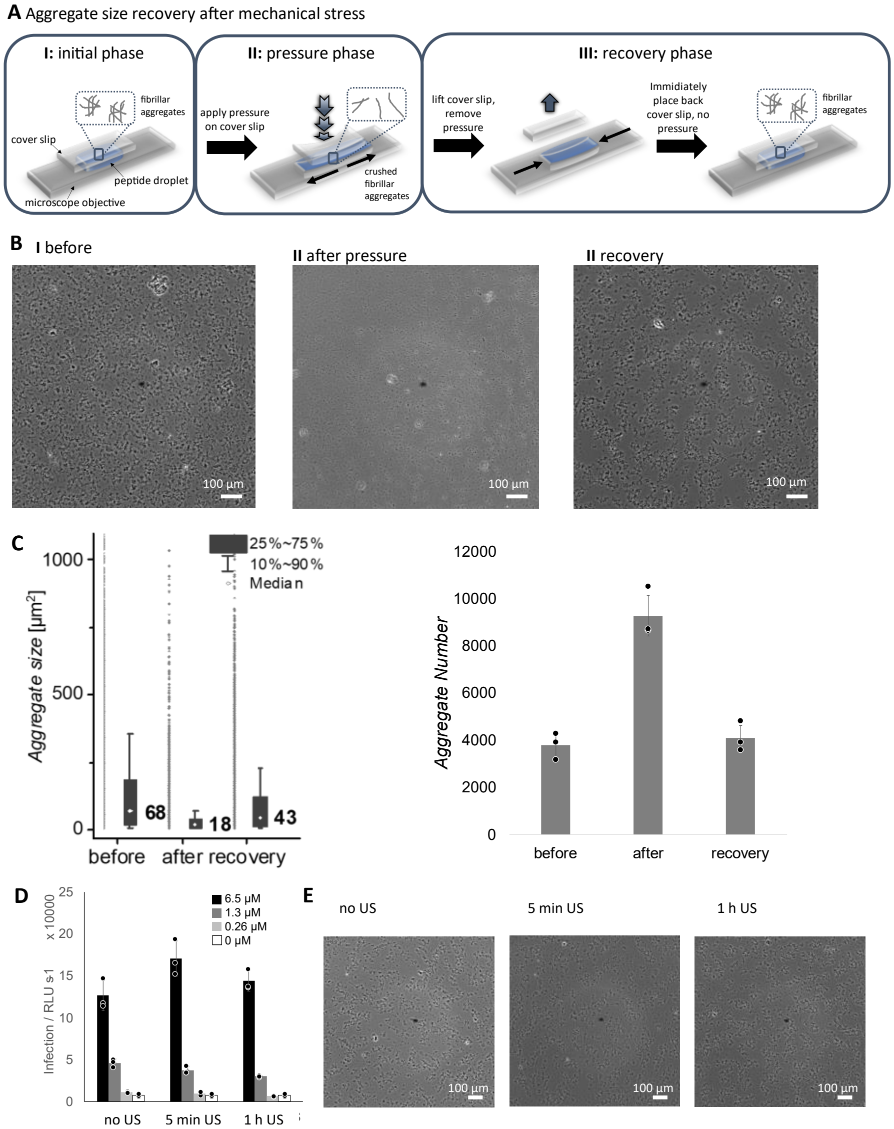

Figure S9 Changes in aggregate size upon mechanical stress. **A** Schematic illustration of **I** aggregates composed of self-assembling peptide fibrils placed on a microscope objective and covered by a glass cover slip. **II** Applying pressure on glass cover slip mechanically downsizes aggregates, **III** however, once the pressure is released (*e.g.* by lifting the cover slip) the initial aggregate size is recovered. **B** Brightfield microscopy measurements (objective 20x) of CKFKFQF (1 mg/mL, 30 µL) covered by a glass cover slip, **I** before applying pressure, **II** after applying pressure and **III** after releasing pressure by lifting cover slip, scale bar 100 µm. **C** Aggregate size distribution for objects > 10 µm^2 in an area of 1331 µm × 1331 µm. Box plots show median with whiskers indicating 10-90% and boxes indicating 25-75% of all data displayed on the left side to each box. In the bar plot mean values are displayed with error bars indicating standard deviation from triplicate measurements. **D** Applying ultrasonication (US) on CKFKFQF (1 mg/mL in PBS) for 5 min or 1 h does not affect infection rates or **E** aggregate size and number compared to no ultrasonication, brightfield microscopy scale bar 100 µm. Error bars indicate standard deviation from triplicate measurements. Source data for Figure S9 C, D is provided.

5. Multiparameter Correlations

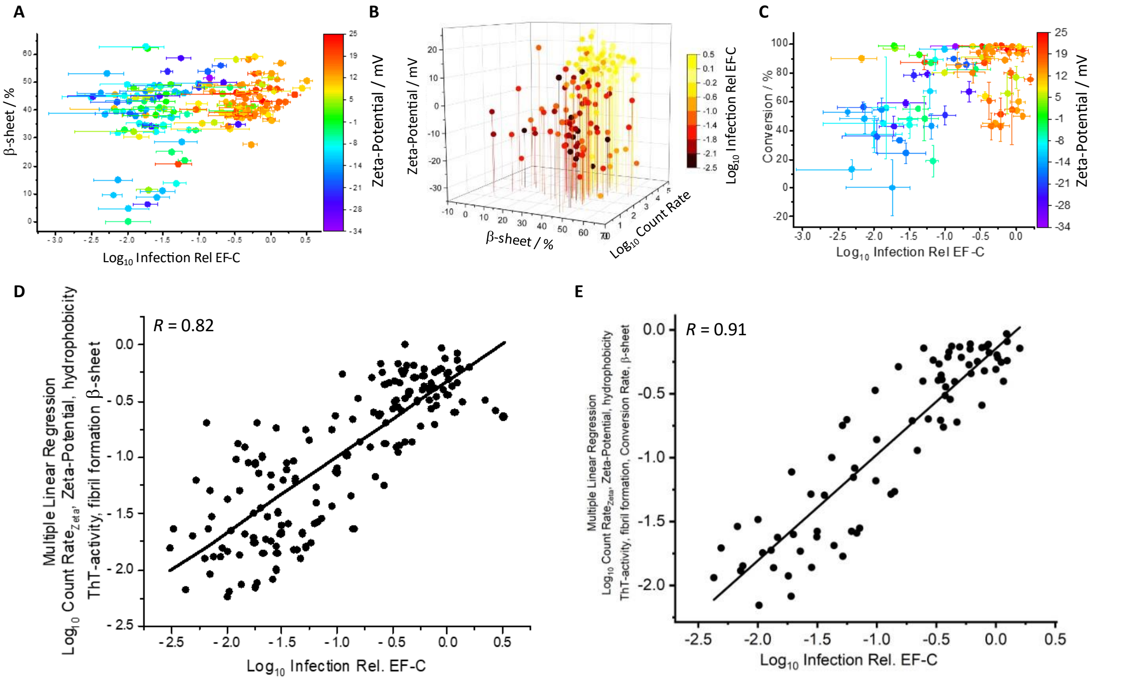

	Value	Standard Error	t-Value	P >|t|
Intercept	-2.33472	0.16267	-14.3527	0
Zeta-Pot.	0.02128	0.00311	6.83608	1.79E-10
Log_{10} Count Rate	0.29879	0.04746	6.29522	3.05E-09
Fibril	0.27355	0.09097	3.00697	0.00308
$H_{Fauchere}$	0.26241	0.12751	2.0579	0.04129
ThT-activity	0.10774	0.07976	1.35076	0.17876
β-sheet	0.00356	0.00323	1.10357	0.2715

	Value	Standard Error	t-Value	P >|t|
Intercept	-2.21225	0.18525	-11.9422	0
Zeta-Pot.	0.01798	0.00283	6.34982	1.79E-08
Fibril	0.47174	0.10511	4.48813	2.71E-05
Log_{10} Count Rate	0.20446	0.0478	4.27703	5.81E-05
ThT-activity	0.19678	0.10179	1.93317	0.0572
β-sheet	0.00698	0.00374	1.86557	0.06623
$H_{Fauchere}$	0.06023	0.13112	0.45933	0.6474
Conversion	2.65E-04	0.00173	0.15333	0.87857

Figure S10 Multiparameter analysis of assembly (**Figure S33**), secondary structure (**Figure S34**), surface charge and microscopic aggregation (**Table S5**). **A** β-sheet content plotted against Log_{10} Infection Rel. EF-C. The color bar indicates the zeta-potential. Error bars indicate standard deviation from triplicate measurements. **B** Peptides with a high β-sheet content, which are microscopically aggregating (high count rate) and have a positive zeta-potential are enhancers of viral transduction (top right corner). The color bar indicates Log_{10} Infection Rel. EF-C. **C** Conversion of monomers to assembled structures (Conversion Rate, CR) plotted against Log_{10} Infection Rel. EF-C of 80 selected peptides (**Table S5**). The color bar indicates the zeta-potential. Error bars indicate standard deviation from triplicate measurements. Note, that for one peptide (CKFKQFF) with -2.2 Log_{10} Infection Rel to EF.C shows no infectivity enhancement despite its positive zeta-potential and high CR. For this peptide the turbidity is very low and amorphous aggregates rather than fibril formation is observed, which indicates no microscopic aggregation. **D** Multiple linear regression (c*onstrained linear least-squares problems solution) with the equation: y* = A0+A1*x1+A2*x2+[…]+ A5 * x5 and zeta-pot, Log_{10} Count Rate, Fibril formation, $H_{fauchere}$, ThT-activity and β-sheet as independent variables (A, listed in the order of ascending P variable) and Log_{10} Infection Relative EF-C as dependent target variable (y). Linear Fit with Pearson correlation factor 0.82. Statistically significant parameters ($p < 0.05$) are highlighted. **E** Multiple linear regression (constrained linear least-squares problems solution) with the equation: y = A0+A1*x1+A2*x2+[…]+ A7 * x7 and Fibril formation, zeta-pot, Log_{10} Count Rate, β-sheet, $H_{fauchere}$, ThT-activity and Conversion Rate as independent variables (A, listed in the order of ascending P variable) and Log_{10} Infection Relative EF-C as dependent target variable (y) with 79 peptides from the library which include CR as well as FT-IR data. Linear Fit with Pearson correlation factor 0.91. Significance level is determined via Pearson two-sided t-distribution test and indicated by p-values. Statistically significant parameters ($p < 0.05$) are highlighted. Source data for Figure S10 is provided.

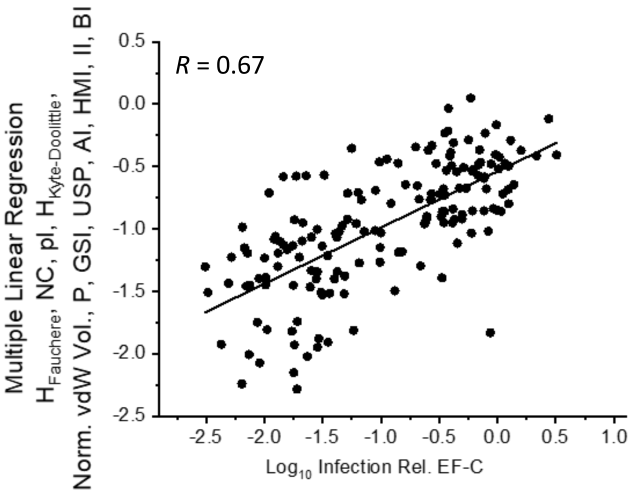

	Value	Standard Error	t-Value	Prob>|t|
Intercept	-3.63904	0.839	-4.33736	2.63E-05
$H_{Fauchere}$	1.79563	0.34155	5.2573	4.95E-07
P	-30.14357	6.52932	-4.61665	8.32E-06
AI	-0.00839	0.0021	-3.98661	1.04E-04
HMI	-1.15218	0.30454	-3.78336	2.23E-04
USP	3.99456	1.36645	2.92331	0.004
Norm. vdW Vol.	0.70321	0.24673	2.8501	0.00499
GSI	0.6973	0.26492	2.63217	0.00937
II	-0.00259	0.00127	-2.04129	0.04297
NC	0.12281	0.0659	1.86361	0.06433
pI	0.09049	0.05484	1.6499	0.10106
BI	0.10473	0.08246	1.27011	0.20601
$H_{Kyte\text{-}Doolittle}$	-0.05017	0.14137	-0.35489	0.72317

Figure S11 Multiple linear regression (constrained linear least-squares problems solution) with the equation: y = A0+A1*x1+A2*x2+[…]+ A12 * x12. The independent variables are Net charge (NC), hydrophobicity according to Fauchère scale ($H_{fauchere}$) and Kyte-Doolittle scale ($H_{Kyte\text{-}Doolittle}$), isoelectric point (pI), aliphatic index (AI), hydrophobic moment index (HMI), boman index (BI), instability index (II), graph shape index (GSI), upsilon steric parameter (USP), polarizability (P), normalized van der Waals Volume (Norm vdW Vol.), **Table S4**. The independent variables (A) are listed in the order of ascending P variable. Log_{10} Infection Relative EF-C is the dependent target variable (y) and linear fit with Pearson correlation factor is 0.67. Significance level is determined via Pearson two-sided t-distribution test and indicated by p-values. Statistically significant parameters ($p < 0.05$) are highlighted. Source data for Figure S11 is provided.

To avoid overfitting of the multiple linear regression control fits were conducted with non-active peptides and showed no statistically significant correlation (**Figure S12**).

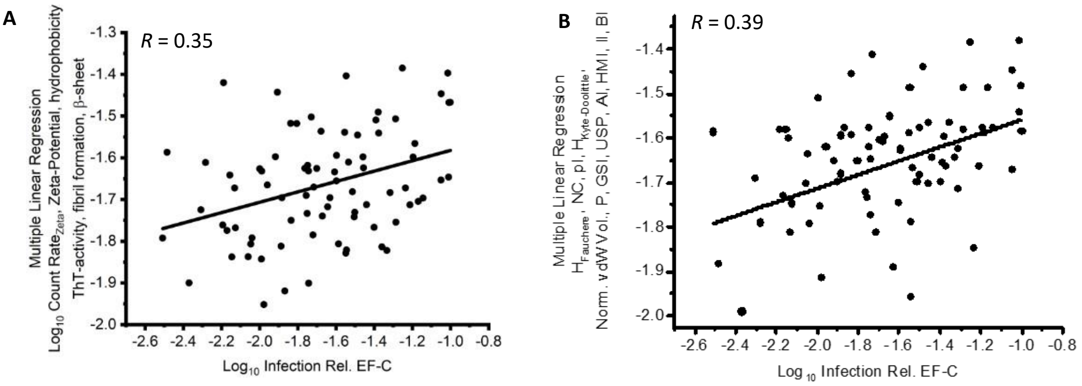

	Value	Standard Error	t-Value	Prob>|t|
Intercept	-1.88885	0.1721	-10.9754	0
Log_{10} Count Rate	0.08681	0.06463	1.34317	0.18338
ThT-activity	0.12609	0.09898	1.2739	0.20674
$H_{Fauchere}$	0.13761	0.12161	1.13159	0.26151
Zeta-Pot.	0.00351	0.00411	0.85224	0.39687
Fibril	0.05948	0.10263	0.57958	0.56398
β-sheet	-0.00119	0.00315	-0.37622	0.70784

	Value	Standard Error	t-Value	Prob>|t|
Intercept	-2.31348	0.7412	-3.12127	0.00264
HMI	-0.48614	0.25335	-1.91886	0.0592
P	-7.86118	4.9337	-1.59336	0.11572
$H_{Fauchere}$	0.45682	0.30324	1.50646	0.13658
AI	-0.00162	0.0016	-1.01192	0.31516
Norm. vdW Vol.	0.16416	0.18077	0.90807	0.36705
USP	1.05354	1.16697	0.9028	0.36982
GSI	0.17629	0.19697	0.89501	0.37394
pI	0.03747	0.04735	0.79131	0.43152
NC	0.03404	0.05421	0.62789	0.53218
II	3.70E-04	9.64E-04	0.38393	0.70223
BI	0.01619	0.06266	0.2584	0.79688
$H_{Kyte-Doolittle}$	-0.01675	0.11812	-0.14179	0.88767

Figure S12 Multiparameter correlation of experimental and bioinformatic features of 81 peptides, which are not enhancing infectivity (**Table S5**, Infection Rel EF-C < 10%) conducted to check for overfitting. As expected, there are no statistically significant correlations found. **A** Multiple linear regression (constrained linear least-squares problems solution) with the equation: y = A0+A1*x1+A2*x2+[…]+ A5 * x5 and zeta-pot, Log10 Count Rate, Fibril formation, $H_{fauchere}$, ThT-activity and β-sheet as independent variables (A, listed in the order of ascending P variable) and Log10 Infection Relative EF-C as dependent target variable (y). Linear Fit with Pearson correlation factor 0.35. **B** Multiple linear regression (constrained linear least-squares problems solution) with the equation: y = A0+A1*x1+A2*x2+[…]+ A12 * x12. The independent variables (A) are all bioinformatic parameters and listed in the order of ascending P variable, **Table S4**. Log10 Infection Relative EF-C is the dependent target variable (y) and linear fit with Pearson correlation factor is 0.39. Significance level is determined via Pearson two-sided t-distribution test and indicated by p-values. Statistically significant parameters (p < 0.05) are highlighted. Source data for Figure S12 is provided.

6. Selected examples showcasing structure–property–activity relationship

The thresholds for classification were set as described in the following. Internal order was found if fibril formation is observed via TEM (**Figure S33**) and characteristic β-sheet peak at 1630 cm^{-1} is observed via FT-IR (**Figure S34**). Microscopic aggregation is positive for peptides showing count rate of scattered light above 100 kcps (**Table S5**) and observable aggregates via brightfield or fluorescence microscopy. The surface charge was assigned via the zeta-potential and considered as positive above a threshold of + 5 mV. Peptides were assigned to be infectivity enhancing if the Infection Rel EF-C at 1.3 µM is greater than 0.1 (> 4 fold of virus only infectivity).

Note, that since the selected thresholds are set arbitrarily and the infectivity enhancement of the peptides are continuous multiparameter dependent properties not all peptides can be covered in the illustrated schemes.

6.1 *N*-terminal cysteine leads to more aggregation and increases infectivity enhancement

The presence of *N*-terminal cysteine has a great impact on aggregation and infectivity enhancement as observed systematically in our library. Peptides which do not contain *N*-terminal cysteine at the first

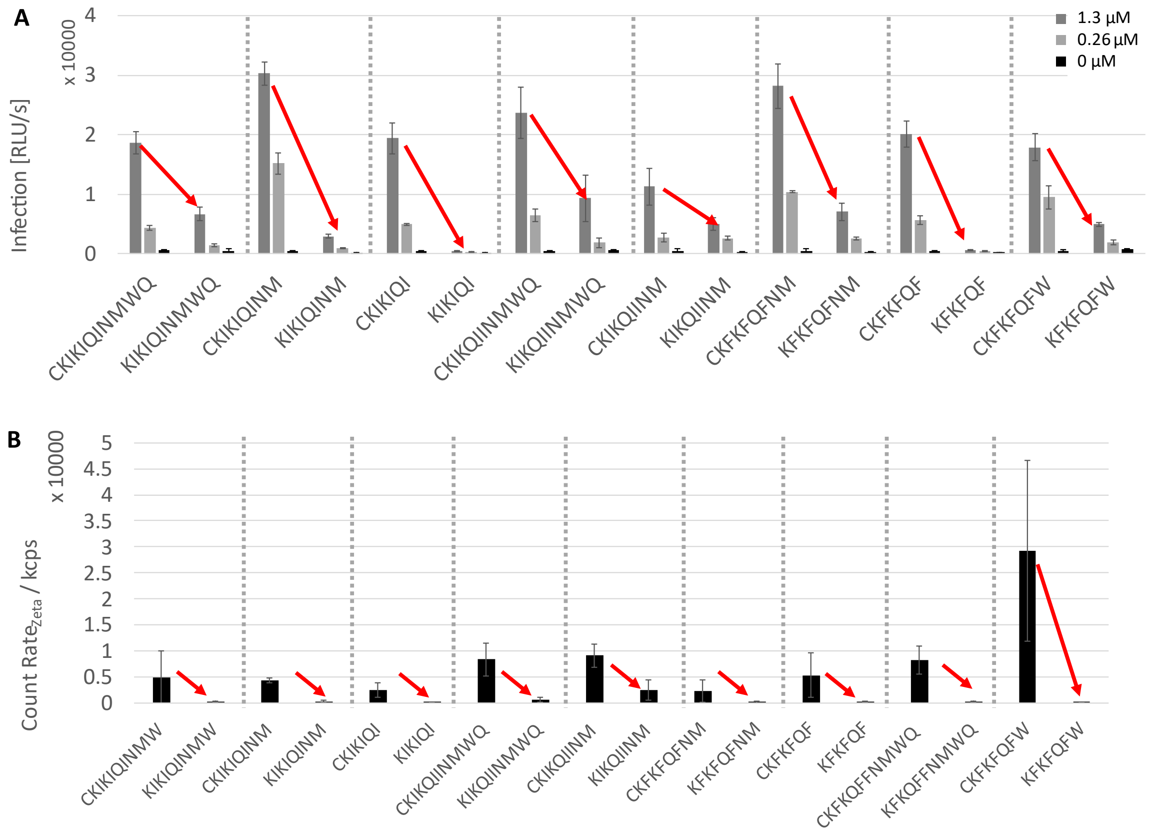

Figure S13 A Comparison of peptides' infection enhancement with and without *N*-terminal cysteine at conc. 1.3, 0.26 and 0 µM (**Table S5**). **B** Count Rate of scattered light of peptides with and without *N*-terminal cysteine (**Table S5**). Red arrows indicate the overall trend showing that infection enhancement and count rate decreases in the absence of an *N*-terminal cysteine. Error bars indicate standard deviation from triplicate measurements. Source data for Figure S13 is provided.

position (**Figure S13**) are less prone to aggregate into µm-sized particles and are inefficient at enhancing infectivity.

Comparing sequences CKIKIQI and KIKIQIC, which switch the *N*-terminal cysteine to the *C*-terminus show, a stronger infectivity enhancement for CKIKIQI (**Figure S14**A). Remarkably, there is no significant change in the intermolecular β-sheet content (ATR-IR **Figure S14**C). However, assembled fibrils of CKIKIQI aggregate more strongly (TEM **Figure S14**E), which results in larger numbers of µm-sized aggregates (ThT-stained peptides via fluorescence microscopy, Count rate of scattered light **Figure S14**B, D, F). We hypothesize, that the higher hydrophobicity of cysteine compared to lysine residues at the N-terminus facing outwards from the fibril, lead to higher fibril-fibril aggregation which might eventually also facilitate the interaction of virus and cells.

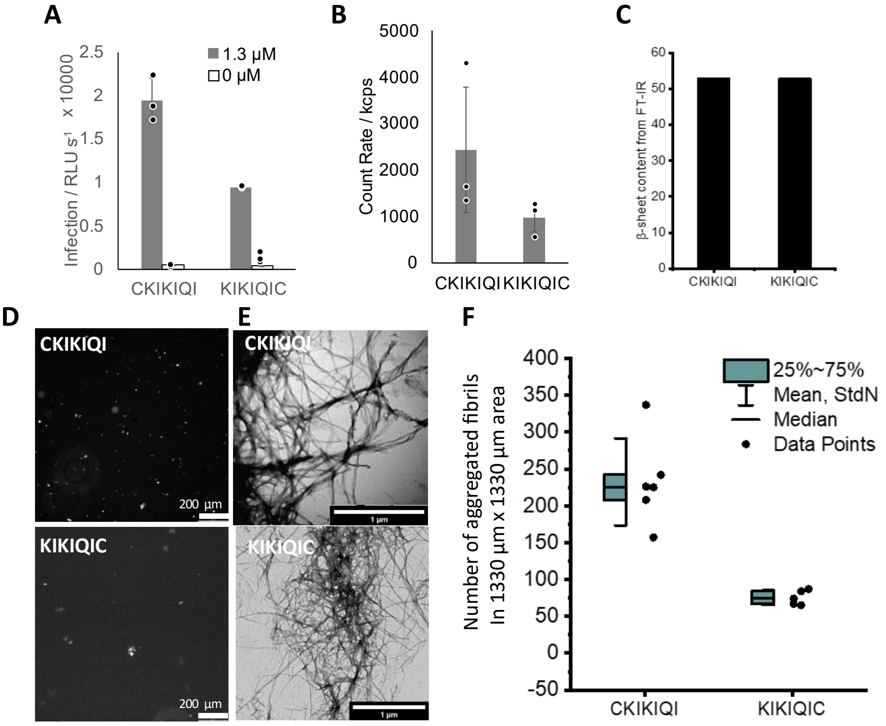

Figure S14 Comparing experimental data for CKIKIQI and KIKIQIC. **A** Infectivity enhancement, **B** Count Rate, **C** β-sheet content, **D** fluorescence microscopy of ThT-stained fibrils (scale bar 200 µm) and **E** TEM micrographs (scale bar 1 µm). **F** Number of ThT-stained µm-sized aggregates (>10 µm^2 in 1330 µm × 1330 µm) determined by fluorescence microscopy. Box plots show median with whiskers indicating mean value with standard deviations and boxes indicating 25-75% of all data displayed on the right side to each box. The bar plot error bars indicate std dev. from triplicate measurements. Source data for Figure S14 is provided.

6.2 Peptides with high hydrophobicity can enhance infectivity even if they have negative zeta-potential or do not form fibrils

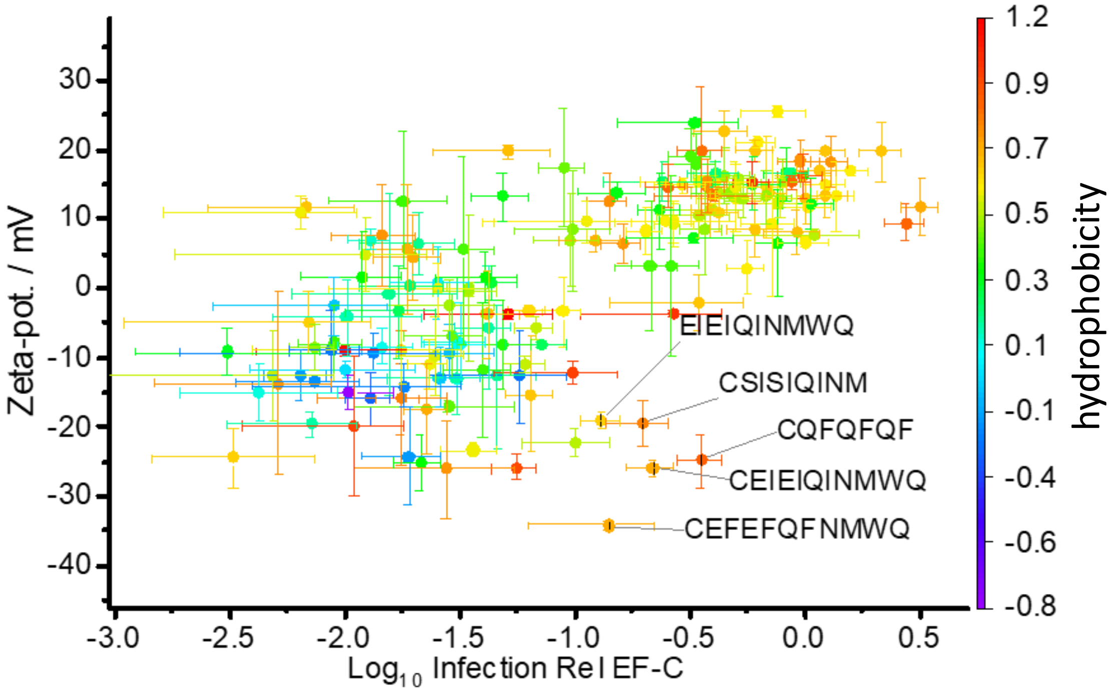

Figure S15 Peptides with negative zeta-potential (marked) slightly enhance infectivity if they have high (>0.6) calculated hydrophobicity (Fauchère, **Table S4**) and fulfill other prerequisites such as fibril formation and microscopic aggregation (**Table S5**). Error bars indicate standard deviation from triplicate measurements. Source data for Figure S15 is provided.

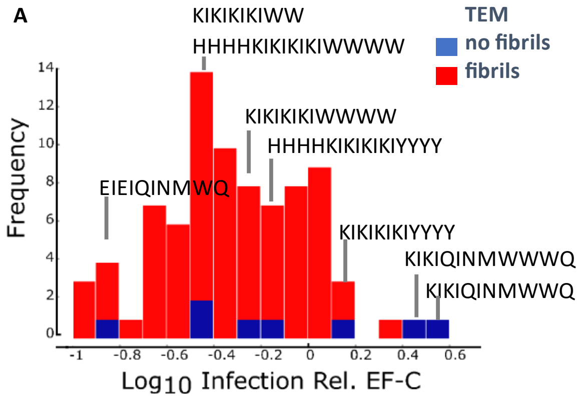

B

sequence	Log_{10} Infection Rel EF-C	Zeta Pot. / mV	Count Rate / kcps	β-sheet / %	Hydro phob.
KIKIQINMWWQ	0.51	11.8	3893	50	0.74
KIKIQINMWWWQ	0.44	9.45	7596	48	0.86
KIKIKIKIYYYY	0.14	13.3	9918	57	0.59
HHHHKIKIKIKIYYYY	-0.17	13.2	12793	53	0.48
KIKIKIKIWWWW	-0.23	15.2	12421	38	1.02
HHHHKIKIKIKIWWWW	-0.42	14.5	23757	49	0.80
KIKIKIKIWW	-0.43	15.4	9436	49	0.77
EIEIQINMWQ	-0.88	-19.13	1264	54	0.66

Figure S16 Peptides which are enhancing infectivity (> 10% relative to EF-C, 4-fold relative to virus only, **Table S5**), which do not form fibrils as analyzed by TEM (**Figure S33**). **A** Distribution plot of log_{10} Infection Rel EF-C > -1. Peptide sequences, which do not form fibrils (blue bars) are indicated. **B** Properties of indicated peptides: All peptides which do not form fibrils but are active, aggregate and show positive zeta-potential and have a high calculated hydrophobicity resulting from the high amount of hydrophobic amino acids (W or Y, > 20%) except for the peptide EIEIQINMWQ, which shows negative zeta-potential and with a log_{10} infectivity of -0.88 (13 % Rel EF-C) is only weakly active. Source data for Figure S16 is provided.

6.3 Peptides substituted with low hydrophobic amino acid alanine do not enhance infectivity

Substituting hydrophobic residues such as phenylalanine or isoleucine with alanine results in a dramatic loss of infectivity enhancement (**Figure S17**). As shown exemplarily for CKIKIQINMWQ and CKAKAQANMWQ, the alanine substitution results in loss of self-assembly capabilities and µm-sized aggregation which can be traced back to reduced hydrophobicity of the side chain residues (**Figure S18)**.

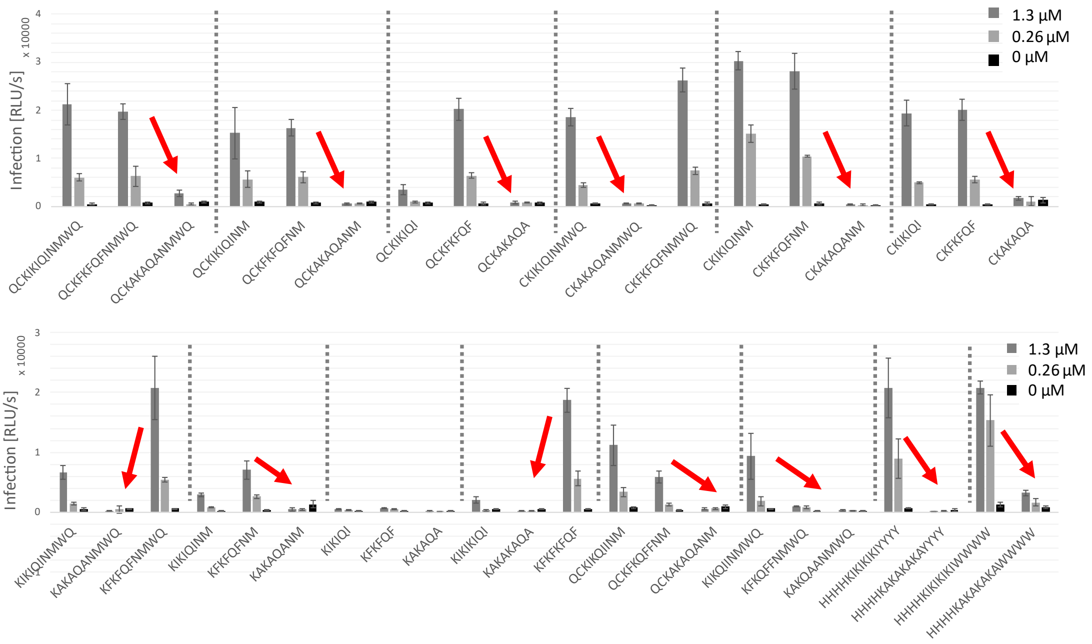

Figure S17 Infection rates of alanine, isoleucine and phenylalanine substituted amphiphilic peptide sequences at 1.3, 0.26 and 0 µM concentration. Dashed lines indicate one group of peptides, where the core sequence order stays same, arrows indicate the change of infection rates relative to 1.3 µM, phenylalanine derivatives. Error bars indicate standard deviation from triplicate measurements. Source data for Figure S17 is provided.

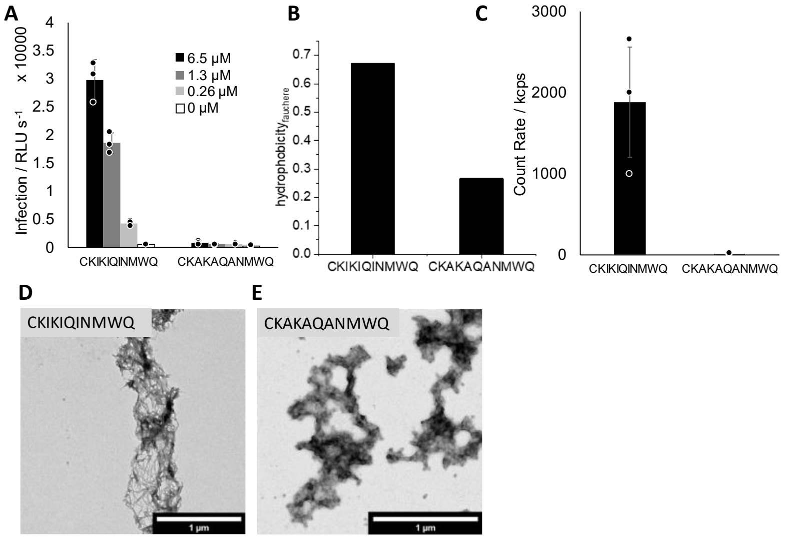

Figure S18 Comparing experimental data for CKIKIQINMWQ and CKAKAQANMWQ. **A** Infectivity enhancement, **B** Hydrophobicity calculated for the sequences according to Fauchère hydropathy scale, **C** Count Rate of scattered light **D** fibril morphology of CKIKIQINMWQ by TEM (scale bar 1 µm) and **E** amorphous aggregates morphology of CKAKAQANMWQ by TEM (scale bar 1 µm). Error bars indicate standard deviation from triplicate measurements. Source data for Figure S18 is provided.

The reduced aggregation propensity for alanine substituted peptides and its direct connection to loss in infectivity enhancement is further emphasized with the examples HHHHKIKIKIKIWWWW / HHHHKAKAKAKAWWWW (**Figure S19**A-G) and HHHHKIKIKIKYYYY / HHHHKAKAKAKAYYYY (**Figure S19**H-N), because other than aggregation and infectivity enhancement no other physicochemical properties are changing for these sequences.

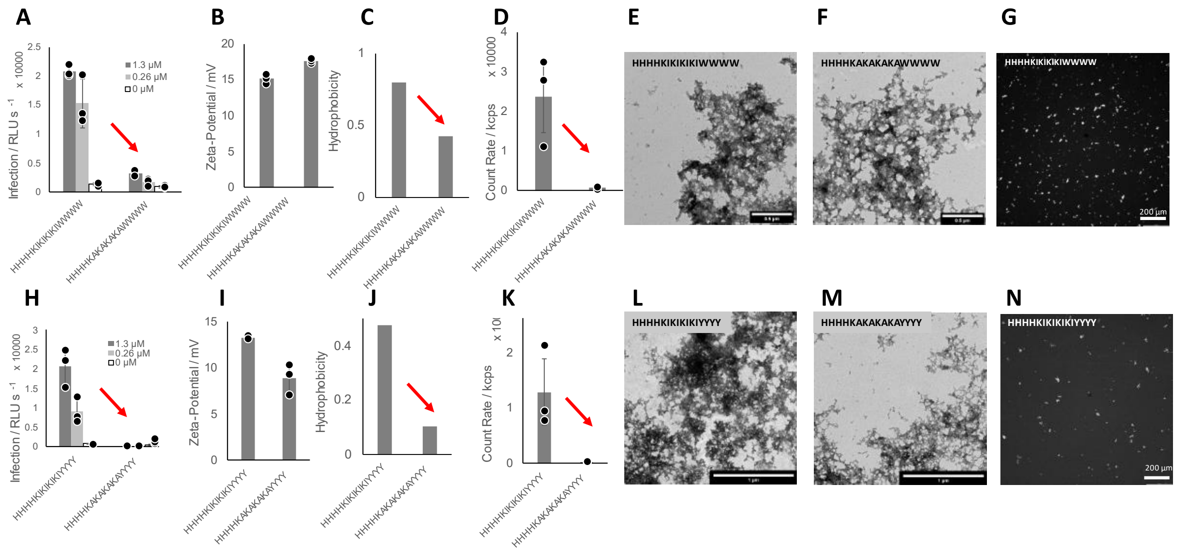

Figure S19 Alanine substitution reduces µm-sized aggregation and thus infectivity enhancement as shown exemplarily for HHHHKIKIKIKIWWWW / HHHHKAKAKAKAWWWW (A-G) and HHHHKIKIKIKYYYY / HHHHKAKAKAKAYYYY (H-N). **A, H** Infectivity data at 1.3 µM and 0.26 µM concentration, **B, I** zeta-potential, **C, J** Hydrophobicity calculated for the sequences according to Fauchère hydropathy scale, **D, K** Count Rate of scattered light **E, F, L, M** amorphous aggregate morphology by TEM (scale bar 1 µm) for HHHHKIKIKIKYYYY, HHHHKAKAKAKAWWWW, HHHHKIKIKIKYYYY and HHHHKAKAKAKAYYYY, respectively. **G, N** fluorescence microscopy of ThT-stained aggregates (scale bar 200 µm) for HHHHKIKIKIKIWWWW and HHHHKIKIKIKYYYY, respectively. Error bars indicate standard deviation from triplicate measurements. Source data for Figure S19 is provided.

6.4 *C*-terminal sequence extensions with hydrophobic amino acids increase infectivity enhancement

If an alternating amphiphilic core sequence, which does not self-assemble is extended with hydrophobic amino acids (I), (F), (W) at the *C*-terminus, fibril formation and infectivity enhancement are observed (**Figure S20**A, B, D). The introduction of hydrophobic amino acids in an already assembling, active sequence (CKIKIQI) is not significantly affecting self-assembly or activity (**Figure S20**C).

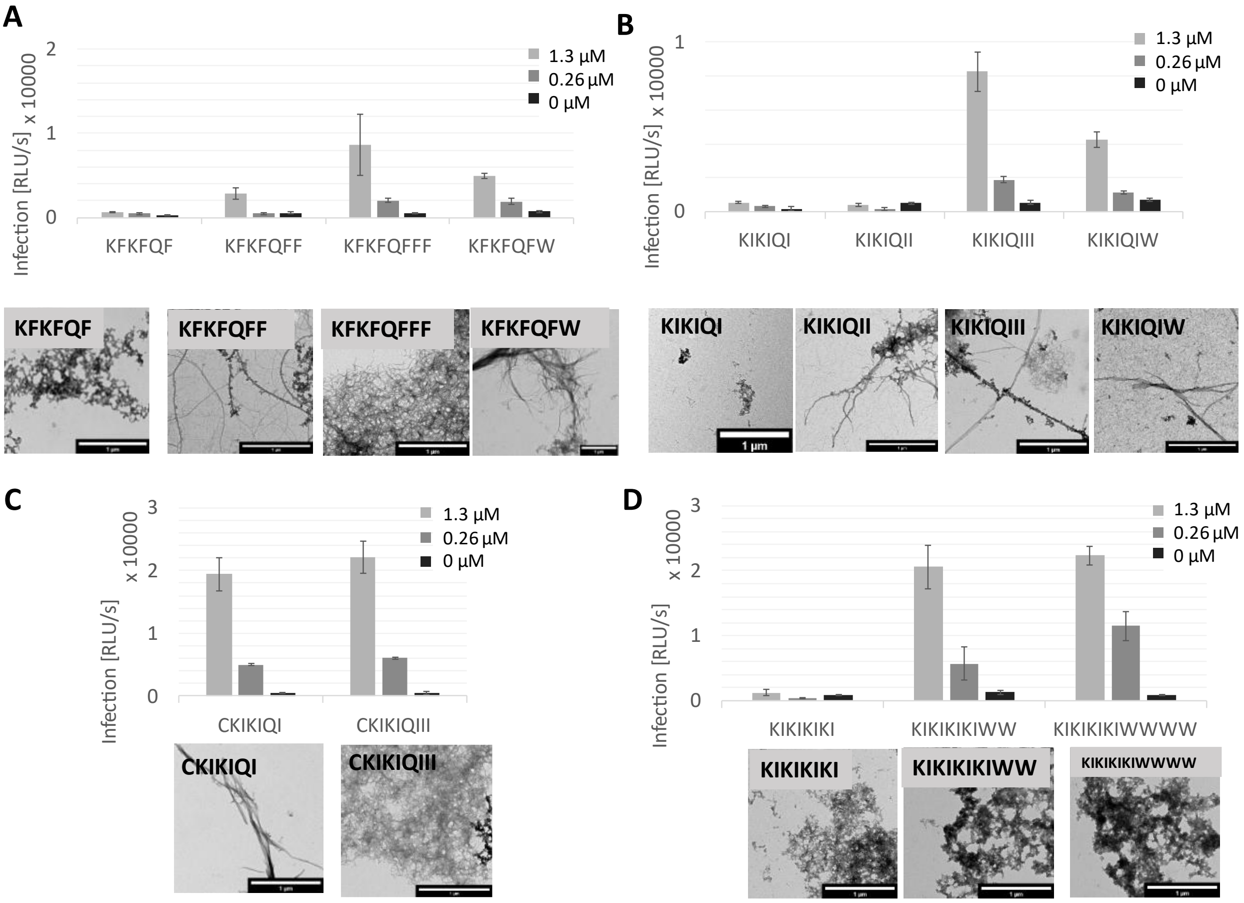

Figure S20 Hydrophobic amino acids at the *C*-terminus result in fibril formation and higher infectivity. Absolute Infection Rates of triplicate measurements of peptide concentrations at 1.3, 0.26, 0 µM. TEM measurements scale bar = 1µm. **A** Extension of the sequence KFKFQF with (F) and (W). **B** Extension of the sequence KFKFQF with (I) and (W). **C** Extension of the sequence CKIKIQI with (I). **D** Extensions of the sequence KIKIKIKI with (W). Error bars indicate standard deviation from triplicate measurements. Source data for Figure S20 is provided.

6.5 The chaotropic substance EGCG changes physicochemical properties and highlights importance of surface charge and internal order

The addition of chaotropic agents can disrupt the β-sheet structure in amyloids.[24,25] This leads to a loss of transduction enhancing effects as was investigated through the addition of epigallocatechin gallate (EGCG) to the peptide CKFKFQF (**Figure S21**A, B) — well in accordance with previous findings for EF-C and SEVI.[26,27] While cell-viability stays remains unaffected by the addition of EGCG to CKFKFQF (data not shown), the fibrillar morphology is lost (**Figure S21**D), which is well in line with previous findings from other groups.[27] Also the intermolecular structure is changing as shown by ATR-IR measurements in which an increase of unordered structural elements can be identified (**Figure S21**C). Beside the loss of fibrillar morphology, the strongest change in physicochemical properties is the decrease of zeta-potential. However, the microscopically formed aggregates do not disappear after the addition of EGCG as clearly visible in brightfield microscopy (**Figure S21**D) and by the negligible change in scattered light count rate (**Figure S21**F). This observation was also previously observed for $PAP_{248–286}$, an amyloidal peptide, showing that addition of chaotropic substances decrease internal order but not microscopic aggregation.[28] These experiments underline that the transduction enhancing properties result synergistically from fibril formation, a strong positive zeta-potential and aggregation propensity into µm-sized particles.

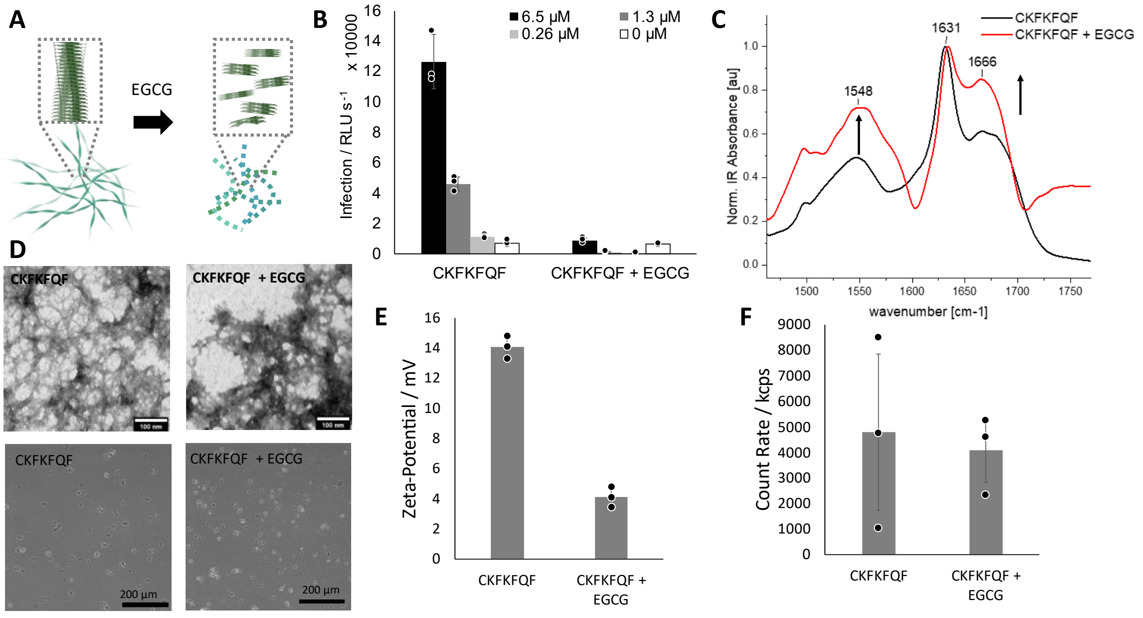

Figure S21 Effect of addition of chaotropic substance epigallocatechin-3-gallate (EGCG) to transduction enhancing peptide, shown exemplarily for CKFKFQF. EGCG is added as one molar equivalents to the preassembled peptide fibrils. **A** Schematic visualization of amyloid fibril disruptive property of EGCG. Created with Biorender.com **B** Infection rates for CKFKFQF with and without EGCG addition. **C** ATR-FT-IR measurement show an increase of unordered structural elements (1548 cm^{-1} and 1666 cm^{-1}) upon addition of EGCG. **D** TEM measurements of CKFKFQF before and after the addition of EGCG, scale bar 100 nm. Brightfield microscopy image of fibrillar aggregates before and after addition of EGCG, scale bar 200 µm. **E** Zeta-potential measurement before and after addition of EGCG. **F** No significant change is visible for the derived count rate of scattered light during DLS measurement for CKFKFQF (1 mg/mL, 1057 µM) with and without EGCG (1057 µM). Control indicates buffer with EGCG (1057 µM) only. Error bars indicate standard deviation from triplicate measurements. Created with BioRender.com. Source data for Figure S21 B, C, E, and F is provided.

7. Further information on pattern and amino acid analysis

To find recurring patterns in active peptides, the sequences in the library (**Table S3**) were simplified by coarse-graining the amino acids and the activity.[29] To this end, the amino acids were categorized in

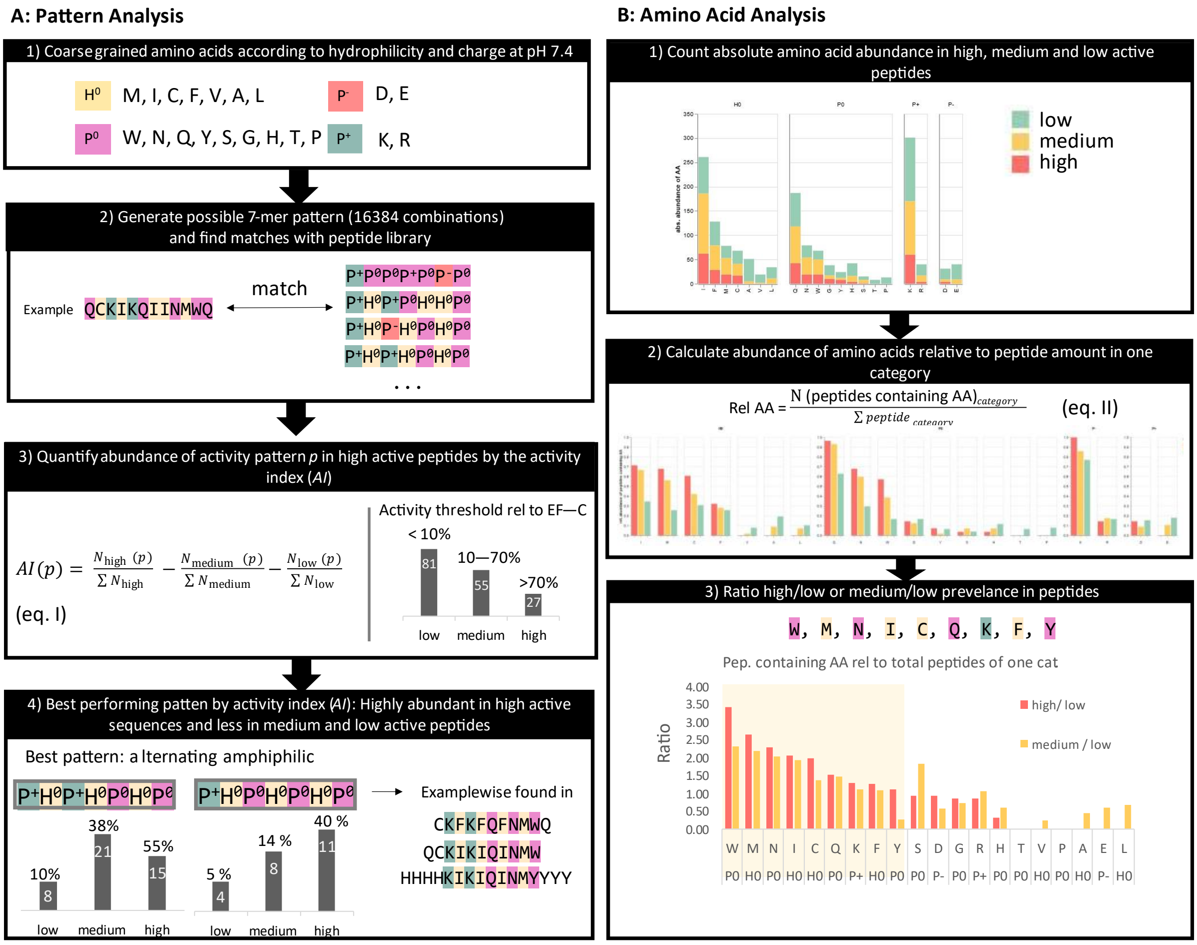

Figure S22 Schematic visualization of pattern analysis and amino acid analysis workflow. **A** Pattern analysis **A1)** First, amino acids are coarse-grained into 4 categories according to their hydrophilicity and charge at pH 7.4 by Kyte-Doolittle classification.[19] **A2)** The coarse-grained amino acids were combined into all possible 7-mer patterns (16384) and matched with existing patterns in the peptide library (163, **Table S3**) were counted. As shown exemplarily for the sequence QCKIKQIINMWQ (EF-C) for matching it is not important at which position in the sequence the pattern occurs. Therefore, several patterns can match one sequence. **A3)** To identify patterns, which are more prevalent in high active sequences compared to medium or low active sequences the activity index (AI, eq. I) was calculated. To this end, first the thresholds for activity were set relative to EF-C. Low active sequences (81 peptides) have an infectivity < 10% rel. to EF-C, which is below 4 fold virus only activity, medium active sequences (55 peptides) have an infectivity between 10 – 70% rel. to EF-C and high active sequences (27 peptides) are defined to have an infectivity enhancement >70% rel. to EF-C (>29 fold of virus only infectivity). For the calculation of the *AI* the relative number *N* of pattern *p* in high-active sequences were subtracted from the relative *N* of *p* in medium and low active sequences to find pattern which are mostly prevalent in high active sequences and less prevalent in medium and low. **A4)** The highest *AI* was found for the alternating amphiphilic pattern $P^+H^0P^+H^0P^0H^0P^0$ and $P^+H^0P^0H^0P^0H^0P^0$ which can be found in 55% and 40 % of all high-active peptides, respectively. **B** Amino acid analysis **B1)** To find amino acids mostly prevalent in high active peptides first the absolute abundance of amino acids in one category (low, medium, high) in the whole peptide library (163 sequences, **Table S3**) was determined. **2)** The relative abundance of amino acids (Rel AA, eq. II) was calculated by determining the number of peptides containing a certain amino acid relative to total amount of peptides of one category. **3)** W, M, N, I, C, Q, K, F, Y are more prevalent in high and medium active sequences than in low active sequences (highlighted box) as determined by the ratio Rel. AA high / low or medium / low > 1. Note, that all pattern and amino acid analyses were conducted with custom made python scripts. Source data for Figure S22 A4 and source code for Figure S22 A, B is provided.[30]

their two basic parameters, that are hydrophilicity and charge according to their side group properties by Kyte-Doolittle classification (H^0 hydrophobic non-charged, P^0 hydrophobic non-charged, P^- hydrophilic negatively charged, P^+ hydrophilic positively charged, **Figure S22**A).[19] The infectivity enhancement was categorized in low (< 4 fold virus only, < 10% relative to EF-C), medium (10 – 70% relative to EF-C), high (> 70% relative to EF-C). The coarse-graining approach simplifies and reduces the number of sequences to enable observation of recurring patterns while still considering the two important physicochemical properties hydrophilicity and charge.

In **Figure S22**A a schematic overview of the pattern and amino acid analysis is shown. For the identification of patterns, coarse-grained sequences from 2-mers to 7-mers were evaluated via a

A

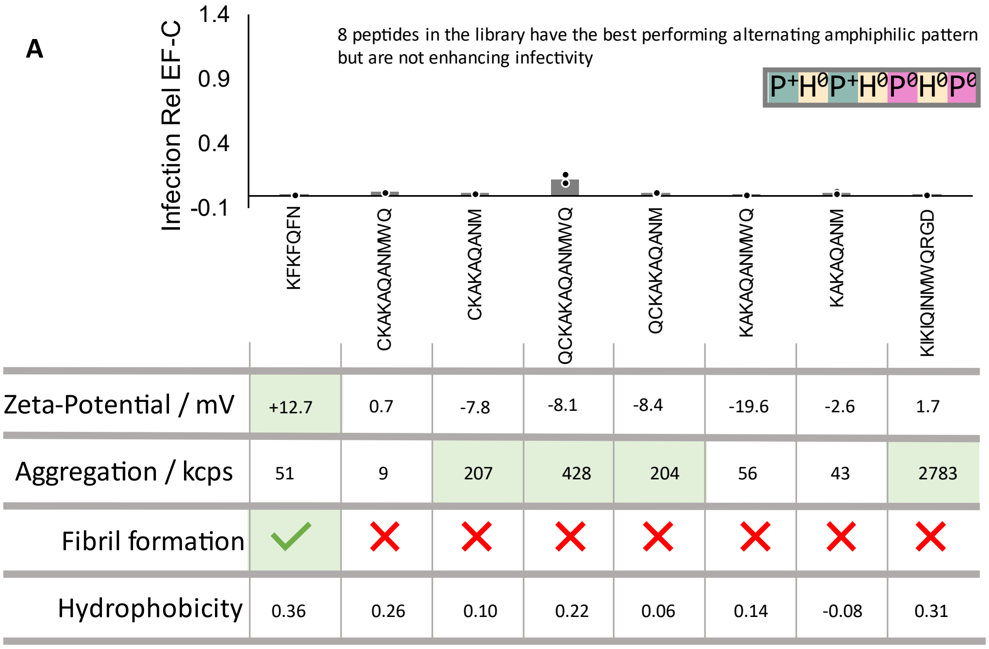

	KFKFQFN	CKAKAQANMWQ	CKAKAQANM	QCKAKAQANMWQ	QCKAKAQANM	KAKAQANMWQ	KAKAQANM	KIKIQINMWQRGD
Zeta-Potential / mV	+12.7	0.7	-7.8	-8.1	-8.4	-19.6	-2.6	1.7
Aggregation / kcps	51	9	207	428	204	56	43	2783
Fibril formation	✓	✗	✗	✗	✗	✗	✗	✗
Hydrophobicity	0.36	0.26	0.10	0.22	0.06	0.14	-0.08	0.31

B

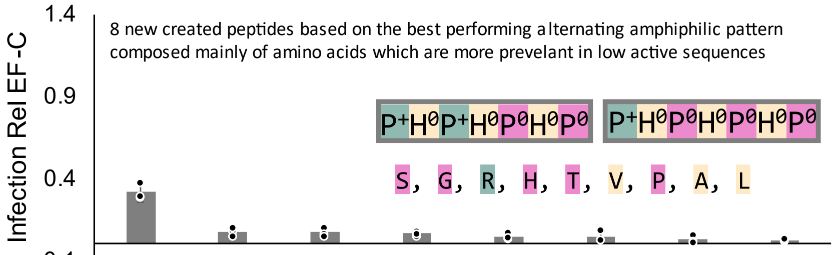

	CRLSLSLS	KAQCTLP	KAKLTCT	RVRVTVT	CRLRVGVS	KLKLTLT	CKFKFPF	RVHLGLG
Zeta-Potential / mV	+8.9	-8.5	-2.3	-3.7	+10.5	+1.7	-1.0	-6.0
Aggregation / kcps	1696	97	16	25	35	198	143	28
Fibril formation	✓	✗	✗	✗	✗	✓	✗	✓
Hydrophobicity	0.68	0.47	0.29	0.30	0.45	0.52	0.80	0.53

Figure S23 Peptides sharing the best performing pattern $P^+H^0P^+H^0P^0H^0P^0$ and $P^+H^0P^0H^0P^0H^0P^0$, which are not enhancing infectivity. **A** 8 of the peptides in the library have the pattern $P^+H^0P^+H^0P^0H^0P^0$ but do not enhance infectivity (**Table S6**). **B** Creating peptides based on the identified patterns $P^+H^0P^+H^0P^0H^0P^0$ and $P^+H^0P^0H^0P^0H^0P^0$ but mainly composed of amino acids prevalent in low active sequences result in weakly active peptides (**Table S6**). Fibril formation determined via TEM (**Figure S25**). Error bars indicate standard deviation from triplicate measurements. Source data for Figure S23 A, B is provided.

python script. A sequence length of 7-mer was selected because all the infectivity enhancing peptides in the library contain more than 7 amino acids. The highest prevalence was found for the alternating amphiphilic pattern $P^+H^0P^+H^0P^0H^0P^0$ and $P^+H^0P^0H^0P^0H^0P^0$ which can be found in 55% and 40 % of all high-active peptides, respectively. Interestingly, the importance for a strictly alternating, amphiphilic pattern is more pronounced for short 7-mer sequences than for 9-mer or 11-mer sequences as shown in direct comparison for sequence scrambled, *C*-terminal truncated versions of (I) and (F) substituted peptides (**Figure S26**).

6 of the 8 peptides in the library which share the pattern $P^+H^0P^+H^0P^0H^0P^0$ but do not enhance infectivity are composed of less hydrophobic amino acids alanine, which drastically reduces hydrophobicity of the peptides, and the ability to form fibrils and aggregate into positively charged clusters. Alanine is not found in high active peptides (**Figure S22**B1) and also reduces infectivity if applied in newly created peptides (**Figure S23**B**)**. The other two peptides in the library have the pattern $P^+H^0P^+H^0P^0H^0P^0$ but do not fulfill the key physicochemical properties for infectivity enhancement, that is for KFKFQFN aggregation into µm-sized aggregates and for KIKIQINMWQRGD fibril formation and a positive zeta-potential (**Figure S23**A**, Table S6)**.

By applying the amino acid analysis (**Figure S22**B) we find the amino acids W, M, N, I, C, Q, K, F and Y present in a higher frequency in highly active sequences "active amino acids" compared to amino acids S, G, R, H, T, V, P, A and L which are predominantly present in weakly active peptides "inactive amino acids" (**Figure S22**B). Interestingly, most of the "active amino acids" W, M, I, C, Q, F and Y are known to promote β-sheet amyloid fibril formation except of N, K (β-sheet breaking), whereas the "inactive amino acids" are either neutral (R, A, D, G) or β-sheet breaking (E, H, P, S) except of T, L, V (β-sheet promoting).[31,32]

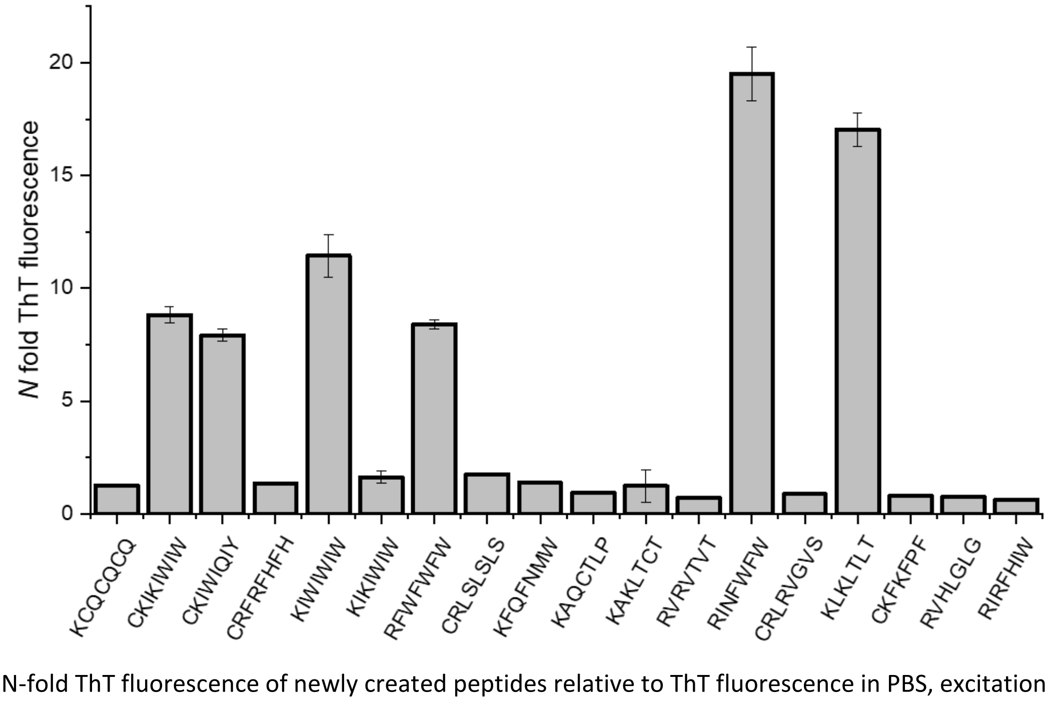

Figure S24 N-fold ThT fluorescence of newly created peptides relative to ThT fluorescence in PBS, excitation 440/10 nm, emission 488/10. The sequences are based on preserved sequence pattern $P^+H^0P^+H^0P^0H^0P^0$ and $P^+H^0P^0H^0P^0H^0P^0$ and listed according to their activity. Error bars indicate standard deviation from triplicate measurements. Source data for Figure S24 is provided.

By applying amino acids prevalent in low active sequences show, sequences are created that also have low activity, despite using high active patterns. The only exception to this trend was observed for the peptide CRLSLSLS (medium active) (**Table S6**, **Figure S23**B**)**. Note, that the amino acids V, L, S, T, P are not strongly represented in the peptide library, which may distort the reliability for classification of these amino acids (**Figure S22**B1). All the non-active sequences created by applying the best performing pattern but mainly composed with amino acids prevalent in low active sequences, as expected, do not fulfill one or more key-physicochemical property (**Figure S23**B**)**. Remarkably, these non-active sequences show on average also lower sequence hydrophobicity compared to the positive examples (**Figure 6**B**, Table S6**). Thus, they demonstrate that not only the sequence pattern, but also physicochemical properties originating from the selected amino acids, play a role in creating active peptides.

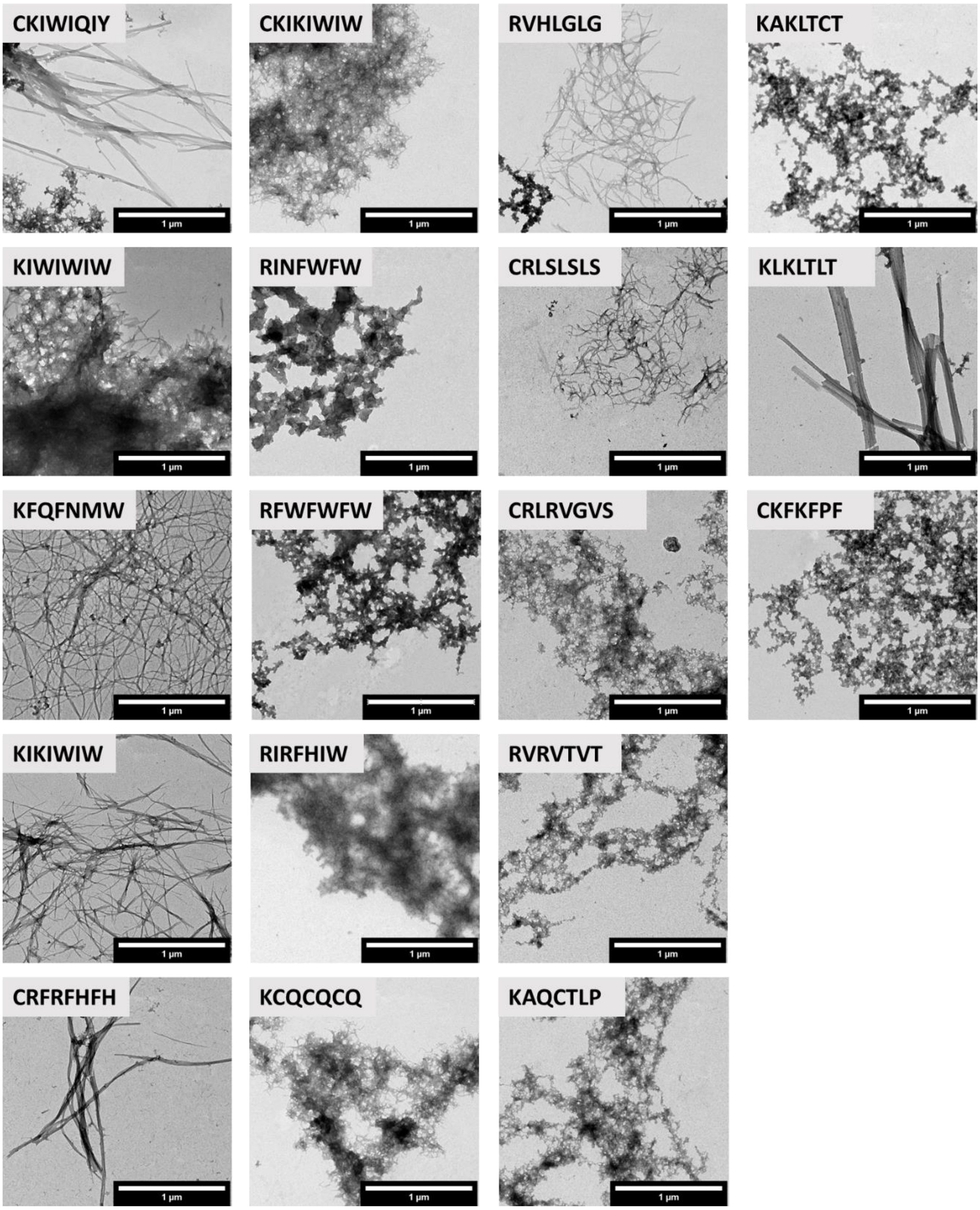

Figure S25 TEM micrographs for all newly created peptides based on best performing sequence pattern $P^+H^0P^+H^0P^0H^0P^0$ or $P^+H^0P^0H^0P^0H^0P^0$, scale bar 1 µm. TEM measurements were conducted once with at least three microscopy images recorded for each peptide sample.

Impact of sequence order is greater for shorter sequences compared to long sequences

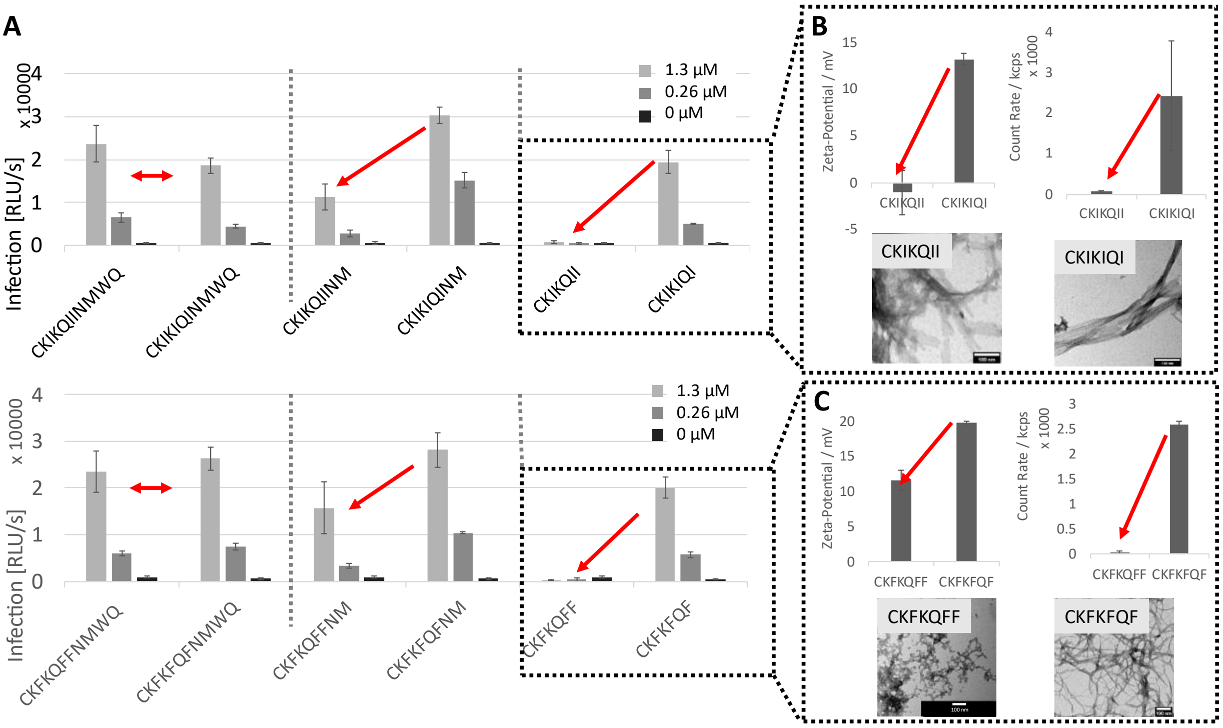

Figure S26 Impact of sequence order on infectivity enhancing properties. **A** Absolute infection of peptides at concentrations 1.3 µM, 0.26 µM and 0 µM of isoleucine (I) containing peptides which were scrambled (…QII…) → (…IQI…) and C-terminal truncated (Top) and phenylalanine (F) containing peptides which were scrambled (…QFF) → (…FQF…) and C-Terminal truncated (Bottom). With decreasing sequence length, the importance of alternating sequence order for infectivity enhancement is more pronounced (CKIKQII → CKIKIQI, CKFKQFF → CKFKFQF). **B, C:** Sequences with alternating sequence order have higher zeta-potential (left) and higher Count Rates (right), and form more ordered fibrils (bottom, TEM scale bar 100 nm). Error bars indicate standard deviation from triplicate measurements. Source data for Figure S26 is provided.

8. Amyloid peptides from functional and pathogenic contexts

Table S1 Selected amyloid peptides from protein data bases and literature reports. Note, that the peptide KLVFFAK, derived from amyloid ab was reported for retroviral transduction enhancement, however with very low efficiencies (> 4 fold compared to virus only).[33] "+" indicates reported ThT fluorescence (ThT) or fibril formation, "0" indicates no data reported. All peptides were synthesized via Fmoc SPPS protocol and purchased from Phtd Peptides industrial Co. limited with purity of ≥ 95%.

sequence	ThT	fibril	pI	charge	$h_{fauchere}$	derived from	original context	ref.
IKFLSVN	+	+	9.7	0.94	0.70	peroxiredoxin III (Prx III)	antioxidant enzyme	34
VQIVYK	+	+	9.3	0.94	0.67	tau 623–628	microtubule-associated protein	35
IIKIIK	+	+	10.8	1.94	0.87	S. aureus phenol-soluble modulin PSMα3	bacterial amyloid biofilm stability	36
GQTVTK	+	+	9.7	0.94	0.09	nucleoprotein of SARS-CoV-2	RNA binding protein	37
DFNKF	0	+	6.3	-0.06	0.24	human calcitonin hormone	medullary thyroid carcinoma	38
RCELARTLKR	0	+	11.3	2.87	0.09	human lysozyme 5–14	antimicrobial enzyme	39
ISKLEYSNFSVRY	0	+	9.1	0.94	0.39	Acyl phosphatase 86-98	hydrolase of acid anhydrides	39
GYVIIK	+	+	9.3	0.94	0.80	Serum amyloid P 213–218	associated with multiple sclerosis	35
KLVFFAK	+	+	10.6	2.00	0.69	Amyloid Aβ 16-22	Alzheimer's disease	33
RATVYV	+	+	9.3	0.94	0.49	VQIVYK (tau 623–628)	microtubule-associated protein	Waltz
RQGNINIVA	0	+	10.5	0.94	0.30	Insects' chorion S36	protective function in the eggshell	40
KYFIL	0	+	9.3	0.94	1.05	-	-	41
NKGAII	+	+	9.7	0.94	0.39	Alzheimer's Aβ 27-32	Alzheimer's disease	42
IIIQGK	0	+	9.7	0.94	0.69	-	-	43
LASLIYR	0	+	9.3	0.94	0.77	Mammalia melanocyte protein PMEL 610-616	control of melanin assembly	40
IIIK	0	+	9.7	0.94	1.10	-	-	44
MKVIFLKDVKG	0	+	10.5	1.94	0.47	N-terminal domain of ribosomal protein L9 (Beta 1)	protein synthesis	39
LANWMCLAKW	0	+	8.5	0.87	0.97	human lysozyme 25-34	antimicrobial enzyme	39
VHDCVNITIK	0	+	7.3	-0.02	0.56	human prion protein 176-185	High expression in nervous system, misfolding associated with neurodegenerative diseases	39
IFQINS	0	+	6.1	-0.06	0.755	human lysozyme	antimicrobial enzyme	45
NSGAITIG	0	+	13.8	1	0.44	Adenovirus Fiber Shaft type 2, 385 -392	receptor-binding head domain	46
GAITIG	0	+	13.8	1	0.69	Adenovirus Fiber Shaft type 2, 387 -392	receptor-binding head domain	46
VLYVGSKT	0	+	9.7	1	0.54	α-Synuclein 37–44	Parkinson's disease	47
GNNQQNY	+	+	5.9	0	-0.18	Yeast Protein Sup35 7-13	Terminate cellular translation	48

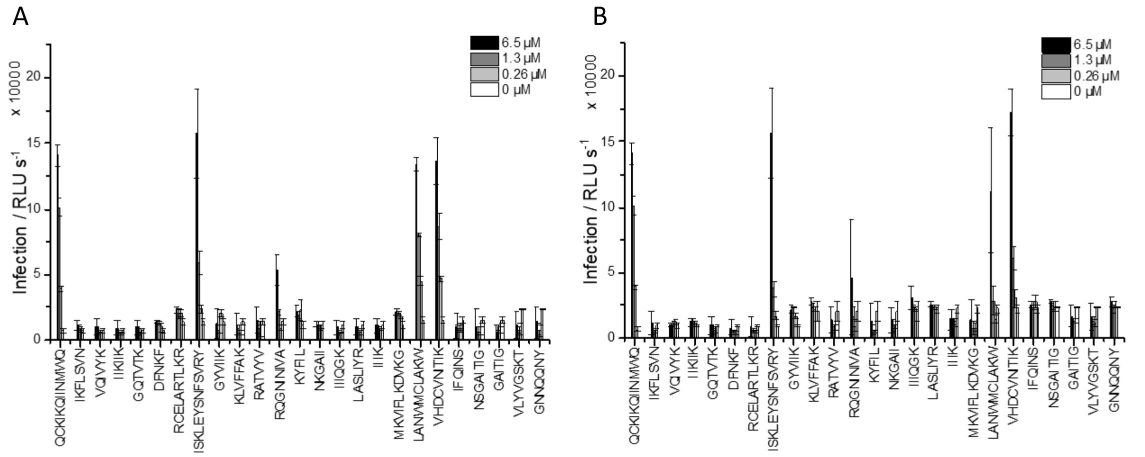

Figure S27 Overview of infectivity enhancing effect of amyloidal peptides derived from functional or pathogenic contexts. Absolute infection rates in RLU/s determined via β-galactosidase based luminescence assay for peptides at different concentration (6.5 µM, 1.3 µM, 0.26 µM) to TZM-bl cells and infected with HIV-1. 0 µM represents the virus only infectivity without the addition of peptide. **A** displays the infectivity rates for peptides prepared by diluting them from DMSO stock solution to PBS (1 mg/mL) and incubation for 1d at rt prior to dilution according to the final concentration (6.5 µM, 1.3 µM, 0.26 µM). **B** displays the infectivity rates for peptides prepared according to literature described procedure (**SI Section 8**). Error bars indicate standard deviation from triplicate measurements. Source data for Figure S27 is provided.

Preparation of peptide fibrils described in literature

Unless stated otherwise all peptides were incubated at rt for 1 w before characterization or evaluation in an infectivity assay. IKFLSVN was dissolved in 50 mM Tris buffer, pH 7.5 containing 100 mM NaCl to a final concentration of 14 mg/mL.[34] VQIVYK was dissolved in 50 mM PB, pH 7.4 to a final concentration of 1 mM.[49] IIKIIK was prepared as a 20 mM DMSO stock before diluting to 6 mM in ultrapure water.[36] GQTVTK was prepared as a 1582 µM solution using 20 mM Tris, 300mM NaCl, pH 8.0 buffer and incubated at 37°C for 1 d under constant agitation.[37] DFNKF was first dissolved as a 100 mM stock solution in DMSO and then diluted to 2 mM in 0.02 M NaCl, 0.01 M Tris, pH 7.2 to induce fibril formation.[50] RCELARTLKR was prepared as a 10 mg/mL in 50 mM PB, pH 7.2.[39] ISKLEYSNFSVRY was incubated in 25% (v/v) TFE, 50 mM in acetate buffer, pH 5.5 at a concentration 0.4 mg/mL.[51] GYVIIK was first prepared as a 4 mg/mL DMSO stock solution and then diluted in 100 mM MES buffer, pH 7.4 to a concentration of 1.3 mg/mL.[52] KLVFFAK was prepared as a 4 mg/mL solution in 50 mM PBS with 1 M NaCl, pH 2. The solution was constantly agitated for 3d at 37 °C.[33] RATVYV was dissolved in ultrapure water to a final concentration of 1 mM and was incubated for 4 weeks at 25°C.[53] RQGNINIVA was dissolved in water with adjusted pH 7.5 and incubated at a concentration of 0.5 mM for 4 weeks at rt.[2] KYFIL was prepared in PBS, pH 7.4 at a concentration of 3 % wt.[41] NKGAII was dissolved in 20 mM ammonium acetate buffer, pH 7.0 to the final concentration of 250 µM.[42] IIIQGK was dissolved in 25 mM Hepes buffer, pH 7.4 and incubated at a concentration of 8 mM.[43] LASLIYR was incubated in water, pH 7.5 at a concentration of 0.5 mM at rt for 4 weeks.[2] IIIK was dissolved in water to a concentration of 4 mM and incubated for 1 week at rt.[44] MKVIFLKDVKG was prepared as 1 mM solution in water at pH 5.4.[54] LANWMCLAKW and VHDCVNITIK were diluted to a concentration of 1 mM in 50 mM PB, pH 7.2.[51] IFQINS was prepared at 1.5 mM in water, pH 6.5.[45] NSGAITIG was incubated at 10 mg/mL concentration in water for 6 weeks, GAITIG was incubated at 20 mg/mL concentration in water for 6 weeks.[46] VLYVGSKT[47] and GNNQQNY[48] were dissolved at 20 mg/mL concentration in water.

Note, that for 20 of the 24 selected peptides fibril formation under the literature described preparation protocol was observed via TEM (**Figure S37**). Noteworthy, 12 of the selected peptides formed also fibrils upon dilution from DMSO stock solutions in PBS to 1 mg/mL (**Figure S36**). For the infectivity assay, the peptide fibrils were diluted in phosphate-buffered solution regardless of the initial preparation protocol. No significant difference in activity was observed for both preparation methods (**Figure S27**).

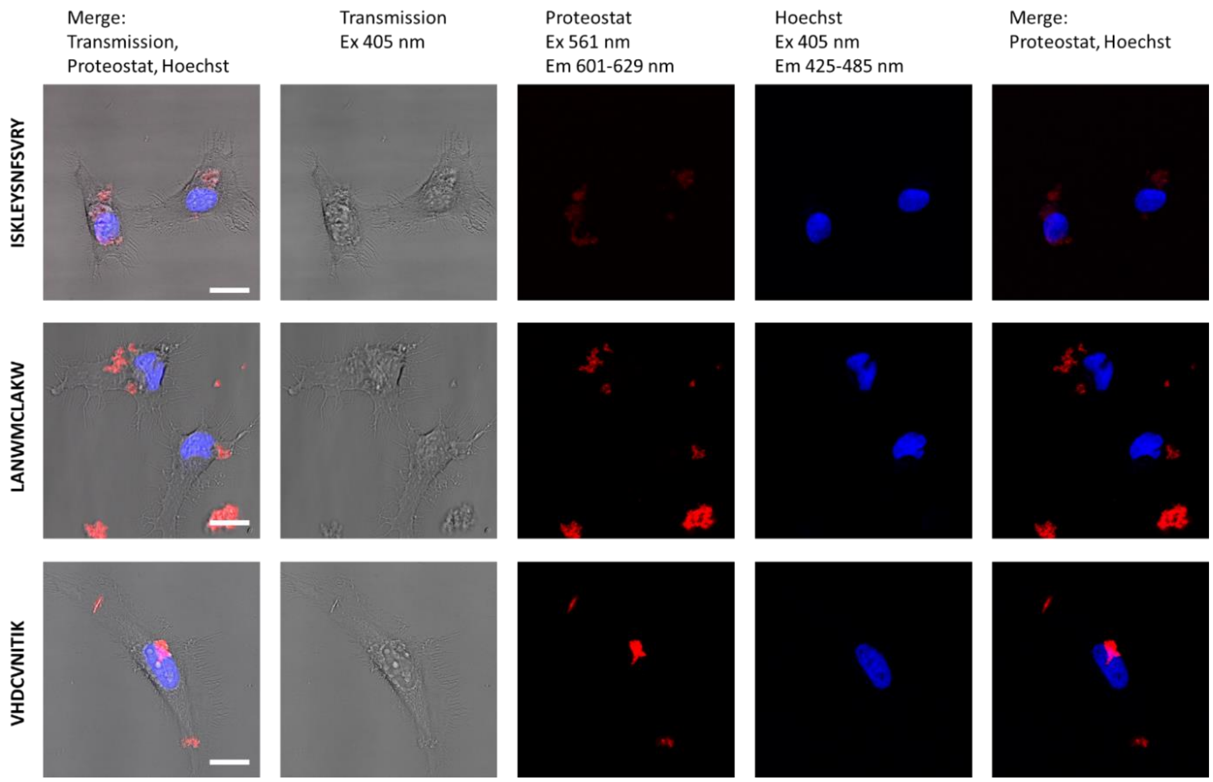

Figure S28 Fibril-cell colocalization shown by confocal microscopy. Acyl phosphatase derived peptide ISKLEYSNFSVRY, human lysozyme derived peptide LANWMCLAKW and human prion protein derived peptide VHDCVNITIK were added to HeLa cells. To this end, 20 µg/mL preassembled peptides (stained with Proteostat, red) were incubated 30 min with HeLa cells (nucleus stained with Hoechst, blue), washed three times with PBS and incubated for further 30 min before imaging, scale bar 20 µm. Note, that ISKLEYSNFSVRY cannot be stained as strongly fluorescent as the other two peptides (**Figure S29**A) therefore it also appears weaker fluorescent in the confocal microscopy. Confocal images have been prepared in biological triplicates and recorded at least at three different sites.

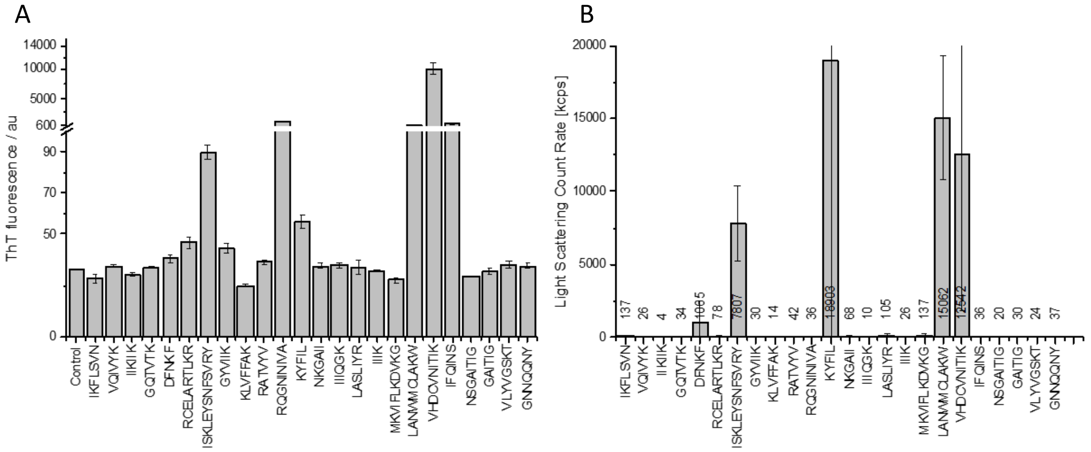

Figure S29 Thioflavin-T (ThT) fluorescence and Count Rate of scattered light of literature known peptides prepared by diluting from 10 mg/mL DMSO stock solution in PBS (1 mg/mL). **A** The ThT-Assay was conducted by adding ThT (50 µM in PBS) to the peptide in a 1:5 (peptide: ThT, v:v) ratio. The control is the background fluorescence of ThT without peptide (50 µM ThT added to PBS with 10% DMSO in 1:5 (PBS: ThT, v:v) ratio). **B** The derived count rate of scattered light was determined at 633 nm, 173° angle for peptides diluted from PBS (1 mg/mL) in KCl (1mM) to a final concentration of 0.1 mg/mL. Error bars indicate standard deviation from triplicate measurements. Source data for Figure S29 is provided.

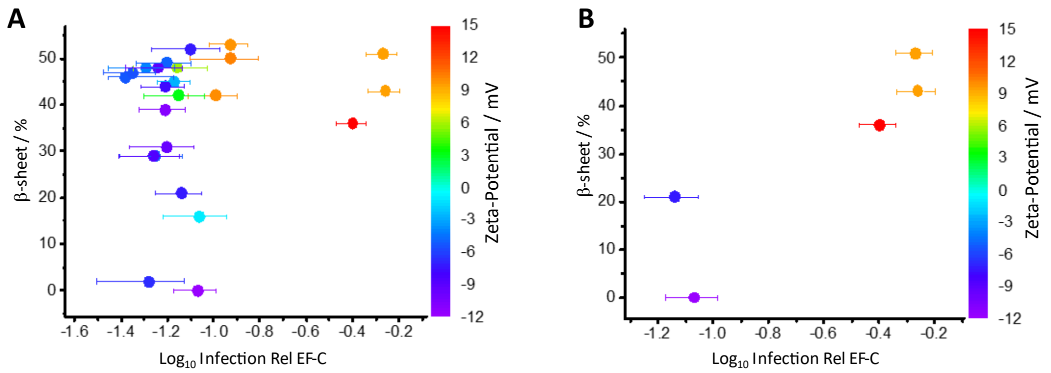

Figure S30 β-sheet content plotted against Log Infection Rel EF-C of 24 literature reported peptides. Color scheme represents zeta-potential. **A** All peptides regardless of the count rate are shown. **B** Peptides above a critical count rate of 100 kcps is shown. All tested peptides with a high β-sheet content and a positive zeta-potential are enhancers of viral transduction (top right corner). Error bars indicate standard deviation from triplicate measurements. Source data for Figure S30 is provided.

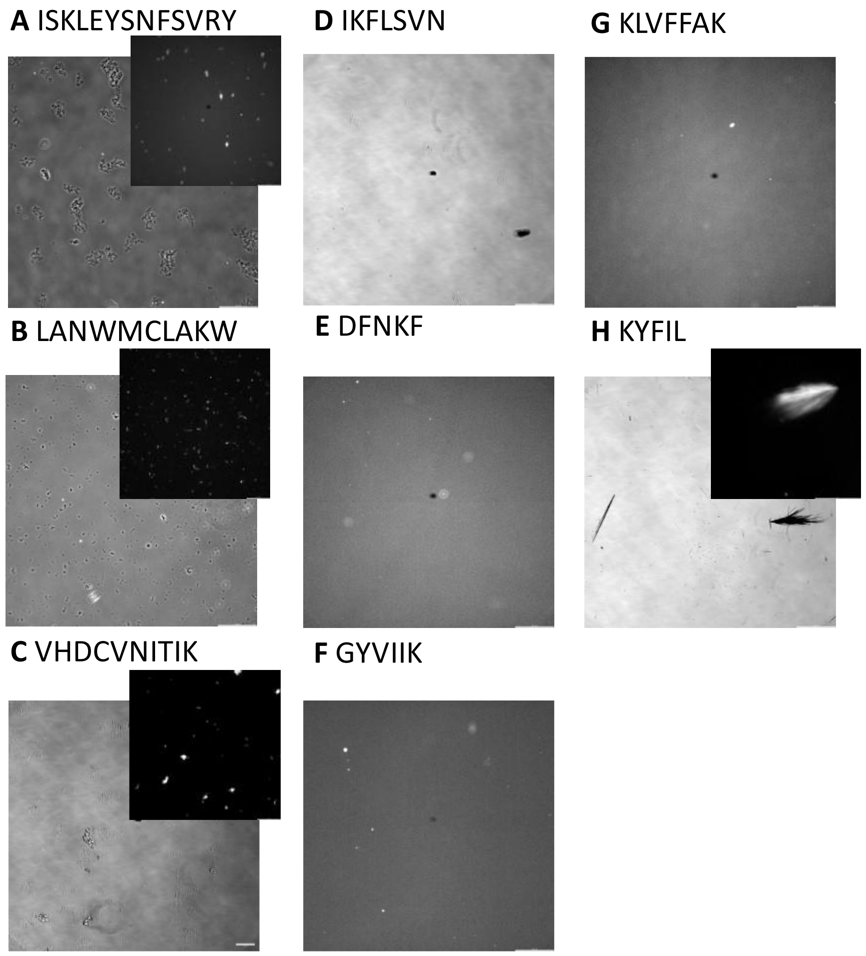

Figure S31 Brightfield and fluorescence microscopy images of selected peptides: **A-C** ISKLEYSNFSVRY, LANWMCLAKW and VHDCVNITIK as infectivity enhancing peptides show fluorescence upon addition of ThT (small image details) and microscopic aggregation (> 10 µm) as observed via brightfield microscopy. **D-G** IKFLSVN, DFNKF, GYVIIK, KLVFFAK do not enhance infectivity, are not ThT active (**Figure S29**) and do not aggregate microscopically in agreement with scattering results. **H** KYFIL is forming large fibrous aggregates, which are ThT active. This peptide is not enhancing infectivity due to its negative zeta-potential (scale bar 200 µm). Microscopy measurements were conducted once with at least three microscopy images recorded for each peptide sample.

9. Peptide library: tables

Table S2 EF-C derived peptides sorted according to their structural relationship.

N-Truncations	C-Truncations	Scrambling	Alternating	Substitutions
RGDSKIKIQIC	QCKFKFQFNMWQ	KAKAQANMWQ	QCKIKQIINM	QCKIKIQINMWQ
RGDKIKIQIC	QCKFKFQFNM	NMWQKAKAQA	QCKIKIQINM	QCKFKFQFNMWQ
KIKIQIC	QCKFKFQF			QCKAKAQANMWQ
		KFKFQFNMWQ	CKIKQIINM	
ILKNLSRSRKIKIQINMWQ	CKFKFQFNMWQ	NMWQKFKFQF	CKIKIQINM	QCKIKIQINM
RGDKIKIQINMWQ	CKFKFQFNMW			QCKFKFQFNM
QCKIKIQINMWQ	CKFKFQFNM	KIKQIINMWQ	KIKQIINM	QCKAKAQANM
CKIKIQINMWQ	CKFKFQF	NMWQKIKQII	KIKIQINM	
MKIKIQINMWQ				QCKIKIQI
KIKIQINMWQ	KFKFQFNMWQ	KIKIQINMWQ	QCKIKQII	QCKFKFQF
KIKIKIQINMWQ	KFKFQFNMW	NMWQKIKIQI	QCKIKIQI	QCKAKAQA
	KFKFQFNM			
CKIKIQINMW	KFKFQFN	KIKIQIC	CKIKQII	CKIKIQINMWQ
KIKIQINMW	KFKFQF	CKIKIQI	CKIKIQI	CKAKAQANMWQ
				CKFKFQFNMWQ
RGDCKIKIQINM	KFKFQFW	KIKIQINMWA	KIKIQI	CEFEFQFNMWQ
QCKIKIQINM	KFKFQF	KIKIQINMAQ	KIKQII	CEIEIQINMWQ
CKIKIQINM	KFKFQFFF	KIKIQINAWQ	KIKIQII	MKIKIQINMWQ
KIKIQINM	KFKFQFF	KIKIQIAMWQ	KIKIKIQI	
		KIKIQINMWQ		CKIKIQINMW
KIKIKIQI	QCKFKQFFNMWQ	KIKIQIMWNQ	KIKQIIN	CKFKFQFNMW
RGDKIKIQI	QCKFKQFFNM	KIKIQIMQWN	KIKIQIN	
QCKIKIQI	QCKFKQFF			CKIKIQINM
CKIKIQI		KIKIQIRGD	NMWQKIKQII	CKFKFQFNM
KIKIQI	CKFKQFFNMWQ	RGDKIKIQI	NMWQKIKIQI	CEIEIQINM
	CKFKQFFNM			CSISIQINM
QCKIKQIINMWQ	CKFKQFF	CKIKIQIRGD	CKIKQIIRGD	MKIKIQINM
CKIKQIINMWQ		RGDKIKIQIC	CKIKIQIRGD	CKAKAQANM
KIKQIINMWQ	QCKAKAQANMWQ			
	QCKAKAQANM	RGDKIKIQINMC	KAKQAANMWQ	CKIKIQI
QCKIKQIINM	QCKAKAQA	RGDCKIKIQINM	KAKAQANMWQ	CKWKWQW
CKIKQIINM				CKYKYQY
KIKQIINM	CKAKAQANMWQ	RGDKIKIQINMWQ	QCKFKQFFNMWQ	CRFRFQF
	CKAKAQANM	KIKIQINMWQRGD	QCKFKFQFNMWQ	CRFRVPF
QCKIKQII	CKAKAQA			CHLHLQL
CKIKQII			CKFKQFFNMWQ	CKFKFQF
KIKQII	KAKAQANMWQ		CKFKFQFNMWQ	CQFQFQF
	KAKAQANM			CSISIQI
QCKAKAQANMWQ	KAKAQA		KFKQFFINMWQ	MKFKFQF
CKAKAQANMWQ			KFKQFFNMWQ	CEIEIQI
KAKAQANMWQ	QCKIKIQINMWQ		KFKFQFNMWQ	CKAKAQA
	QCKIKIQINM			CEFEFQF
QCKAKAQANM	QCKIKIQI		QCKFKQFFNM	
CKAKAQANM			QCKFKFQFNM	KIKIQINMWQ
KAKAQANM	MKIKIQINMWQ			KAKAQANMWQ
	CKIKIQINMWQ		CKFKQFFNM	KFKFQFNMWQ
KAKAKAQA	CKIKIQINMW		CKFKFQFNM	EIEIQINMWQ
QCKAKAQA	CKIKIQINM			
CKAKAQA	CKIKIQI		QCKFKQFF	KIKIQINM
KAKAQA	CKIKIQIII		QCKFKFQF	KFKFQFNM
				KAKAQANM
QCKFKFQFNMWQ	KIKIQINMWQRGD		CKFKQFF	EIEIQINM
CKFKFQFNMWQ	KIKIQINMWQ		CKFKFQF	KIEIQINM
KFKFQFNMWQ	KIKIQINMWWWQ			EIKIQINM
	KIKIQINMWWQ		KFKFQFF	
QCKFKFQFNM	KIKIQINMW		KFKFQF	KIKIQI
CKFKFQFNM	KIKIQINM		KFKFQFFF	KAKAQA
KFKFQFNM	KIKIQIN			KFKFQF
	KIKIQI			KYKYQY
RGDCKFKFQF	KIKIQII			KWKWQW
QCKFKFQF	KIKIQIII			EFEFQF
CKFKFQF	KIKIQIW			
KFKFKFQF				KFKFQFW
KFKFQF	RGDCKIKIQINM			KIKIQIW

	RGDKIKIQINMC			
QCKFKQFFNMWQ	RGDKIKIQINM			KIKIQIC
CKFKQFFNMWQ	RGDKIKIQINMWQ			HIHIQIC
KFKQFFNMWQ				RLRLTLC
	RGDKIKIQI			
QCKFKQFFNM	RGDKIKIQIC			KIKIKIQI
CKFKQFFNM				KAKAKAQA
	KIKIKIKIWWWW			KLKLKLQL
QCKFKQFF	KIKIKIKIWW			KFKFKFQF
CKFKQFF	KIKIKIKI			KVKVKVQV
KLKLQLL	QCKIKQIINMWQ			KIKIKIKI
KLKQLL	QCKIKQIINM			EIEIEIEI
	QCKIKQII			KFKFEFEF
KIKIKIKIYYYY				
HHHHKIKIKIKIYYYY	CKIKQIINMWQ			
	CKIKQIINM			QCKIKQIINMWQ
CKFKFQFW	CKIKQII			QCKFKQFFNMWQ
KFKFQFW				
	KIKQIINMWQ			QCKIKQIINM
	KIKQIINM			QCKFKQFFNM
	KIKQIIN			QCKAKAQANM
	KIKQII			
				QCKIKQII
	CEIEIQINMWQ			QCKFKQFF
	CEIEIQINM			
	CEIEIQI			CKIKQIINMWQ
				CKFKQFFNMWQ
	EIEIQINMWQ			
	EIEIQINM			CKIKQIINM
				CKFKQFFNM
	RGDKIKIQINM			
	RGDKIKIQINMC			CKIKQII
				CKFKQFF
	KFKFQFFF			
	KFKFQFF			KIKQIINMWQ
				KFKQFFNMWQ
	KIKIQIII			KAKQAANMWQ
	KIKIQII			KFKQFFINMWQ
	KVKVQVVV			KIKQII
	KVKVQVV			KLKQLL
				KIKIQII
				KFKFQFF
				HLHLPLL
				KLKLQLL
				KVKVQVV
				KIKIQIII
			**Random Sequences**	KFKFQFFF
			HGDKCHGDKC	KVKVQVVV
			KKFQKKFQ	
			PPFHPPPFHP	RGECKFKFQF
			MDQMDQMDQMDQMDQ	RGDCKFKFQF
			FDPFDPFDP	
			TKTLTKTL	RGEKIKIQINM
			FKFDKFKFDK	RGDKIKIQINM
			KVKGVGK	
			SISISRRI	HHHHKIKIKIKIYYYY
			HRRHFRHKITKKK	HHHHKAKAKAKAYYYY
			KNERIKNERI	HHHHKIKIQINMYYYY
			KIRGKFEKED	
			HGDKCHGDKC	HHHHKIKIKIKIWWWW
			KKFQKKFQ	HHHHKAKAKAKAWWWW

Table S3 Overview of all peptides investigated in this study. Sequences indicated with references were reported previously by us (Schilling et al.[29] and Sieste et al.[55]) or are derived from literature reports. Sequences indicated with * have been synthesized by us (see **Figure S35** for LC-MS of peptides, which were not reported before by us) other sequences have been commercially obtained with purity of ≥ 95 % from Phtd Peptides industrial Co. limited.

Sequence	Report	Synthesis
CKIKIQIII	29	*
CEIEIQI	55	
CKIKQIINM	29	
CKFKFQFNMWQ	55	
CKFKFQFNM	55	
CKFKFQF	29	
KFKFQFNMW		*
KFKFQFNM	29	
KFKFQFN		*
CKAKAQANMWQ	55	
CKAKAQANM	55	
QCKFKQFFNMWQ		
QCKFKQFFNM		
QCKFKQFF		
CKFKQFFNMWQ		
CKFKQFFNM		
CKFKQFF		
QCKIKIQINM		
QCKAKAQANMWQ		
QCKAKAQANM		
QCKAKAQA		
QCKIKIQI		
QCKIKQIINM		
QCKIKQII		
QCKFKFQFNMWQ		
QCKFKFQFNM		
QCKFKFQF		
CRFRFQF		
HIHIQIC		
RLRLTLC		
HLHLPLL		
RGECKFKFQF		
RGEKIKIQINM		
KYKGAIIGNIK		
HGDKCHGDKC		
RPRGLLLGNLR		
KKFQKKFQ		
PPFHPPPFHP		
MDQMDQMDQMDQMDQ		
FDPFDPFDP		
TKTLTKTL		
FKFDKFKFDK		
KVKGVGK		
SISISRRI		
HRRHFRHKITKKK		
KNERIKNERI		
KIRGKFEKED		
CKFQC		
MKFM		
CKFC		
RGDKIRGDKI		
KNDKND		
HGEHGE		
HGEHGEHGE		
CRFRVPF		
CHLHLQL		
CETMYDKILKNLSRSR		
MKFKFQF	55	
QCKIKQIINMWQ	55	
QCKIKIQINMWQ	55	
KIKIQINMWQ	29	

NMWQKAKAQA		*
NMWQKFKFQF		*
KVKVKVQV		*
KIKQIINMWQ	55	
KAKAQANMWQ	55	
KFKFQFNMWQ	55	
KFKQFFNMWQ		*
KAKQAANMWQ		*
NMWQKVGTPL		*
NMWQKIKQII		*
NMWQKIKIQI		*
KIKQIINM	55	
KIKQIIN		*
KIKIQINMW		*
KIKIQINM	29	
KIKIQIN		*
KAKAQA	55	
KIKIQI	29	
KFKFQF	29	
KIKIQINMWA		*
EIEIEIEI		*
KIKIQINMAQ		*
KIKIQINAWQ		*
KIKIQIAMWQ		*
KIKIKIKIYYYY		*
HHHHEIEIEIEI		*
KYKYQY		*
EFEFQF		*
CEFEFQF		
CKFKFQFNMW		*
CKYKYQY		*
KWKWQW		*
CKWKWQW		*
KLKQLL		*
CKIKIQINMWQ	29	
RGDKIKIQI		*
CKIKIQINM	29	
CKIKIQI	29	
CKIKQII	29	
CKIKQIINMWQ	29	
KIKIQIRGD	29	*
RGDKIKIQIC	29	
RGDKIKIQINM	29	
RGDKIKIQINMWQ	29	*
KFKFEFEF		*
KIKQII	55	
KIEIQINM		*
CKIKIQIRGD	29	*
CKIKQIIRGD		
RGDKIKIQINMC	29	*
CQFQFQF		
KIKIQII		*
KFKFQFFF	29	*
KIKIKIQI	29	*
KAKAKAQA		*
KLKLKLQL	29	*
KFKFKFQF	29	*
KVKVQVV		*
KLKLQLL		*
KFKFQFF		*
KIKIQIII	29	*
KVKVQVVV		*
HHHHKAKAKAKAYYYY		*
KIKIQIC		
RGDSKIKIQIC		
RGDSGGGGGGKIKIQIC		
ILKNLSRSRKIKIQIC		
KIKIKIKIWWWW		*

KIKIKIKI		*
CEFEFQFNMWQ		
CSISIQI	55	
CEIEIQINMWQ	55	
CEIEIQINM	55	
CSISIQINM	55	
KAKAQANM	55	
CKAKAQA	55	
HHHHKIKIQINMYYYY		*
KIKIKIKIWW	29	*
HHHHKIKIKIKIWWWW	29	*
MKIKIQINM		
RGDCKIKIQINM		*
MKIKIQINMWQ		*
HHHHKIKIKIKIYYYY		*
RGDCKFKFQF		
KIKIQIW		*
KFKFQFW		*
CKFKFQFW		
EIKIQINM		*
IKVAVKIKIQINM		*
HHHHKAKAKAKAWWWW		*
CKIW		*
CKIKIQINMW		*
ILKNLSRSRKIKIQINMWQ		
EIEIQINM	29	
KIKIQINMWWQ		*
KIKIQINMWWWQ		*
KFKQFFINMWQ		*
KIKIQIMWNQ		*
KIKIQIMQWN		*
KIKIQINMWQRGD		*
EIEIQINMWQ		
KIKIKIQINMWQ		*
IKFLSVN	34	
VQIVYK	35	
IIKIIK	36	
GQTVTK	37	
DFNKF	38	
RCELARTLKR	39	
ISKLEYSNFSVRY	39	
GYVIIK	35	
KLVFFAK	33	
RATVYV	Waltz	
RQGNINIVA	40	
KYFIL	41	
NKGAII	42	
IIIQGK	43	
LASLIYR	40	
IIIK	44	
MKVIFLKDVKG	39	
LANWMCLAKW	39	
VHDCVNITIK	39	
IFQINS	45	
NSGAITIG	46	
GAITIG	46	
VLYVGSKT	47	
GNNQQNY	48	
CKIWIQIY		
KIWIWIW		
KFQFNMW		
KIKIWIW		
CKIKIWIW		
RVHLGLG		
CRLSLSLS		
CRLRVGVS		
RVRVTVT		
RINFWFW		

RFWFWFW		
RIRFHIW		
CRFRFHFH		
KAQCTLP		
KCQCQCQ		
KAKLTCT		
KLKLTLT		
CKFKFPF		

Bioinformatic descriptors

Table S4 Summary of bioinformatic descriptors calculated for the peptide library: Net charge (NC), hydrophobicity according to Fauchère scale ($H_{fauchere}$) and Kyte-Doolittle scale ($H_{Kyte-Doolittle}$), isoelectric point (pI), aliphatic index (AI), hydrophobic moment index (HMI), boman index (BI), instability index (II), graph shape index (GSI), upsilon steric parameter (USP), polarizability (P), normalized van der Waals Volume (Norm vdW Vol.).

Sequence	NC	$H_{fauchere}$	$H_{kyte-Doolittle}$	pI	AI	HMI	BI	II	GSI	USP	P	Norm. vdW Vol.
CKIKIQIII	1.87	0.93	1.52	9.68	216.67	0.14	-1.03	-19.41	3.12	0.86	0.19	3.99
CEIEIQI	-2.13	0.78	0.79	3.61	167.14	0.20	0.45	126.37	2.72	0.82	0.17	3.71
CKIKQIINM	1.87	0.60	0.34	9.68	130.00	0.51	0.54	16.33	2.63	0.81	0.18	3.92
CKFKFQFNMWQ	1.87	0.67	-0.58	9.68	0.00	0.18	1.27	-11.43	1.95	0.63	0.20	4.08
CKFKFQFNM	1.87	0.59	-0.22	9.68	0.00	0.12	1.19	-16.19	2.21	0.70	0.22	4.55
CKFKFQF	1.87	0.67	-0.06	9.68	0.00	0.21	0.92	-23.67	2.28	0.68	0.23	4.80
KFKFQFNMW	1.94	0.67	-0.60	10.81	0.00	0.24	1.07	-16.19	2.01	0.63	0.20	4.28
KFKFQFNM	1.94	0.48	-0.56	10.81	0.00	0.17	1.50	-19.46	2.26	0.71	0.23	4.82
KFKFQFN	1.94	0.37	-0.91	10.81	0.00	0.25	2.05	-23.67	2.25	0.70	0.23	4.87
CKAKAQANMWQ	1.87	0.27	-0.85	9.68	27.27	0.15	1.58	9.09	1.50	0.59	0.13	2.50
CKAKAQANM	1.87	0.10	-0.56	9.68	33.33	0.07	1.58	8.89	1.66	0.64	0.14	2.62
QCKFKQFFNMWQ	1.87	0.60	-0.83	9.68	0.00	0.46	1.62	3.81	1.92	0.64	0.19	4.07
QCKFKQFFNM	1.87	0.51	-0.55	9.68	0.00	0.40	1.63	2.57	2.14	0.70	0.22	4.49
QCKFKQFF	1.87	0.56	-0.49	9.68	0.00	0.42	1.50	0.71	2.19	0.68	0.22	4.69
CKFKQFFNMWQ	1.87	0.67	-0.58	9.68	0.00	0.46	1.27	10.10	1.95	0.63	0.20	4.08
CKFKQFFNM	1.87	0.59	-0.22	9.68	0.00	0.46	1.19	10.12	2.21	0.70	0.22	4.55
CKFKQFF	1.87	0.67	-0.06	9.68	0.00	0.54	0.92	10.16	2.28	0.68	0.23	4.80
QCKIKIQINM	1.87	0.52	-0.04	9.68	117.00	0.10	1.04	-24.01	2.52	0.79	0.18	3.93
QCKAKAQANMWQ	1.87	0.23	-1.08	9.68	25.00	0.15	1.91	2.88	1.50	0.59	0.13	2.63
QCKAKAQANM	1.87	0.07	-0.85	9.68	30.00	0.08	1.98	1.46	1.65	0.64	0.14	2.76
QCKAKAQA	1.87	0.01	-0.86	9.68	37.50	0.04	1.93	-0.68	1.56	0.61	0.13	2.52
QCKIKIQI	1.87	0.57	0.15	9.68	146.25	0.09	0.77	-32.51	2.66	0.80	0.19	3.98
QCKIKQIINM	1.87	0.52	-0.04	9.68	117.00	0.44	1.04	8.16	2.52	0.79	0.18	3.93
QCKIKQII	1.87	0.57	0.15	9.68	146.25	0.48	0.77	7.70	2.66	0.80	0.19	3.98
QCKFKFQFNMWQ	1.87	0.60	-0.83	9.68	0.00	0.18	1.62	-15.93	1.92	0.64	0.19	4.07
QCKFKFQFNM	1.87	0.51	-0.55	9.68	0.00	0.09	1.63	-21.11	2.14	0.70	0.22	4.49
QCKFKFQF	1.87	0.56	-0.49	9.68	0.00	0.07	1.50	-28.89	2.19	0.68	0.22	4.69
CRFRFQF	1.87	0.67	-0.23	10.53	0.00	0.24	3.59	-2.20	2.40	0.68	0.25	5.19
HIHIQIC	0.09	1.00	0.87	7.52	167.14	0.21	-0.17	151.74	3.13	0.82	0.19	3.96
RLRLTLC	1.87	0.70	0.60	10.53	167.14	0.23	2.34	36.09	2.46	0.77	0.20	4.18
HLHLPLL	0.16	1.11	1.03	7.71	222.86	0.13	-1.48	36.09	2.72	0.76	0.17	3.23
RGECKFKFQF	1.87	0.31	-0.88	9.67	0.00	0.33	2.72	14.34	1.98	0.61	0.21	4.35
RGEKIKIQINM	1.94	0.20	-0.71	10.79	106.36	0.18	2.45	-28.64	2.34	0.73	0.18	3.89
KYKGAIIGNIK	2.94	0.28	-0.18	10.64	115.45	0.24	0.45	18.16	2.19	0.64	0.15	3.26
HGDKCHGDKC	0.02	-0.02	-1.70	7.42	0.00	0.16	3.34	3.88	1.65	0.55	0.14	2.93

RPRGLLLGNLR	2.94	0.35	-0.38	12.80	141.82	0.28	2.71	21.82	1.97	0.61	0.16	3.15
KKFQKKFQ	3.94	-0.10	-2.13	11.28	0.00	0.71	3.42	8.75	2.07	0.69	0.23	4.85
PPFHPPPFHP	0.16	0.82	-1.04	7.71	0.00	0.08	0.34	99.54	2.79	0.28	0.10	0.48
MDQMDQMDQMDQMDQ	-5.06	0.08	-1.70	3.14	0.00	0.29	3.97	9.33	1.84	0.74	0.17	3.72
FDPFDPFDP	-3.06	0.58	-0.77	3.34	0.00	0.34	1.91	92.82	2.40	0.49	0.13	1.98
TKTLTKTL	1.94	0.31	-0.38	10.81	97.50	0.59	1.44	8.75	2.64	0.67	0.16	3.49
FKFDKFKFDK	1.94	0.17	-1.14	10.33	0.00	0.17	2.77	-13.36	2.25	0.70	0.22	4.82
KVKGVGK	2.94	-0.08	-0.59	11.10	82.86	0.52	0.96	-44.06	1.86	0.51	0.13	2.13
SISISRRI	1.94	0.41	0.26	12.50	146.25	0.60	3.16	104.43	2.65	0.75	0.17	3.63
HRRHFRHKITKKK	7.27	-0.21	-2.47	12.83	30.00	0.38	5.82	86.63	2.59	0.70	0.23	4.92
KNERIKNERI	1.94	-0.29	-2.18	10.77	78.00	0.19	5.80	0.51	2.32	0.76	0.20	4.33
KIRGKFEKED	0.94	-0.24	-1.98	9.44	39.00	0.25	4.51	2.79	1.99	0.66	0.18	4.07
CKFQC	0.79	0.73	0.08	8.23	0.00	0.25	1.11	-7.08	1.99	0.66	0.19	3.89
MKFM	0.94	0.82	0.68	9.70	0.00	0.61	-0.53	7.50	2.38	0.74	0.24	4.88
CKFC	0.79	0.97	0.98	8.23	0.00	0.54	0.00	7.50	2.09	0.66	0.19	3.88
RGDKIRGDKI	1.94	-0.19	-1.56	10.77	78.00	0.23	4.67	-41.94	2.00	0.63	0.16	3.54
KNDKND	-0.06	-0.79	-3.63	6.45	0.00	0.20	6.97	-5.82	1.70	0.73	0.15	3.50
HGEHGE	-1.83	-0.17	-2.37	5.33	0.00	0.22	3.51	-63.93	1.52	0.46	0.13	2.81
HGEHGEHGE	-2.72	-0.17	-2.37	5.32	0.00	0.17	3.51	-67.57	1.52	0.46	0.13	2.81
CRFRVPF	1.87	0.72	0.24	10.53	41.43	0.26	2.65	63.60	2.67	0.59	0.20	3.44
CHLHLQL	0.09	0.95	0.57	7.52	167.14	0.17	-0.17	101.71	2.44	0.81	0.19	3.96
CETMYDKILKNLSRSR	1.87	0.19	-0.90	9.40	73.13	0.78	3.39	86.63	2.21	0.72	0.18	3.90
MKFKFQF	1.94	0.63	-0.14	10.81	0.00	0.20	0.76	-23.67	2.36	0.70	0.24	5.08
QCKIKQIINMWQ	1.87	0.60	-0.40	9.68	97.50	0.50	1.14	8.47	2.23	0.72	0.17	3.60
QCKIKIQINMWQ	1.87	0.60	-0.40	9.68	97.50	0.19	1.14	-18.34	2.23	0.72	0.17	3.60
KIKIQINMWQ	1.94	0.59	-0.38	10.81	117.00	0.24	0.94	-16.47	2.34	0.73	0.17	3.68
NMWQKAKAQA	1.94	0.14	-1.19	10.81	30.00	0.32	1.87	9.00	1.47	0.58	0.13	2.51
NMWQKFKFQF	1.94	0.58	-0.89	10.81	0.00	0.37	1.52	-13.57	1.97	0.64	0.20	4.25
KVKVKVQV	2.94	0.21	0.20	11.10	145.00	0.10	0.75	-39.71	2.74	0.72	0.17	2.43
KIKQIINMWQ	1.94	0.59	-0.38	10.81	117.00	0.58	0.94	15.70	2.34	0.73	0.17	3.68
KAKAQANMWQ	1.94	0.14	-1.19	10.81	30.00	0.20	1.87	9.00	1.47	0.58	0.13	2.51
KFKFQFNMWQ	1.94	0.58	-0.89	10.81	0.00	0.22	1.52	-13.57	1.97	0.64	0.20	4.25
KFKQFFNMWQ	1.94	0.58	-0.89	10.81	0.00	0.54	1.52	10.11	1.97	0.64	0.20	4.25
KAKQAANMWQ	1.94	0.14	-1.19	10.81	30.00	0.41	1.87	32.68	1.47	0.58	0.13	2.51
NMWQKVGTPL	0.94	0.56	-0.46	9.70	68.00	0.54	0.57	-16.47	1.94	0.51	0.12	2.03
NMWQKIKQII	1.94	0.59	-0.38	10.81	117.00	0.68	0.94	15.70	2.34	0.73	0.17	3.68
NMWQKIKIQI	1.94	0.59	-0.38	10.81	117.00	0.39	0.94	-16.47	2.34	0.73	0.17	3.68
KIKQIINM	1.94	0.48	0.08	10.81	146.25	0.61	0.77	17.13	2.73	0.83	0.19	4.11
KIKQIIN	1.94	0.37	-0.19	10.81	167.14	0.72	1.22	18.14	2.79	0.84	0.19	4.06
KIKIQINMW	1.94	0.68	-0.03	10.81	130.00	0.25	0.43	-19.41	2.43	0.74	0.17	3.65
KIKIQINM	1.94	0.48	0.08	10.81	146.25	0.19	0.77	-23.09	2.73	0.83	0.19	4.11
KIKIQIN	1.94	0.37	-0.19	10.81	167.14	0.28	1.22	-27.81	2.79	0.84	0.19	4.06
KAKAQA	1.94	-0.21	-0.98	10.81	50.00	0.18	1.87	8.33	1.53	0.60	0.13	2.30
KIKIQI	1.94	0.53	0.37	10.81	195.00	0.28	0.31	-34.12	2.99	0.85	0.20	4.25

KFKFQF	1.94	0.53	-0.48	10.81	0.00	0.26	1.28	-29.28	2.36	0.69	0.25	5.19
KIKIQINMWA	1.94	0.64	0.15	10.81	127.00	0.24	0.20	-31.50	2.31	0.72	0.16	3.30
EIEIEIEI	-4.06	0.58	0.50	3.37	195.00	0.14	0.95	269.83	2.88	0.85	0.17	3.89
KIKIQINMAQ	1.94	0.39	-0.11	10.81	127.00	0.22	0.99	-4.13	2.47	0.78	0.18	3.69
KIKIQINAWQ	1.94	0.49	-0.39	10.81	127.00	0.24	0.99	-16.47	2.24	0.71	0.15	3.25
KIKIQIAMWQ	1.94	0.68	0.15	10.81	127.00	0.11	0.09	-16.47	2.31	0.71	0.16	3.40
KIKIKIKIYYYY	3.93	0.59	-0.23	10.20	130.00	0.21	0.26	-9.51	3.01	0.80	0.23	5.08
HHHHEIEIEIEI	-3.61	0.43	-0.73	5.32	130.00	0.17	2.18	183.22	2.91	0.80	0.19	4.15
KYKYQY	1.93	0.11	-2.53	9.74	0.00	0.15	2.84	-4.23	2.36	0.69	0.25	5.48
EFEFQF	-2.06	0.65	-0.35	3.61	0.00	0.23	1.70	-4.23	2.25	0.69	0.23	4.86
CEFEFQF	-2.13	0.77	0.06	3.61	0.00	0.19	1.28	-2.20	2.18	0.68	0.21	4.52
CKFKFQFNMW	1.87	0.76	-0.29	9.68	0.00	0.19	0.84	-13.57	1.99	0.63	0.20	4.10
CKYKYQY	1.86	0.32	-1.81	9.19	0.00	0.13	2.25	-2.20	2.28	0.68	0.23	5.05
KWKWQW	1.94	0.76	-2.33	10.81	0.00	0.21	1.61	8.33	0.89	0.34	0.10	2.25
CKWKWQW	1.87	0.87	-1.64	9.68	0.00	0.17	1.20	8.57	1.02	0.38	0.11	2.27
KLKQLL	1.94	0.48	0.02	10.81	195.00	0.62	0.31	19.50	2.19	0.83	0.20	4.25
CKIKIQINMWQ	1.87	0.67	-0.12	9.68	106.36	0.19	0.74	-14.06	2.29	0.72	0.17	3.57
RGDKIKIQI	1.94	0.16	-0.69	10.79	130.00	0.32	2.73	-38.28	2.43	0.73	0.17	3.82
CKIKIQINM	1.87	0.60	0.34	9.68	130.00	0.14	0.54	-19.41	2.63	0.81	0.18	3.92
CKIKIQI	1.87	0.68	0.67	9.68	167.14	0.23	0.09	-27.81	2.81	0.82	0.19	3.99
CKIKQII	1.87	0.68	0.67	9.68	167.14	0.60	0.09	18.14	2.81	0.82	0.19	3.99
CKIKQIINMWQ	1.87	0.67	-0.12	9.68	106.36	0.50	0.74	15.18	2.29	0.72	0.17	3.57
KIKIQIRGD	1.94	0.16	-0.69	10.79	130.00	0.24	2.73	-28.84	2.43	0.73	0.17	3.82
RGDKIKIQIC	1.87	0.30	-0.37	9.67	117.00	0.31	2.33	-33.45	2.36	0.72	0.17	3.68
RGDKIKIQINM	1.94	0.19	-0.71	10.79	106.36	0.18	2.62	-29.50	2.35	0.73	0.18	3.80
RGDKIKIQINMWQ	1.94	0.31	-0.94	10.79	90.00	0.31	2.47	-23.42	2.10	0.67	0.16	3.52
KFKFEFEF	-0.06	0.49	-0.45	6.53	0.00	0.15	1.60	-10.04	2.33	0.69	0.24	5.08
KIKQII	1.94	0.53	0.37	10.81	195.00	0.74	0.31	19.50	2.99	0.85	0.20	4.25
KIEIQINM	-0.06	0.52	0.13	6.41	146.25	0.20	0.93	77.14	2.69	0.83	0.18	3.99
CKIKIQIRGD	1.87	0.30	-0.37	9.67	117.00	0.19	2.33	-24.96	2.36	0.72	0.17	3.68
CKIKQIIRGD	1.87	0.30	-0.37	9.67	117.00	0.53	2.33	7.21	2.36	0.72	0.17	3.68
RGDKIKIQINMC	1.87	0.30	-0.44	9.67	97.50	0.26	2.30	-26.21	2.30	0.73	0.17	3.68
CQFQFQF	-0.13	0.89	0.06	5.92	0.00	0.19	0.91	-34.51	2.18	0.68	0.22	4.56
KIKIQII	1.94	0.71	0.96	10.81	222.86	0.29	-0.43	-27.81	3.16	0.87	0.19	4.21
KFKFQFFF	1.94	0.84	0.34	10.81	0.00	0.12	0.22	-19.46	2.51	0.69	0.26	5.37
KIKIKIQI	2.94	0.50	0.35	11.10	195.00	0.11	0.31	-44.31	3.00	0.85	0.20	4.28
KAKAKAQA	2.94	-0.24	-1.00	11.10	50.00	0.09	1.87	8.75	1.54	0.60	0.13	2.33
KLKLKLQL	2.94	0.45	0.00	11.10	195.00	0.10	0.31	-3.56	2.20	0.83	0.20	4.28
KFKFKFQF	2.94	0.50	-0.50	11.10	0.00	0.10	1.28	-38.25	2.37	0.69	0.25	5.23
KVKVQVV	1.94	0.38	0.79	10.81	165.71	0.25	0.07	-30.57	2.86	0.73	0.17	2.10
KLKLQLL	1.94	0.66	0.56	10.81	222.86	0.25	-0.43	18.76	2.24	0.85	0.19	4.21
KFKFQFF	1.94	0.71	-0.01	10.81	0.00	0.27	0.67	-23.67	2.44	0.69	0.25	5.29
KIKIQIII	1.94	0.85	1.40	10.81	243.75	0.12	-1.00	-23.09	3.29	0.89	0.19	4.19
KVKVQVVV	1.94	0.49	1.21	10.81	181.25	0.11	-0.45	-25.50	2.96	0.73	0.16	1.87

HHHHKAKAKAKAYYYY	4.38	0.10	-1.65	10.20	25.00	0.17	2.14	47.31	2.28	0.65	0.20	4.00
KIKIQIC	1.87	0.68	0.67	9.68	167.14	0.24	0.09	-27.81	2.81	0.82	0.19	3.99
RGDSKIKIQIC	1.87	0.27	-0.41	9.67	106.36	0.32	2.43	-4.27	2.27	0.70	0.16	3.49
RGDSGGGGGGKIKIQIC	1.87	0.17	-0.41	9.67	68.82	0.34	1.24	32.06	1.47	0.45	0.10	2.26
ILKNLSRSRKIKIQIC	4.87	0.39	-0.08	11.75	146.25	0.52	2.17	51.73	2.49	0.79	0.18	3.94
KIKIKIKIWWWW	3.94	1.02	-0.10	11.28	130.00	0.23	-0.57	-40.36	2.03	0.57	0.14	2.92
KIKIKIKI	3.94	0.41	0.30	11.28	195.00	0.18	0.32	-65.54	3.04	0.85	0.20	4.39
CEFEFQFNMWQ	-2.13	0.73	-0.51	3.61	0.00	0.16	1.49	2.24	1.89	0.63	0.18	3.90
CSISIQI	-0.13	0.95	1.56	5.92	167.14	0.19	-0.53	8.57	2.65	0.77	0.14	3.08
CEIEIQINMWQ	-2.13	0.74	-0.05	3.61	106.36	0.17	0.97	84.05	2.23	0.72	0.15	3.39
CEIEIQINM	-2.13	0.67	0.43	3.61	130.00	0.11	0.82	100.51	2.55	0.81	0.17	3.70
CSISIQINM	-0.13	0.81	1.03	5.92	130.00	0.09	0.07	8.89	2.50	0.77	0.15	3.22
KAKAQANM	1.94	-0.08	-0.94	10.81	37.50	0.11	1.94	8.75	1.64	0.64	0.14	2.65
CKAKAQA	1.87	0.04	-0.49	9.68	42.86	0.15	1.42	8.57	1.56	0.60	0.13	2.32
HHHHKIKIQINMYYYY	2.38	0.51	-1.09	9.61	73.13	0.20	1.59	46.19	2.85	0.77	0.23	4.84
KIKIKIKIWW	3.94	0.77	0.06	11.28	156.00	0.23	-0.21	-50.43	2.43	0.68	0.16	3.51
HHHHKIKIKIKIWWWW	4.39	0.80	-0.88	11.28	97.50	0.24	0.74	-12.97	2.27	0.60	0.16	3.36
MKIKIQINM	1.94	0.56	0.28	10.81	130.00	0.11	0.42	-19.41	2.69	0.82	0.19	4.14
RGDCKIKIQINM	1.87	0.30	-0.44	9.67	97.50	0.33	2.30	-19.13	2.30	0.73	0.17	3.68
MKIKIQINMWQ	1.94	0.64	-0.17	10.81	106.36	0.16	0.64	-14.06	2.34	0.74	0.18	3.75
HHHHKIKIKIKIYYYY	4.38	0.48	-0.98	10.20	97.50	0.22	1.36	10.17	3.00	0.78	0.23	4.98
RGDCKFKFQF	1.87	0.29	-0.88	9.67	0.00	0.32	2.91	-22.06	1.99	0.62	0.20	4.25
KIKIQIW	1.94	0.78	0.19	10.81	167.14	0.25	-0.06	-27.81	2.56	0.73	0.17	3.64
KFKFQFW	1.94	0.77	-0.54	10.81	0.00	0.24	0.77	-23.67	2.02	0.59	0.21	4.45
CKFKFQFW	1.87	0.87	-0.16	9.68	0.00	0.22	0.51	-19.46	1.99	0.60	0.20	4.20
EIKIQINM	-0.06	0.52	0.13	6.41	146.25	0.19	0.93	11.60	2.69	0.83	0.18	3.99
IKVAVKIKIQINM	2.94	0.57	0.88	11.10	172.31	0.36	-0.24	-25.64	2.81	0.80	0.17	3.26
HHHHKAKAKAKAWWWW	4.39	0.43	-1.55	11.28	25.00	0.22	1.52	24.18	1.54	0.48	0.12	2.38
CKIW	0.87	1.15	0.55	8.55	97.50	0.67	-0.75	-13.73	1.96	0.58	0.13	2.80
CKIKIQINMW	1.87	0.76	0.22	9.68	117.00	0.20	0.26	-16.47	2.36	0.73	0.17	3.53
ILKNLSRSRKIKIQINMWQ	4.94	0.39	-0.51	12.55	123.16	0.52	2.29	45.14	2.29	0.75	0.18	3.79
EIEIQINM	-2.06	0.57	0.18	3.61	146.25	0.16	1.09	111.83	2.65	0.83	0.17	3.86
KIKIQINMWWQ	1.94	0.74	-0.43	10.81	106.36	0.30	0.64	-14.06	2.13	0.67	0.16	3.35
KIKIQINMWWWQ	1.94	0.86	-0.47	10.81	97.50	0.16	0.39	-12.06	1.95	0.61	0.14	3.07
KFKQFFINMWQ	1.94	0.69	-0.40	10.81	35.45	0.49	0.93	10.10	2.17	0.67	0.20	4.23
KIKIQIMWNQ	1.94	0.59	-0.38	10.81	117.00	0.06	0.94	-11.67	2.34	0.73	0.17	3.68
KIKIQIMQWN	1.94	0.59	-0.38	10.81	117.00	0.22	0.94	-11.67	2.34	0.73	0.17	3.68
KIKIQINMWQRGD	1.94	0.31	-0.94	10.79	90.00	0.49	2.47	-16.89	2.10	0.67	0.16	3.52
EIEIQINMWQ	-2.06	0.66	-0.30	3.61	117.00	0.21	1.19	91.46	2.28	0.73	0.16	3.48
KIKIKIQINMWQ	2.94	0.56	-0.27	11.10	130.00	0.09	0.83	-26.21	2.46	0.75	0.18	3.80

Experimental descriptors

Table S5 Summary of experimental descriptors measured for the peptide library: Absolute Infectivity at 1.3 µM peptide concentration (Abs. Inf.) and the corresponding standard deviation from triplicate measurements (Std. Dev.), the infectivity relative to EF-C (Inf. rel. EF-C) and the logarithmic infectivity relative to EF-C (Log_{10} Inf. rel EF-C), percentual β–sheet content from FT-IR measurements (**Figure S34**), ThT-fluorescence relative to control (Rel. ThT) and categorical ThT fluorescence (ThT. Cat is 1, if Rel. ThT >2), zeta potential (Zeta-pot.) and the derived count rate of scattered light from zeta-potential measurements (Count Rate), Conversion of monomers to assembled structure (Conversion Rate CR) and fibril formation (1 = fibrils present, 0 non-fibrillar structures) as evaluated qualitatively from TEM measurements (**Figure S33**).

Sequence	Abs. Inf. / RLU s^{-1}	Std. Dev. / RLU s^{-1}	Inf. rel. EF-C	Std. Dev.	Log_{10} Inf. rel. EF-C	ß-sheet / %	Rel. ThT	ThT Cat.	Zeta pot. / mV	Std. Dev. / mV	Count Rate / kcps	Std. Dev. / kcps	Log_{10} Count Rate	CR/ %	Std. Dev. CR / %	Fibril
CKIKIQIII	22150	2532	0.98	0.20	-0.01	37	4.1	1	16.2	3.2	4953	4191	3.7			1
CEIEIQI	633	578	0.03	0.03	-1.55	45	2.2	1	-26.1	7.1	52	25	1.7	59	3	1
CKIKQIINM	11280	3038	0.50	0.16	-0.30	46	14.7	1	12.9	4.8	9058	2261	4.0	45	2	1
CKFKFQFNMWQ	26320	2463	1.16	0.23	0.06	47	2.4	1	17.0	0.3	143	56	2.2	98	2	1
CKFKFQFNM	28183	3701	1.24	0.27	0.09	41	2.4	1	15.1	0.9	2224	2193	3.3	94	6	1
CKFKFQF	20067	2227	1.23	0.25	0.09	47	2.3	1	19.7	0.2	5303	4282	3.7	96	1	1
KFKFQFNMW	6690	1272	0.45	0.12	-0.35	38	1.3	0	22.6	2.8	610	162	2.8			1
KFKFQFNM	7067	1516	0.47	0.13	-0.33	40	1.2	0	13.9	1.5	132	141	2.1	95	4	1
KFKFQFN	270	135	0.02	0.01	-1.74	38	1.1	0	12.7	10.0	51	9	1.7			1
CKAKAQANMWQ	600	134	0.04	0.01	-1.36	39	1.9	0	0.7	2.3	9	7	1.0	92	0	0
CKAKAQANM	430	114	0.03	0.01	-1.50	34	1.2	0	-7.8	1.4	207	146	2.3	48	10	0
QCKFKQFFNMWQ	30897	2664	0.25	0.07	-0.61	42	25.4	1	9.6	0.4	18911	16193	4.3	99	0	1
QCKFKQFFNM	5847	75	0.43	0.10	-0.37	42	12.7	1	13.7	0.4	10385	1136	4.0	99	0	1
QCKFKQFF	170	1010	0.01	0.01	-1.91	32	2.3	1	4.9	5.2	225	280	2.4			1
CKFKQFFNMWQ	23487	142	0.61	0.16	-0.21	59	27.7	1	8.6	4.1	8210	2689	3.9	99	1	1
CKFKQFFNM	15720	4394	0.41	0.16	-0.39	42	46.0	1	15.2	3.0	27264	5488	4.4			1
CKFKQFF	260	5573	0.01	0.00	-2.17	45	1.3	0	11.6	1.4	3	1	0.4	90	3	0
QCKIKIQINM	15250	156	0.40	0.16	-0.40	37	2.5	1	11.4	0.3	9524	5626	4.0	90	7	1
QCKAKAQANMWQ	2767	5347	0.07	0.02	-1.14	33	1.5	0	-8.1	0.4	428	487	2.6	89	7	0
QCKAKAQANM	563	671	0.01	0.01	-1.83	35	1.4	0	-8.4	2.6	204	224	2.3	56	35	0
QCKAKAQA	807	176	0.03	0.01	-1.60	34	1.3	0	0.7	5.2	1319	1729	3.1			0
QCKIKIQI	3577	272	0.11	0.04	-0.95	35	2.0	1	9.5	3.2	12427	14002	4.1			1
QCKIKQIINM	11187	1058	0.35	0.12	-0.46	38	23.3	1	10.4	3.2	20551	16126	4.3			1
QCKIKQII	207	3309	0.01	0.00	-2.19	41	3.0	1	11.0	2.5	301	199	2.5			1
QCKFKFQFNMWQ	19753	151	0.62	0.12	-0.21	47	1.9	0	21.1	0.8	1208	302	3.1	99	0	1
QCKFKFQFNM	16353	1583	0.51	0.10	-0.29	37	2.3	1	15.7	0.7	3670	2822	3.6	99	2	1
QCKFKFQF	20303	1679	0.77	0.16	-0.12	40	2.0	1	12.2	3.7	2669	932	3.4			1
CRFRFQF	7508	2292	0.34	0.20	-0.46	40	3.5	1	-2.1	3.9	391	338	2.6	98	0	1
HIHIQIC	4621	1135	0.27	0.16	-0.57	48	12.5	1	-3.7	0.5	261	128	2.4	83	5	1
RLRLTLC	1334	826	0.05	0.03	-1.29	21	1.9	0	19.7	0.9	126	117	2.1	87	5	1
HLHLPLL	133	167	0.01	0.01	-2.00	49	1.1	0	-9.1	0.5	200	207	2.3	38	10	0

RGECKFKFQF	7411	193	0.33	0.18	-0.48	46	2.4	1	24.0	0.3	219	213	2.3	99	0	1
RGEKIKIQINM	7838	1054	0.24	0.11	-0.61	38	3.4	1	15.3	3.0	595	686	2.8	80	7	1
KYKGAIIGNIK	80	1260	0.00	0.00	-2.51	45	1.4	0	-9.1	3.6	44	21	1.6			0
HGDKCHGDKC	263	121	0.01	0.01	-1.99	5	1.2	0	-11.6	1.2	12	10	1.1	37	13	0
RPRGLLLGNLR	717	229	0.05	0.02	-1.32	14	1.3	0	-8.2	6.5	210	156	2.3			0
KKFQKKFQ	423	220	0.03	0.02	-1.54	40	1.2	0	-9.2	4.0	74	37	1.9			0
PPFHPPPFHP	263	234	0.02	0.01	-1.75	47	1.4	0	-15.8	9.7	59	26	1.8			0
MDQMDQMDQMDQMDQ	63	143	0.00	0.01	-2.37	46	1.1	0	-15.0	4.0	10	4	1.0	53	3	0
FDPFDPFDP	347	75	0.02	0.00	-1.63	49	1.3	0	-10.9	3.6	258	235	2.4			0
TKTLTKTL	317	31	0.02	0.01	-1.67	34	1.3	0	-25.1	4.1	515	356	2.7			0
FKFDKFKFDK	513	55	0.05	0.02	-1.33	47	1.5	0	-12.7	10.3	45	22	1.7			0
KVKGVGK	287	213	0.03	0.01	-1.59	9	1.8	0	-12.8	4.2	51	30	1.7			0
SISISRRI	313	133	0.03	0.03	-1.55	49	1.0	0	-17.0	0.7	25	14	1.4	24	7	0
HRRHFRHKITKKK	150	289	0.01	0.01	-1.87	45	1.1	0	-9.2	2.7	10	4	1.0	45	2	0
KNERIKNERI	143	126	0.01	0.00	-1.89	38	1.3	0	-16.0	3.7	322	166	2.5	54	8	0
KIRGKFEKED	643	20	0.06	0.03	-1.24	29	3.4	1	-12.7	6.7	214	151	2.3			0
CKFQC	497	364	0.02	0.00	-1.64	44	1.5	0	-17.4	6.5	130	106	2.1	33	2	0
MKFM	383	57	0.02	0.00	-1.76	35	1.1	0	-16.0	5.2	131	159	2.1			0
CKFC	240	46	0.01	0.01	-1.96	39	1.4	0	-19.9	10.0	235	136	2.4	36	11	0
RGDKIRGDKI	163	156	0.01	0.00	-2.13	15	1.1	0	-13.6	0.4	286	367	2.5	48	10	0
KNDKND	230	85	0.01	0.01	-1.98	43	1.3	0	-15.0	25.8	52	29	1.7			0
HGEHGE	140	116	0.01	0.00	-2.19	10	1.6	0	-12.6	3.5	241	285	2.4			0
HGEHGEHGE	360	35	0.02	0.01	-1.72	6	1.2	0	-24.2	7.2	340	318	2.5	43	7	0
CRFRVPF	377	117	0.02	0.01	-1.70	11	0.9	0	4.4	6.0	74	90	1.9	97	1	0
CHLHLQL	2934	106	0.10	0.05	-1.01	50	3.2	1	-12.3	1.5	1155	381	3.1	97	2	0
CETMYDKILKNLSRSR	363	670	0.02	0.01	-1.72	62	1.1	0	0.4	0.3	1099	1415	3.0	99	0	0
MKFKFQF	1427	127	0.06	0.01	-1.20	22	1.0	0	-3.1	0.9	124	68	2.1	87	1	1
QCKIKQIINMWQ	21312	70	1.00	0.25	0.00	52	3.8	1	6.6	0.4	2606	936	3.4	77	7	1
QCKIKIQINMWQ	21221	3959	1.04	0.27	0.02	36	9.1	1	11.7	0.4	8271	7432	3.9	94	4	1
KIKIQINMWQ	6650	4304	0.30	0.07	-0.53	42	5.9	1	14.9	0.9	2196	1431	3.3	94	1	1
NMWQKAKAQA	1137	1144	0.04	0.01	-1.38	37	5.2	1	-5.6	8.9	242	186	2.4			1
NMWQKFKFQF	7323	244	0.27	0.23	-0.57	33	1.0	0	9.6	3.0	14045	10746	4.1			1
KVKVKVQV	467	6103	0.02	0.01	-1.76	43	5.8	1	-3.4	6.8	94	74	2.0			0
KIKQIINMWQ	9363	178	0.35	0.16	-0.46	41	4.9	1	15.4	0.8	616	488	2.8	90	2	1
KAKAQANMWQ	193	3859	0.01	0.00	-2.15	43	1.0	0	-19.6	1.7	56	45	1.7	56	4	0
KFKFQFNMWQ	20677	104	0.76	0.24	-0.12	43	2.8	1	25.4	0.8	735	170	2.9	96	0	1
KFKQFFNMWQ	1000	5282	0.09	0.02	-1.05	33	1.6	0	-3.4	5.1	237	94	2.4			0
KAKQAANMWQ	343	79	0.03	0.02	-1.51	11	1.2	0	-12.9	5.2	690	585	2.8			0
NMWQKVGTPL	277	162	0.02	0.01	-1.61	32	2.2	1	-9.6	5.7	27	8	1.4			0
NMWQKIKQII	283	76	0.03	0.02	-1.60	38	1.3	0	-0.2	3.8	982	389	3.0			0
NMWQKIKIQI	2300	180	0.21	0.04	-0.69	39	5.0	1	8.3	3.4	10024	8218	4.0			1
KIKQIINM	5053	145	0.45	0.12	-0.35	44	1.2	0	16.3	4.1	2481	1952	3.4	43	9	1
KIKQIIN	323	1079	0.04	0.01	-1.40	38	1.1	0	-11.7	9.8	64	61	1.8			0
KIKIQINMW	17523	90	2.16	0.44	0.33	46	4.6	1	19.7	4.2	240	92	2.4			1

KIKIQINM	2947	1917	0.36	0.08	-0.44	41	1.6	0	8.5	6.4	262	143	2.4	64	3	1
KIKIQIN	2153	339	0.27	0.09	-0.58	42	1.8	0	3.3	12.9	43	26	1.6			1
KAKAQA	147	595	0.02	0.01	-1.74	40	1.2	0	-14.1	3.1	21	12	1.3	0	20	0
KIKIQI	550	111	0.07	0.01	-1.17	39	1.1	0	-5.6	1.6	108	80	2.0	19	11	0
KFKFQF	687	60	0.06	0.01	-1.21	46	1.2	0	-11.0	1.3	183	152	2.3	67	12	0
KIKIQINMWA	7677	60	0.69	0.15	-0.16	50	1.5	0	14.7	7.3	1101	467	3.0			1
EIEIEIEI	200	961	0.02	0.01	-1.75	63	2.0	0	-8.9	4.9	538	681	2.7			1
KIKIQINMAQ	2383	152	0.21	0.06	-0.67	46	1.6	0	3.1	92.6	5859	8262	3.8			1
KIKIQINAWQ	6013	567	0.54	0.12	-0.27	28	2.5	1	13.1	3.0	1494	1899	3.2			1
KIKIQIAMWQ	4350	783	0.39	0.07	-0.41	37	2.4	1	11.3	4.0	541	620	2.7			1
KIKIKIKIYYYY	30950	289	1.38	0.26	0.14	57	1.3	0	13.3	5.1	9918	3446	4.0			0
HHHHEIEIEIEI	655	1980	0.03	0.01	-1.53	48	10.6	1	-6.9	62.3	103	53	2.0			0
KYKYQY	230	219	0.01	0.01	-1.99	0	6.1	1	-4.2	5.4	32	19	1.5			0
EFEFQF	73	246	0.00	0.00	-2.49	45	2.8	1	-24.5	4.3	405	111	2.6			0
CEFEFQF	117	91	0.01	0.00	-2.28	53	1.4	0	-13.7	13.1	367	494	2.6			1
CKFKFQFNMW	21517	80	0.96	0.21	-0.02	48	6.3	1	18.4	3.2	4107	2513	3.6			1
CKYKYQY	1083	2692	0.04	0.02	-1.39	49	3.1	1	1.5	3.6	1061	439	3.0			0
KWKWQW	387	383	0.01	0.01	-1.84	30	1.5	0	7.6	7.3	1545	1957	3.2			0
CKWKWQW	6760	136	0.25	0.05	-0.60	34	1.3	0	14.7	3.1	7511	924	3.9			1
KLKQLL	197	575	0.01	0.00	-2.13	32	1.1	0	-8.5	3.3	320	216	2.5			0
CKIKIQINMWQ	18623	25	0.70	0.14	-0.16	45	4.6	1	13.9	0.4	1881	680	3.3	89	2	1
RGDKIKIQI	417	1812	0.02	0.02	-1.81	40	5.6	1	-0.9	14.5	298	401	2.5			1
CKIKIQINM	30273	394	1.59	0.30	0.20	43	2.6	1	16.8	0.5	4228	485	3.6	75	3	1
CKIKIQI	19407	1919	1.02	0.23	0.01	53	3.9	1	12.8	3.5	2433	1343	3.4	54	1	1
CKIKQII	797	2633	0.04	0.02	-1.38	25	3.9	1	-3.8	6.4	75	27	1.9	55	1	1
CKIKQIINMWQ	23697	276	1.24	0.31	0.09	51	48.5	1	13.4	3.4	8317	3111	3.9	50	2	1
KIKIQIRGD	400	4304	0.02	0.01	-1.68	47	40.5	1	6.4	4.6	114	32	2.1			1
RGDKIKIQIC	19382	163	0.83	0.17	-0.08	41	19.8	1	16.5	3.8	7509	8351	3.9	80	4	1
RGDKIKIQINM	9554	2669	0.41	0.10	-0.38	46	9.1	1	16.4	1.9	57	32	1.8	61	13	1
RGDKIKIQINMWQ	12503	1540	0.76	0.15	-0.12	49	1.8	0	6.6	7.7	1554	1432	3.2	66	14	1
KFKFEFEF	577	1244	0.03	0.02	-1.55	41	2.7	1	-2.6	3.6	2186	1813	3.3			1
KIKQII	100	319	0.00	0.00	-2.31	39	1.0	0	-12.7	6.4	122	146	2.1	13	7	0
KIEIQINM	707	82	0.03	0.01	-1.46	43	1.0	0	-0.4	-1.0	275	215	2.4			1
CKIKIQIRGD	6660	181	0.33	0.16	-0.48	58	0.0	0	7.2	0.6	5777	938	3.8	91	1	1
CKIKQIIRGD	993	3045	0.05	0.01	-1.31	nd	1.4	0	13.2	3.4	3495	2551	3.5			0
RGDKIKIQINMC	21717	198	1.07	0.26	0.03	nd	1.4	0	12.2	3.7	1404	774	3.1	60	8	1
CQFQFQF	7570	3707	0.35	0.08	-0.45	35	13.0	1	-24.8	3.8	7929	4815	3.9			1
KIKIQII	400	955	0.02	0.01	-1.73	45	1.4	0	5.6	9.3	1039	219	3.0			1
KFKFQFFF	8657	93	0.40	0.18	-0.39	49	2.6	1	13.3	0.7	3039	608	3.5	98	1	1
KIKIKIQI	2073	3591	0.10	0.03	-1.01	40	2.0	1	7.0	6.6	999	801	3.0	80	1	1
KAKAKAQA	187	563	0.01	0.00	-2.06	40	1.2	0	-9.0	5.9	77	65	1.9			0
KLKLKLQL	2097	55	0.10	0.04	-1.01	42	1.6	0	8.4	8.8	43	2	1.6			1
KFKFKFQF	18697	862	0.80	0.16	-0.10	40	41.9	1	15.2	3.3	1036	442	3.0	94	5	1
KVKVQVV	213	2009	0.01	0.00	-2.04	45	1.3	0	-7.9	5.6	28	21	1.4			0

KLKLQLL	163	53	0.01	0.01	-2.16	40	0.8	0	-4.9	4.4	73	62	1.9			1
KFKFQFF	2850	135	0.12	0.04	-0.91	48	2.6	1	6.9	1.7	38	13	1.6			1
KIKIQIII	8260	702	0.35	0.08	-0.45	46	2.3	1	19.8	9.3	95	40	2.0	87	2	1
KVKVQVVV	820	1160	0.04	0.01	-1.46	46	0.9	0	0.1	10.5	163	192	2.2			1
HHHHKAKAKAKAYYYY	203	78	0.01	0.00	-1.88	44	1.4	0	7.0	1.4	241	83	2.4			0
KIKIQIC	9447	17	0.61	0.11	-0.22	53	4.1	1	19.9	1.6	981	305	3.0	52	11	1
RGDSKIKIQIC	10816	150	0.60	0.19	-0.22	39	13.8	1	15.1	3.9	2699	210	3.4	43	11	1
RGDSGGGGGGKIKIQIC	13453	3541	0.87	0.18	-0.06	41	42.0	1	16.7	0.7	10416	5402	4.0	55	25	1
ILKNLSRSRKIKIQIC	12740	1659	0.34	0.08	-0.47	38	2.7	1	18.0	4.2	2392	1866	3.4			1
KIKIKIKIWWWW	22347	1999	0.59	0.11	-0.23	38	2.3	1	15.2	3.0	12421	2307	4.1			0
KIKIKIKI	1237	1418	0.03	0.01	-1.49	40	3.3	1	5.5	13.4	155	38	2.2			0
CEFEFQFNMWQ	2517	411	0.14	0.08	-0.85	51	6.1	1	-34.2	0.4	186	44	2.3	99	0	1
CSISIQI	3087	326	0.06	0.01	-1.25	59	15.0	1	-25.9	1.8	10241	5508	4.0	79	3	1
CEIEIQINMWQ	12067	422	0.22	0.05	-0.66	50	3.7	1	-25.8	1.3	1570	1451	3.2	67	7	1
CEIEIQINM	3593	1935	0.07	0.01	-1.19	46	5.1	1	-15.3	8.4	58	75	1.8	43	9	1
CSISIQINM	10943	497	0.20	0.06	-0.70	56	5.9	1	-19.4	3.2	3291	1256	3.5	86	3	1
KAKAQANM	497	2645	0.01	0.01	-2.05	33	1.1	0	-2.6	4.3	43	25	1.6			0
CKAKAQA	1740	335	0.03	0.01	-1.50	35	1.2	0	-8.0	5.8	363	387	2.6	44	9	0
HHHHKIKIQINMYYYY	22409	406	1.11	0.62	0.04	47	12.2	1	7.8	0.6	5659	3747	3.8	98	0	1
KIKIKIKIWW	20557	1379	0.37	0.09	-0.43	49	3.5	1	15.4	3.1	9436	981	4.0	92	3	0
HHHHKIKIKIKIWWWW	20797	3303	0.38	0.07	-0.42	49	4.0	1	14.5	3.4	23757	9196	4.4	96	1	0
MKIKIQINM	15000	1065	0.27	0.05	-0.56	39	1.3	0	9.1	2.9	981	707	3.0			1
RGDCKIKIQINM	7203	1002	0.24	0.05	-0.63	43	1.8	0	11.3	5.5	1623	1122	3.2			1
MKIKIQINMWQ	12787	869	0.42	0.10	-0.38	39	4.1	1	10.8	2.9	1475	1141	3.2			1
HHHHKIKIKIKIYYYY	20713	2171	0.68	0.20	-0.17	53	9.9	1	13.2	3.8	12793	5972	4.1			0
RGDCKFKFQF	4637	4934	0.15	0.05	-0.82	47	6.8	1	13.9	1.5	1338	300	3.1	85	9	1
KIKIQIW	4280	1272	0.14	0.03	-0.85	49	2.0	1	12.5	4.2	145	168	2.2			1
KFKFQFW	4953	453	0.16	0.03	-0.79	35	3.5	1	6.5	2.8	122	56	2.1			1
CKFKFQFW	17862	357	0.88	0.18	-0.06	43	1.8	0	15.2	1.1	29211	17451	4.5	99	0	1
EIKIQINM	3607	2252	0.10	0.04	-1.00	48	3.8	1	-22.3	2.1	2816	2882	3.4	51	7	1
IKVAVKIKIQINM	17920	1317	0.50	0.09	-0.30	58	1.8	0	14.5	3.5	4848	1475	3.7			1
HHHHKAKAKAKAWWWW	3233	592	0.09	0.02	-1.05	40	3.7	1	17.4	8.6	659	233	2.8			0
CKIW	290	434	0.05	0.03	-1.29	46	1.0	0	-3.7	0.6	3	2	0.5	48	15	0
CKIKIQINMW	7253	159	1.29	0.24	0.11	43	1.4	0	18.3	3.7	4915	5114	3.7			1
ILKNLSRSRKIKIQINMWQ	1797	478	0.32	0.07	-0.49	39	3.3	1	19.2	3.8	307	189	2.5			1
EIEIQINM	203	221	0.04	0.01	-1.44	54	1.5	0	-23.3	1.1	134	85	2.1	78	4	1
KIKIQINMWWQ	17977	44	3.21	0.56	0.51	50	1.3	0	11.8	4.0	3893	1970	3.6			0
KIKIQINMWWWQ	15400	408	2.75	0.51	0.44	48	1.3	0	9.5	2.6	7596	2548	3.9			0
KFKQFFINMWQ	14953	937	0.92	0.26	-0.04	38	1.1	0	8.0	3.2	8996	4347	4.0			1
KIKIQIMWNQ	9147	3226	0.56	0.10	-0.25	41	1.2	0	3.0	4.0	1887	306	3.3			1
KIKIQIMQWN	11623	168	0.72	0.18	-0.15	40	1.3	0	9.4	10.5	2203	415	3.3			1
KIKIQINMWQRGD	197	2183	0.01	0.01	-1.92	48	1.5	0	1.7	6.4	2783	3327	3.4			0
EIEIQINMWQ	2137	85	0.13	0.03	-0.88	54	4.1	1	-19.1	1.3	1264	133	3.1	90	1	0
KIKIKIQINMWQ	20353	176	0.79	0.15	-0.10	40	1.6	0	13.1	2.9	1603	498	3.2		3	1

Table S6 Summary of experimental descriptors measured for the peptides designed via pattern analysis: Absolute Infectivity at 1.3 µM peptide concentration (Abs. Inf.) and the corresponding standard deviation from triplicate measurements (Std. Dev.), the infectivity relative to EF-C (Inf. rel. EF-C) and the logarithmic infectivity relative to EF-C (Log_{10} Inf. rel EF-C), percentual β–sheet content from FT-IR measurements (**Figure S38**), ThT-fluorescence relative to control (Rel. ThT) and categorical ThT fluorescence (ThT. Cat is 1, if Rel. ThT >2), zeta potential (Zeta-pot.) and the derived count rate of scattered light from zeta-potential measurements (Count Rate), and fibril formation (1 = fibrils present, 0 non-fibrillar structures) as evaluated qualitatively from TEM measurements (**Figure S25**).

**Sequence**	**Abs. Inf. / RLU s^{-1}**	**Std. Dev. / RLU s^{-1}**	**Inf. rel. EF-C**	**Std. Dev.**	**Log_{10} Inf. rel. EF-C**	**ß-sheet / %**	**Rel. ThT**	**ThT Cat.**	**Zeta pot. / mV**	**Std. Dev. / mV**	**Count Rate / kcps**	**Std. Dev. / kcps**	**Log_{10} Count Rate**	**Fibril**
CKIWIQIY	115280	27300	0.57	0.14	-0.24	31	6	1	4.5	0.1	1842	818	3.3	1
KIWIWIW	105670	2895	0.53	0.05	-0.28	33	9	1	11.3	0.3	6187	4991	3.8	1
KFQFNMW	36267	3869	0.18	0.02	-0.74	28	1	0	8.8	0.7	332	261	2.5	1
KIKIWIW	65420	6552	0.33	0.04	-0.49	40	1	0	17.2	0.4	608	46	2.8	1
CKIKIWIW	173537	22193	0.86	0.13	-0.06	39	2	1	19.1	0.3	3321	309	3.5	1
RVHLGLG	4140	1152	0.02	0.01	-1.69	30	1	0	-6.0	1.0	28	34	1.5	1
CRLSLSLS	63003	10341	0.31	0.06	-0.50	38	2	0	9.0	0.6	1697	1030	3.2	1
CRLRVGVS	8737	3852	0.04	0.02	-1.36	0	1	0	10.5	0.5	35	16	1.5	0
RVRVTVT	11983	2526	0.06	0.01	-1.22	40	1	0	-3.7	1.5	25	12	1.4	0
RINFWFW	9033	4840	0.04	0.02	-1.35	52	16	1	10.8	0.2	50996	8911	4.7	0
RFWFWFW	64303	19492	0.32	0.10	-0.49	31	7	1	13.6	0.1	30037	37815	4.5	0
RIRFHIW	2087	1777	0.01	0.01	-1.98	37	1	0	0.1	0.2	12	4	1.1	0
CRFRFHFH	106307	27039	0.53	0.14	-0.28	38	1	0	19.6	0.5	430	217	2.6	1
KAQCTLP	14133	5124	0.07	0.03	-1.15	0	1	0	-8.5	1.8	97	119	2.0	0
KCQCQCQ	230517	3686	1.15	0.10	0.06	33	15	0	11.8	0.3	15236	3247	4.2	1
KAKLTCT	13367	5300	0.07	0.03	-1.18	41	1	0	-2.3	1.6	17	15	1.2	0
KLKLTLT	7733	6934	0.04	0.03	-1.41	40	1	1	1.7	0.1	199	83	2.3	1
CKFKFPF	4987	4704	0.02	0.02	-1.61	0	1	0	-1.1	1.4	143	147	2.2	0

Table S7 Summary of experimental descriptors measured for the peptides found from data base screening: Absolute Infectivity at 1.3 µM peptide concentration (Abs. Inf.) and the corresponding standard deviation from triplicate measurements (Std. Dev.), the infectivity relative to EF-C (Inf. rel. EF-C) and the logarithmic infectivity relative to EF-C (Log_{10} Inf. rel EF-C), percentual β–sheet content from FT-IR measurements (**Figure S39**), ThT-fluorescence relative to control (Rel. ThT) and categorical ThT fluorescence (ThT. Cat is 1, if Rel. ThT >2), zeta potential (Zeta-pot.) and the derived count rate of scattered light from zeta-potential measurements (Count Rate), and fibril formation (1 = fibrils present, 0 non-fibrillar structures) as evaluated qualitatively from TEM measurements (**Figure S36**).

Sequence	Abs. Inf. / RLU s^{-1}	Std. Dev. / RLU s^{-1}	Inf. rel. EF-C	Std. Dev.	Log_{10} Inf. rel. EF-C	ß-sheet / %	Rel. ThT	ThT Cat.	Zeta pot. / mV	Std. Dev. / mV	Count Rate / kcps	Std. Dev. / kcps	Log_{10} Count Rate	Fibril
IKFLSVN	8601	3301	0.07	0.02	-1.16	48	1	0	5.9	0.7	137	43	2.1	1
VQIVYK	7994	1434	0.07	0.01	-1.17	45	2	0	-2.7	1.0	26	22	1.4	0
IIKIIK	9742	3518	0.08	0.03	-1.10	52	1	0	-7.0	1.0	4	2	0.6	0
GQTVTK	6998	2415	0.06	0.02	-1.25	29	1	0	-5.3	1.0	34	8	1.5	0
DFNKF	9988	2476	0.08	0.02	-1.07	53	1	0	-11.8	0.4	1065	1483	3.0	1
RCELARTLKR	13881	4241	0.09	0.03	-1.06	16	4	1	-0.9	0.6	78	40	1.9	0
ISKLEYSNFSVRY	92993	11998	0.54	0.09	-0.26	43	1	0	9.3	0.2	7807	2555	3.9	1
GYVIIK	13085	3603	0.10	0.02	-0.99	42	1	0	9.9	1.3	30	4	1.5	0
KLVFFAK	13708	3677	0.07	0.02	-1.15	42	1	0	3.2	0.3	14	9	1.2	1
RATVYV	7568	4530	0.04	0.03	-1.38	46	1	0	-5.2	1.6	42	41	1.6	1
RQGNINIVA	21525	7173	0.12	0.04	-0.93	50	5	1	10.2	0.8	36	29	1.6	1
KYFIL	11684	2396	0.07	0.02	-1.14	21	203	1	-7.2	0.5	18903	20938	4.3	0
NKGAII	10918	3461	0.06	0.02	-1.20	31	1	0	-9.5	2.5	68	82	1.8	0
IIIQGK	11434	2250	0.06	0.01	-1.21	44	1	0	-7.6	0.1	10	2	1.0	0
LASLIYR	12491	2722	0.06	0.02	-1.20	49	1	0	-5.0	2.6	105	123	2.0	0
IIIK	9273	3071	0.05	0.02	-1.29	48	1	0	-3.8	1.9	26	7	1.4	0
MKVIFLKDVKG	20404	3725	0.12	0.02	-0.93	53	1	0	9.6	0.8	137	109	2.1	1
LANWMCLAKW	76339	8813	0.40	0.06	-0.40	36	11	1	14.7	0.3	15062	4245	4.2	0
VHDCVNITIK	103498	13829	0.53	0.08	-0.27	51	428	1	9.3	0.3	12542	10750	4.1	1
IFQINS	12629	2531	0.06	0.01	-1.21	39	7	1	-11.0	3.8	36	20	1.6	1
NSGAITIG-NH_2	11611	2899	0.05	0.02	-1.26	32	4	1	-8.4	0.9	20	18	1.3	0
GAITIG-NH_2	8902	2091	0.04	0.01	-1.35	39	1	0	-5.4	0.9	30	31	1.5	0
VLYVGSKT	12423	2982	0.06	0.02	-1.24	48	1	0	-9.1	3.3	24	19	1.4	0
GNNQQNY	11825	4465	0.05	0.02	-1.28	31	2	1	-6.6	1.1	37	31	1.6	0

Table S8 Summary of aggregate analysis from widefield microscopy measurements (**Figure S32**). The Number of aggregated peptides, the median size area, average size area and the cumulative total size area of all particles (> 10 µm²) in an area of 1330 µm × 1330 µm was determined via automated 3D object counting (ImageJ) in triplicate measurements (Std. Dev.).

Sequence	Abs. Inf. / RLU s^{-1}	Std. Dev. / RLU s^{-1}	Count Rate / kcps	Std. Dev. / kcps	Aggregate Number	Std. Dev. Aggregate Number	Median Area / µm²	Std. Dev. Median Area / µm²	Average Area / µm²	Std. Dev. Average Area / µm²	Total Area/ µm²	Std. Dev. Total Area/ µm²
CKFKQFFNM	15720	4394	27264	5488	704	90	15	1	31	44	21881	2095
CKFKFQFW	17862	357	29211	17451	517	80	17	1	28	35	14537	2313
QCKIKQIINMWQ	21312	70	2602	937	408	35	28	3	72	112	29314	1897
HHHHKIKIKIKIWWWW	20797	3303	23757	9196	446	122	25	4	50	70	22294	7605
QCKIKIQINMWQ	21221	3959	8271	7432	398	139	12	1	21	28	8454	4577
HHHHKIKIQINMYYYY	22409	406	5659	3747	234	44	28	2	97	217	22687	8518
CKIKIQI	19407	1919	2433	1343	317	36	16	2	59	196	18615	2924
CKFKFQFNM	28183	3701	2224	2193	170	33	12	0	20	27	3388	573
CKIKQIINMWQ	23697	276	8317	3111	195	28	92	33	183	263	35687	4610
HHHHKIKIKIKIYYYY	20713	2171	12793	5972	147	39	20	3	72	155	10553	5882
CKFKFQF	20067	2227	5303	4282	128	22	22	5	87	199	11107	3665
CSISIQI	3087	326	10241	5508	123	6	19	3	112	366	13871	3490
RGDSGGGGGGKIKIQIC	13453	3541	10416	5402	72	5	53	44	373	836	26844	7348
CKWKWQW	6760	136	7511	924	87	10	29	18	96	211	8346	4684
KIKIQIC	9447	17	981	305	82	6	13	1	23	31	1915	358
CQFQFQF	7570	3707	7929	4815	46	23	15	5	73	186	3337	308
KFKFQFNMWQ	20677	104	735	170	64	9	26	5	599	1583	38349	5028
CKIKQII	797	2633	75	27	76	12	36	5	111	231	8372	789
KIKQIINMWQ	9363	178	616	488	39	5	199	92	398	443	15530	2319
HIHIQIC	4621	1135	261	128	33	7	86	68	748	1646	24920	8795
CHLHLQL	2934	106	1155	381	36	10	36	18	330	1136	11885	4102
KWKWQW	387	383	1545	1957	28	25	41	21	112	221	3098	2408
KIKQIIN	323	1079	64	61	12	2	16	8	52	91	601	454
CKAKAQANMWQ	600	134	9	7	6	3	480	345	858	1579	5432	2557

Microscopy images of µm-sized peptide aggregation

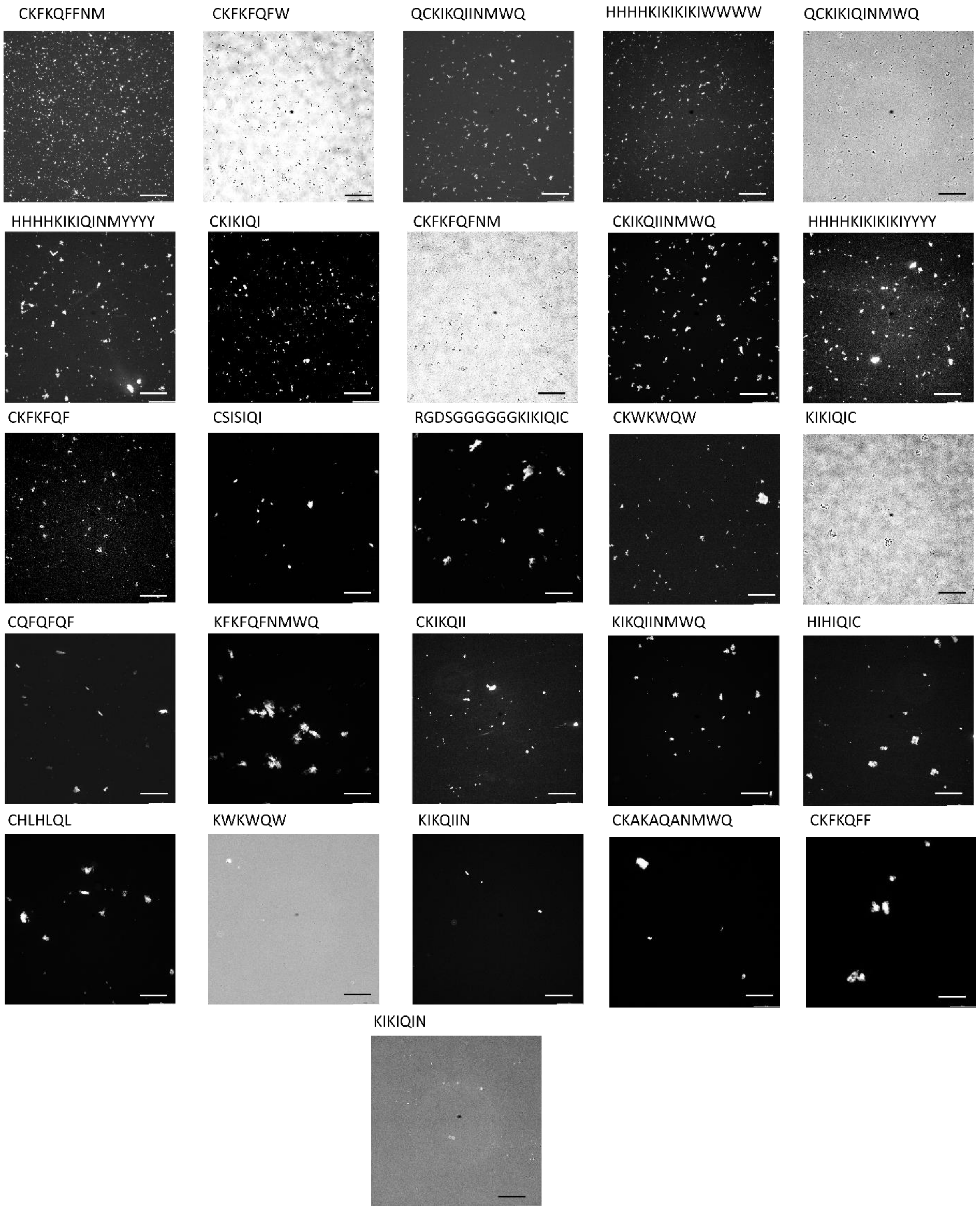

Figure S32 Representative selection of widefield microscopy images of peptides from the peptide library. Images with a dark background (white scale bar) are fluorescence microscopy images, and images with a bright background (black scale bar) are brightfield microscopy images. Peptides were diluted from 10 mg/mL in DMSO to 1 mg/mL in PBS and incubated for 1d at RT. Before the measurement they were diluted to 0.1 mg/mL with 50 µM ThT-solution in PBS, scale bar 200 µm. Microscopy measurements were conducted once with at least three microscopy images recorded for each peptide sample.

TEM micrographs of peptide library

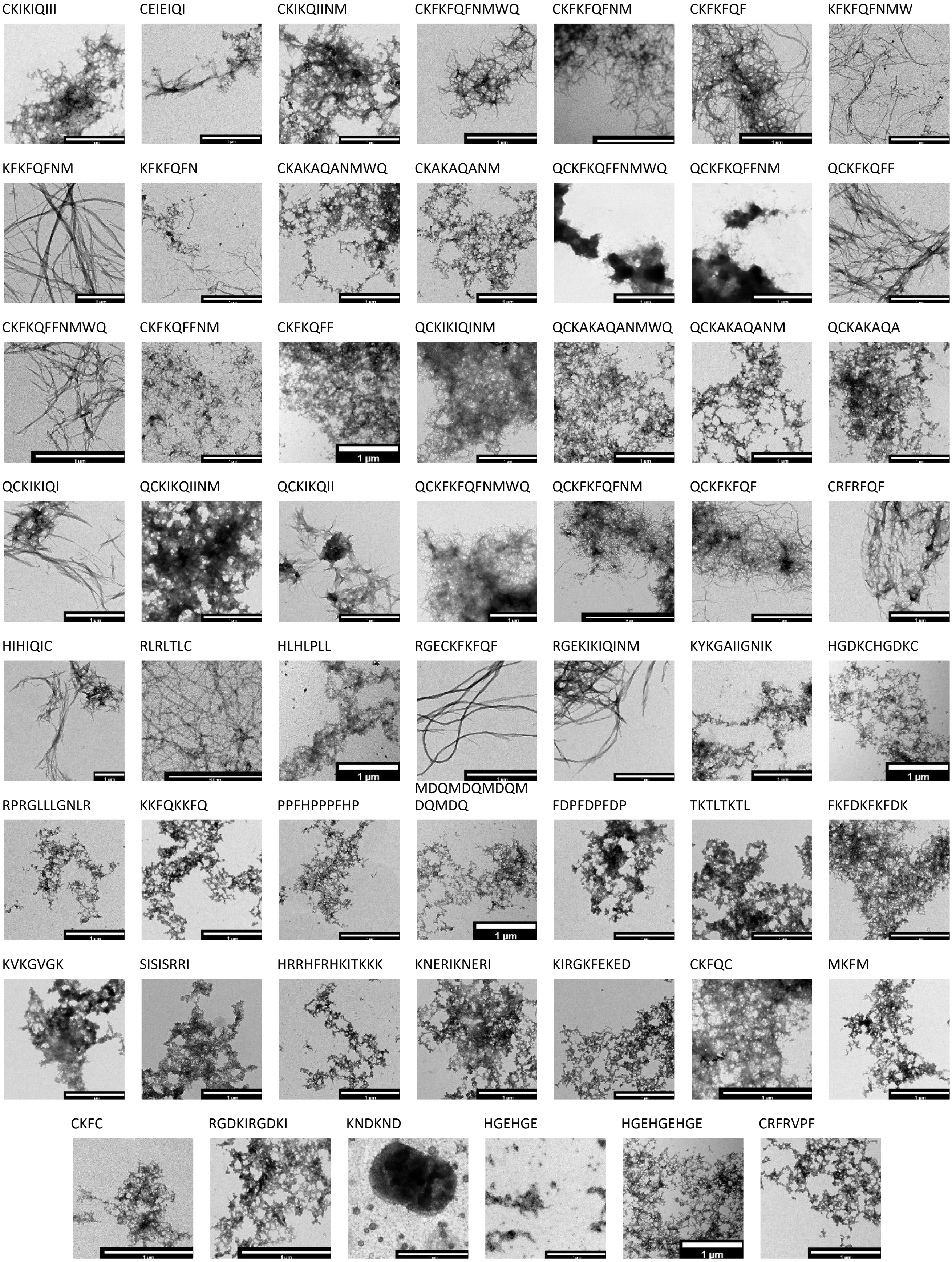

Figure S33 TEM micrographs of peptide library. Peptides were diluted from 10 mg/mL in DMSO to 1 mg/mL in PBS and incubated for 1d at RT, scale bar 1 µm. TEM measurements were conducted once with at least three microscopy images recorded for each peptide sample.

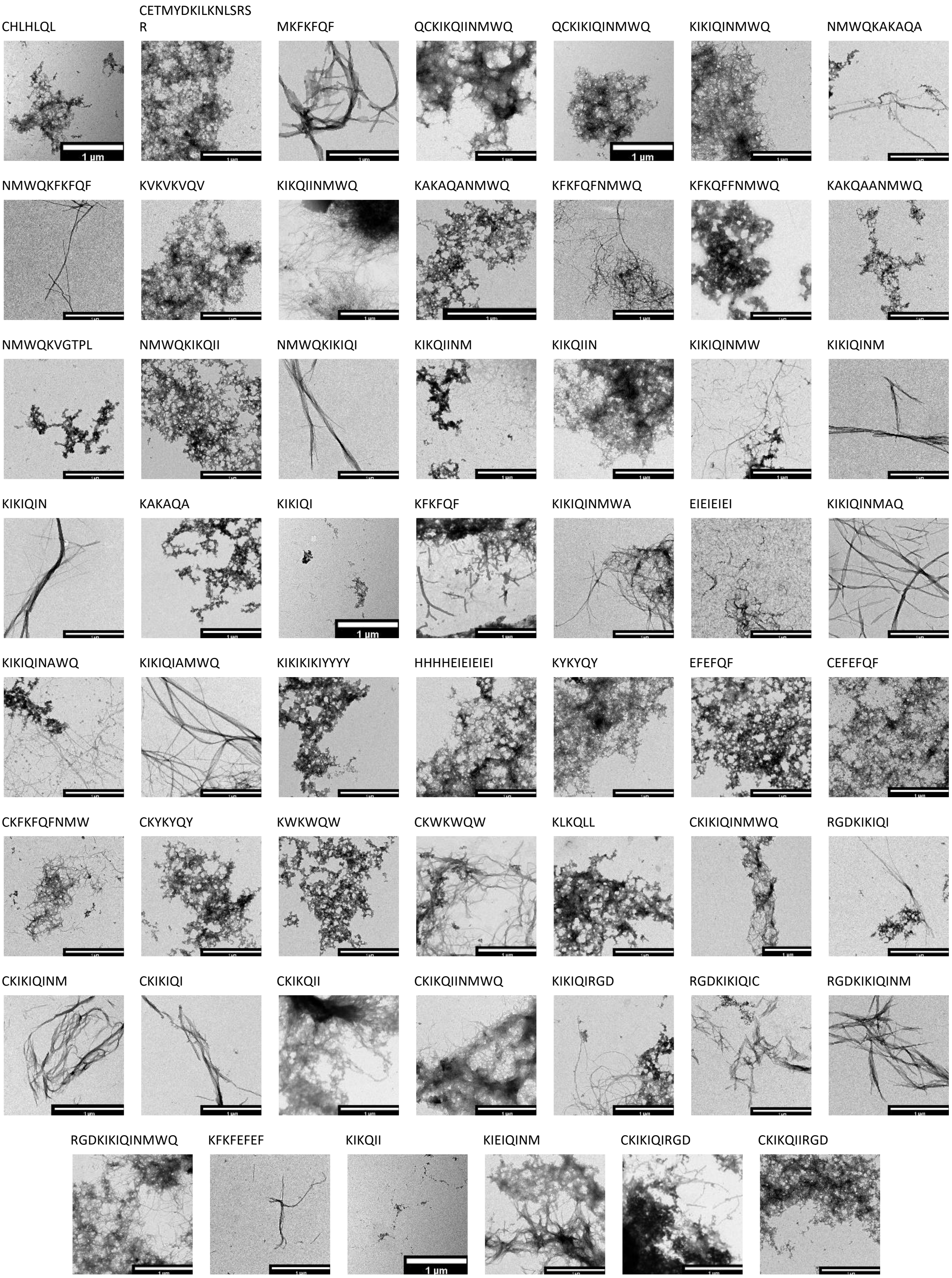

Figure S33 cont. TEM micrographs of peptide library. Peptides were diluted from 10 mg/mL in DMSO to 1 mg/mL in PBS and incubated for 1d at RT, scale bar 1 µm. TEM measurements were conducted once with at least three microscopy images recorded for each peptide sample.

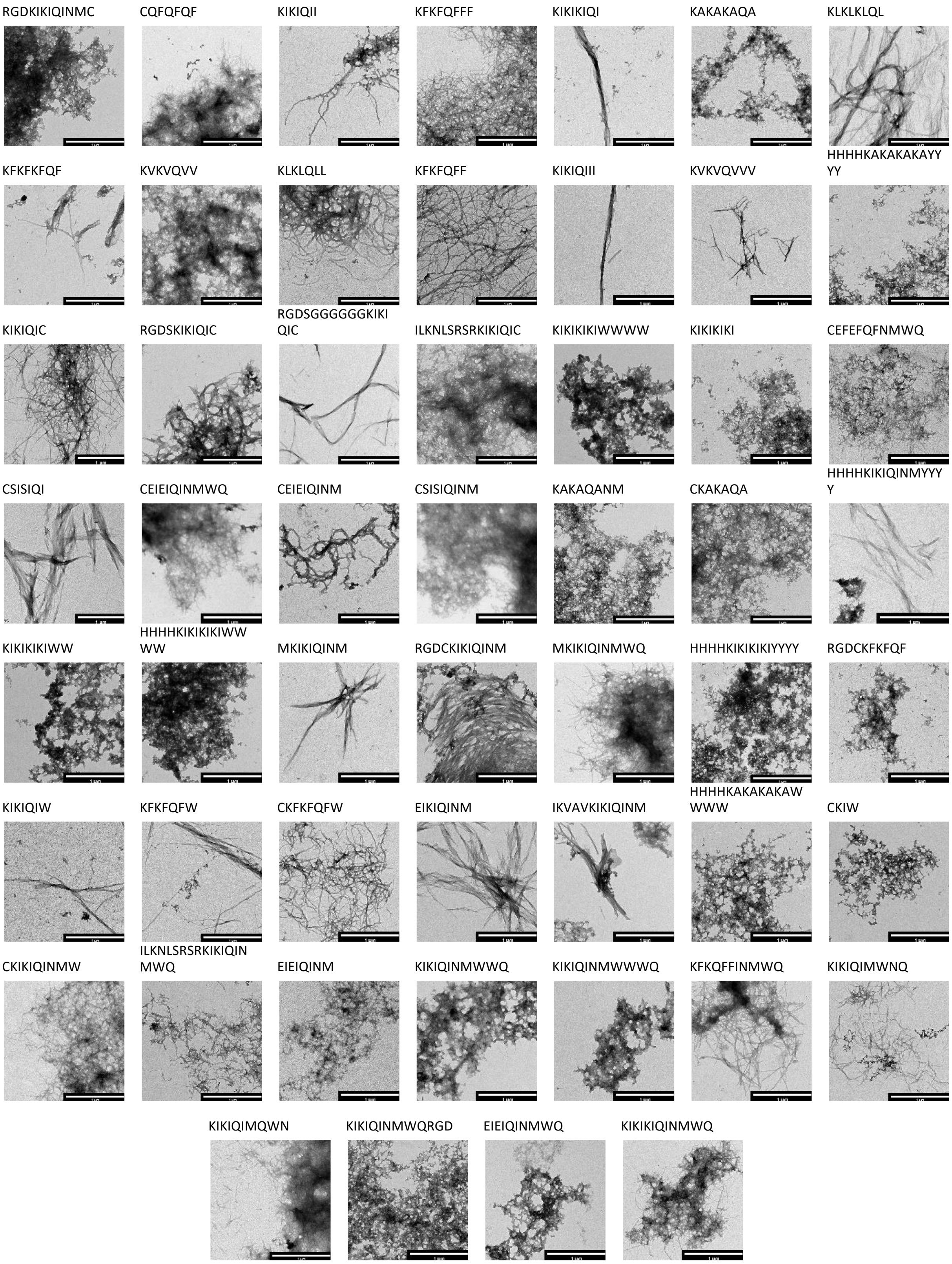

Figure S33 cont. TEM micrographs of peptide library. Peptides were diluted from 10 mg/mL in DMSO to 1 mg/mL in PBS and incubated for 1d at RT, scale bar 1 µm. TEM measurements were conducted once with at least three microscopy images recorded for each peptide sample.

FT-IR of Peptide Library

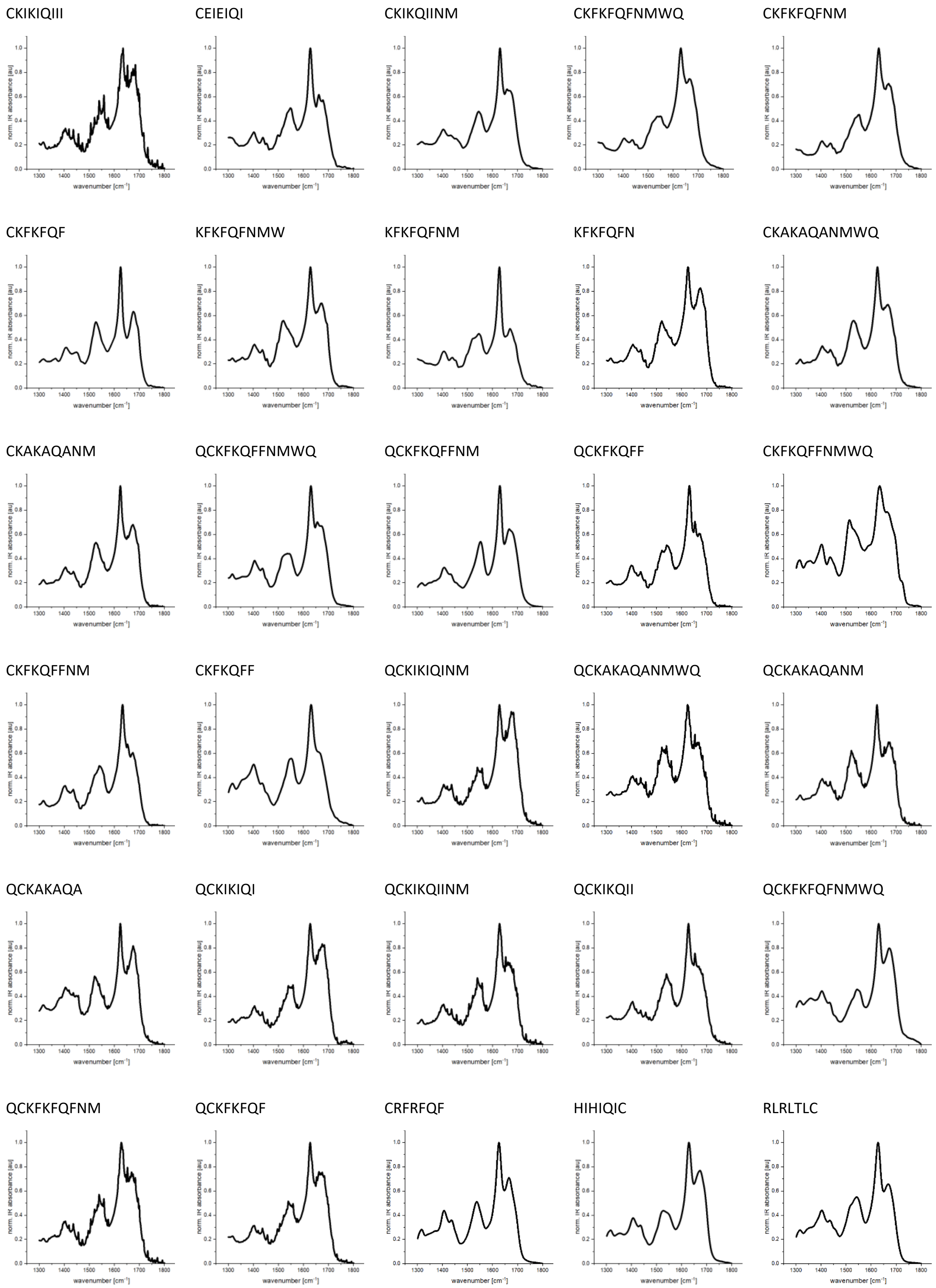

Figure S34 ATR FT-IR-measurement of peptide library. Each graph shows infrared absorbance from 1300–1800 cm^{-1} normalized to the maximum of the amide I region. For the measurement peptides were lyophilized after diluted from 10 mg/mL in DMSO to 1 mg/mL in PBS and incubated for 1d at RT. Source data for Figure S34 is provided.

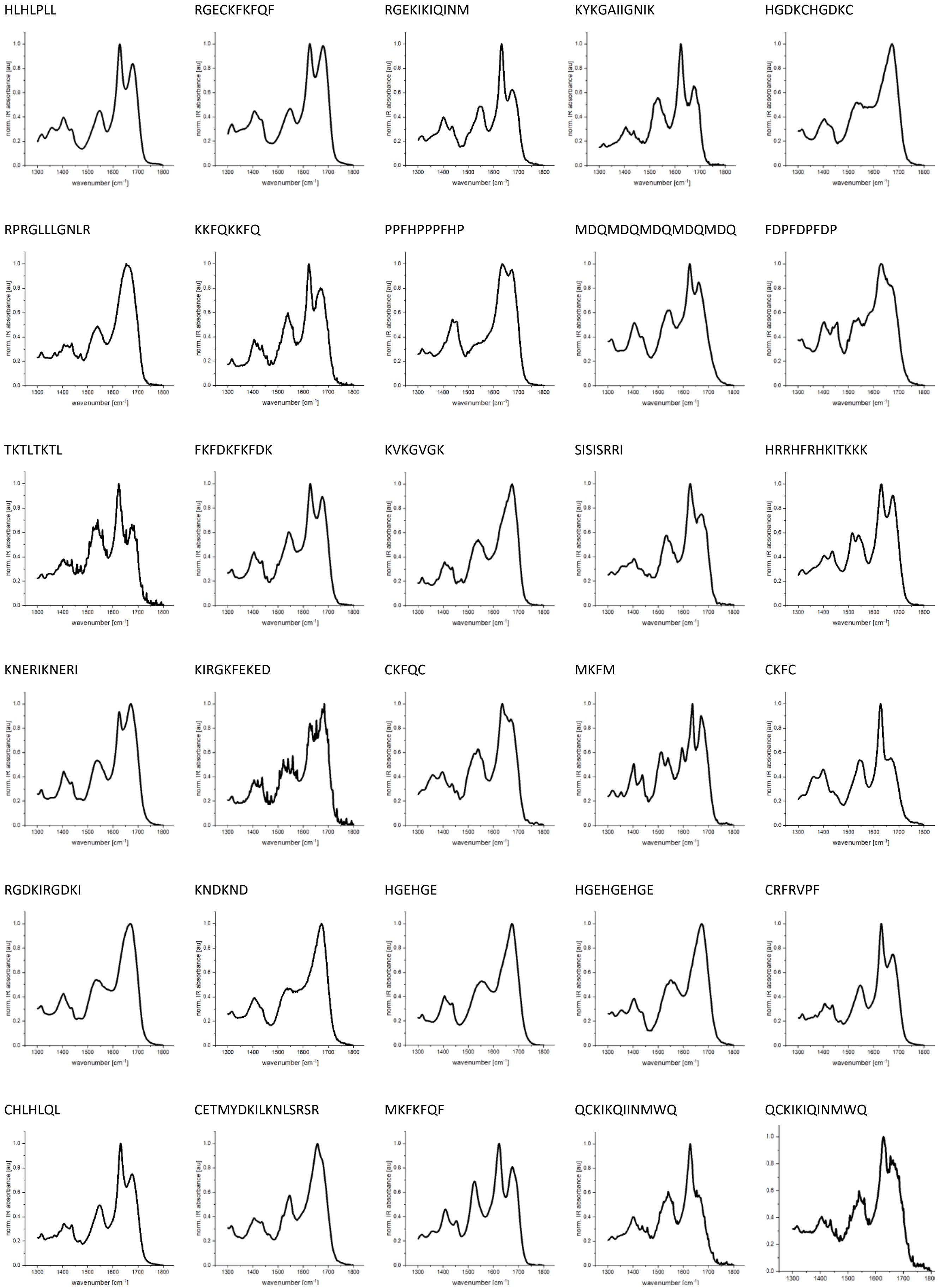

Figure S34 cont. ATR FT-IR-measurement of peptide library. Each graph shows infrared absorbance from 1300–1800 cm^{-1} normalized to the maximum of the amide I region. For the measurement peptides were lyophilized after diluted from 10 mg/mL in DMSO to 1 mg/mL in PBS and incubated for 1d at RT. Source data for Figure S34 is provided.

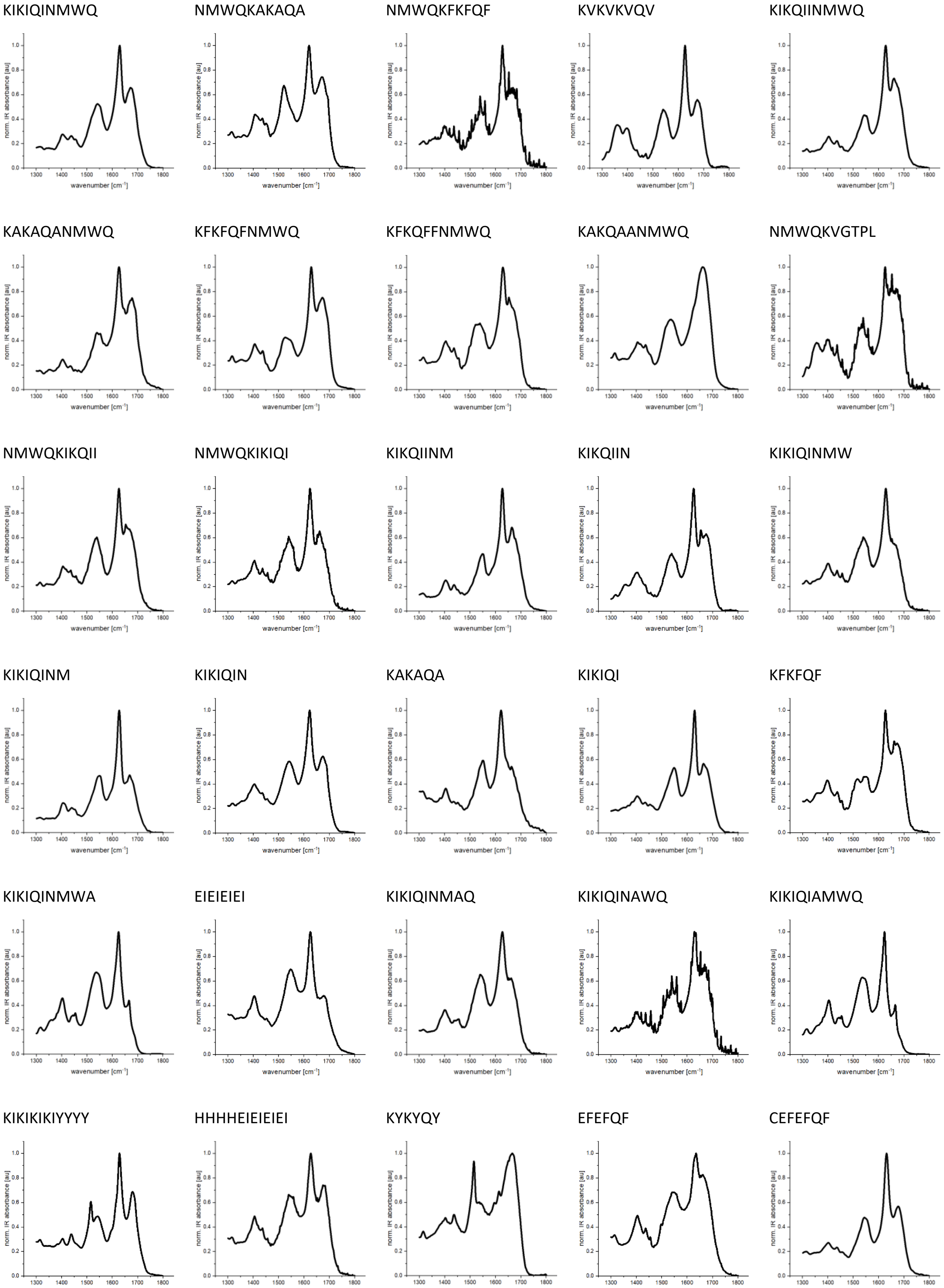

Figure S34 cont. ATR FT-IR-measurement of peptide library. Each graph shows infrared absorbance from 1300–1800 cm^{-1} normalized to the maximum of the amide I region. For the measurement peptides were lyophilized after diluted from 10 mg/mL in DMSO to 1 mg/mL in PBS and incubated for 1d at RT. Source data for Figure S34 is provided.

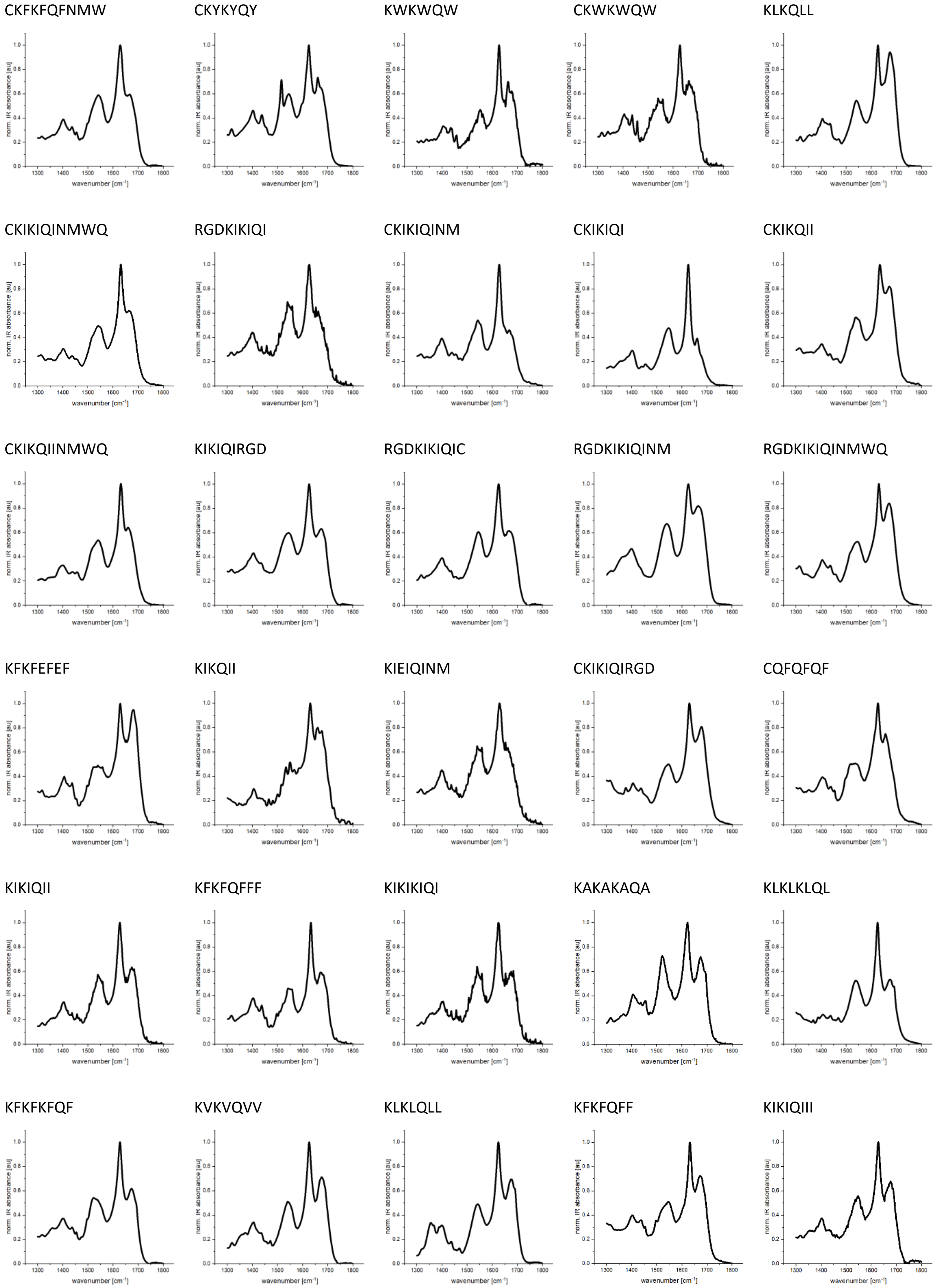

Figure S34 cont. ATR FT-IR-measurement of peptide library. Each graph shows infrared absorbance from 1300–1800 cm^{-1} normalized to the maximum of the amide I region. For the measurement peptides were lyophilized after diluted from 10 mg/mL in DMSO to 1 mg/mL in PBS and incubated for 1d at RT. Source data for Figure S34 is provided.

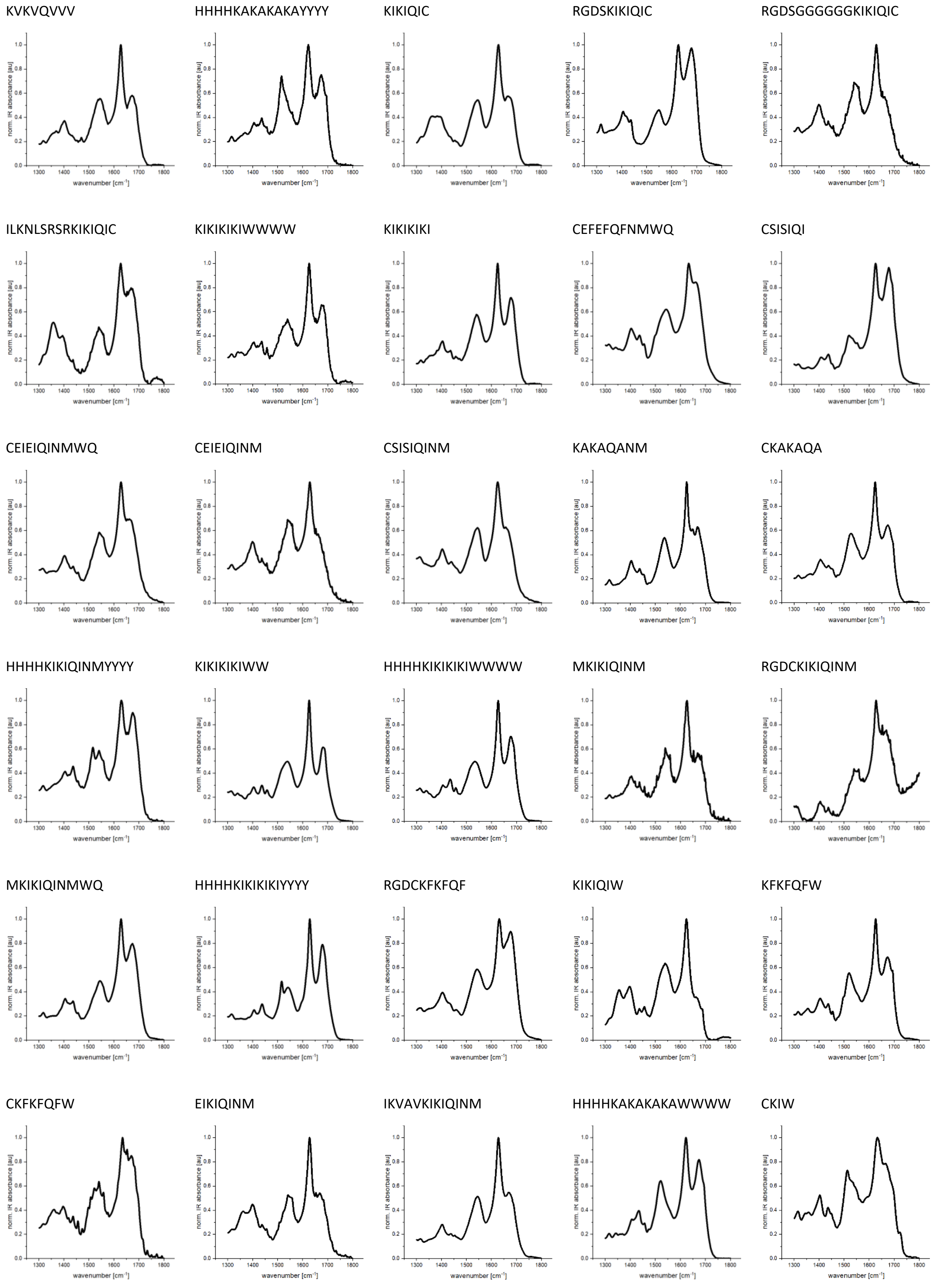

Figure S34 cont. ATR FT-IR-measurement of peptide library. Each graph shows infrared absorbance from 1300–1800 cm^{-1} normalized to the maximum of the amide I region. For the measurement peptides were lyophilized after diluted from 10 mg/mL in DMSO to 1 mg/mL in PBS and incubated for 1d at RT. Source data for Figure S34 is provided.

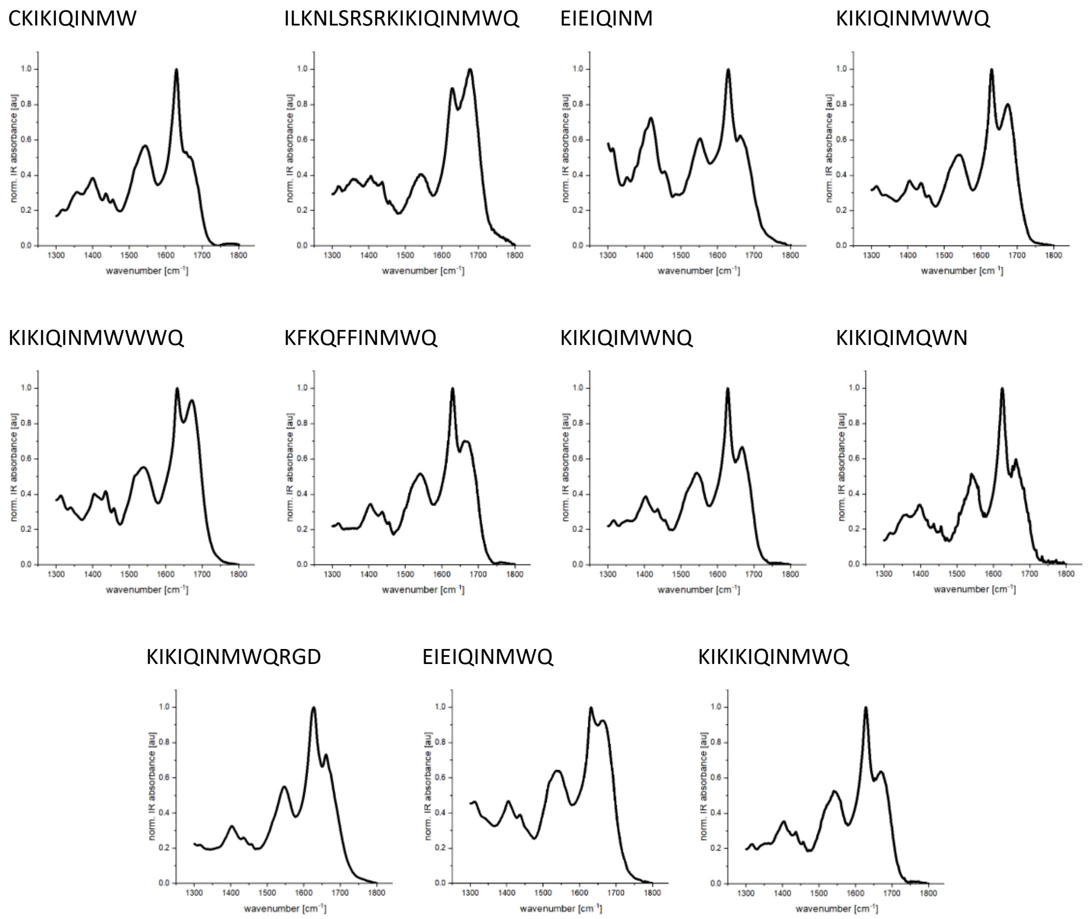

Figure S34 cont. ATR FT-IR-measurement of peptide library. Each graph shows infrared absorbance from 1300–1800 cm^{-1} normalized to the maximum of the amide I region. For the measurement peptides were lyophilized after diluted from 10 mg/mL in DMSO to 1 mg/mL in PBS and incubated for 1d at RT. Source data for Figure S34 is provided.

LC-MS measurement of synthesized peptides

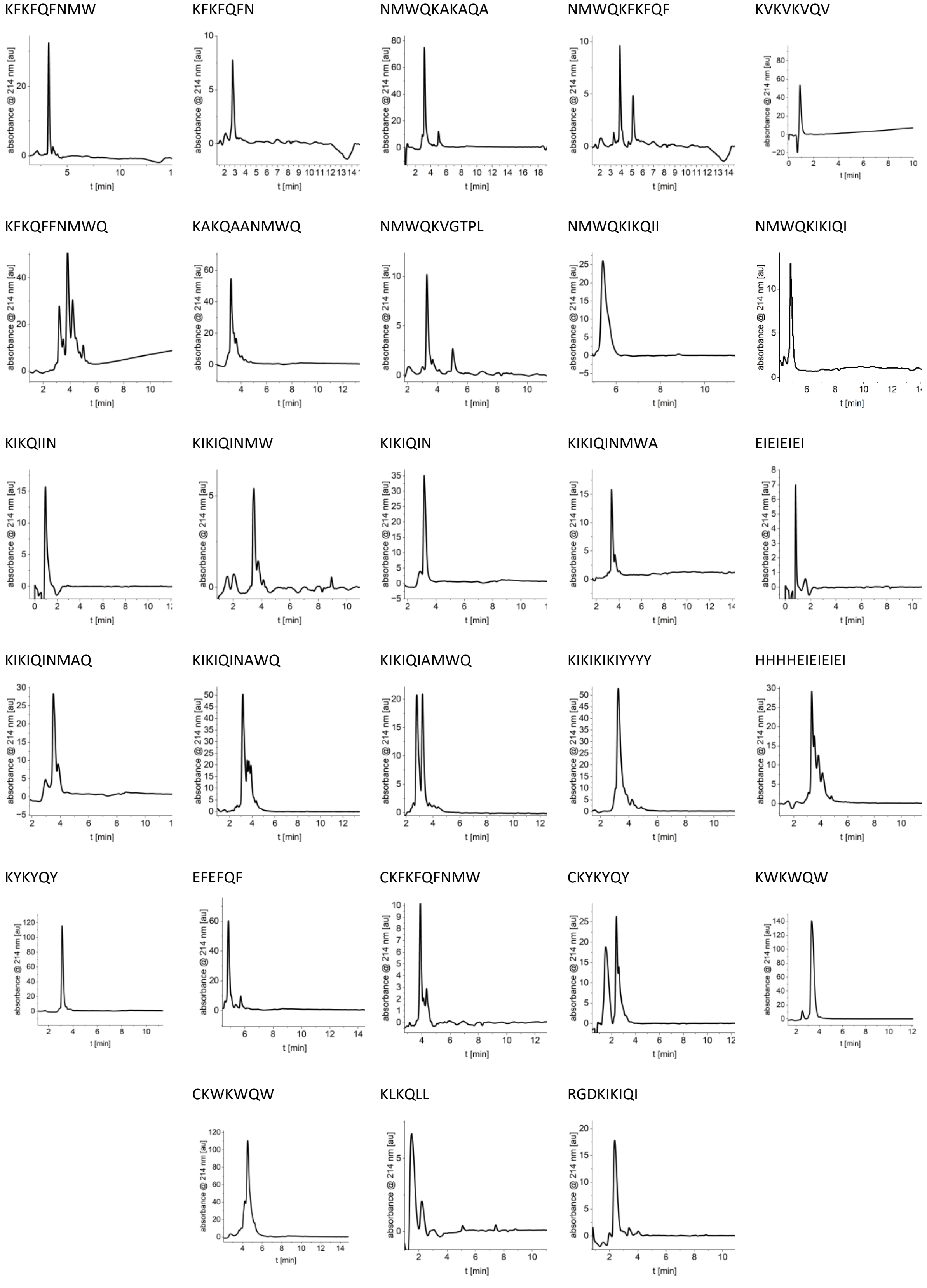

Figure S35 LC-MS chromatogram of peptides, which were not commercially obtained or reported before. Source data for Figure S35 is provided.

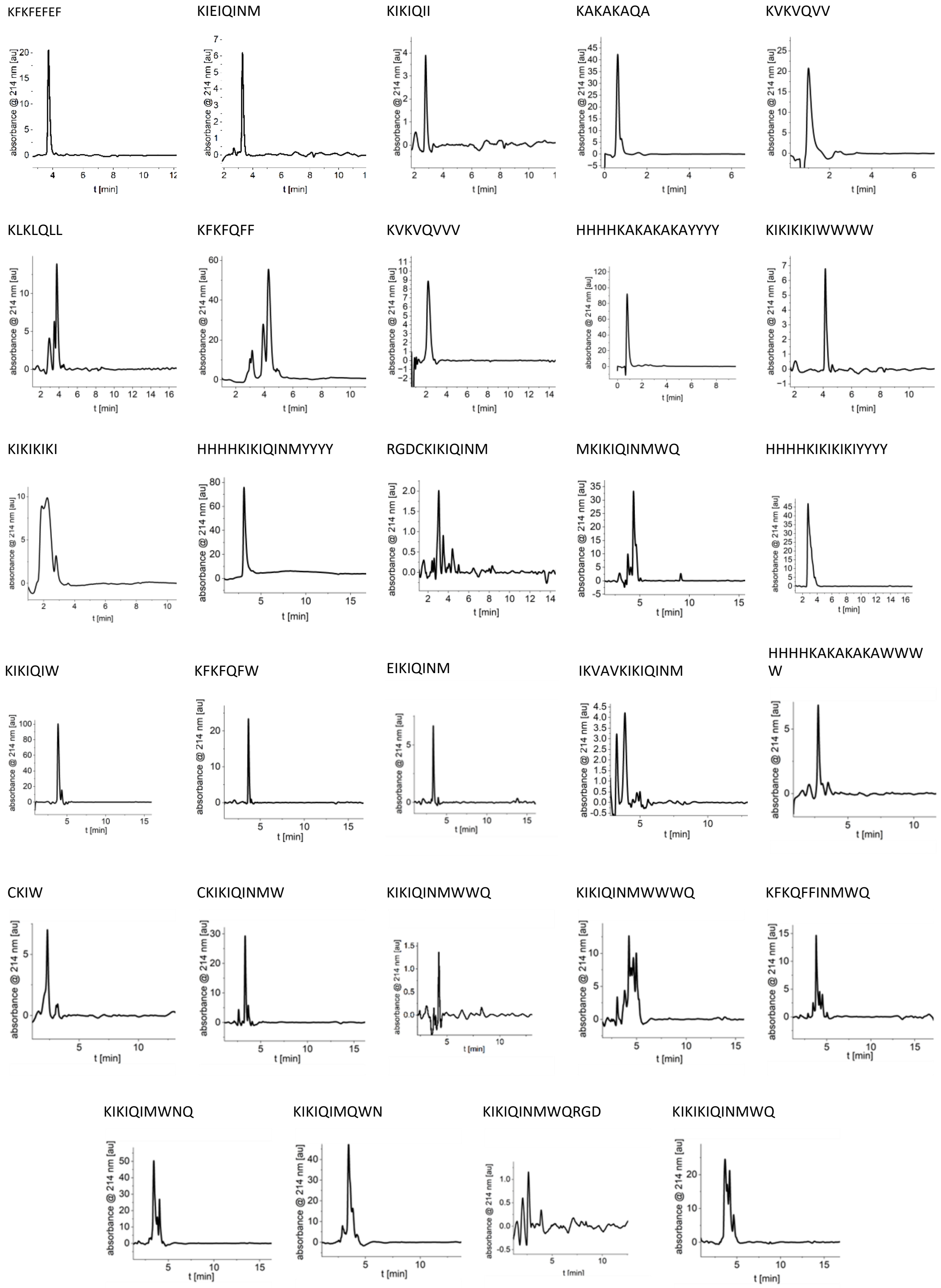

Figure S35 cont. LC-MS chromatogram of peptides, which were not commercially obtained or reported before. Source data for Figure S35 is provided.

TEM of literature derived amyloid peptides

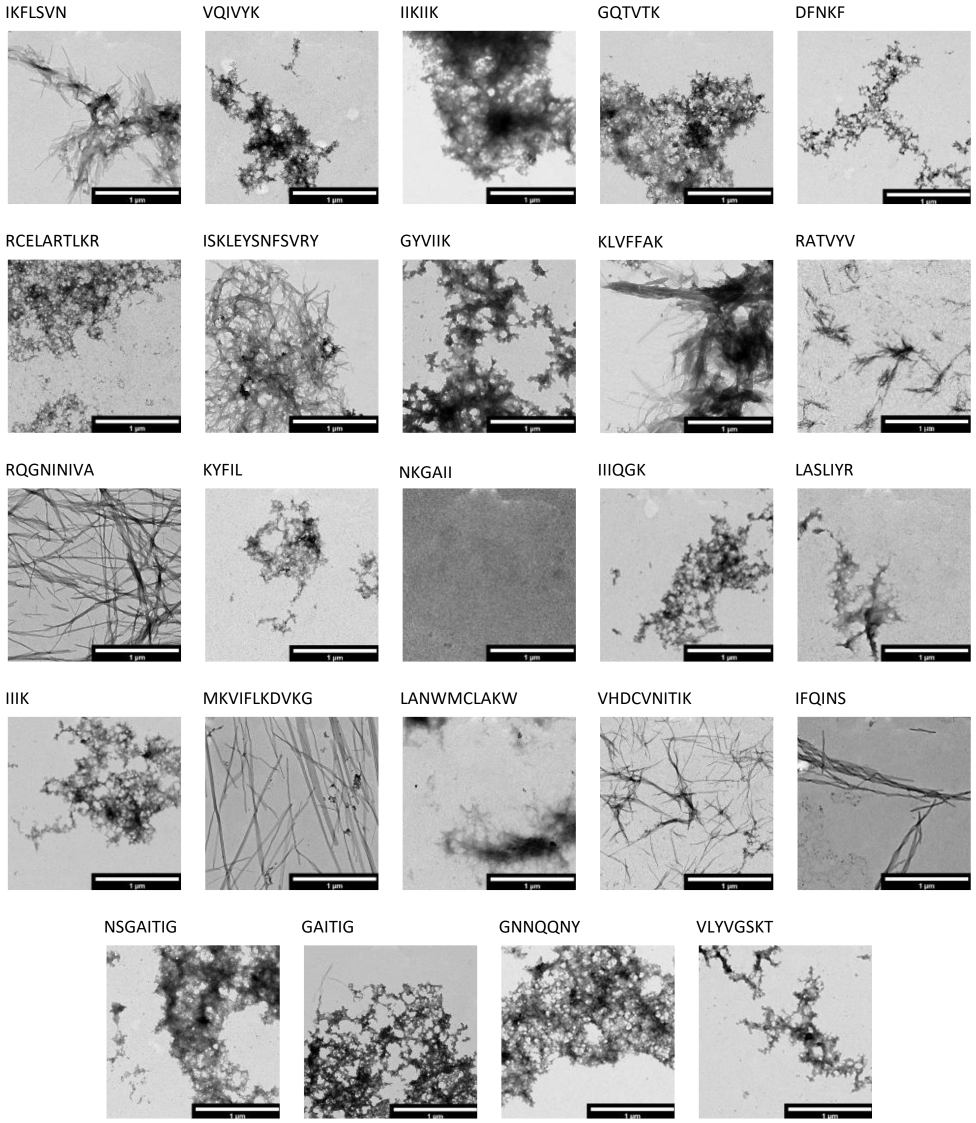

Figure S36 TEM images of literature derived peptides prepared according to standard procedure from 10 mg/mL DMSO diluted in PBS to 1mg/mL. TEM measurements were conducted once with at least three microscopy images recorded for each peptide sample.

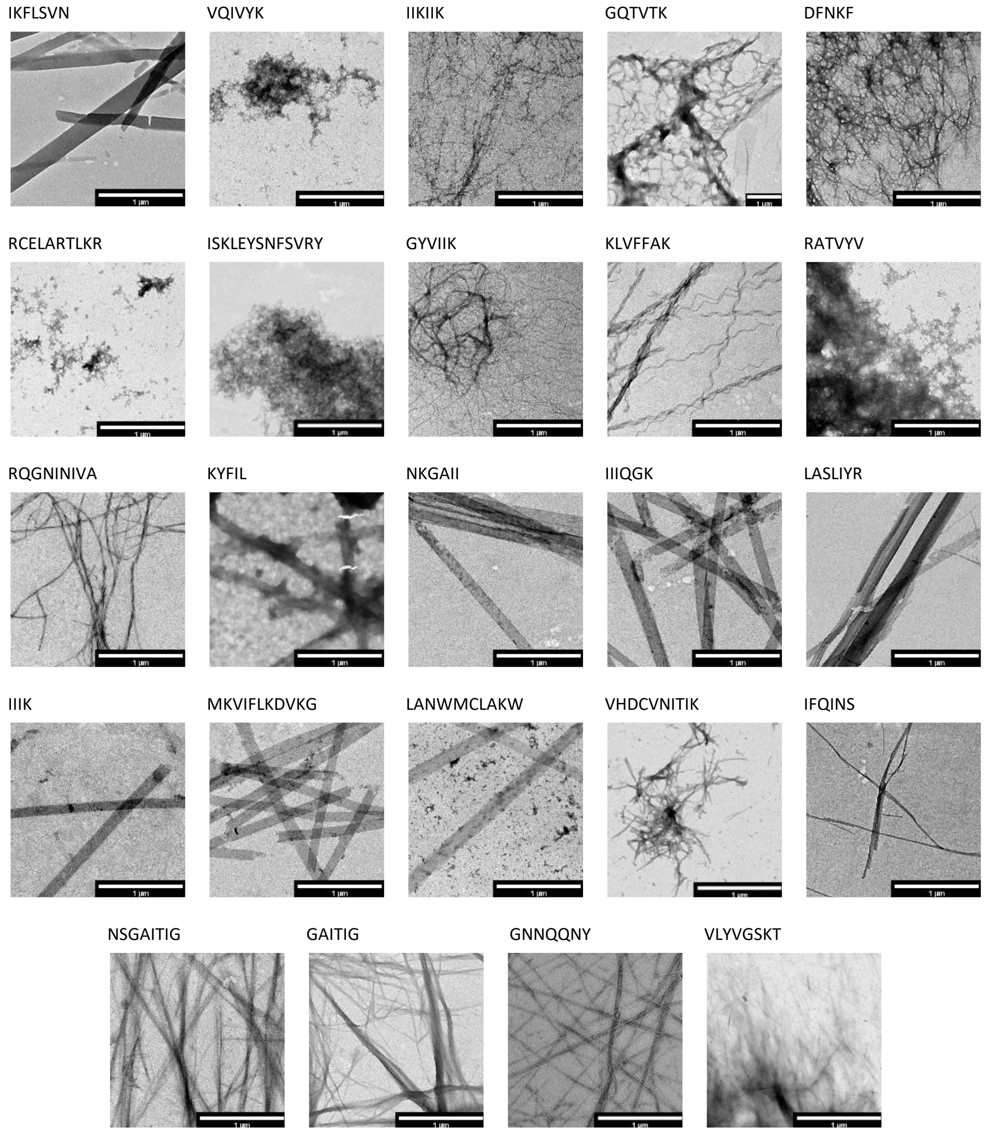

Figure S37 TEM images of literature derived peptides prepared according to literature protocol. TEM measurements were conducted once with at least three microscopy images recorded for each peptide sample.

FT-IR of peptides designed via pattern analysis

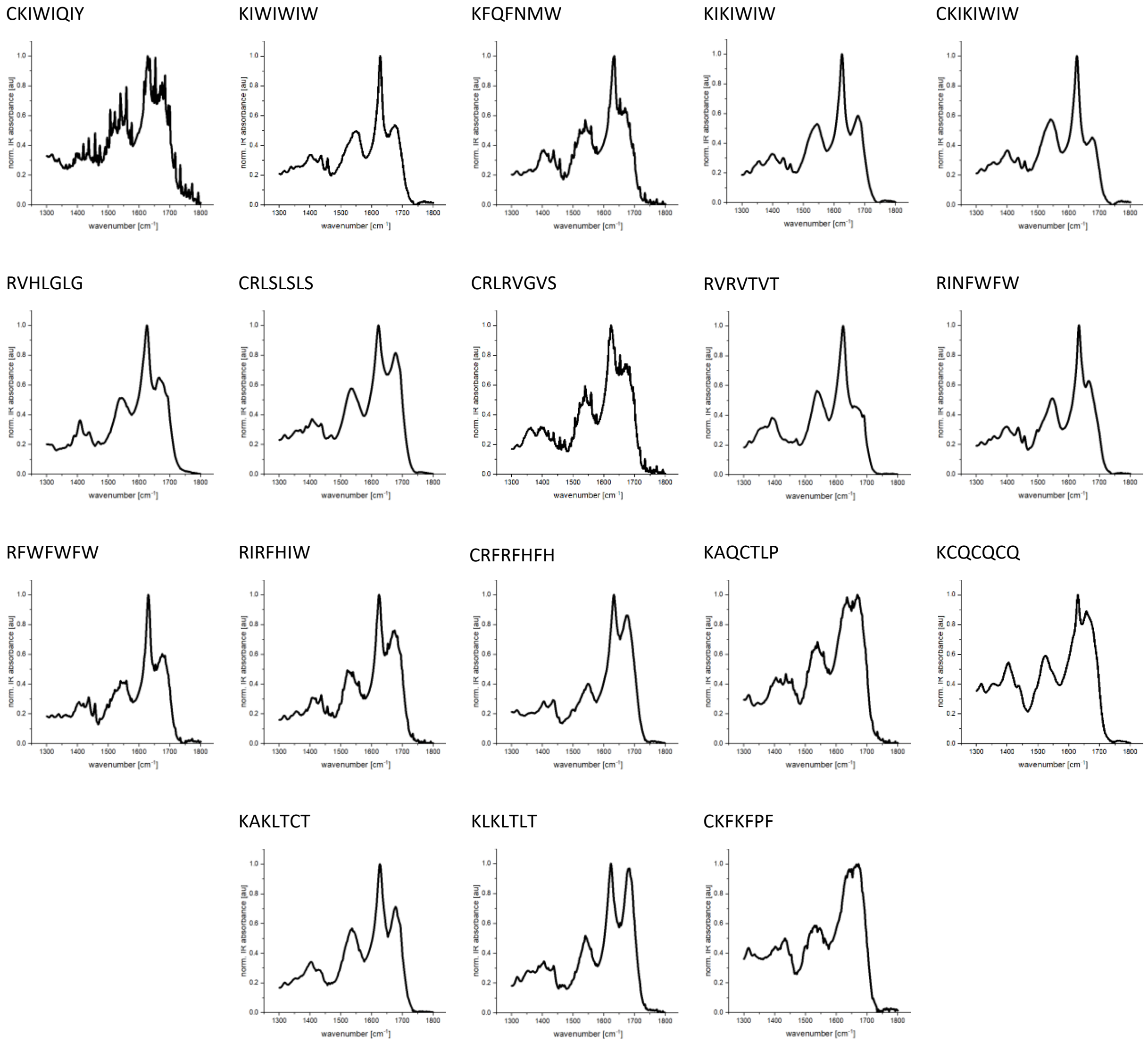

Figure S38 ATR FT-IR measurements of peptides designed via pattern analysis prepared according to standard procedure from 10 mg/mL DMSO diluted in PBS to 1 mg/mL. Source data for Figure S38 is provided.

FT-IR of literature derived peptides

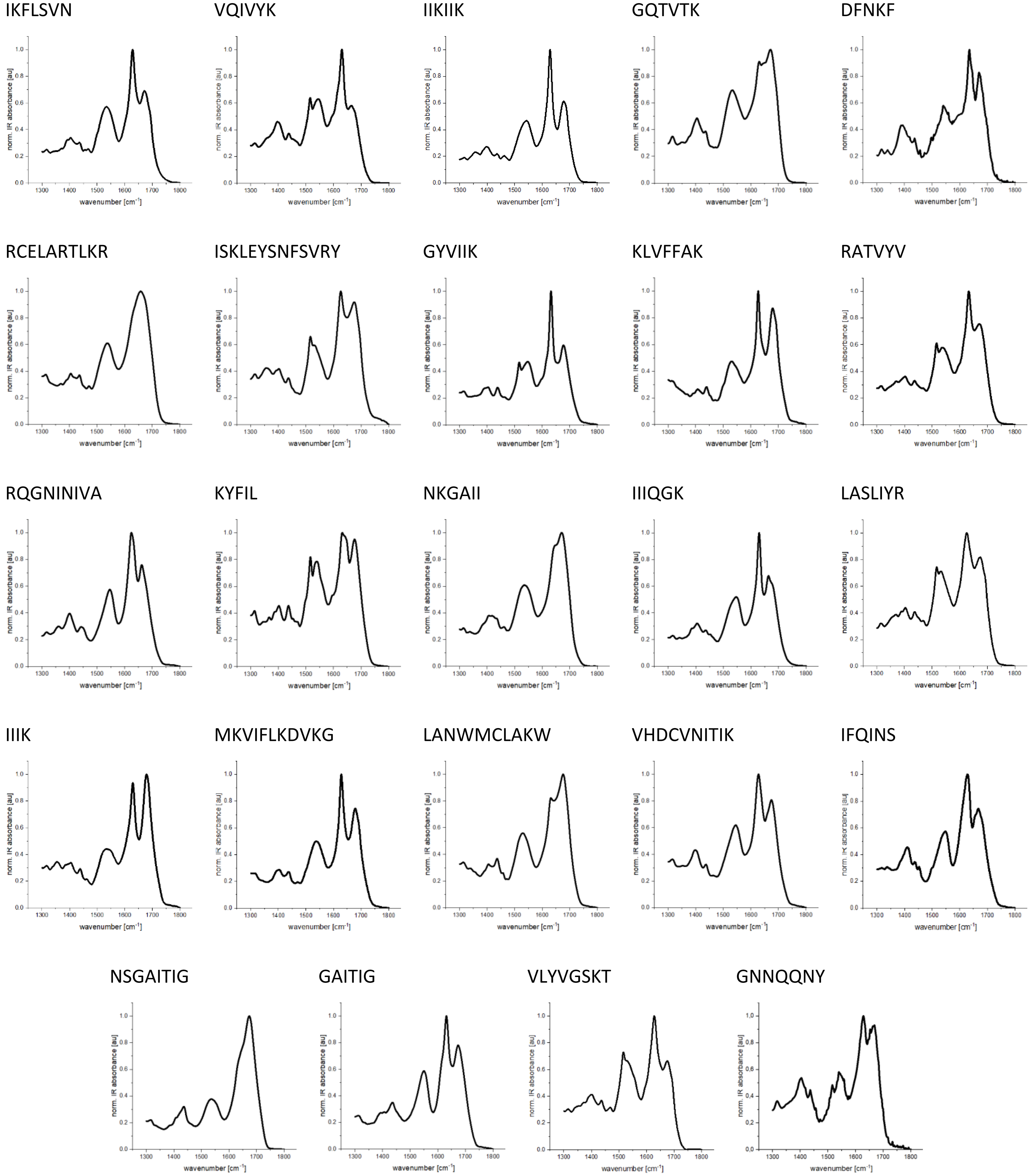

Figure S39 ATR FT-IR measurements of literature peptides prepared according to standard procedure from 10 mg/mL DMSO diluted in PBS to 1 mg/mL. Source data for Figure S39 is provided.

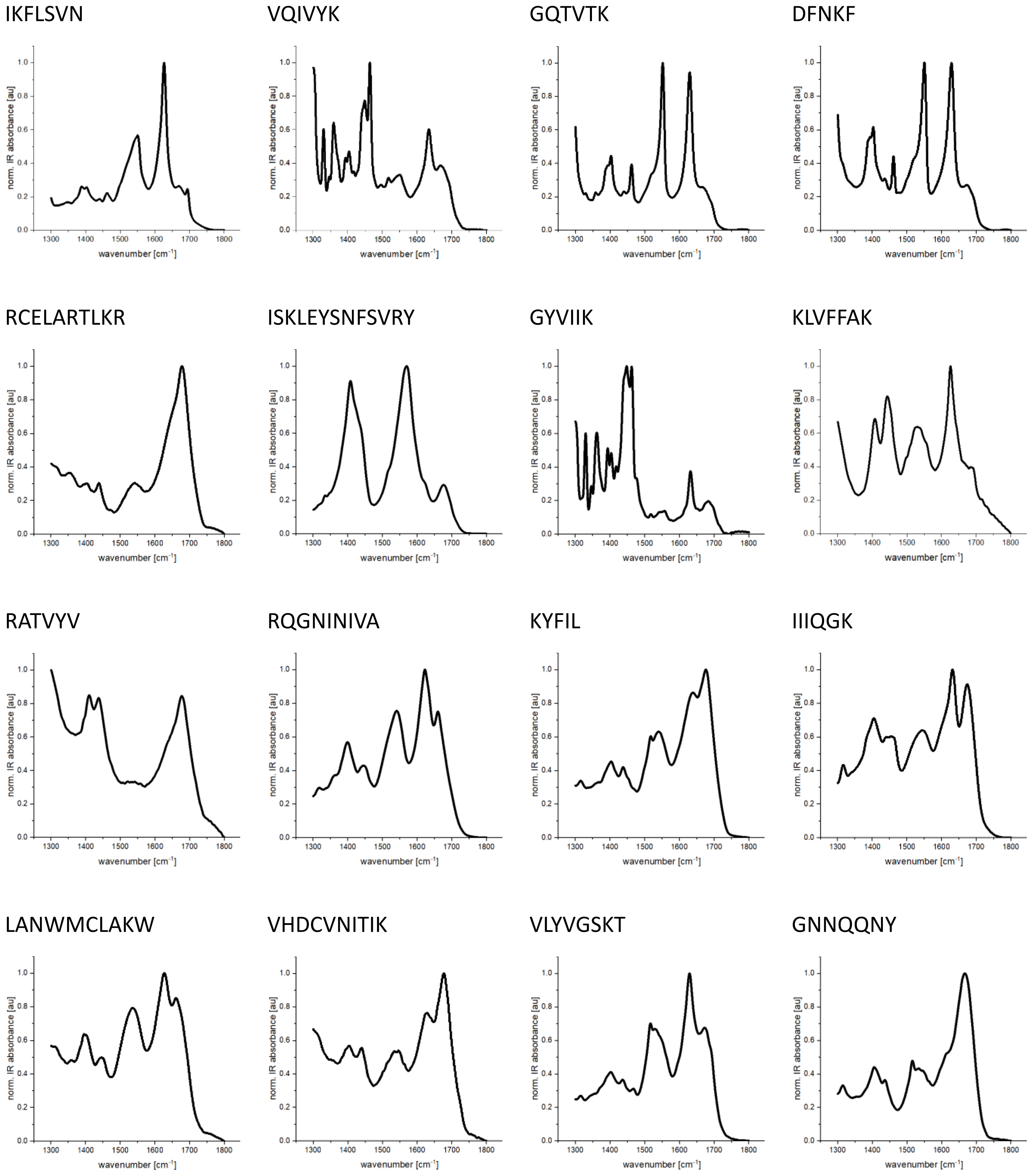

Figure S39 cont. ATR FT-IR measurements of literature peptides prepared according to literature protocol. Source data for Figure S39 is provided.

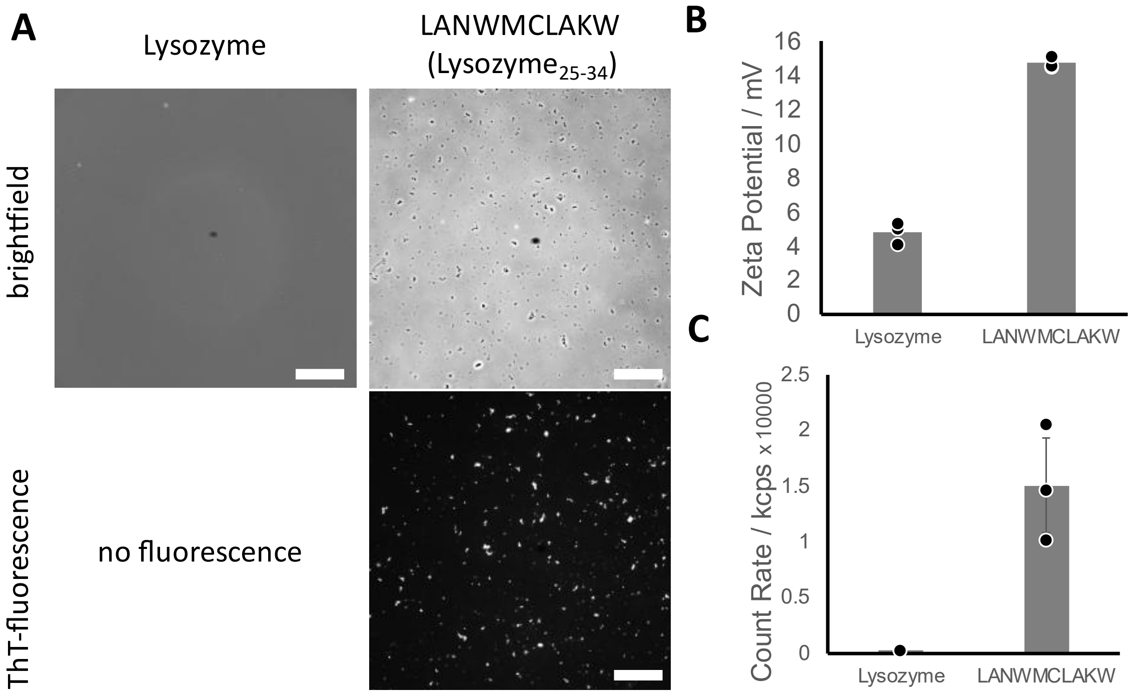

Figure S40 Comparison of physicochemical properties of the full protein lysozyme and a sequence fragment Lysozyme$_{25-34}$ LANWMCLAKW. **A** No µm-sized aggregation or ThT-fluorescence can be observed for lysozyme, while LANWMCLAKW is yielding clearly visible µm-sized aggregates in brightfield microscopy and ThT-fluorescent aggregates via fluorescence microscopy, scale bar 200 µm. Preparation of both samples at 809.7 µM (1 mg/mL LANWMCLAKW) in PBS (10 vol% DMSO), incubation for 1d at rt. Just before microscopy measurement samples were diluted to 80.9 µM with 50 µM ThT-dye solution in PBS. **B** Zeta-Potential for lysozyme and LANWMCLAKW and **C** derived Count Rate of scattered light for lysozyme and LANWMCLAKW from zeta-potential measurements. Error bars indicate Std. Dev. from triplicate measurements. Samples were incubated at 809.7 µM in PBS (10 vol% DMSO), for 1d at rt before diluting for zeta-potential measurement samples to 80.9 µM with 1 mM KCl. Mean values are displayed with error bars indicating standard deviation from triplicate measurements. Source data for Figure S40 B, C is provided.

Supplementary References

1. Yoshimura, Y. *et al.* Distinguishing crystal-like amyloid fibrils and glass-like amorphous aggregates from their kinetics of formation. *Proc. Natl. Acad. Sci. U. S. A.* **109**, 14446–14451 (2012).

2. Maurer-Stroh, S. *et al.* Exploring the sequence determinants of amyloid structure using position-specific scoring matrices. *Nat. Methods* **7**, 237–242 (2010).

3. Adachi, M., So, M., Sakurai, K., Kardos, J. & Goto, Y. Supersaturation-limited and Unlimited Phase Transitions Compete to Produce the Pathway Complexity in Amyloid Fibrillation. *J. Biol. Chem.* **290**, 18134–18145 (2015).

4. Ke, P. C. *et al.* Half a century of amyloids: past, present and future. *Chem. Soc. Rev.* **49**, 5473–5509 (2020).

5. Biancalana, M., Makabe, K., Koide, A. & Koide, S. Molecular Mechanism of Thioflavin-T Binding to the Surface of β-Rich Peptide Self-Assemblies. *J. Mol. Biol.* **385**, 1052–1063 (2009).

6. Naiki, H., Higuchi, K., Hosokawa, M. & Takeda, T. Fluorometric determination of amyloid fibrils in vitro using the fluorescent dye, thioflavine T. *Anal. Biochem.* **177**, 244–249 (1989).

7. Sidhu, A., Vaneyck, J., Blum, C., Segers-Nolten, I. & Subramaniam, V. Polymorph-specific distribution of binding sites determines thioflavin-T fluorescence intensity in α-synuclein fibrils. *Amyloid* **25**, 189–196 (2018).

8. Arad, E., Green, H., Jelinek, R. & Rapaport, H. Revisiting thioflavin T (ThT) fluorescence as a marker of protein fibrillation – The prominent role of electrostatic interactions. *J. Colloid Interface Sci.* **573**, 87–95 (2020).

9. Namioka, S., Yoshida, N., Konno, H. & Makabe, K. Residue-Specific Binding Mechanisms of Thioflavin T to a Surface of Flat β-Sheets within a Peptide Self-Assembly Mimic. *Biochemistry* **59**, 2782–2787 (2020).

10. Zhao, R. *et al.* Measurement of amyloid formation by turbidity assay—seeing through the cloud. *Biophys. Rev.* **8**, 445–471 (2016).

11. Pignataro, M. F., Herrera, M. G. & Dodero, V. I. Evaluation of Peptide/Protein Self-Assembly and Aggregation by Spectroscopic Methods. *Molecules* **25**, 4854 (2020).

12. Stetefeld, J., McKenna, S. A. & Patel, T. R. Dynamic light scattering: a practical guide and applications in biomedical sciences. *Biophys. Rev.* **8**, 409–427 (2016).

13. Malm, A. V. & Corbett, J. C. W. Improved Dynamic Light Scattering using an adaptive and statistically driven time resolved treatment of correlation data. *Sci. Rep.* **9**, 1–11 (2019).

14. Martinek, T. A. *et al.* Secondary Structure Dependent Self-Assembly of β-Peptides into Nanosized Fibrils and Membranes. *Angew. Chemie* **118**, 2456–2460 (2006).

15. Toksoz, S., Mammadov, R., Tekinay, A. B. & Guler, M. O. Electrostatic effects on nanofiber formation of self-assembling peptide amphiphiles. *J. Colloid Interface Sci.* **356**, 131–137 (2011).

16. Chen, Y., Gan, H. X. & Tong, Y. W. pH-controlled hierarchical self-assembly of peptide amphiphile. *Macromolecules* **48**, 2647–2653 (2015).

17. Fauchère, J.-L., Charton, M., Kier, L. B., Verloop, A. & Pliska, V. Amino acid side chain parameters for correlation studies in biology and pharmacology. *Int. J. Pept. Protein Res.* **32**, 269–278 (2009).

18. Osorio, D., Rondón-Villarreal, P. & Torres, R. Peptides: A Package for Data Mining of Antimicrobial Peptides. *R J.* **7**, 4 (2015).

19. Kyte, J. & Doolittle, R. F. A simple method for displaying the hydropathic character of a protein. *J. Mol. Biol.* **157**, 105–132 (1982).

20. Ikai, A. Thermostability and Aliphatic Index of Globular Proteins. *J. Biochem.* **88**, 1895–1898 (1980).

21. Eisenberg, D., Weiss, R. M. & Terwilliger, T. C. The helical hydrophobic moment: a measure of the amphiphilicity of a helix. *Nature* **299**, 371–374 (1982).

22. Boman, H. G. Antibacterial peptides: basic facts and emerging concepts. *J. Intern. Med.* **254**, 197–215 (2003).

23. Guruprasad, K., Reddy, B. V. B. & Pandit, M. W. Correlation between stability of a protein and its dipeptide composition: a novel approach for predicting in vivo stability of a protein from its primary sequence. *Protein Eng. Des. Sel.* **4**, 155–161 (1990).

24. Sheik, D. A., Dewhurst, S. & Yang, J. Natural Seminal Amyloids as Targets for Development of Synthetic Inhibitors of HIV Transmission. *Acc. Chem. Res.* **50**, 2159–2166 (2017).

25. Lee, Y.-H. & Ramamoorthy, A. Semen-derived amyloidogenic peptides-Key players of HIV infection. *Protein Sci.* **27**, 1151–1165 (2018).

26. Tan, S. *et al.* Peptides derived from HIV-1 gp120 co-receptor binding domain form amyloid fibrils and enhance HIV-1 infection. *FEBS Lett.* **588**, 1515–1522 (2014).

27. Hauber, I., Hohenberg, H., Holstermann, B., Hunstein, W. & Hauber, J. The main green tea polyphenol epigallocatechin-3-gallate counteracts semen-mediated enhancement of HIV infection. *Proc. Natl. Acad. Sci.* **106**, 9033–9038 (2009).

28. Viswanathan, G. *et al.* Inhibitory Effect of Naphthoquinone-Tryptophan Hybrid towards Aggregation of PAP f39 Semen Amyloid. *Molecules* **23**, 3279 (2018).

29. Schilling, C. *et al.* Sequence-Optimized Peptide Nanofibers as Growth Stimulators for Regeneration of Peripheral Neurons. *Adv. Funct. Mater.* **29**, 1809112 (2019).

30. Kaygisiz, K. *et al.* Data-Mining Unveils Structure-Property-Activity Correlation of Viral Infectivity Enhancing Self-Assembling Peptides. *Zenodo.org* doi:10.5281/ZENODO.8079727 (2023).

31. Mendes, A. C., Baran, E. T., Reis, R. L. & Azevedo, H. S. Self-assembly in nature: using the principles of nature to create complex nanobiomaterials. *Wiley Interdiscip. Rev. Nanomed. Nanobiotechnol.* **5**, 582–612 (2013).

32. Fujiwara, K., Toda, H. & Ikeguchi, M. Dependence of alpha-helical and beta-sheet amino acid propensities on the overall protein fold type. *BMC Struct. Biol.* **12**, 18 (2012).

33. Dai, B. *et al.* Tunable assembly of amyloid-forming peptides into nanosheets as a retrovirus carrier. *Proc. Natl. Acad. Sci. U. S. A.* **112**, 2996–3001 (2015).

34. Medini, K. *et al.* Controlling gelation with sequence: Towards programmable peptide

hydrogels. *Acta Biomater.* **43**, 30–37 (2016).

35. Kurnellas, M. P., Adams, C. M., Sobel, R. A., Steinman, L. & Rothbard, J. B. Amyloid Fibrils Composed of Hexameric Peptides Attenuate Neuroinflammation. *Sci. Transl. Med.* **5**, 179ra42-179ra42 (2013).

36. Salinas, N., Colletier, J.-P., Moshe, A. & Landau, M. Extreme amyloid polymorphism in Staphylococcus aureus virulent PSMα peptides. *Nat. Commun.* **9**, 3512 (2018).

37. Tayeb-Fligelman, E. *et al.* Inhibition of amyloid formation of the Nucleoprotein of SARS-CoV-2. *bioRxiv* 2021.03.05.434000 (2021) doi:10.1101/2021.03.05.434000.

38. Reches, M., Porat, Y. & Gazit, E. Amyloid Fibril Formation by Pentapeptide and Tetrapeptide Fragments of Human Calcitonin. *J. Biol. Chem.* **277**, 35475–35480 (2002).

39. Fernandez-Escamilla, A.-M., Rousseau, F., Schymkowitz, J. & Serrano, L. Prediction of sequence-dependent and mutational effects on the aggregation of peptides and proteins. *Nat. Biotechnol.* **22**, 1302–1306 (2004).

40. Maurer-Stroh, S. *et al.* Exploring the sequence determinants of amyloid structure using position-specific scoring matrices. *Nat. Methods* **7**, 237–242 (2010).

41. Tang, J. D., Mura, C. & Lampe, K. J. Stimuli-Responsive, Pentapeptide, Nanofiber Hydrogel for Tissue Engineering. *J. Am. Chem. Soc.* **141**, 4886–4899 (2019).

42. Do, T. D. *et al.* Distal amyloid β-protein fragments template amyloid assembly. *Protein Sci.* **27**, 1181–1190 (2018).

43. Chen, C. *et al.* Hydrogelation of the Short Self-Assembling Peptide I 3 QGK Regulated by Transglutaminase and Use for Rapid Hemostasis. *ACS Appl. Mater. Interfaces* **8**, 17833–17841 (2016).

44. Han, S. *et al.* Self-Assembly of Short Peptide Amphiphiles: The Cooperative Effect of Hydrophobic Interaction and Hydrogen Bonding. *Chem. - A Eur. J.* **17**, 13095–13102 (2011).

45. Reynolds, N. P. *et al.* Competition between crystal and fibril formation in molecular mutations of amyloidogenic peptides. *Nat. Commun.* **8**, 1–10 (2017).

46. Papanikolopoulou, K. *et al.* Amyloid Fibril Formation from Sequences of a Natural β-Structured Fibrous Protein, the Adenovirus Fiber. *J. Biol. Chem.* **280**, 2481–2490 (2005).

47. Morris, K. L. *et al.* The Structure of Cross-β Tapes and Tubes Formed by an Octapeptide, αSβ1. *Angew. Chem. Int. Ed.* **52**, 2279–2283 (2013).

48. Balbirnie, M., Grothe, R. & Eisenberg, D. S. An amyloid-forming peptide from the yeast prion Sup35 reveals a dehydrated β-sheet structure for amyloid. *Proc. Natl. Acad. Sci. U. S. A.* **98**, 2375–2380 (2001).

49. Beerten, J. *et al.* WALTZ-DB: a benchmark database of amyloidogenic hexapeptides. *Bioinformatics* **31**, 1698–1700 (2015).

50. Azriel, R. & Gazit, E. Analysis of the minimal amyloid-forming fragment of the islet amyloid polypeptide. An experimental support for the key role of the phenylalanine residue in amyloid formation. *J. Biol. Chem.* **276**, 34156–34161 (2001).

51. Chiti, F. *et al.* Kinetic partitioning of protein folding and aggregation. *Nat. Struct. Biol.* **9**, 137–143 (2002).

52. Kurnellas, M. P., Adams, C. M., Sobel, R. A., Steinman, L. & Rothbard, J. B. Amyloid fibrils composed of hexameric peptides attenuate neuroinflammation. *Sci. Transl. Med.* **5**, (2013).

53. Louros, N. *et al.* WALTZ-DB 2.0: an updated database containing structural information of experimentally determined amyloid-forming peptides. *Nucleic Acids Res.* **48**, D389–D393 (2020).

54. Luisi, D. L., Wu, W.-J. & Raleigh, D. P. Conformational analysis of a set of peptides corresponding to the entire primary sequence of the N-terminal domain of the ribosomal protein L9: evidence for stable native-like secondary structure in the unfolded state. *J. Mol. Biol.* **287**, 395–407 (1999).

55. Sieste, S. *et al.* Supramolecular Peptide Nanofibrils with Optimized Sequences and Molecular Structures for Efficient Retroviral Transduction. *Adv. Funct. Mater.* **31**, 2009382 (2021).
