# Supplementary material for: Data-mining unveils structure–property–activity correlation of viral infectivity enhancing self-assembling peptides
Source: Nat Commun. 2023 Aug 23;14:5121. doi: 10.1038/s41467-023-40663-6 (PMC10447463; doi:10.1038/s41467-023-40663-6)
Supplement: Supplementary file 3 — Description of Additional Supplementary Files [file 41467_2023_40663_MOESM3_ESM.pdf]

Description of Additional Supplementary Files:

Supplementary Data 1: Supplementary Data 1 summarizes the suppliers and catalog numbers of chemicals used in this report.

Supplementary Data 2: Supplementary Data 2 summarizes peptide properties from databases considered for the selection of literature known peptides.
