# Supplementary material for: Data-mining unveils structure–property–activity correlation of viral infectivity enhancing self-assembling peptides
Source: Nat Commun. 2023 Aug 23;14:5121. doi: 10.1038/s41467-023-40663-6 (PMC10447463; doi:10.1038/s41467-023-40663-6)
Supplement: Supplementary file 5 — Supplementary Data 2 [file 41467_2023_40663_MOESM5_ESM.pdf]

Berman, H. M. *et al.* The Protein Data Bank. *Nucleic Acids Research* vol. 28 235–242 (2000).

Sequence	Source hyperlink	fibrils	hydrophobicity	amino acid length	pI	charge	mw	
FDFSFDFS	https://pubs.acs.org/doi/10.1021/jacs.8b13363	+	0,6925	8	3,4919014	-2,05856022	1011,0552	
IDIDI	https://pubs.acs.org/doi/10.1021/jacs.8b13363	+	0,772	5	3,4919014	-2,05856022	587,67064	
XFTLDADF	1AFT		0,53875	8	3,4919014	-2,05856022	827,88894	
AADTWE	6C3T		0,286666667	6	3,55006433	-2,05837541	691,69524	
GDVIEV	3SGS	+	0,471666667	6	3,55006433	-2,05837541	630,69584	
GDVIEV	3SGS	+	0,471666667	6	3,55006433	-2,05837541	630,69584	
GDVIEV	3SGS	+	0,471666667	6	3,55006433	-2,05837541	630,69584	
IFAEDV	5TXJ		0,618333333	6	3,55006433	-2,05837541	692,76674	
DSVISLS	4NIN	+	0,547142857	7	3,74997248	-1,05887635	719,78984	
GFGGNDNFG	6J60		0,178888889	9	3,74997248	-1,05887635	883,87224	
GFGGNDNFG	5ZGD		0,178888889	9	3,74997248	-1,05887635	883,87224	
LIVAGD	https://pubs.acs.org/doi/10.1021/jacs.8b13363	+	0,71	6	3,74997248	-1,05887635	586,68594	
LVEALYL	3HYD	+	0,992857143	7	3,8499736	-1,06068283	819,99634	
LVEALYL	3HYD		0,992857143	7	3,8499736	-1,06068283	819,99634	
LYQLEN	2OMP	+	0,483333333	6	3,8499736	-1,06068283	778,86004	
TESYFVFSVGM	https://amypro.net/data/amypro.txt	+	0,704545455	11	3,8499736	-1,06068283	1266,4311	
VEALYL	2OMQ	+	0,875	6	3,8499736	-1,06068283	706,83694	
AEVVFT	4XFN	+	0,693333333	6	3,84997404	-1,05869154	664,75644	
LTIITLE	4RP6	+	0,982857143	7	3,84997404	-1,05869154	801,97854	
LTIITLE	4RP6		0,982857143	7	3,84997404	-1,05869154	801,97854	
TIITLE	4RP7	+	0,863333333	6	3,84997404	-1,05869154	688,81914	
TIITLE	4RP7	+	0,863333333	6	3,84997404	-1,05869154	688,81914	
DVPKSDQFVGLM	https://amypro.net/data/amypro.txt	+	0,424166667	12	4,10922403	-1,05895817	1335,5378	
RQEFEV	6N4P		0,083333333	6	4,25817255	-1,05819855	806,87364	
MIHFGND	3NVH	+	0,511428571	7	5,2922762	-0,94706058	832,92924	
MIHFGND	3NVH		0,511428571	7	5,2922762	-0,94706058	832,92924	

YYCYY	7C0N		1,076	5	5,91065589	-0,14074519	773,85804	
SYSGYS	6KJ2		0,3	6	6,08659065	-0,06317506	662,65374	
SYSGYS	6KJ1		0,3	6	6,08659065	-0,06317506	662,65374	
SYSGYS	6KJ4		0,3	6	6,08659065	-0,06317506	662,65374	
SYSGYS	6KJ3		0,3	6	6,08659065	-0,06317506	662,65374	
SYSGYS	5XSG		0,3	6	6,08659065	-0,06317506	662,65374	
SYSGYS	6BWZ		0,3	6	6,08659065	-0,06317506	662,65374	
SYSSYG	5XRR		0,3	6	6,08659065	-0,06317506	662,65374	
SYSSYGQS	6BXV		0,1925	8	6,08659065	-0,06317506	877,86264	
YTIAALLSPYS	https://www.rcsb.org/structure/2M5K	+	0,785454545	11	6,08659065	-0,06317506	1198,3812	
YTIAALLSPYS	1RVS	+	0,785454545	11	6,08659065	-0,06317506	1198,3812	
YTIAALLSPYS	1RVS		0,785454545	11	6,08659065	-0,06317506	1198,3812	
AYFIL	https://pubs.acs.org/doi/10.1021/jacs.8b13363	+	1,312	5	6,09322135	-0,06118377	625,76544	
GNNQQNY	https://www.rcsb.org/structure/2OMM	+	-0,18285714	7	6,09322135	-0,06118377	836,81594	
GYMLGS	3NHC	+	0,641666667	6	6,09322135	-0,06118377	626,72524	
GYMLGS	3NHC		0,641666667	6	6,09322135	-0,06118377	626,72524	
GYMLGS	3MD4		0,641666667	6	6,09322135	-0,06118377	626,72524	
GYVLGS	3NHD	+	0,64	6	6,09322135	-0,06118377	594,66524	
GYVLGS	3NHD		0,64	6	6,09322135	-0,06118377	594,66524	
GYVLGS	3MD5		0,64	6	6,09322135	-0,06118377	594,66524	
NNQQNY	https://www.rcsb.org/structure/5K2E	+	-0,21333333	6	6,09322135	-0,06118377	779,76404	
NVGSNTY	https://www.rcsb.org/structure/3FTK	+	0,171428571	7	6,09322135	-0,06118377	753,76664	
NVGSNTY	3FTK		0,171428571	7	6,09322135	-0,06118377	753,76664	
QYNNQNNFV	6AXZ		0,125555556	9	6,09322135	-0,06118377	1140,177	
QYNNQNNFV	6BTK		0,125555556	9	6,09322135	-0,06118377	1140,177	
STGGYG	6BZP		0,196666667	6	6,09322135	-0,06118377	540,53024	
SVQIVY	6ODG		0,823333333	6	6,09322135	-0,06118377	707,82474	
YTFGQ	6DIY	+	0,558	5	6,09322135	-0,06118377	614,65554	
YTIAAL	6C3S		0,89	6	6,09322135	-0,06118377	650,77274	
YVVFL	4ONK	+	1,378	5	6,09322135	-0,06118377	639,79244	
YVVFL	https://doi.org/10.2210/pdb4ONK/pdb		1,378	5	6,09322135	-0,06118377	639,79244	
YVVFL	4ONK		1,378	5	6,09322135	-0,06118377	639,79244	
YVVFV	4OLR	+	1,282	5	6,09322135	-0,06118377	625,76564	

YVVFV	4OLR		1,282	5	6,09322135	-0,06118377	625,76564	
NNQQ	https://www.rcsb.org/structure/2OLX	+	-0,41	4	6,09998233	-0,05919248	502,48424	
AALALL	7LUX		1,005	6	6,09998233	-0,05919248	570,72984	
AALALL	7LTU		1,005	6	6,09998233	-0,05919248	570,72984	
AGAAAA	6PQ5		0,258333333	6	6,09998233	-0,05919248	430,46114	
AIIGLM	2Y3J	+	1,14	6	6,09998233	-0,05919248	616,81674	
AILSST	3FOD	+	0,665	6	6,09998233	-0,05919248	590,67434	
ASLTVS	6DJ0		0,568333333	6	6,09998233	-0,05919248	576,64754	
AVVTGVTAV	4RIK		0,668888889	9	6,09998233	-0,05919248	815,96534	
GAIIGL	3PZZ	+	0,935	6	6,09998233	-0,05919248	542,67604	
GAIIGL	3PZZ		0,935	6	6,09998233	-0,05919248	542,67604	
GAVVGG	6PQA		0,458333333	6	6,09998233	-0,05919248	458,51494	
GAVVTGVTAVA	4RIL		0,575454545	11	6,09998233	-0,05919248	944,09604	
GGVLVN	3PPD	+	0,59	6	6,09998233	-0,05919248	557,64744	
GGVVIA	2ONV	+	0,758333333	6	6,09998233	-0,05919248	514,62244	
GNLVS	4QXX	+	0,456	5	6,09998233	-0,05919248	488,54114	
GSQASS	7LV2		-0,005	6	6,09998233	-0,05919248	535,51124	
GSTSTA	6M7M		0,125	6	6,09998233	-0,05919248	522,51254	
GSTSTA	6EEX		0,125	6	6,09998233	-0,05919248	522,51254	
GSTSTA	6M9J		0,125	6	6,09998233	-0,05919248	522,51254	
GSTSTA	6M9I		0,125	6	6,09998233	-0,05919248	522,51254	
GVIGIAQ	4NIP	+	0,701428571	7	6,09998233	-0,05919248	656,77994	
GVTGIAQ	4NIO	+	0,481428571	7	6,09998233	-0,05919248	644,72564	
IFQINS	4R0P	+	0,755	6	6,09998233	-0,05919248	720,82334	
MVGGVV	https://www.rcsb.org/structure/2OKZ	+	0,815	6	6,09998233	-0,05919248	560,70944	
MVGGVVIA	https://www.rcsb.org/structure/2Y3K	+	0,875	8	6,09998233	-0,05919248	744,94764	
NFGAIL	https://pubs.acs.org/doi/10.1021/jacs.8b13363	+	0,833333333	6	6,09998233	-0,05919248	633,74514	
NFGAILS	2KIB	+	0,708571429	7	6,09998233	-0,05919248	720,82334	
NNFGAIL	3DGJ	+	0,628571429	7	6,09998233	-0,05919248	747,84894	
NNQNTF	3FVA	+	0,005	6	6,09998233	-0,05919248	736,73904	
NNQNTF	3FVA	+	0,005	6	6,09998233	-0,05919248	736,73904	
SAFSAFA	5VSG		0,632857143	7	6,09998233	-0,05919248	699,76124	
SGNNFGAILSS	5KNZ	+	0,389090909	11	6,09998233	-0,05919248	1066,1354	

SGNNFGAILSS	5KNZ		0,389090909	11	6,09998233	-0,05919248	1066,1354	
SNNFGAILSS	1KUW	+	0,428	10	6,09998233	-0,05919248	1009,0835	
SNNFGAILSS	1KUW		0,428	10	6,09998233	-0,05919248	1009,0835	
SNQNNF	2OL9	+	-0,045	6	6,09998233	-0,05919248	722,71214	
SSTNVG	https://www.rcsb.org/structure/3FTR	+	0,133333333	6	6,09998233	-0,05919248	563,56504	
SSTNVG	3FTR	+	0,133333333	6	6,09998233	-0,05919248	563,56504	
SSTNVG	3DG1	+	0,133333333	6	6,09998233	-0,05919248	563,56504	
SSTSAA	2ONW	+	0,126666667	6	6,09998233	-0,05919248	522,51254	
TAVVTN	4XFO	+	0,445	6	6,09998233	-0,05919248	603,67324	
TGVTAVA	4R0U	+	0,511428571	7	6,09998233	-0,05919248	617,70014	
TGVTAVA	4R0U		0,511428571	7	6,09998233	-0,05919248	617,70014	
TTTTTT	6WK7		0,26	6	6,09998233	-0,05919248	624,64584	
UGGGGU	4RKV		0	6	6,09998233	-0,05919248	546,30044	
UGGGGU	4RJ1		0	6	6,09998233	-0,05919248	546,30044	
VVTGVTA	4R0W	+	0,641428571	7	6,09998233	-0,05919248	645,75394	
VVTGVTA	4R0W		0,641428571	7	6,09998233	-0,05919248	645,75394	
KDWSFY	4E0K	+	0,533333333	6	6,33056908	-0,06126559	844,92194	
QADPNKFYGLM	https://amypro.net/data/amypro.txt	+	0,375454545	11	6,33056908	-0,06126559	1283,4644	
DFNKF	https://pubs.acs.org/doi/10.1021/jacs.8b13363	+	0,244	5	6,3373688	-0,0592743	669,73494	
LSFSKD	3LOZ	+	0,275	6	6,3373688	-0,0592743	695,77034	
IYKVEI	6C3F		0,691666667	6	6,40198668	-0,06108078	763,93224	
KLVFFAE	https://pubs.acs.org/doi/10.1021/jacs.8b13363	+	0,74	7	6,40878379	-0,05908949	853,02884	
KVKVLGDVIEV	3SGO		0,453636364	11	6,49297173	-0,05917131	1198,4686	
DRVYIHP	2JP8		0,435714286	7	7,54294793	0,050940186	899,01714	
DRVYIHPF	1N9V		0,605	8	7,54294793	0,050940186	1046,1937	
FLVHSSNNFGA	5KO0	+	0,514545455	11	7,55032539	0,052623291	1192,2962	
GVVHGVTTVA	4ZNN		0,584	10	7,55032539	0,052623291	939,07954	
HGGGWGQP	1OEH	+	0,36	8	7,55032539	0,052623291	794,82454	
HGGGWGQP	1OEH		0,36	8	7,55032539	0,052623291	794,82454	
HSSNNF	3FPO	+	0,106666667	6	7,55032539	0,052623291	704,69694	
HSSNNF	3FPO	+	0,106666667	6	7,55032539	0,052623291	704,69694	
IIHFGS	3NVF	+	0,913333333	6	7,55032539	0,052623291	672,78184	
IIHFGS	3NVF	+	0,913333333	6	7,55032539	0,052623291	672,78184	

MIHFGN	3NVG	+	0,725	6	7,55032539	0,052623291	717,84064	
MIHFGN	3NVG	+	0,725	6	7,55032539	0,052623291	717,84064	
MMHFGN	3NVE	+	0,63	6	7,55032539	0,052623291	735,87384	
MMHFGN	3NVE		0,63	6	7,55032539	0,052623291	735,87384	
NFLVHS	3FR1	+	0,7	6	7,55032539	0,052623291	715,80694	
NFLVHS	3FR1	+	0,7	6	7,55032539	0,052623291	715,80694	
NFLVHSS	3FTH	+	0,594285714	7	7,55032539	0,052623291	802,88514	
VAVHVF	6C88	+	0,981666667	6	7,55032539	0,052623291	670,80954	
DRVYIHPFHL	1N9U		0,667	10	7,70633267	0,162755956	1296,4942	
CGGIRGERA	1CS9	-	0,11	9	8,54983271	0,867705015	918,03844	
CGGIRGERG	1CT6	-	0,075555556	9	8,54983271	0,867705015	904,01154	
KYFIL	https://pubs.acs.org/doi/10.1021/jacs.8b13363	+	1,052	5	9,2982946	0,938418283	682,86074	
VQIVYK	4NP8		0,665	6	9,2982946	0,938418283	748,92064	
VQIVYK	5K7N		0,665	6	9,2982946	0,938418283	748,92064	
VQIVYK	2ON9		0,665	6	9,2982946	0,938418283	748,92064	
XSYVKA	1BFZ		0,243333333	6	9,2982946	0,938418283	566,65494	
KFFEAAAKKFFE	2BFI	+	0,32	12	9,44155743	0,940615547	1462,7113	
GQTVTK	7LUZ		0,088333333	6	9,70001568	0,940409572	632,71474	
GSNKGAIIGLM	1QCM	+	0,473636364	11	9,70001568	0,940409572	1060,2766	
GSNKGAIIGLM	1QWP	+	0,473636364	11	9,70001568	0,940409572	1060,2766	
GSNKGAIIGLM	1QYT	+	0,473636364	11	9,70001568	0,940409572	1060,2766	
GSNKGAIIGLM	1QXC	+	0,473636364	11	9,70001568	0,940409572	1060,2766	
GSNKGAIIGLM	1QWP	+	0,473636364	11	9,70001568	0,940409572	1060,2766	
GSNKGAIIGLM	1QXC	+	0,473636364	11	9,70001568	0,940409572	1060,2766	
GSNKGAIIGLM	1QYT	+	0,473636364	11	9,70001568	0,940409572	1060,2766	
GSNKGAIIGLM	1QCM	+	0,473636364	11	9,70001568	0,940409572	1060,2766	
KALGIS	6C3G	+	0,463333333	6	9,70001568	0,940409572	587,71704	
NKGAIF	5TXD	+	0,385	6	9,70001568	0,940409572	648,75984	
NKGAII	3Q2X	+	0,386666667	6	9,70001568	0,940409572	614,74264	
VGSNKGAIIGL	5VOS	+	0,472727273	11	9,70001568	0,940409572	1028,2166	
VQIINK	5V5C	+	0,501666667	6	9,70001568	0,940409572	713,87524	
KVQIINKKLD	5V5B	+	0,196	10	10,5069011	1,939929803	1198,4714	
IIKIIK	https://doi.org/10.1038/s41467-018-05490-0	+	0,87	6	10,8053935	1,940011624	727,00104	

IIKVIK	https://doi.org/10.1038/s41467-018-05490-0	+	0,773333333	6	10,8053935	1,940011624	712,97424	
K2(QL)6K2	https://doi.org/10.1038/s41467-018-05490-0	+	-0,05555556	9	10,8053935	1,940011624	515,65354	
KVQIINKKL	6NK4	-	0,303333333	9	11,1030807	2,939613675	1083,3828	
RPKPQQFFGLM	https://amypro.net/#/browse	-	0,500909091	11	11,6517687	1,940401629	1348,6287	
GFIVKRFKILV	6RRO	-	0,757272727	11	11,8230627	2,94000368	1319,6994	
VRRFDLLKRILK	2N5R	-	0,344166667	12	12,2436696	3,940303922	1556,9613	
RGPGRAFVTI	6B9K	-	0,408	10	12,5000712	1,940791635	1073,2632	
FRIMRILRVLKL	6RRL	-	0,743333333	12	12,8074301	3,940385743	1558,0506	
DFNKF	https://www.jbc.org/article/S0021-9258(18)36785-1/pdf	+	0,244	5	6,3373688	-0,0592743	669,73494	
IIIGGK	https://doi.org/10.1038/s41467-018-07583-2	+	0,735	6	9,70001568	0,940409572	599,77134	
AIIGLM	https://www.rcsb.org/structure/2Y3J		1,14	6	6,09998233	-0,05919248	616,81674	
YVVFV	https://www.rcsb.org/structure/4OLR		1,282	5	6,09322135	-0,06118377	625,76564	
YVVFV	https://www.rcsb.org/structure/4ONK		1,282	5	6,09322135	-0,06118377	625,76564	
AYFIL	https://doi.org/10.1021/jacs.8b13363		1,312	5	6,09322135	-0,06118377	625,76544	
YVVFL	https://doi.org/10.2210/pdb4ONK/pdb		1,378	5	6,09322135	-0,06118377	639,79244	
IIKVIK	https://www.rcsb.org/structure/6FG4		0,773333333	6	10,8053935	1,940011624	712,97424	
IIKIIK	https://www.rcsb.org/structure/6FGR		0,87	6	10,8053935	1,940011624	727,00104	
IIIQGK	https://doi.org/10.1021/acsami.6b04939	+	0,69		9,7	0,94		

Fernandez-Escamilla, A.-M., Rousseau, F., Schymkowitz, J. & Serrano, L. Prediction of sequence-dependent and mutational effects on the aggregation of peptides and proteins. *Nat. Biotechnol.* **22**, 1302–1306 (2004).								
sequence	Source hyperlink	CD FTIR	length	pI	charge	hydrophobicity (fauchere)	derived from	original function or misfunction
PGGGKVQIVYKPV	https://www.nature.com/articles/nbt1012	+	13	10,2476	2	0,4353846	K19	t-protein
NLKHQPGGGKVQIVYKPVDLSKVTSKCGSLGNIHHKPGGGQVE	https://www.nature.com/articles/nbt1012	+	43	10,294	4	0,2506977	K19Gluc41	t-protein
NLKHQPGGGKVQIVYKEVD	https://www.nature.com/articles/nbt1012	+	19	9,22264	1	0,1868421	K19Gluc782	t-protein
GKVQIVYK	https://www.nature.com/articles/nbt1012	+	8	10,2476	2	0,375	PHF8	t-protein
VQIVYK	https://www.nature.com/articles/nbt1012	+	6	9,2983	0,9384183	0,665	PHF6	t-protein
DAEFRHDSGYEVHHQKLVFFAEDVGSNKGAIIGLMVGGVV	https://www.nature.com/articles/nbt1012	+	40	5,42862	-3	0,377	Whole	Amyloid beta Aβ peptide (1-40)
VPHQKLVFFAEDVGS	https://www.nature.com/articles/nbt1012	+	15	5,45428	-0,9469576	0,496	HABP1	Amyloid beta Aβ peptide (1-40)
VHPQKLVFFAEDVGS	https://www.nature.com/articles/nbt1012	+	15	5,45428	-0,9469576	0,496	HABP2	Amyloid beta Aβ peptide (1-40)
VHHPKLVFFAEDVGS	https://www.nature.com/articles/nbt1012	+	15	6,49983	-0,8351418	0,5193333	HABP3	Amyloid beta Aβ peptide (1-40)

VHHQPLVFFAEDVGS	https://www.nature.com/articles/nbt1012	+	15	5,2961	-2	0,5706667	HABP4	Amyloid beta Aβ peptide (1-40)
KKLVFFPED	https://www.nature.com/articles/nbt1012	+	9	6,49297	-0,05917131	0,4255556	HABP10	Amyloid beta Aβ peptide (1-40)
VHHQEKLVFFAEPVGS	https://www.nature.com/articles/nbt1012	+	16	6,50106	-0,834957	0,48125	HABP12	Amyloid beta Aβ peptide (1-40)
VHHQEKLVFFAEDPGS	https://www.nature.com/articles/nbt1012	+	16	5,3903	-2	0,356875	HABP13	Amyloid beta Aβ peptide (1-40)
VHHQEKLVFFAEDVPS	https://www.nature.com/articles/nbt1012	+	16	5,3903	-2	0,433125	HABP14	Amyloid beta Aβ peptide (1-40)
KKLVFFAED	https://www.nature.com/articles/nbt1012	+	9	6,49297	-0,05917131	0,38	HABP15	Amyloid beta Aβ peptide (1-40)
VHHQKLVFFAEDVGS	https://www.nature.com/articles/nbt1012	+	15	6,49983	-0,8351418	0,4566667	HABP16	Amyloid beta Aβ peptide (1-40)
HHQKLVFFAED	https://www.nature.com/articles/nbt1012	+	11	6,49983	-0,8351418	0,4045455	AB4	Amyloid beta Aβ peptide (1-40)

VHHQKLVFFAEDV	https://www.nature.com/articles/nbt1012	+		13	6,49983	-0,8351418	0,53	AB5	Amyloid beta Aβ peptide (1-40)
EVHHQKLVFFAEDVG	https://www.nature.com/articles/nbt1012	+		15	5,3903	-2	0,4166667	AB6	Amyloid beta Aβ peptide (1-40)
YEVHHQKLVFFAEDVGS	https://www.nature.com/articles/nbt1012	+		17	5,39027	-2	0,4217647	AB7	Amyloid beta Aβ peptide (1-40)
GYEVHHQKLVFFAEDVGSN	https://www.nature.com/articles/nbt1012	+		19	5,39027	-2	0,3457895	AB8	Amyloid beta Aβ peptide (1-40)
SGYEVHHQKLVFFAEDVGSNK	https://www.nature.com/articles/nbt1012	+		21	6,50288	-0,8370301	0,2638095	AB9	Amyloid beta Aβ peptide (1-40)
DSGYEVHHQKLVFFAEDVGSNKG	https://www.nature.com/articles/nbt1012	+		23	5,43536	-2	0,2073913	AB10	Amyloid beta Aβ peptide (1-40)
HDSGYEVHHQKLVFFAEDVGSNKGA	https://www.nature.com/articles/nbt1012	+		25	6,21389	-2	0,2084	AB11	Amyloid beta Aβ peptide (1-40)
EQVTNVGGAVVTGVTAVA	https://www.nature.com/articles/nbt1012	+		18	3,84997	-1	0,4205556	NAC1-18	Alpha synuclein
TVNGVGEVTATAVQGVAV	https://www.nature.com/articles/nbt1012	+		18	3,84997	-1	0,4205556	NAC1-18s	Alpha synuclein

VTNVGGAVVTGVTAVA	https://www.nature.com/articles/nbt1012	+		16	6,09998	-0,05919248	0,526875	NAC3-18	Alpha synuclein
EQVTNVGGAVVTG	https://www.nature.com/articles/nbt1012	+		13	3,84997	-1	0,3269231	NAC1-13	Alpha synuclein
VGGAVVTGV	https://www.nature.com/articles/nbt1012	+		9	6,09998	-0,05919248	0,6055556	NAC6-14	Alpha synuclein
GVVGWVKNTSKGTVTGQVQG	https://www.nature.com/articles/nbt1012	+		20	10,8054	2	0,3035	34-53	Acyl phosphatase
ISKLEYSNFSVRY	https://www.nature.com/articles/nbt1012	+		13	9,1451	0,93692	0,39	86-98	Acyl phosphatase
DWSFYLLYYTEFT	https://www.nature.com/articles/nbt1012	+		13	3,55006	-2	0,86	E	β2-microglobulin
DWSFYLLYYTEFTPTGKDEYA	https://www.nature.com/articles/nbt1012	+		21	3,7379	-3	0,5252381	E1	β2-microglobulin
TKRPRFLYEIAMALNSD	https://www.nature.com/articles/nbt1012	+		17	9,29641	0,9392195	0,3364706	4Cro	434 Cro repressor
VLSEGEWQLVLHVWAKVEA	https://www.nature.com/articles/nbt1012	+		19	4,47386	-2	0,6347368	A-Helix	Sperm whale myoglobin
EGEWQLVLHVWAKVEADVAGHGQDILIRLFK	https://www.nature.com/articles/nbt1012	+		31	5,49185	-2	0,5119355	AB-Domain	Sperm whale myoglobin
DVAGHGQDILIRLFKS	https://www.nature.com/articles/nbt1012	+		16	7,54998	0,05284965	0,415625	B-Helix	Sperm whale myoglobin

YEQLDEEHKKIFKG IFDCIRD	https://www.nature.com/articles/nbt1012	+		21	4,73411	-2	0,232381	A_helix	Myoheme rithrin
SAPNLATLVKVTTN HFTHEEAMMD	https://www.nature.com/articles/nbt1012	+		24	5,3903	-2	0,365	B_helix	Myoheme rithrin
EVVPHKKMHKDFL EKIGGL	https://www.nature.com/articles/nbt1012	+		19	9,3775	1	0,2963158	C_Helix	Myoheme rithrin
LEVLLGSGDGSLVF V	https://www.nature.com/articles/nbt1012	+		15	3,55006	-2	0,7173333	Pc-2	French bean plastocya nin
GEKIVFKNNAGFP HNVVFDE	https://www.nature.com/articles/nbt1012	+		20	5,54939	-0,9468546	0,308	Pc-6a	French bean plastocya nin
IPAGVDAVKISM	https://www.nature.com/articles/nbt1012	+		12	6,33737	-0,0592743	0,5675	Pc-10	French bean plastocya nin
MPEEELLNAPGET YVVTL	https://www.nature.com/articles/nbt1012	+		18	3,37081	-4	0,4911111	Pc-12	French bean plastocya nin
GETYVVTL	https://www.nature.com/articles/nbt1012	+		8	3,84997	-1	0,6225	Pc-14	French bean plastocya nin
GTYSFYT	https://www.nature.com/articles/nbt1012	+		7	6,08659	-0,06317506	0,5985714	Pc-16	French bean plastocya nin

GTVSFVTSPHQGA GMVGKVTVN	https://www.nature.com/articles/nbt1012	+	22	9,702	1	0,4168182	Pc-19	French bean plastocyanin
LSQTFVYGGSRAK RNN	https://www.nature.com/articles/nbt1012	+	16	11,5305	3	0,108125	P29-44	Bovine pancreatic trypsin inhibitor (BPTI)
MKVIFLKDVKG	https://www.nature.com/articles/nbt1012	+	11	10,5069	2	0,4745455	Beta_1	N-terminal domain of ribosomal protein L9
GYANNFLFKQG	https://www.nature.com/articles/nbt1012	+	11	9,2983	0,9384183	0,3763636	Alpha_1	N-terminal domain of ribosomal protein L9
QISFADYNLLDLLRI HQVLN	https://www.nature.com/articles/nbt1012	+	20	5,40807	-0,9487437	0,614	Alpha_6	Glutathione S transeferase P domain II (Glutex)
DILTLLNSTNKDW WKVEVND	https://www.nature.com/articles/nbt1012	+	20	4,06268	-2	0,3795	M_8	Spectrin SH3
DWWKVEVNDRQ GFVPA	https://www.nature.com/articles/nbt1012	+	16	4,37042	-1	0,37375	M_6	Spectrin SH3

DILTLLNSTNKDWWKVEVNDRQGFVPA	https://www.nature.com/articles/nbt1012	+	27	4,49931	-1	0,3851852	M_68	Spectrin SH3
FVNVQAVKVFLESQGIAY	https://www.nature.com/articles/nbt1012	+	18	6,40199	-0,06108078	0,6016667	H2_WT	Spectrin SH3
FVNVEAVKAFLEAHGIAY	https://www.nature.com/articles/nbt1012	+	18	5,49303	-0,9487641	0,5666667	H2_Mt	Spectrin SH3
STNVKTAFEMVILDIYNNV	https://www.nature.com/articles/nbt1012	+	19	4,18439	-1	0,5015789	Ara5	Spectrin SH3
TESKEKITQYIYHVLNGEIL	https://www.nature.com/articles/nbt1012	+	20	5,57604	-0,9506524	0,3915	ComA5	Spectrin SH3
AKKENIIAAAQAGASGY	https://www.nature.com/articles/nbt1012	+	17	9,25605	0,9385213	0,1729412	Che_Y4	Spectrin SH3
PFTAATLEEKLNKIFEKLGMY	https://www.nature.com/articles/nbt1012	+	21	6,60106	-0,06087481	0,4304762	Che_Y5	Spectrin SH3
GVGKSALTIQLIQNHFVY	https://www.nature.com/articles/nbt1012	+	18	9,30071	1	0,6011111	P21A	Flavodoxin
RQGVEDAFYTLVREIRQHK	https://www.nature.com/articles/nbt1012	+	19	9,29789	1	0,1515789	P21E	Flavodoxin
VTIKANLIFANGFTQTAEFKG	https://www.nature.com/articles/nbt1012	+	21	9,53732	0,9405126	0,4552381	PL_B1_95-114_pH_4.1	PL B1 protein
KGTFEKATSEAYAYADTLKKDNGEY	https://www.nature.com/articles/nbt1012	+	25	4,67258	-1	0,0132	PL_B1_114-138_pH_2.4	PL B1 protein
GEYTVDVADKGYTLNIKFAGD	https://www.nature.com/articles/nbt1012	+	21	4,06271	-2	0,2504762	PL_B1_136-155D_pH_6.1	PL B1 protein
GEWTYDDATKTFTVTE	https://www.nature.com/articles/nbt1012	+	16	3,7379	-3	0,25125	ProteinG41-56	PL B1 protein

VHDCVNITIK	https://www.nature.com/articles/nbt1012	+	10	7,35902	-0,02104609	0,561	86-95	human prion protein
SMVLFSSPPV	https://www.nature.com/articles/nbt1012	+	10	6,09998	-0,05919248	0,848	141-150	human prion protein
SSPPVILLIS	https://www.nature.com/articles/nbt1012	+	10	6,09998	-0,05919248	0,954	146-155	human prion protein
ILLISFLIFL	https://www.nature.com/articles/nbt1012	+	10	6,09998	-0,05919248	1,574	151-160	human prion protein
FLIFLIVG	https://www.nature.com/articles/nbt1012	+	8	6,09998	-0,05919248	1,475	156-163	human prion protein
RCELARTLKR	https://www.nature.com/articles/nbt1012	+	10	11,3797	3	0,085	41760	human lysozyme
LANWMCLAKW	https://www.nature.com/articles/nbt1012	+	10	8,54518	0,866822	0,97	25-34	human lysozyme
DLSFSKDWSF	https://www.nature.com/articles/nbt1012	+	10	4,10922	-1	0,488	54-63	β2-microglobulin
KDWSFYLLYY	https://www.nature.com/articles/nbt1012	+	10	6,31764	-0,06524817	0,852	59-68	β2-microglobulin
YLLYYTEFTP	https://www.nature.com/articles/nbt1012	+	10	3,84997	-1	0,867	64-73	β2-microglobulin
TEFTPTEKDE	https://www.nature.com/articles/nbt1012	+	10	3,77825	-3	-0,039	69-78	β2-microglobulin
PGGGKVYKPV	https://www.nature.com/articles/nbt1012	-	10	10,2476	2	0,286	K19d	t-protein

PGGGKNAEVYKPV	https://www.nature.com/articles/nbt1012	-	13	9,25605	0,9385213	0,1484615	Mut1	t-protein
PGGGKVQIVEKPV	https://www.nature.com/articles/nbt1012	-	13	9,53732	0,9405126	0,3123077	Mut2	t-protein
QTAPVPMPDLKNVKSKIGSTENLKHQPGGGKVQIVY	https://www.nature.com/articles/nbt1012	-	36	10,3741	3	0,2669444	K19Chym	t-protein
KPVDLSKVTSKCGSLGNIHHKPGGGQVEVKSEKLDF	https://www.nature.com/articles/nbt1012	-	36	9,42108	2	0,2036111	K19Chym1	t-protein
KDRVQSKIGSLDNITHVPGGGN	https://www.nature.com/articles/nbt1012	-	22	9,53693	1	0,1281818	K19Chym2	t-protein
QTAPVPMPDLKNVKSKIGSTE	https://www.nature.com/articles/nbt1012	-	21	9,44155	0,9404307	0,232381	K19Gluc4	t-protein
VKSE	https://www.nature.com/articles/nbt1012	-	4	6,40878	-0,05908949	-0,1125	K19Gluc42	t-protein
KLDFKDRVQSKIGSLDNITHVPGGGN	https://www.nature.com/articles/nbt1012	-	26	9,44206	1	0,175	K19Gluc43	t-protein
QTAPVPMPD	https://www.nature.com/articles/nbt1012	-	9	3,74997	-1	0,4655556	K19Gluc78	t-protein
LKNVKSKIGSTE	https://www.nature.com/articles/nbt1012	-	12	10,5069	2	0,0575	K19Gluc781	t-protein
LSKVTSKCGSLGNIHHKPGGGQVE	https://www.nature.com/articles/nbt1012	-	24	9,58374	2	0,2445833	K19Gluc783	t-protein
VKSEKLDFKDRVQSKIGSLDNITHVPGGGN	https://www.nature.com/articles/nbt1012	-	30	9,37412	1	0,1366667	K19Gluc784	t-protein
VDLSKVTSK	https://www.nature.com/articles/nbt1012	-	9	9,5373	0,9403278	0,1744444	V313-K321	t-protein
VTSKCGSLGNIHHKPGGG	https://www.nature.com/articles/nbt1012	-	18	9,67846	2	0,2688889	V318-G335	t-protein
GQVEVSKE	https://www.nature.com/articles/nbt1012	-	8	4,25814	-1	-0,01125	V335-E342	t-protein

KKPVFFAED	https://www.nature.com/articles/nbt1012	-		9	6,49297	-0,05917131	0,2711111	HABP5	Amyloid beta Aβ peptide (1-40)
KKLPFFAED	https://www.nature.com/articles/nbt1012	-		9	6,49297	-0,05917131	0,3244444	HABP6	Amyloid beta Aβ peptide (1-40)
KKLVPFAED	https://www.nature.com/articles/nbt1012	-		9	6,49297	-0,05917131	0,2611111	HABP7	Amyloid beta Aβ peptide (1-40)
VHHQKLVPFAEDV GS	https://www.nature.com/articles/nbt1012	-		15	6,49983	-0,8351418	0,3853333	HABP8	Amyloid beta Aβ peptide (1-40)
KKLVFPAED	https://www.nature.com/articles/nbt1012	-		9	6,49297	-0,05917131	0,2611111	HABP9	Amyloid beta Aβ peptide (1-40)
VHHQEKLVFFAPD VGS	https://www.nature.com/articles/nbt1012	-		16	6,49983	-0,8351418	0,473125	HABP11	Amyloid beta Aβ peptide (1-40)
KLVFF	https://www.nature.com/articles/nbt1012	-		5	9,70002	0,9404096	1,102	AB1	Amyloid beta Aβ peptide (1-40)
QKLVFFA	https://www.nature.com/articles/nbt1012	-		7	9,70002	0,9404096	0,8	AB2	Amyloid beta Aβ peptide (1-40)

HQKLVFFAE	https://www.nature.com/articles/nbt1012	-		9	7,54974	0,05272628	0,5655556	AB3	Amyloid beta Aβ peptide (1-40)
STAQSLKSVDYEVFGRV	https://www.nature.com/articles/nbt1012	-		17	6,48753	-0,0607726	0,29	42736	Acyl phosphatase
QGVSFRMYTEDEARKI	https://www.nature.com/articles/nbt1012	-		16	6,57794	-0,0602796	0,140625	18-33	Acyl phosphatase
PEDKVNSMKSWLSKV	https://www.nature.com/articles/nbt1012	-		15	9,44155	0,9404307	0,216	54-68	Acyl phosphatase
GSPSSRIDRTNFSNEKT	https://www.nature.com/articles/nbt1012	-		17	9,69014	0,9412107	-0,05588235	69-85	Acyl phosphatase
IQRTPKIQVYSRHPAE	https://www.nature.com/articles/nbt1012	-		16	10,4466	2	0,236875	A	β2-microglobulin
NGKSNFLNCYVSG	https://www.nature.com/articles/nbt1012	-		13	8,52295	0,8648307	0,3338462	B	β2-microglobulin
FHPSDIEVDLLK	https://www.nature.com/articles/nbt1012	-		12	4,36609	-2	0,4875	C	β2-microglobulin
NGERIEKVEHSDLSFSKD	https://www.nature.com/articles/nbt1012	-		18	4,66497	-2	-0,02944444	D	β2-microglobulin
PTGKDEYACRVNHVT	https://www.nature.com/articles/nbt1012	-		15	7,35655	-0,02254438	0,174	F	β2-microglobulin

LSQPKIVKWDRDM	https://www.nature.com/articles/nbt1012	-	13	9,53486	0,9406359	0,3176923	G	β2-microglobulin
MQTLSERLKKRRIALKY	https://www.nature.com/articles/nbt1012	-	17	11,6681	5	0,1623529	1Cro	434 Cro repressor
YKMTQTELATKAGVK	https://www.nature.com/articles/nbt1012	-	15	10,1196	2	0,1786667	2Cro	434 Cro repressor
YKQQSIQLIEAGVTKR	https://www.nature.com/articles/nbt1012	-	16	10,2436	2	0,2325	3Cro	434 Cro repressor
AMALNCDPVWLQYGTKRGKA	https://www.nature.com/articles/nbt1012	-	20	9,40498	2	0,3965	5Cro	434 Cro repressor
KSHPET	https://www.nature.com/articles/nbt1012	-	6	7,54974	0,05272628	-0,09333333	BC-Turn	Sperm whale myoglobin
HPETLEKFDRFKHLK	https://www.nature.com/articles/nbt1012	-	15	9,4442	1	0,146	CCD-Domai	Sperm whale myoglobin
TEAEMKA	https://www.nature.com/articles/nbt1012	-	7	4,25814	-1	-0,02285714	D-Helix	Sperm whale myoglobin
SEDLKKHGVTVLTALGAILK	https://www.nature.com/articles/nbt1012	-	20	9,44369	1	0,3945	E-Helix	Sperm whale myoglobin
KKGHHEAE	https://www.nature.com/articles/nbt1012	-	8	7,71199	0,164645	-0,33625	EF-Turn	Sperm whale myoglobin

ELKPLAQSHA	https://www.nature.com/articles/nbt1012	-		10	7,54974	0,05272628	0,298	F-helix	Sperm whale myoglobin
ATKHKIP	https://www.nature.com/articles/nbt1012	-		7	10,8054	2	0,1771429	FG-Turn	Sperm whale myoglobin
GWEIPEPYVWDESFRVFY	https://www.nature.com/articles/nbt1012	-		18	3,77825	-3	0,6633333	N-terminal	Myohemerithrin
GTDFKYKGKL	https://www.nature.com/articles/nbt1012	-		10	10,1196	2	0,097	C-terminal	Myohemerithrin
YEQLDEEHKKIFKGIFDCIRD	https://www.nature.com/articles/nbt1012	-		21	4,73411	-2	0,232381	A_helix	Myohemerithrin
RDNSA	https://www.nature.com/articles/nbt1012	-		5	6,33873	-0,05888429	-0,422	AB_loop	Myohemerithrin
DAAKYSEV	https://www.nature.com/articles/nbt1012	-		8	4,18439	-1	0,045	BC_loop	Myohemerithrin
GLSAPVD	https://www.nature.com/articles/nbt1012	-		7	3,74997	-1	0,4485714	CD_loop	Myohemerithrin
AKNVDYCKEWLVNHIK	https://www.nature.com/articles/nbt1012	-		16	8,52211	0,9766677	0,346875	D_helix	Myohemerithrin
AKNVDYCKEWLVNHIK	https://www.nature.com/articles/nbt1012	-		16	8,52211	0,9766677	0,346875	D_helix	Myohemerithrin
LEVLLGSG	https://www.nature.com/articles/nbt1012	-		8	3,84997	-1	0,705	Pc-1	French bean plastocyanin
SGDGSL	https://www.nature.com/articles/nbt1012	-		6	3,74997	-1	0,1416667	Pc-2a	French bean plastocyanin

SLVFVPSEFS	https://www.nature.com/articles/nbt1012	-	10	3,84997	-1	0,768	Pc-3	French bean plastocyanin
SEFSV	https://www.nature.com/articles/nbt1012	-	5	3,84997	-1	0,458	Pc-4	French bean plastocyanin
SEFSVPSGEK	https://www.nature.com/articles/nbt1012	-	10	4,25814	-1	0,134	Pc-5	French bean plastocyanin
KIVFKNNA	https://www.nature.com/articles/nbt1012	-	8	10,8054	2	0,2425	Pc-6	French bean plastocyanin
KIVFKNNAGFPH	https://www.nature.com/articles/nbt1012	-	12	10,8054	2	0,3816667	Pc-7	French bean plastocyanin
KNNAGFPHNV	https://www.nature.com/articles/nbt1012	-	10	9,702	1	0,138	Pc-8	French bean plastocyanin
PHNVVFDEDDEIP	https://www.nature.com/articles/nbt1012	-	13	3,60564	-5	0,2623077	Pc-9	French bean plastocyanin
EIPAGV	https://www.nature.com/articles/nbt1012	-	6	3,84997	-1	0,5683333	Pc-10a	French bean plastocyanin

DAVKIS	https://www.nature.com/articles/nbt1012	-		6	6,33737	-0,0592743	0,255	Pc-10b	French bean plastocyanin
MPEEELL	https://www.nature.com/articles/nbt1012	-		7	3,47281	-3	0,49	Pc-11	French bean plastocyanin
ELLNAPGETY	https://www.nature.com/articles/nbt1012	-		10	3,6137	-2	0,377	Pc-13	French bean plastocyanin
NAPGETY	https://www.nature.com/articles/nbt1012	-		7	3,84997	-1	0,1442857	Pc-13a	French bean plastocyanin
APGET	https://www.nature.com/articles/nbt1012	-		5	3,84997	-1	0,13	Pc-13b	French bean plastocyanin
ETYVVT	https://www.nature.com/articles/nbt1012	-		6	3,84997	-1	0,5466667	Pc-14a	French bean plastocyanin
VTLDTKGTY	https://www.nature.com/articles/nbt1012	-		9	6,33057	-0,06126559	0,3222222	Pc-15	French bean plastocyanin
TYSFYC	https://www.nature.com/articles/nbt1012	-		6	5,91651	-0,1367626	0,9116667	Pc-16a	French bean plastocyanin

YTSPHQGAGMV	https://www.nature.com/articles/nbt1012	-		11	7,54233	0,050632	0,4154545	Pc-17	French bean plastocyanin
MVGKVTVN	https://www.nature.com/articles/nbt1012	-		8	9,70002	0,9404096	0,445	Pc-18	French bean plastocyanin
RPDFSLEPPYTGPSK	https://www.nature.com/articles/nbt1012	-		15	6,48753	-0,0607726	0,2733333	P1-15	Bovine pancreatic trypsin inhibitor (BPTI)
PSKARIIRY	https://www.nature.com/articles/nbt1012	-		9	11,5305	3	0,2822222	P13-21	Bovine pancreatic trypsin inhibitor (BPTI)
KRNNFKSAEDS	https://www.nature.com/articles/nbt1012	-		11	9,53486	0,9408207	-0,3254545	P41-51	Bovine pancreatic trypsin inhibitor (BPTI)
ARIIRYFYNAKAG	https://www.nature.com/articles/nbt1012	-		13	10,6544	3	0,3561538	P16-28	Bovine pancreatic trypsin inhibitor (BPTI)
FKSAEDSMRTSGGA	https://www.nature.com/articles/nbt1012	-		14	6,49432	-0,05878131	0,02642857	P45-58	Bovine pancreatic trypsin inhibitor (BPTI)

NAKAGLSQT	https://www.nature.com/articles/nbt1012	-	9	9,70002	0,9404096	0,08111111	P24-32	Bovine pancreatic trypsin inhibitor (BPTI)
KGKKGEIKNVAD	https://www.nature.com/articles/nbt1012	-	12	10,3334	2	-0,22	Beta_2	N-terminal domain of ribosomal protein L9
LAIEATPA	https://www.nature.com/articles/nbt1012	-	8	3,84997	-1	0,59625	Beta_3	N-terminal domain of ribosomal protein L9
TPANLKALEAQKQKEQR	https://www.nature.com/articles/nbt1012	-	17	10,5006	2	-0,07117647	Alpha_2	N-terminal domain of ribosomal protein L9
DQKEAALVDMVNDGVEDLRCKYATLIYT	https://www.nature.com/articles/nbt1012	-	28	4,04406	-3	0,3046429	Alpha_4	Glutathione S transeferase P domain II (Glutex)

YEAGKEKYVKELPE HLKPFETLLSQ	https://www.nature.com/articles/nbt1012	-	25	5,68636	-0,9504464	0,258	Alpha_5	Glutathione S transeferase P domain II (Glutex)
PLLSAYVARLSA	https://www.nature.com/articles/nbt1012	-	12	9,34882	0,9388083	0,6533333	Alpha_7	Glutathione S transeferase P domain II (Glutex)
PKIKAFLA	https://www.nature.com/articles/nbt1012	-	8	10,8054	2	0,58125	Alpha_8	Glutathione S transeferase P domain II (Glutex)
AYVKKLDSGTGKEL VLAL	https://www.nature.com/articles/nbt1012	-	18	9,22014	0,9384394	0,37	M_2	Spectrin SH3
YDYQEKSPREVTM KKGD	https://www.nature.com/articles/nbt1012	-	17	6,61115	-0,06274272	-0,1005882	M_4	Spectrin SH3
GGKDWWKVGG	https://www.nature.com/articles/nbt1012	-	10	9,5373	0,9403278	0,297	M_C	Spectrin SH3
DILTLLNSTNKDW WKVEVNDRQGFV PA	https://www.nature.com/articles/nbt1012	-	27	4,49931	-1	0,3851852	M_681	Spectrin SH3
VPSNEEQIKNLLQL EAQEHLQY	https://www.nature.com/articles/nbt1012	-	22	4,1874	-3	0,285	H1_Wt	Spectrin SH3
VPSNEEQIKKLLELE AKKHLQY	https://www.nature.com/articles/nbt1012	-	22	7,54021	0,05104395	0,1972727	H1_Mt	Spectrin SH3
AVGKSNLLSRYAR NEFSA	https://www.nature.com/articles/nbt1012	-	18	10,4464	2	0,185	Ara1	Spectrin SH3

RFRAVTSAYYRGAVG	https://www.nature.com/articles/nbt1012	-		15	11,2103	3	0,2846667	Ara2	Spectrin SH3
TRRTTFESVGRWLDELKIHSD	https://www.nature.com/articles/nbt1012	-		21	7,55196	0,05383564	0,2119048	Ara3	Spectrin SH3
AVSVEEGKALAEEEGLF	https://www.nature.com/articles/nbt1012	-		17	3,7065	-4	0,2547059	Ara4	Spectrin SH3
DHPAVMEGTKTILETDSNLS	https://www.nature.com/articles/nbt1012	-		20	4,10372	-3	0,255	ComA1	Spectrin SH3
EPSEQFIKQHDFSSY	https://www.nature.com/articles/nbt1012	-		15	4,42148	-2	0,2393333	ComA2	Spectrin SH3
VNGMELSKQILQENPH	https://www.nature.com/articles/nbt1012	-		16	5,49306	-0,9467728	0,284375	ComA3	Spectrin SH3
EVEDYFEEAIRAGLH	https://www.nature.com/articles/nbt1012	-		15	3,986	-4	0,2586667	ComA4	Spectrin SH3
DFSTMRRIVRNLLKELGYN	https://www.nature.com/articles/nbt1012	-		19	10,4383	2	0,2994737	Che_Y1	Spectrin SH3
EDGVDALNKLQAGGY	https://www.nature.com/articles/nbt1012	-		15	3,876	-2	0,1473333	Che_Y2	Spectrin SH3
MDGLELLKTIRADSAY	https://www.nature.com/articles/nbt1012	-		16	4,37042	-1	0,359375	Che_Y3	Spectrin SH3
GTGNTEKMAELIAKGIIESGKDY	https://www.nature.com/articles/nbt1012	-		23	4,58761	-1	0,1795652	FXN1	Flavodoxin
EESEFEPFIEEISTKISY	https://www.nature.com/articles/nbt1012	-		18	3,61872	-5	0,3316667	FXN3	Flavodoxin
GDGKWMRDFEQRMNGYGSV	https://www.nature.com/articles/nbt1012	-		19	6,5488	-0,06046441	0,1384211	FXN4	Flavodoxin
EPDEAEQDSIEFGKKIANIY	https://www.nature.com/articles/nbt1012	-		20	3,84413	-4	0,1275	FXN5	Flavodoxin
EYSAMRDQYMRTGEG	https://www.nature.com/articles/nbt1012	-		15	4,42636	-1	0,04133333	P21B	Flavodoxin
INNTKSFEDIHQYREQIKRVKDS	https://www.nature.com/articles/nbt1012	-		23	9,2213	1	0,01	P21C	Flavodoxin

ARTVESRQAQDLARSYGIP	https://www.nature.com/articles/nbt1012	-		19	9,34644	0,9396095	0,1384211	P21D	Flavodoxin
TYKLINGKTLKGETTTEA	https://www.nature.com/articles/nbt1012	-		18	9,22014	0,9386243	0,1622222	ProteinG2-19	PL B1 protein
GDAATAEKVFKQYANDNGVD	https://www.nature.com/articles/nbt1012	-		20	4,0627	-2	0,017	ProteinG21-40	PL B1 protein
QGGGTHSQWN	https://www.nature.com/articles/nbt1012	-		10	7,55033	0,05262329	0,156	1-10	human prion protein
HSQWNKPSKP	https://www.nature.com/articles/nbt1012	-		10	10,8054	2	0,094	6-15	human prion protein
KPSKPKTNMK	https://www.nature.com/articles/nbt1012	-		10	11,2784	4	-0,167	11-20	human prion protein
KTNMKHMAGA	https://www.nature.com/articles/nbt1012	-		10	10,8054	2	0,089	16-25	human prion protein
HMAGAAAAGA	https://www.nature.com/articles/nbt1012	-		10	7,55033	0,05262329	0,322	21-30	human prion protein
AAAGAVVGGL	https://www.nature.com/articles/nbt1012	-		10	6,09998	-0,05919248	0,538	26-35	human prion protein
VVGGLGGYML	https://www.nature.com/articles/nbt1012	-		10	6,09322	-0,06118377	0,803	31-40	human prion protein
GGYMLGSAMS	https://www.nature.com/articles/nbt1012	-		10	6,09322	-0,06118377	0,535	36-45	human prion protein
GSAMSRPIIH	https://www.nature.com/articles/nbt1012	-		10	10,5518	1	0,49	41-50	human prion protein

FGSDYEDRYY	https://www.nature.com/articles/nbt1012	-	10	3,876	-2	0,144	51-60	human prion protein
EDRYYRENMH	https://www.nature.com/articles/nbt1012	-	10	5,54928	-0,9500572	-0,139	56-65	human prion protein
RENMHRYPNQ	https://www.nature.com/articles/nbt1012	-	10	9,35001	1	-0,104	61-70	human prion protein
RYPNQVYYRP	https://www.nature.com/articles/nbt1012	-	10	9,83185	2	0,27	66-75	human prion protein
VYYRPMDEYS	https://www.nature.com/articles/nbt1012	-	10	4,18442	-1	0,359	71-80	human prion protein
MDEYSNQNNF	https://www.nature.com/articles/nbt1012	-	10	3,55006	-2	0,051	76-85	human prion protein
NQNNFVHDCV	https://www.nature.com/articles/nbt1012	-	10	5,28988	-1	0,311	81-90	human prion protein
NITIKQHTVT	https://www.nature.com/articles/nbt1012	-	10	9,702	1	0,392	91-100	human prion protein
QHTVTTTTKG	https://www.nature.com/articles/nbt1012	-	10	9,702	1	0,144	96-105	human prion protein
TTTKGENFTE	https://www.nature.com/articles/nbt1012	-	10	4,25814	-1	-0,004	101-110	human prion protein
ENFTETDVKM	https://www.nature.com/articles/nbt1012	-	10	3,92756	-2	0,112	106-115	human prion protein

TDVKMMERVV	https://www.nature.com/articles/nbt1012	-	10	6,49432	-0,05878131	0,297	111-120	human prion protein
MERVVEQMCI	https://www.nature.com/articles/nbt1012	-	10	4,25811	-1	0,573	116-125	human prion protein
EQMCITQYER	https://www.nature.com/articles/nbt1012	-	10	4,25811	-1	0,306	121-130	human prion protein
TQYERESQAY	https://www.nature.com/articles/nbt1012	-	10	4,25814	-1	-0,028	126-135	human prion protein
ESQAYYQRGS	https://www.nature.com/articles/nbt1012	-	10	6,39669	-0,06268206	0,006	131-140	human prion protein
YQRGSSMVLF	https://www.nature.com/articles/nbt1012	-	10	9,34882	0,9388083	0,559	136-145	human prion protein
RTLKRLGMDG	https://www.nature.com/articles/nbt1012	-	10	11,4797	2	0,111	10-19	human lysozyme
LGMDGYRGIS	https://www.nature.com/articles/nbt1012	-	10	6,33191	-0,06087558	0,387	15-24	human lysozyme
YRGISLANWM	https://www.nature.com/articles/nbt1012	-	10	9,34882	0,9388083	0,66	20-29	human lysozyme
CLAKWESGYN	https://www.nature.com/articles/nbt1012	-	10	6,2288	-0,1346683	0,449	30-39	human lysozyme
ESGYNTRATN	https://www.nature.com/articles/nbt1012	-	10	6,40333	-0,06069078	-0,11	35-44	human lysozyme
TRATNYNAGD	https://www.nature.com/articles/nbt1012	-	10	6,33191	-0,06087558	-0,088	40-49	human lysozyme
YNAGDRSTDY	https://www.nature.com/articles/nbt1012	-	10	4,10927	-1	-0,07	45-54	human lysozyme

RSTDYGIFQI	https://www.nature.com/articles/nbt1012	-	10	6,33191	-0,06087558	0,457	50-59	human lysozyme
GIFQINSRYW	https://www.nature.com/articles/nbt1012	-	10	9,34882	0,9388083	0,673	55-64	human lysozyme
NSRYWCNDGK	https://www.nature.com/articles/nbt1012	-	10	8,52286	0,8651389	0,074	60-69	human lysozyme
CNDGKTPGAV	https://www.nature.com/articles/nbt1012	-	10	6,16057	-0,1328619	0,169	65-74	human lysozyme
TPGAVNACHL	https://www.nature.com/articles/nbt1012	-	10	7,35905	-0,02096427	0,559	70-79	human lysozyme
NACHLSCSAL	https://www.nature.com/articles/nbt1012	-	10	7,25315	-0,09455182	0,655	75-84	human lysozyme
LQDNIADAVA	https://www.nature.com/articles/nbt1012	-	10	3,4919	-2	0,329	85-94	human lysozyme
ADAVACAKRV	https://www.nature.com/articles/nbt1012	-	10	8,54512	0,8671302	0,245	90-99	human lysozyme
CAKRVVRDPQ	https://www.nature.com/articles/nbt1012	-	10	9,82824	2	0,101	95-104	human lysozyme
VRDPQGIRAW	https://www.nature.com/articles/nbt1012	-	10	10,3978	0,9411078	0,329	100-109	human lysozyme
GIRAWVAWRN	https://www.nature.com/articles/nbt1012	-	10	12,5001	2	0,552	105-114	human lysozyme
VAWRNRCQNR	https://www.nature.com/articles/nbt1012	-	10	12,1993	3	0,087	110-119	human lysozyme
RCQNRDVRQY	https://www.nature.com/articles/nbt1012	-	10	9,49871	2	-0,112	115-124	human lysozyme
DVRQYVQGCG	https://www.nature.com/articles/nbt1012	-	10	6,15812	-0,1344631	0,272	120-129	human lysozyme
RTPKIQVYSR	https://www.nature.com/articles/nbt1012	-	10	11,5305	3	0,169	4-13	β2-microglobulin

QVYSRHPAEN	https://www.nature.com/articles/nbt1012	-	10	7,54335	0,05112499	0,083	9-18	β2-microglobulin
HPAENGKSNF	https://www.nature.com/articles/nbt1012	-	10	7,54974	0,05272628	0,008	14-23	β2-microglobulin
LNCYVSGFHP	https://www.nature.com/articles/nbt1012	-	10	7,35552	-0,02295555	0,742	24-33	β2-microglobulin
SGFHPSDIEV	https://www.nature.com/articles/nbt1012	-	10	4,18118	-2	0,417	29-38	β2-microglobulin
SDIEVDLLKN	https://www.nature.com/articles/nbt1012	-	10	3,876	-2	0,261	34-43	β2-microglobulin
DLLKNGERIE	https://www.nature.com/articles/nbt1012	-	10	4,42637	-1	0,055	39-48	β2-microglobulin
GERIEKVEHS	https://www.nature.com/articles/nbt1012	-	10	5,57618	-0,9462798	-0,081	44-53	β2-microglobulin
KVEHSDLSFS	https://www.nature.com/articles/nbt1012	-	10	5,45428	-0,9469576	0,232	49-58	β2-microglobulin
TEKDEYACRV	https://www.nature.com/articles/nbt1012	-	10	4,42635	-1	0,024	74-83	β2-microglobulin
YACRVNHVTL	https://www.nature.com/articles/nbt1012	-	10	8,5348	0,9770365	0,573	79-88	β2-microglobulin
NHVTLSQPKI	https://www.nature.com/articles/nbt1012	-	10	9,702	1	0,398	84-93	β2-microglobulin

VHDCVNITIK	https://www.nature.com/articles/nbt1012		11	7,35902	-0,02104609	0,561	86-95	human prion protein
SMVLFSSPPV	https://www.nature.com/articles/nbt1012		10	6,09998	-0,05919248	0,848	41-150*	human prion protein
SSPPVILLIS	https://www.nature.com/articles/nbt1012		10	6,09998	-0,05919248	0,954	146-155	human prion protein
LLISFLIFL	https://www.nature.com/articles/nbt1012		9	6,09998	-0,05919248	1,548889	151-160*	human prion protein
FLIFLIVG	https://www.nature.com/articles/nbt1012		8	6,09998	-0,05919248	1,475	156-163	human prion protein
RCELARTLKR	https://www.nature.com/articles/nbt1012		15	11,3797	3	0,085	5 bis 15	human lysozyme
LANWMCLAKW	https://www.nature.com/articles/nbt1012		11	8,54518	0,866822	0,97	25-35	human lysozyme
DLSFSKDWSF	https://www.nature.com/articles/nbt1012		11	4,10922	-1	0,488	54-63	β-microglobulin
KDWSFYLLYY	https://www.nature.com/articles/nbt1012		10	6,31764	-0,06524817	0,852	59-68	β-microglobulin
YLLYYTEFTP	https://www.nature.com/articles/nbt1012		11	3,84997	-1	0,867	64-73	β-microglobulin
TEFTPTEKDE	https://www.nature.com/articles/nbt1012		10	3,77825	-3	-0,039	69-78	β-microglobulin

Louros, N. *et al.* WALTZ-DB 2.0: an updated database containing structural information of experimentally determined amyloid-forming peptides. *Nucleic Acids Res.* **48**, D389–D393 (2020).

Sequence	Source hyperlink	net charge	theoretical isolectric point	TEM fibrils observed	Th-T Binding	FTIR peaks	Proteostat binding	
VQIVRK	http://waltzdb.switchlab.org/sequences/VQIVRK	1,940402	11,65177	yes	yes			
KTKQGV	http://waltzdb.switchlab.org/sequences/KTKQGV	1,940012	10,80539	N.A.				
LKVKVL	http://waltzdb.switchlab.org/sequences/LKVKVL	1,940012	10,80539	N.A.	only high ph			
VQIVKK	http://waltzdb.switchlab.org/sequences/VQIVKK	1,940012	10,80539	yes	yes			
VQIRYK	http://waltzdb.switchlab.org/sequences/VQIRYK	1,93841	10,45493	yes	yes			
VRIVYK	http://waltzdb.switchlab.org/sequences/VRIVYK	1,93841	10,45493	yes	yes			
KQIVYK	http://waltzdb.switchlab.org/sequences/KQIVYK	1,93802	10,2476	yes	yes			
VKIVYK	http://waltzdb.switchlab.org/sequences/VKIVYK	1,93802	10,2476	yes	yes			
VQIKYK	http://waltzdb.switchlab.org/sequences/VQIKYK	1,93802	10,2476	yes	yes			
FAIRHF	http://waltzdb.switchlab.org/sequences/FAIRHF	1,052615	10,55177	N.A.				
TAVTHR	http://waltzdb.switchlab.org/sequences/TAVTHR	1,052615	10,55177	N.A.	ph 5.5			
KHIIVA	http://waltzdb.switchlab.org/sequences/KHIIVA	1,052225	9,702002	N.A.	only high ph			
QANKHI	http://waltzdb.switchlab.org/sequences/QANKHI	1,052225	9,702002	N.A.	omly low ph			
VQIVHK	http://waltzdb.switchlab.org/sequences/VQIVHK	1,052225	9,702002	yes	yes			
VHIVYK	http://waltzdb.switchlab.org/sequences/VHIVYK	1,050234	9,300705	yes	yes			
TCVTHR	http://waltzdb.switchlab.org/sequences/TCVTHR	0,979028	8,557474	N.A.	ph 5.5			
IVATTR	http://waltzdb.switchlab.org/sequences/IVATTR	0,9408	10,55	yes		1678.73/1646.17	0	
LATVRV	http://waltzdb.switchlab.org/sequences/LATVRV	0,9408	10,55	yes	no			
RQVLIF	http://waltzdb.switchlab.org/sequences/RQVLIF	0,9408	10,55	N.A.				
RVFNIM	http://waltzdb.switchlab.org/sequences/RVFNIM	0,9408	10,55	N.A.				

KAKEGV	http://waltzdb.switchlab.org/sequences/KAKEGV	0,940513	9,537315	N.A.				
KTKEGV	http://waltzdb.switchlab.org/sequences/KTKEGV	0,940513	9,537315	N.A.				
KTKEQV	http://waltzdb.switchlab.org/sequences/KTKEQV	0,940513	9,537315	N.A.				
GIFNIK	http://waltzdb.switchlab.org/sequences/GIFNIK	0,94041	9,700016	N.A.				
KLFIIQ	http://waltzdb.switchlab.org/sequences/KLFIIQ	0,94041	9,700016	N.A.				
KLFIQF	http://waltzdb.switchlab.org/sequences/KLFIQF	0,94041	9,700016	yes		1677.65/1650 .87/1629	16,11	
KLVFFA	http://waltzdb.switchlab.org/sequences/KLVFFA	0,94041	9,700016	N.A.				
KQIGII	http://waltzdb.switchlab.org/sequences/KQIGII	0,94041	9,700016	yes		1678.24/1648 /1637	49,97	
KVQIIN	http://waltzdb.switchlab.org/sequences/KVQIIN	0,94041	9,700016	N.A.				
LATVKV	http://waltzdb.switchlab.org/sequences/LATVKV	0,94041	9,700016	yes	no			
LIVAGK	http://waltzdb.switchlab.org/sequences/LIVAGK	0,94041	9,700016	N.A.				
NINKSN	http://waltzdb.switchlab.org/sequences/NINKSN	0,94041	9,700016	yes		1680/1645	0	
NKGAII	http://waltzdb.switchlab.org/sequences/NKGAII	0,94041	9,700016	N.A.				
VQIINK	http://waltzdb.switchlab.org/sequences/VQIINK	0,94041	9,700016	N.A.				
VQIINK	http://waltzdb.switchlab.org/sequences/VQIINK	0,94041	9,700016	N.A.				
VQIVAK	http://waltzdb.switchlab.org/sequences/VQIVAK	0,94041	9,700016	yes	yes			
VQIVFK	http://waltzdb.switchlab.org/sequences/VQIVFK	0,94041	9,700016	yes	yes			
VQIVGK	http://waltzdb.switchlab.org/sequences/VQIVGK	0,94041	9,700016	yes	yes			
VQIVIK	http://waltzdb.switchlab.org/sequences/VQIVIK	0,94041	9,700016	yes	yes			
VQIVLK	http://waltzdb.switchlab.org/sequences/VQIVLK	0,94041	9,700016	yes				
VQIVMK	http://waltzdb.switchlab.org/sequences/VQIVMK	0,94041	9,700016	yes	yes			
VQIVNK	http://waltzdb.switchlab.org/sequences/VQIVNK	0,94041	9,700016	yes	yes			
VQIVPK	http://waltzdb.switchlab.org/sequences/VQIVPK	0,94041	9,700016	yes	no			
VQIVQK	http://waltzdb.switchlab.org/sequences/VQIVQK	0,94041	9,700016	yes	yes			
VQIVSK	http://waltzdb.switchlab.org/sequences/VQIVSK	0,94041	9,700016	yes	yes			
VQIVTK	http://waltzdb.switchlab.org/sequences/VQIVTK	0,94041	9,700016	yes	yes			
VQIVVK	http://waltzdb.switchlab.org/sequences/VQIVVK	0,94041	9,700016	yes	yes			
VQIVWK	http://waltzdb.switchlab.org/sequences/VQIVWK	0,94041	9,700016	yes	yes			

KIVKWD	http://waltzdb.switchlab.org/sequences/KIVKWD	0,940328	9,537301	N.A.					
RATVYV	http://waltzdb.switchlab.org/sequences/RATVYV	0,938808	9,348818	yes	yes				
VQIVYR	http://waltzdb.switchlab.org/sequences/VQIVYR	0,938808	9,348818	yes	yes				
FQIVYK	http://waltzdb.switchlab.org/sequences/FQIVYK	0,938418	9,298295	yes	yes				
GYVIIK	http://waltzdb.switchlab.org/sequences/GYVIIK	0,938418	9,298295	N.A.					
IQIVYK	http://waltzdb.switchlab.org/sequences/IQIVYK	0,938418	9,298295	yes	yes				
KATVYV	http://waltzdb.switchlab.org/sequences/KATVYV	0,938418	9,298295	yes	no				
KNFNYN	http://waltzdb.switchlab.org/sequences/KNFNYN	0,938418	9,298295	N.A.					
LQIVYK	http://waltzdb.switchlab.org/sequences/LQIVYK	0,938418	9,298295	yes	yes				
MQIVYK	http://waltzdb.switchlab.org/sequences/MQIVYK	0,938418	9,298295	yes	yes				
NQIVYK	http://waltzdb.switchlab.org/sequences/NQIVYK	0,938418	9,298295	yes	no				
QQIVYK	http://waltzdb.switchlab.org/sequences/QQIVYK	0,938418	9,298295	yes	yes				
SQIVYK	http://waltzdb.switchlab.org/sequences/SQIVYK	0,938418	9,298295	yes	yes				
TQIVYK	http://waltzdb.switchlab.org/sequences/TQIVYK	0,938418	9,298295	yes	no				
VAIVYK	http://waltzdb.switchlab.org/sequences/VAIVYK	0,938418	9,298295	yes	yes				
VFIVYK	http://waltzdb.switchlab.org/sequences/VFIVYK	0,938418	9,298295	yes	yes				
VGIVYK	http://waltzdb.switchlab.org/sequences/VGIVYK	0,938418	9,298295	yes	yes				
VIIVYK	http://waltzdb.switchlab.org/sequences/VIIVYK	0,938418	9,298295	yes	yes				
VLIVYK	http://waltzdb.switchlab.org/sequences/VLIVYK	0,938418	9,298295	yes	yes				
VMIVYK	http://waltzdb.switchlab.org/sequences/VMIVYK	0,938418	9,298295	yes	yes				
VNIVYK	http://waltzdb.switchlab.org/sequences/VNIVYK	0,938418	9,298295	yes	yes				
VQFVYK	http://waltzdb.switchlab.org/sequences/VQFVYK	0,938418	9,298295	yes	yes				
VQIFYK	http://waltzdb.switchlab.org/sequences/VQIFYK	0,938418	9,298295	yes	yes				
VQIIYK	http://waltzdb.switchlab.org/sequences/VQIIYK	0,938418	9,298295	yes	yes				
VQILYK	http://waltzdb.switchlab.org/sequences/VQILYK	0,938418	9,298295	yes	yes				
VQIMYK	http://waltzdb.switchlab.org/sequences/VQIMYK	0,938418	9,298295	yes	yes				
VQINYK	http://waltzdb.switchlab.org/sequences/VQINYK	0,938418	9,298295	yes	yes				
VQIQYK	http://waltzdb.switchlab.org/sequences/VQIQYK	0,938418	9,298295	yes	yes				
VQISYK	http://waltzdb.switchlab.org/sequences/VQISYK	0,938418	9,298295	yes	yes				
VQITYK	http://waltzdb.switchlab.org/sequences/VQITYK	0,938418	9,298295	yes	yes				
VQIVYK	http://waltzdb.switchlab.org/sequences/VQIVYK	0,938418	9,298295	N.A.					
VQIWYK	http://waltzdb.switchlab.org/sequences/VQIWYK	0,938418	9,298295	yes	yes				
VQLVYK	http://waltzdb.switchlab.org/sequences/VQLVYK	0,938418	9,298295	yes	yes				

VQMVYK	http://waltzdb.switchlab.org/sequences/VQMVYK	0,938418	9,298295	yes	yes			
VQNVYK	http://waltzdb.switchlab.org/sequences/VQNVYK	0,938418	9,298295	yes	yes			
VQQVYK	http://waltzdb.switchlab.org/sequences/VQQVYK	0,938418	9,298295	yes	no			
VQTVYK	http://waltzdb.switchlab.org/sequences/VQTVYK	0,938418	9,298295	yes	yes			
VQVVYK	http://waltzdb.switchlab.org/sequences/VQVVYK	0,938418	9,298295	yes	yes			
VSIVYK	http://waltzdb.switchlab.org/sequences/VSIVYK	0,938418	9,298295	yes	yes			
VTIVYK	http://waltzdb.switchlab.org/sequences/VTIVYK	0,938418	9,298295	yes	yes			
VVIVYK	http://waltzdb.switchlab.org/sequences/VVIVYK	0,938418	9,298295	yes	yes			
VWIVYK	http://waltzdb.switchlab.org/sequences/VWIVYK	0,938418	9,298295	yes	yes			
WQIVYK	http://waltzdb.switchlab.org/sequences/WQIVYK	0,938418	9,298295	yes	yes			
VQIYYK	http://waltzdb.switchlab.org/sequences/VQIYYK	0,936427	9,145597	yes	yes			
VYIVYK	http://waltzdb.switchlab.org/sequences/VYIVYK	0,936427	9,145597	yes	yes			
YQIVYK	http://waltzdb.switchlab.org/sequences/YQIVYK	0,936427	9,145597	yes	yes			
QGVCFR	http://waltzdb.switchlab.org/sequences/QGVCFR	0,867212	8,549921	N.A.				
VQIVCK	http://waltzdb.switchlab.org/sequences/VQIVCK	0,866822	8,545179	yes	yes			
CQIVYK	http://waltzdb.switchlab.org/sequences/CQIVYK	0,864831	8,52295	yes	yes			
KCLNYL	http://waltzdb.switchlab.org/sequences/KCLNYL	0,864831	8,52295	N.A.				
VCIVYK	http://waltzdb.switchlab.org/sequences/VCIVYK	0,864831	8,52295	yes	yes			
VQCVYK	http://waltzdb.switchlab.org/sequences/VQCVYK	0,864831	8,52295	yes	yes			
VQICYK	http://waltzdb.switchlab.org/sequences/VQICYK	0,864831	8,52295	yes	yes			
KCWCFT	http://waltzdb.switchlab.org/sequences/KCWCFT	0,793235	8,229679	N.A.				
