# Supplementary material for: Data-mining unveils structure–property–activity correlation of viral infectivity enhancing self-assembling peptides
Source: Nat Commun. 2023 Aug 23;14:5121. doi: 10.1038/s41467-023-40663-6 (PMC10447463; doi:10.1038/s41467-023-40663-6)
Supplement: Supplementary file 7 — Source Data [file 41467_2023_40663_MOESM7_ESM.zip › Source-Data/SI/FigS34-38-39/summary_FTIR_library.pptx]

## Slide 1
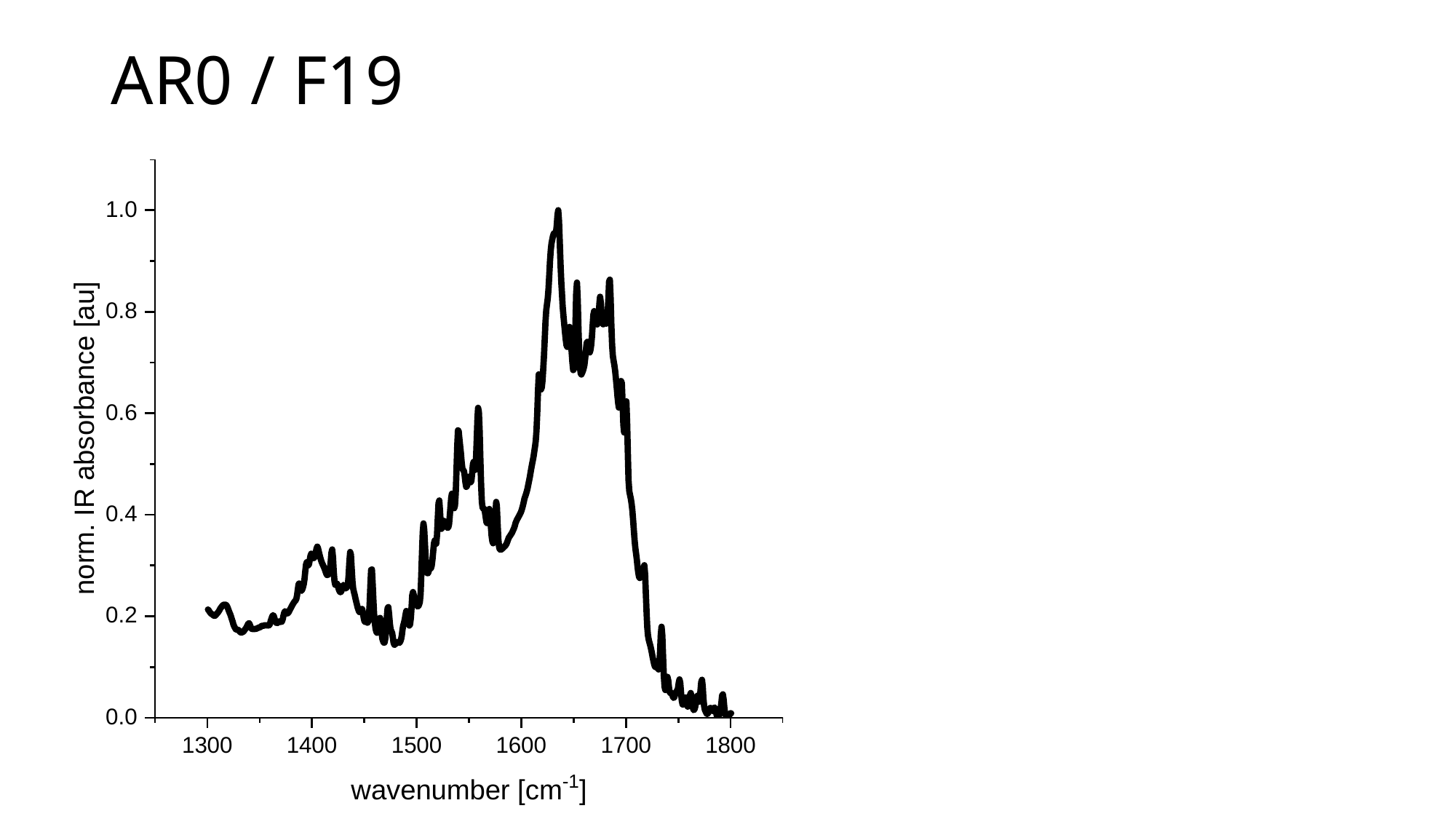

# AR0 / F19

## Slide 2
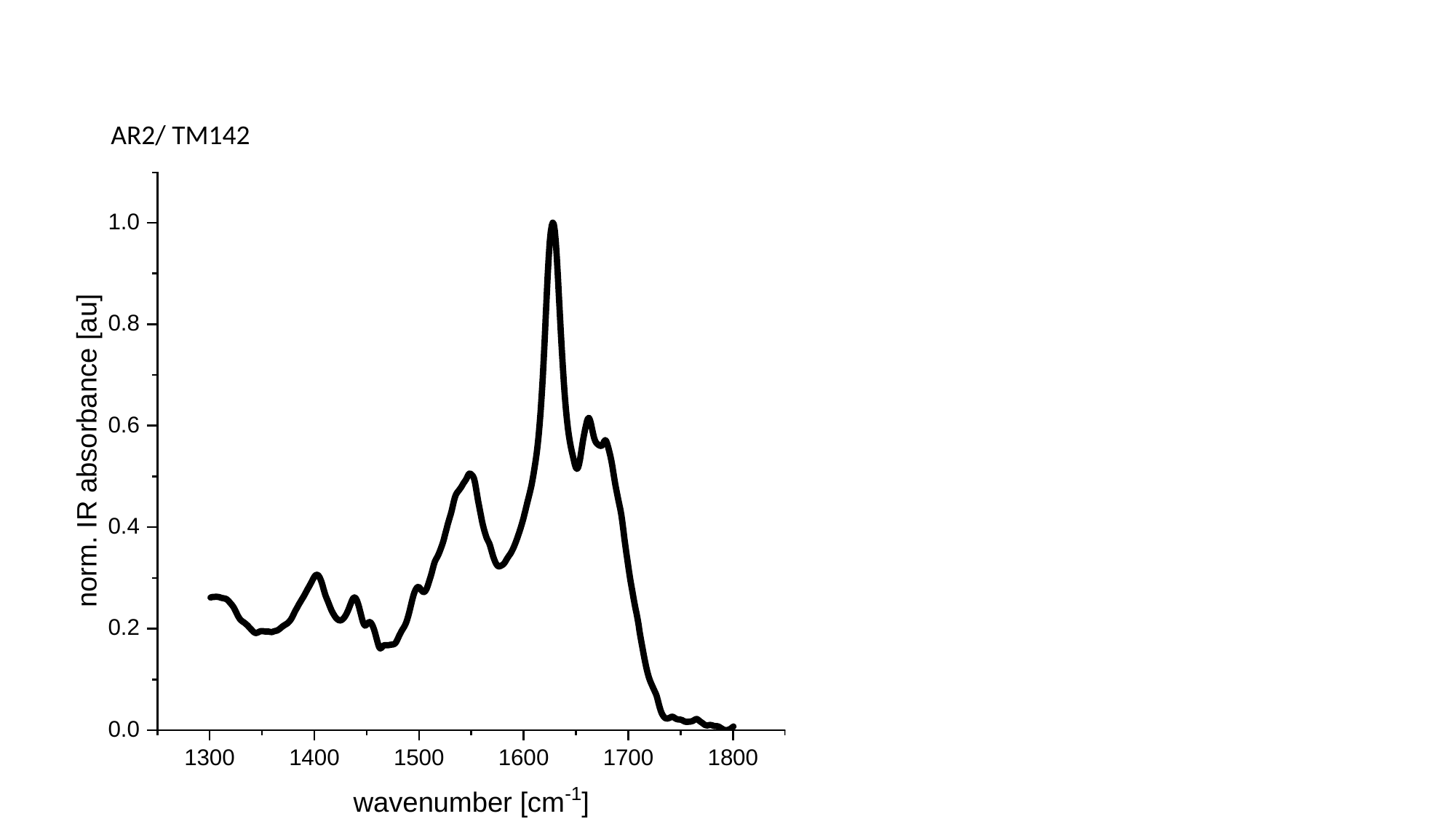

# AR2/ TM142

## Slide 3
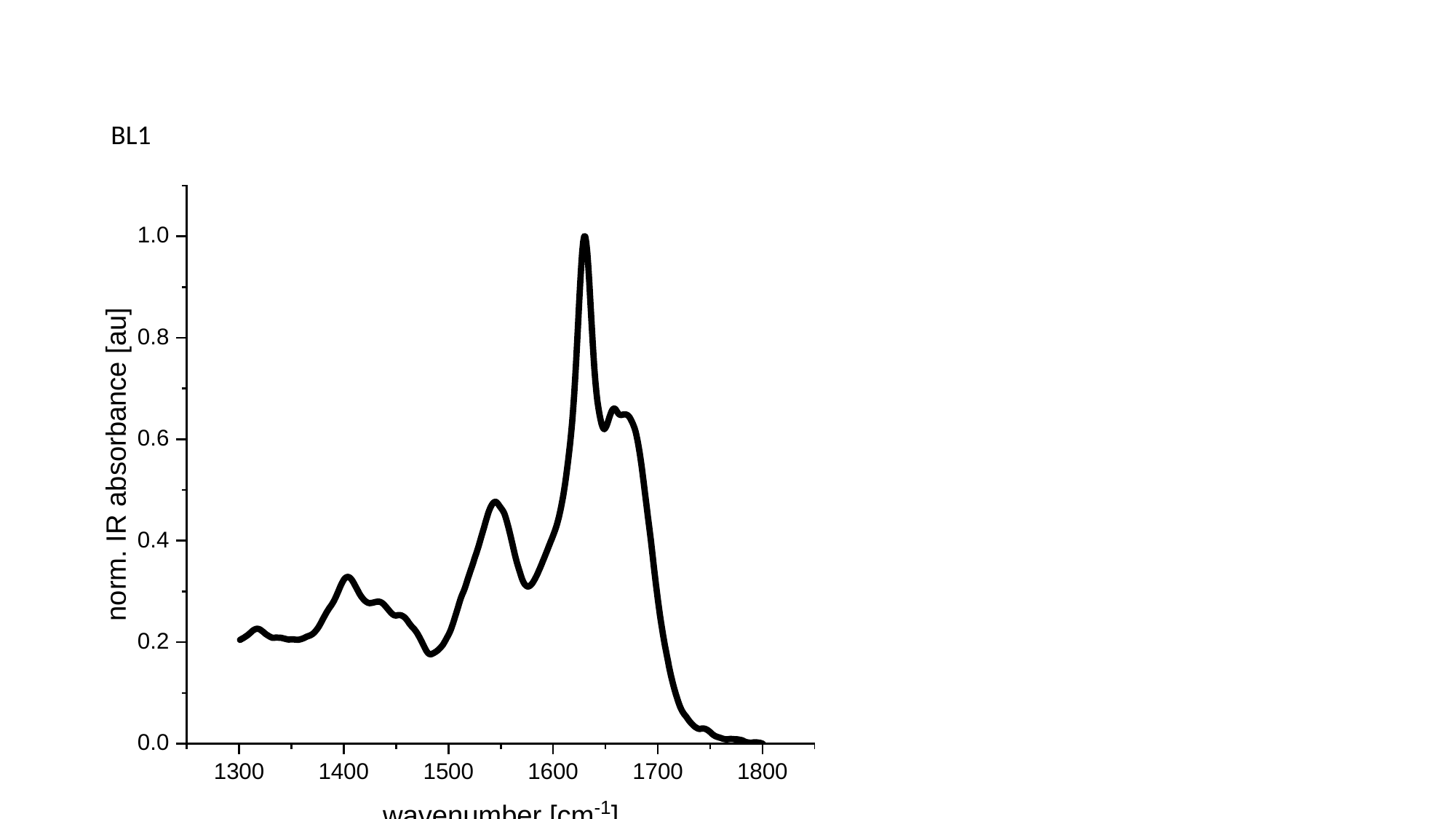

# BL1

## Slide 4
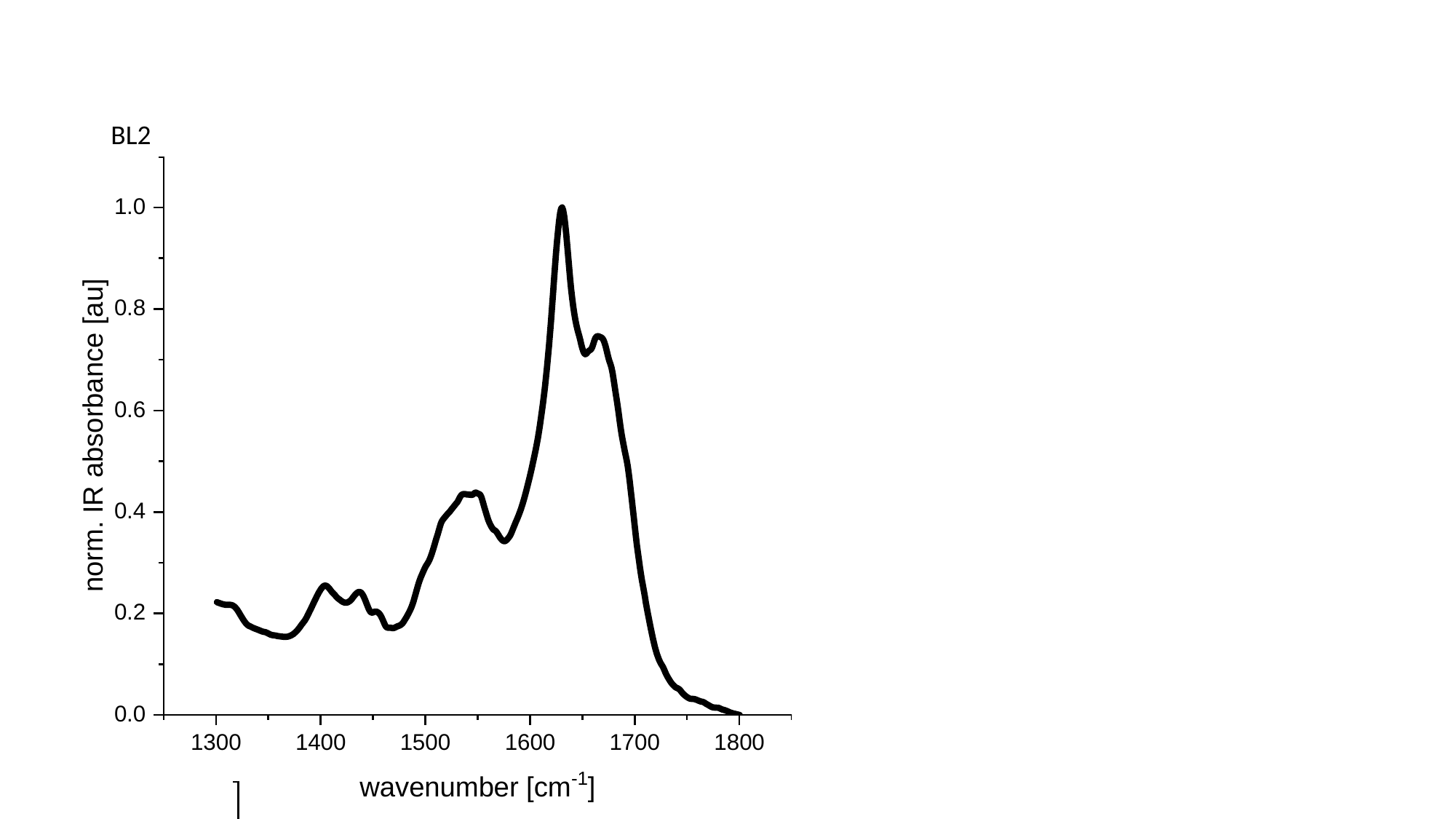

# BL2

## Slide 5
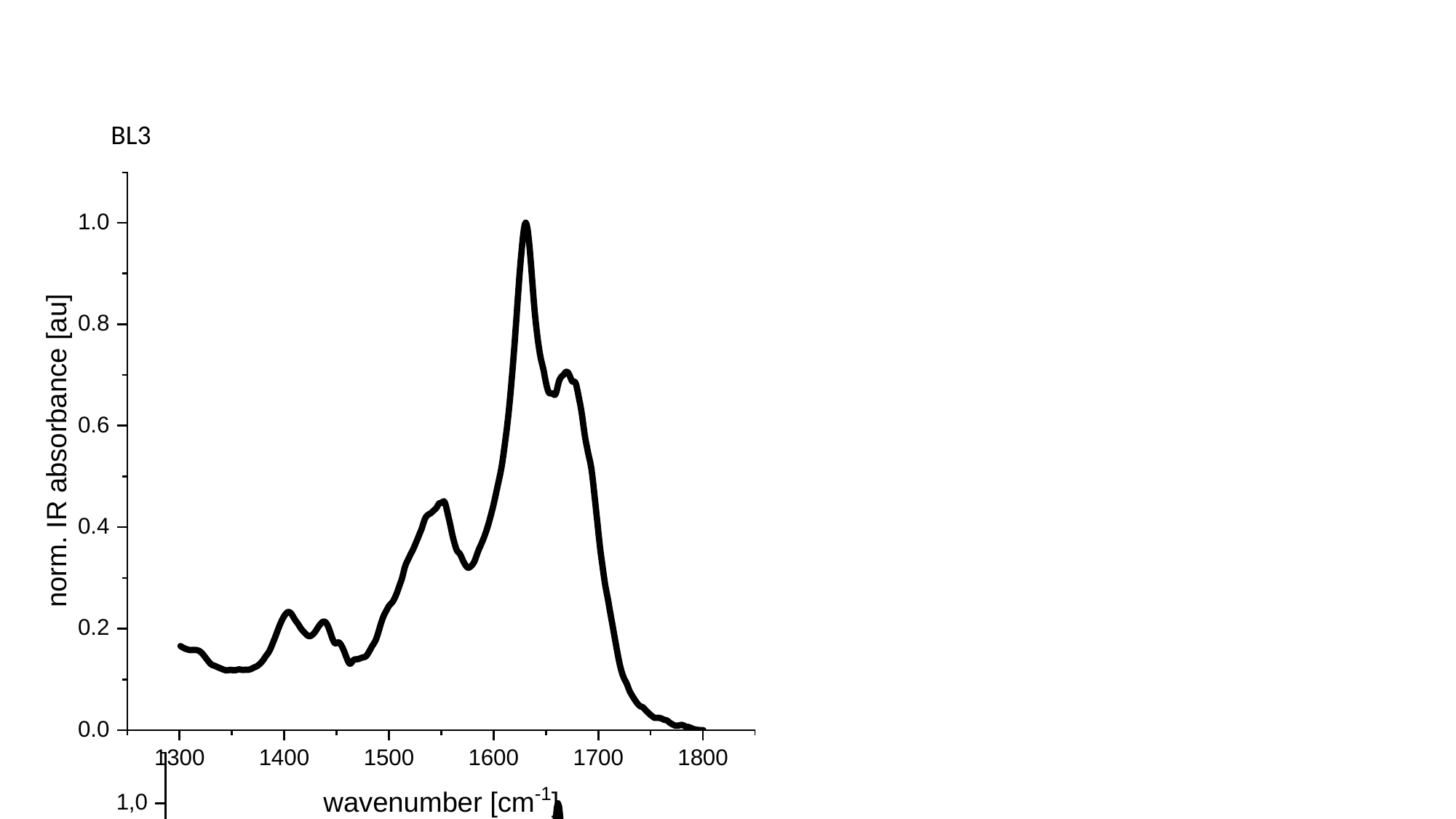

# BL3

## Slide 6
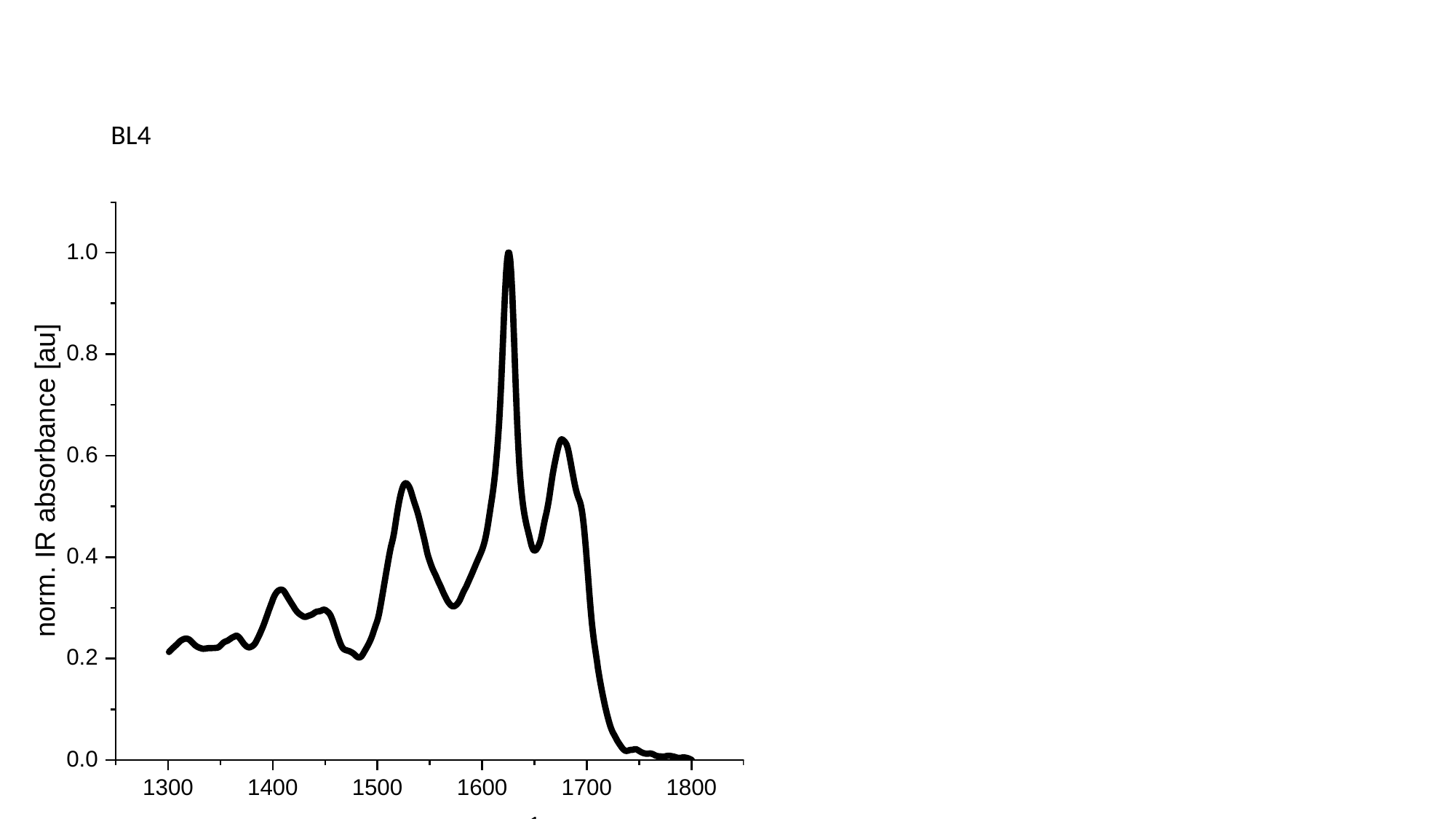

# BL4

## Slide 7
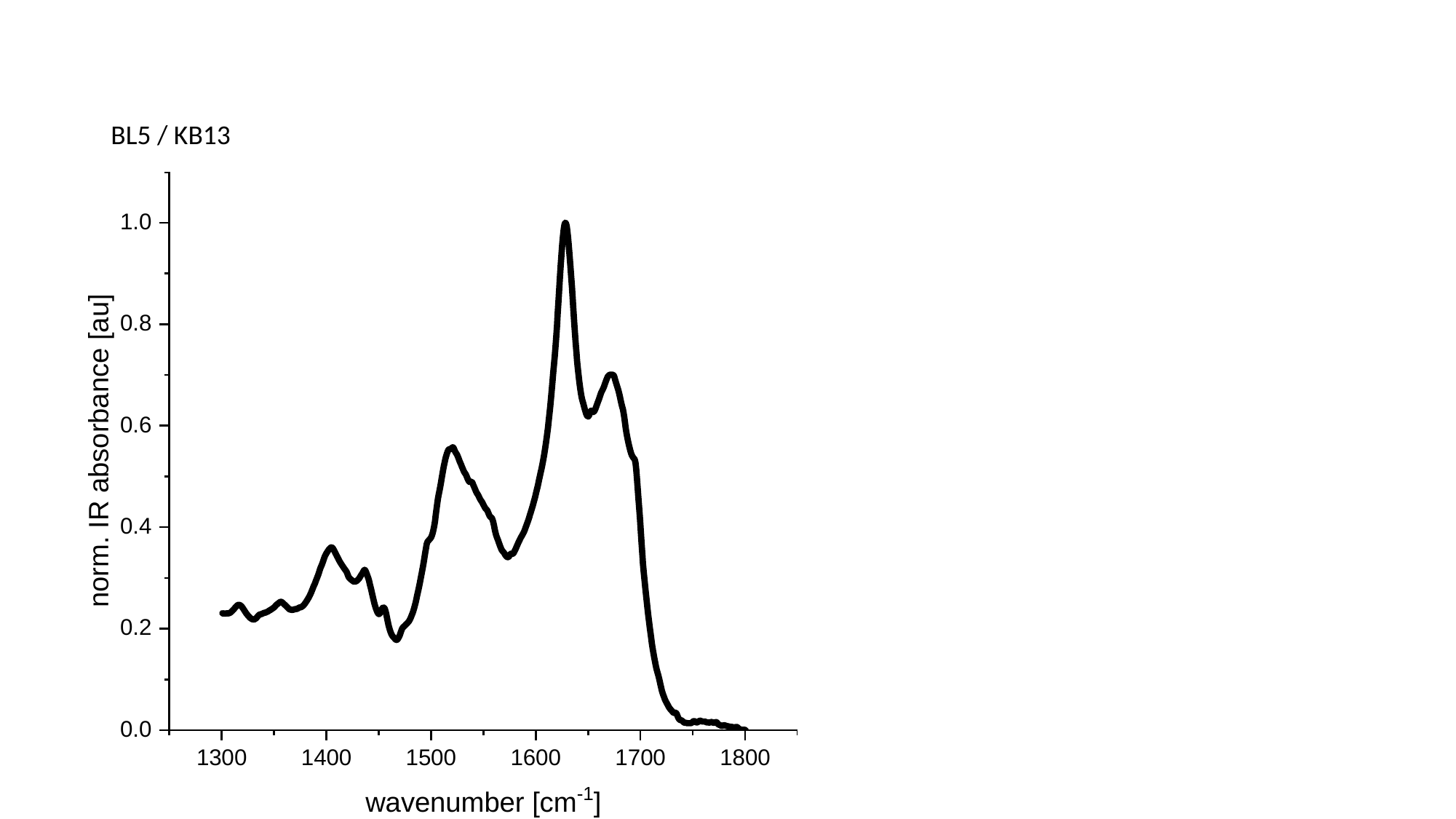

# BL5 / KB13

## Slide 8
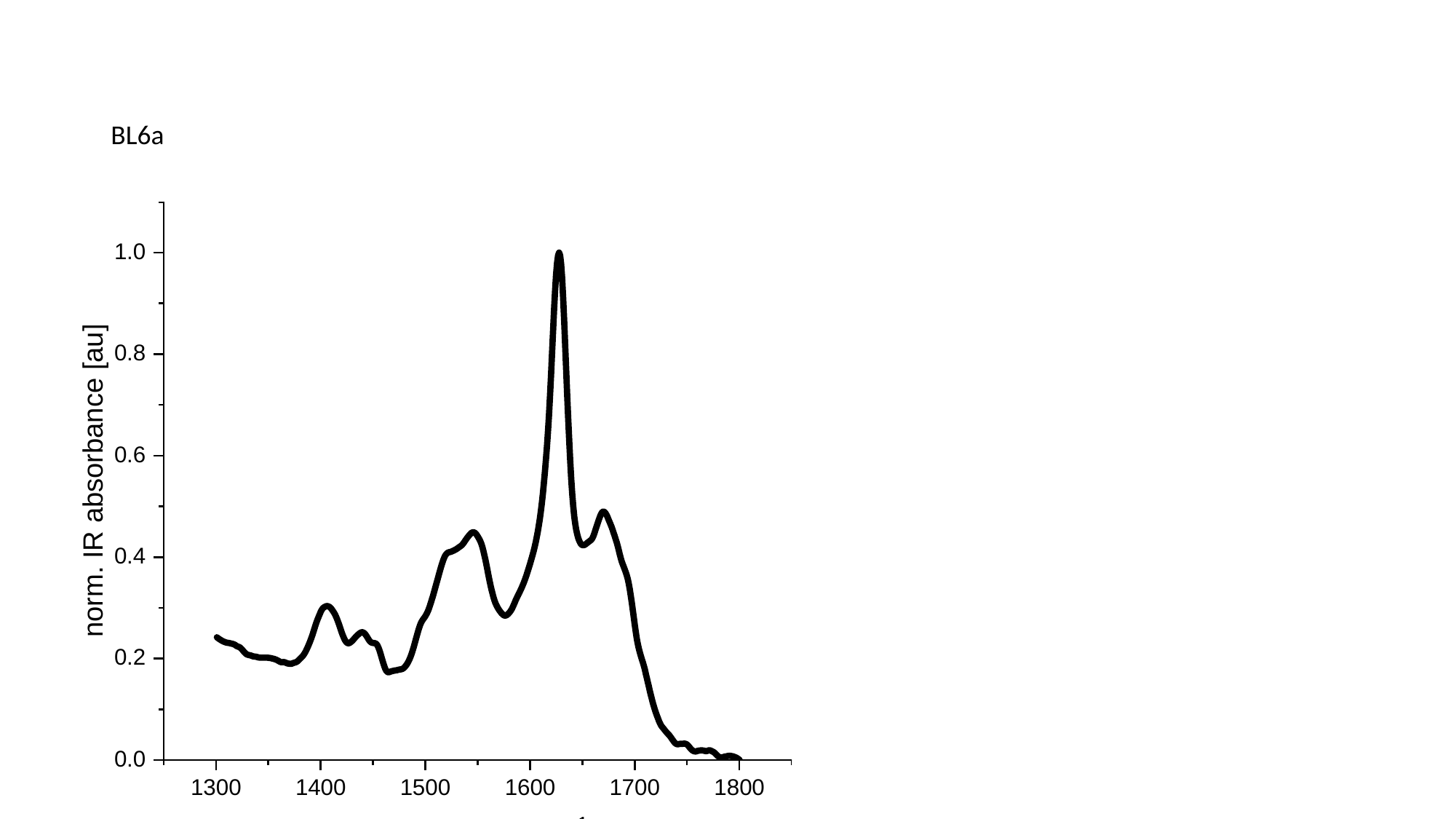

# BL6a

## Slide 9
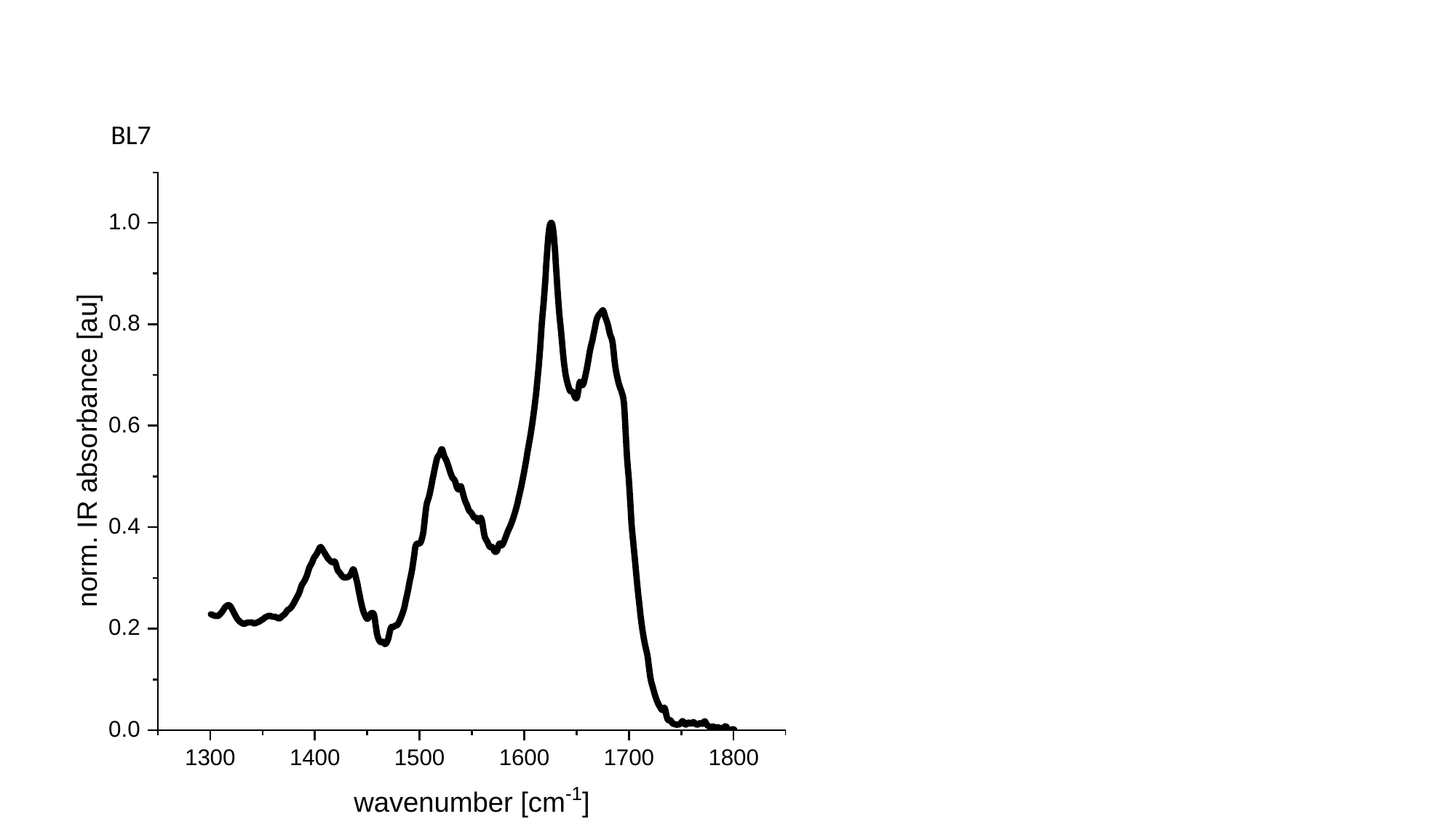

# BL7

## Slide 10
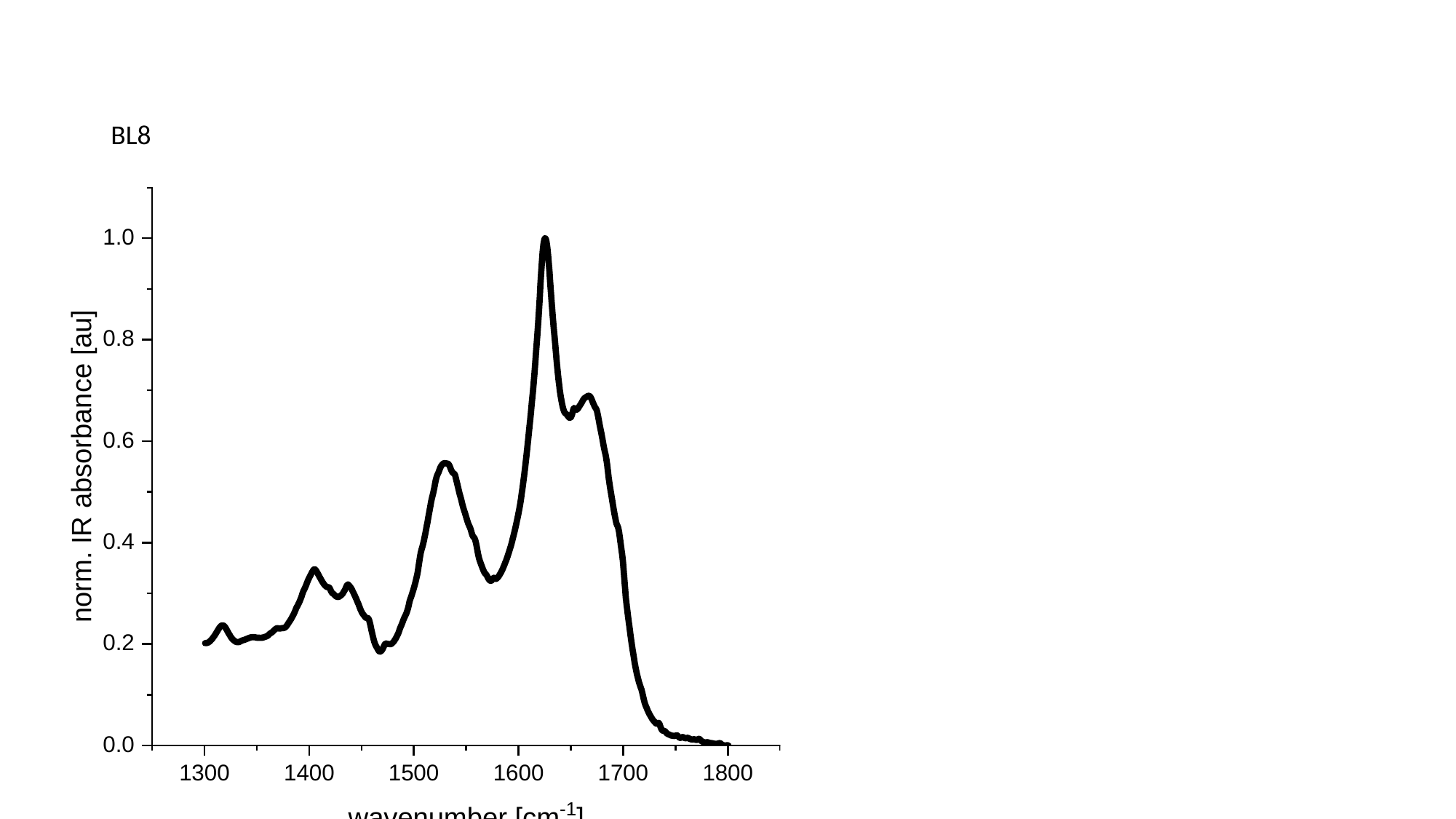

# BL8

## Slide 11
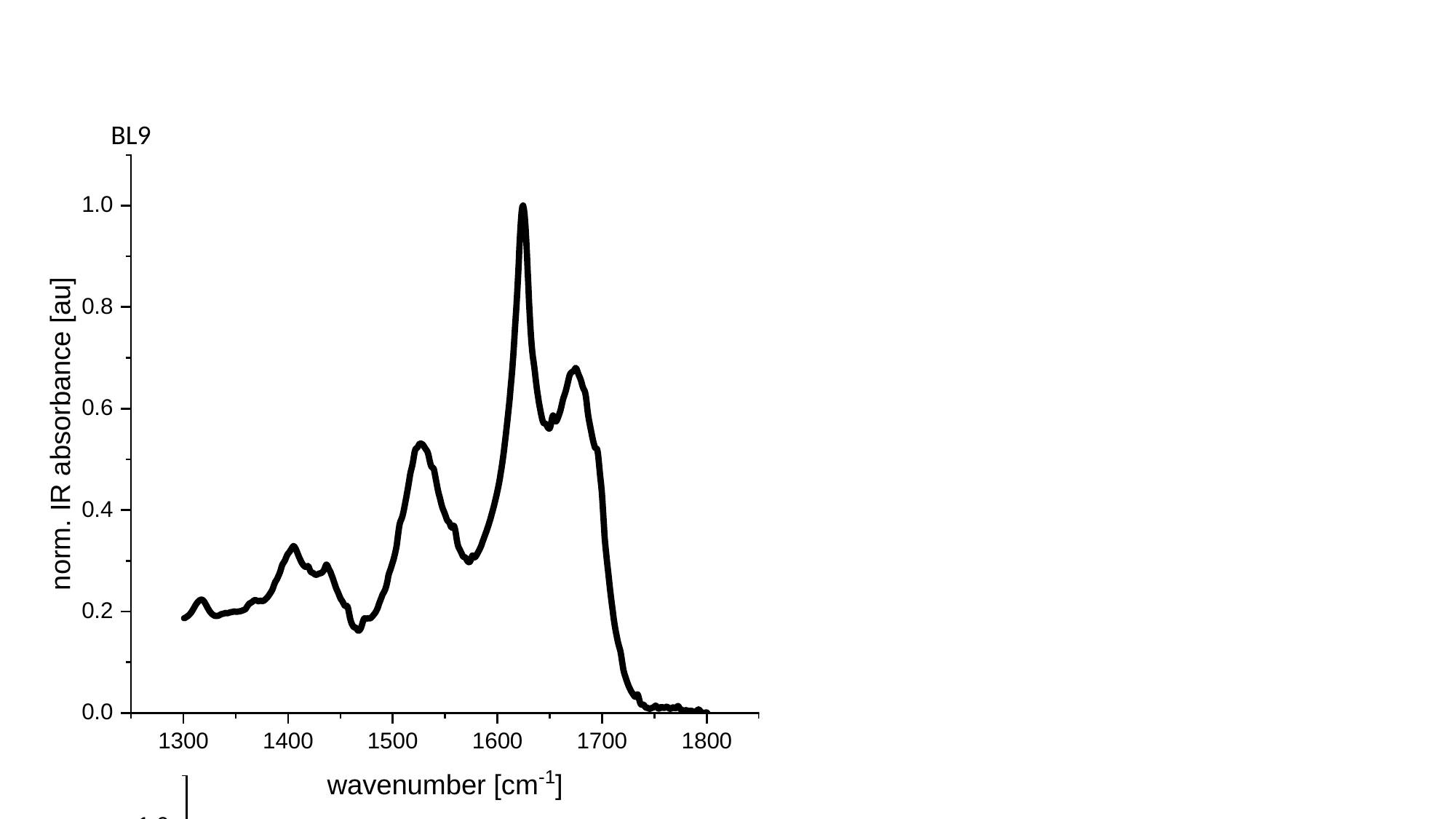

# BL9

## Slide 12
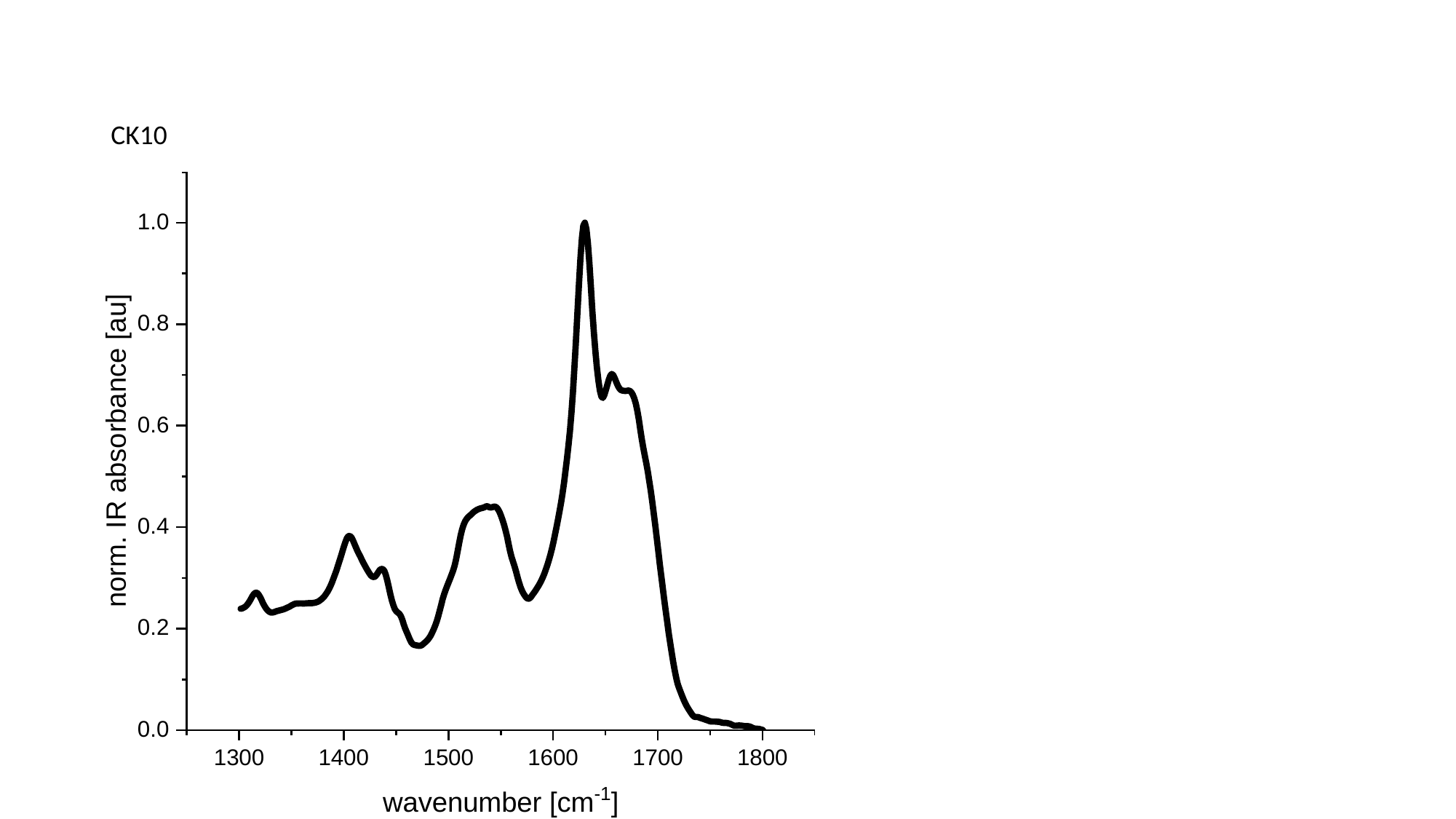

# CK10

## Slide 13
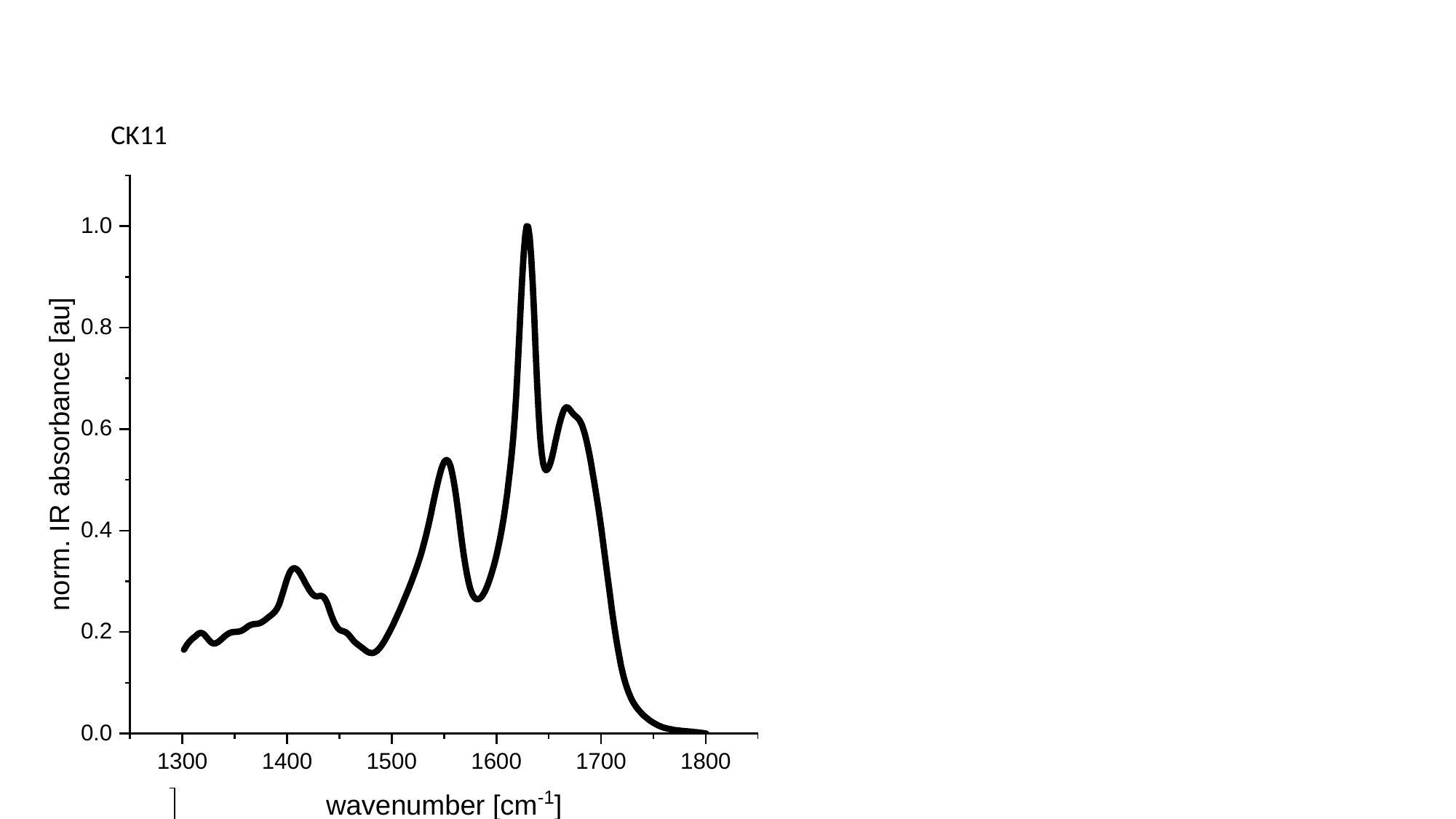

# CK11

## Slide 14
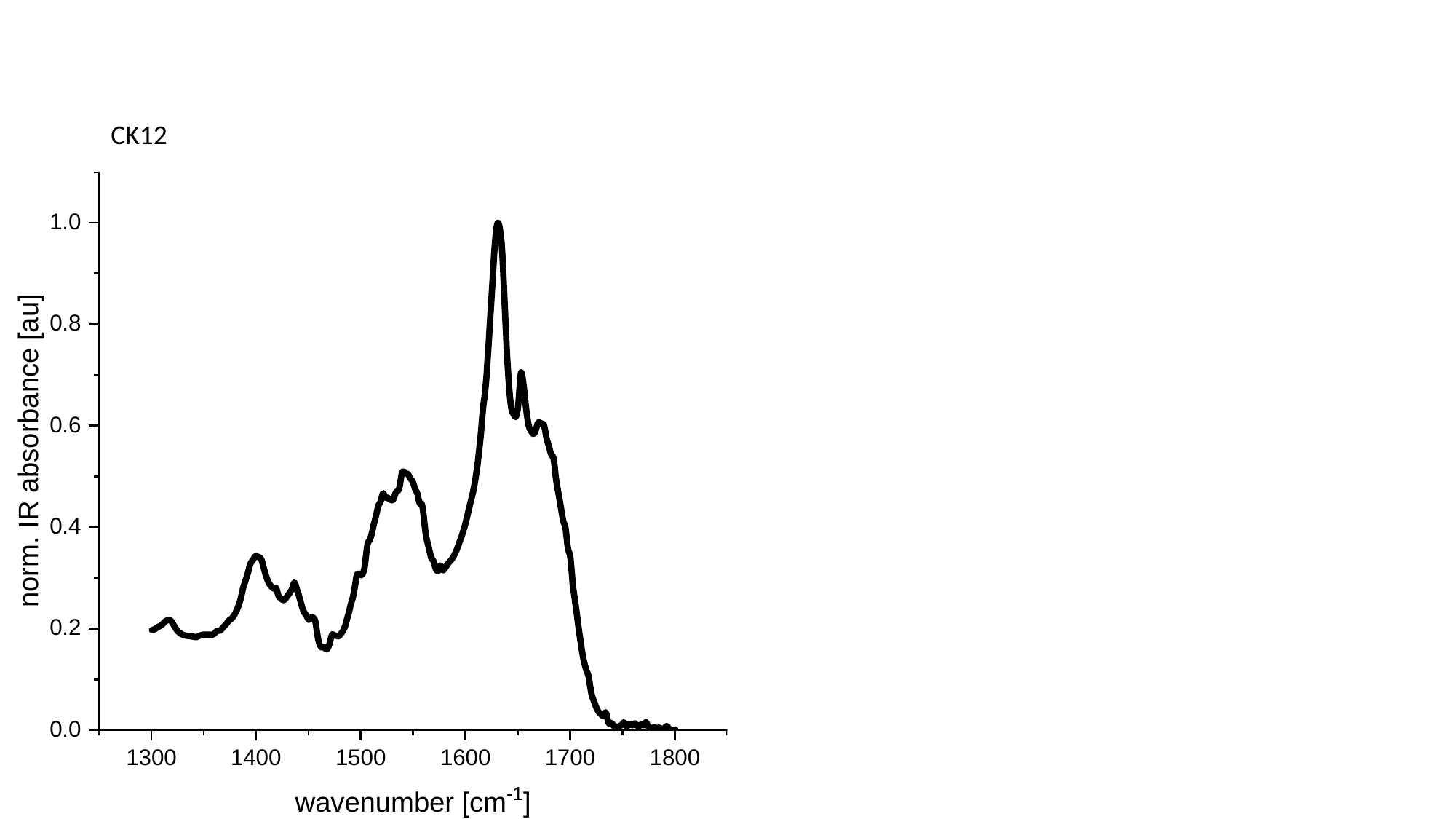

# CK12

## Slide 15
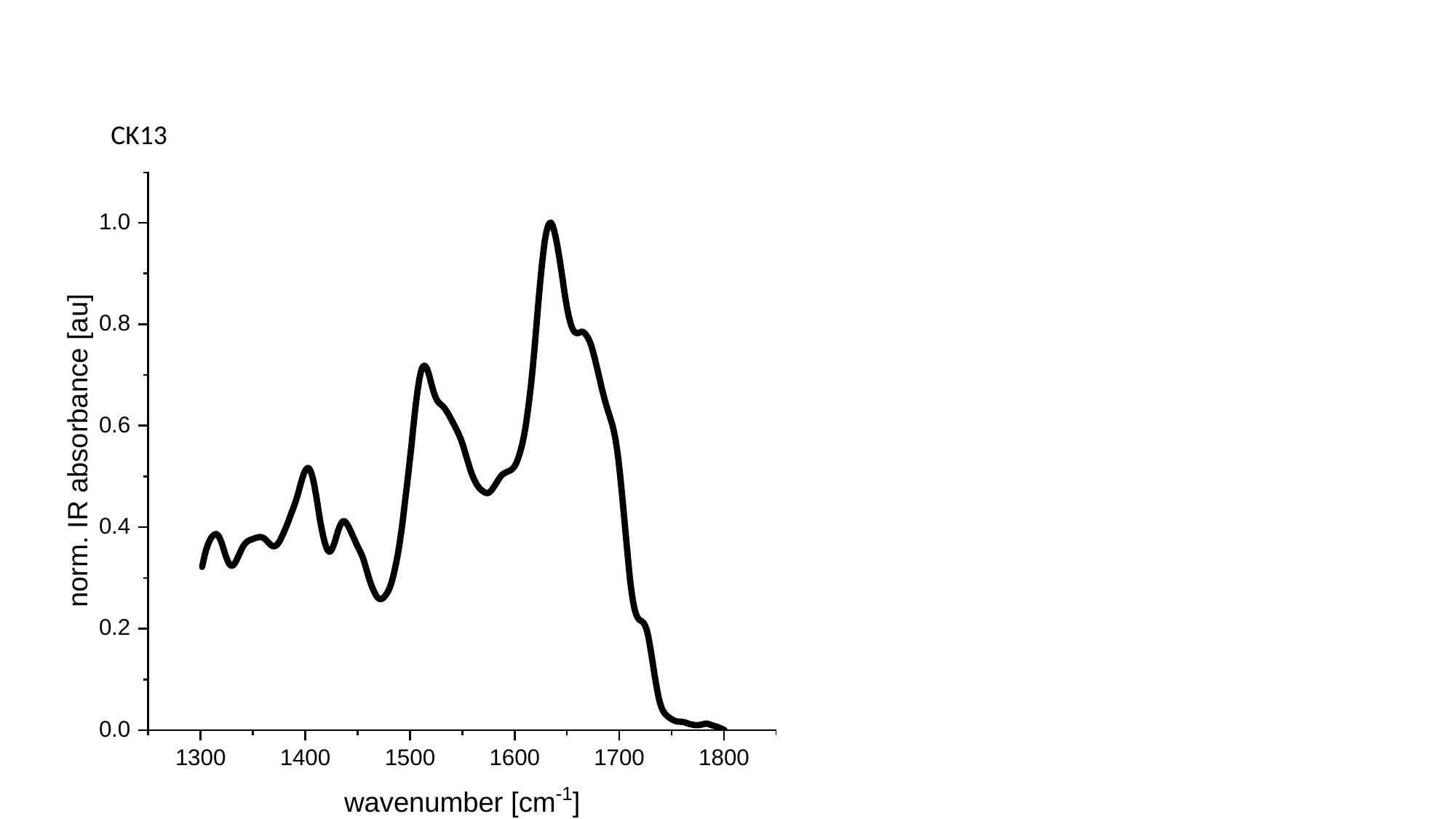

# CK13

## Slide 16
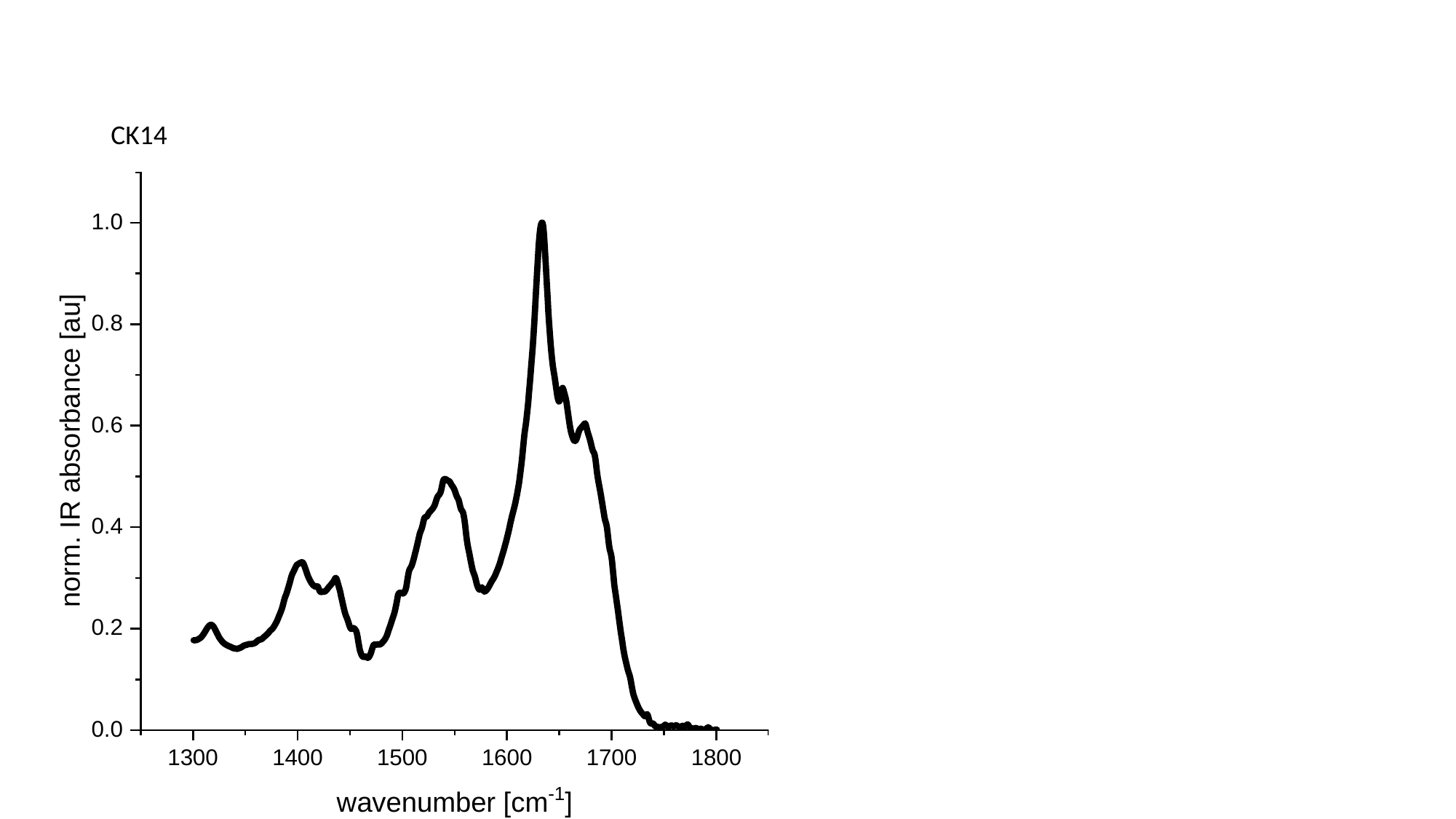

# CK14

## Slide 17
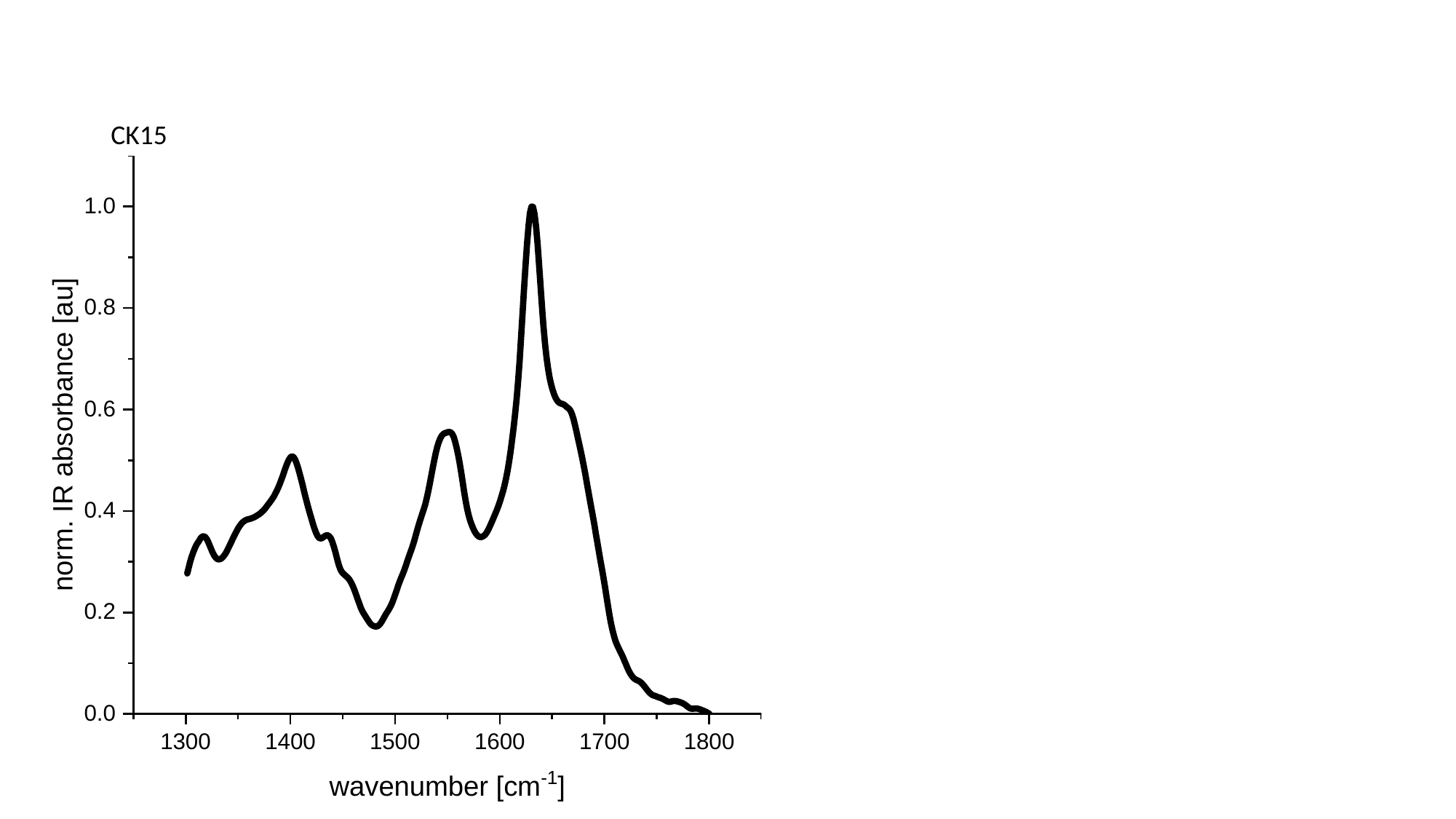

# CK15

## Slide 18
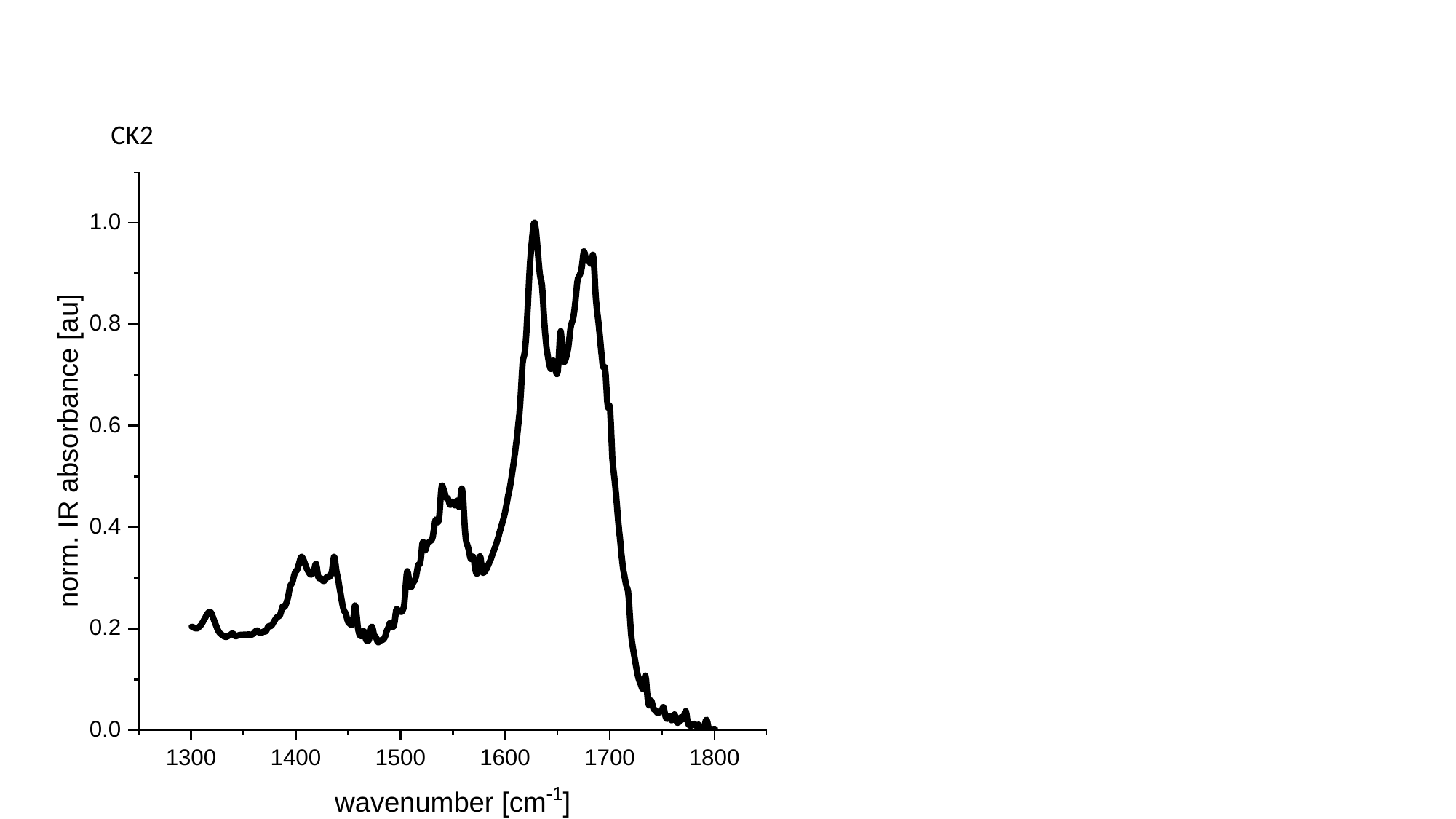

# CK2

## Slide 19
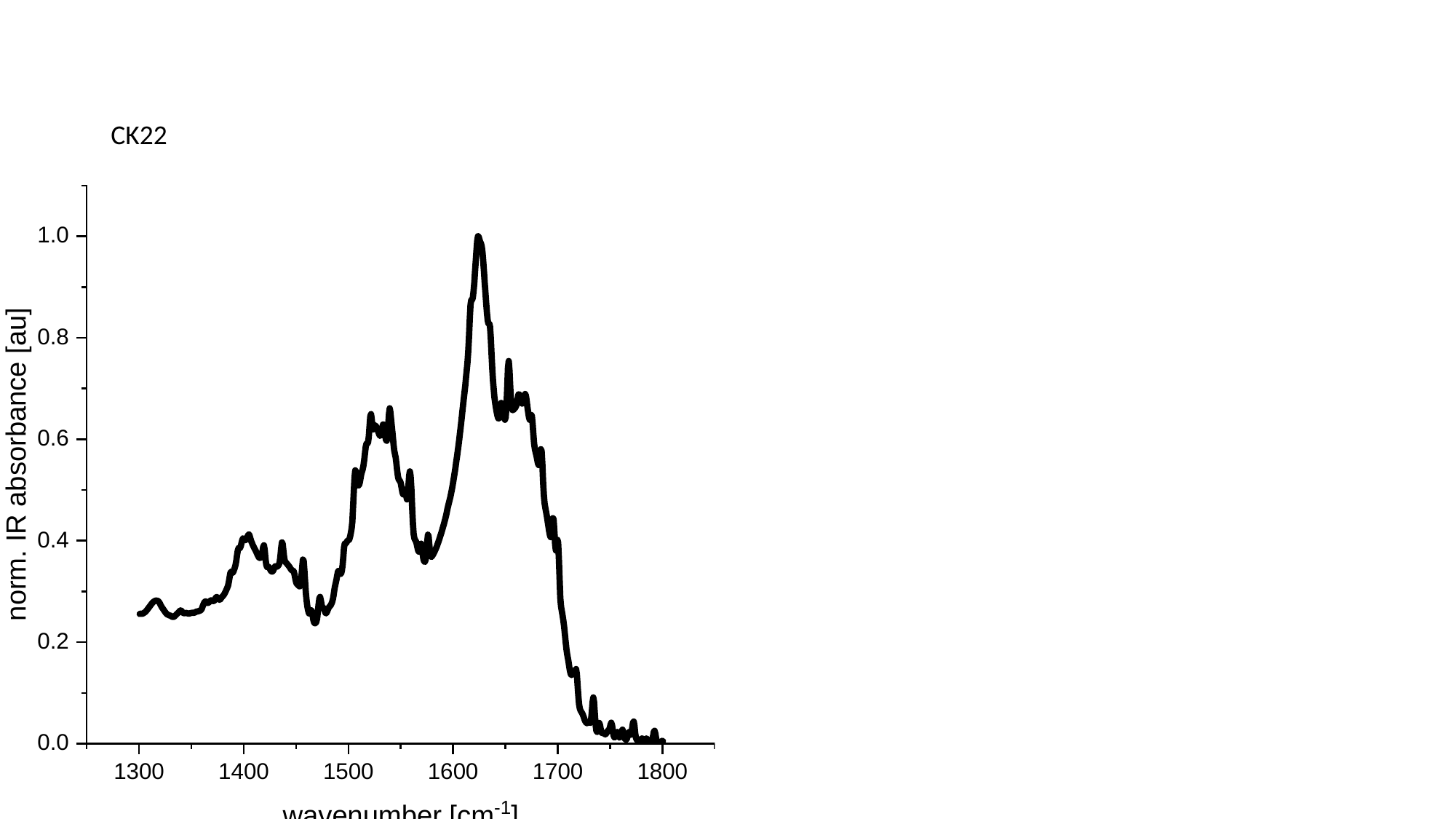

# CK22

## Slide 20
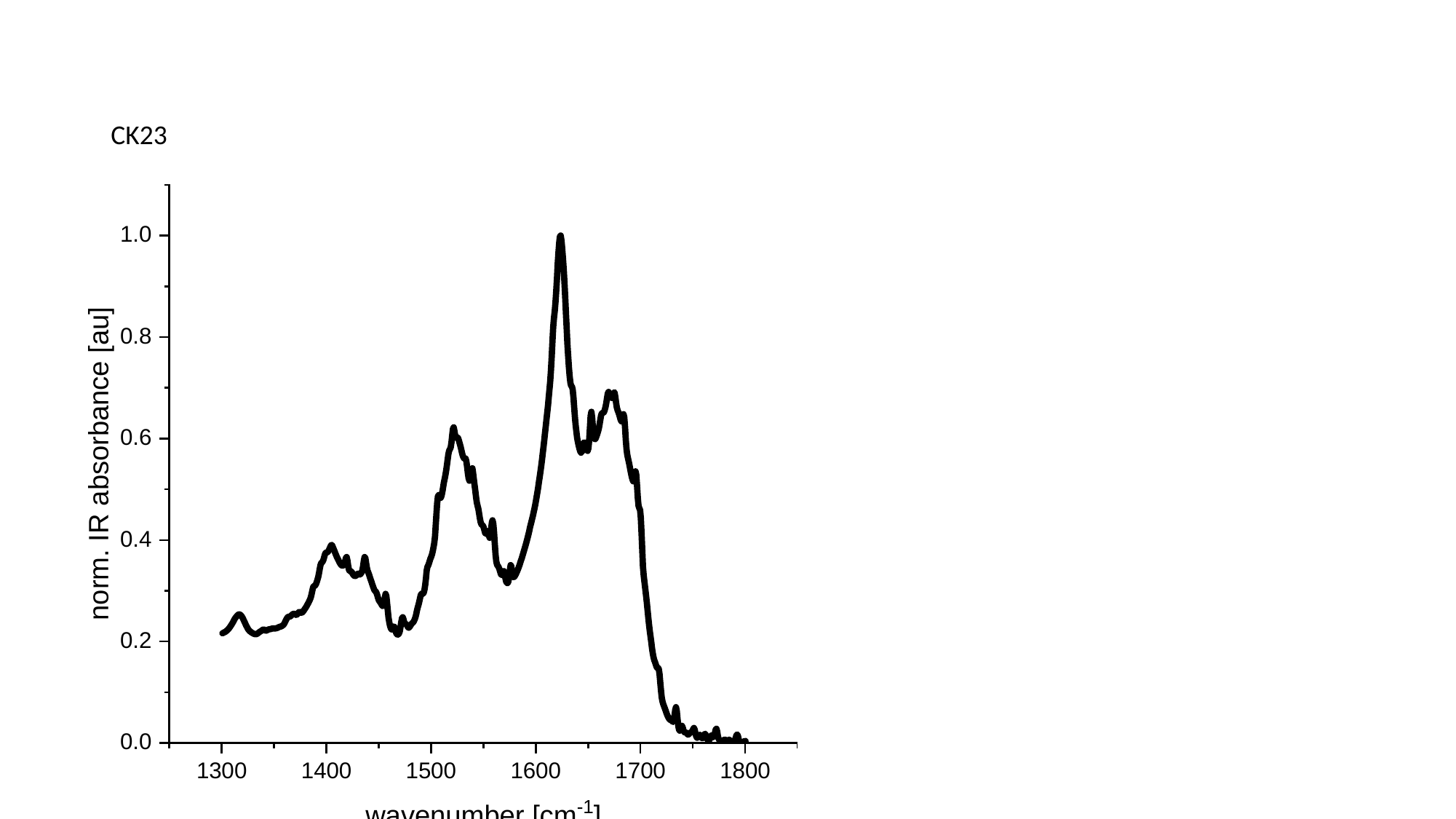

# CK23

## Slide 21
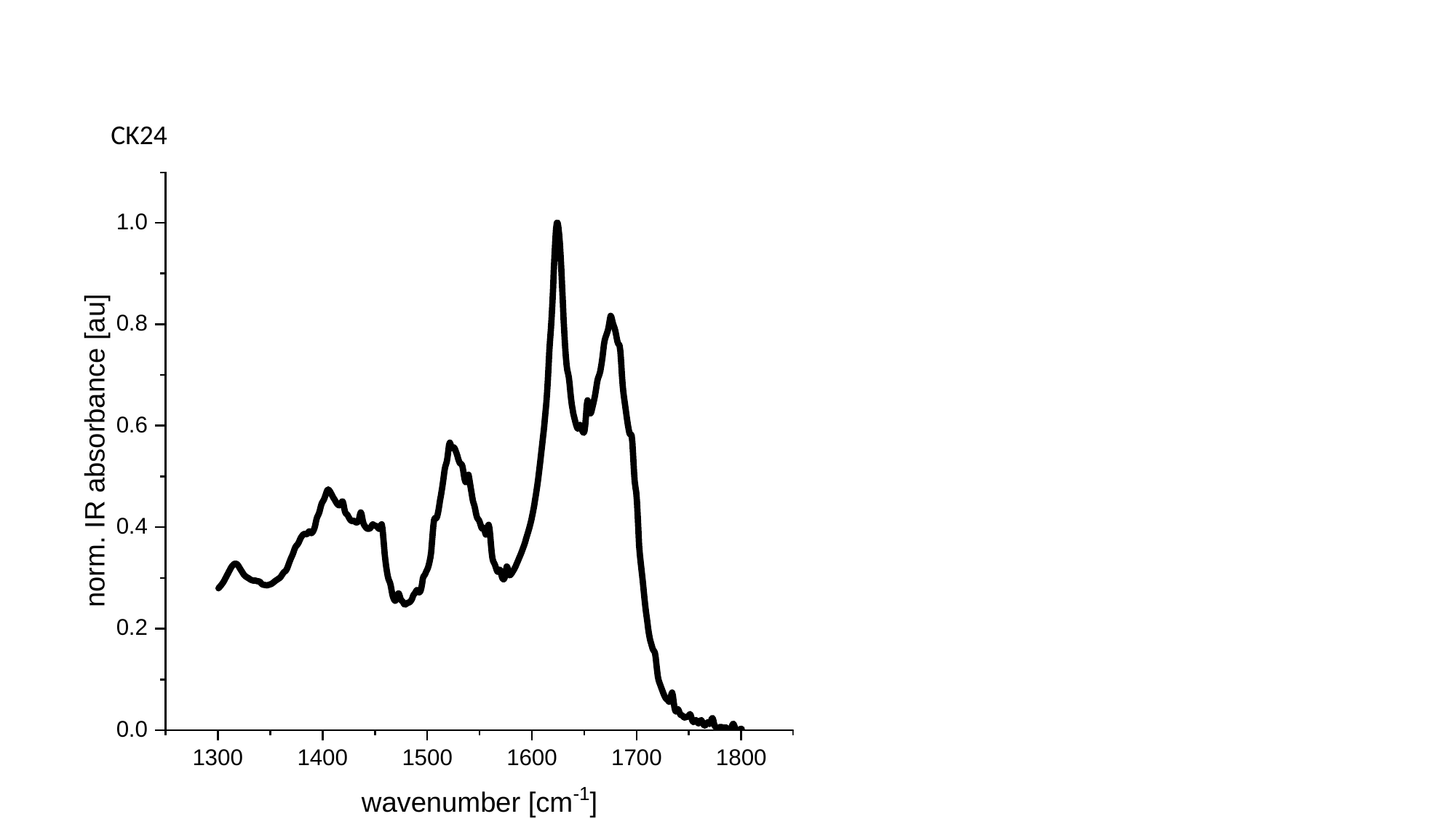

# CK24

## Slide 22
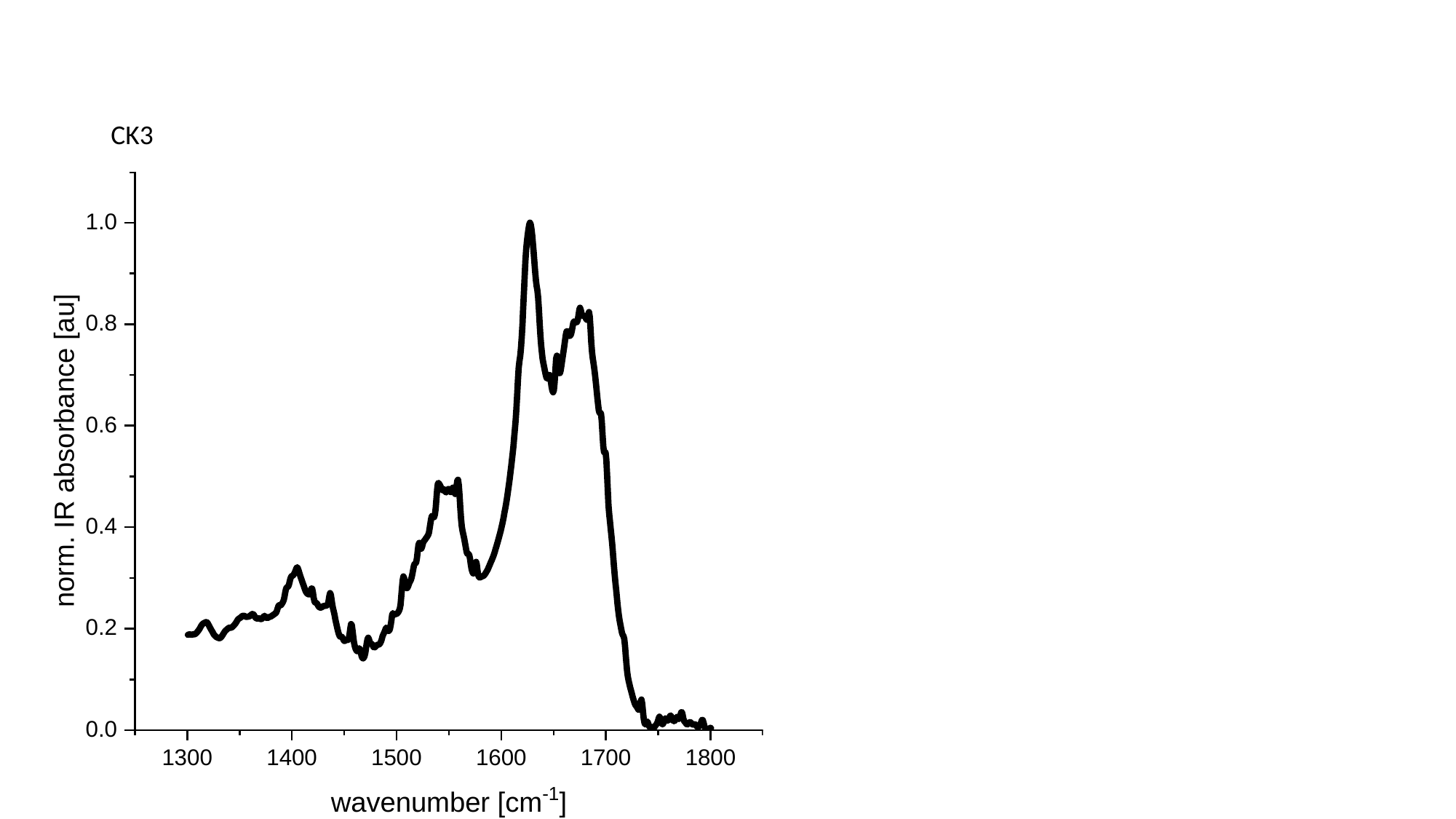

# CK3

## Slide 23
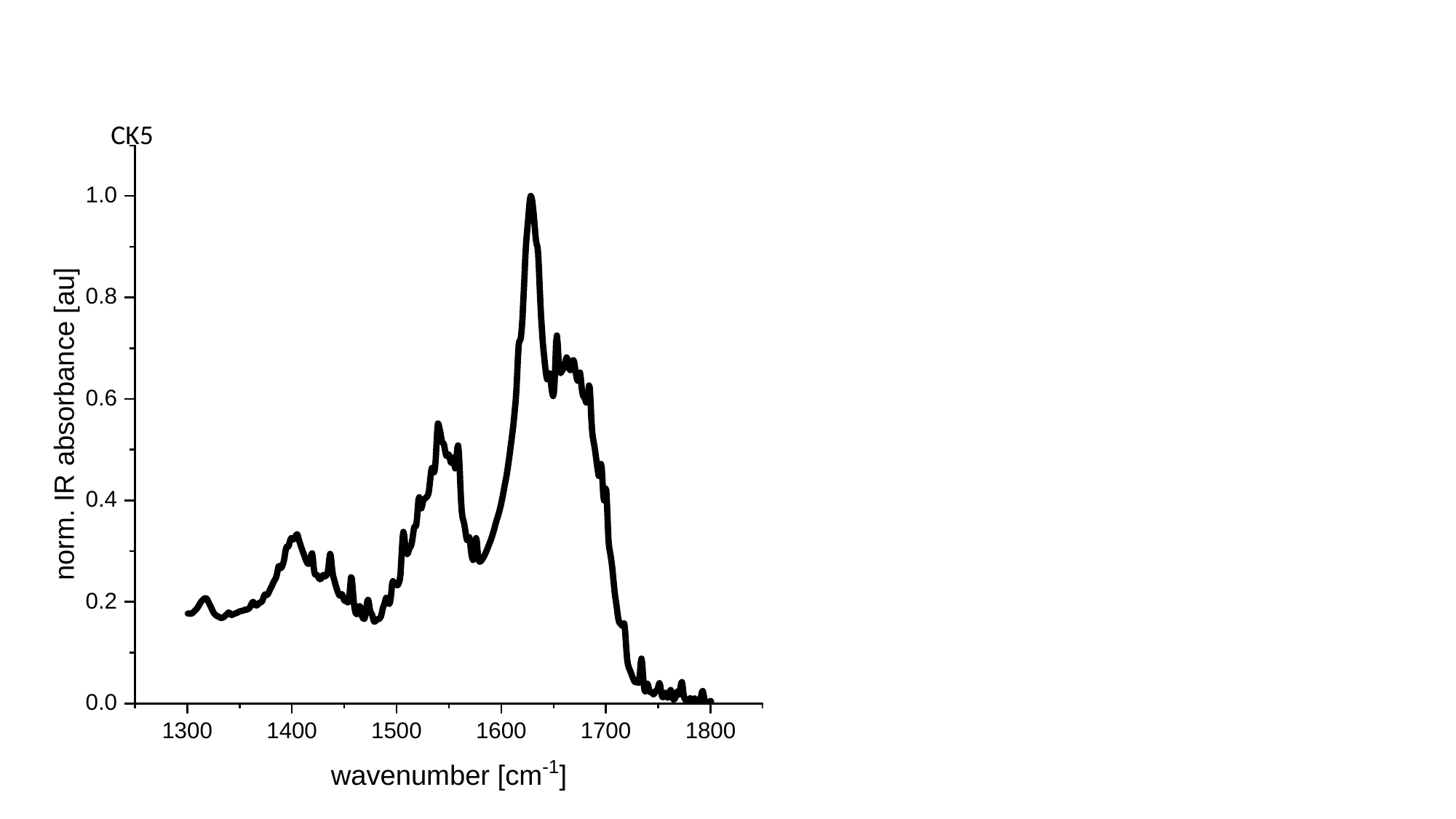

# CK5

## Slide 24
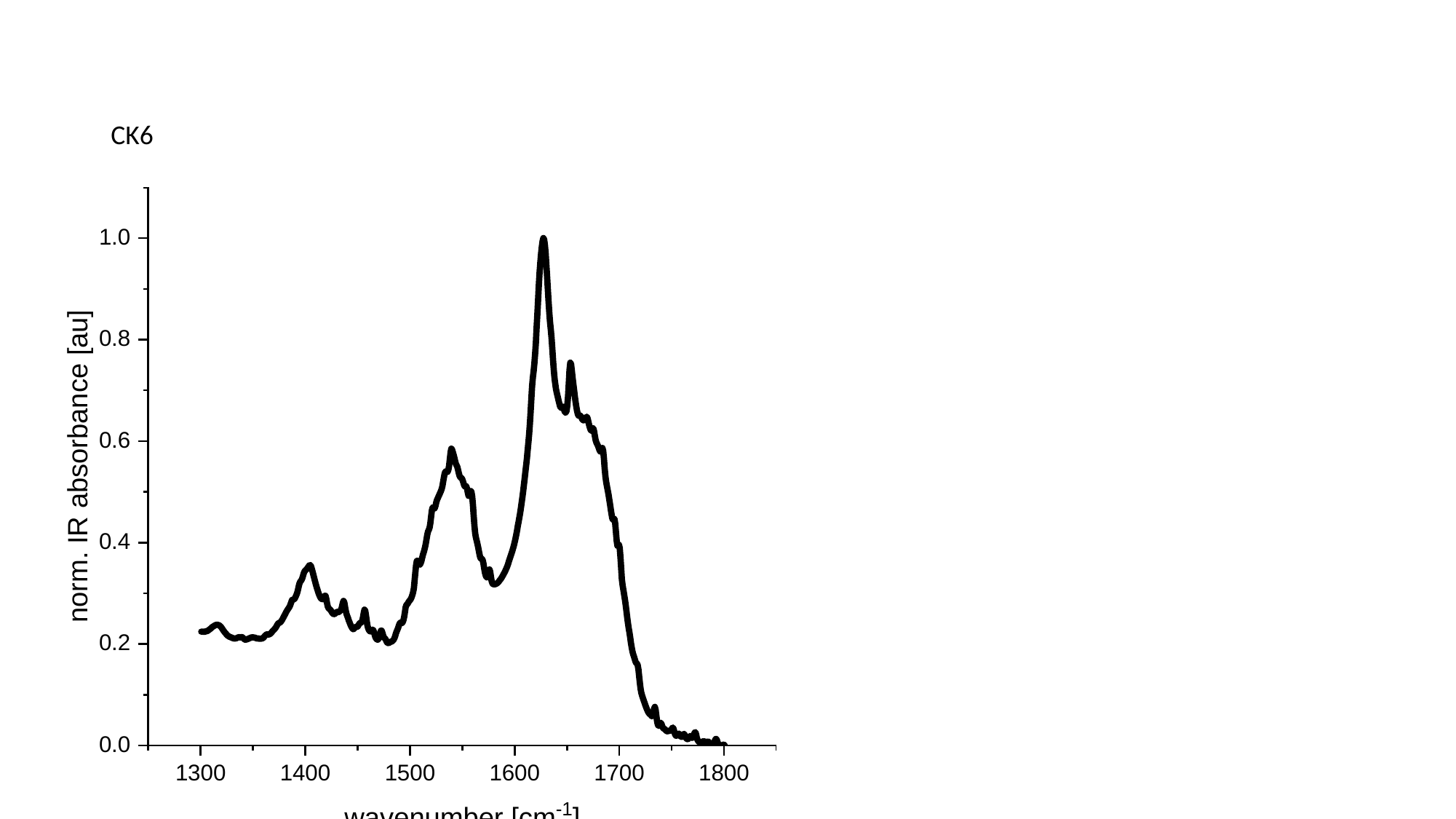

# CK6

## Slide 25
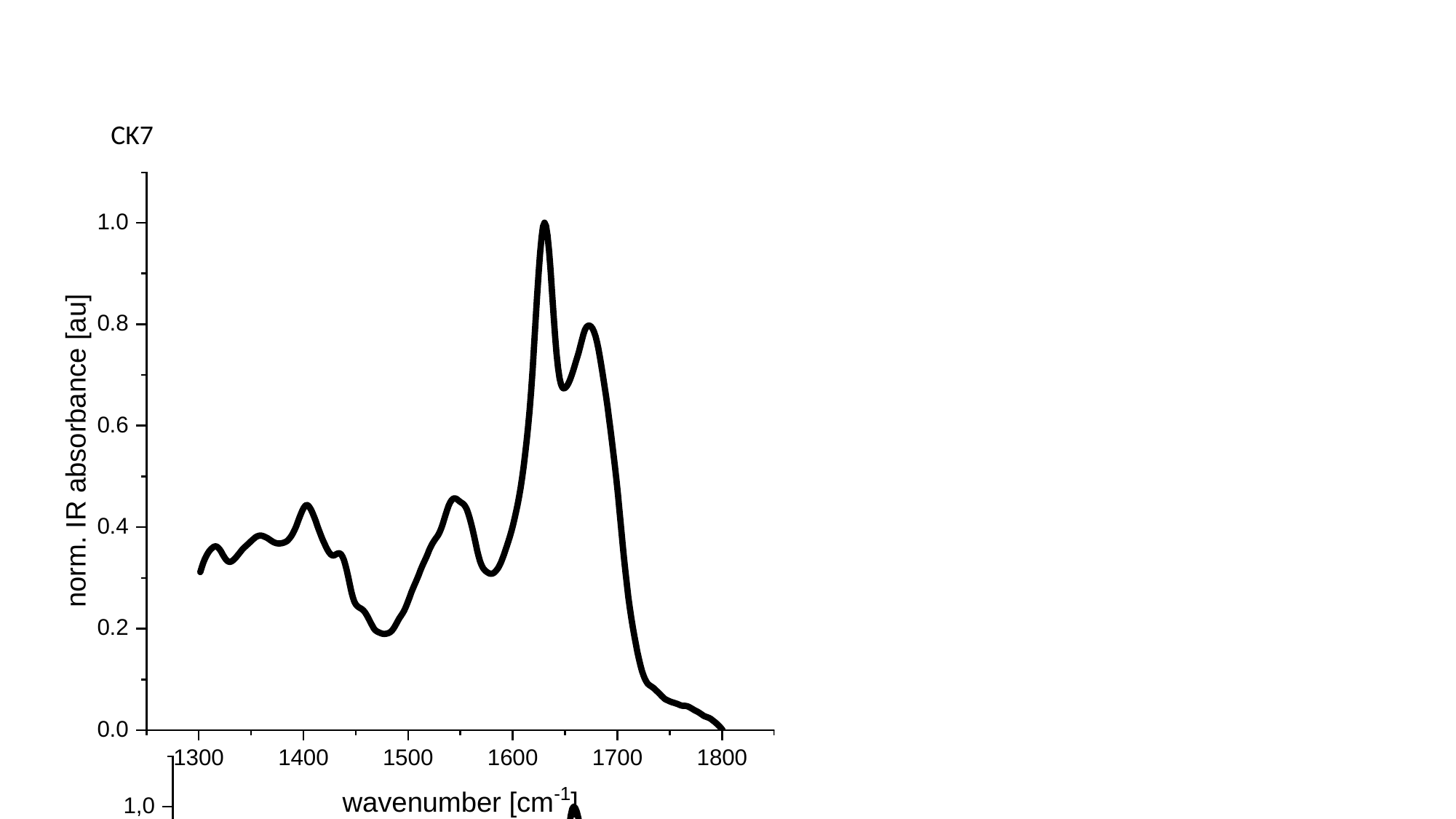

# CK7

## Slide 26
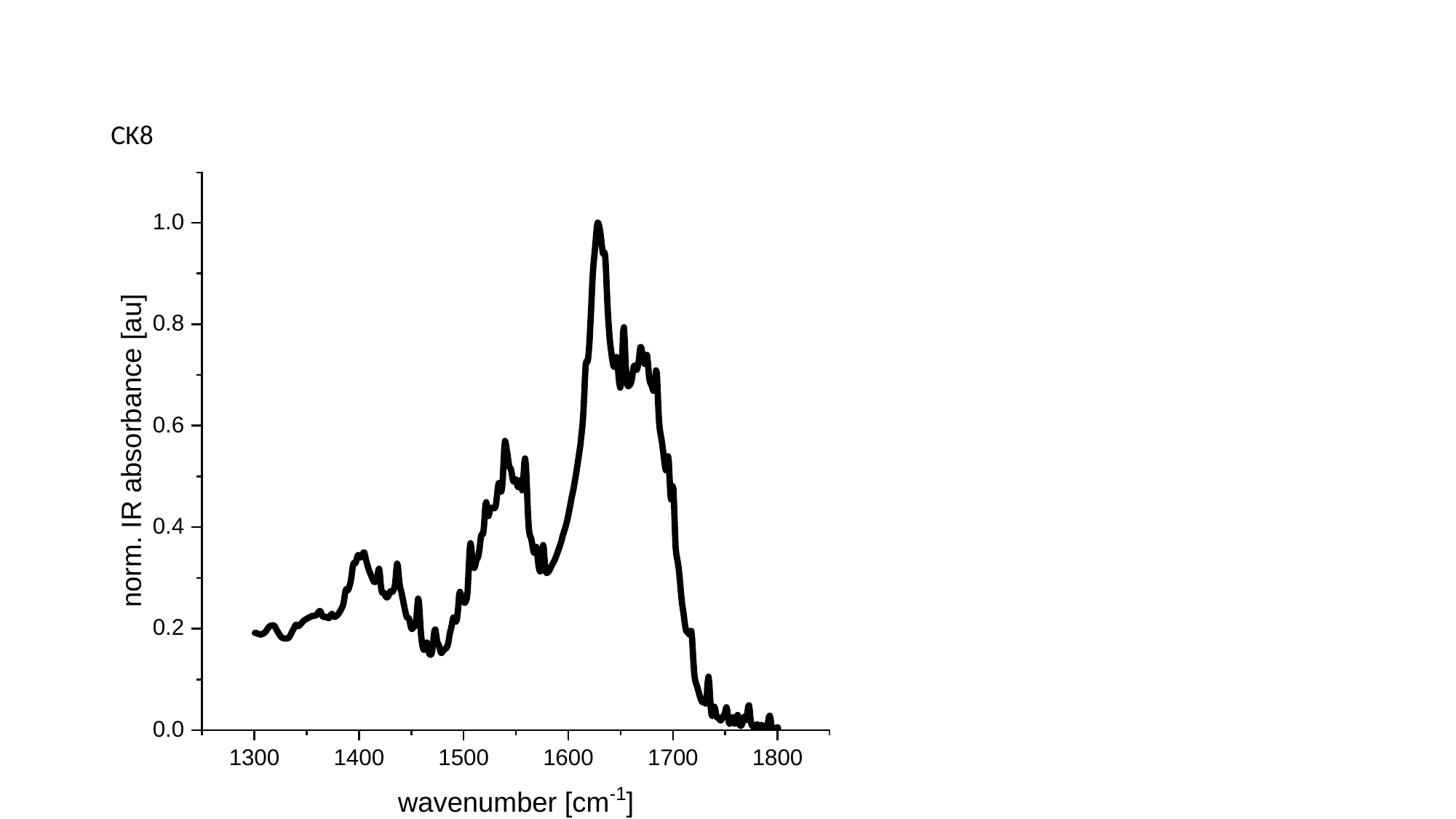

# CK8

## Slide 27
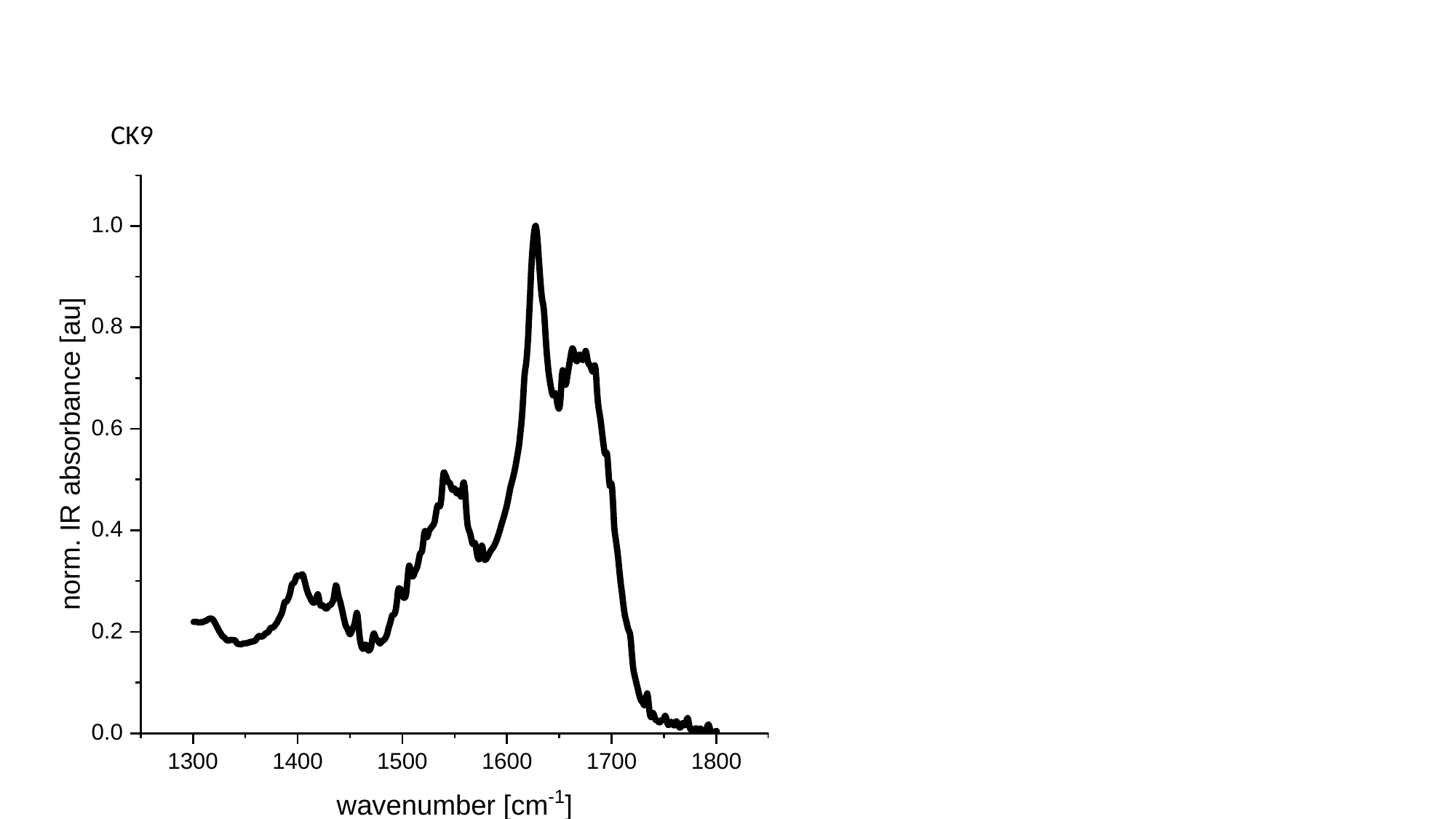

# CK9

## Slide 28
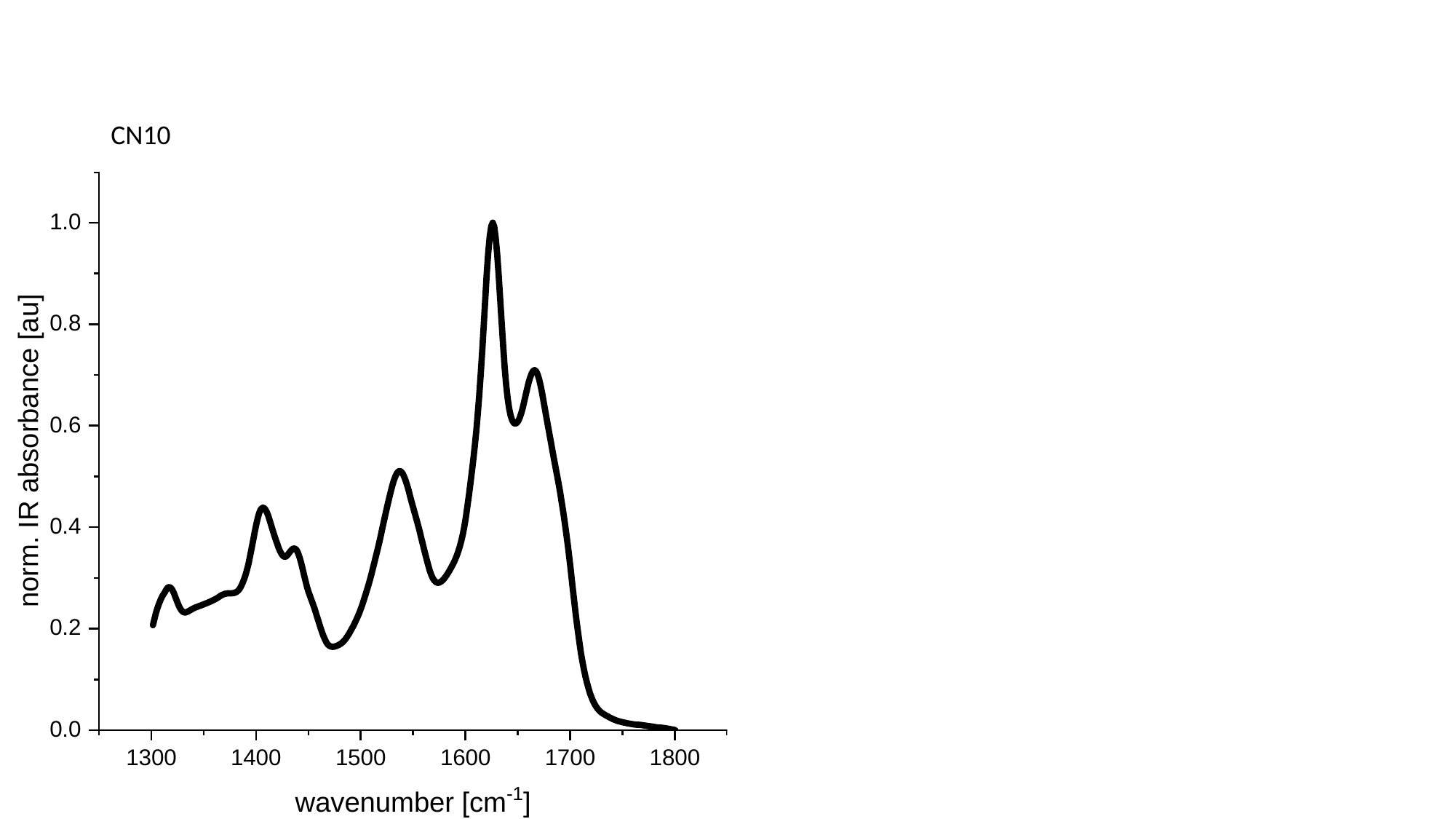

# CN10

## Slide 29
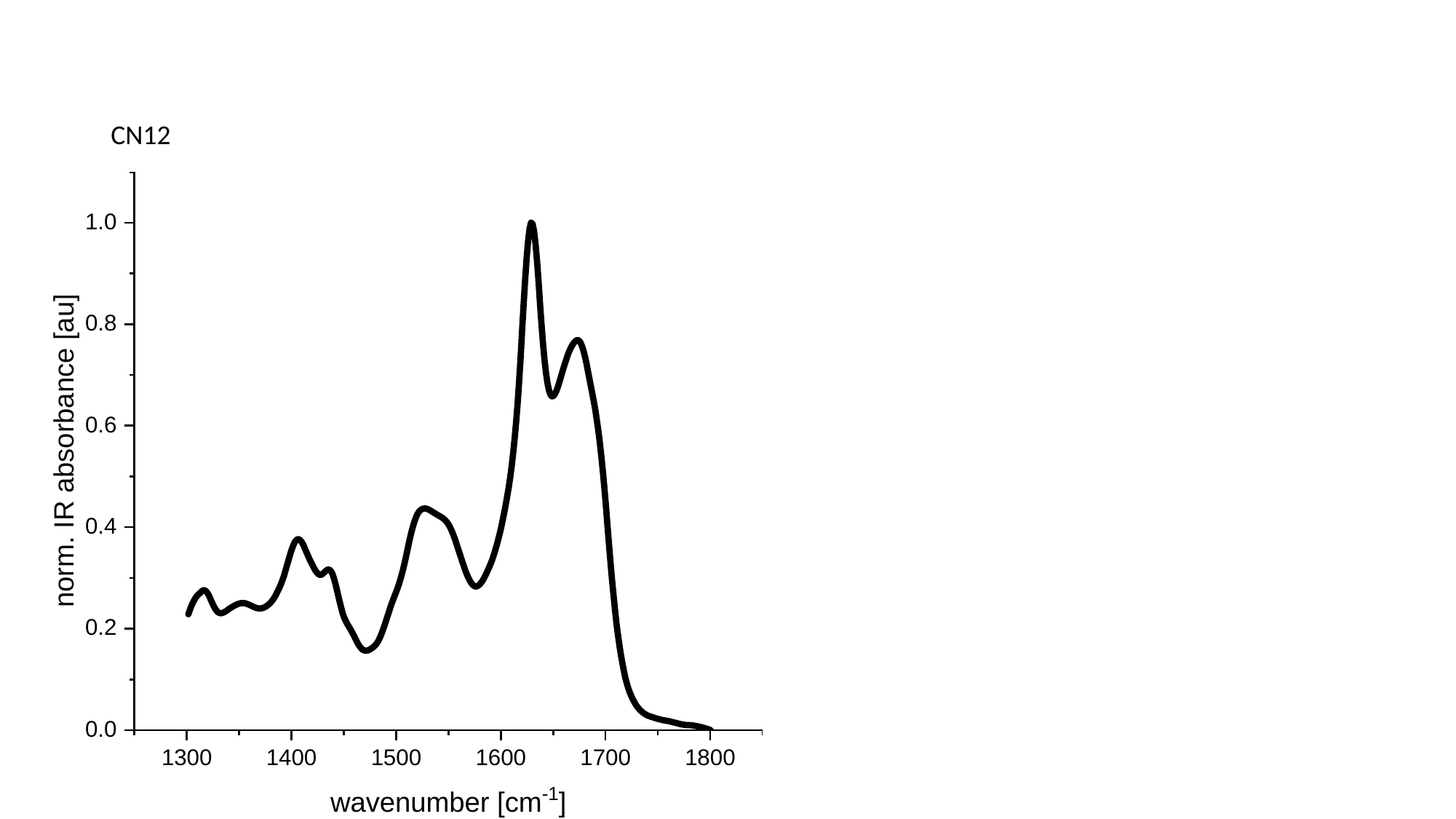

# CN12

## Slide 30
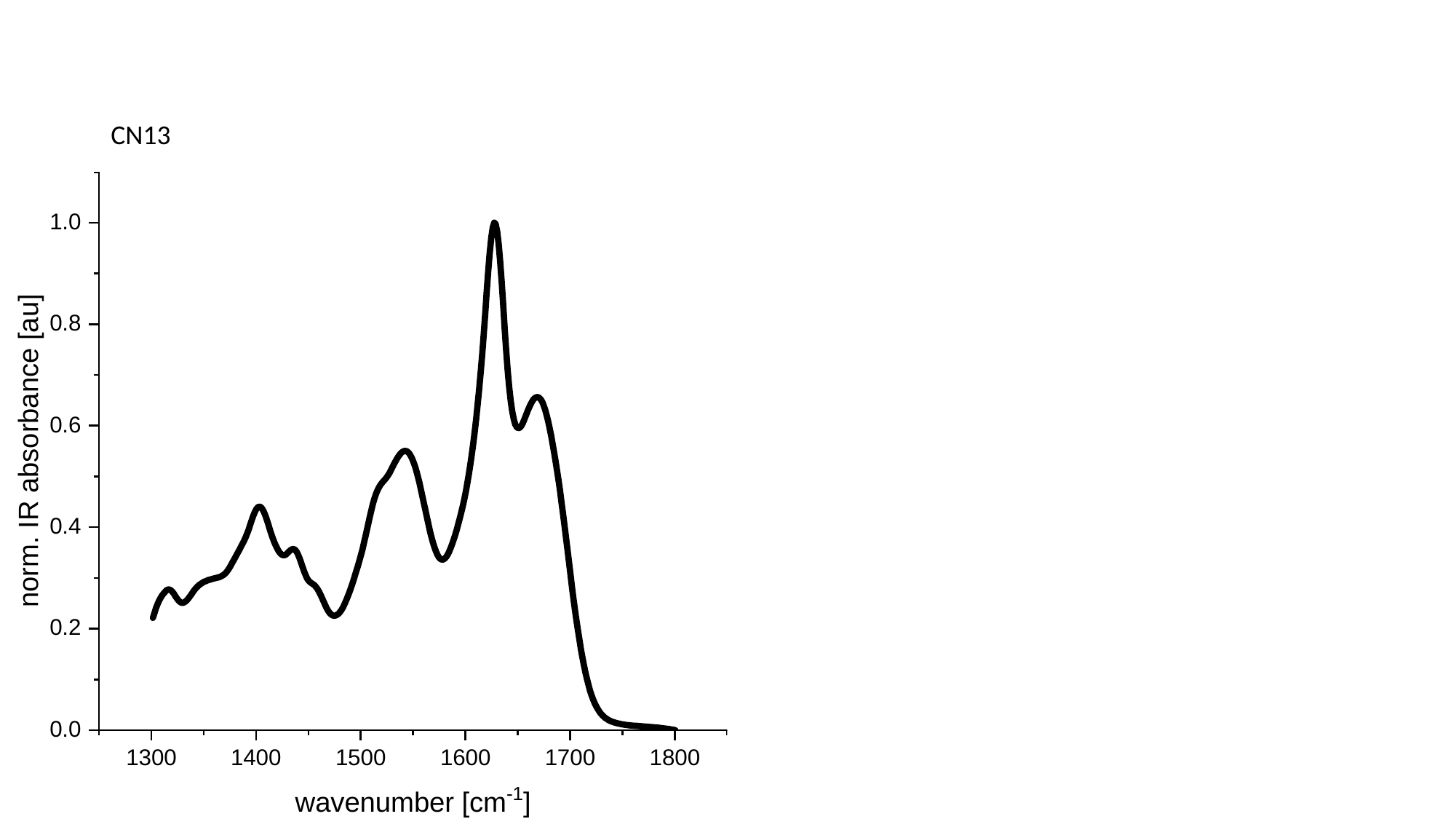

# CN13

## Slide 31
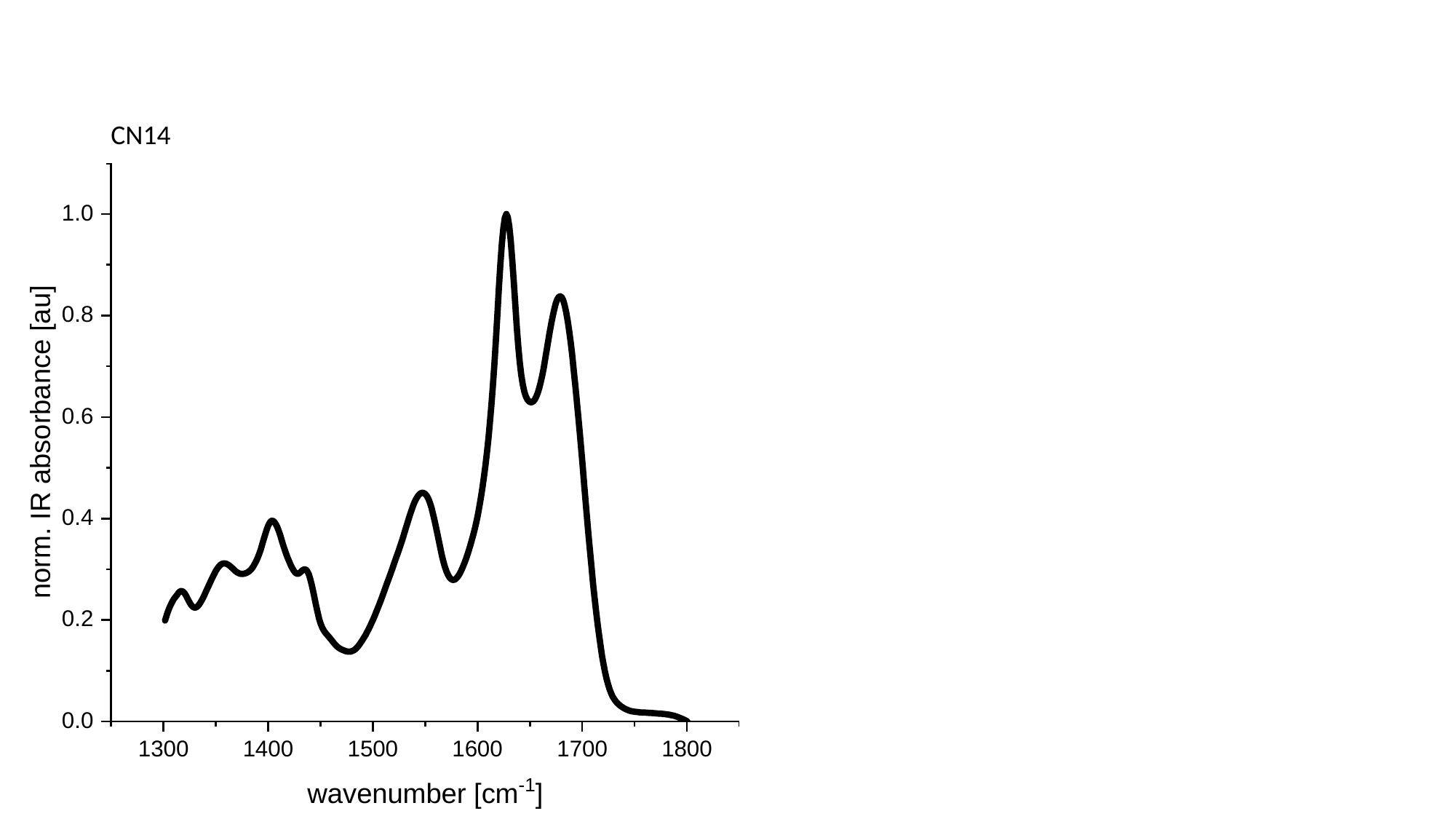

# CN14

## Slide 32
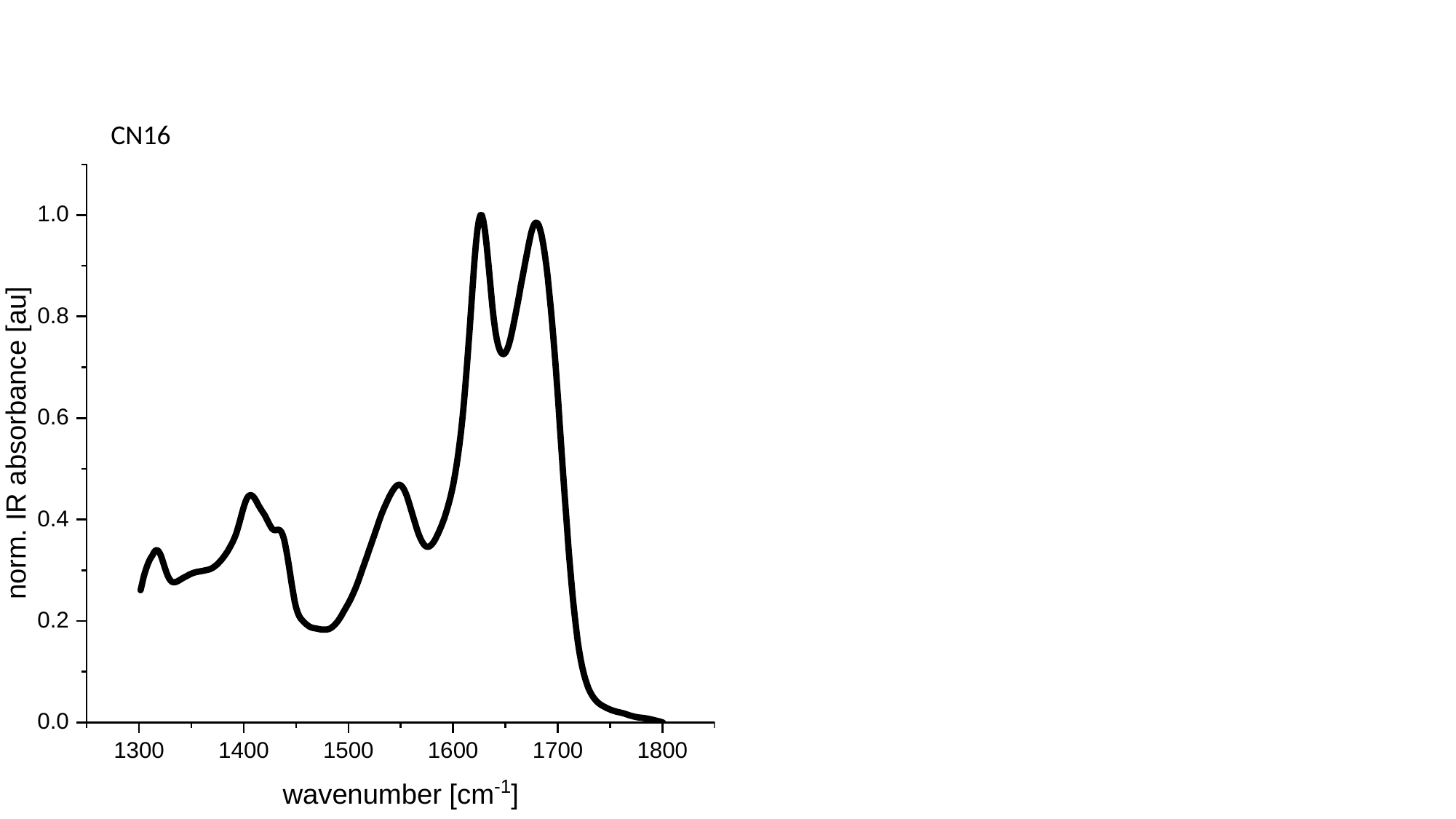

# CN16

## Slide 33
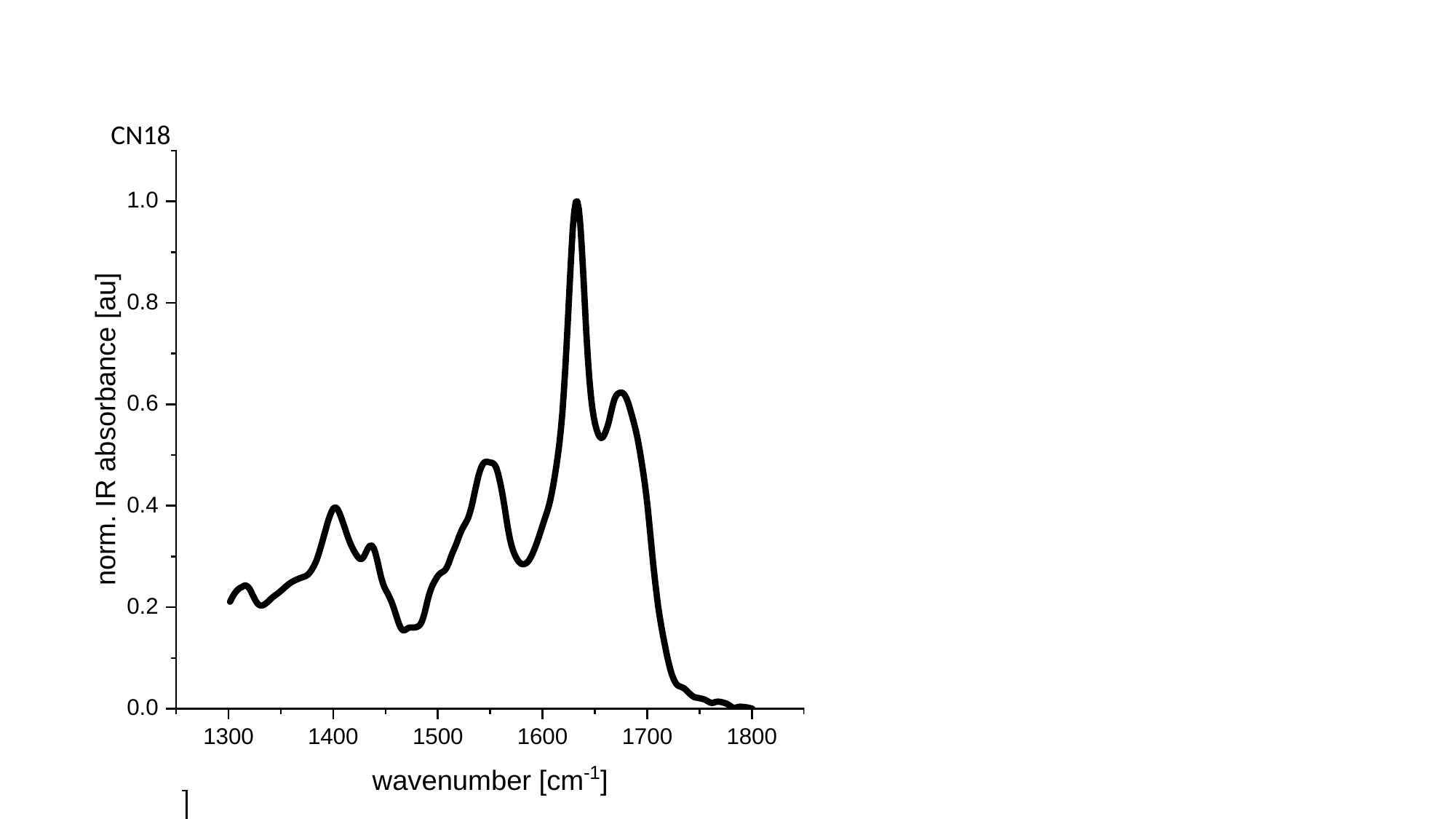

# CN18

## Slide 34
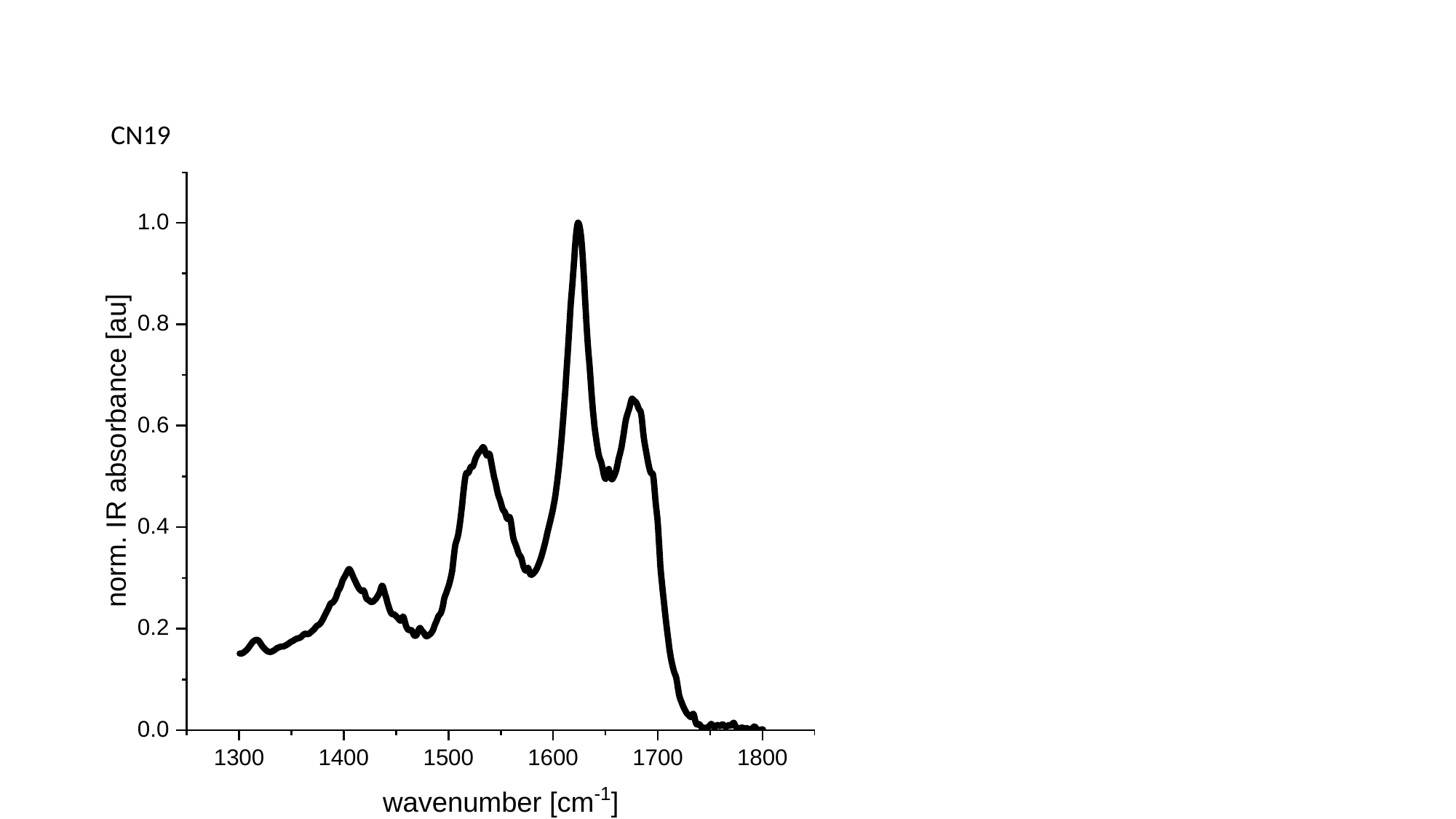

# CN19

## Slide 35
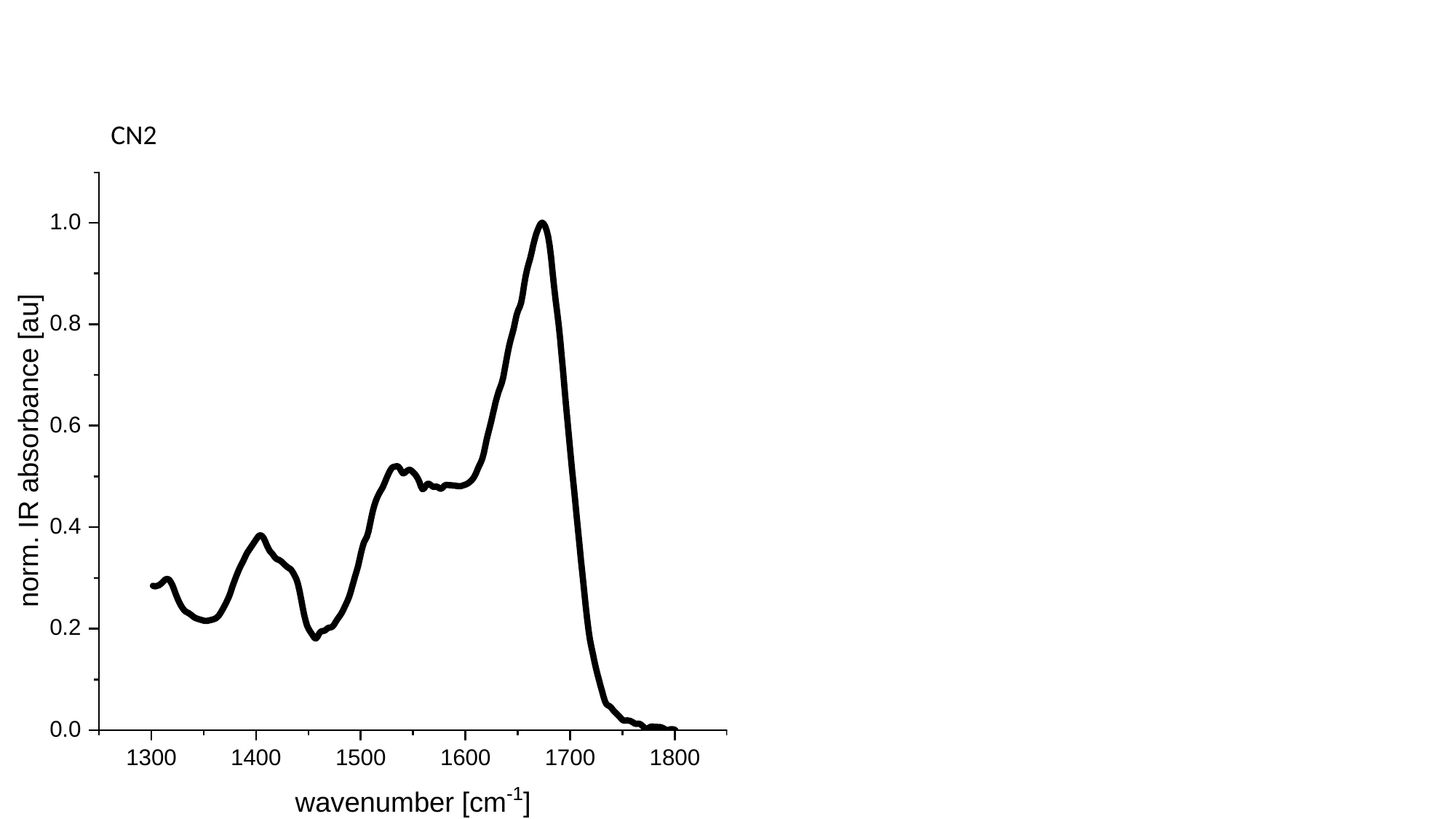

# CN2

## Slide 36
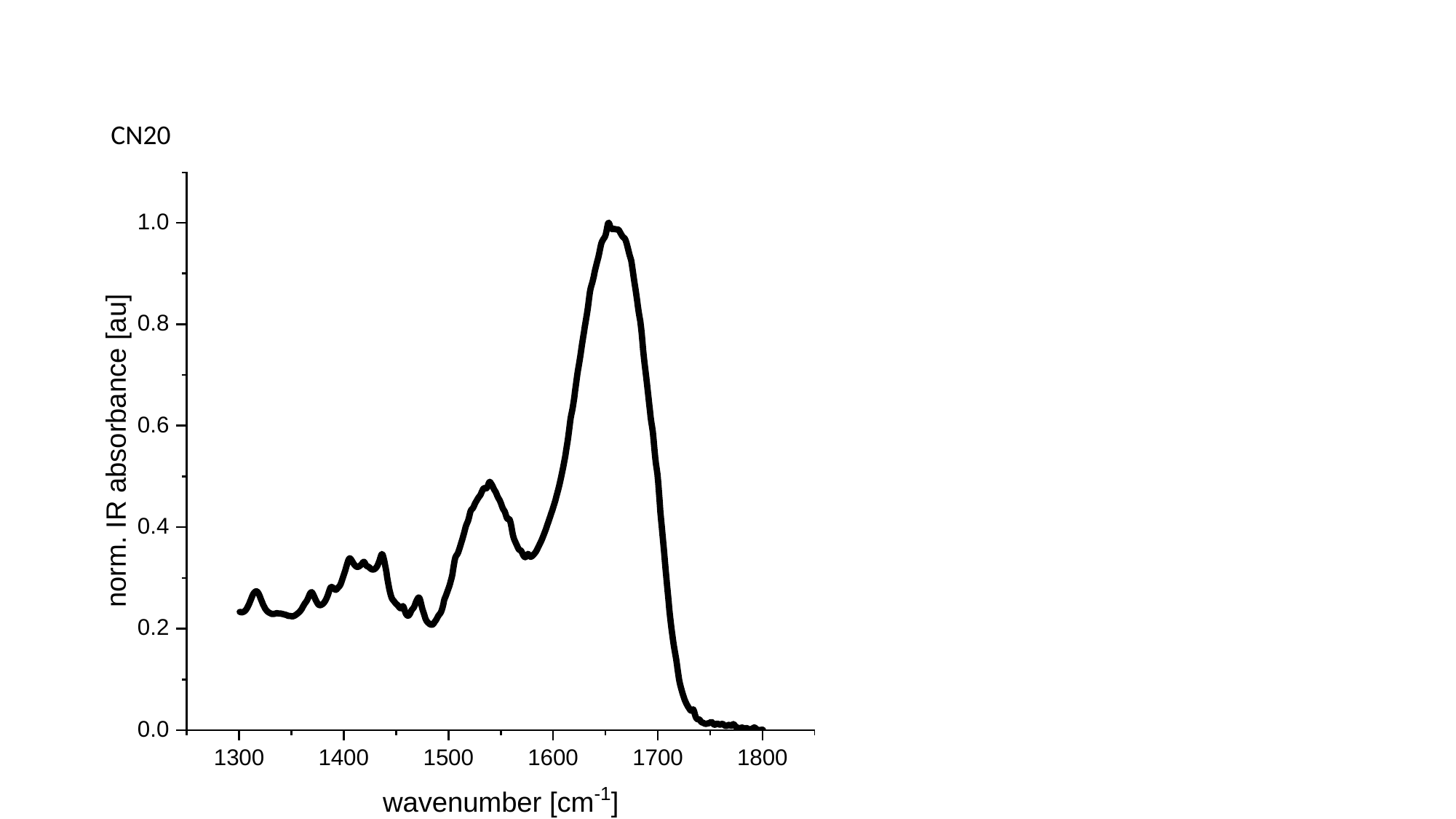

# CN20

## Slide 37
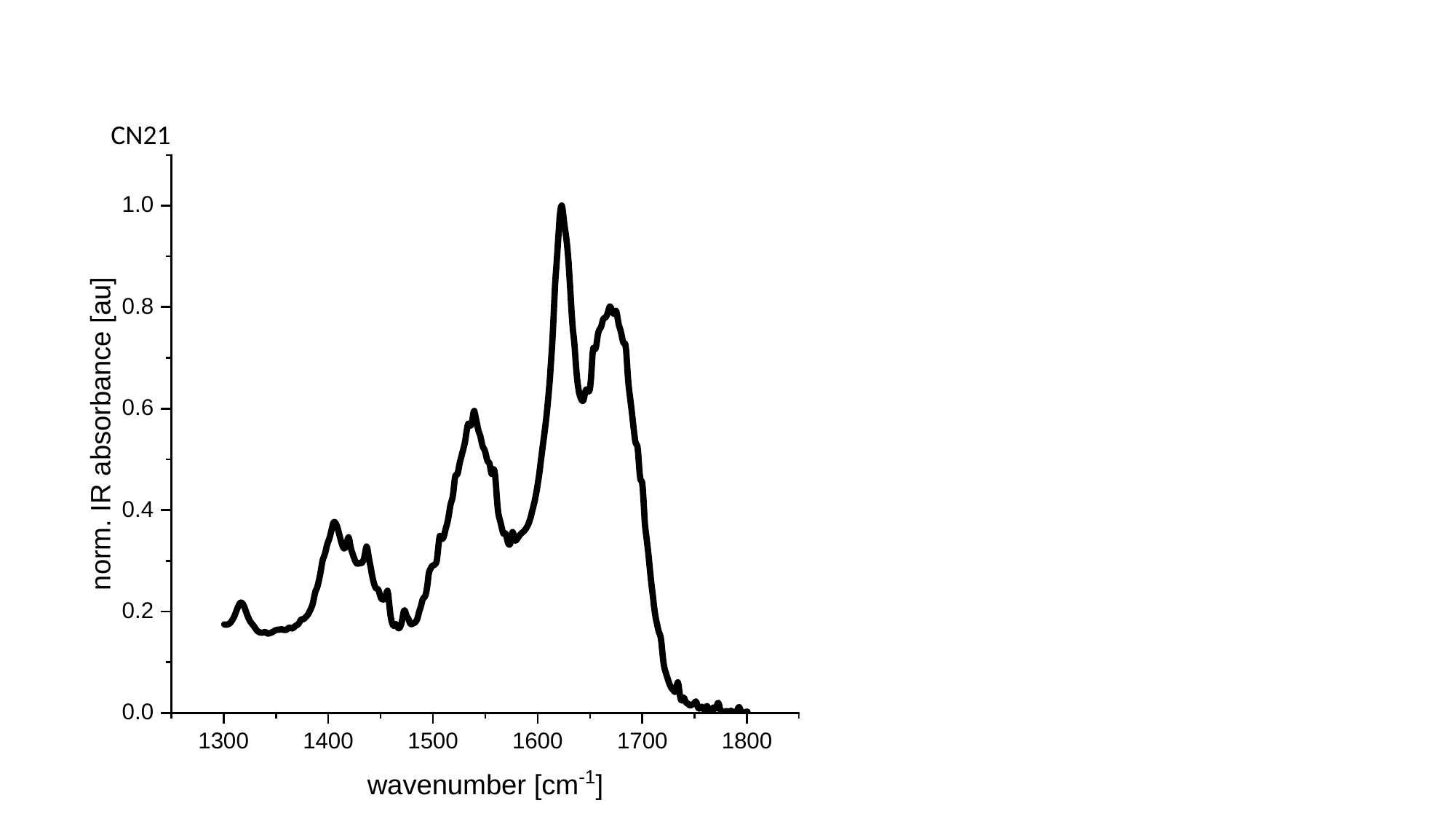

# CN21

## Slide 38
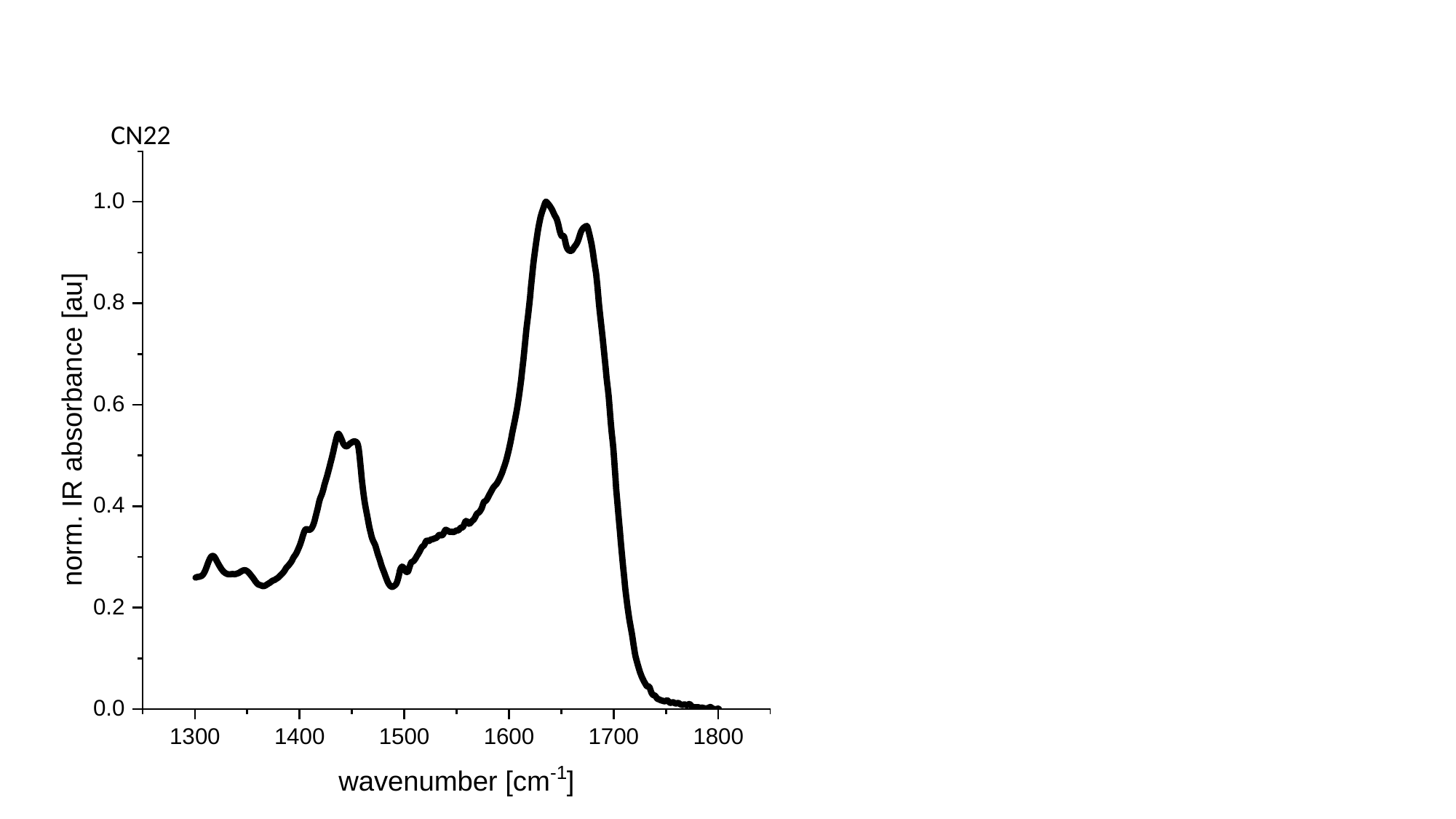

# CN22

## Slide 39
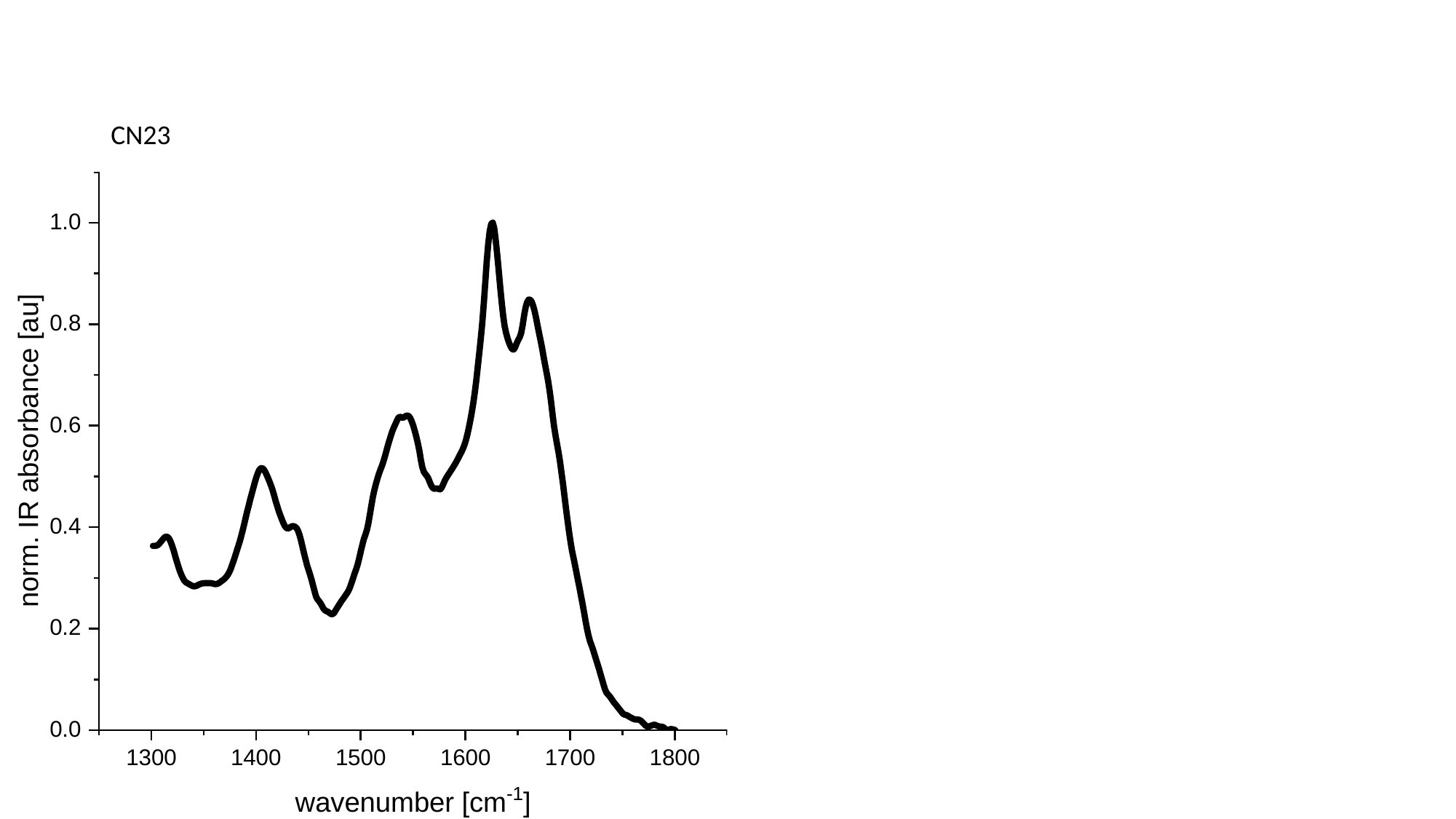

# CN23

## Slide 40
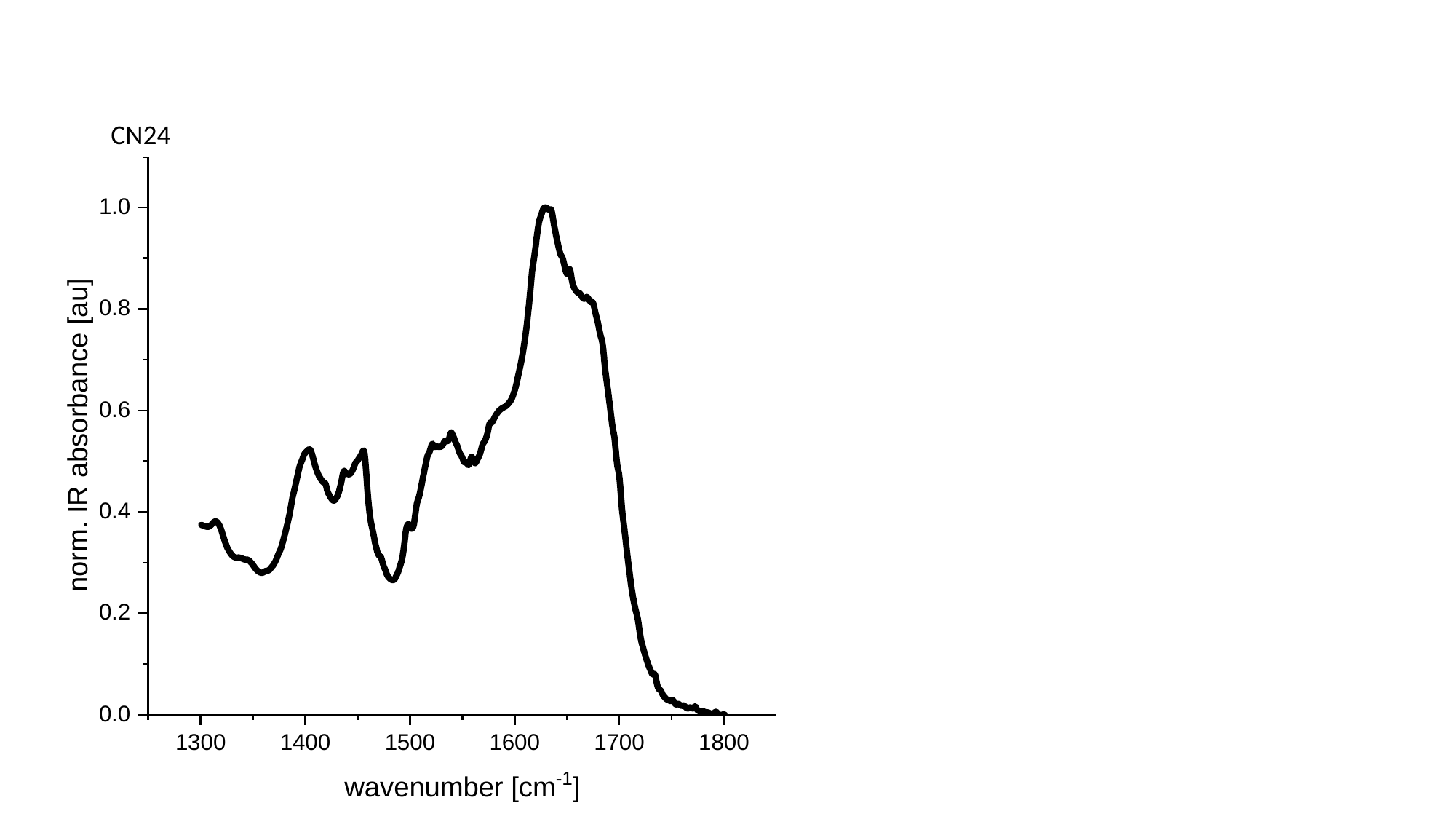

# CN24

## Slide 41
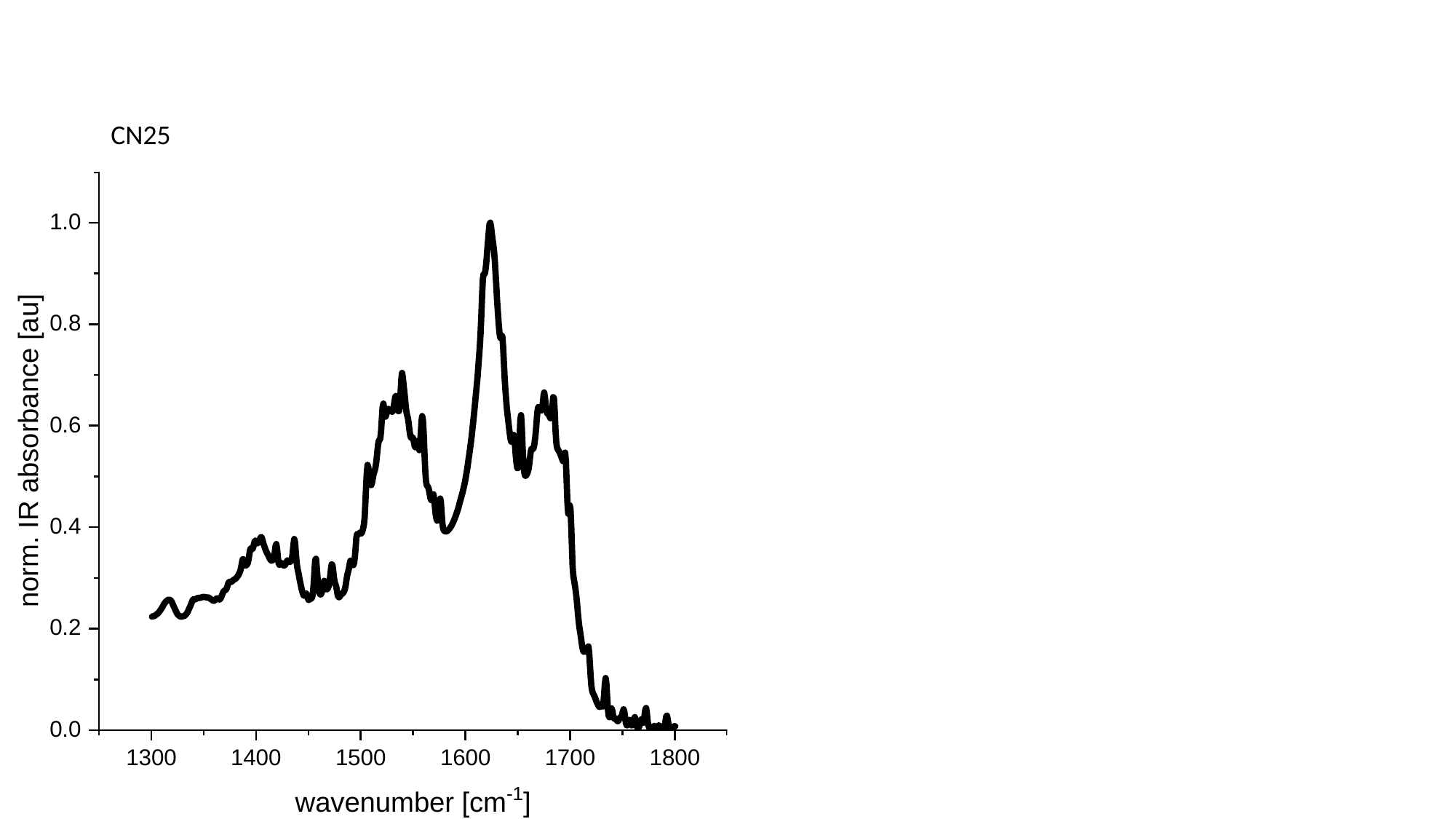

# CN25

## Slide 42
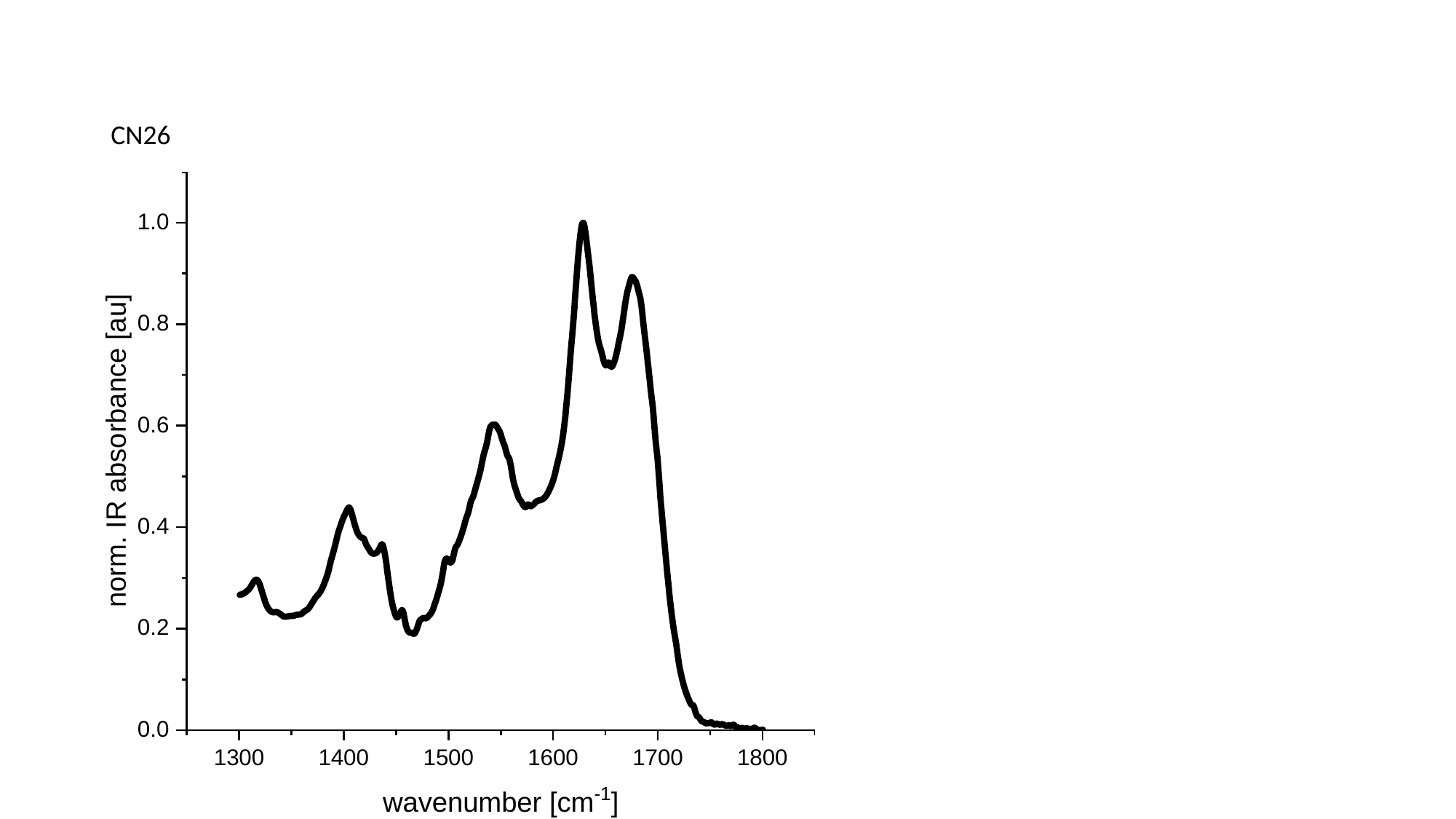

# CN26

## Slide 43
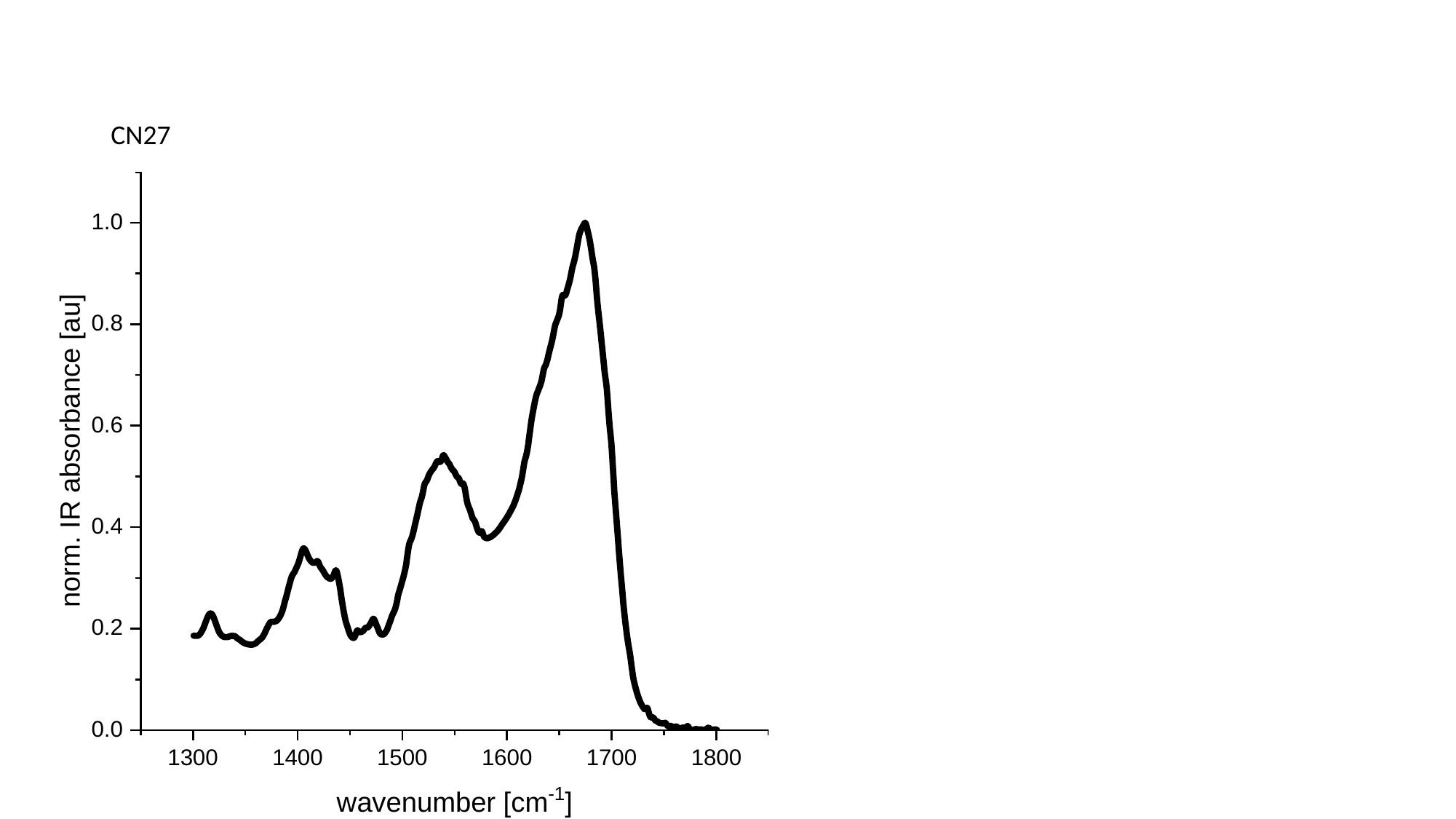

# CN27

## Slide 44
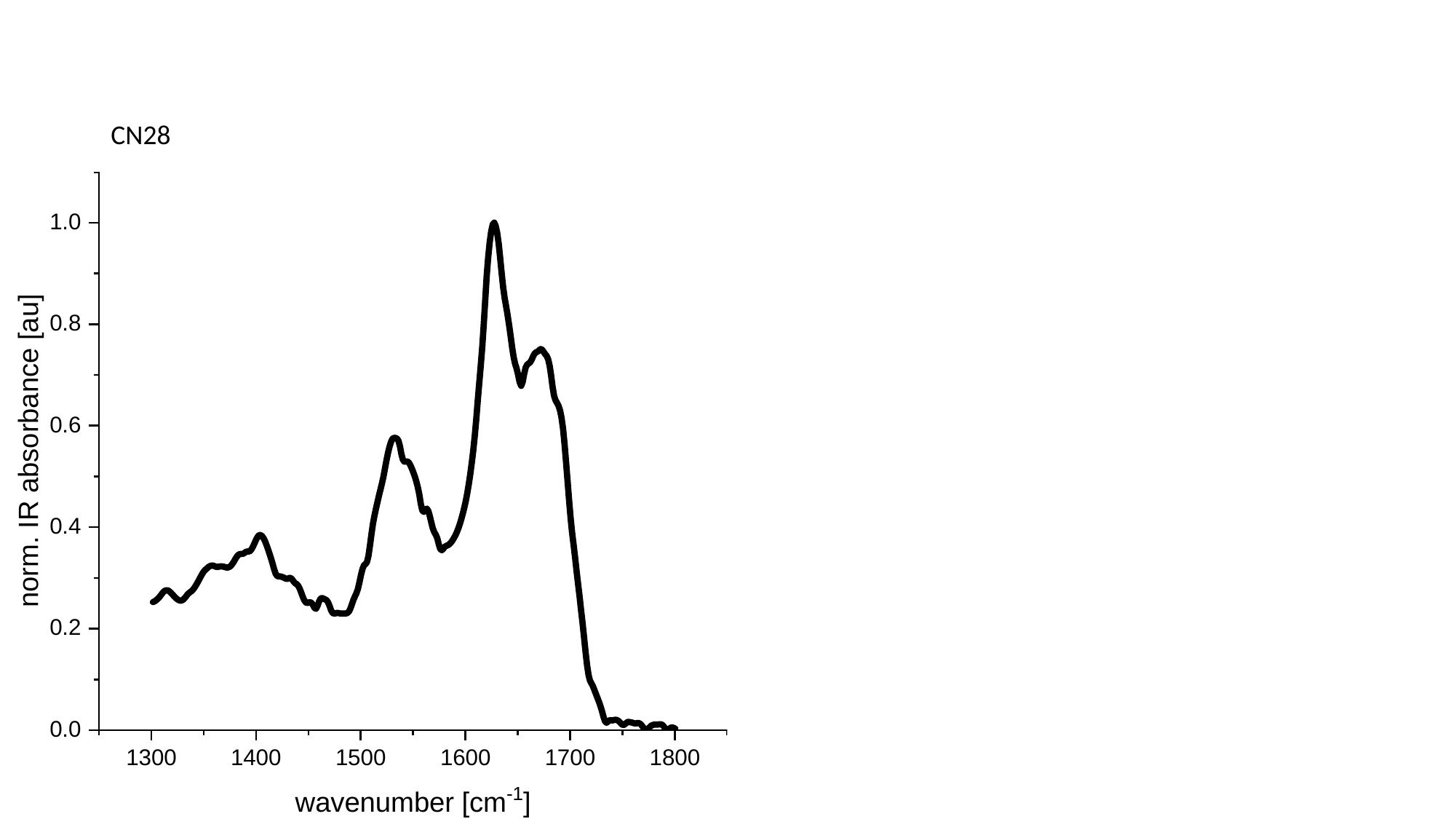

# CN28

## Slide 45
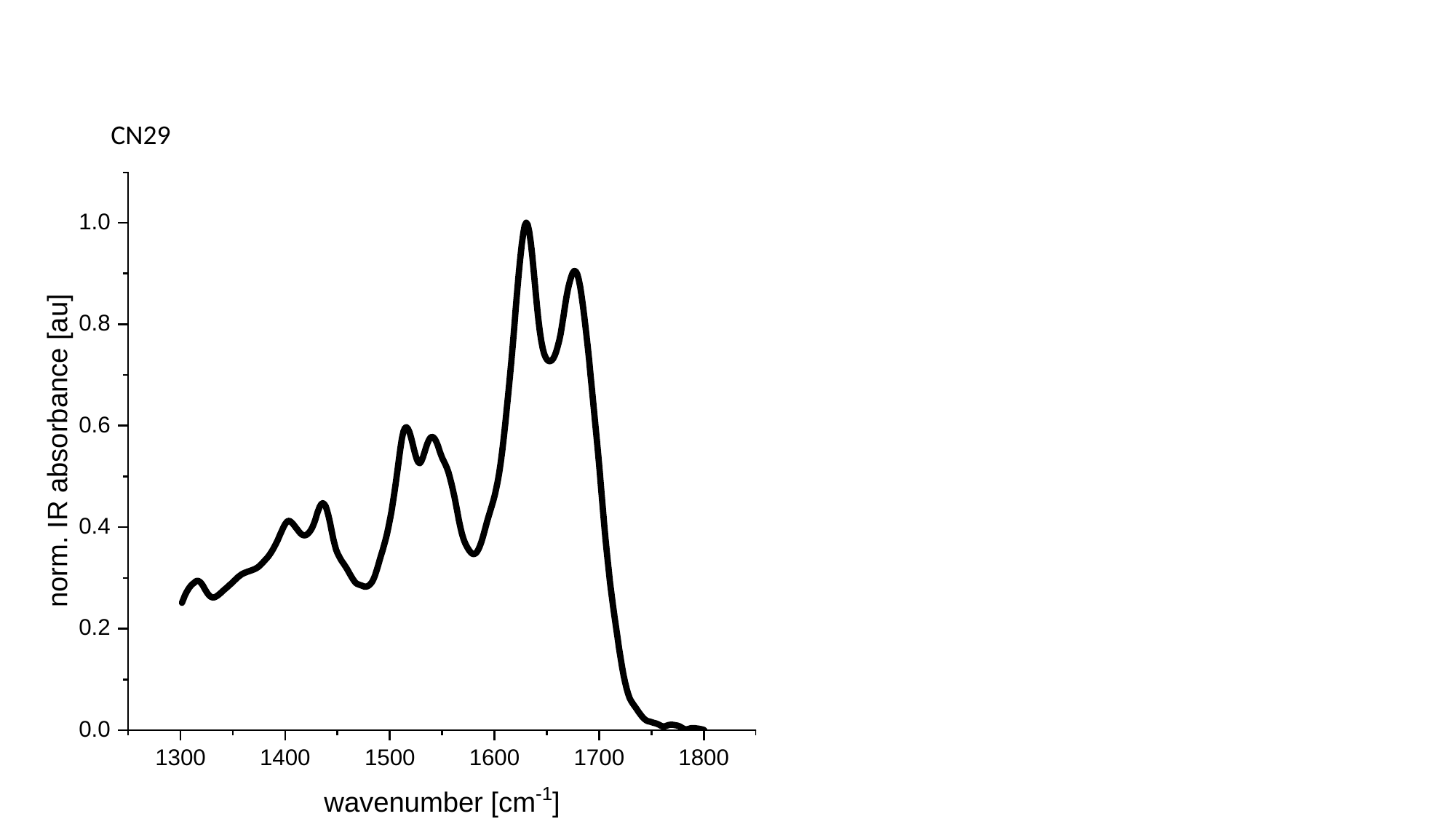

# CN29

## Slide 46
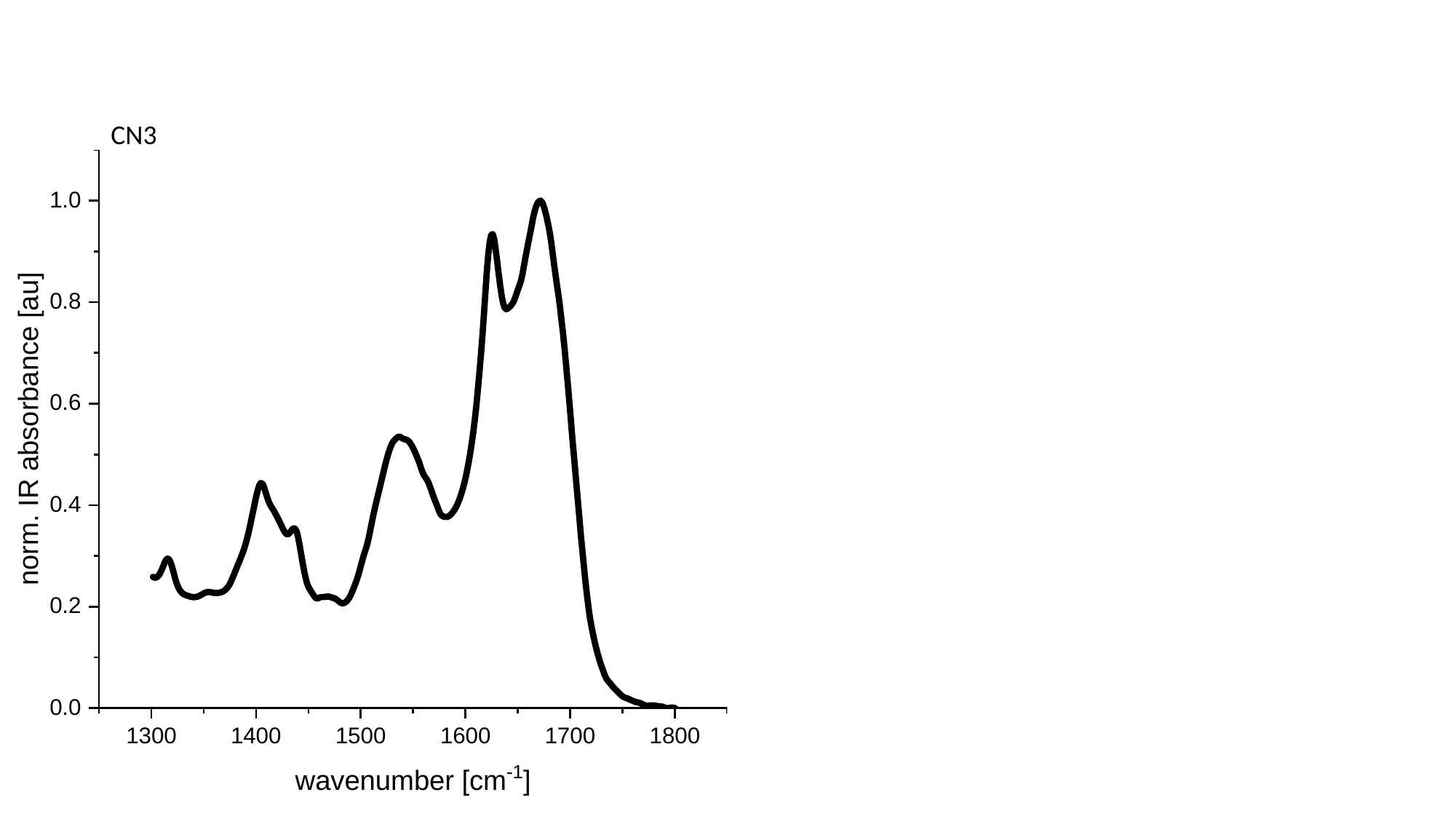

# CN3

## Slide 47
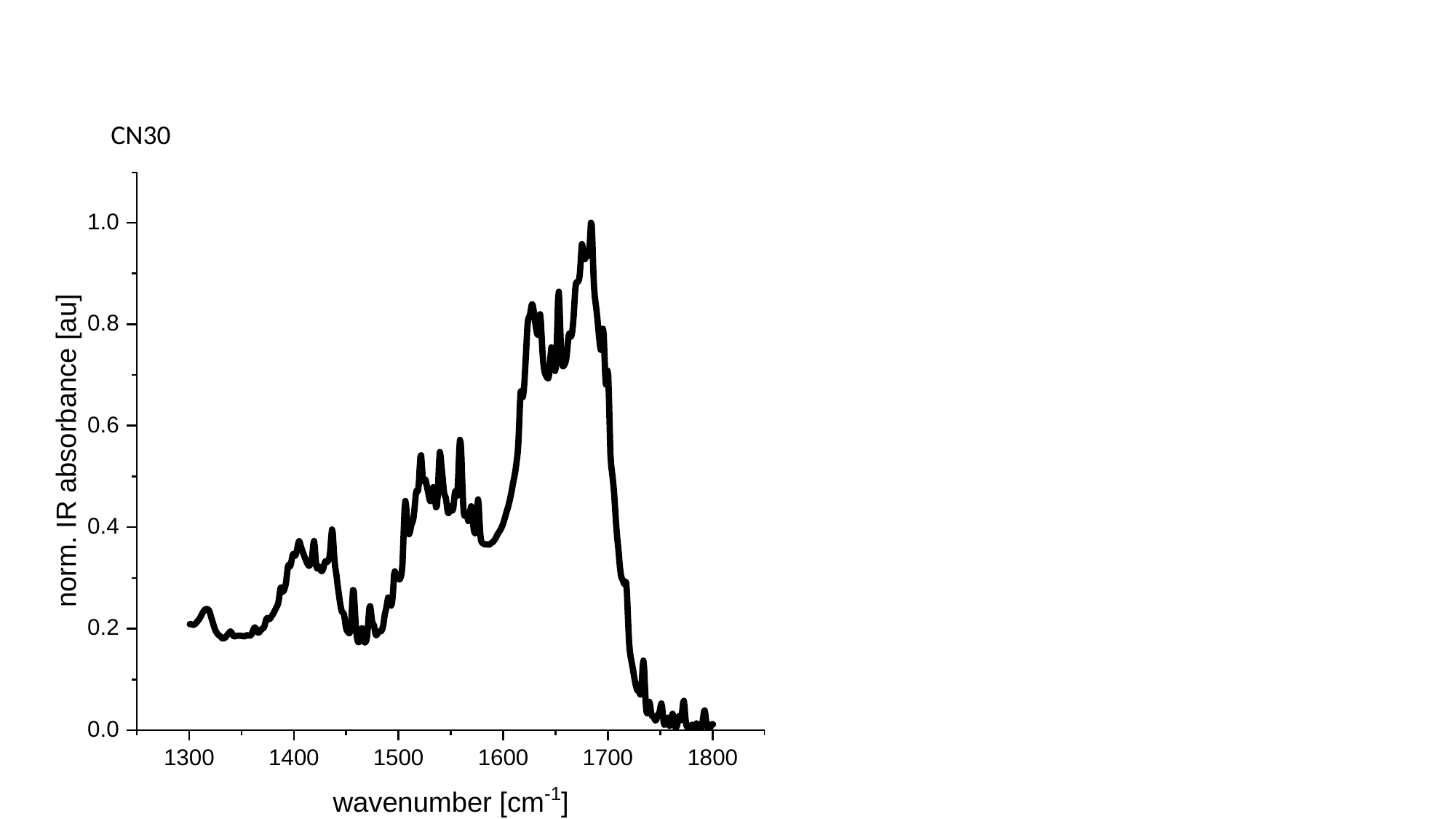

# CN30

## Slide 48
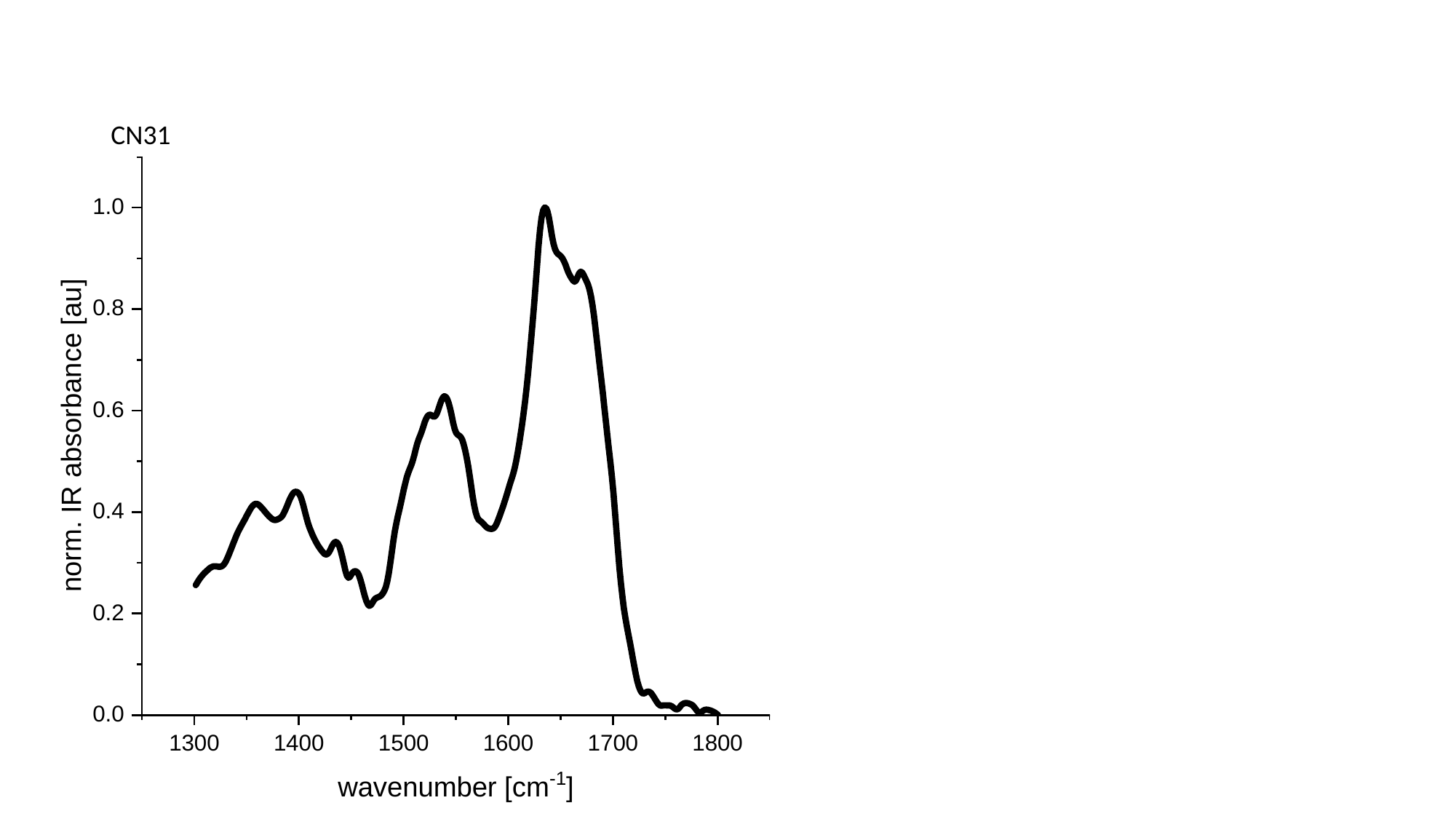

# CN31

## Slide 49
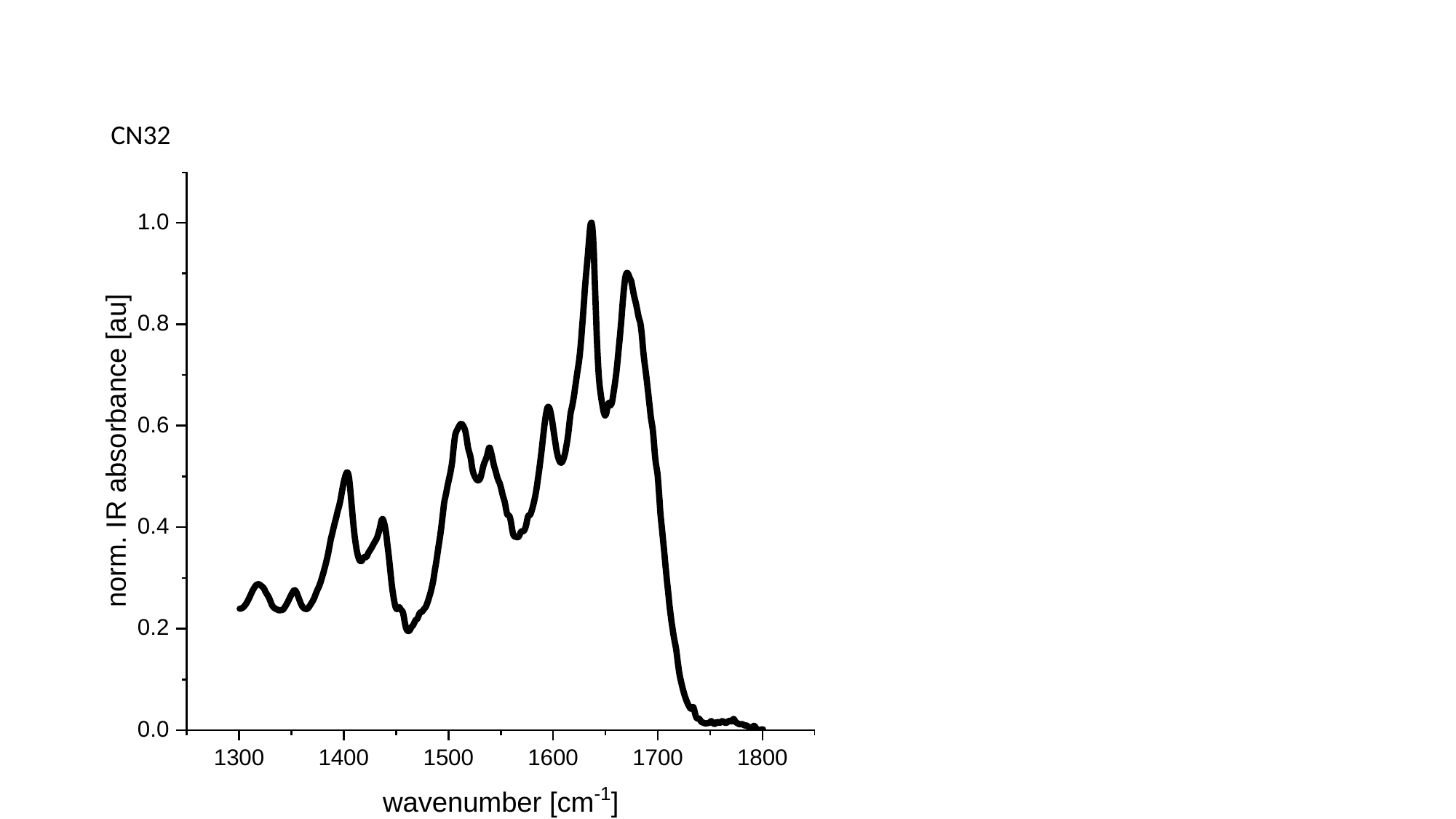

# CN32

## Slide 50
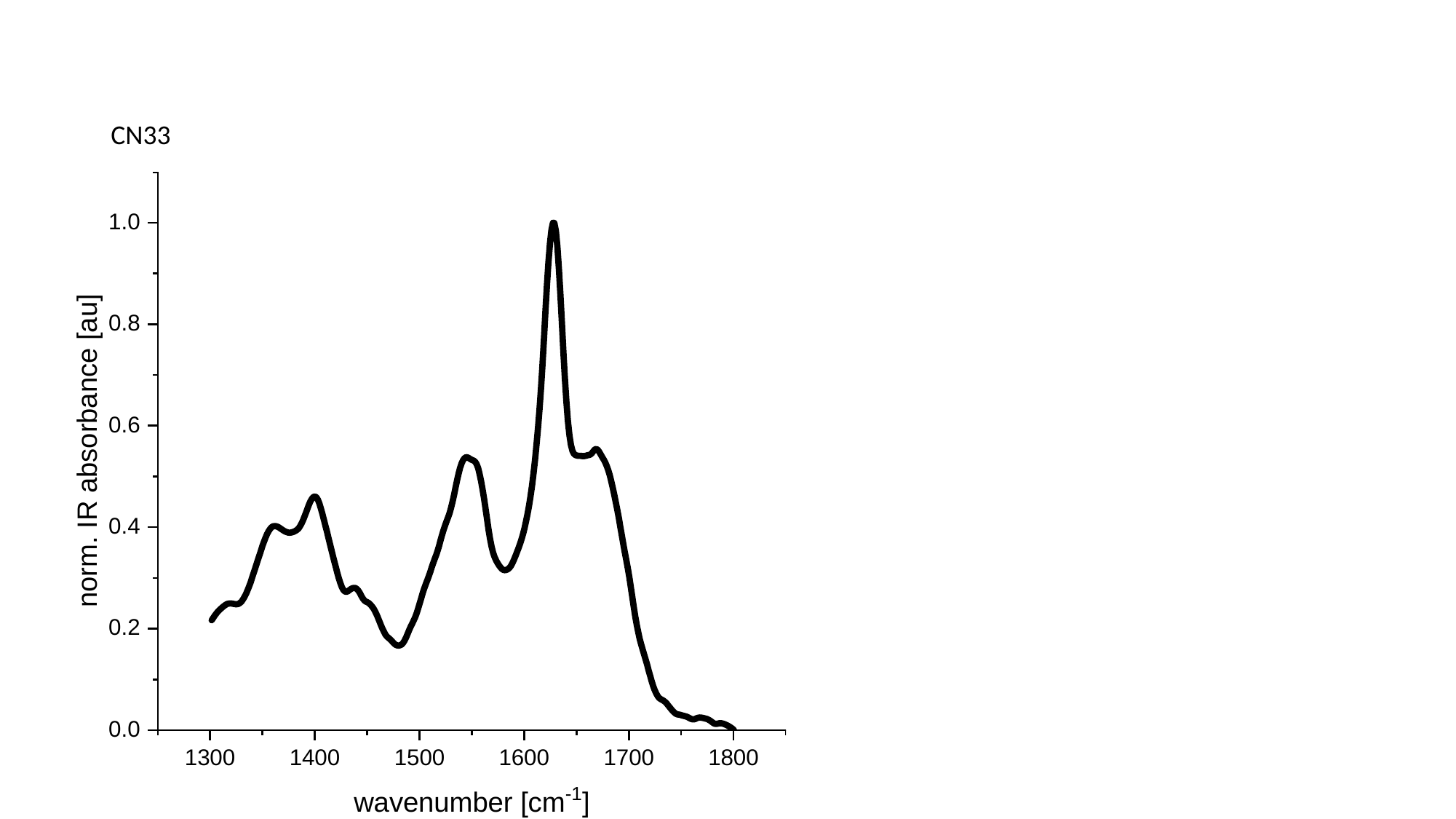

# CN33

## Slide 51
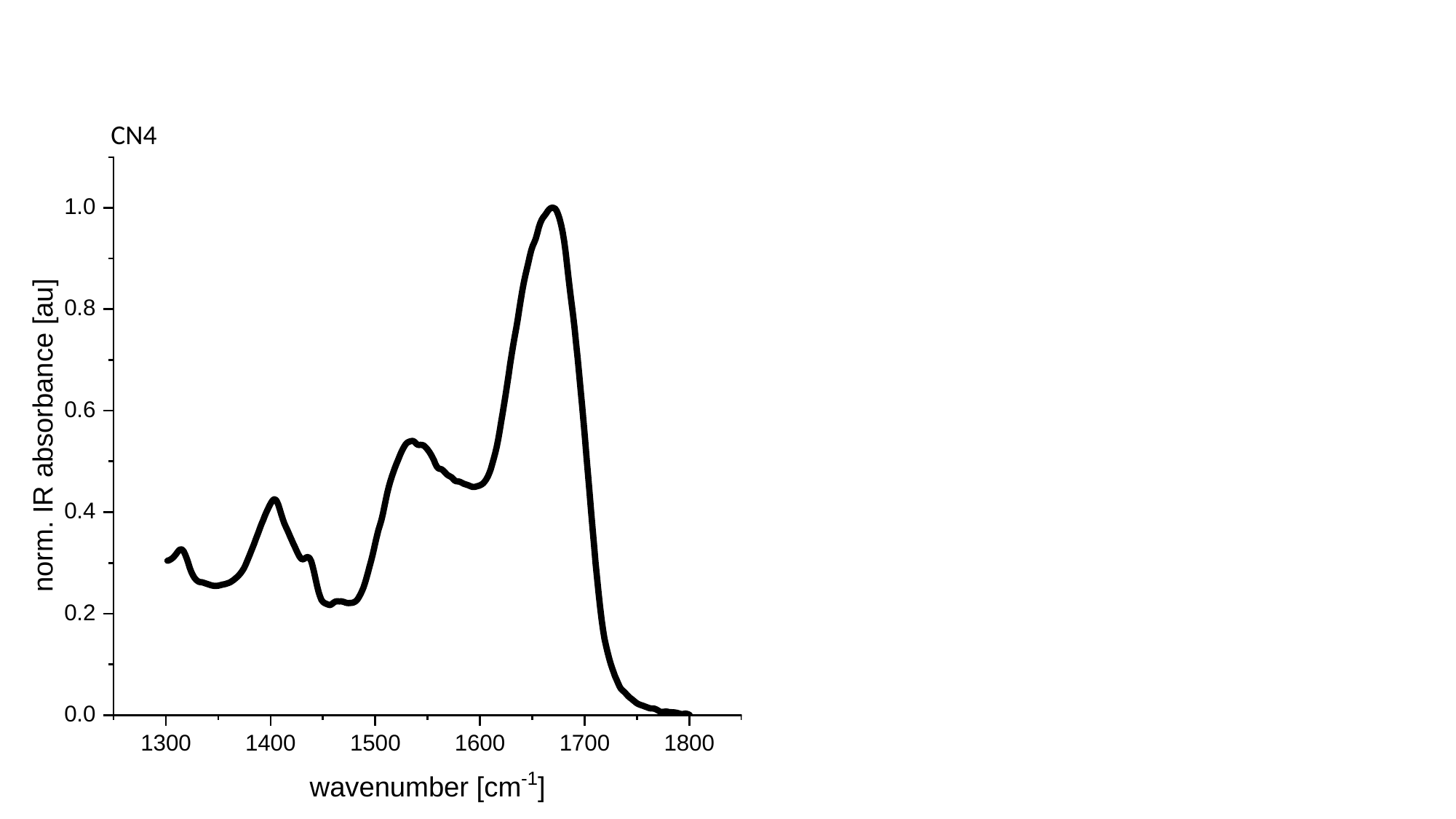

# CN4

## Slide 52
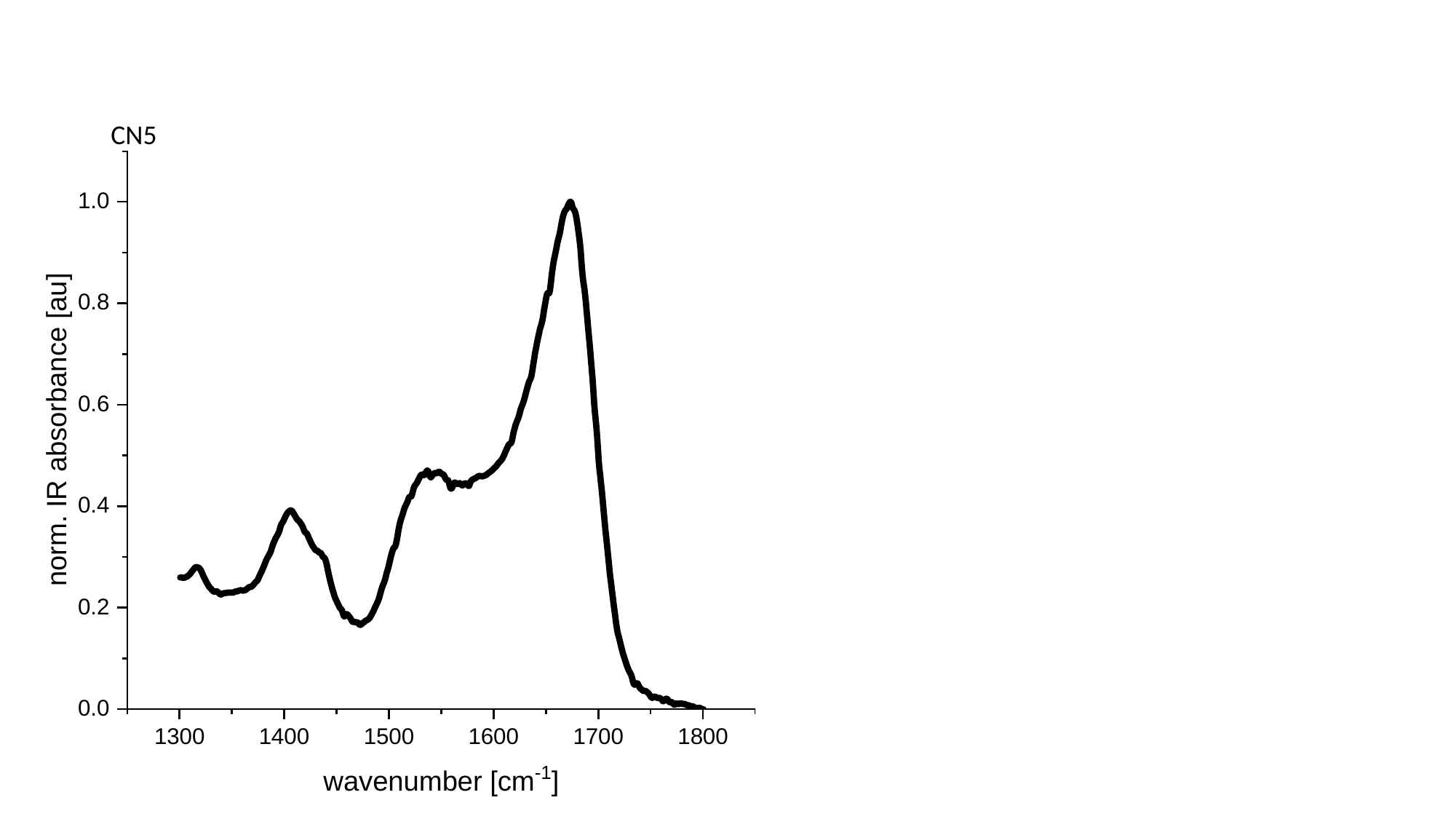

# CN5

## Slide 53
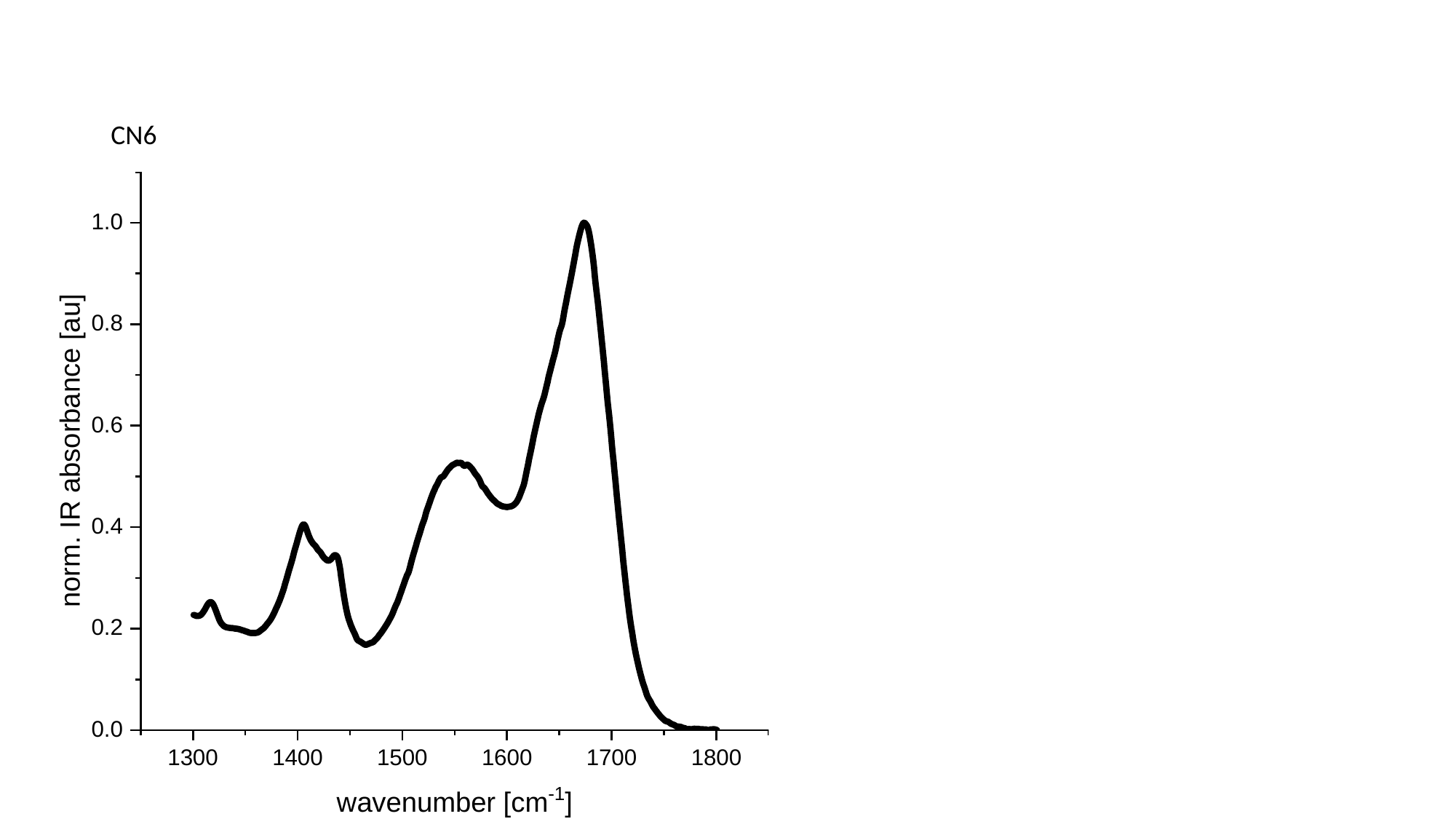

# CN6

## Slide 54
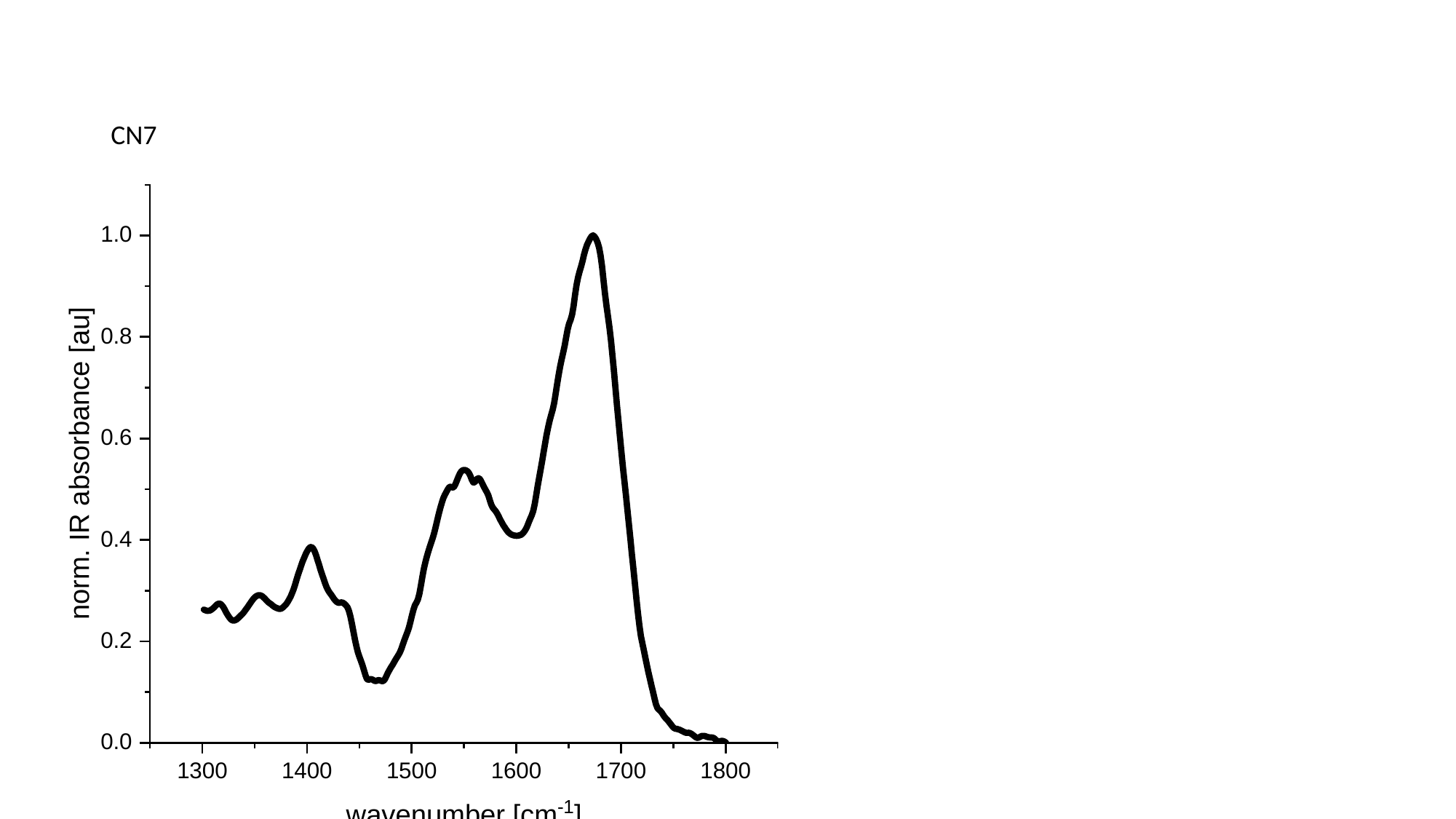

# CN7

## Slide 55
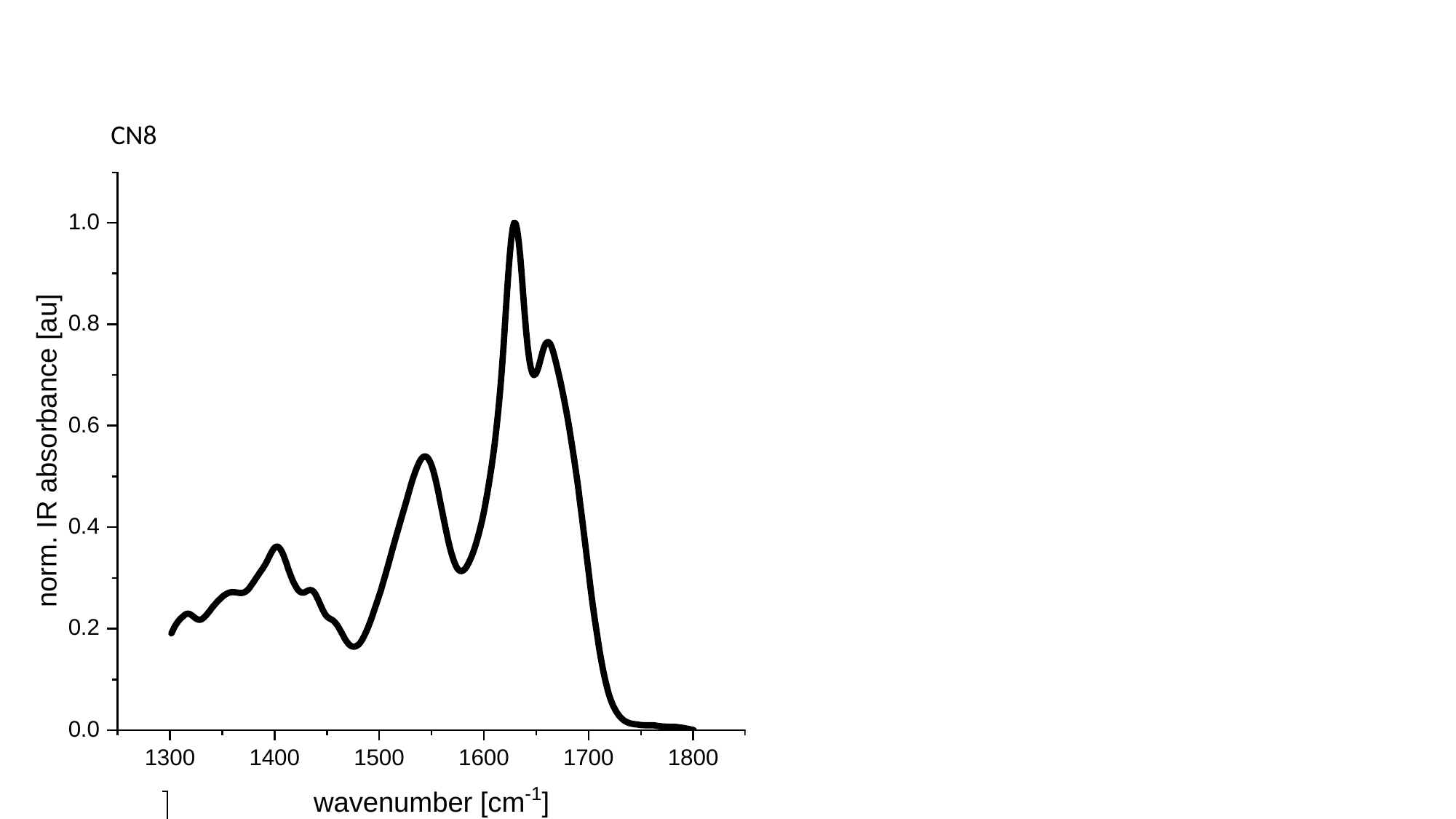

# CN8

## Slide 56
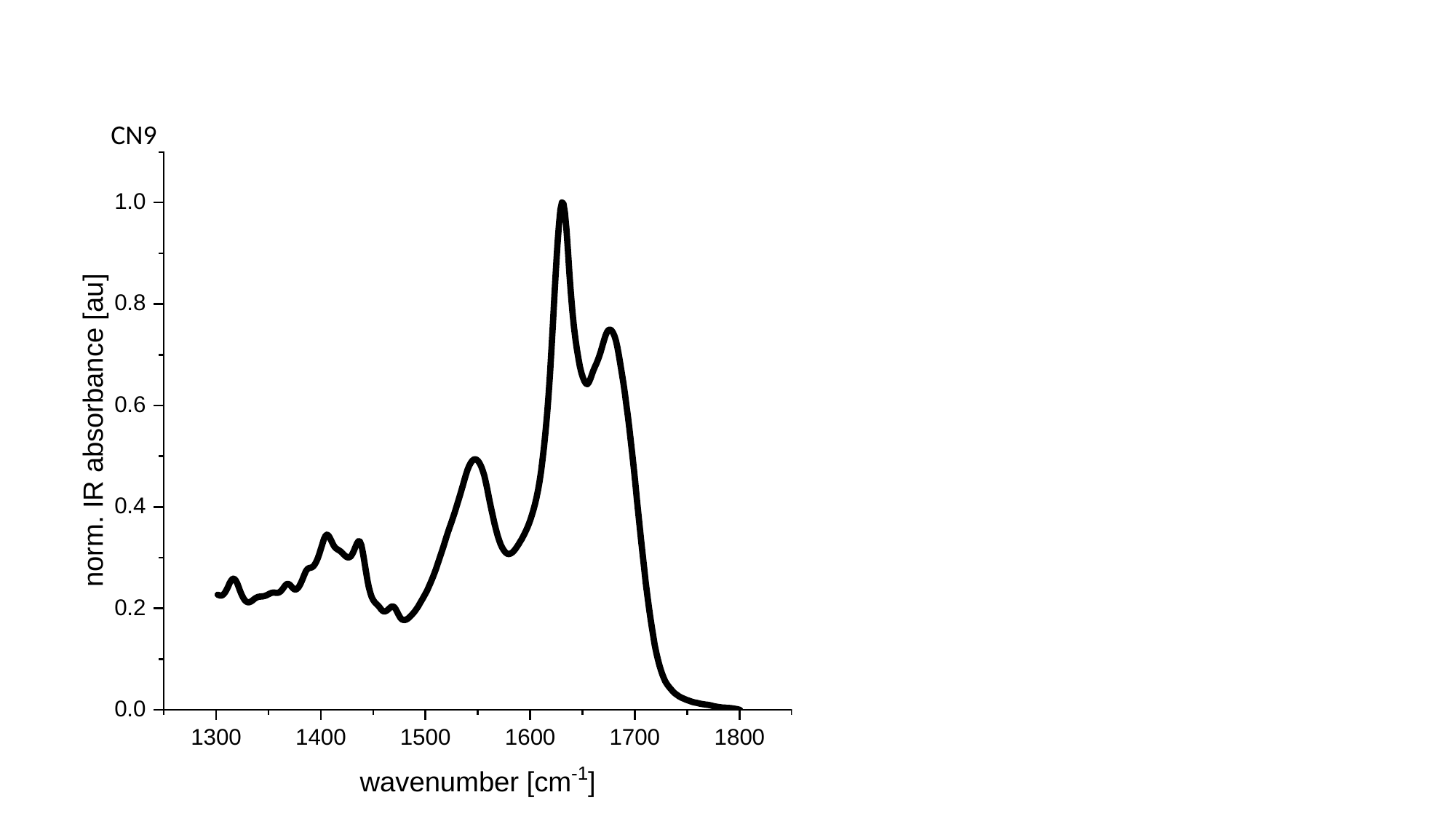

# CN9

## Slide 57
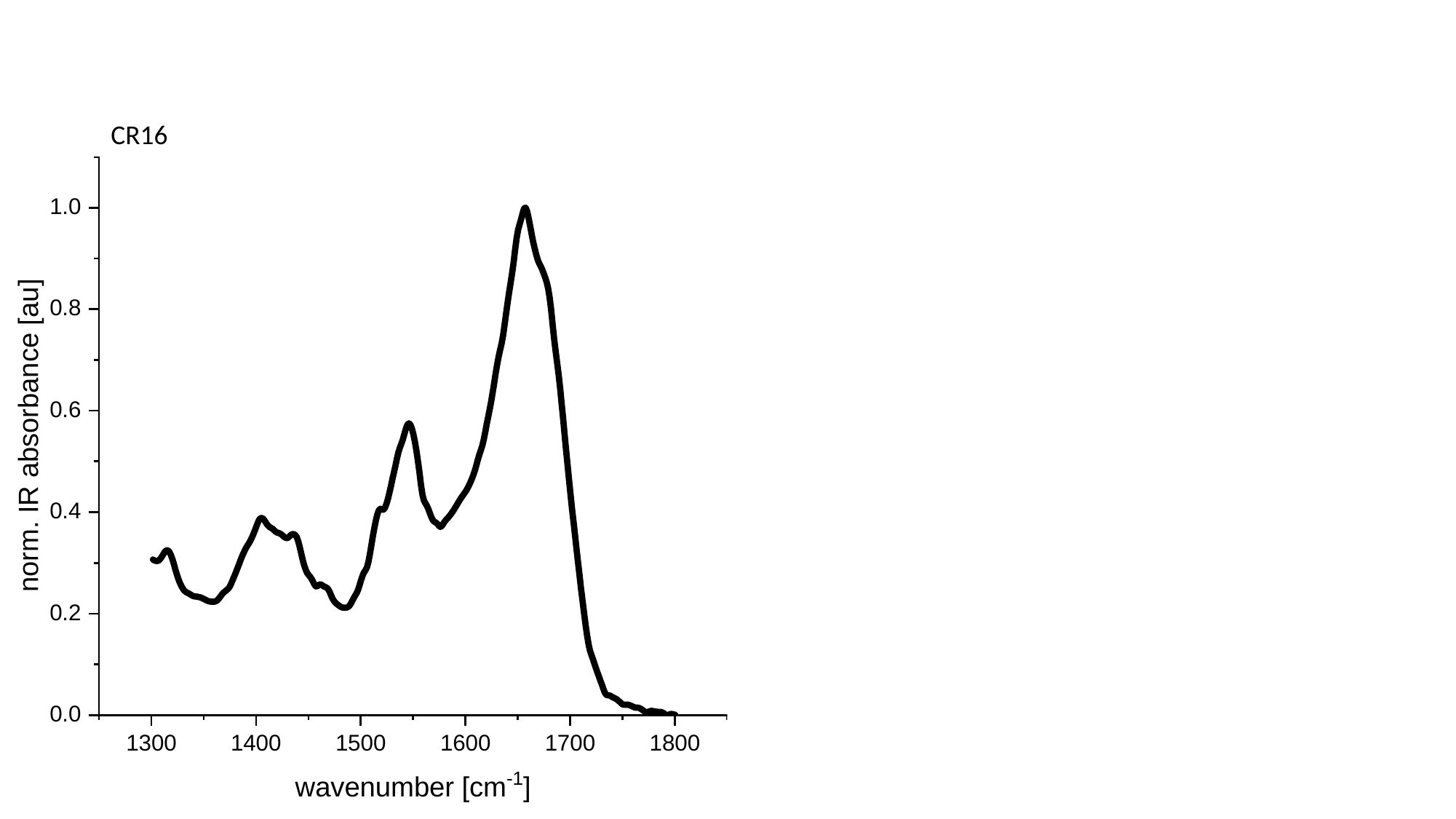

# CR16

## Slide 58
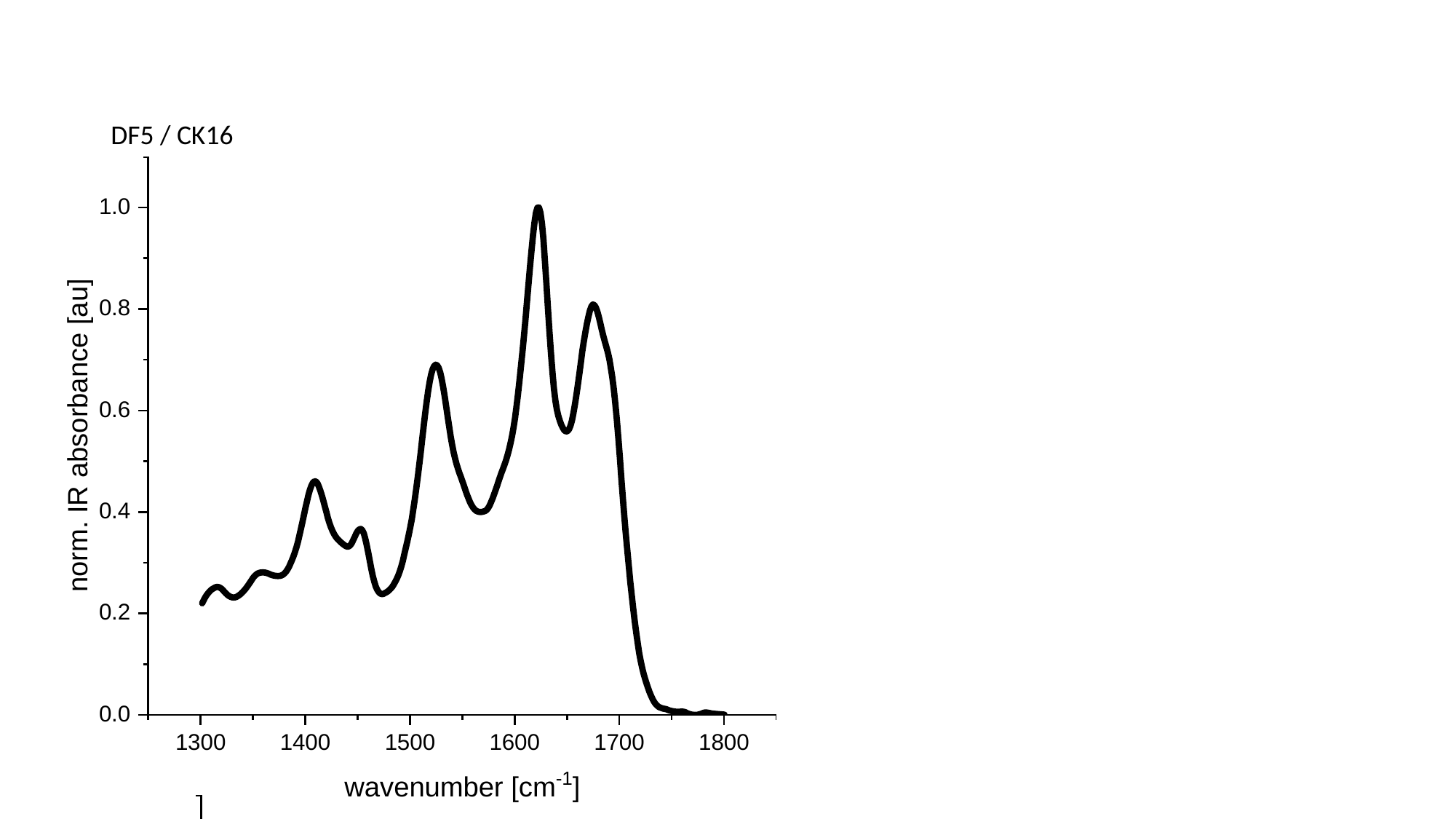

# DF5 / CK16

## Slide 59
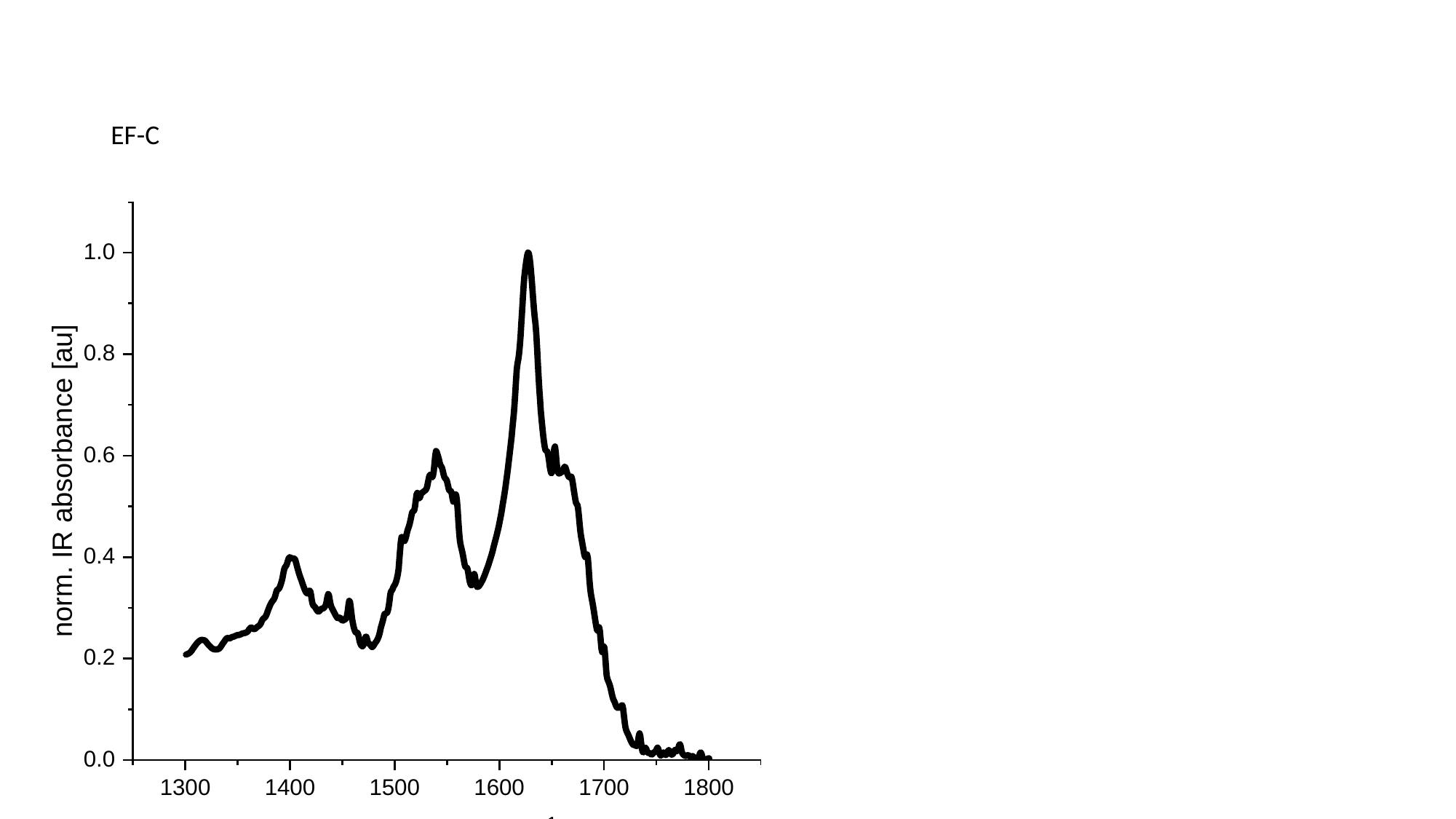

# EF-C

## Slide 60
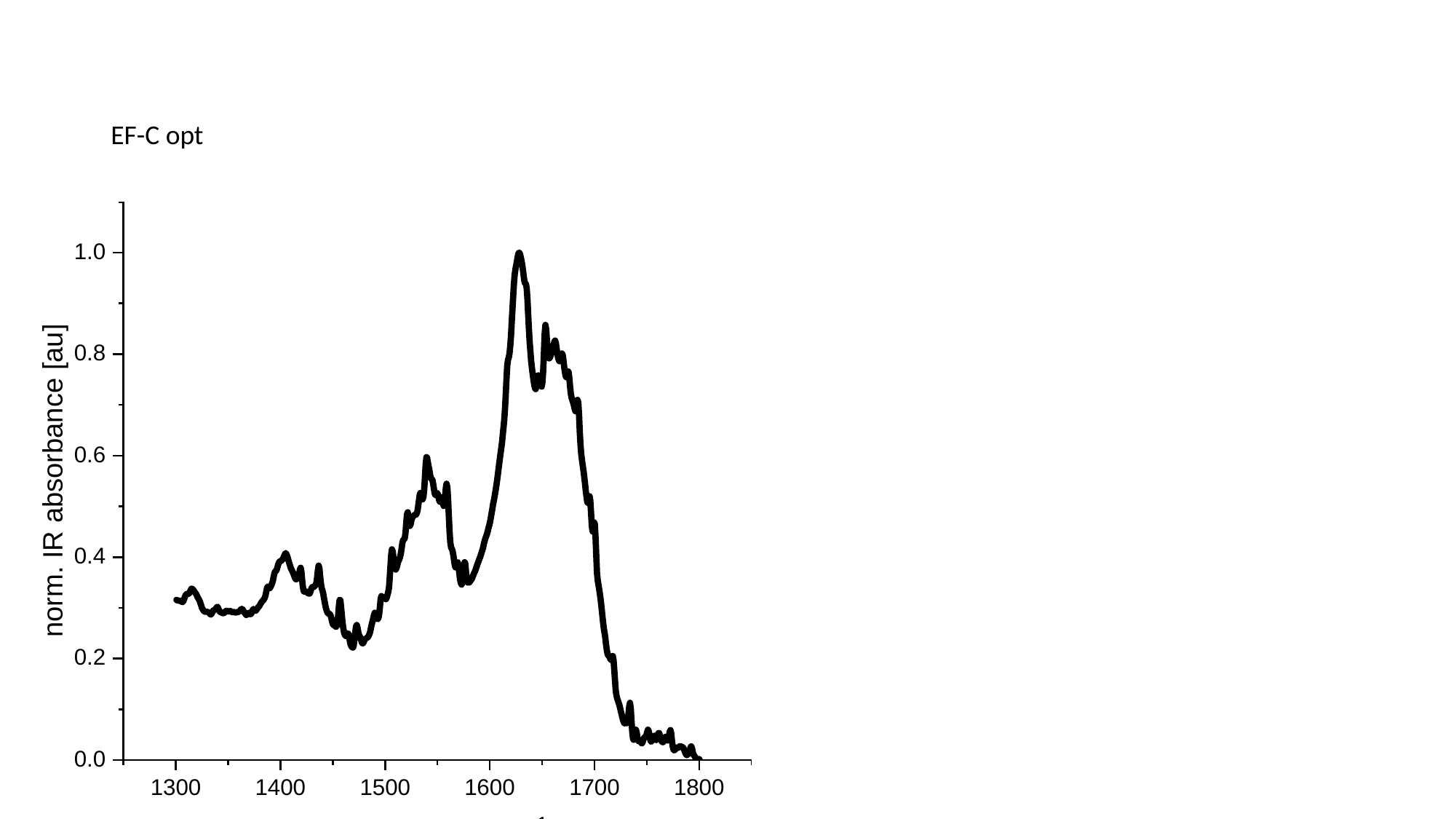

# EF-C opt

## Slide 61
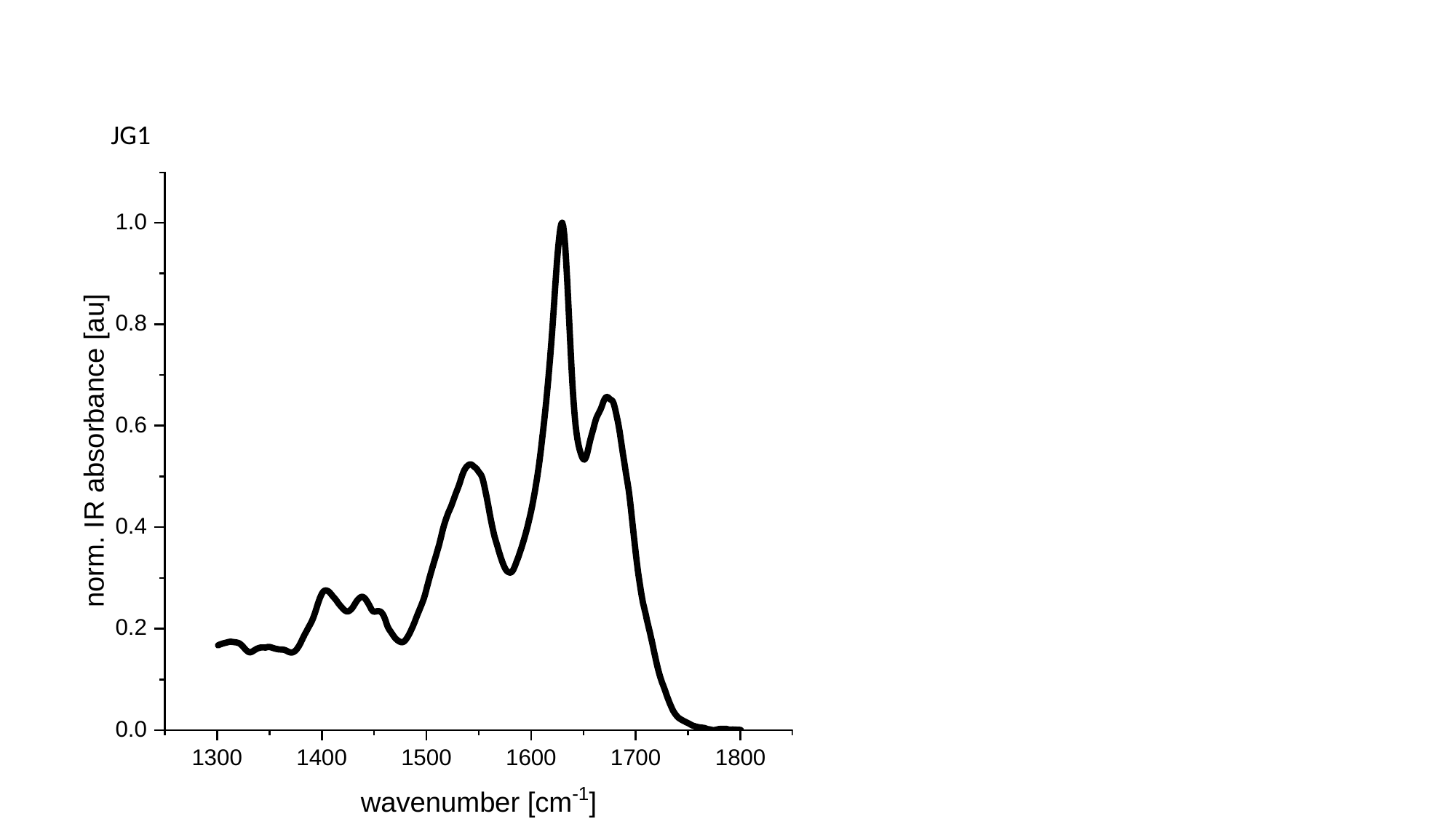

# JG1

## Slide 62
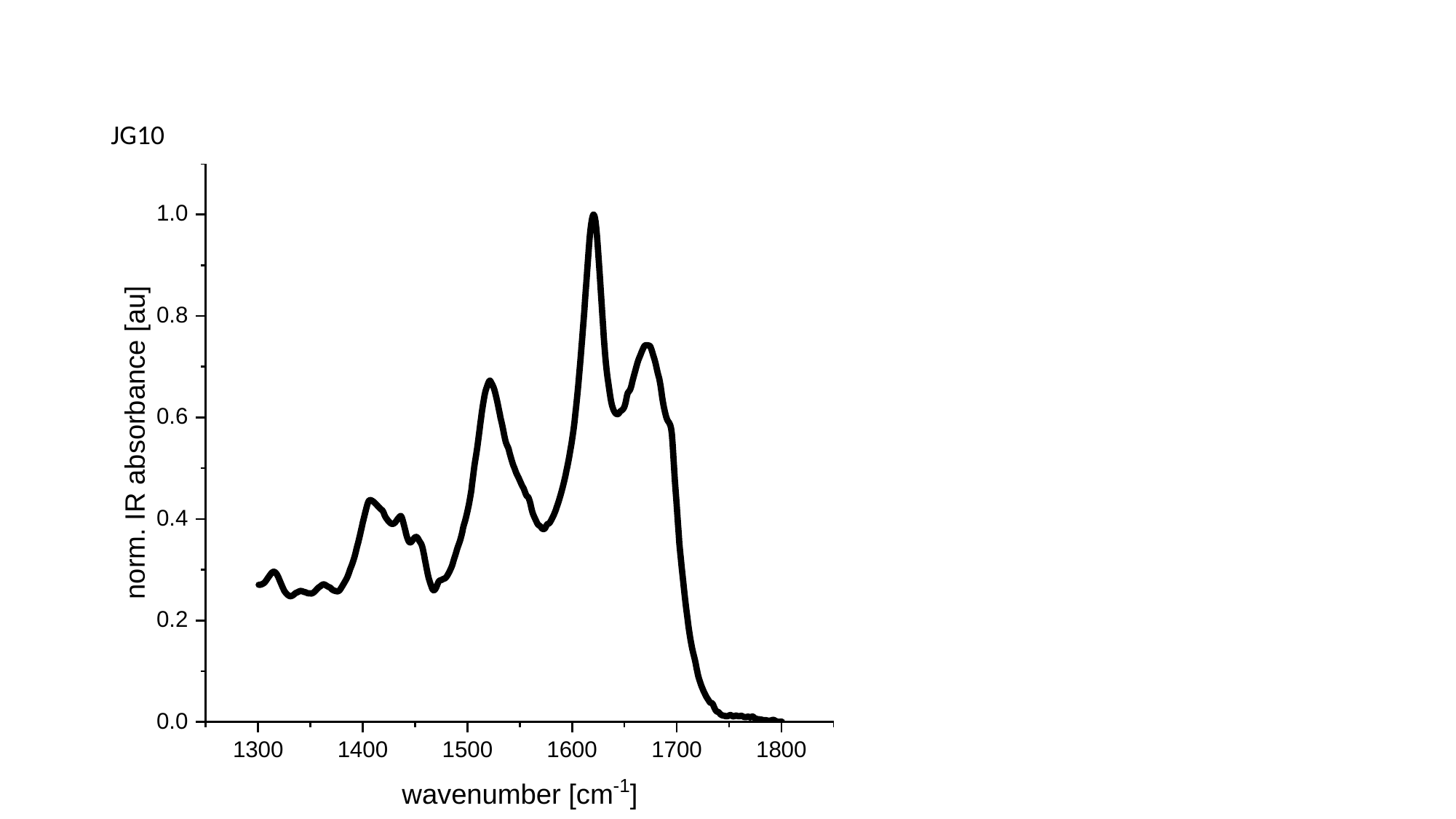

# JG10

## Slide 63
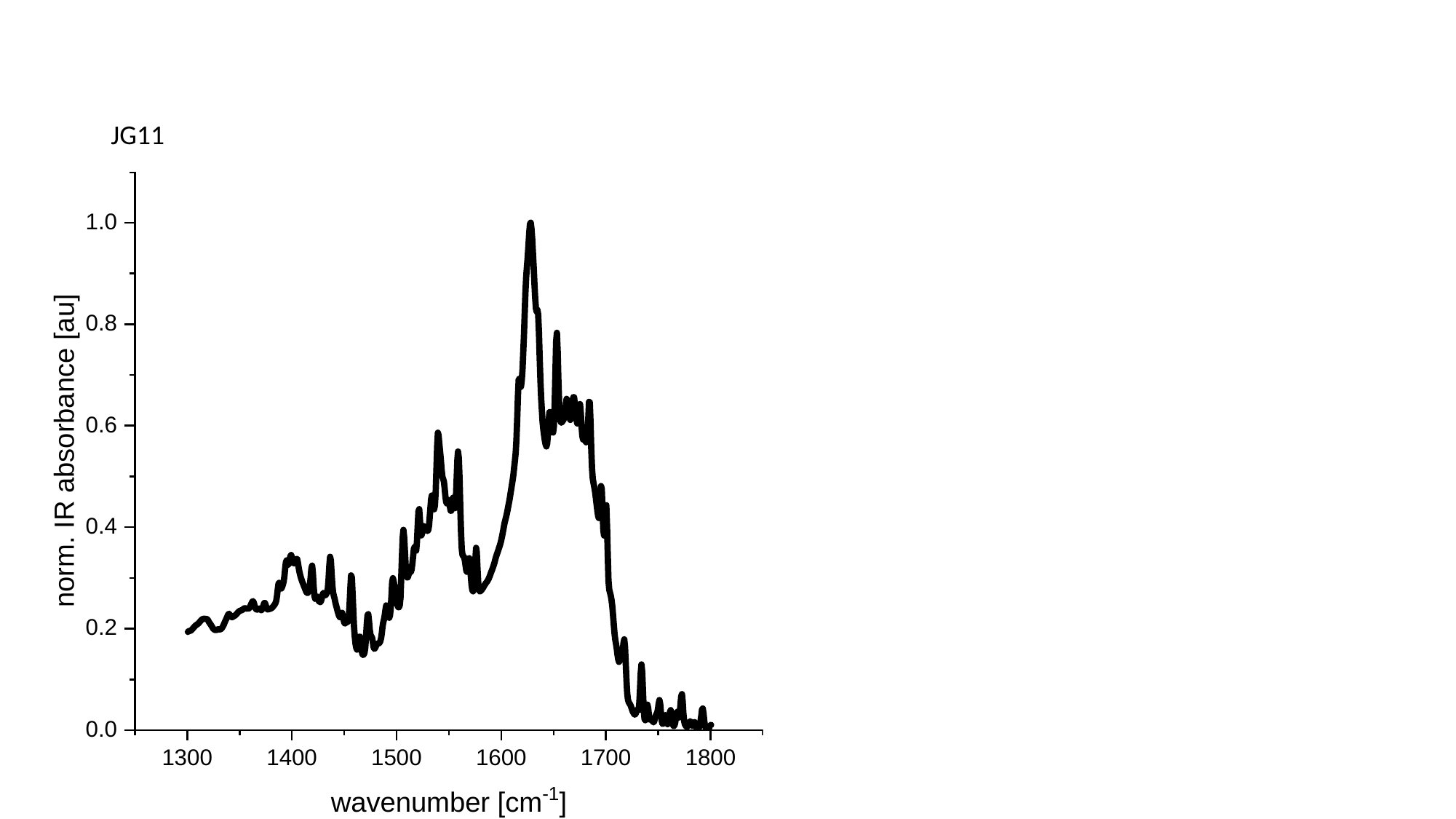

# JG11

## Slide 64
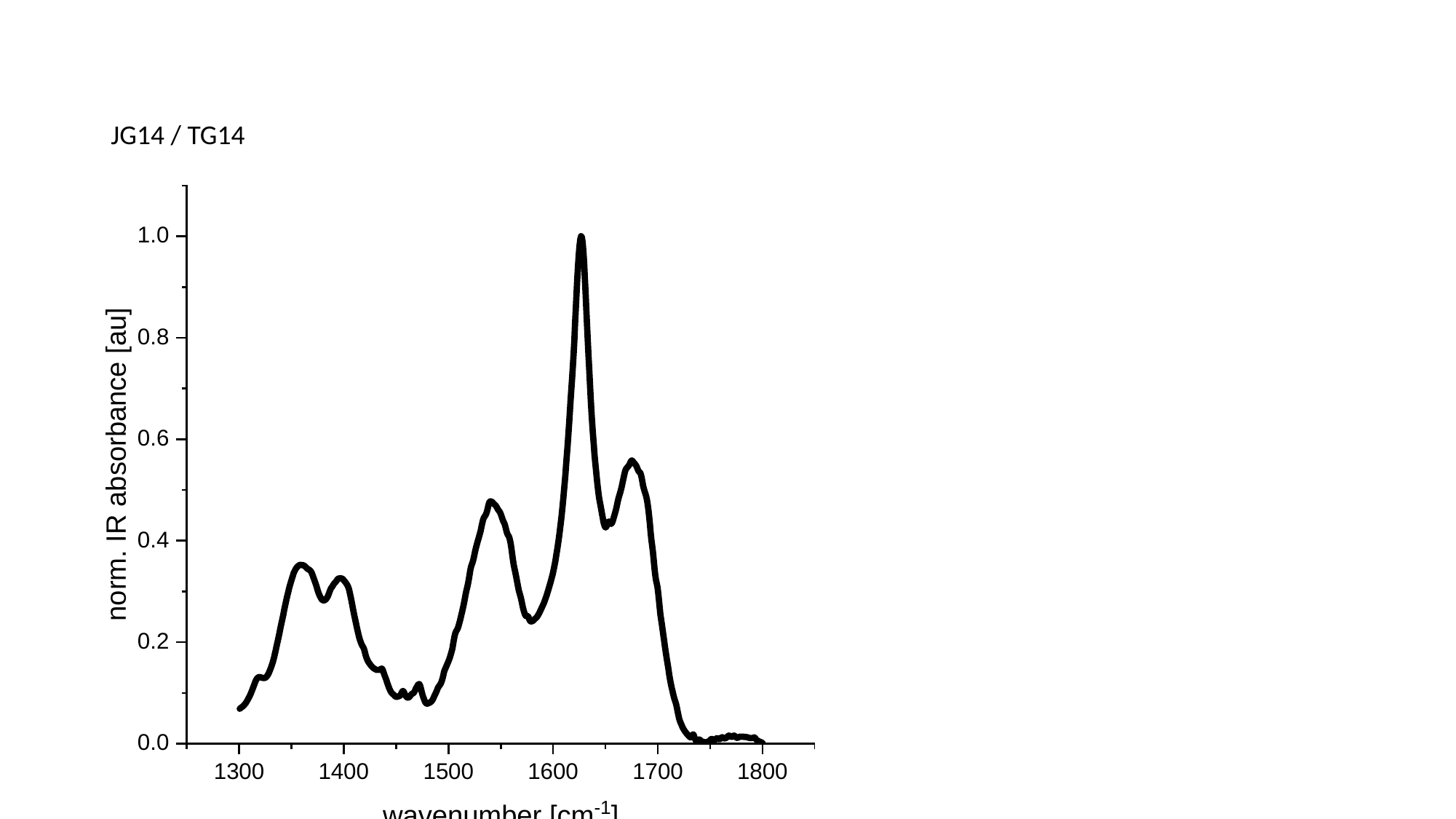

# JG14 / TG14

## Slide 65
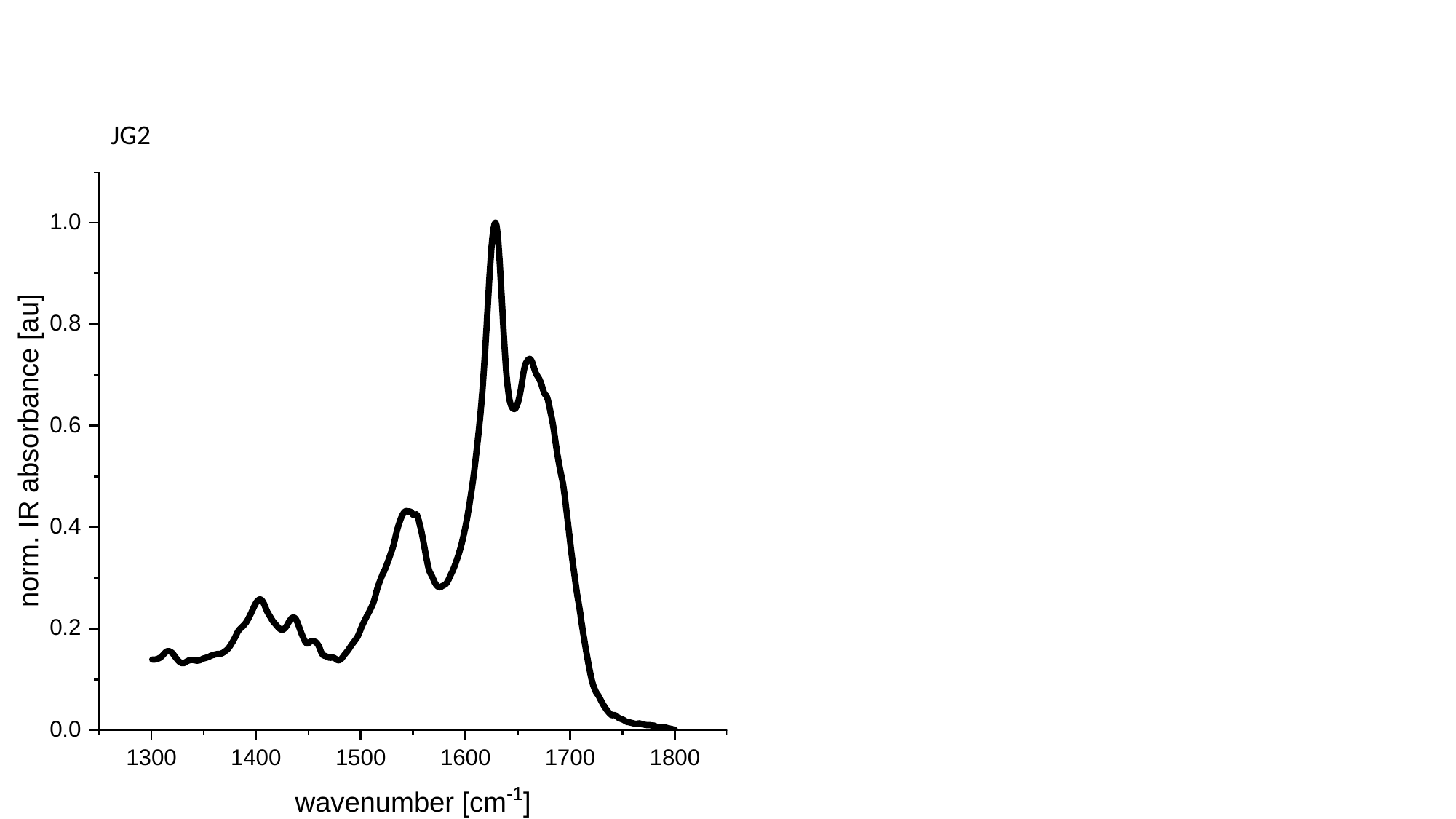

# JG2

## Slide 66
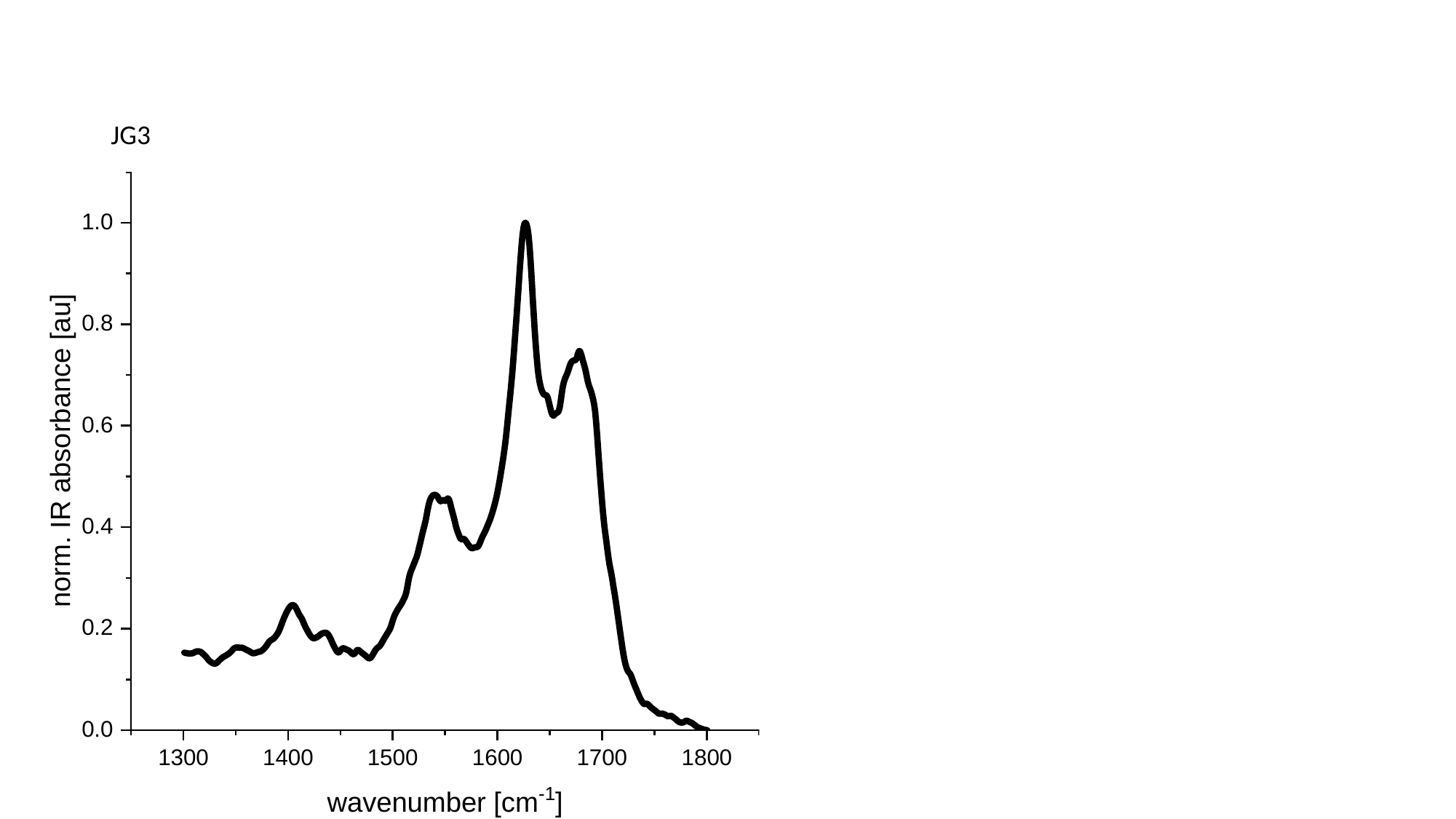

# JG3

## Slide 67
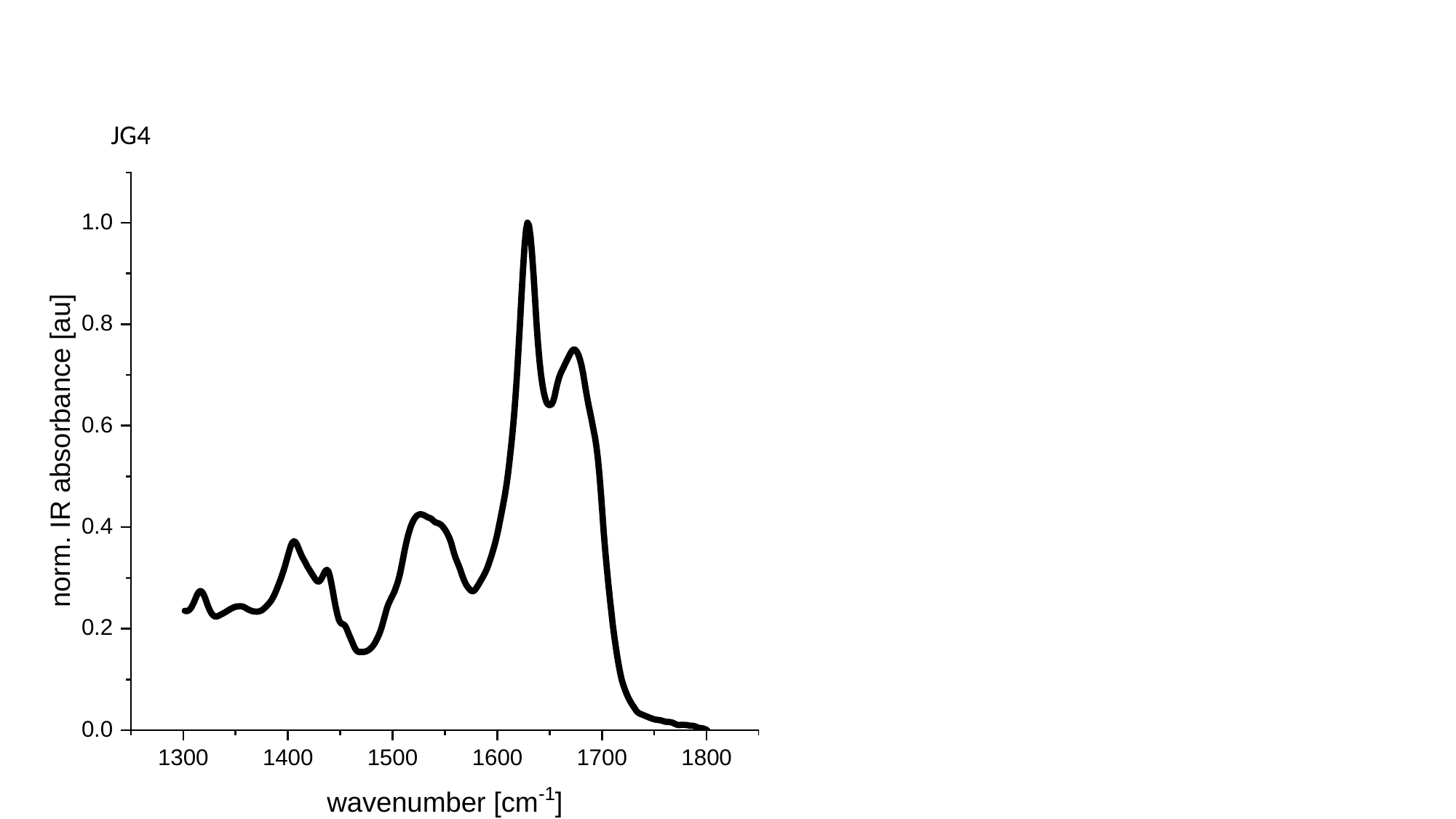

# JG4

## Slide 68
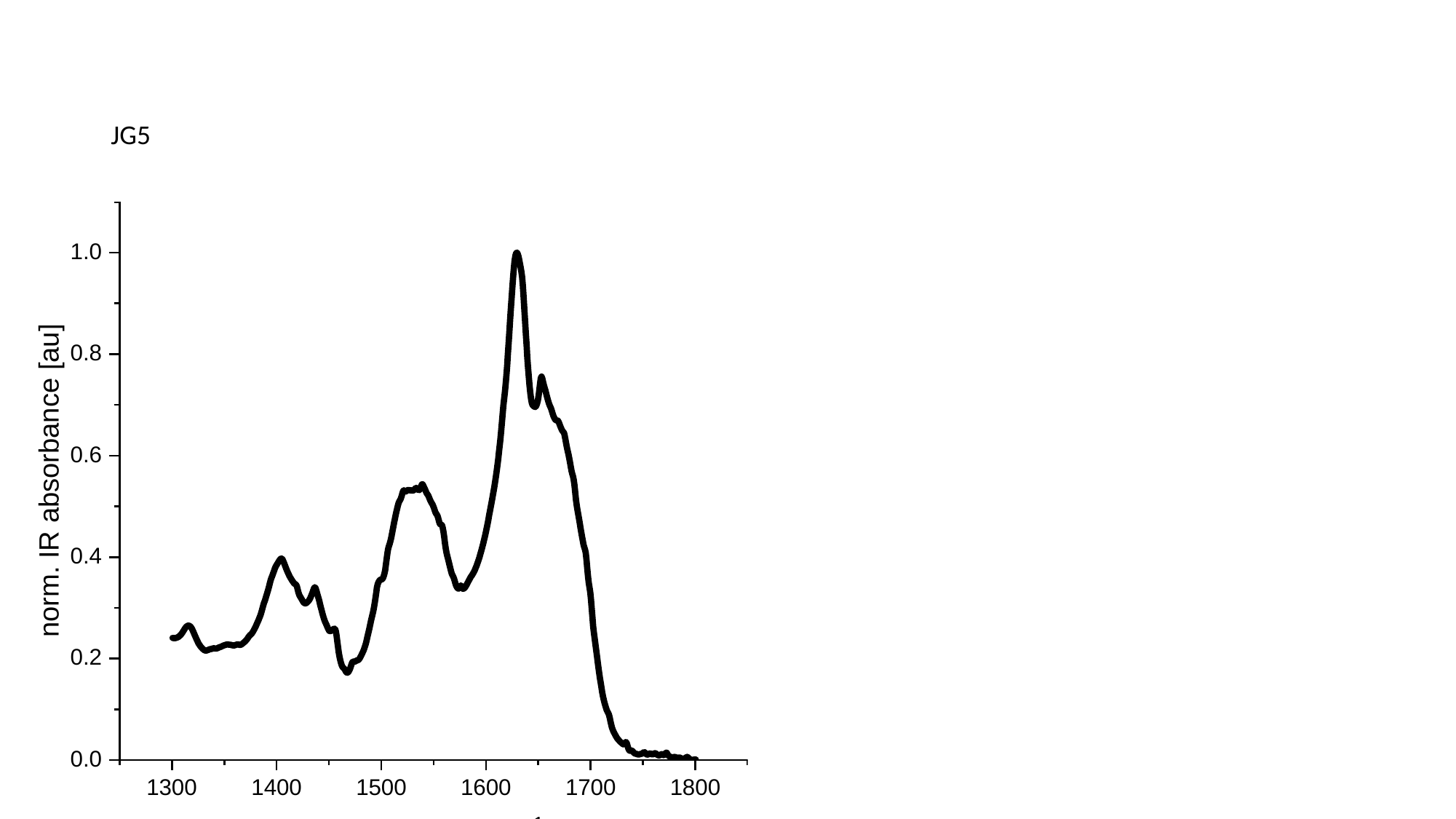

# JG5

## Slide 69
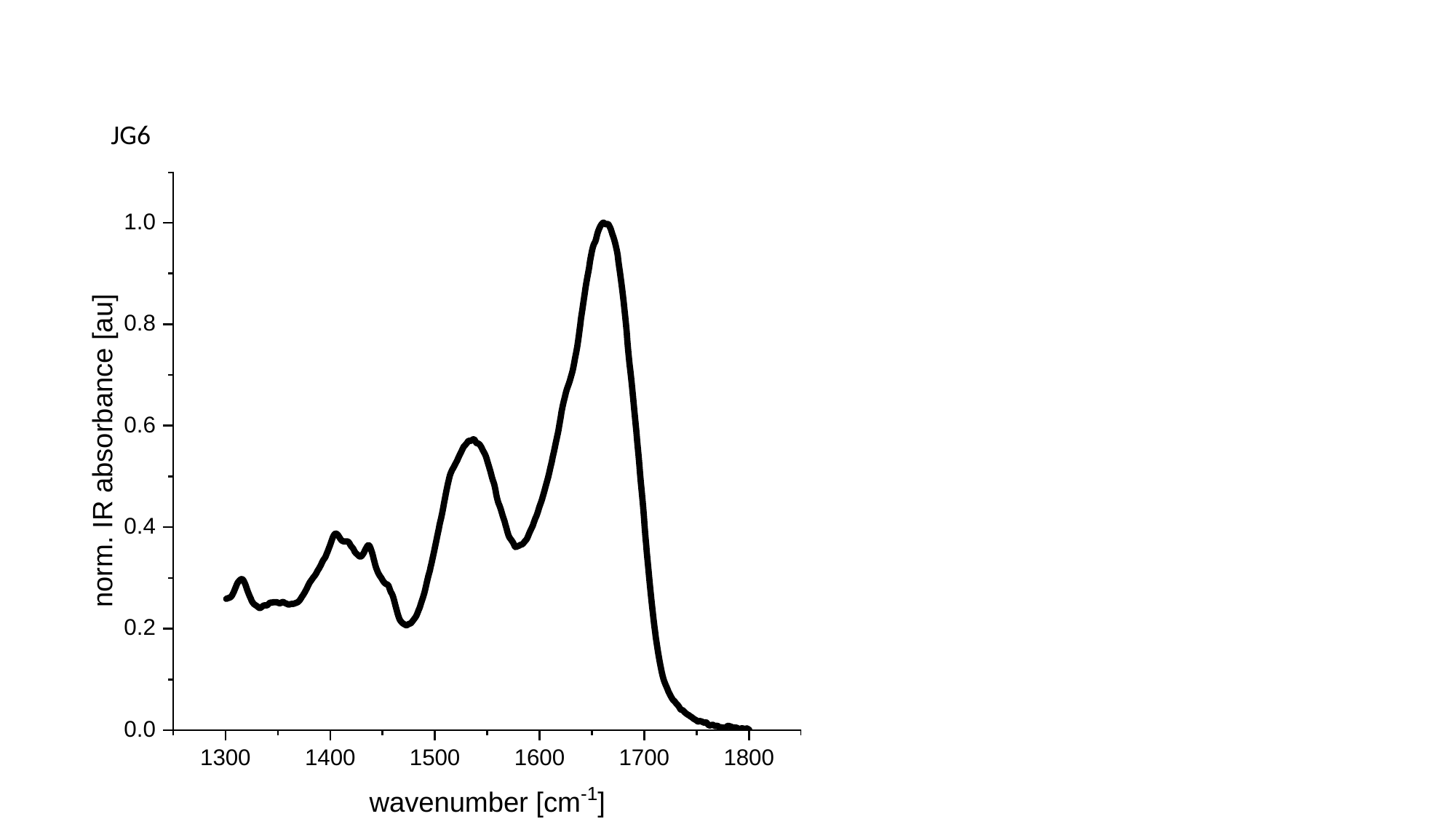

# JG6

## Slide 70
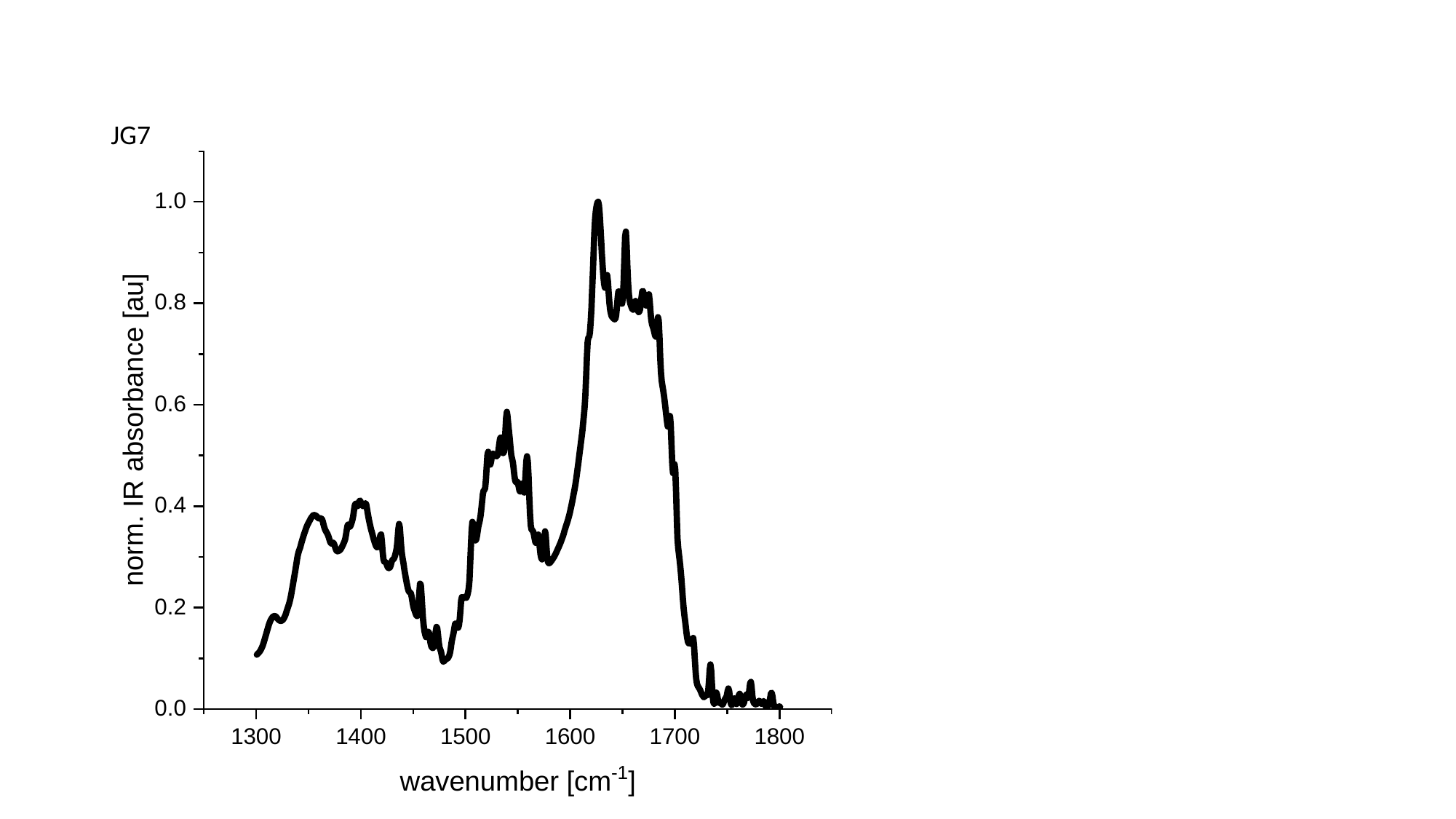

# JG7

## Slide 71
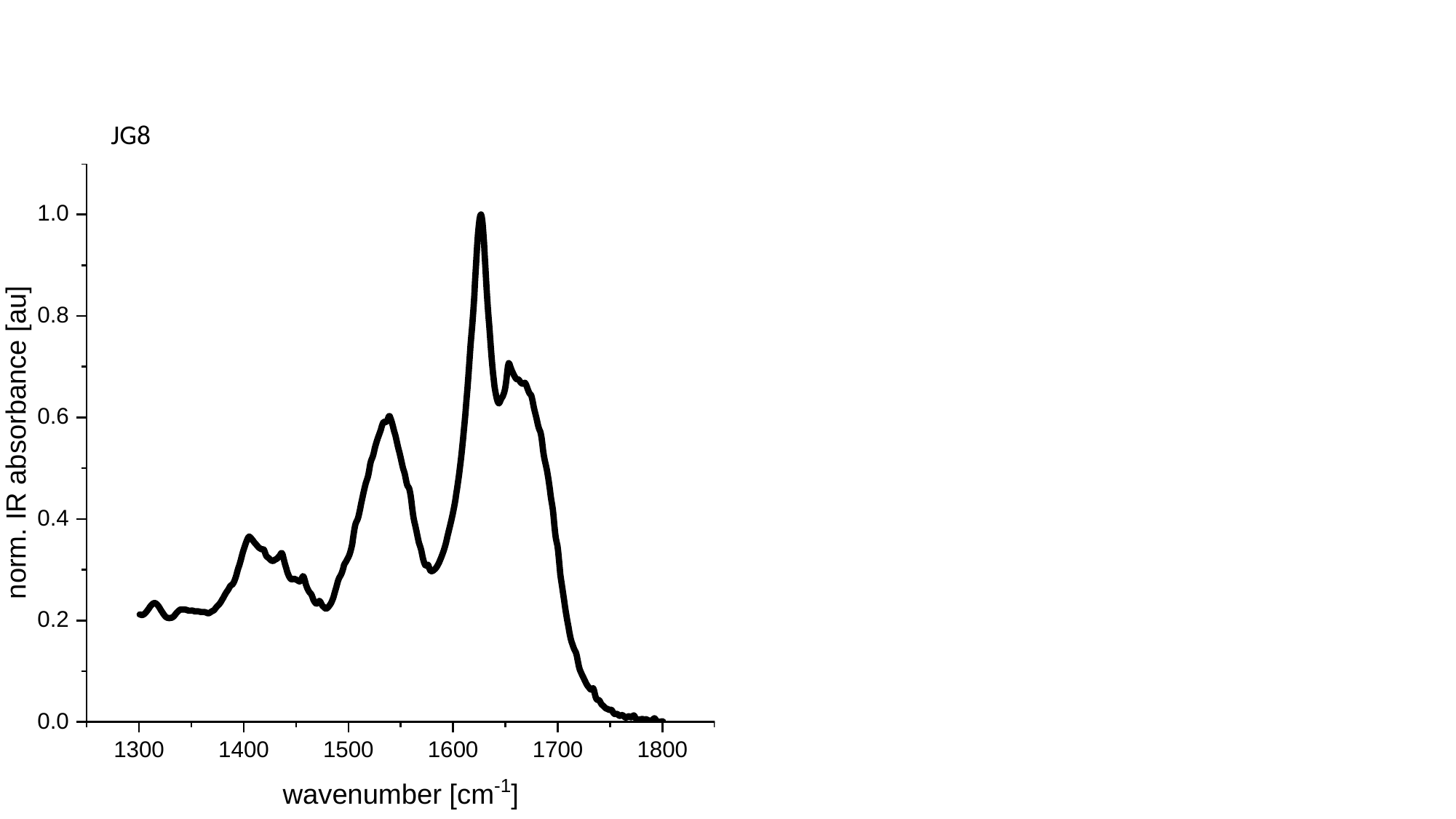

# JG8

## Slide 72
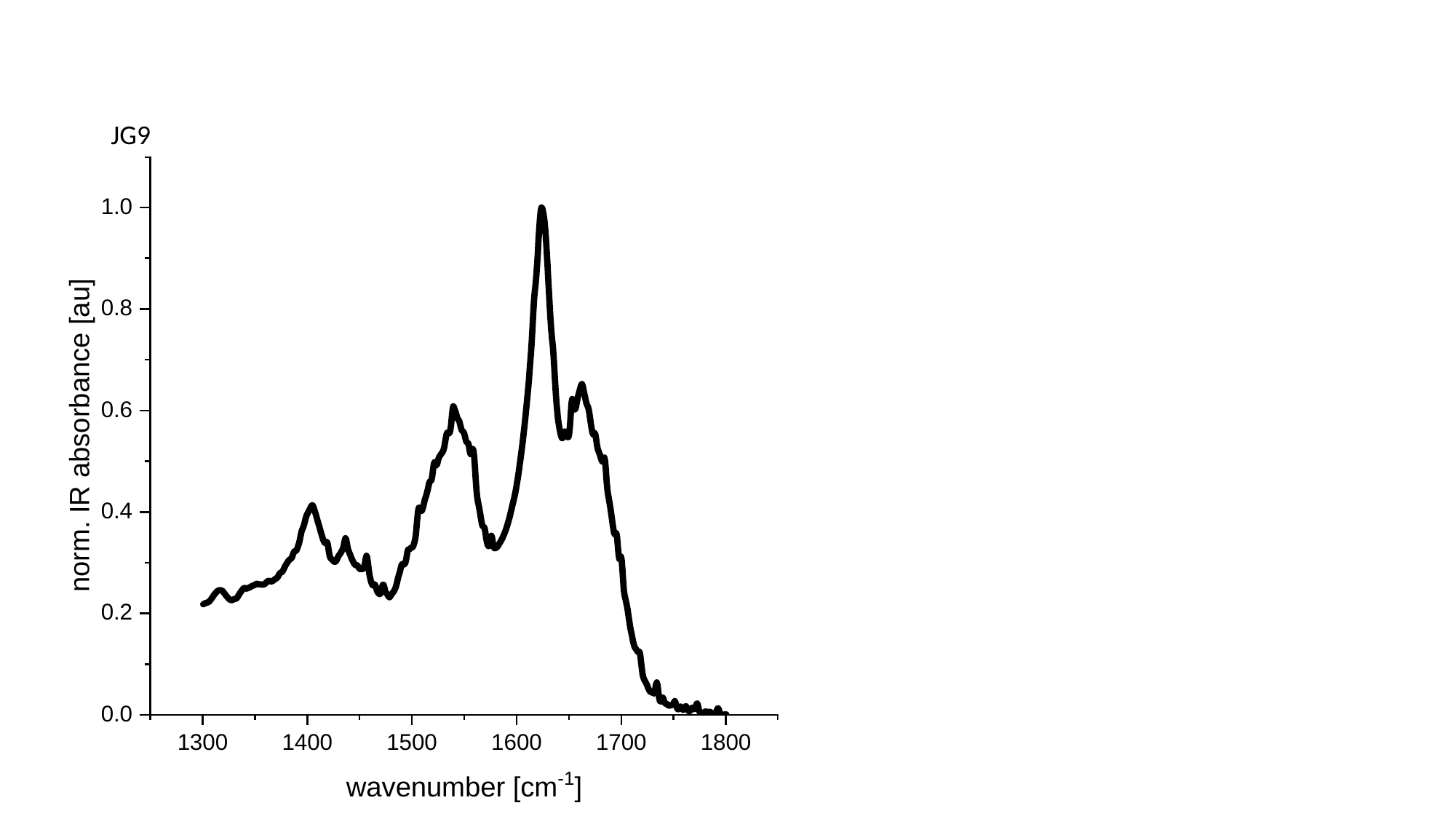

# JG9

## Slide 73
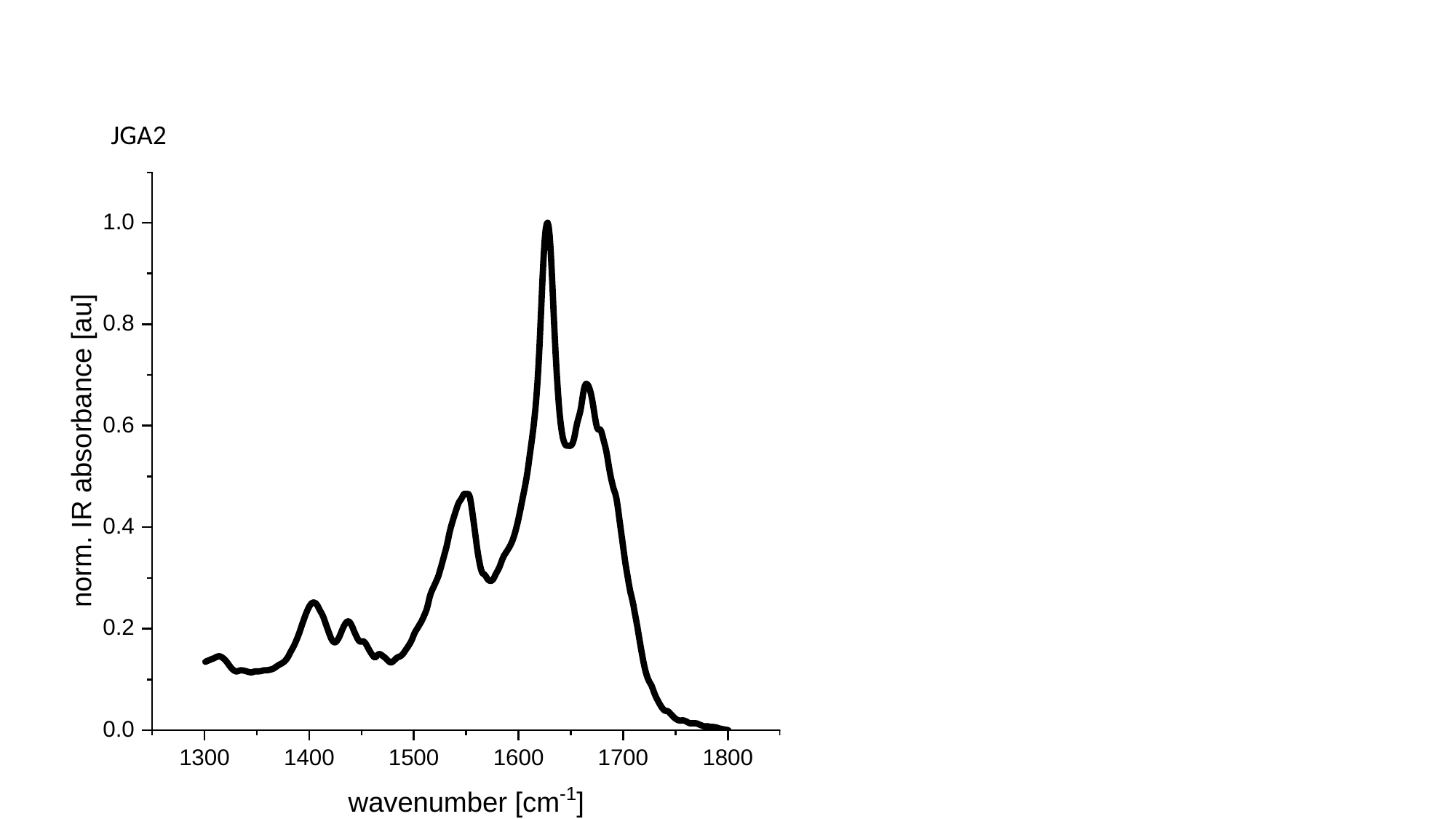

# JGA2

## Slide 74
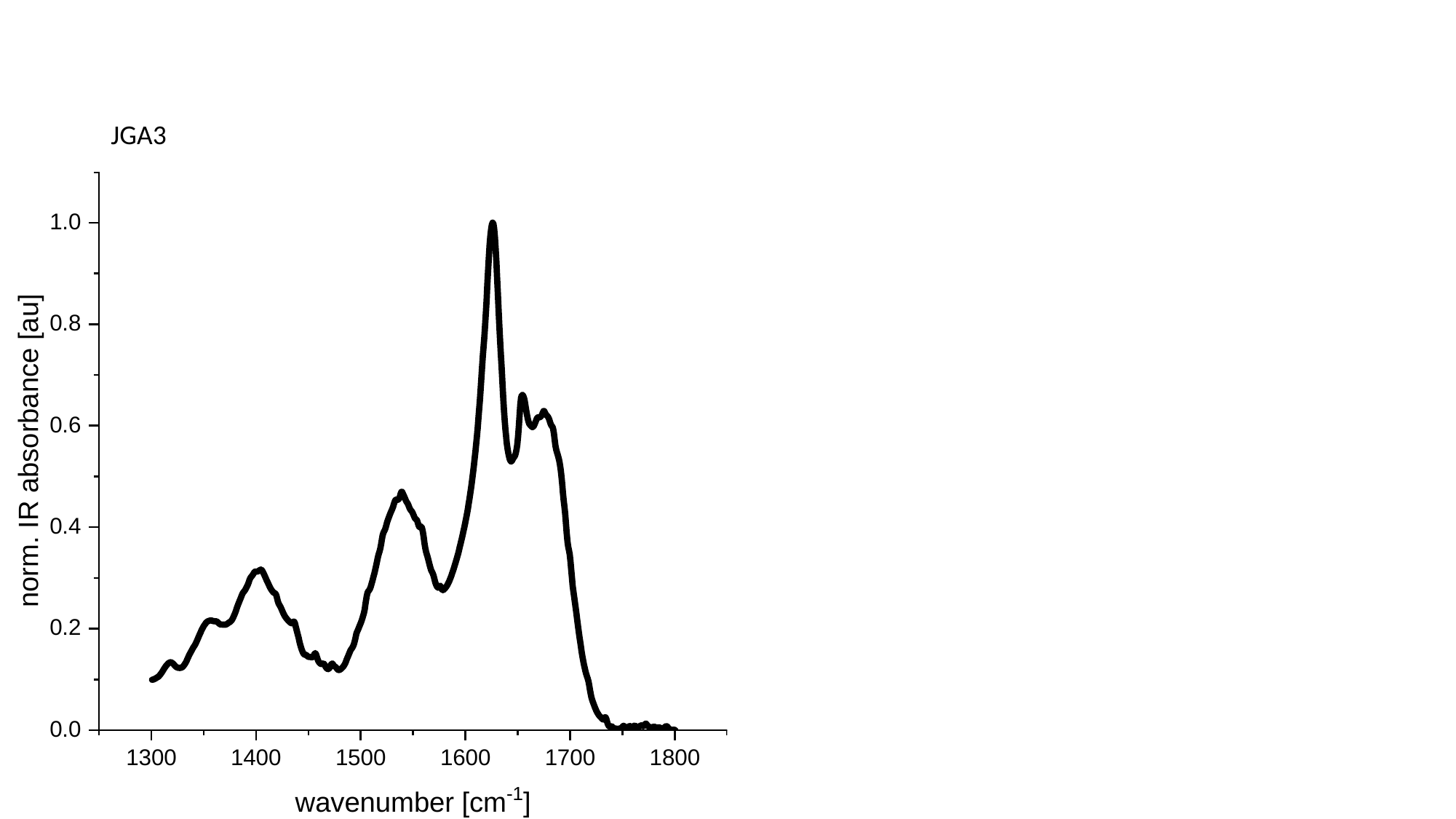

# JGA3

## Slide 75
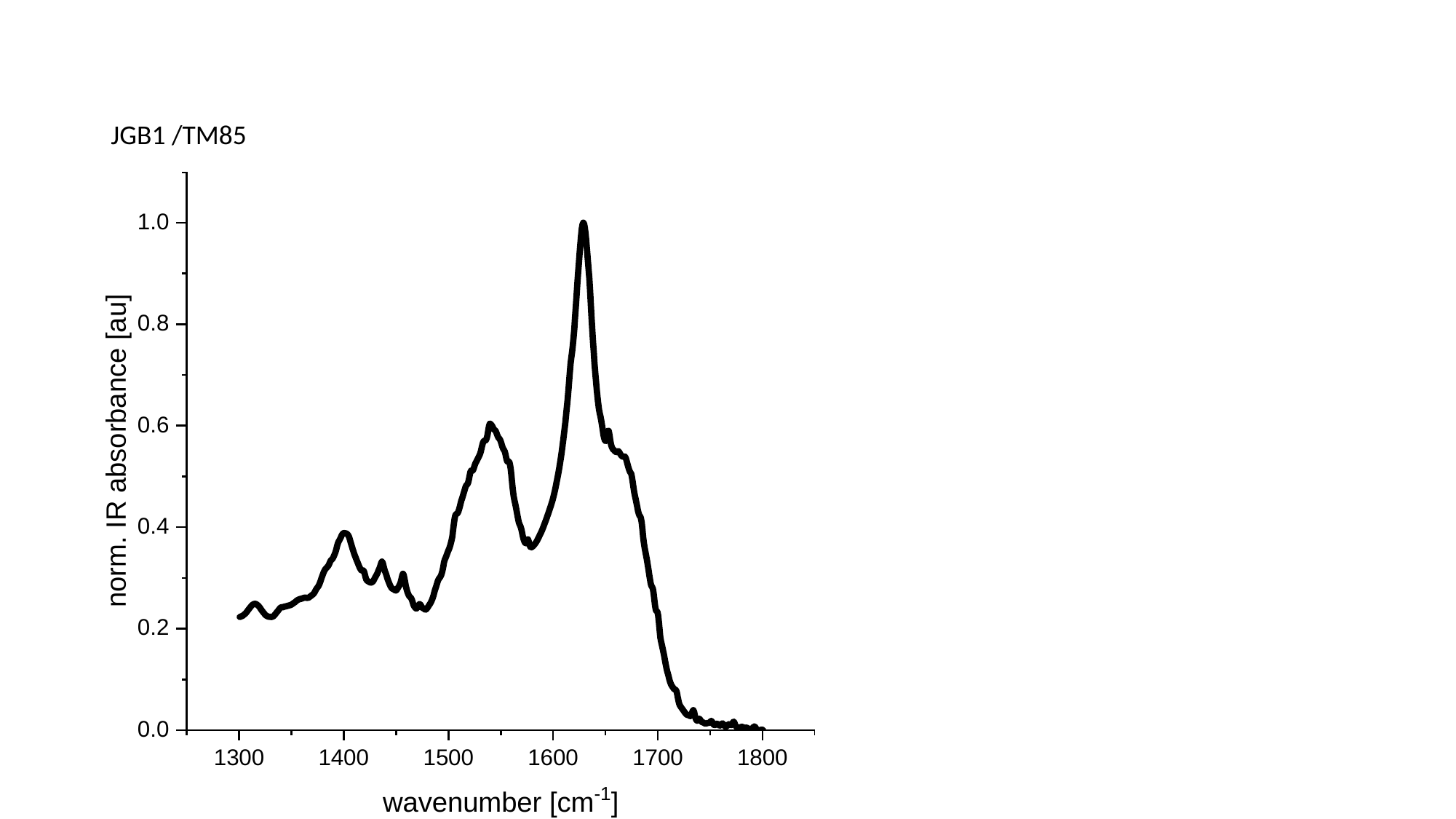

# JGB1 /TM85

## Slide 76
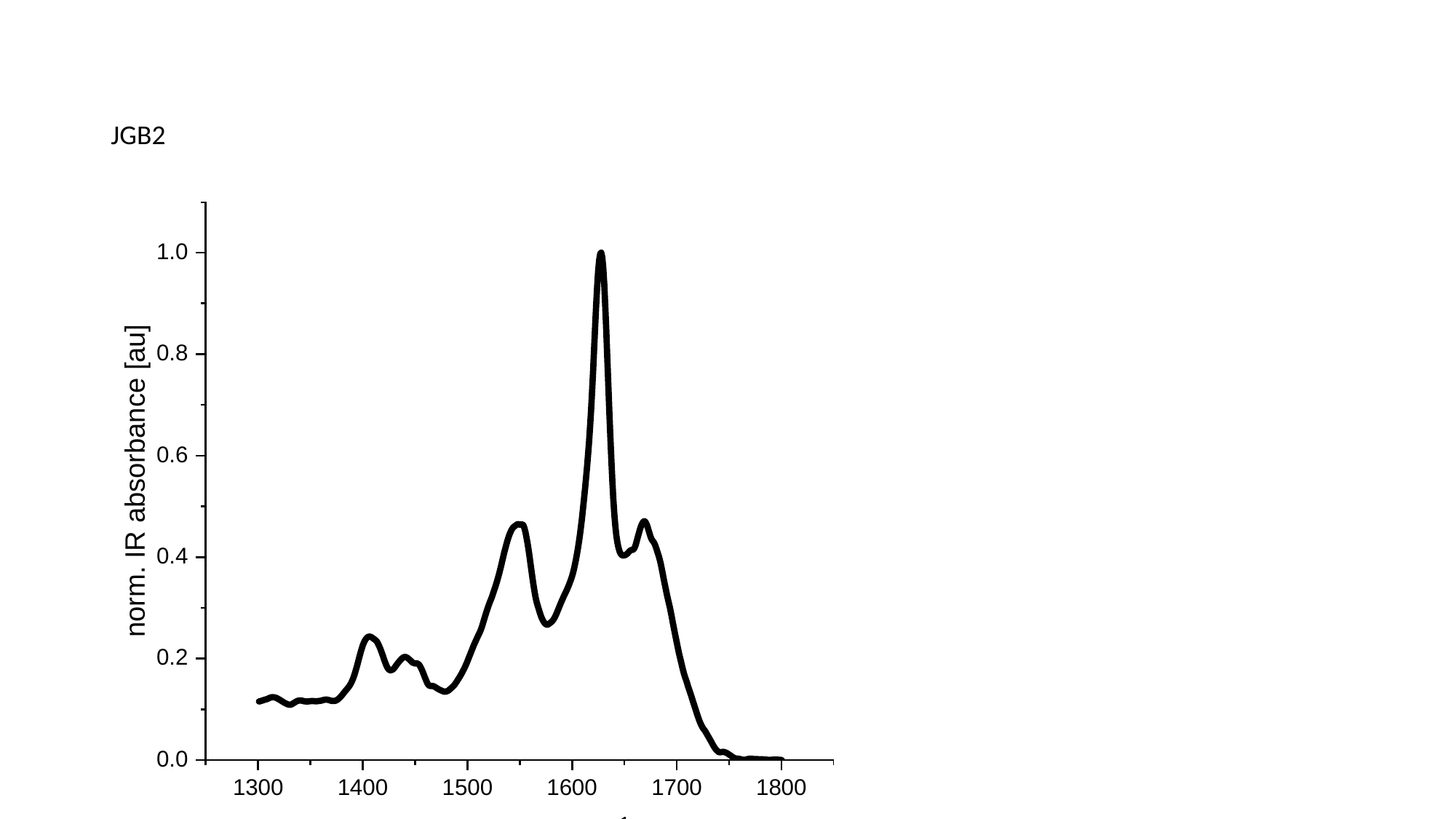

# JGB2

## Slide 77
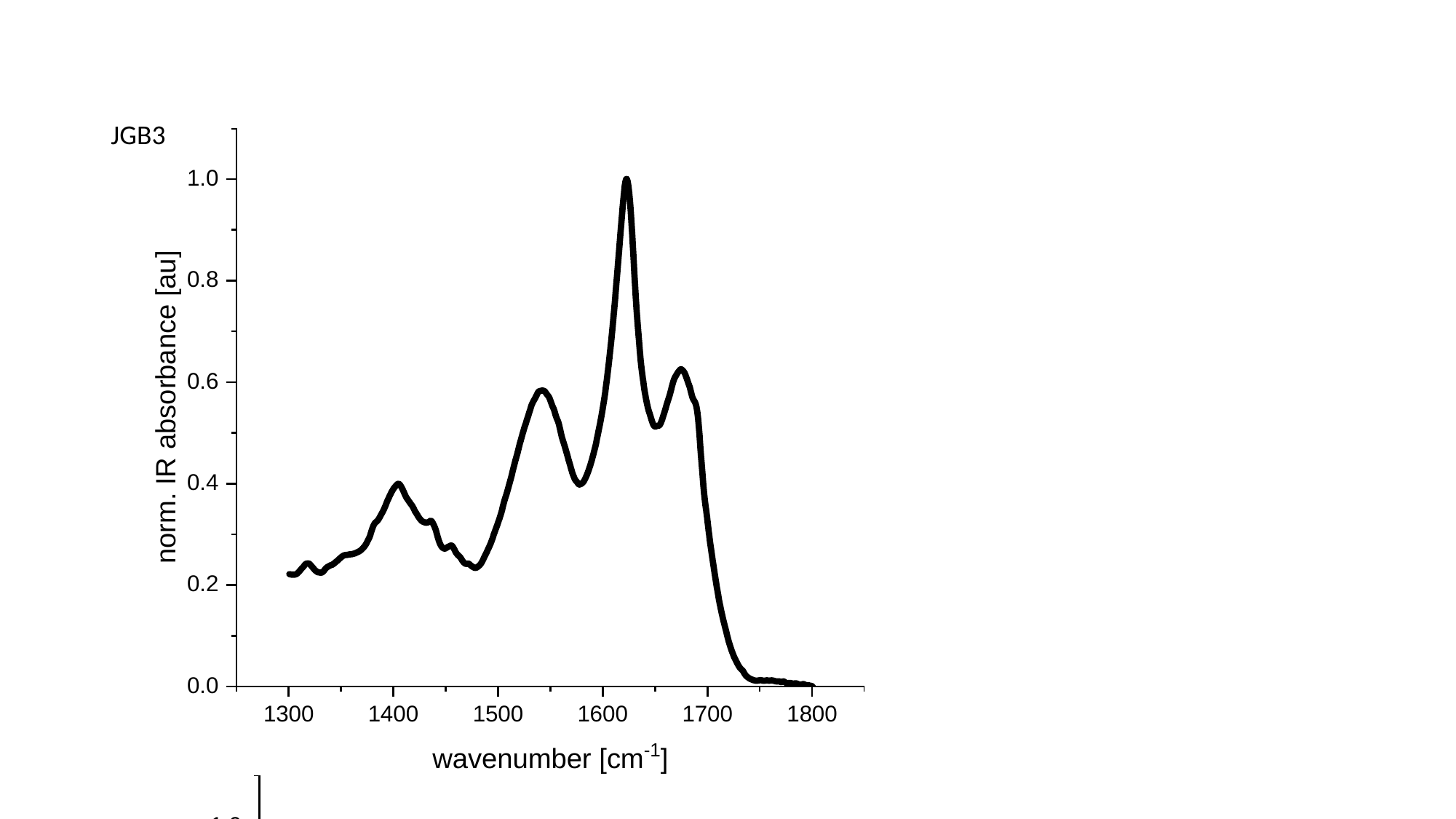

# JGB3

## Slide 78
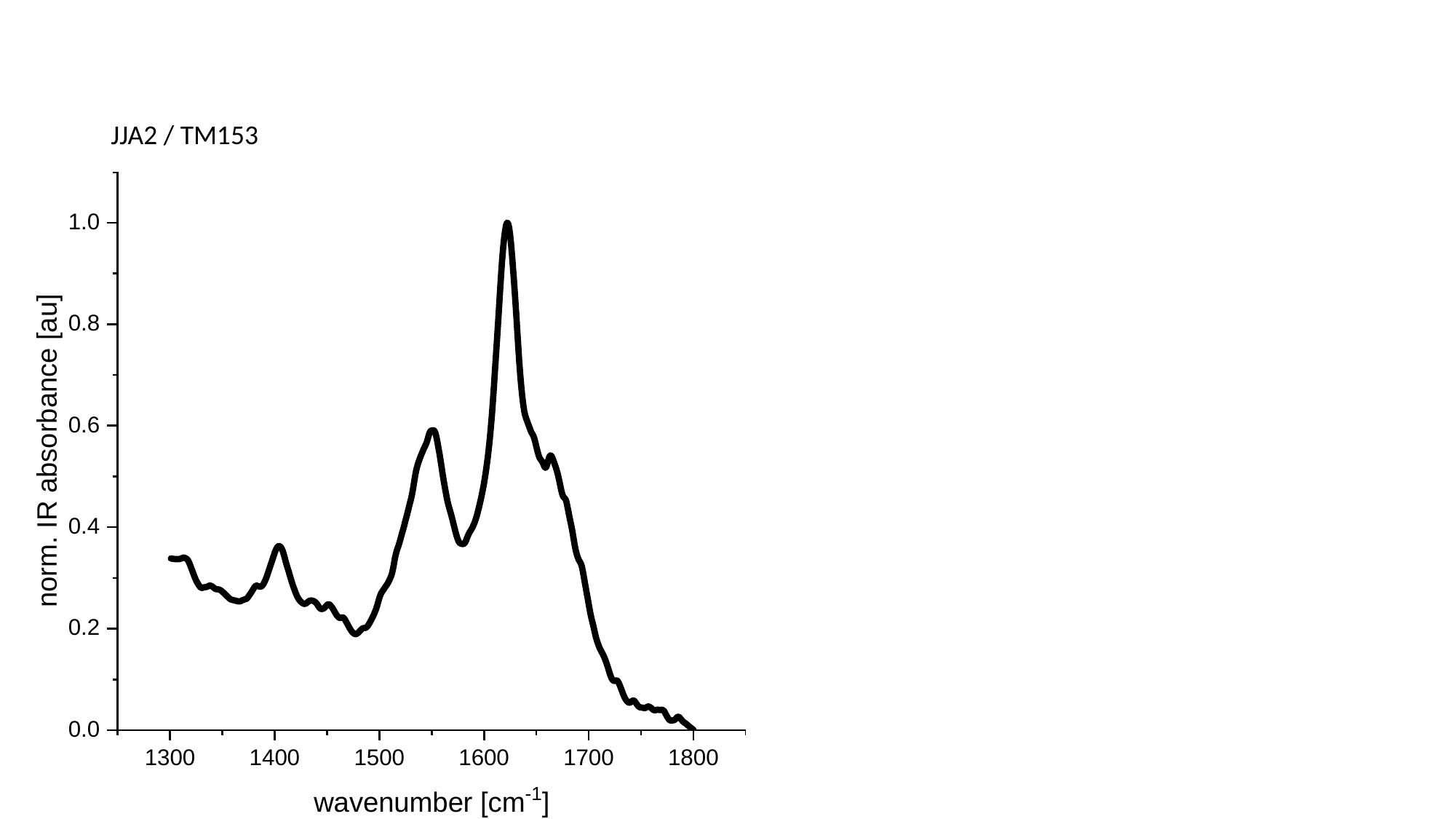

# JJA2 / TM153

## Slide 79
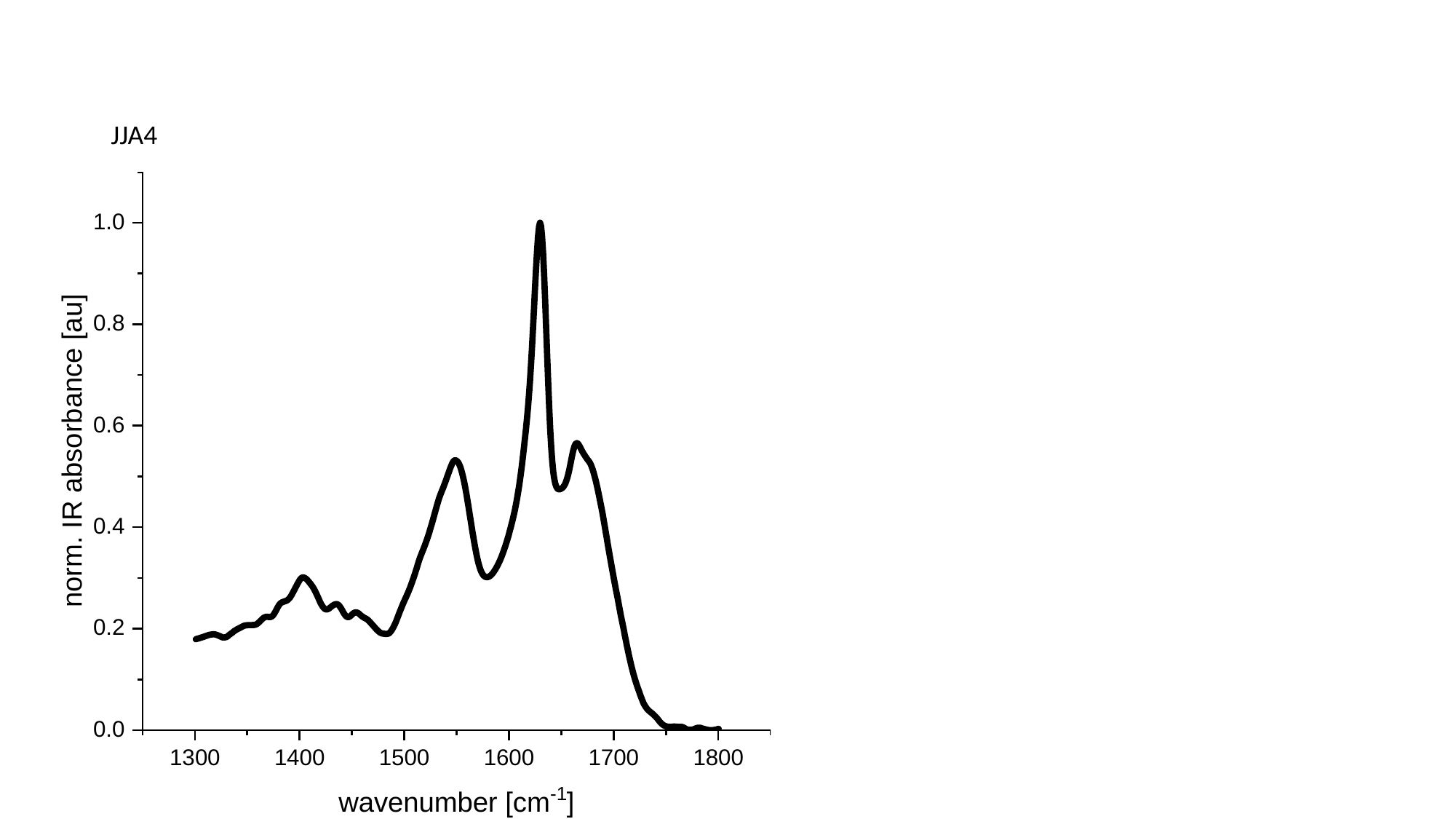

# JJA4

## Slide 80
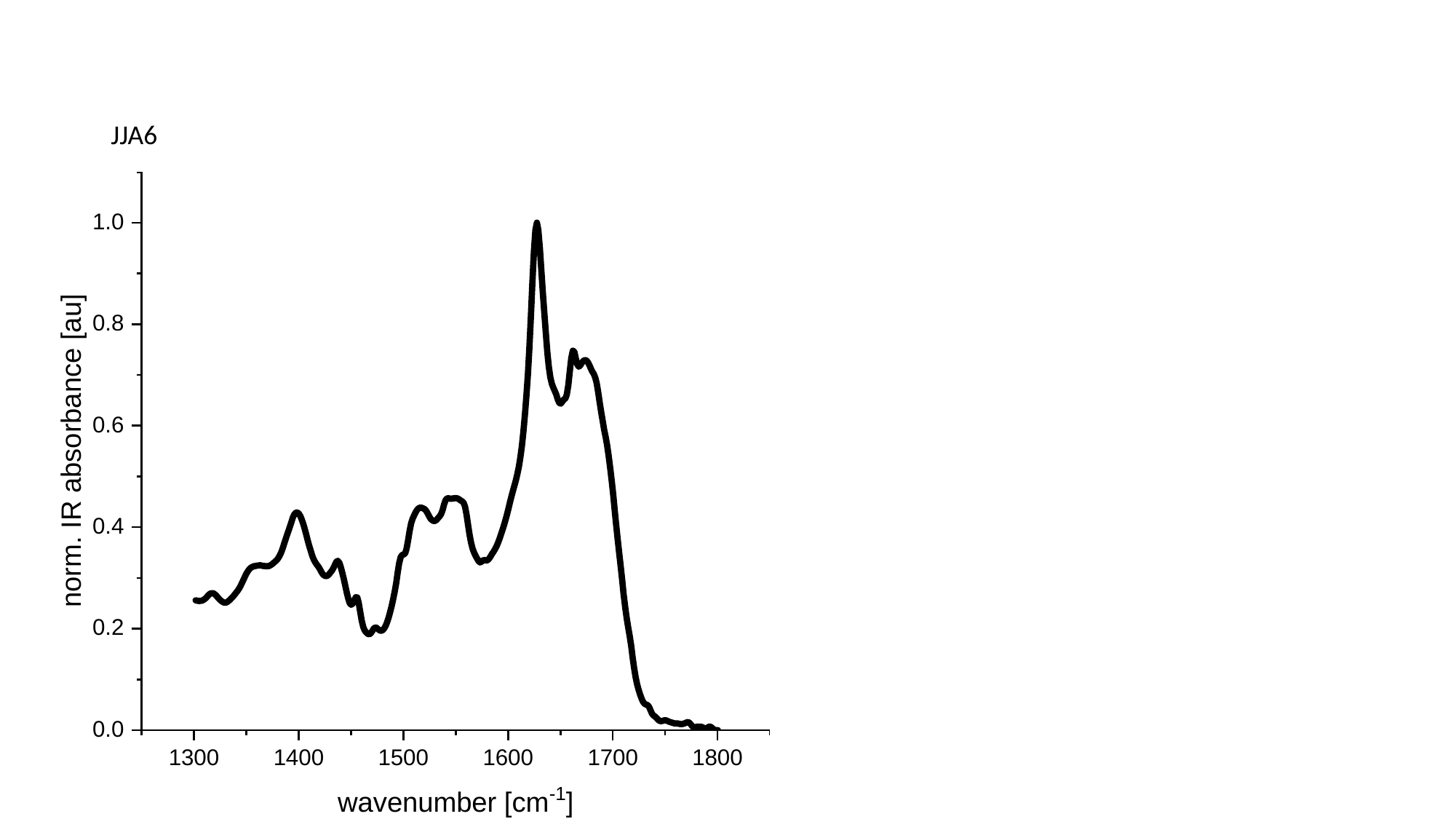

# JJA6

## Slide 81
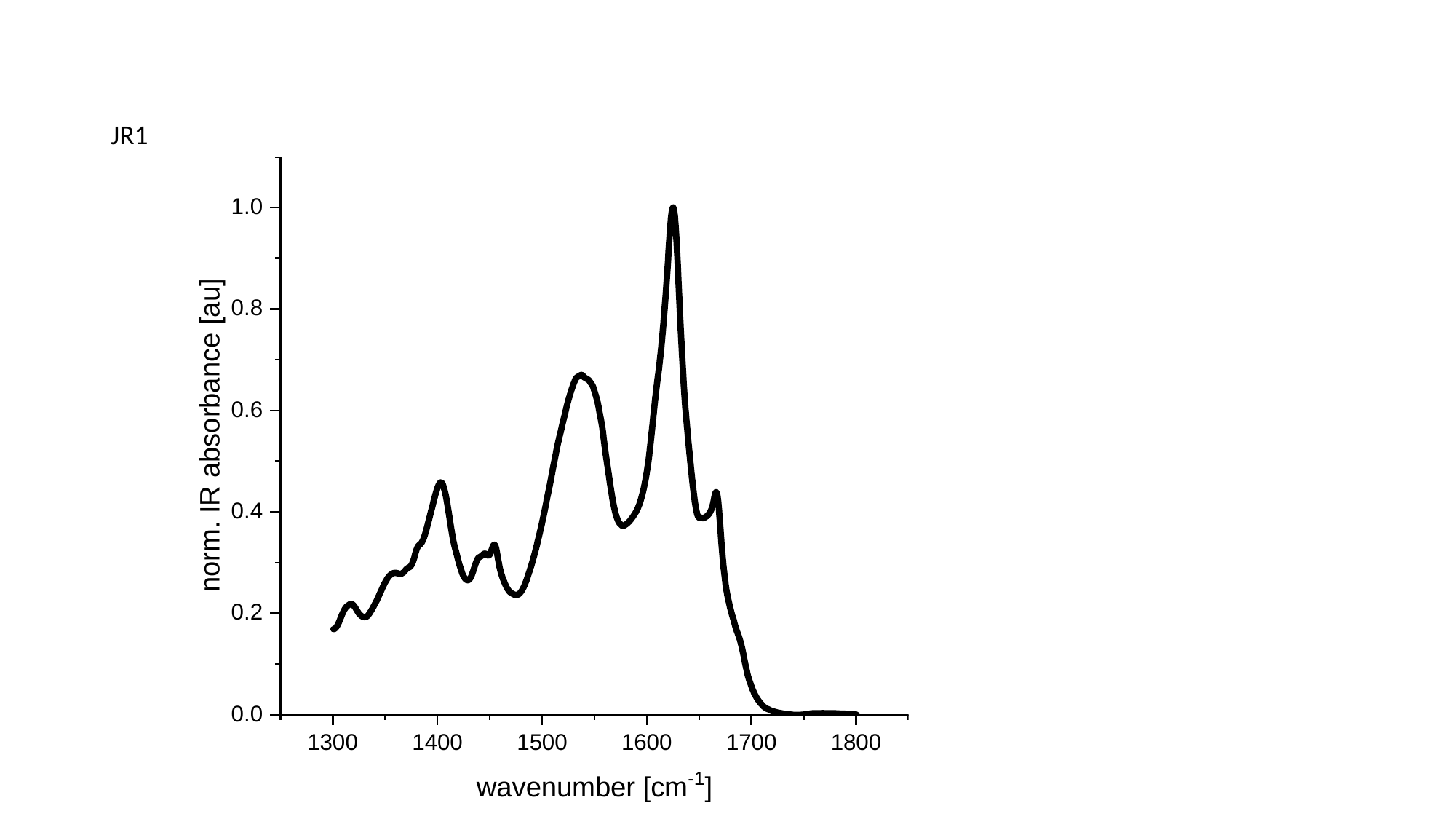

# JR1

## Slide 82
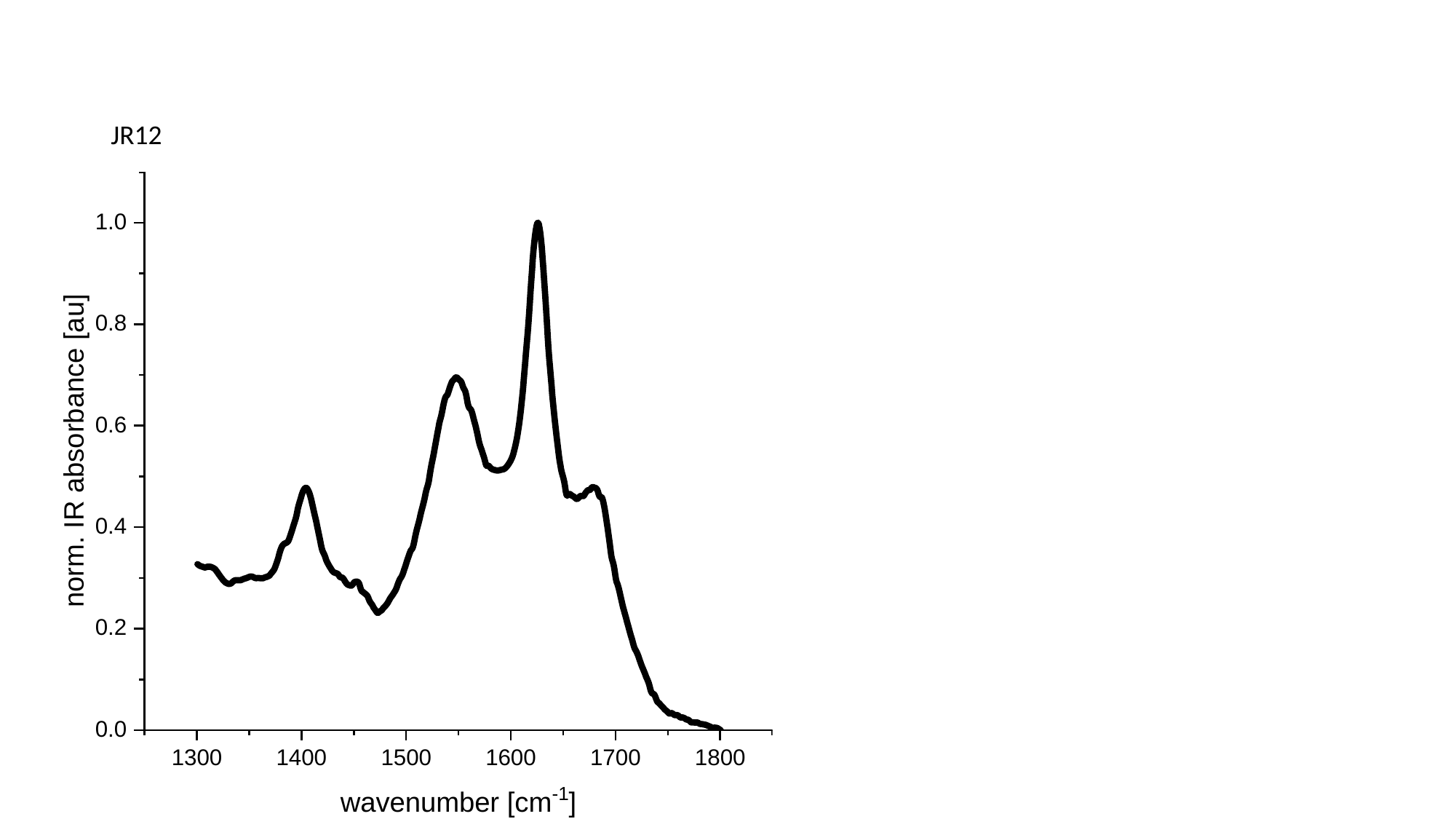

# JR12

## Slide 83
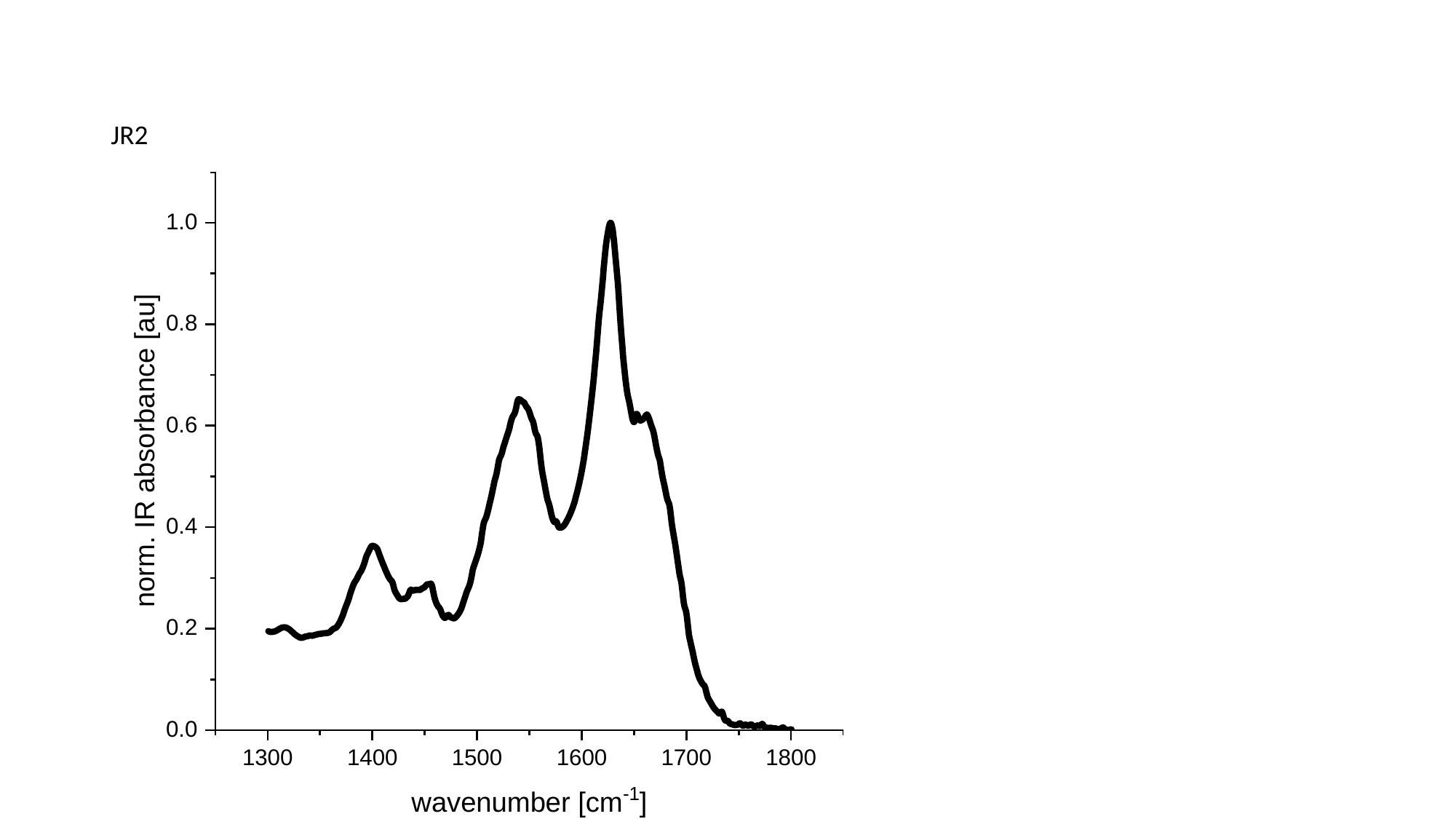

# JR2

## Slide 84
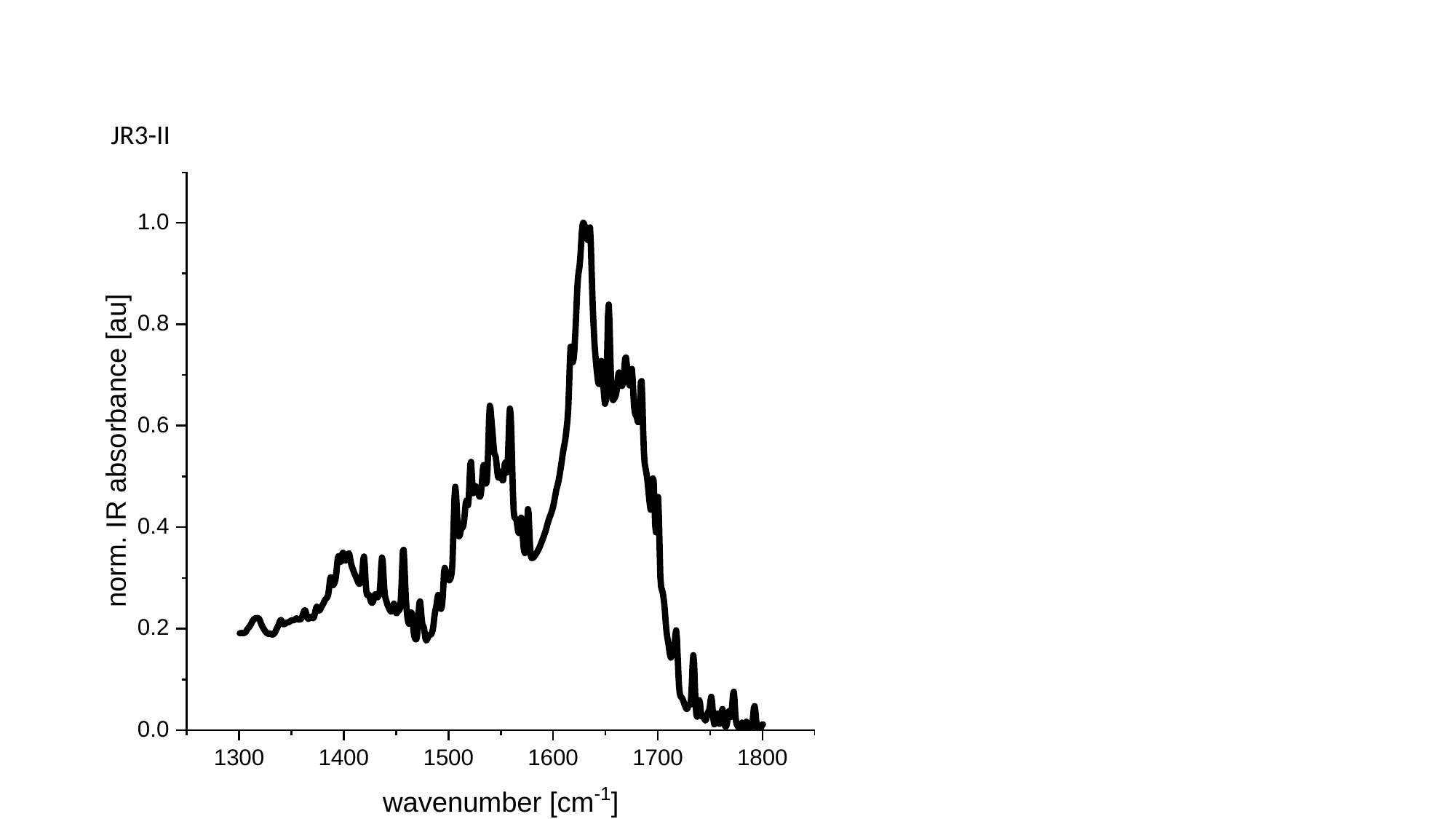

# JR3-II

## Slide 85
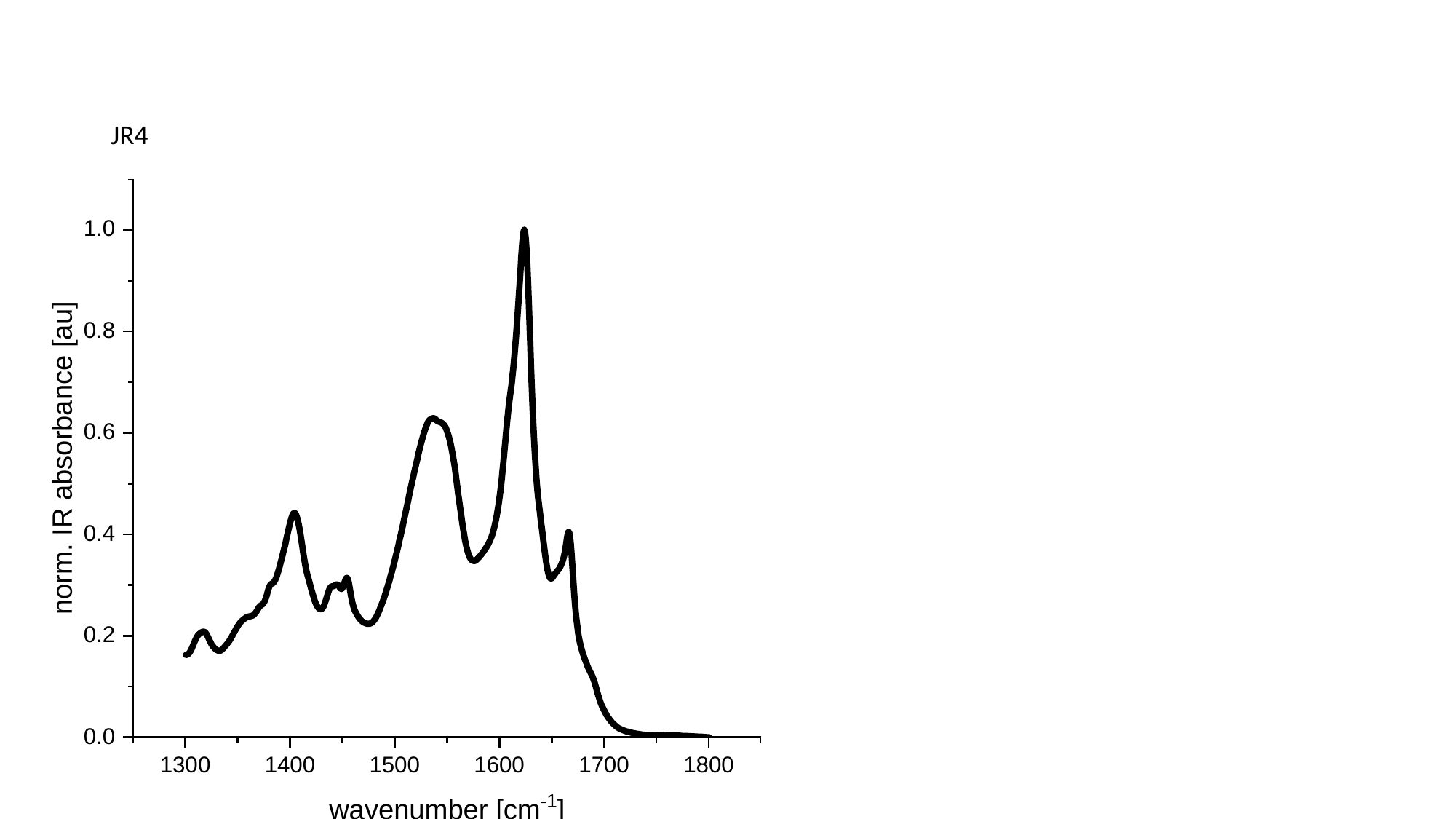

# JR4

## Slide 86
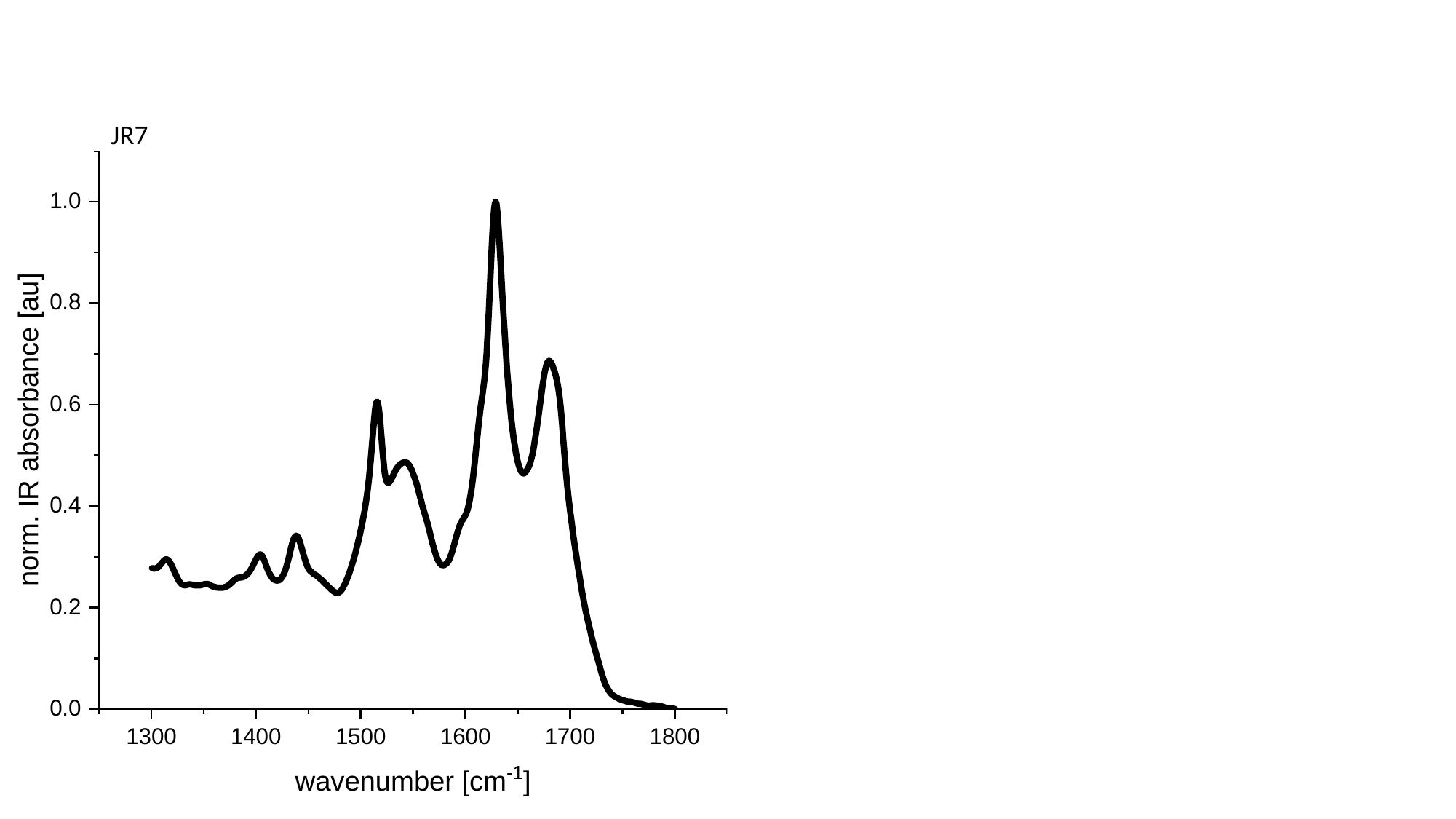

# JR7

## Slide 87
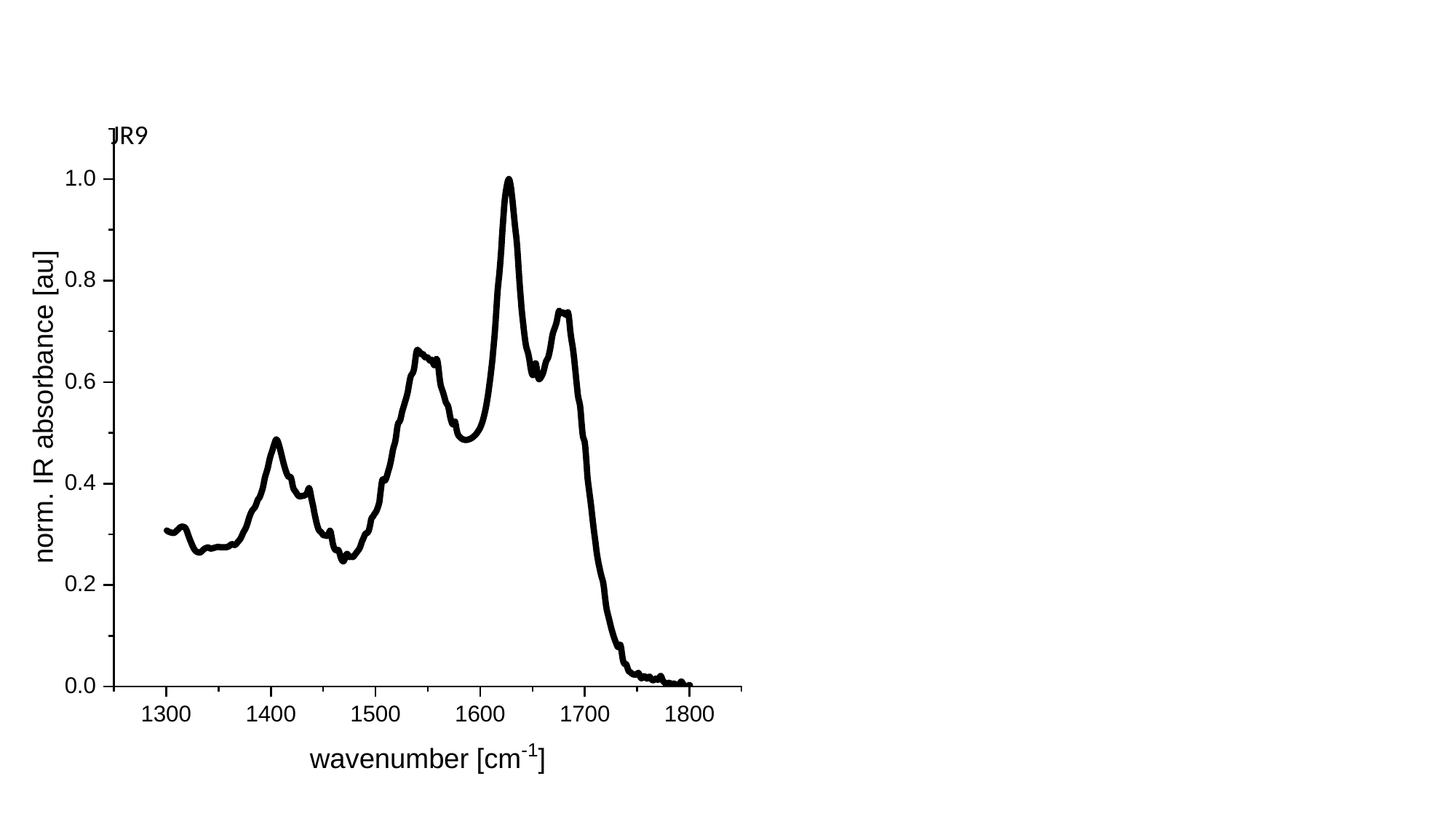

# JR9

## Slide 88
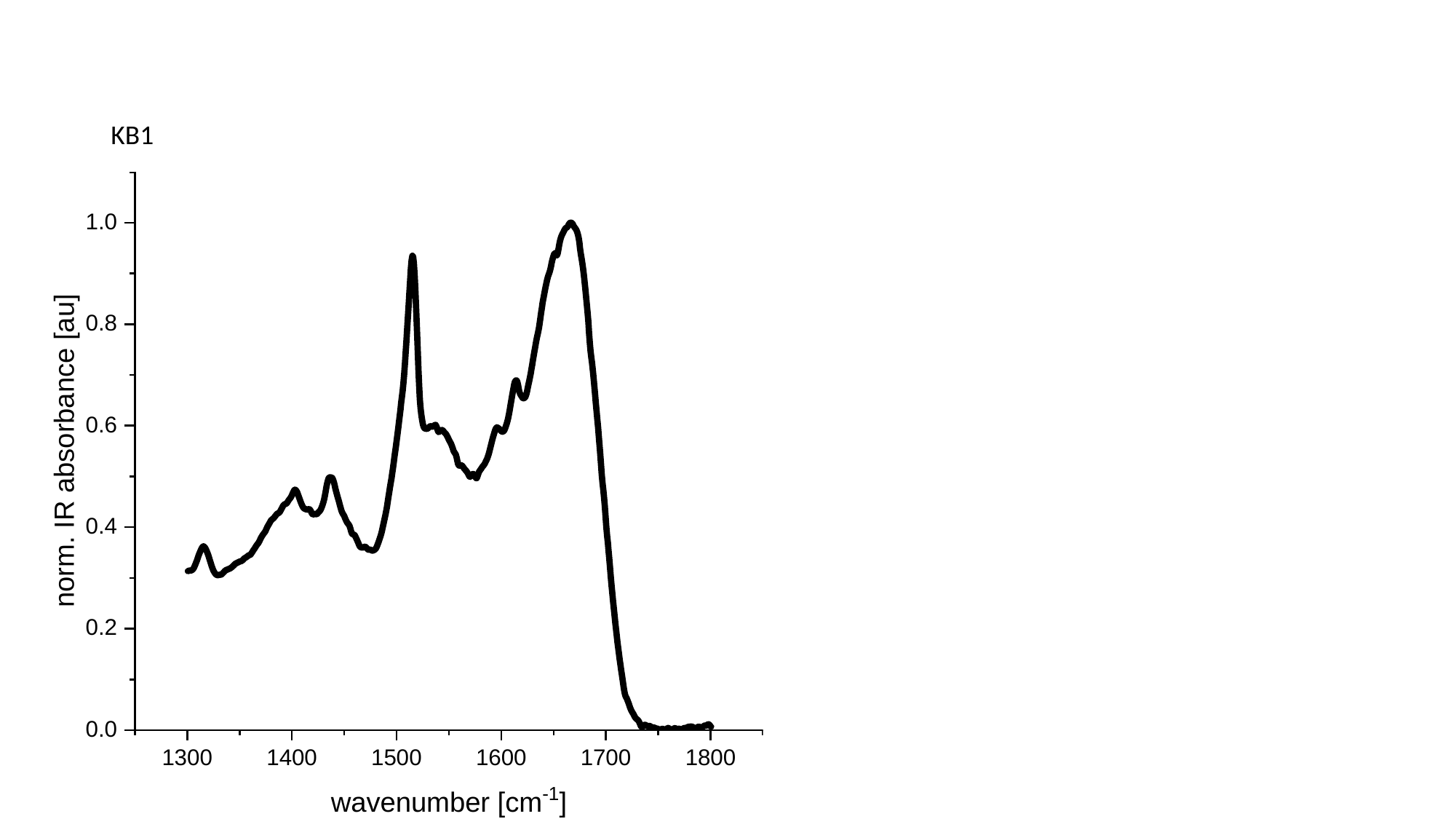

# KB1

## Slide 89
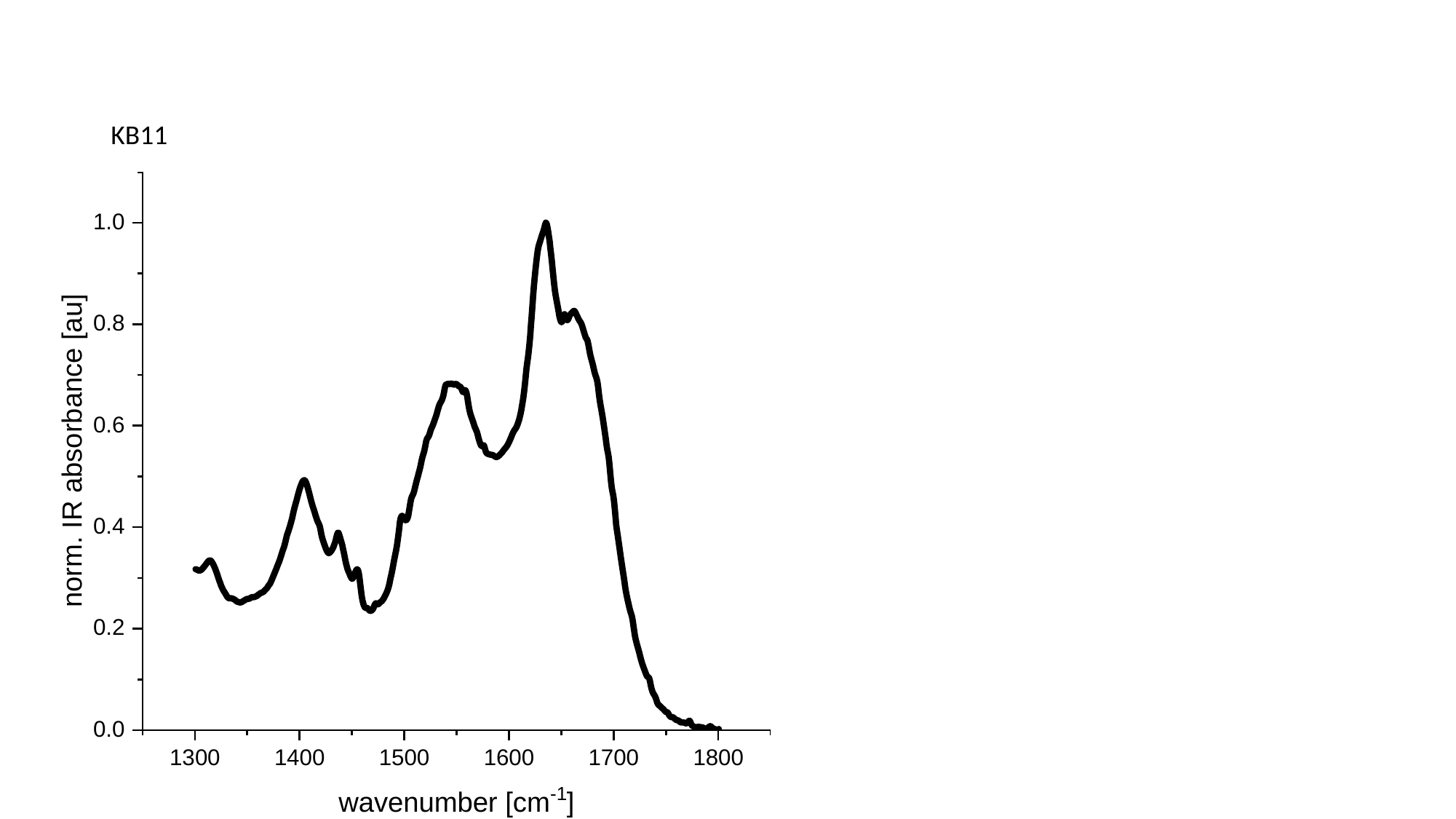

# KB11

## Slide 90
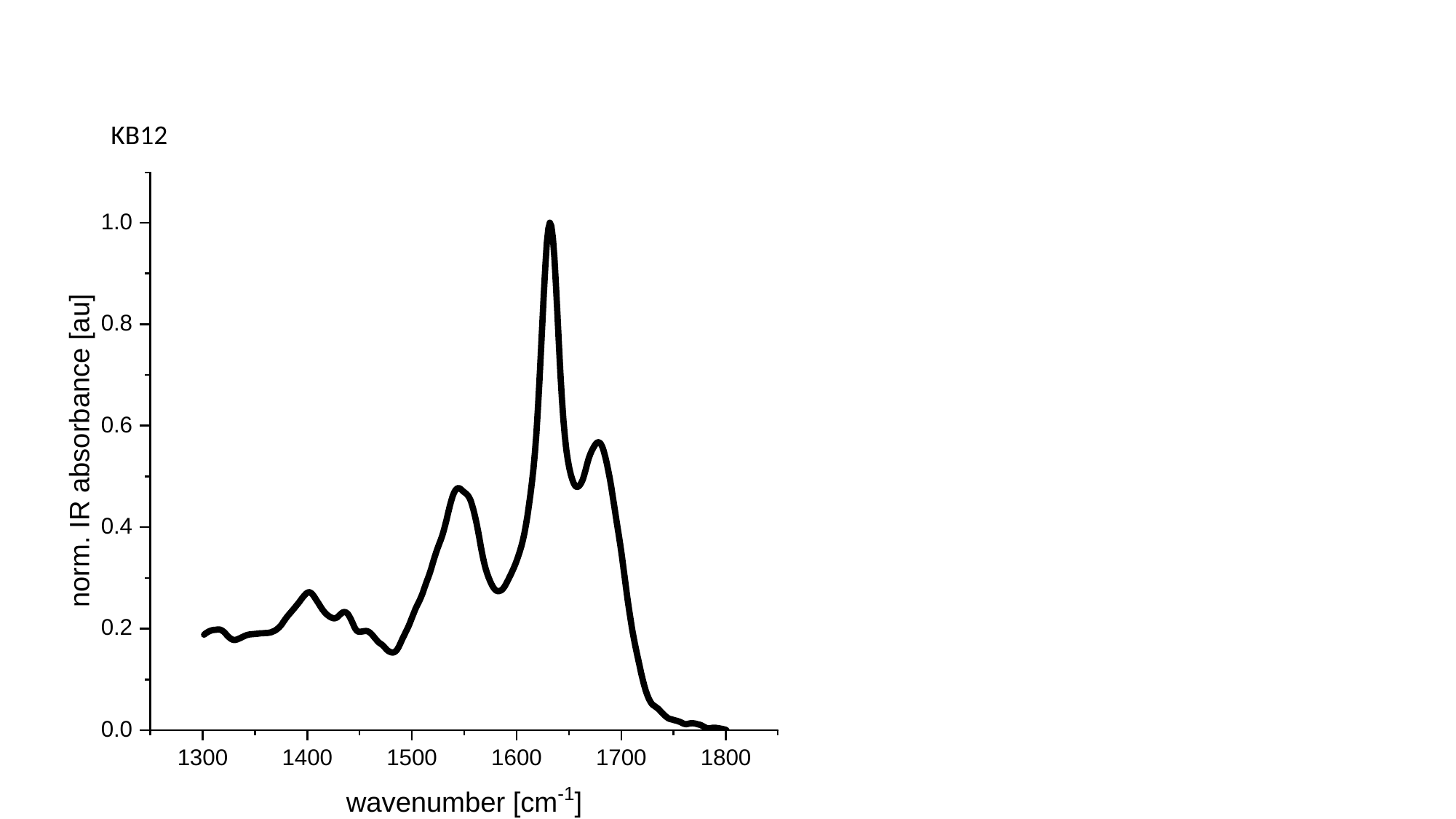

# KB12

## Slide 91
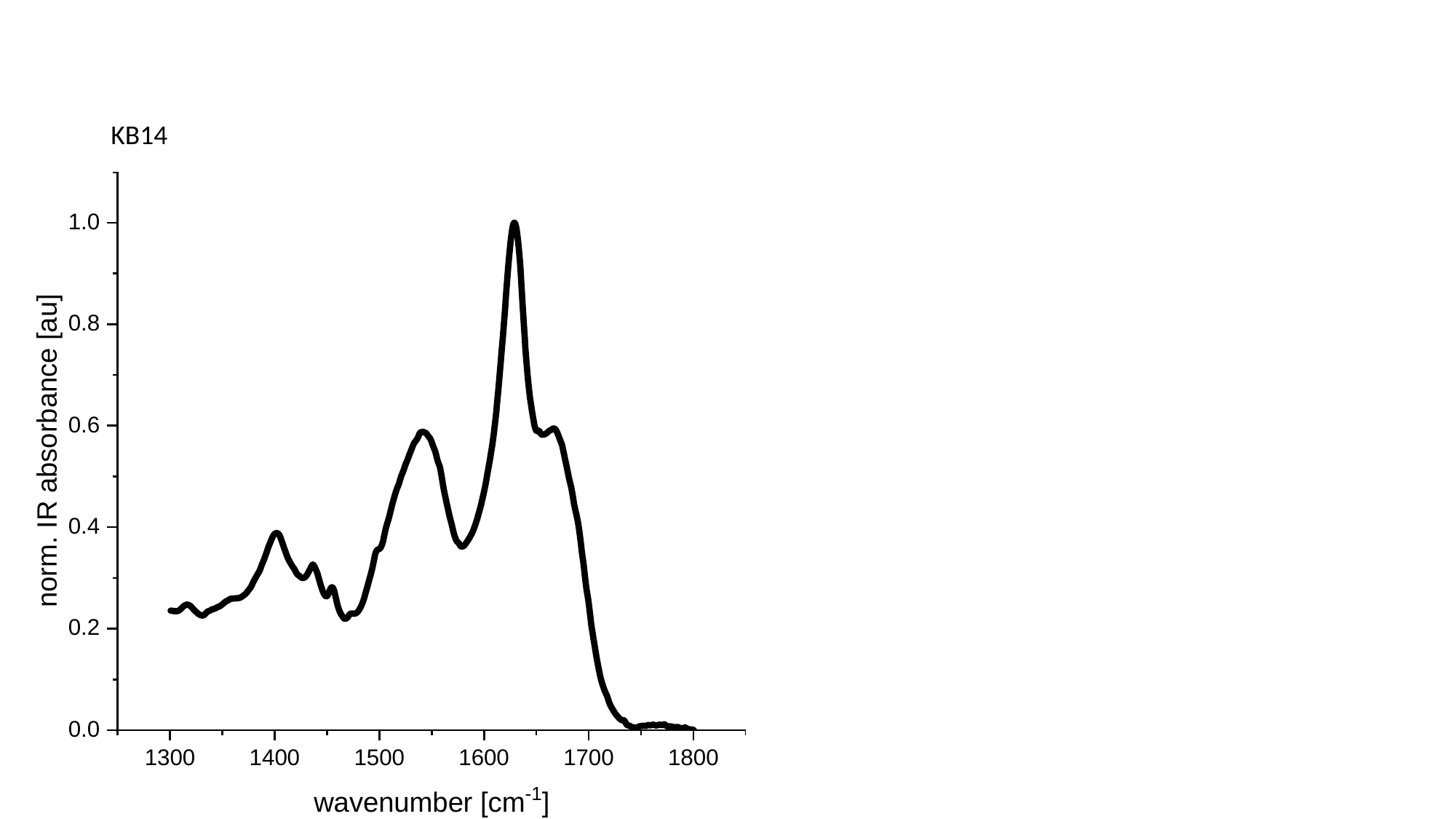

# KB14

## Slide 92
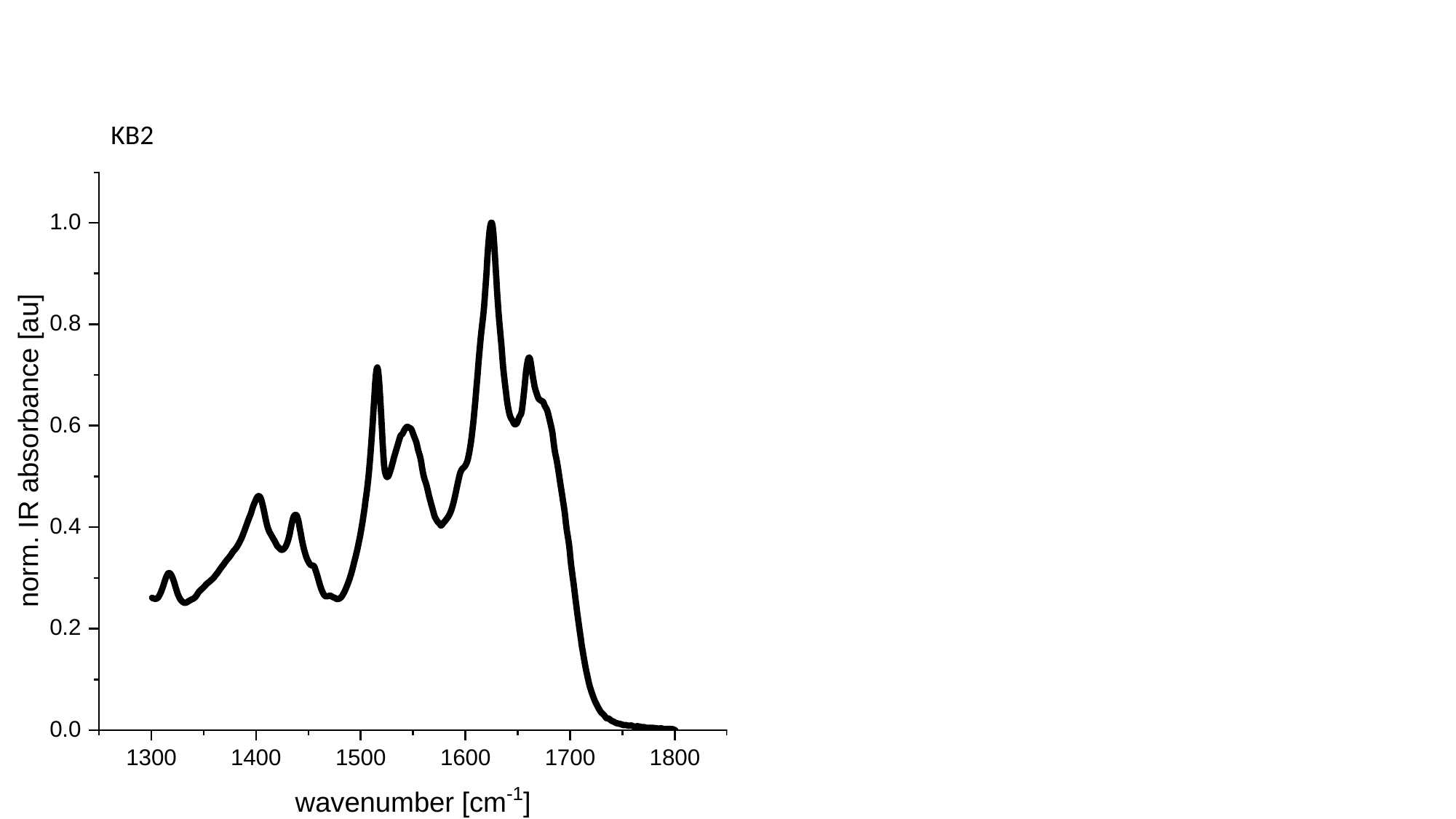

# KB2

## Slide 93
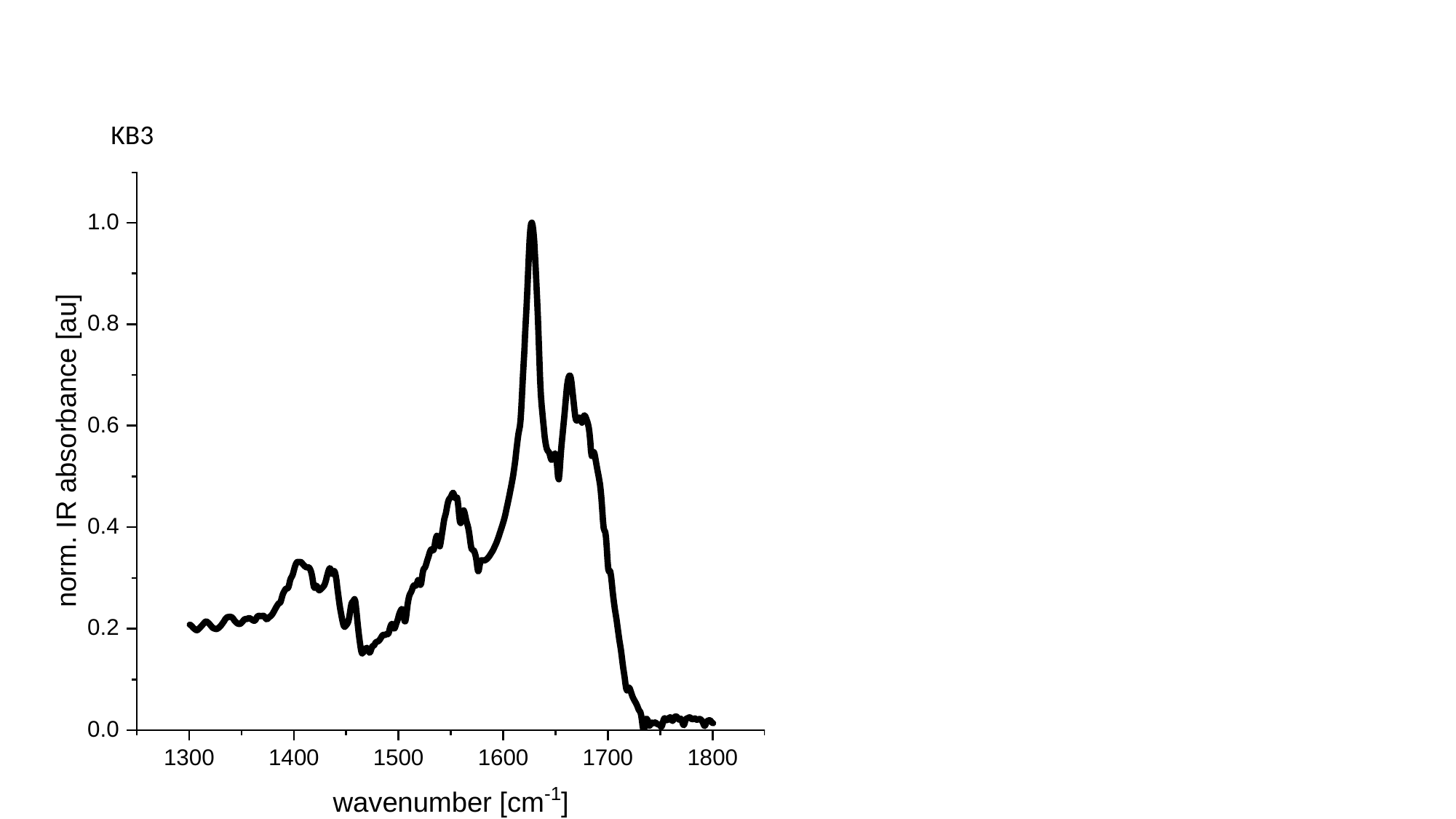

# KB3

## Slide 94
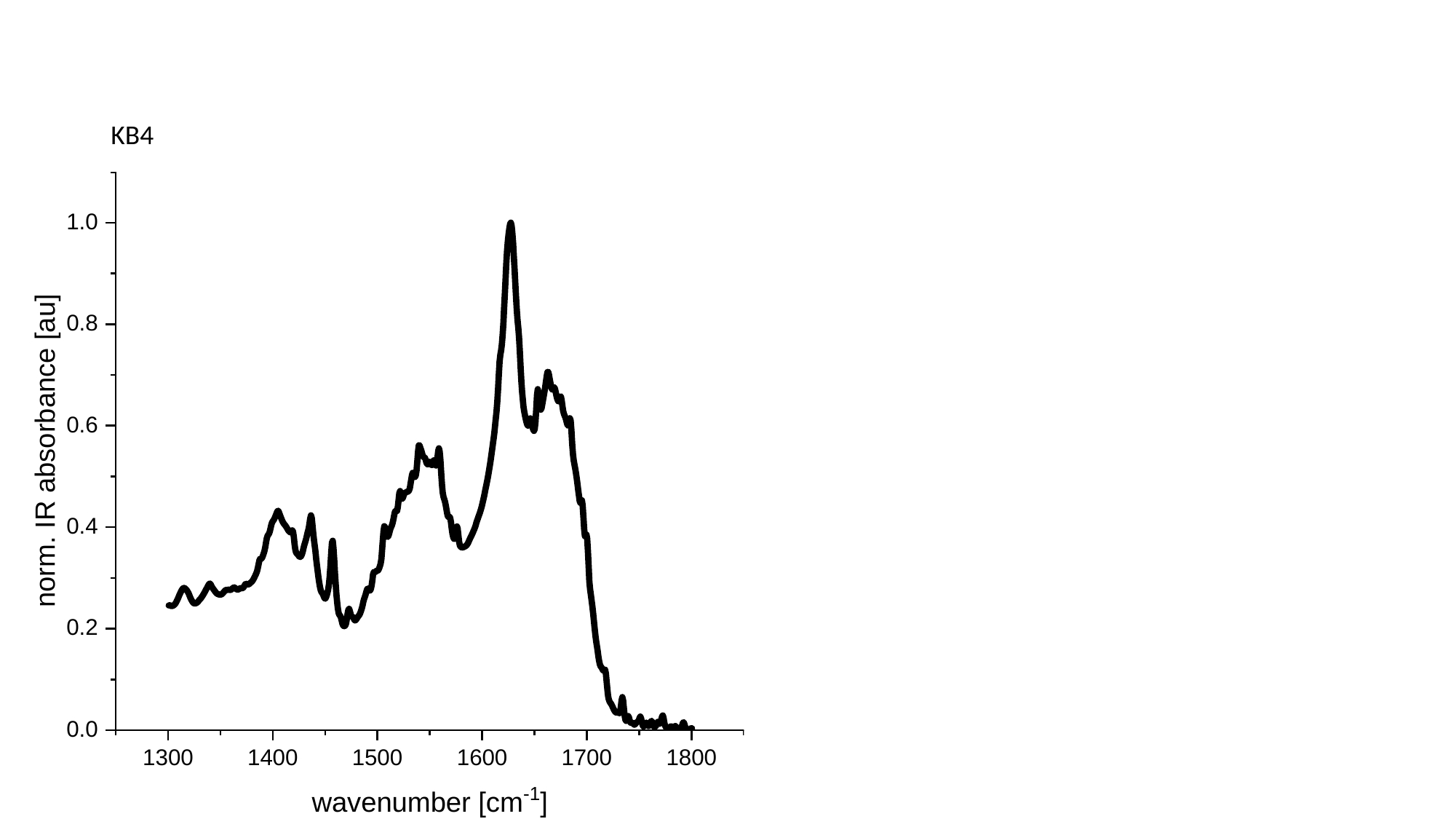

# KB4

## Slide 95
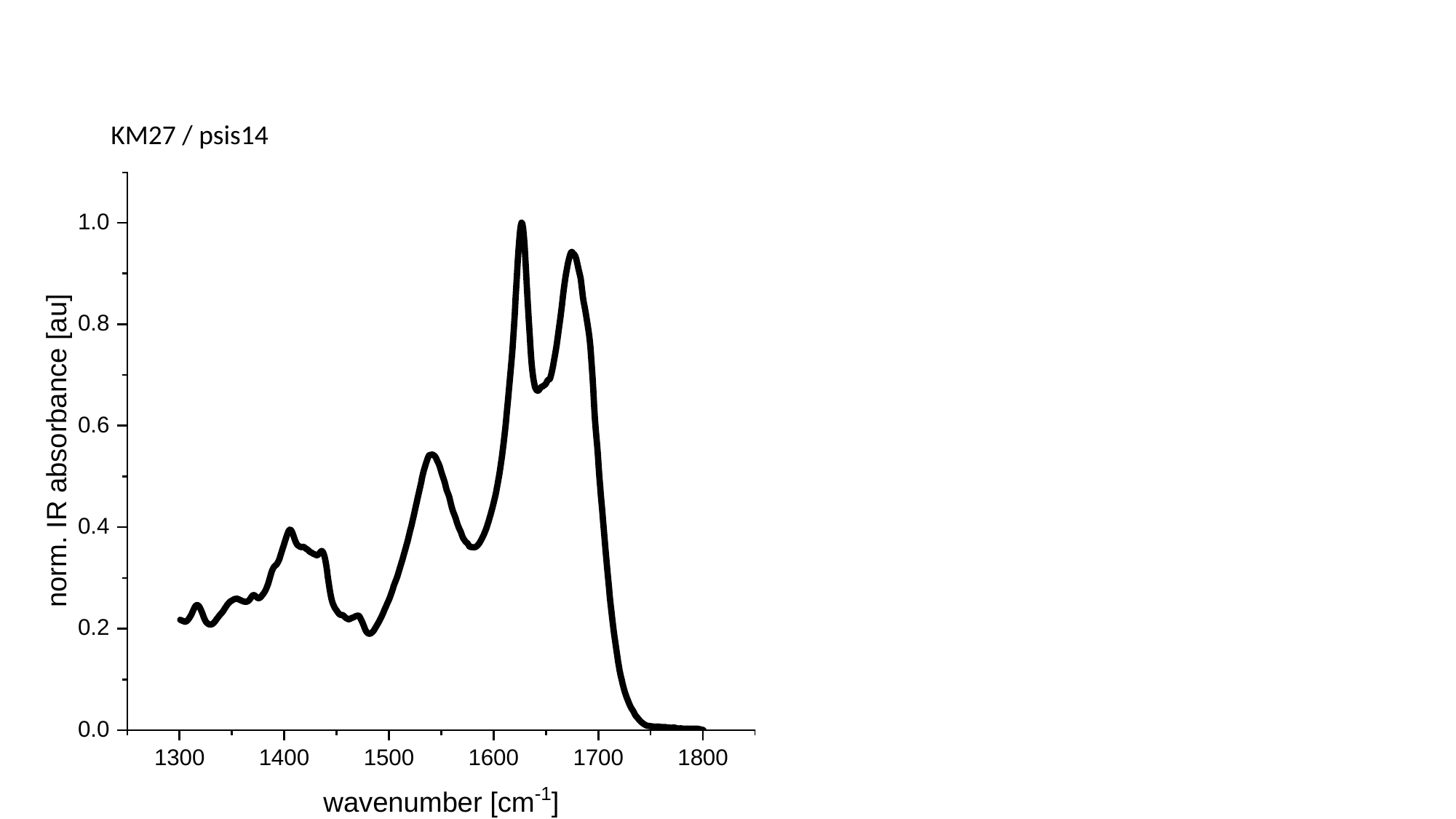

# KM27 / psis14

## Slide 96
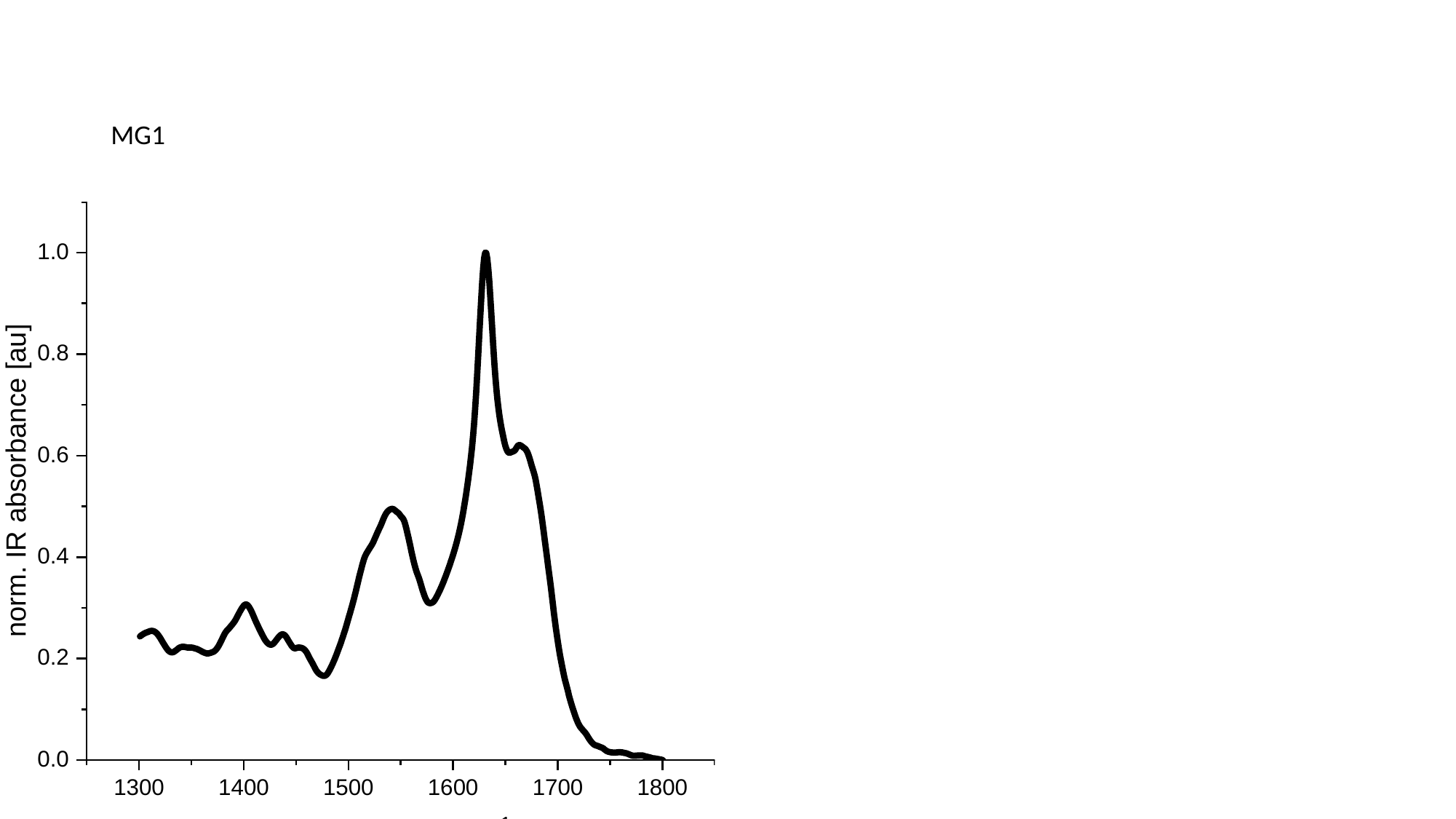

# MG1

## Slide 97
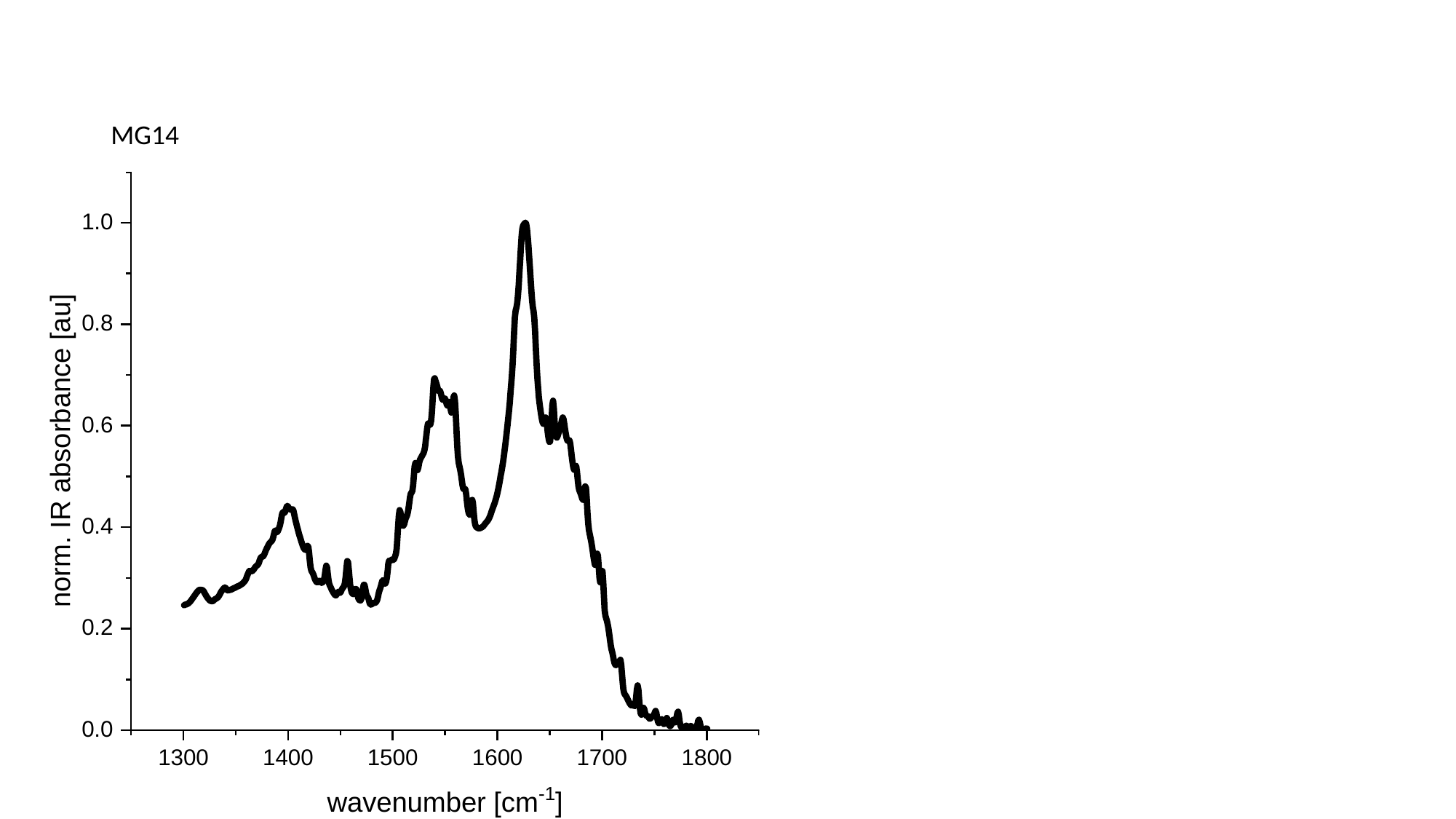

# MG14

## Slide 98
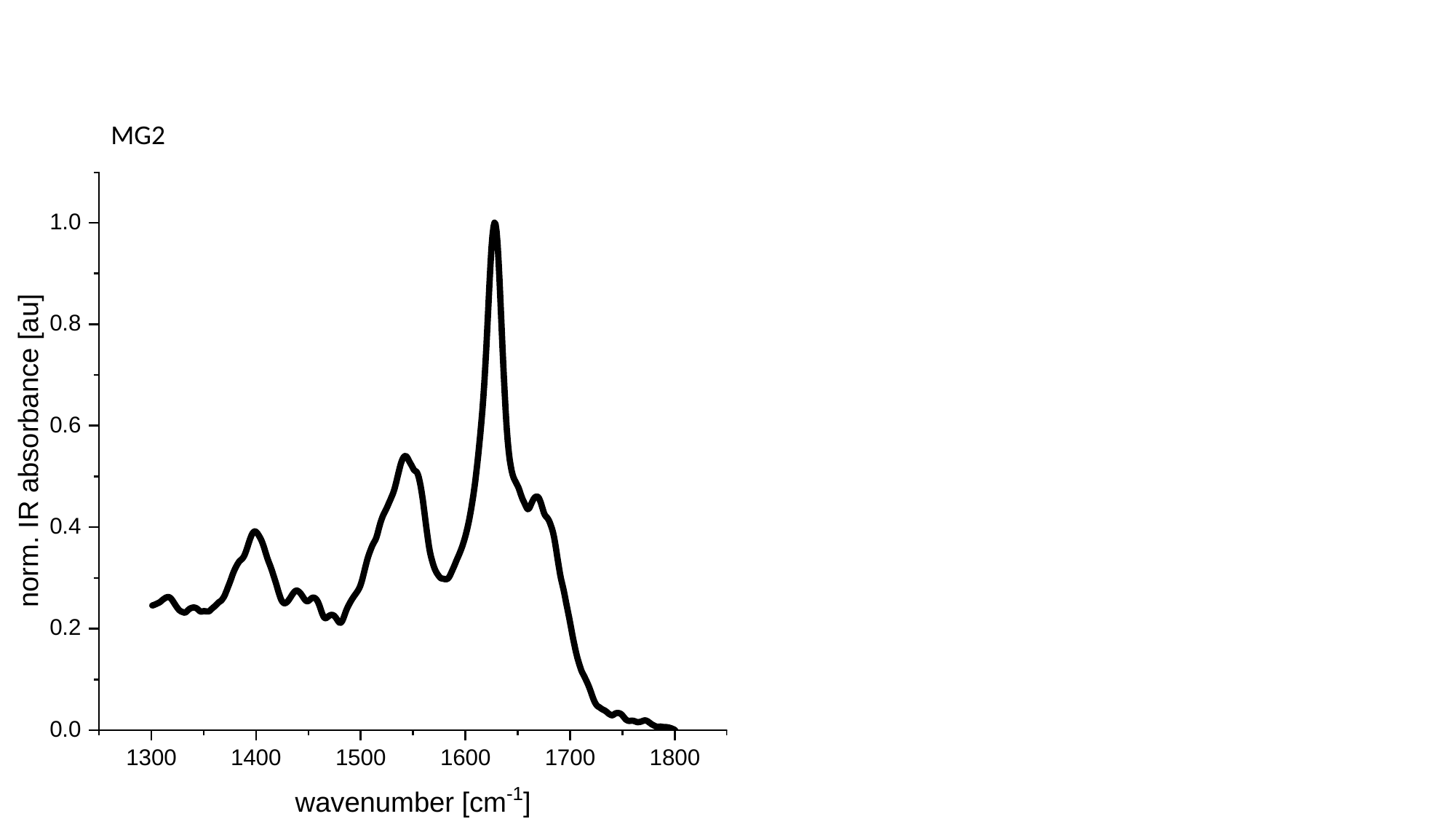

# MG2

## Slide 99
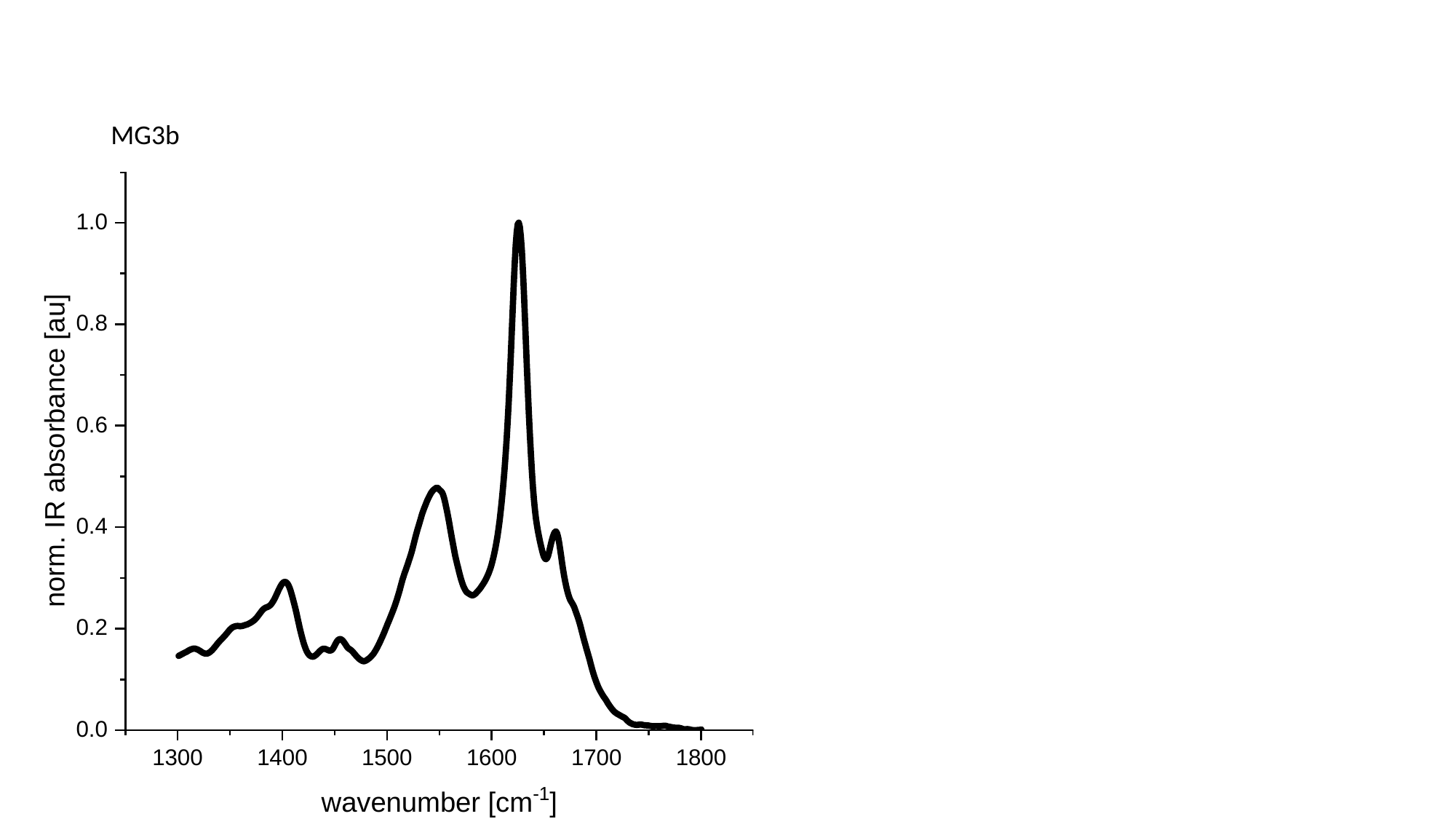

# MG3b

## Slide 100
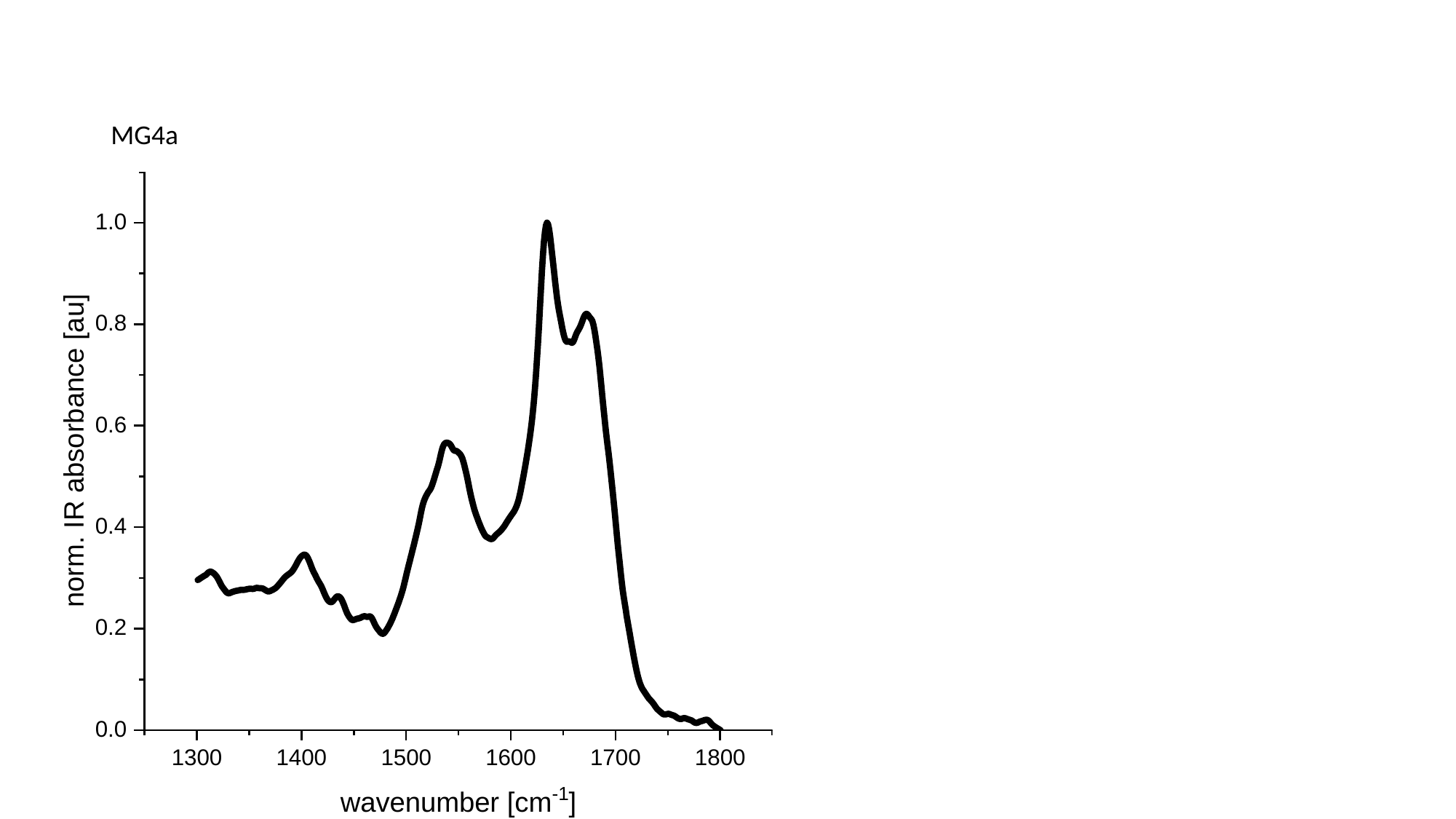

# MG4a

## Slide 101
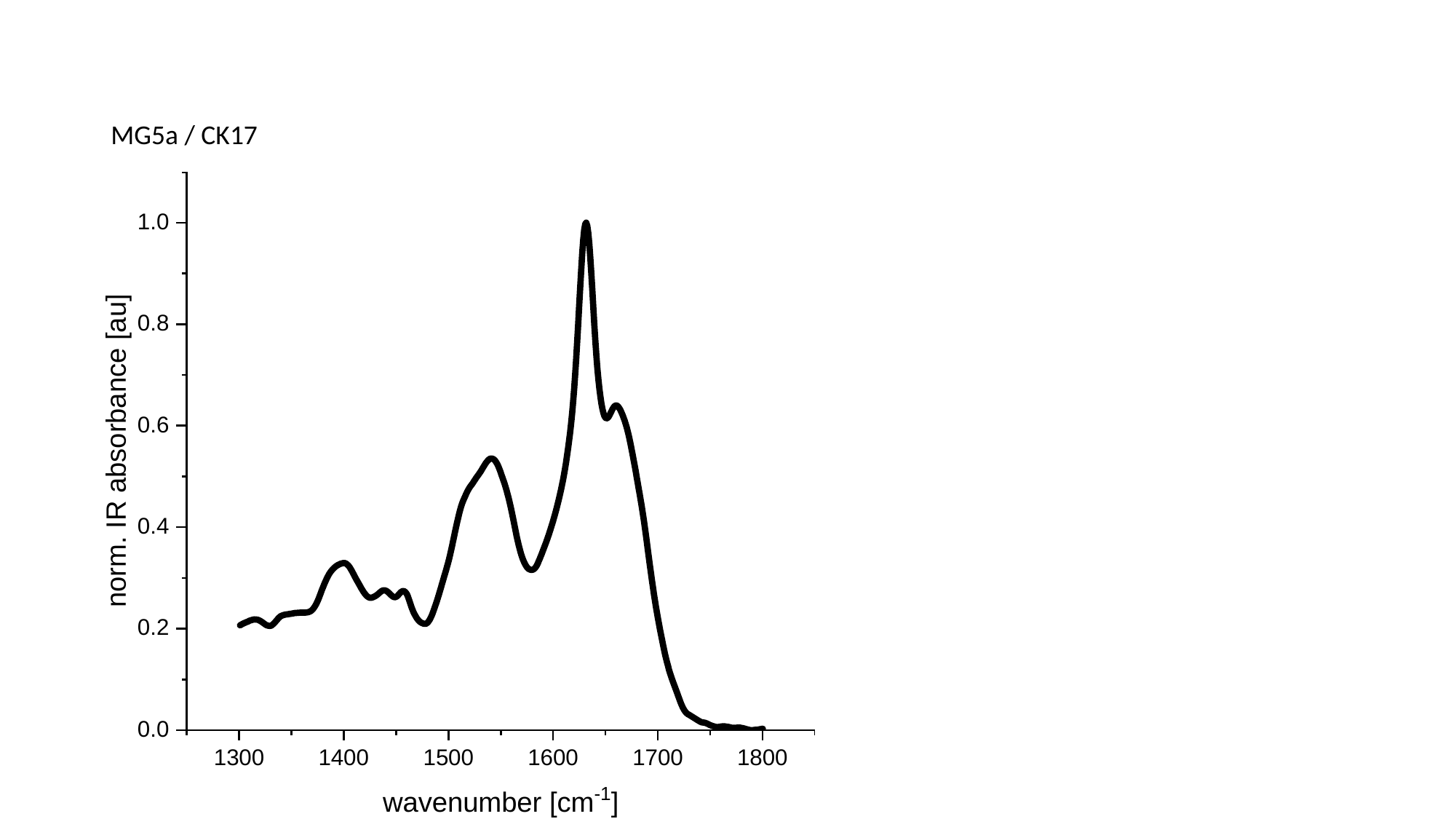

# MG5a / CK17

## Slide 102
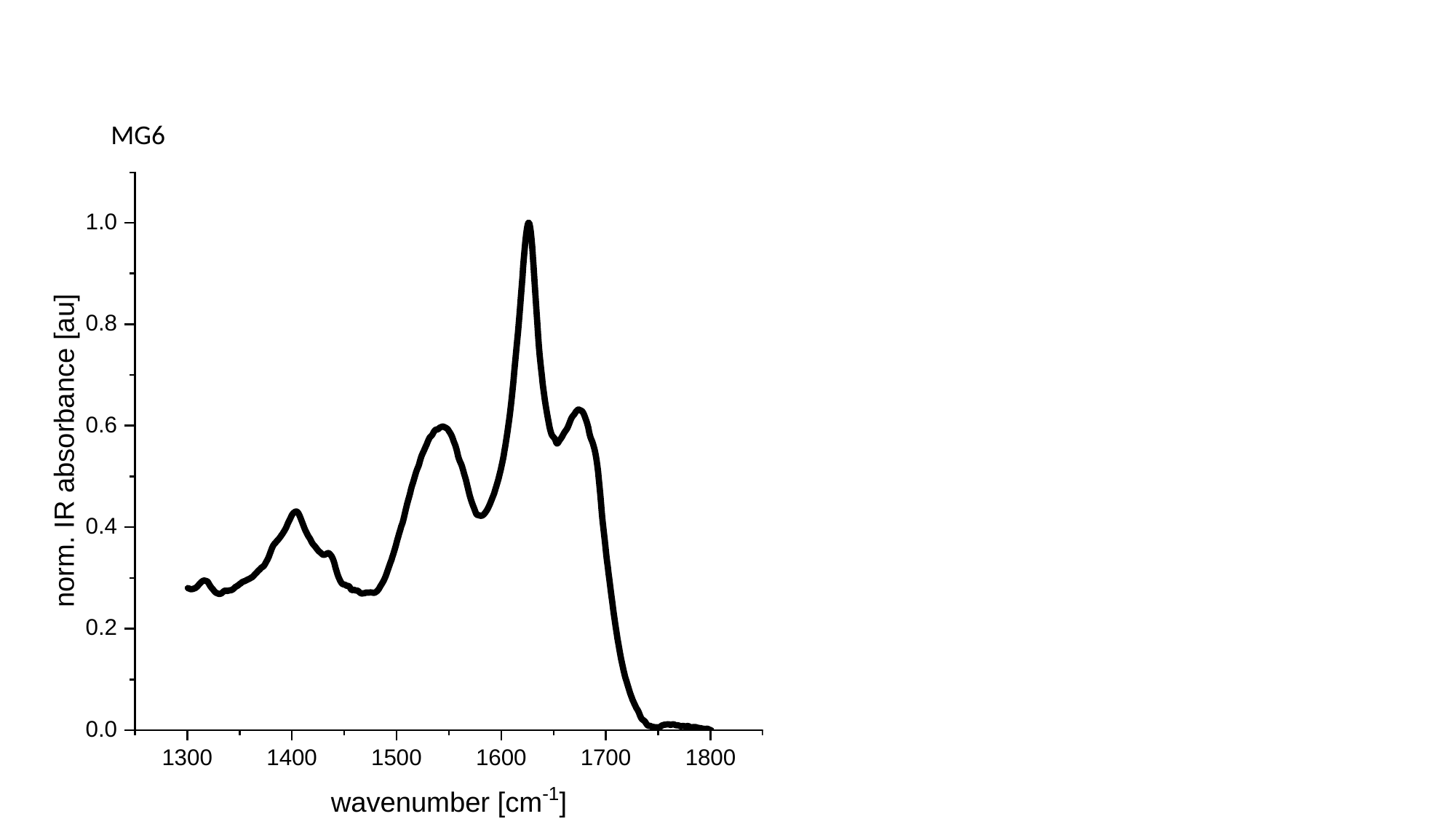

# MG6

## Slide 103
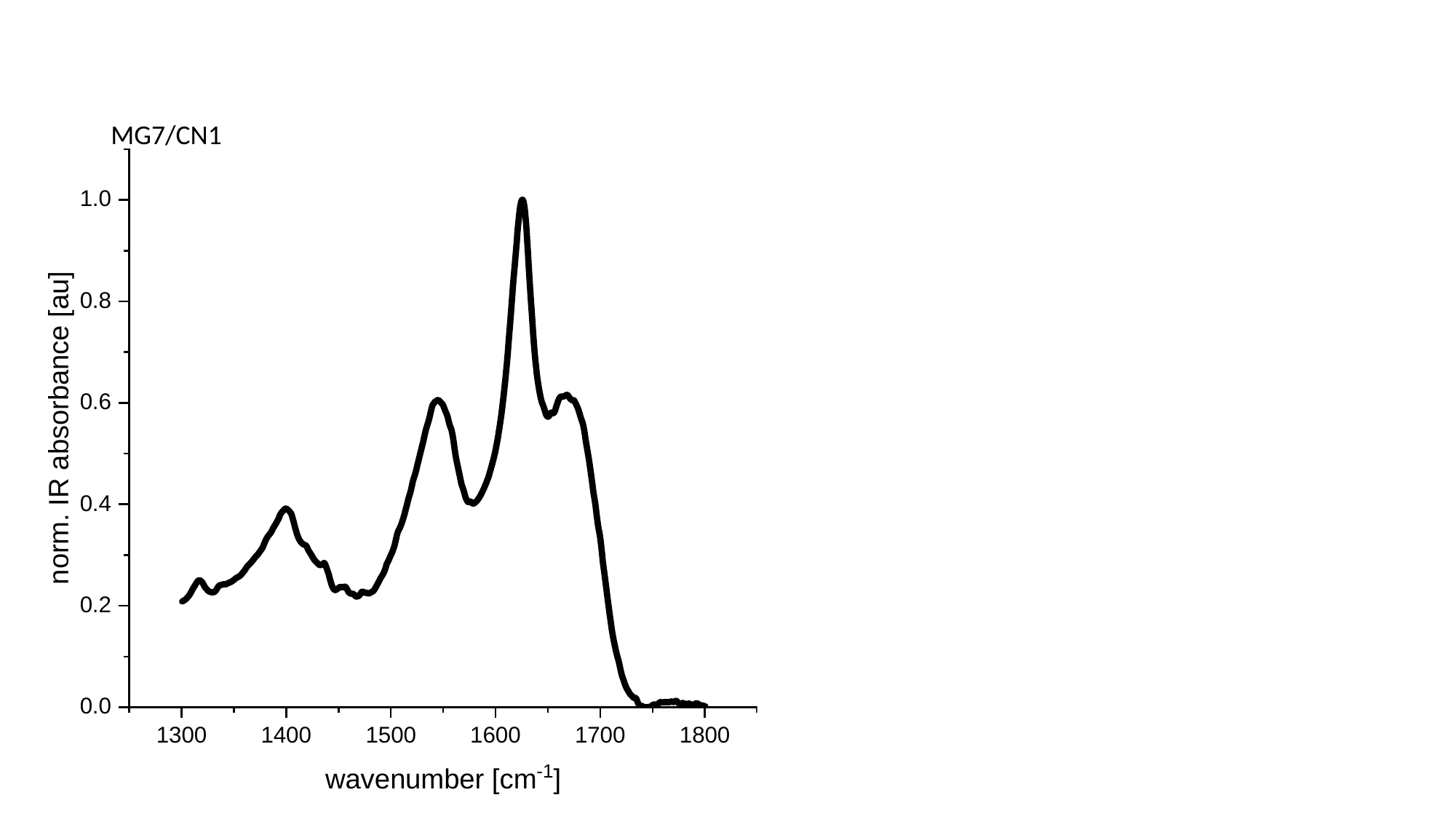

# MG7/CN1

## Slide 104
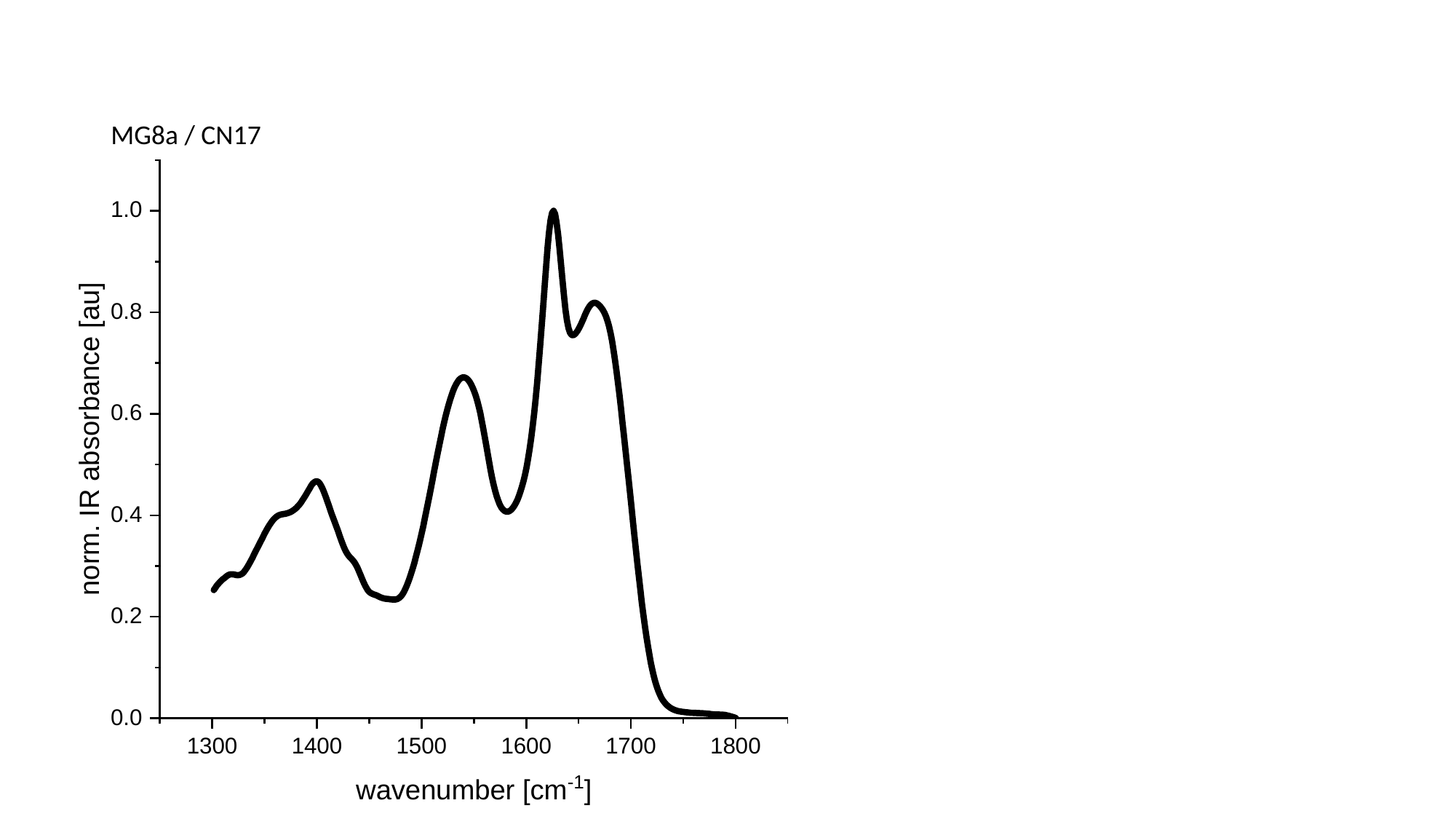

# MG8a / CN17

## Slide 105
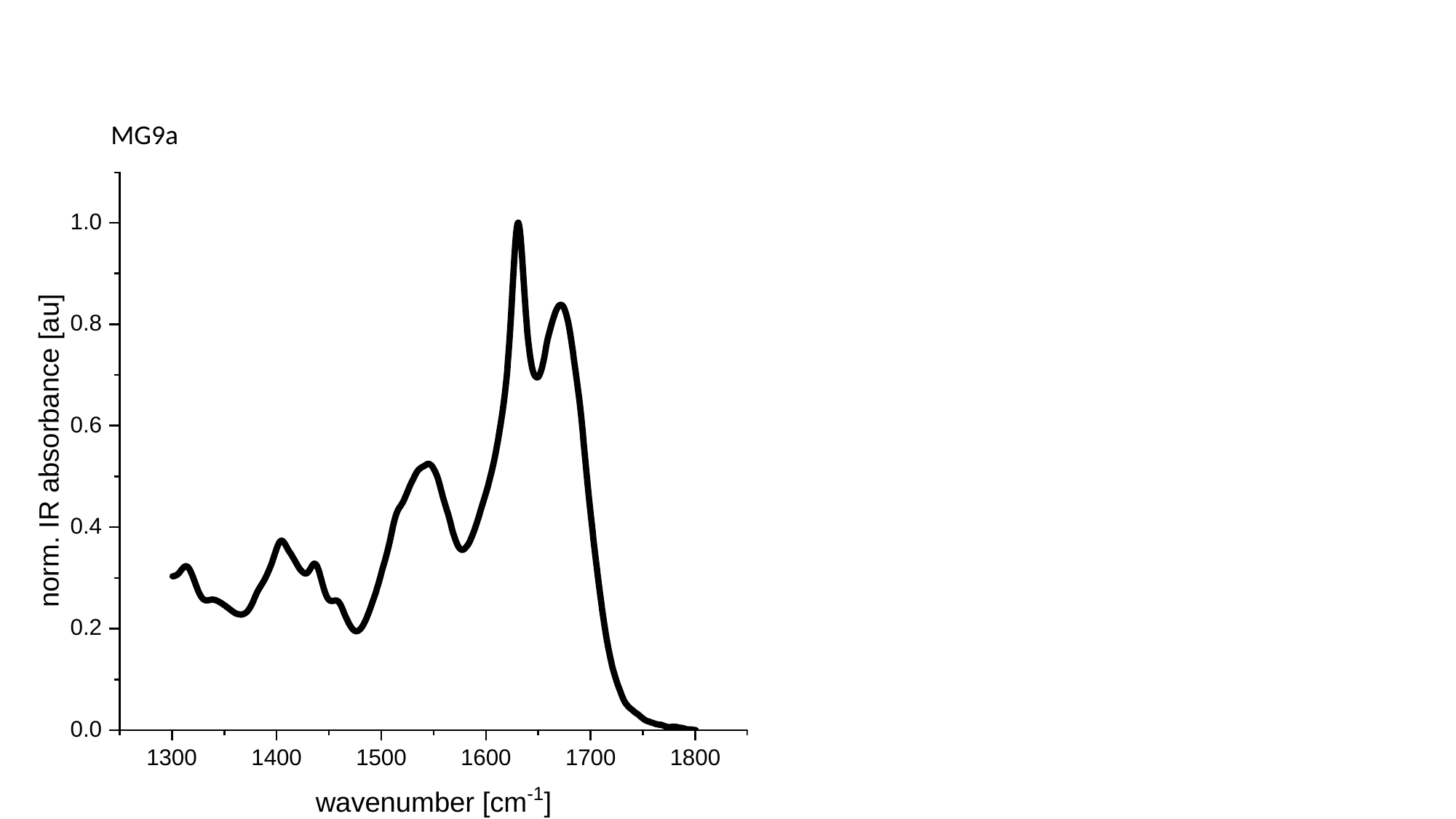

# MG9a

## Slide 106
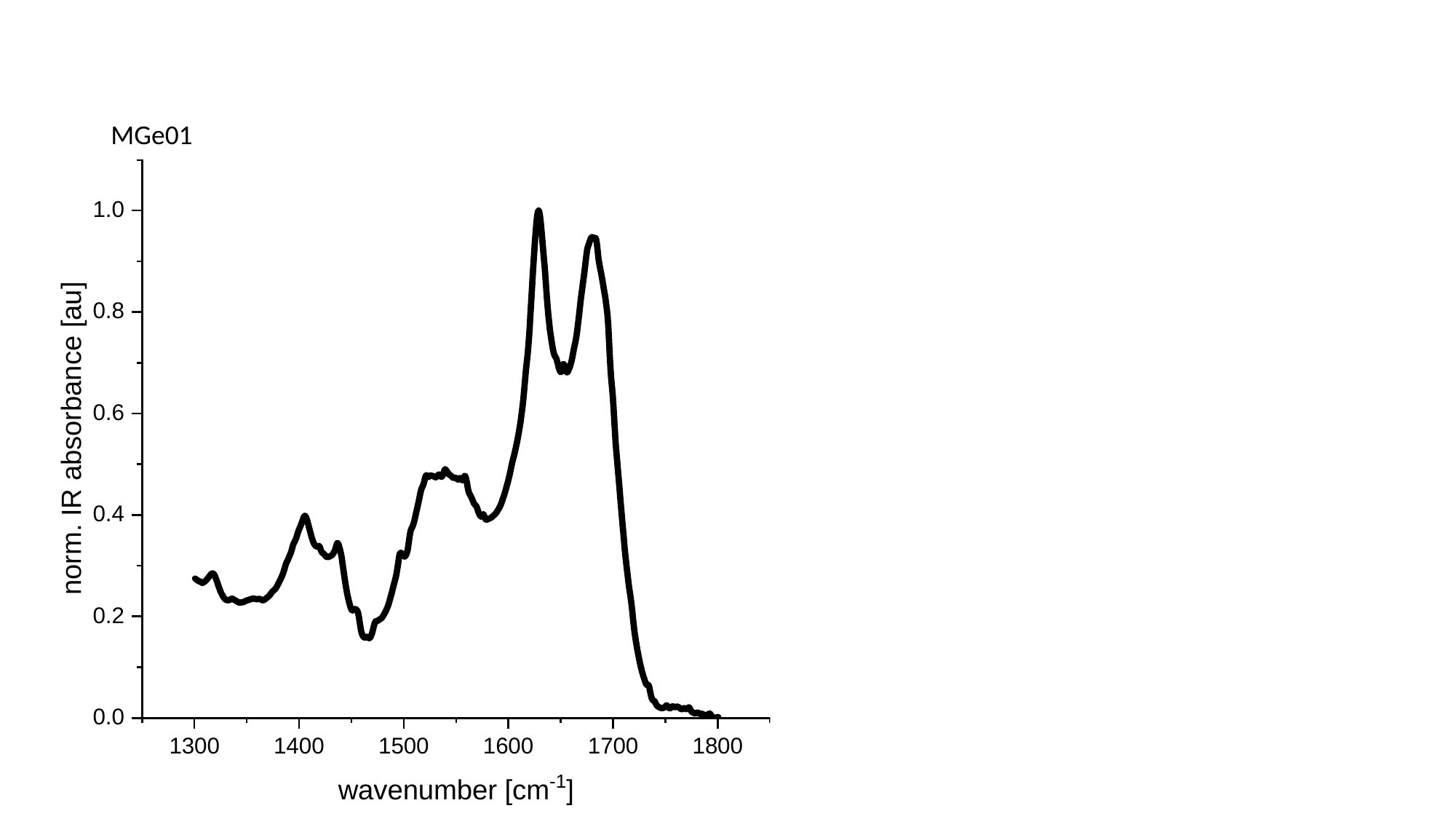

# MGe01

## Slide 107
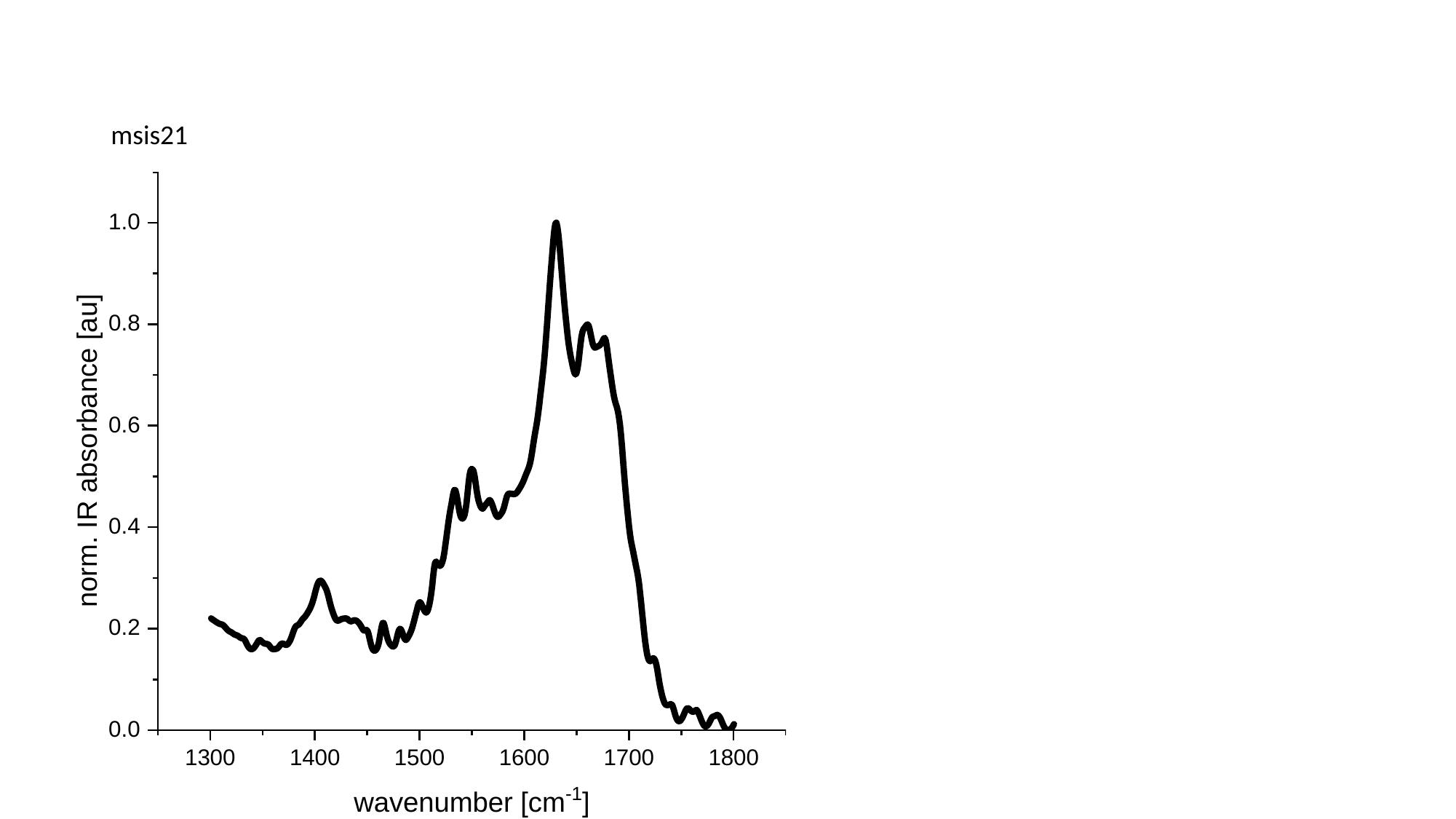

# msis21

## Slide 108
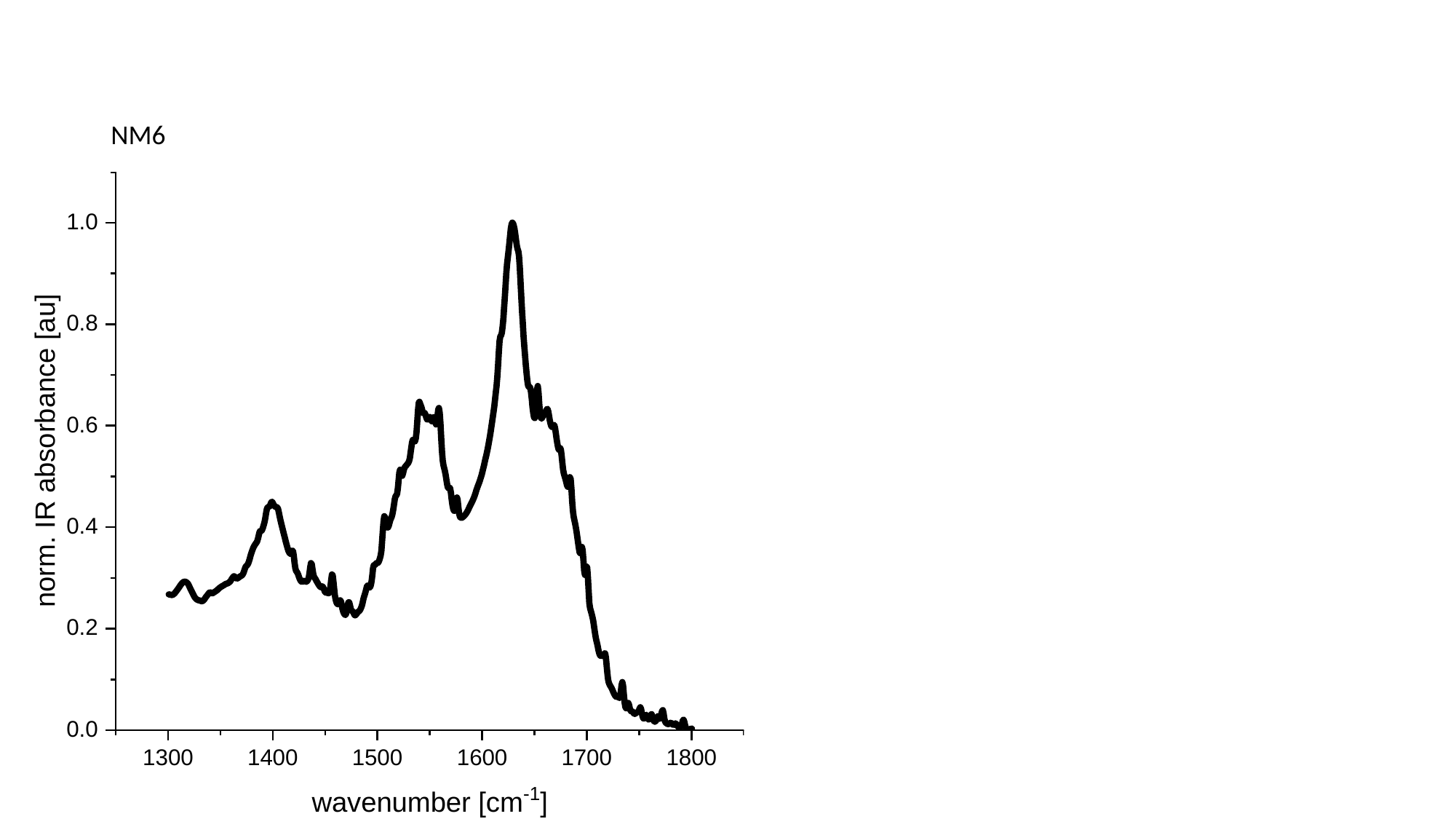

# NM6

## Slide 109
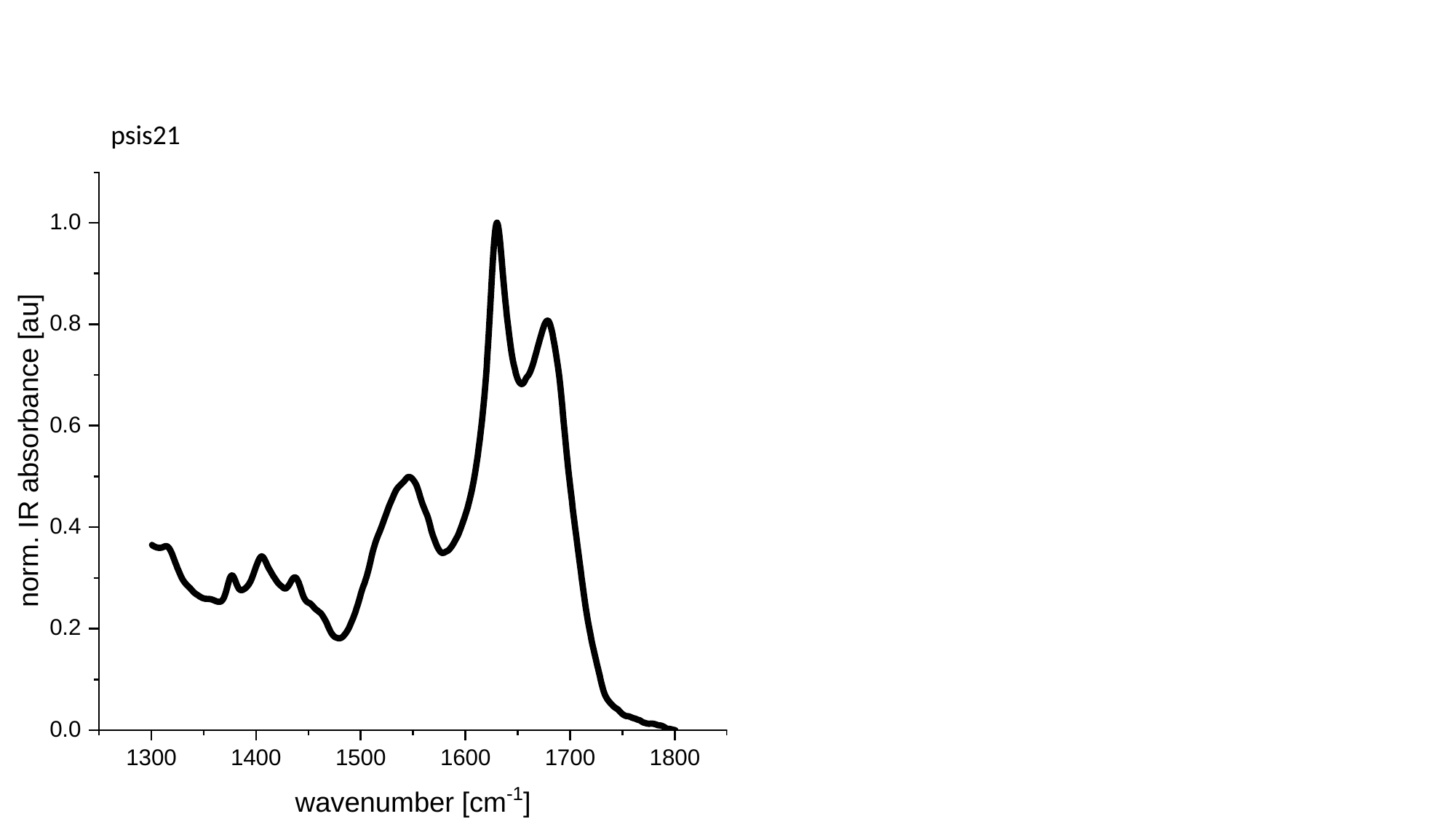

# psis21

## Slide 110
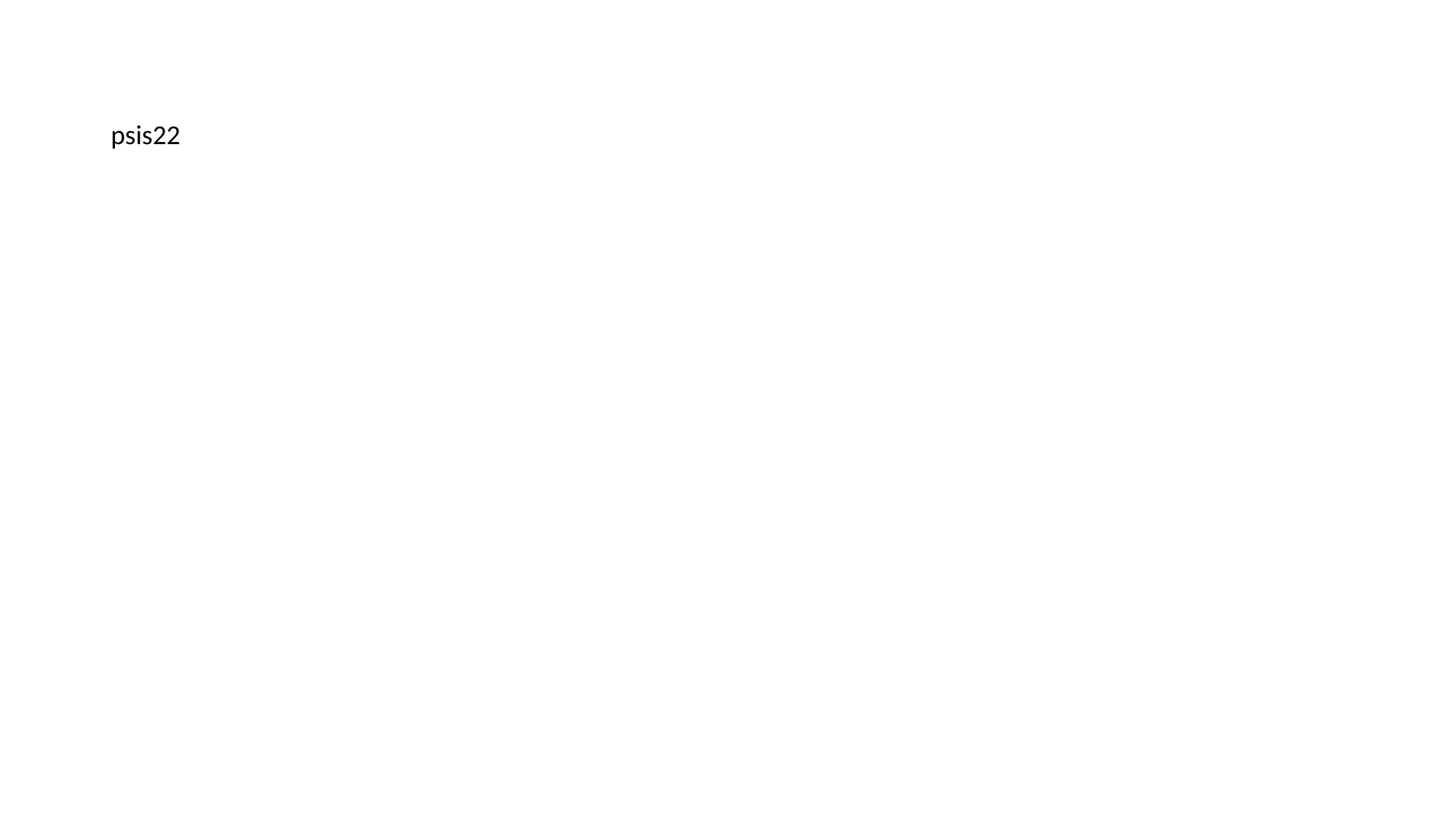

# psis22

## Slide 111
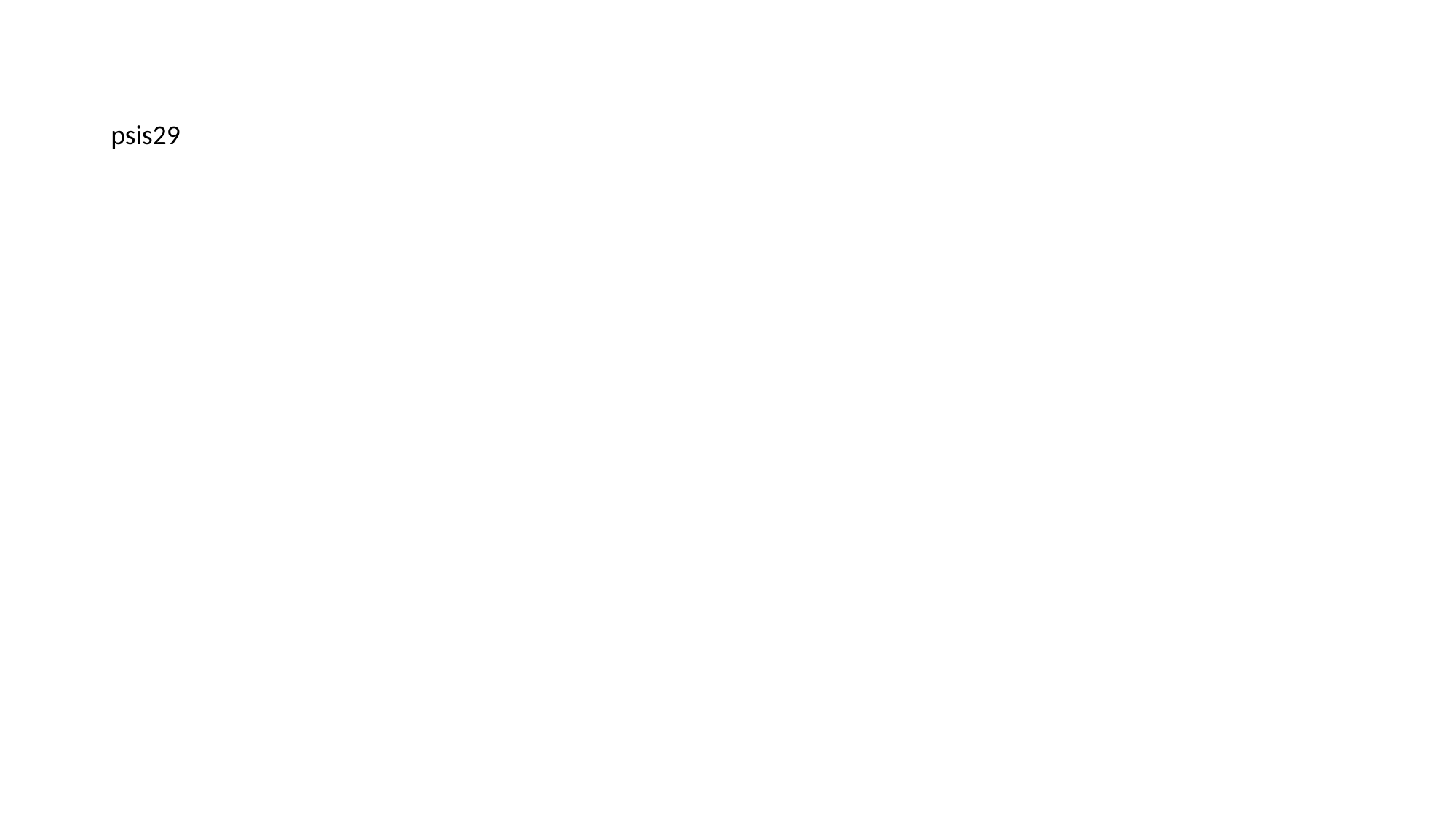

# psis29

## Slide 112
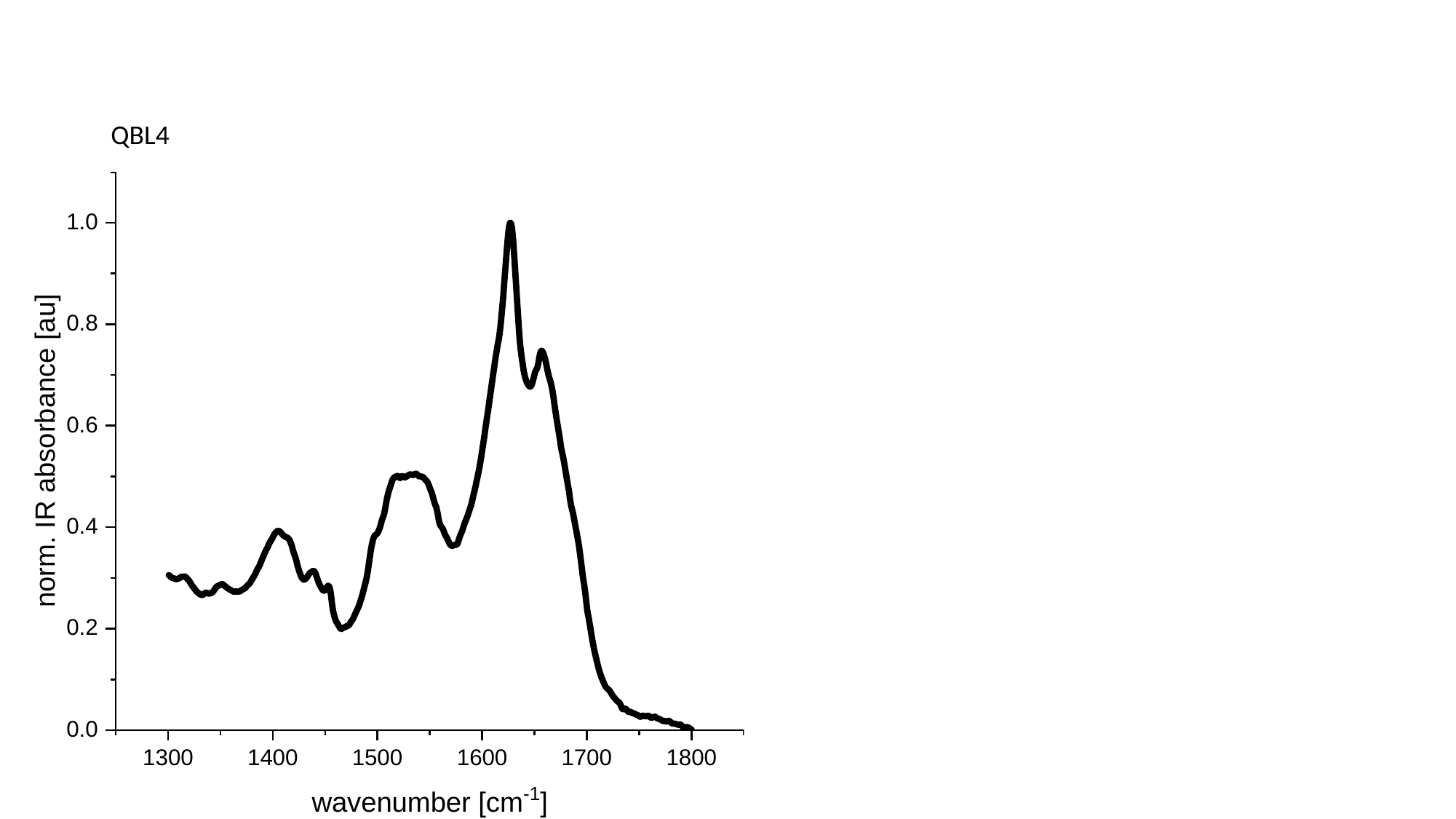

# QBL4

## Slide 113
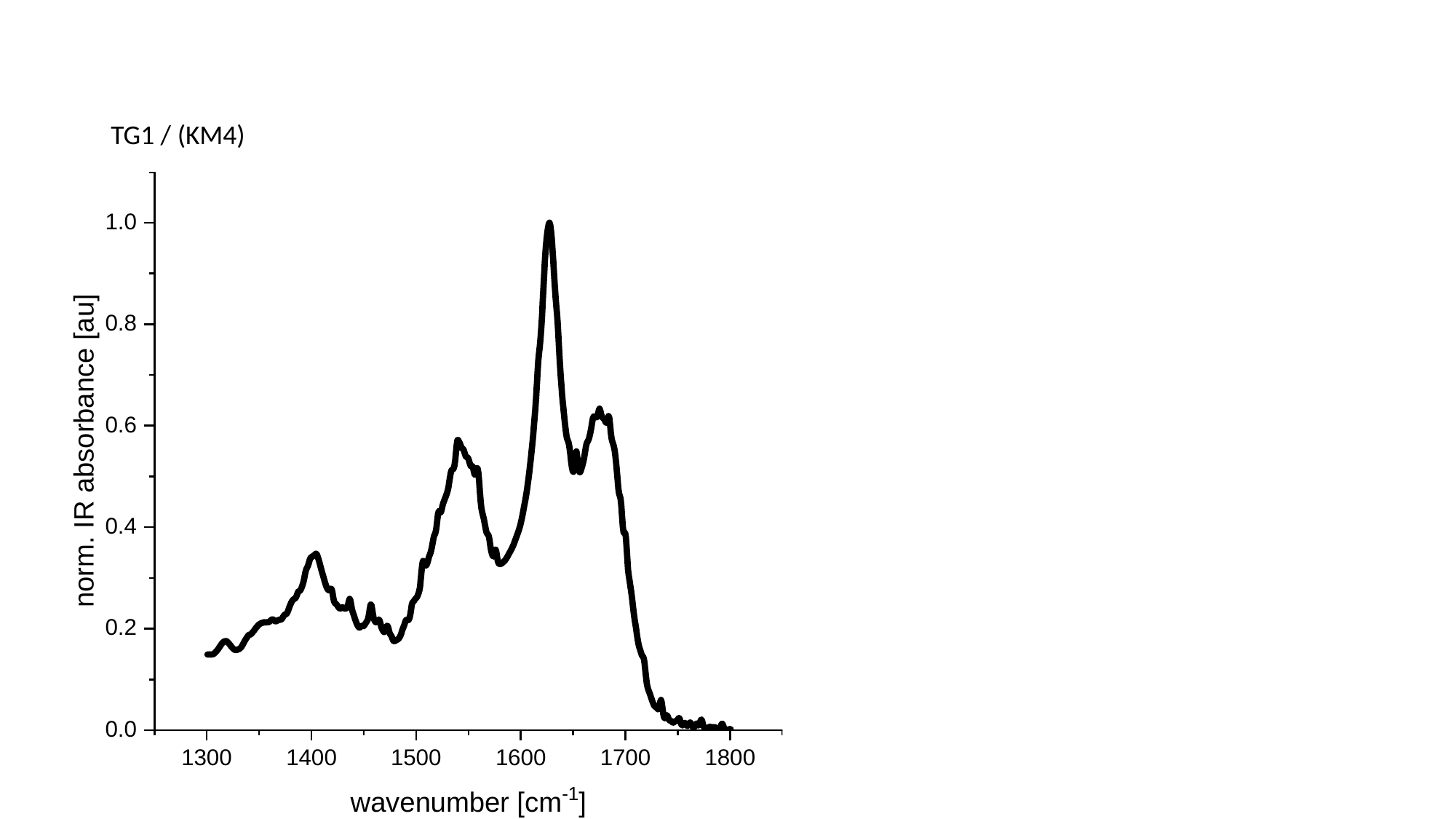

# TG1 / (KM4)

## Slide 114
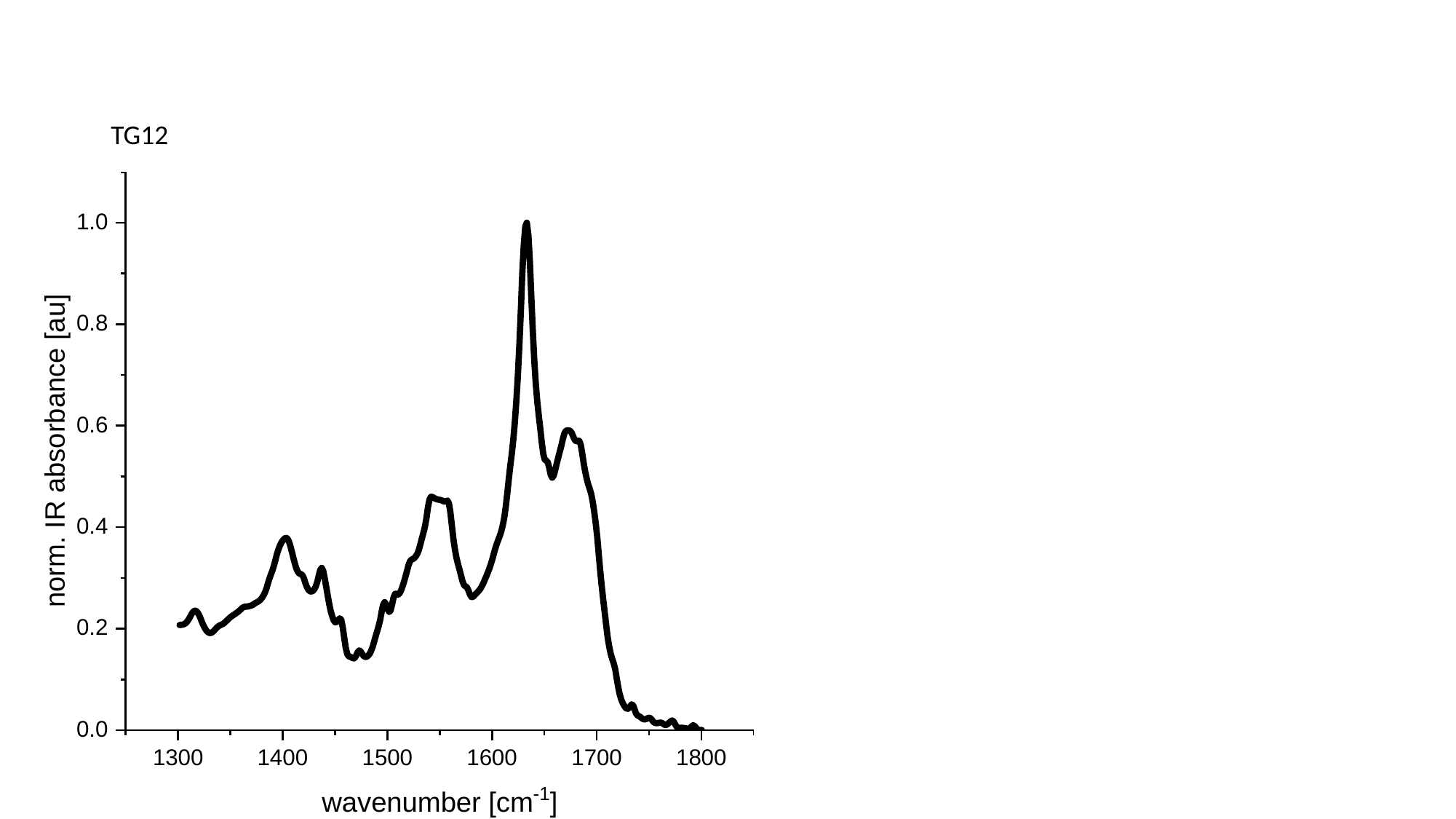

# TG12

## Slide 115
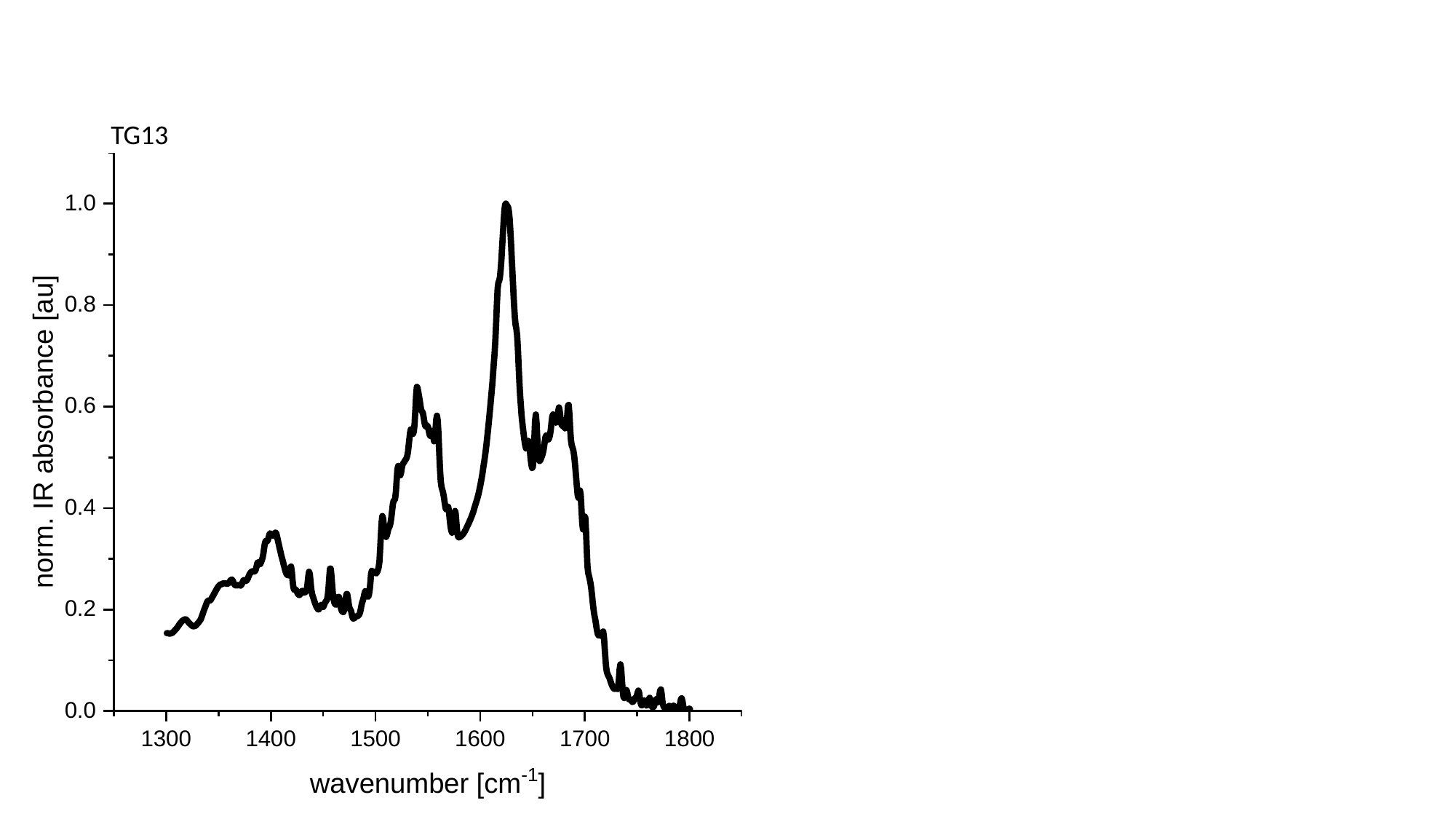

# TG13

## Slide 116
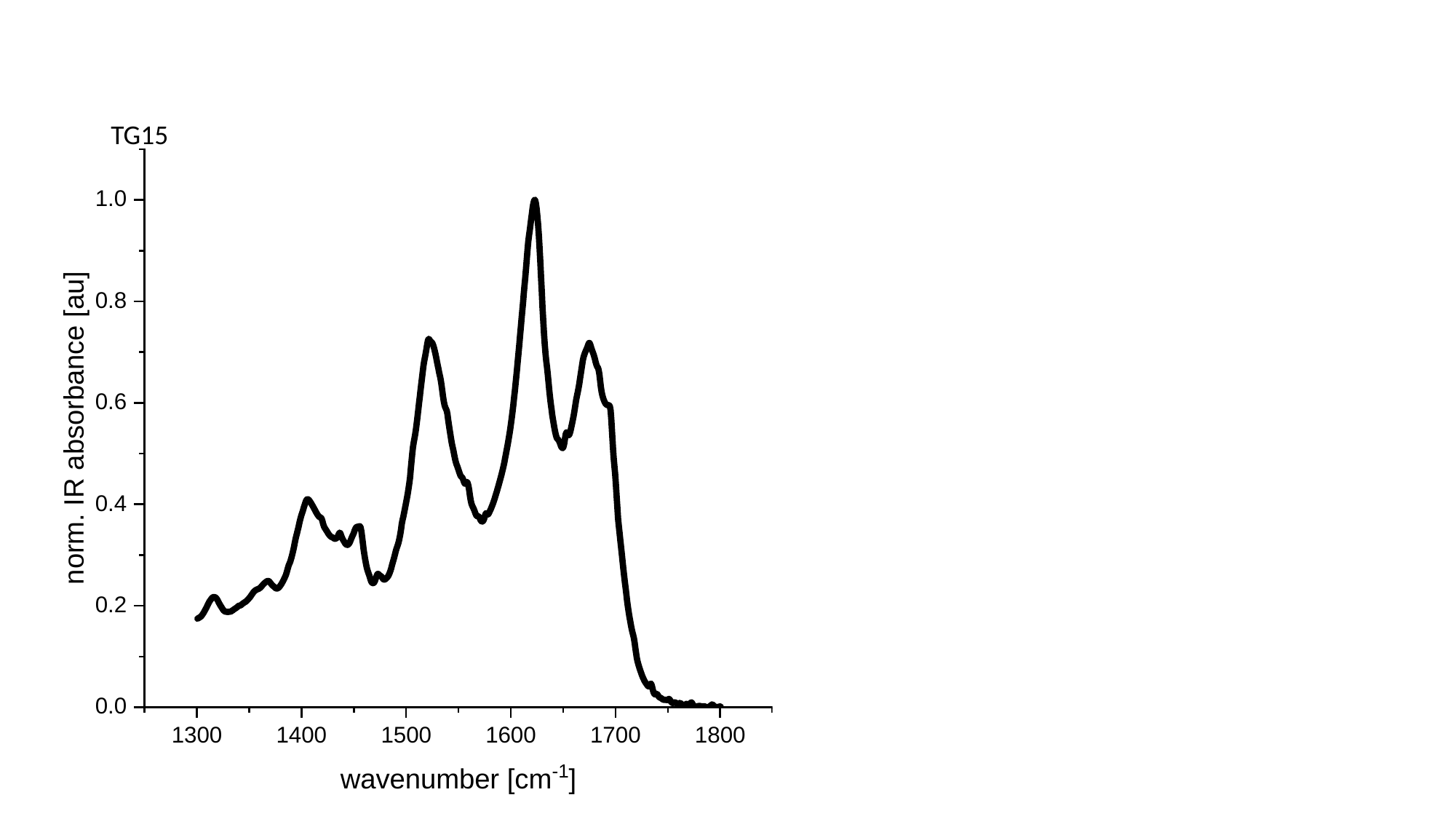

# TG15

## Slide 117
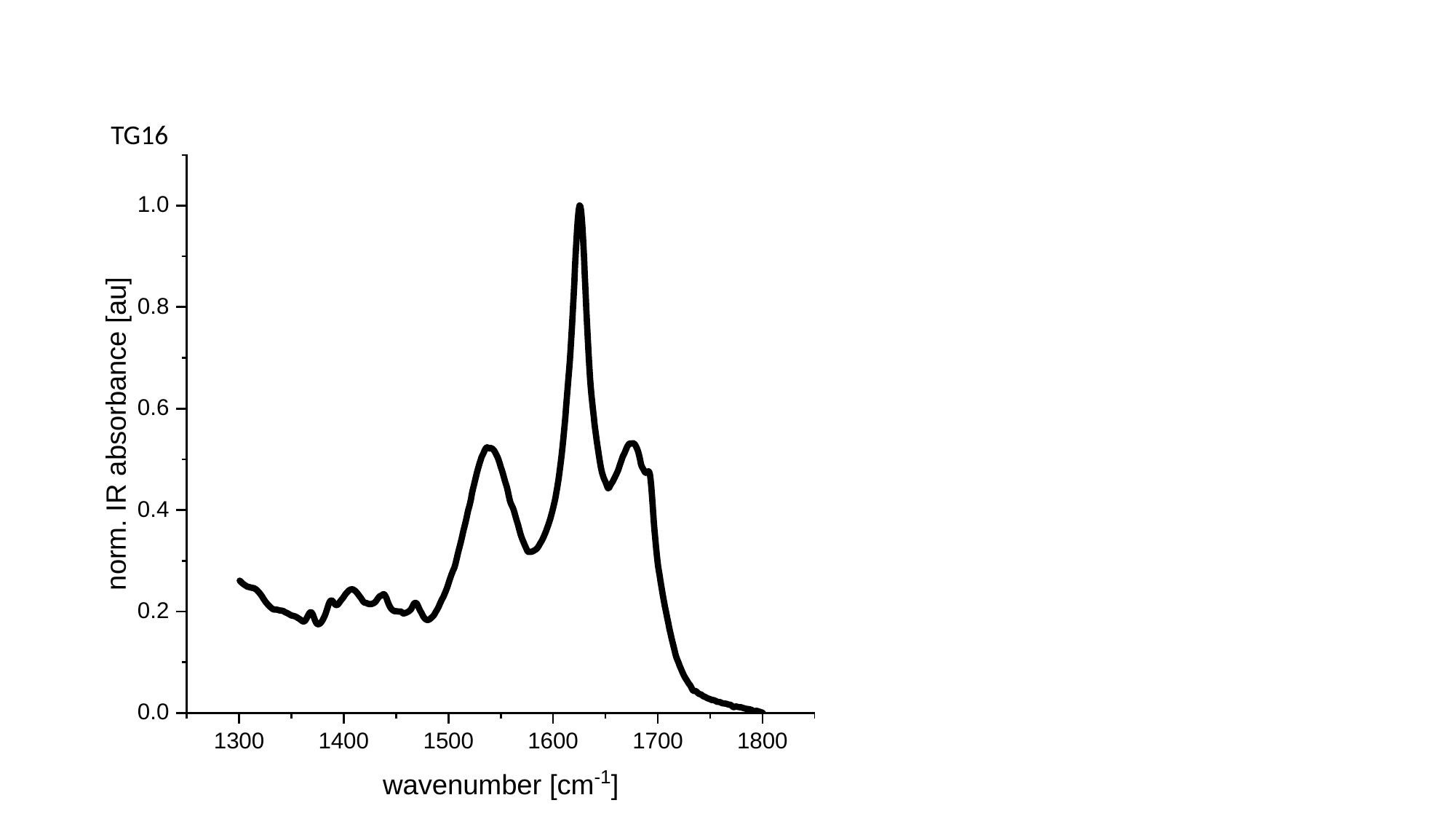

# TG16

## Slide 118
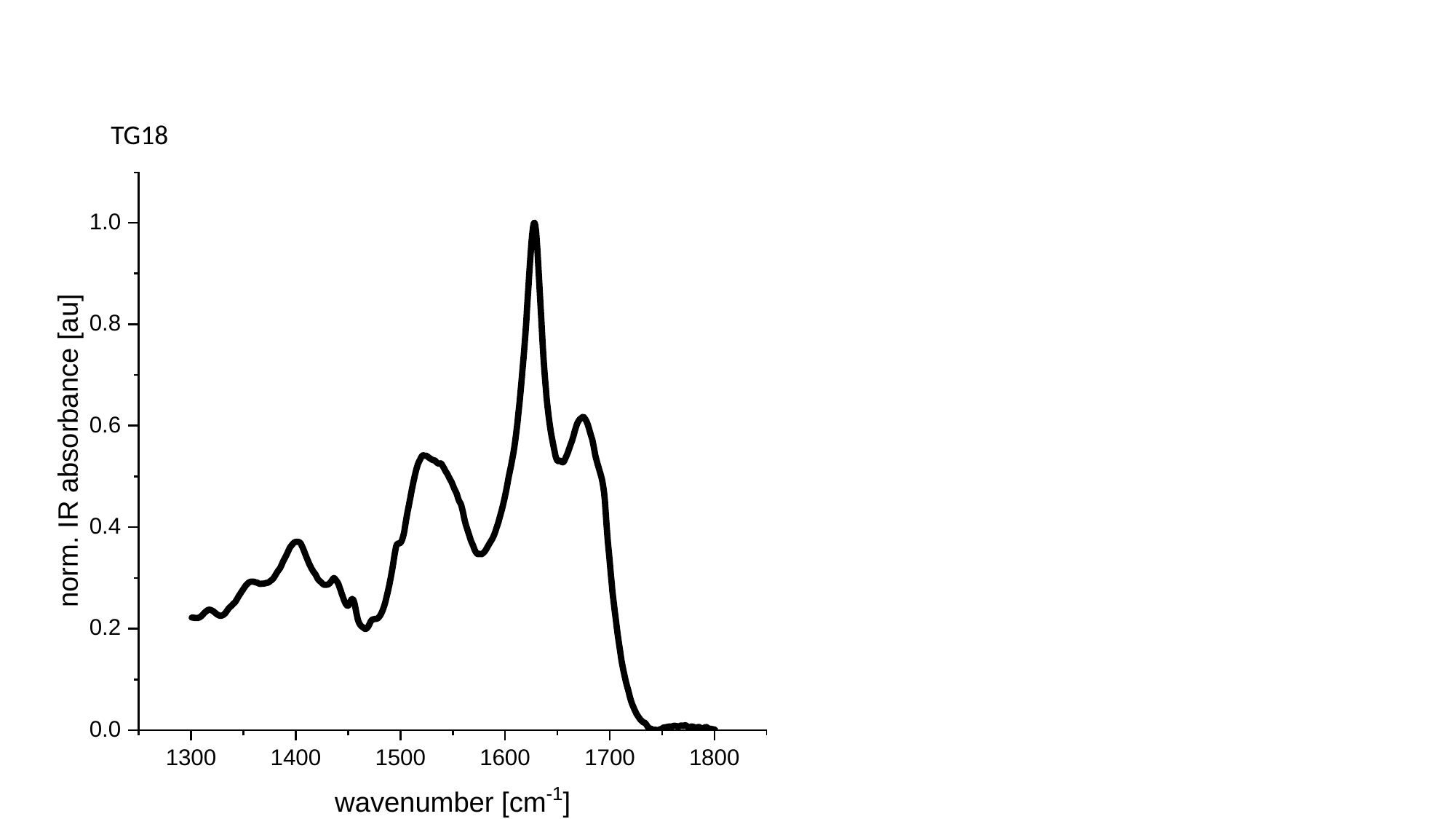

# TG18

## Slide 119
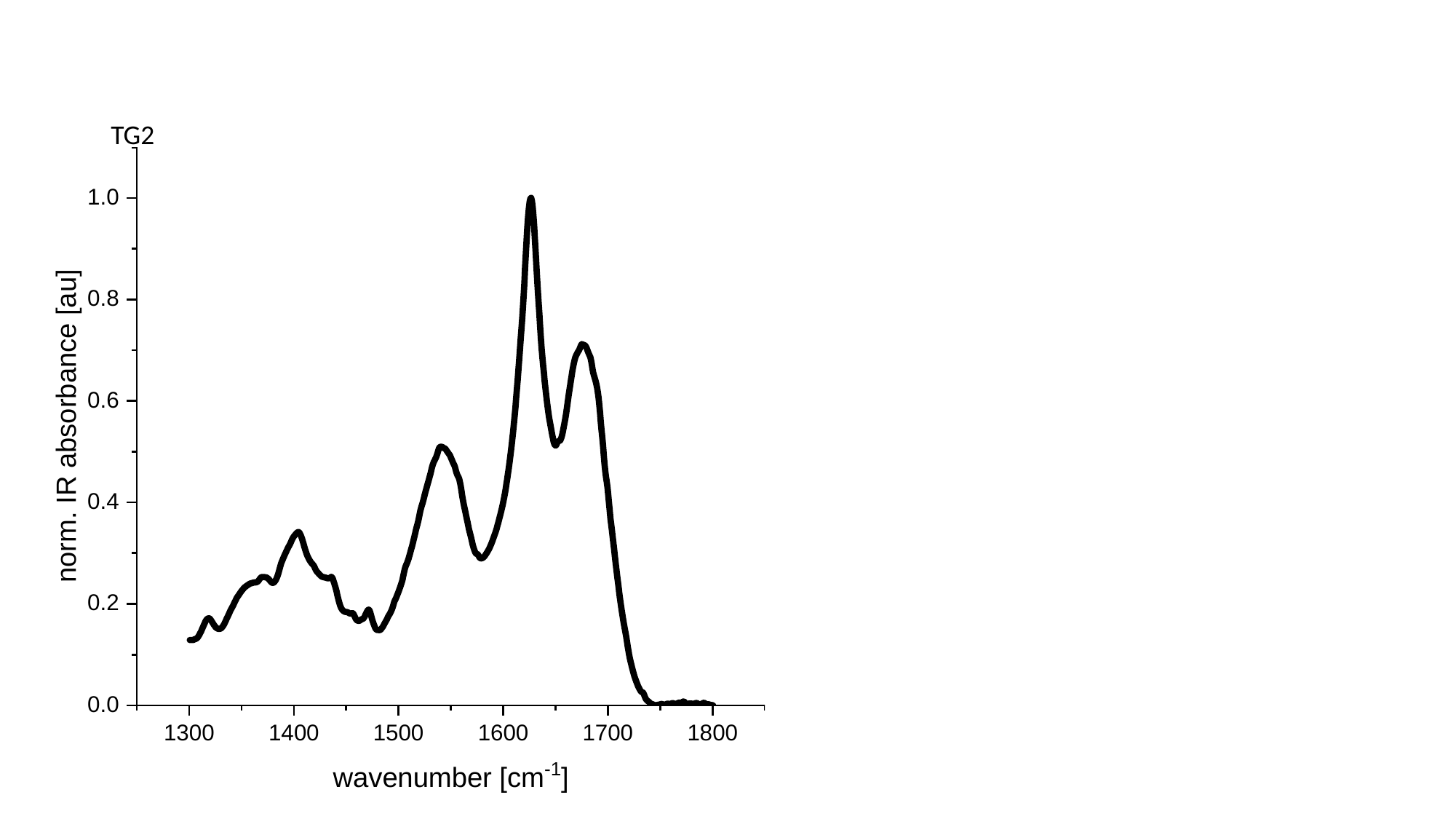

# TG2

## Slide 120
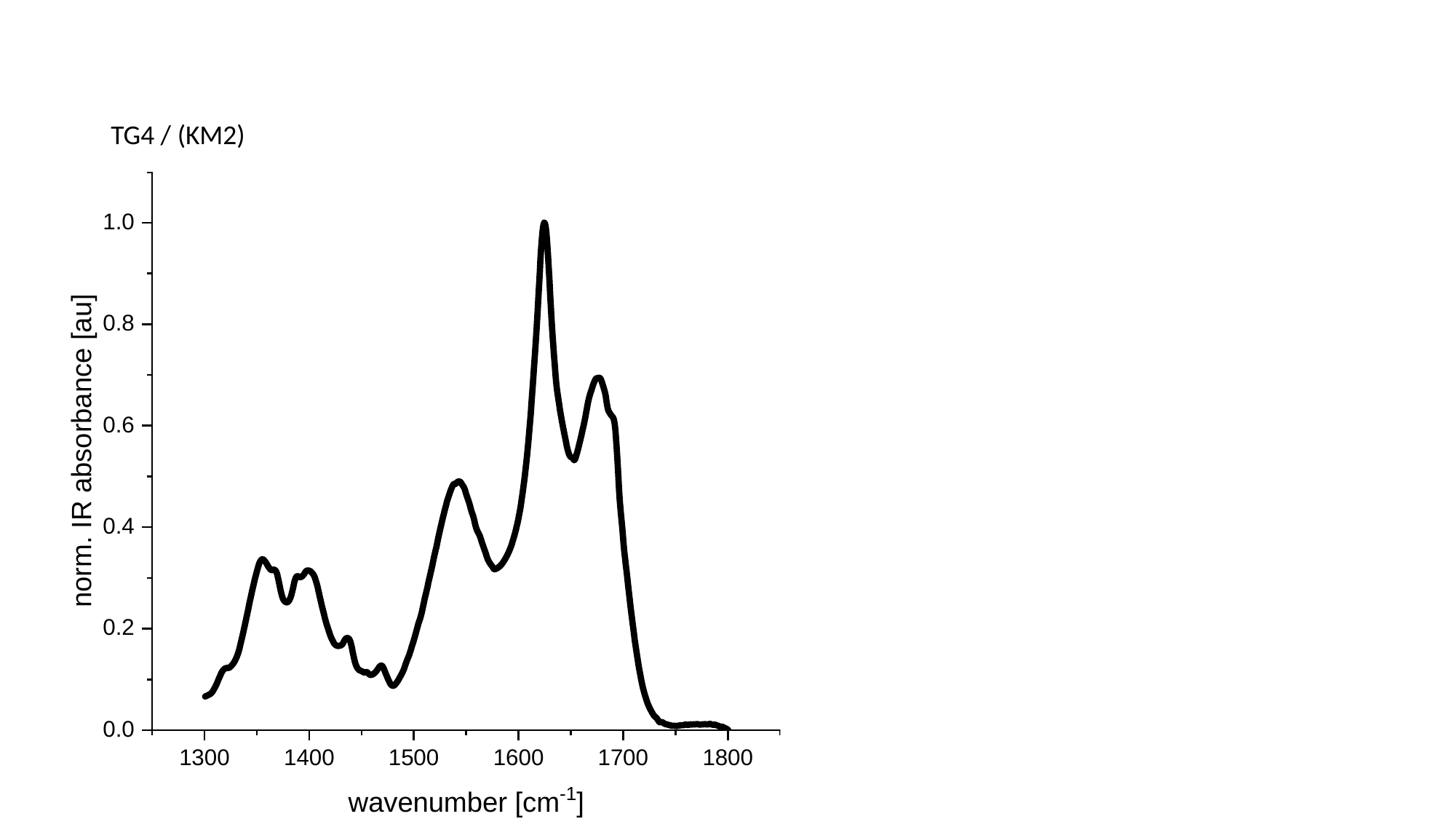

# TG4 / (KM2)

## Slide 121
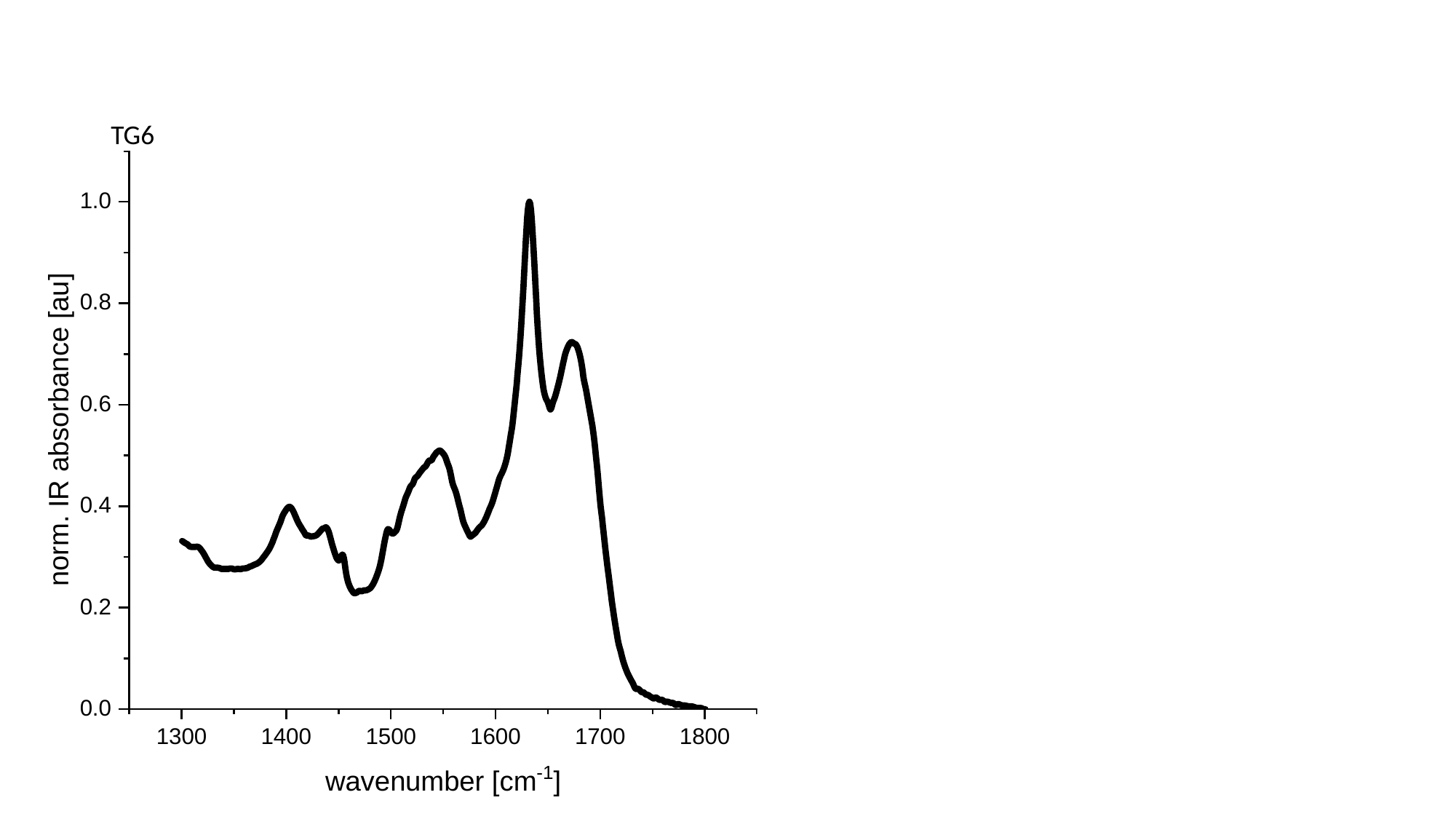

# TG6

## Slide 122
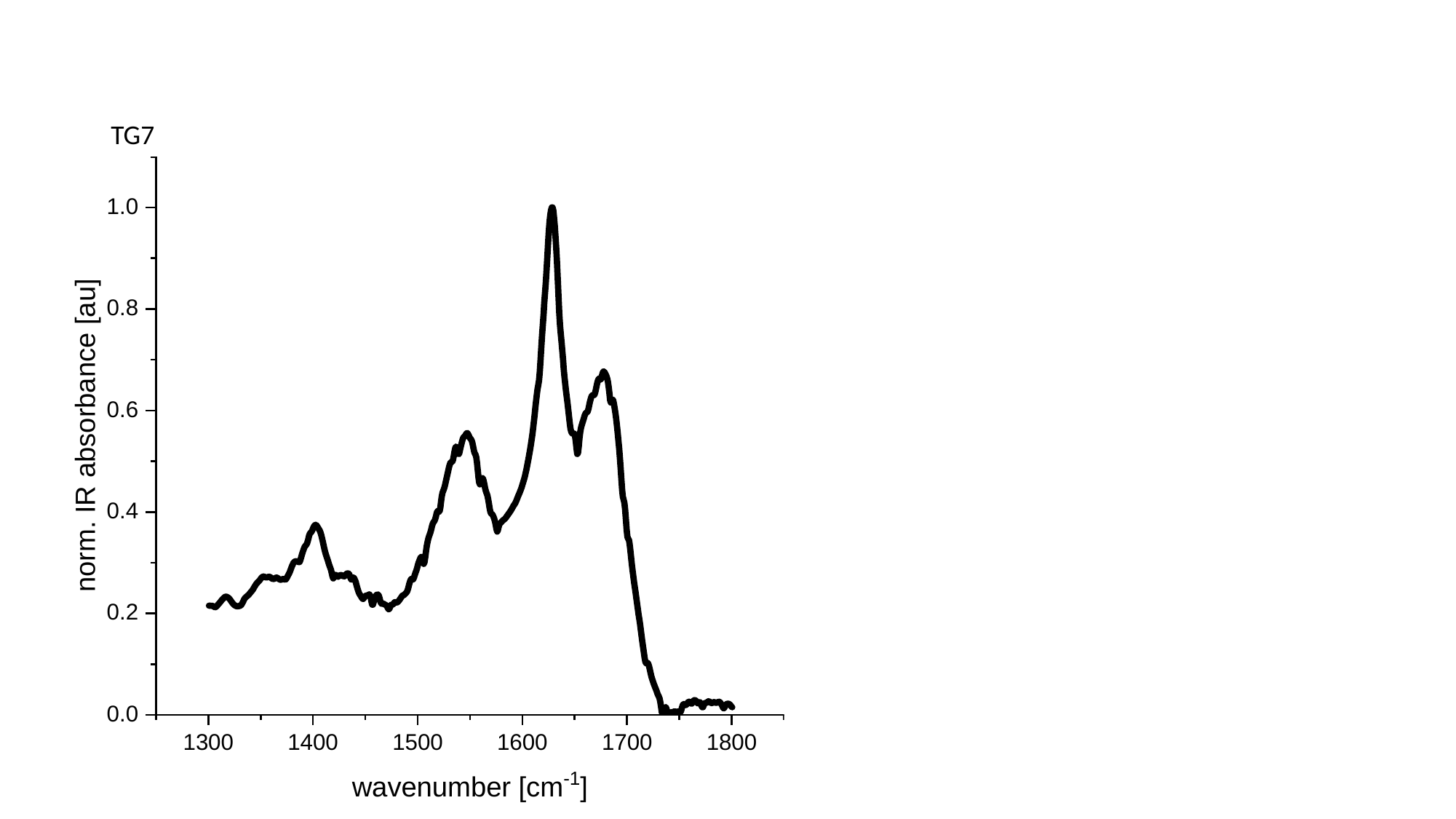

# TG7

## Slide 123
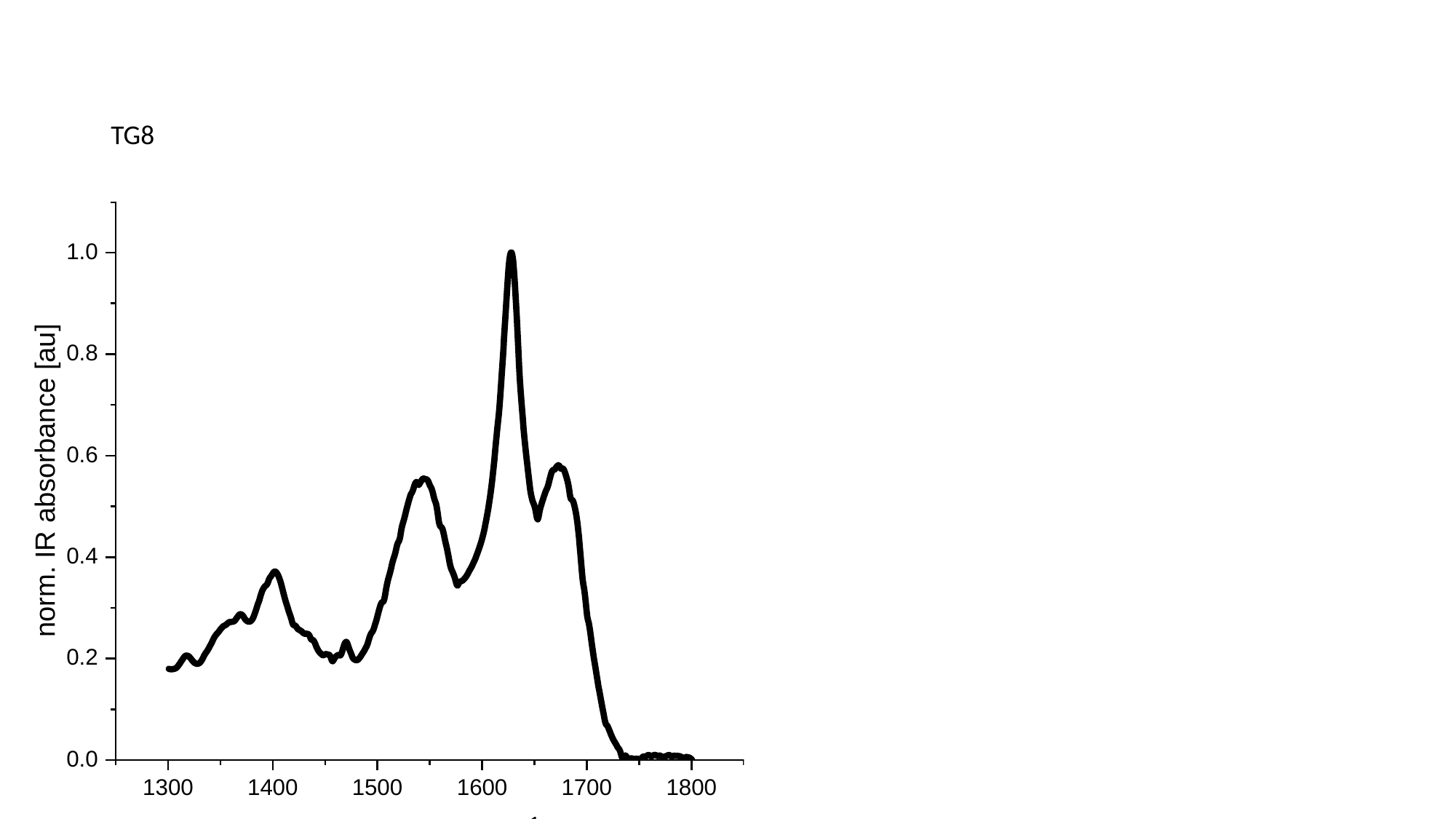

# TG8

## Slide 124
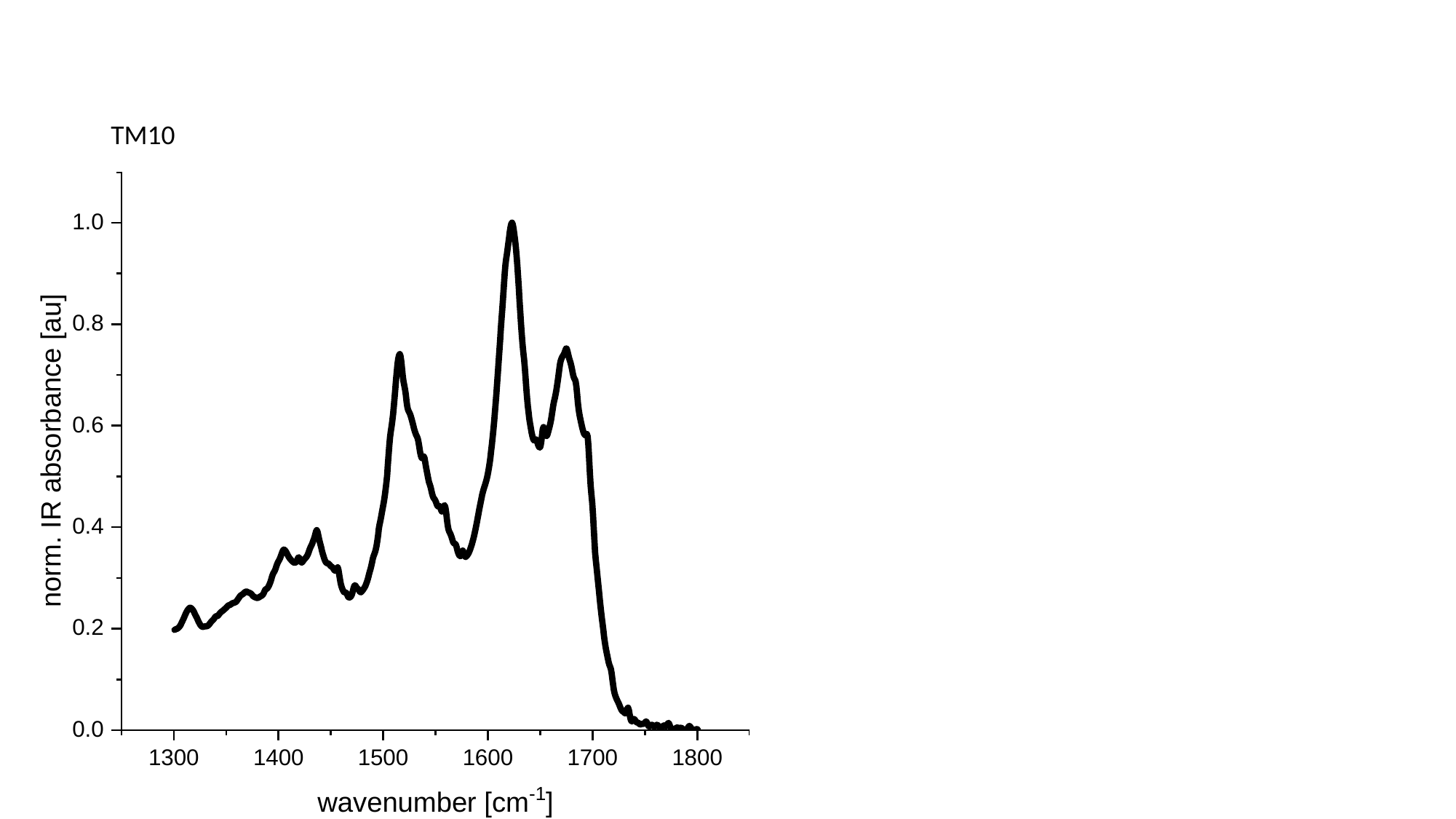

# TM10

## Slide 125
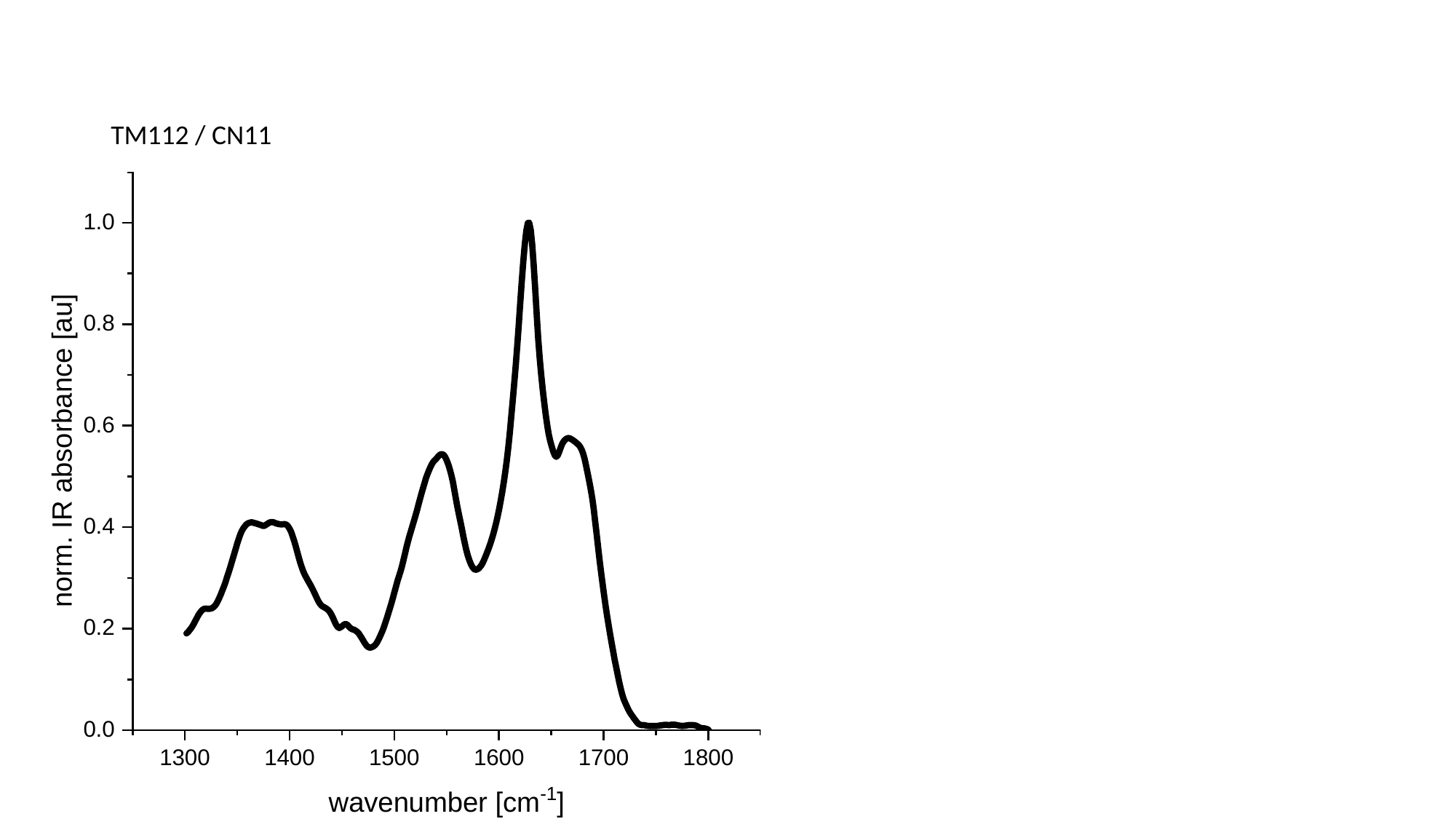

# TM112 / CN11

## Slide 126
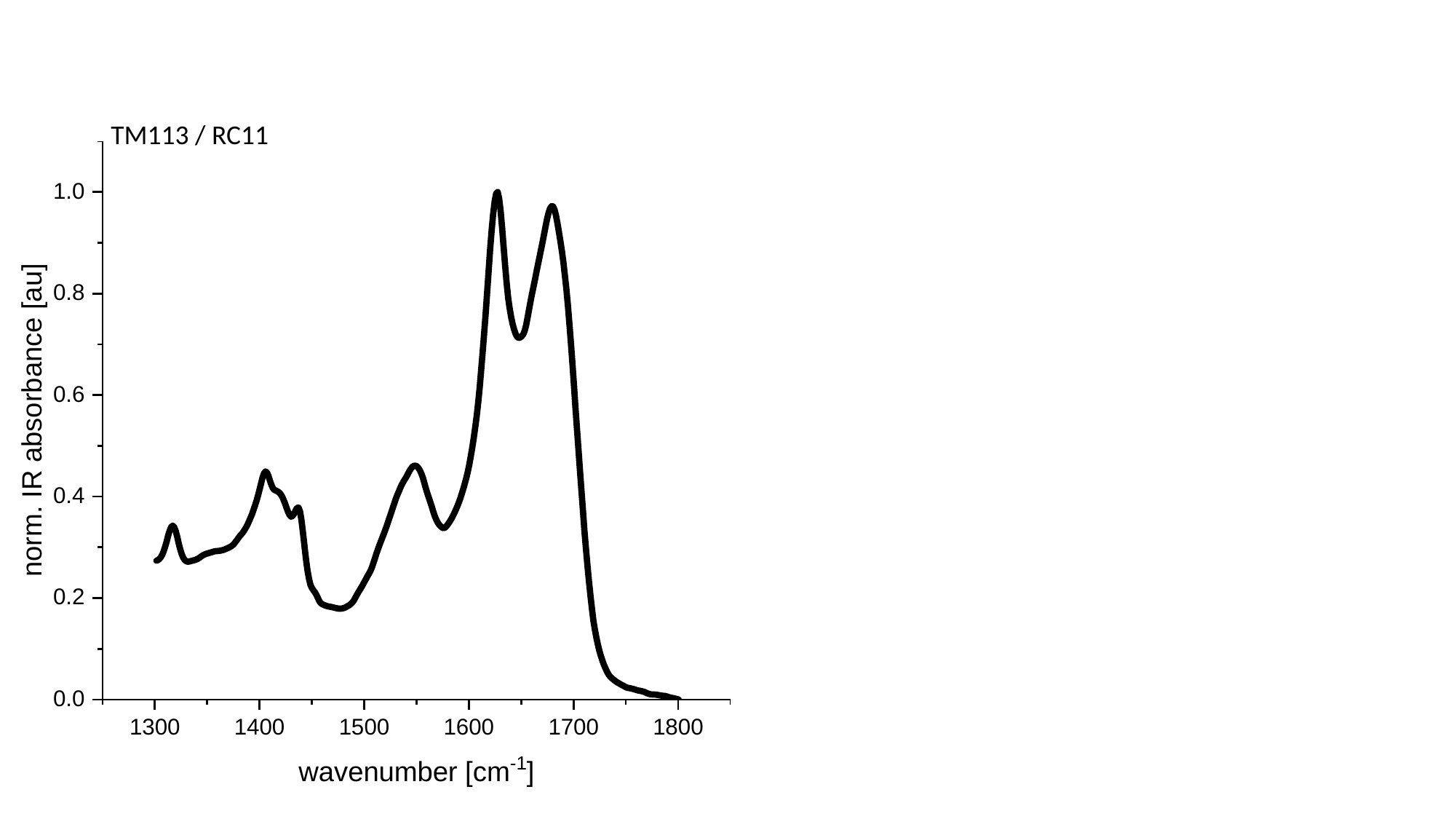

# TM113 / RC11

## Slide 127
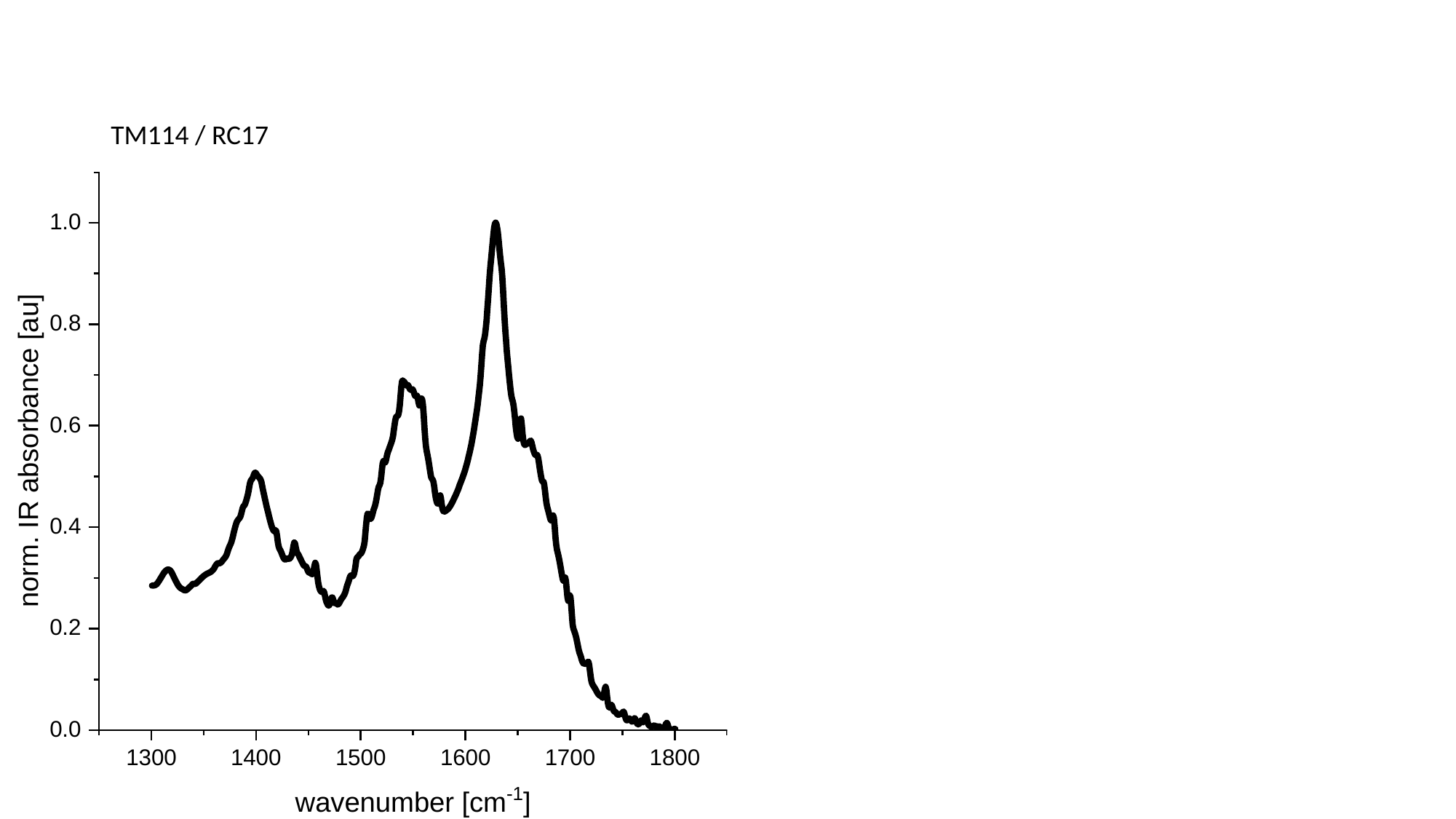

# TM114 / RC17

## Slide 128
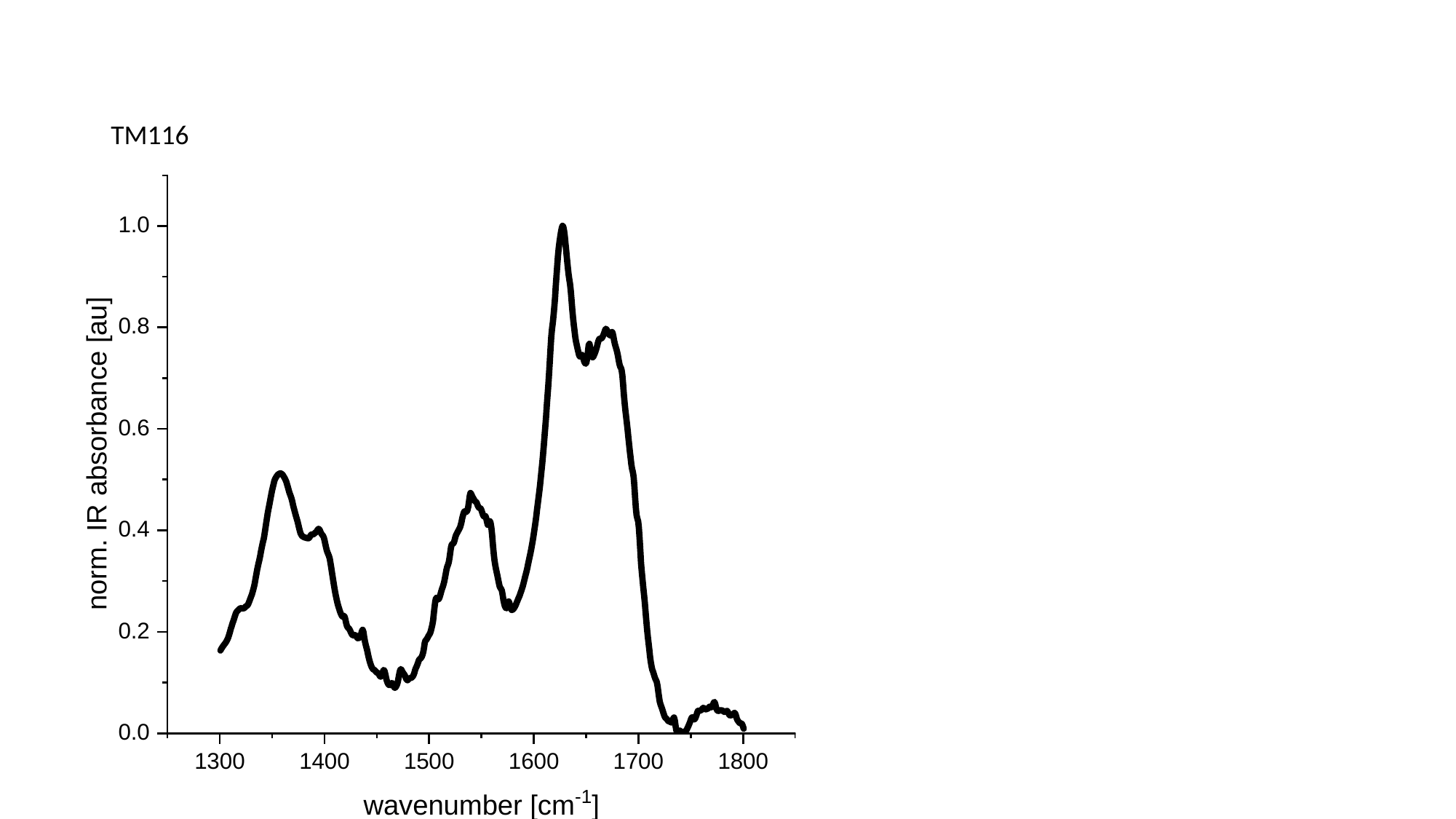

# TM116

## Slide 129
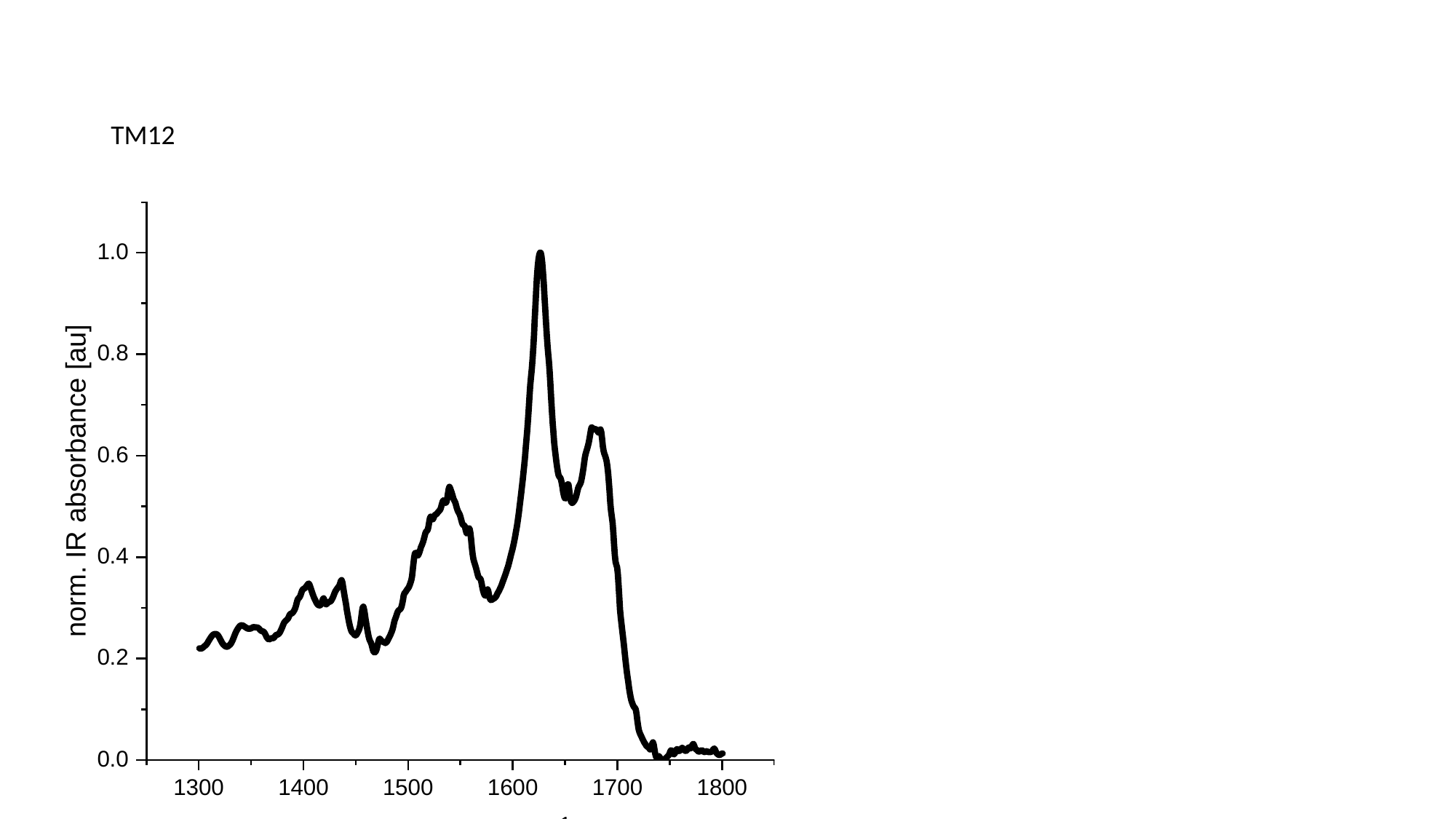

# TM12

## Slide 130
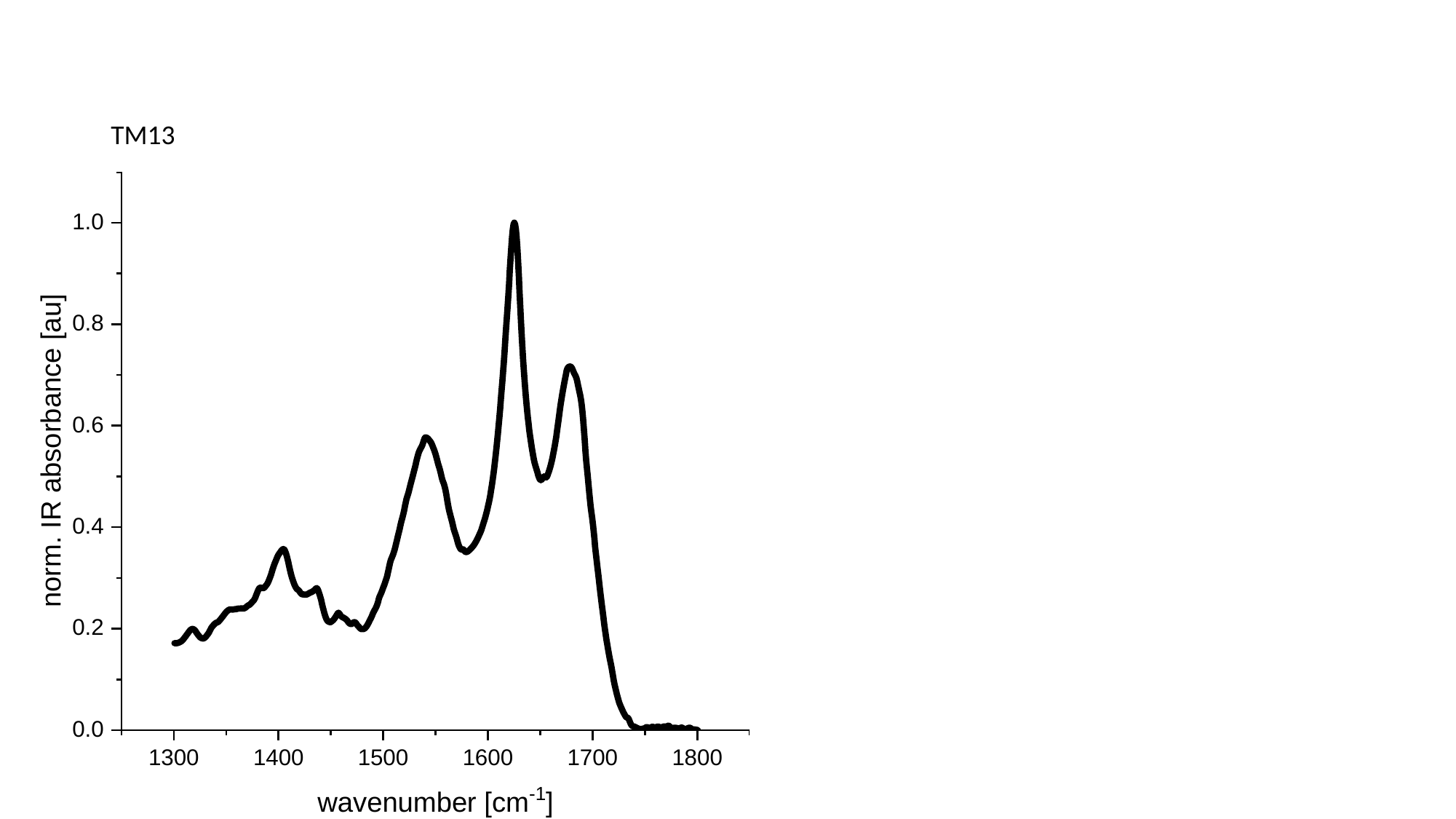

# TM13

## Slide 131
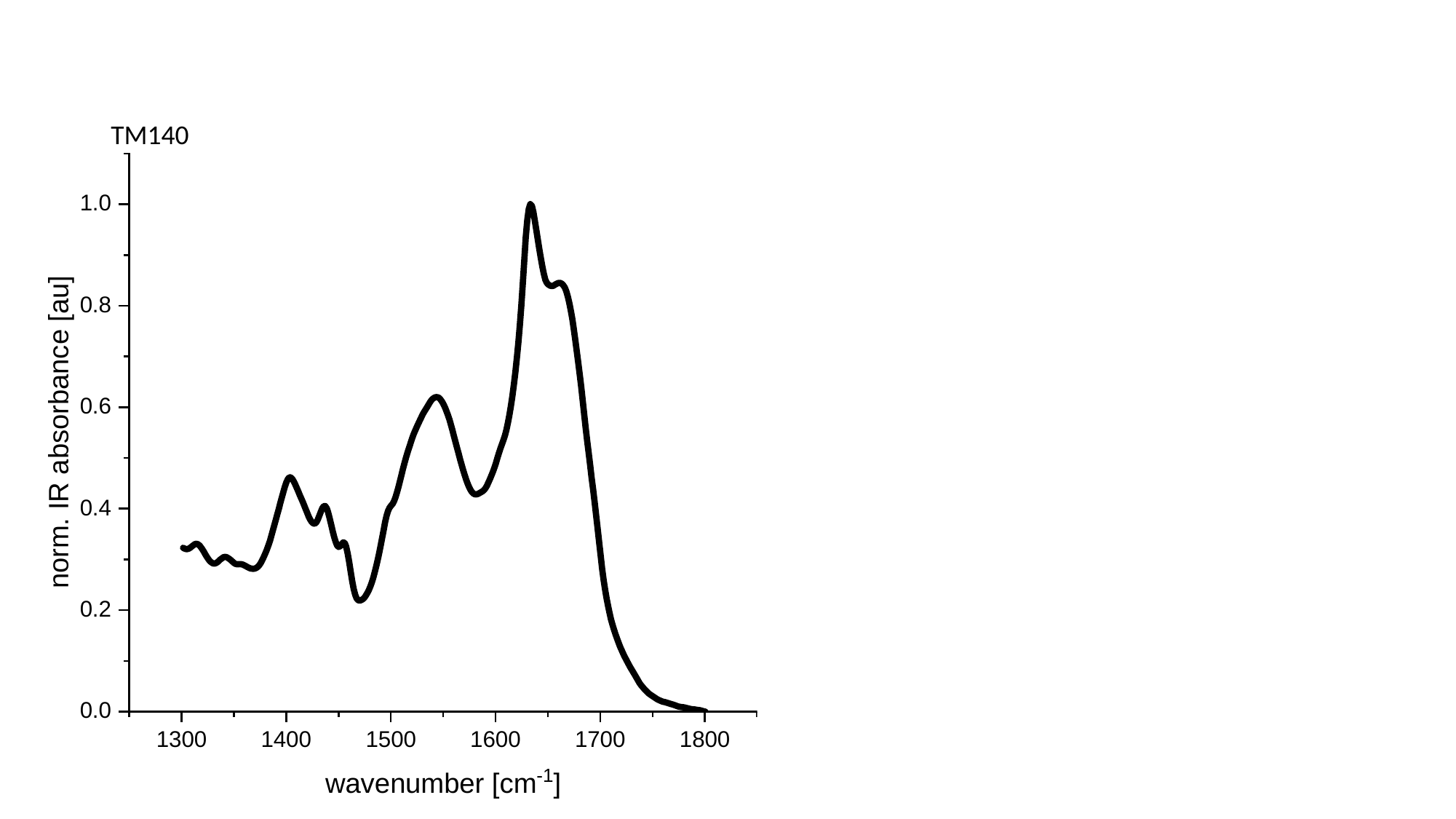

# TM140

## Slide 132
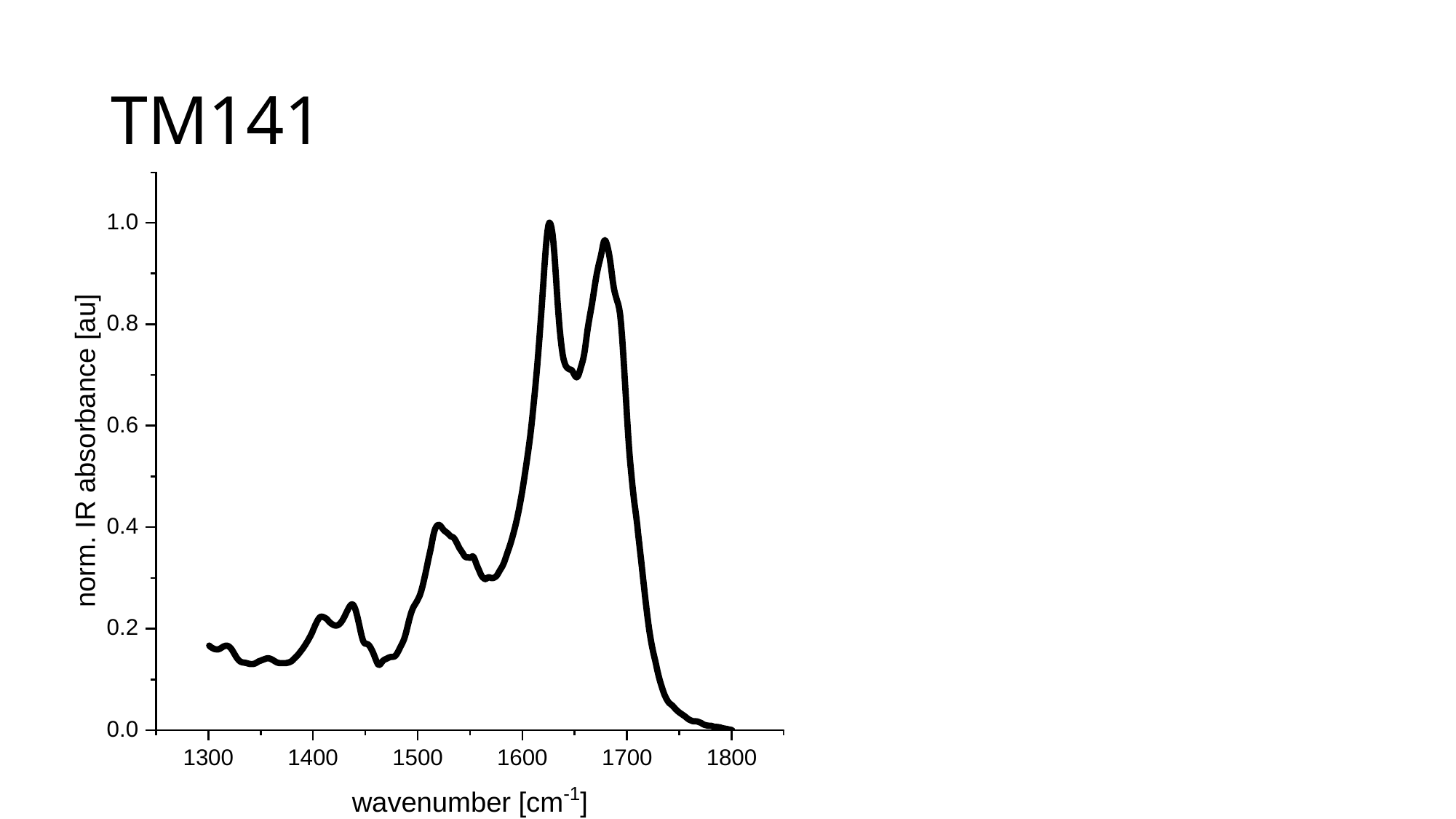

# TM141

## Slide 133
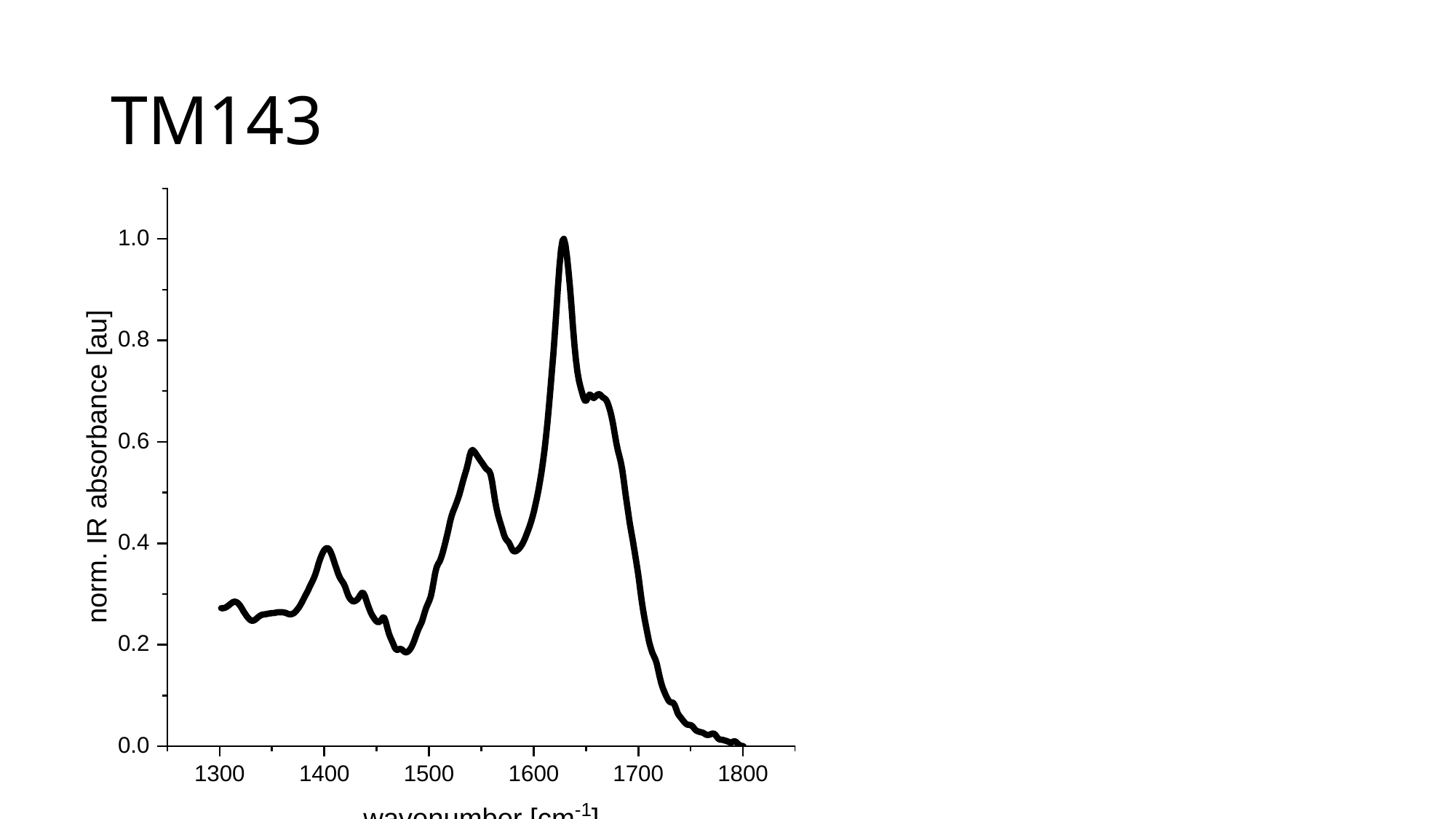

# TM143

## Slide 134
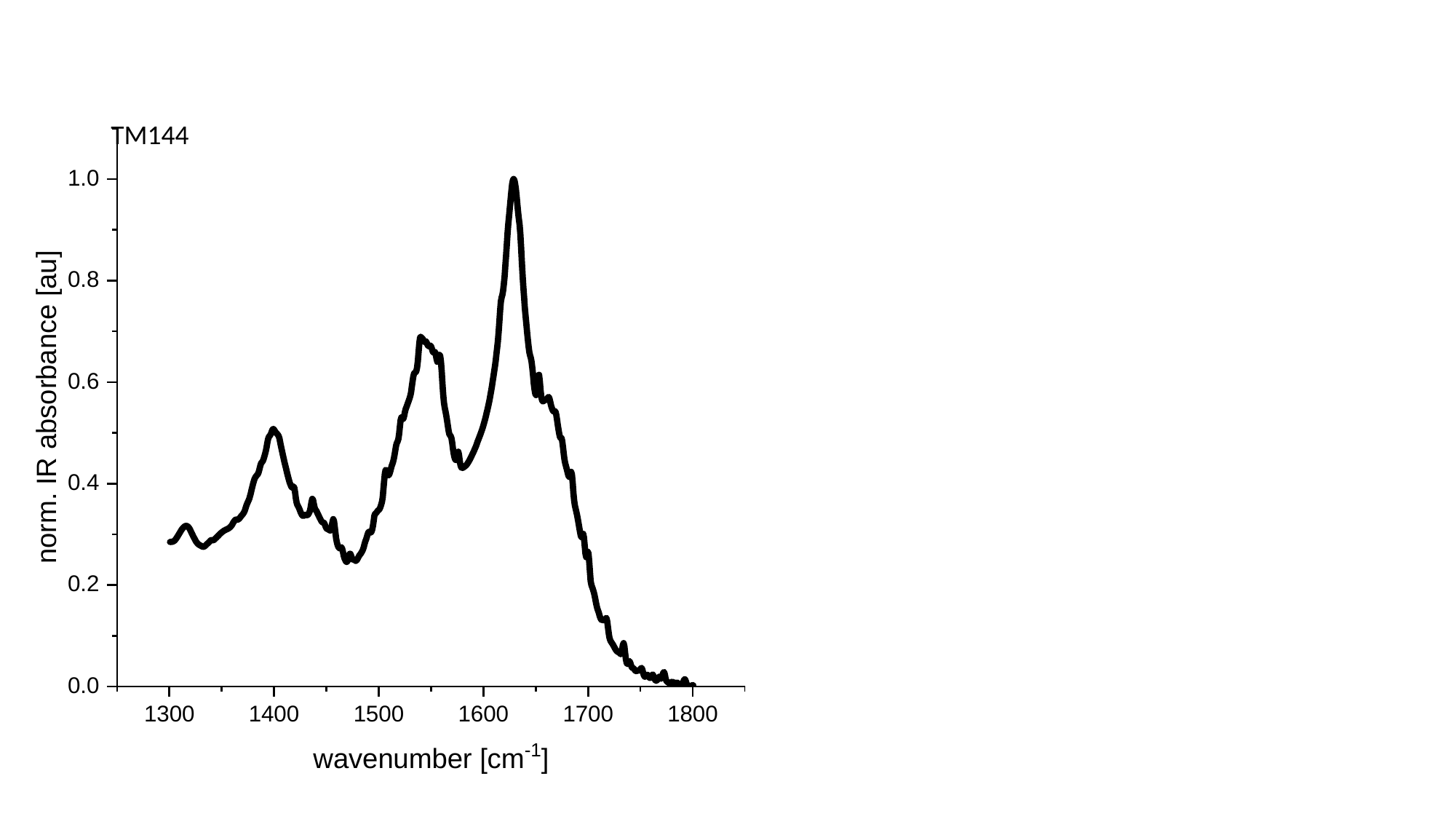

# TM144

## Slide 135
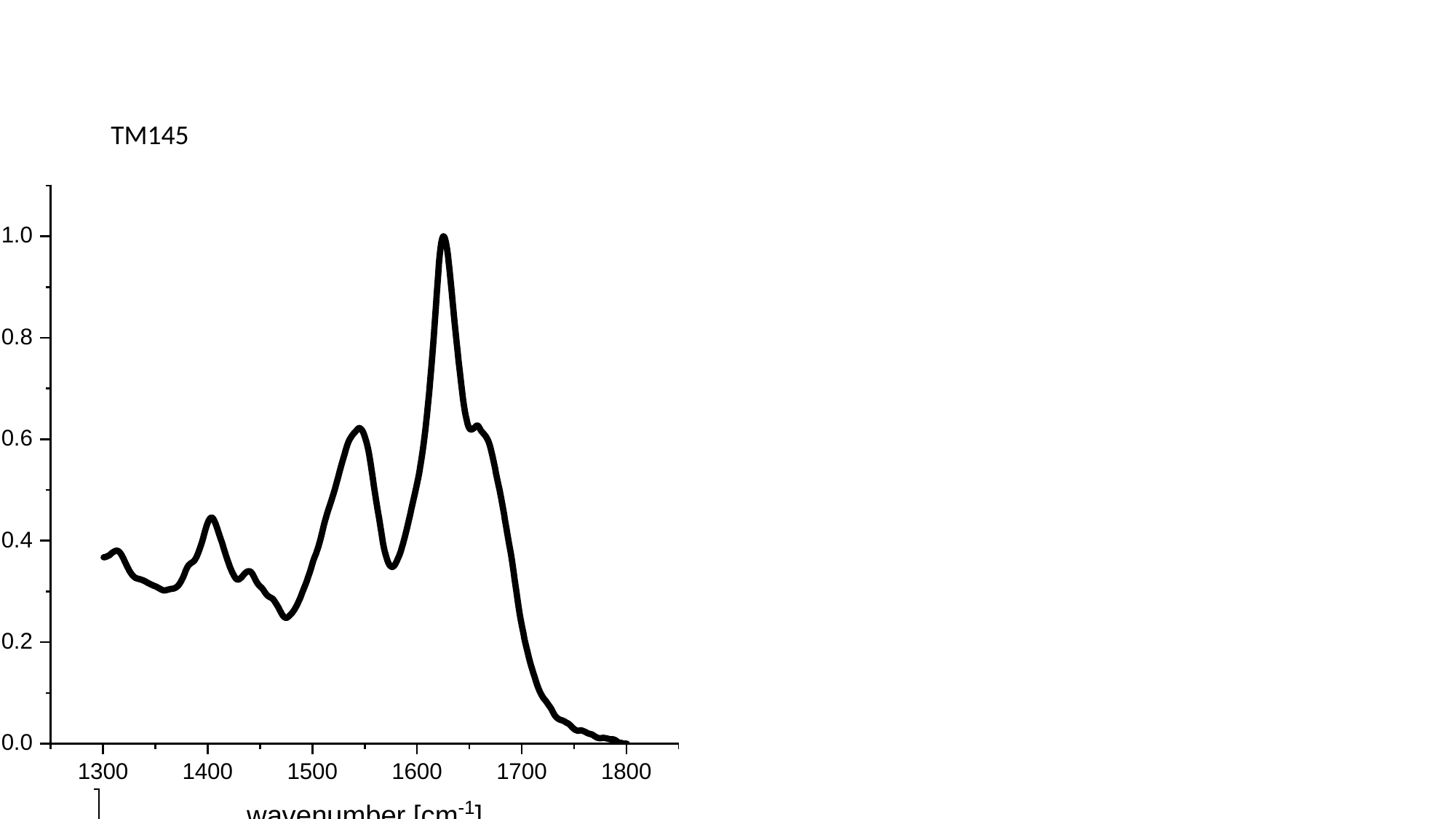

# TM145

## Slide 136
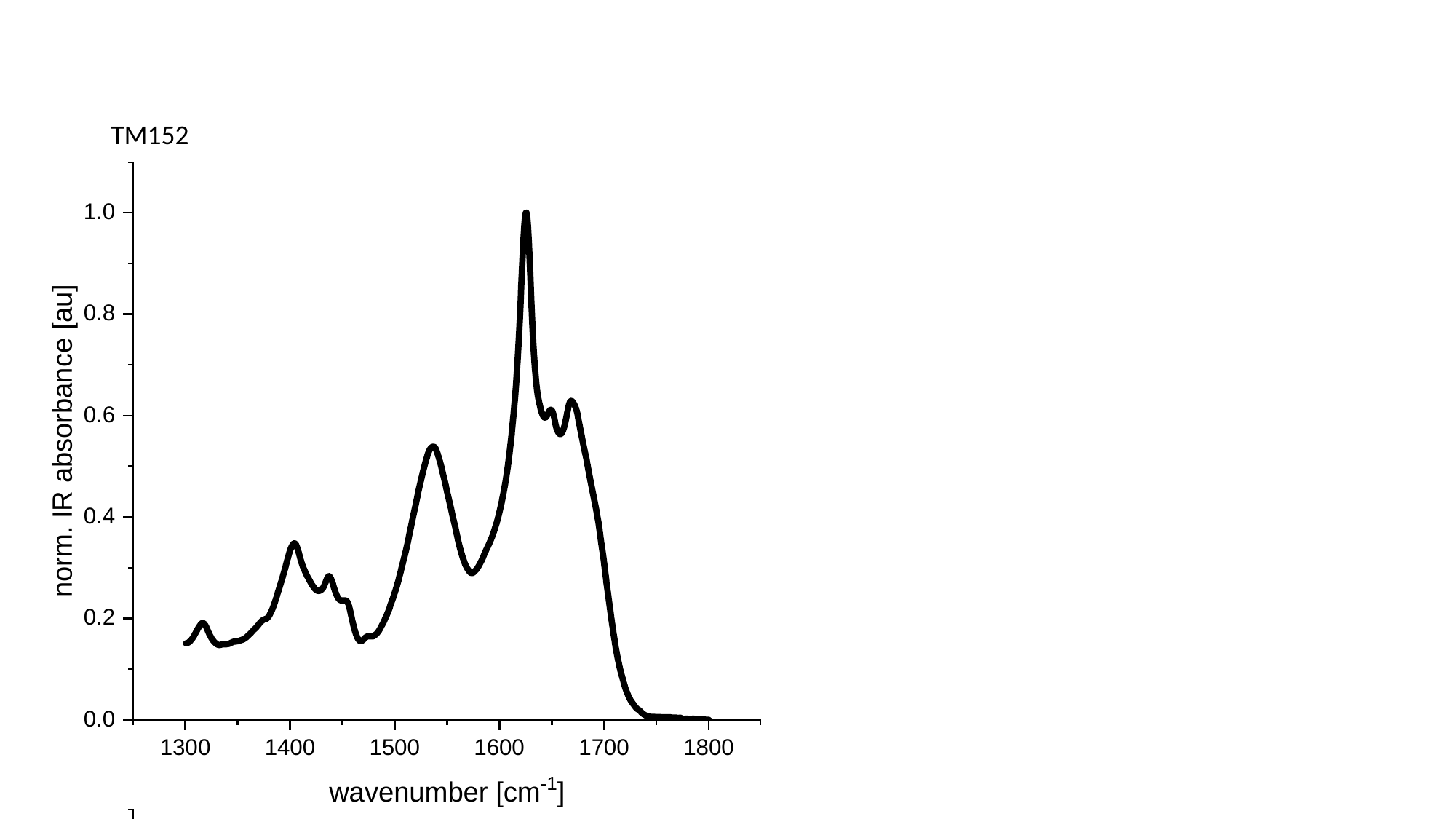

# TM152

## Slide 137
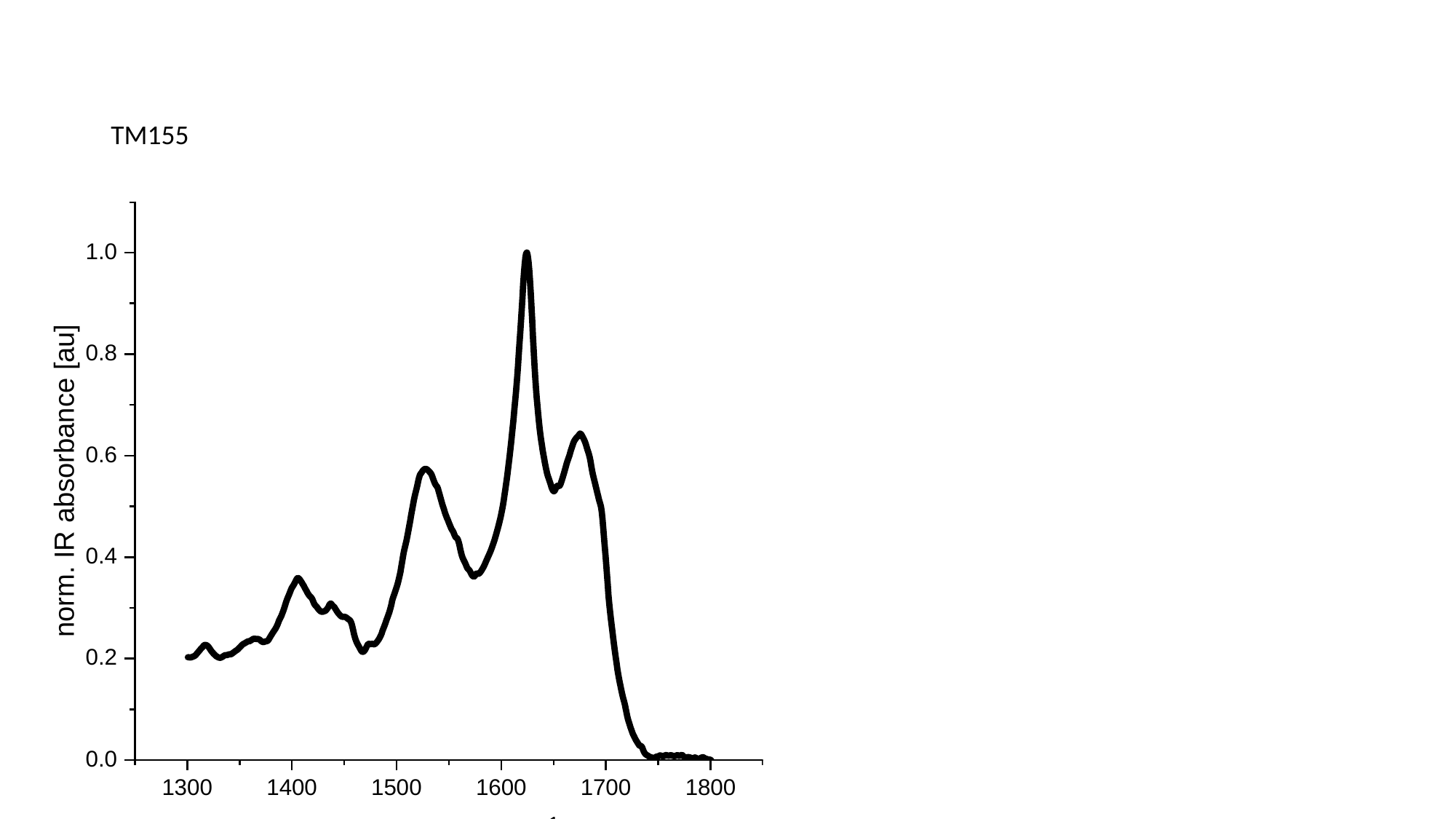

# TM155

## Slide 138
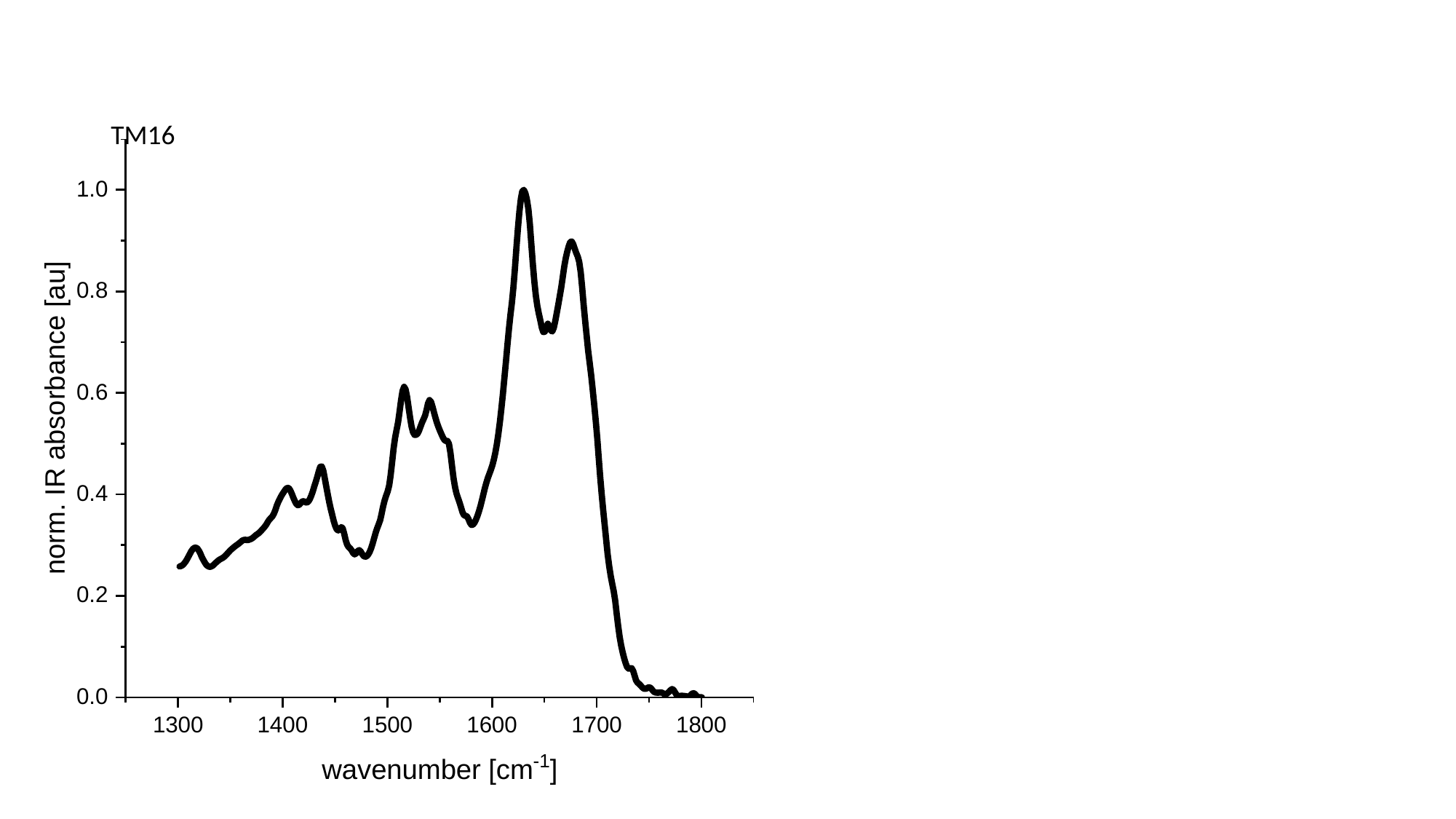

# TM16

## Slide 139
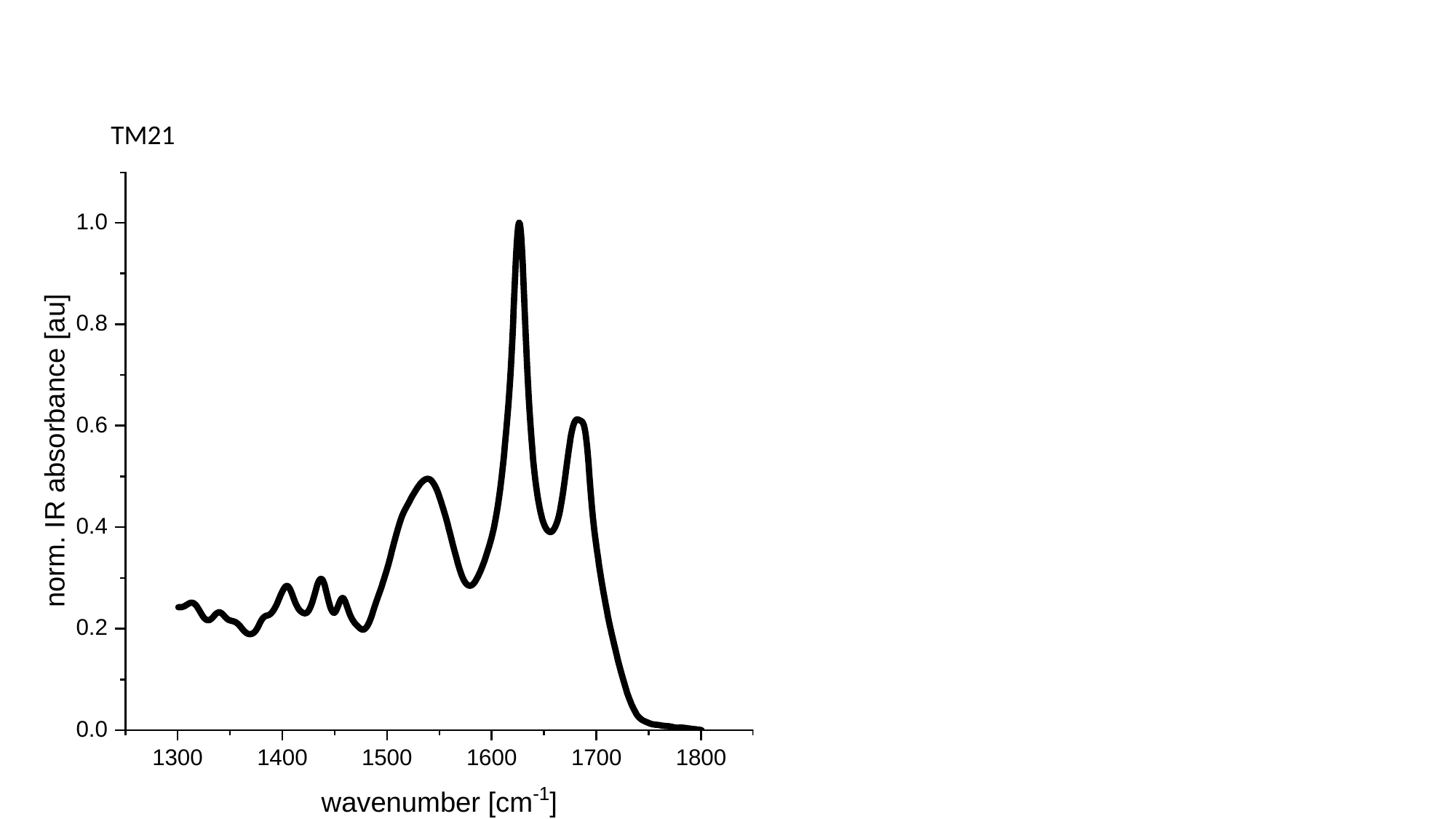

# TM21

## Slide 140
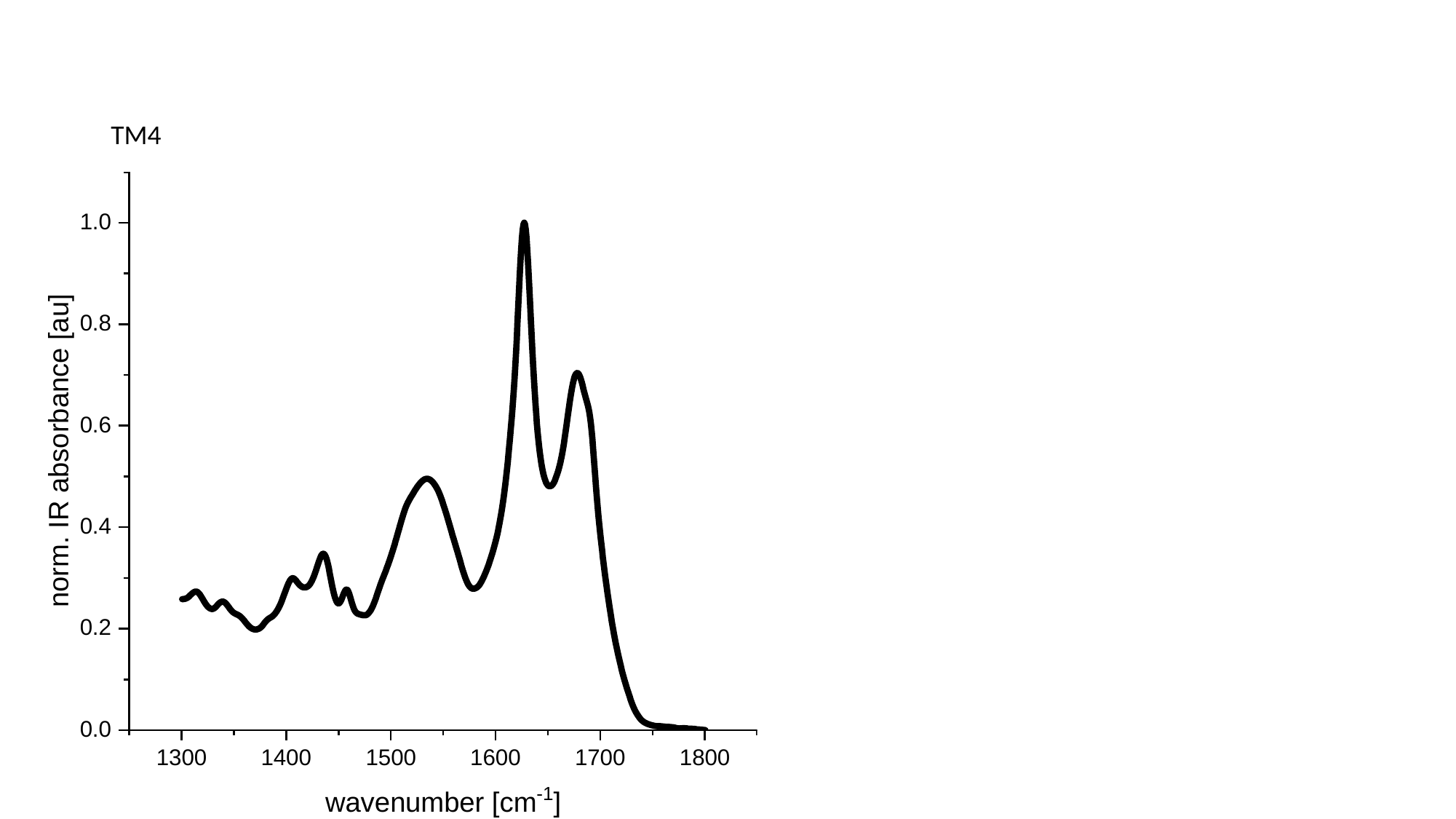

# TM4

## Slide 141
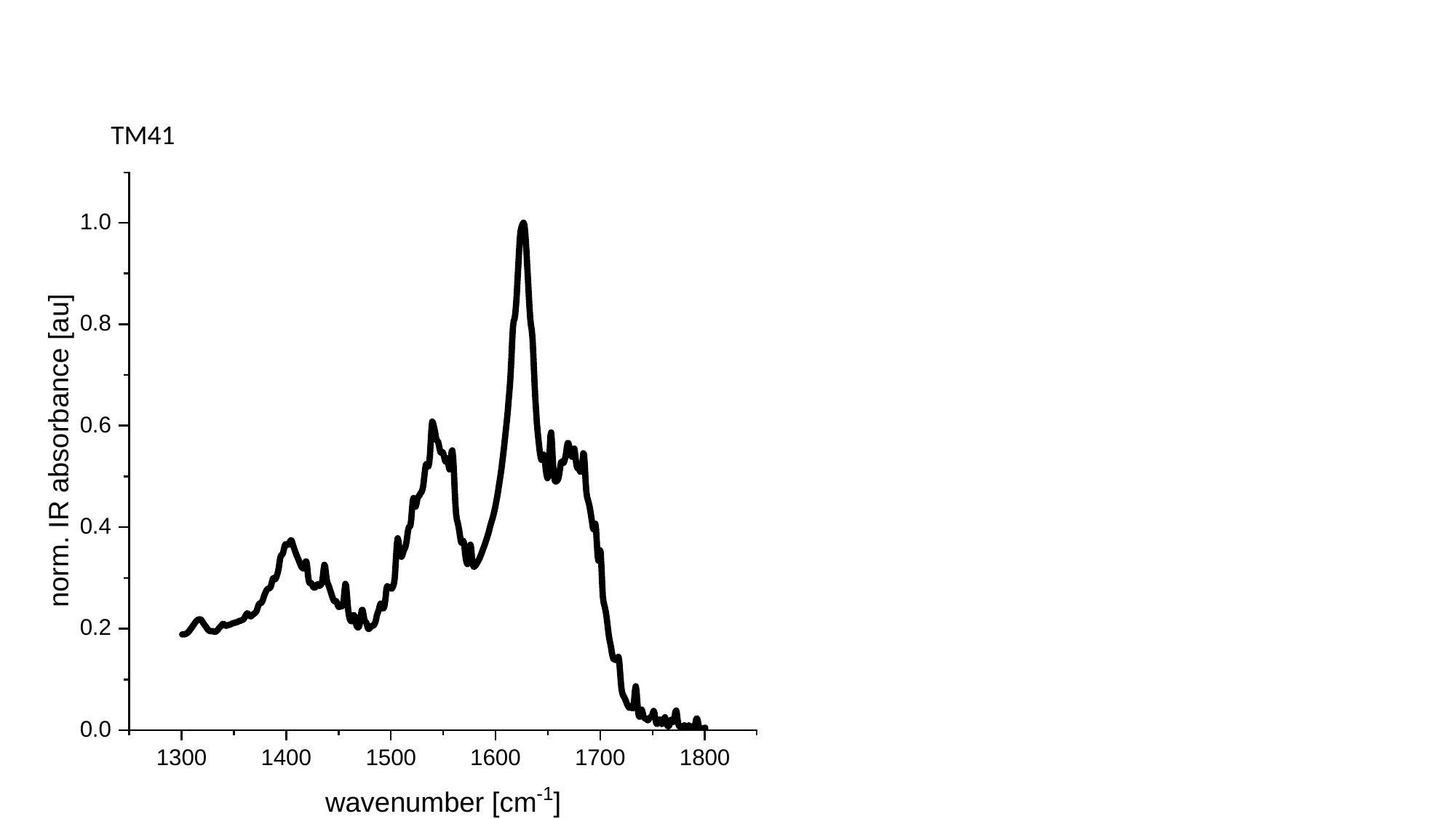

# TM41

## Slide 142
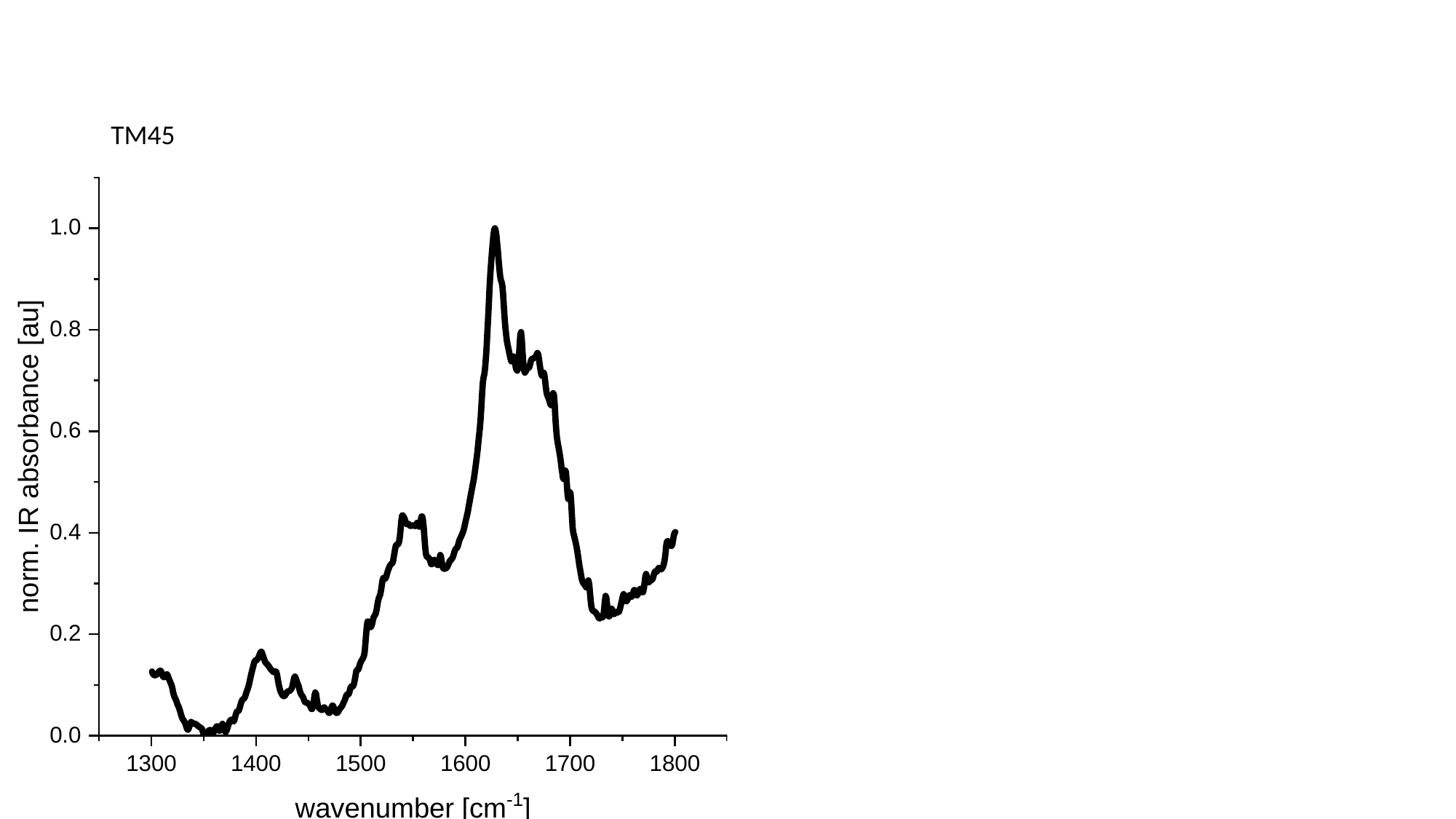

# TM45

## Slide 143
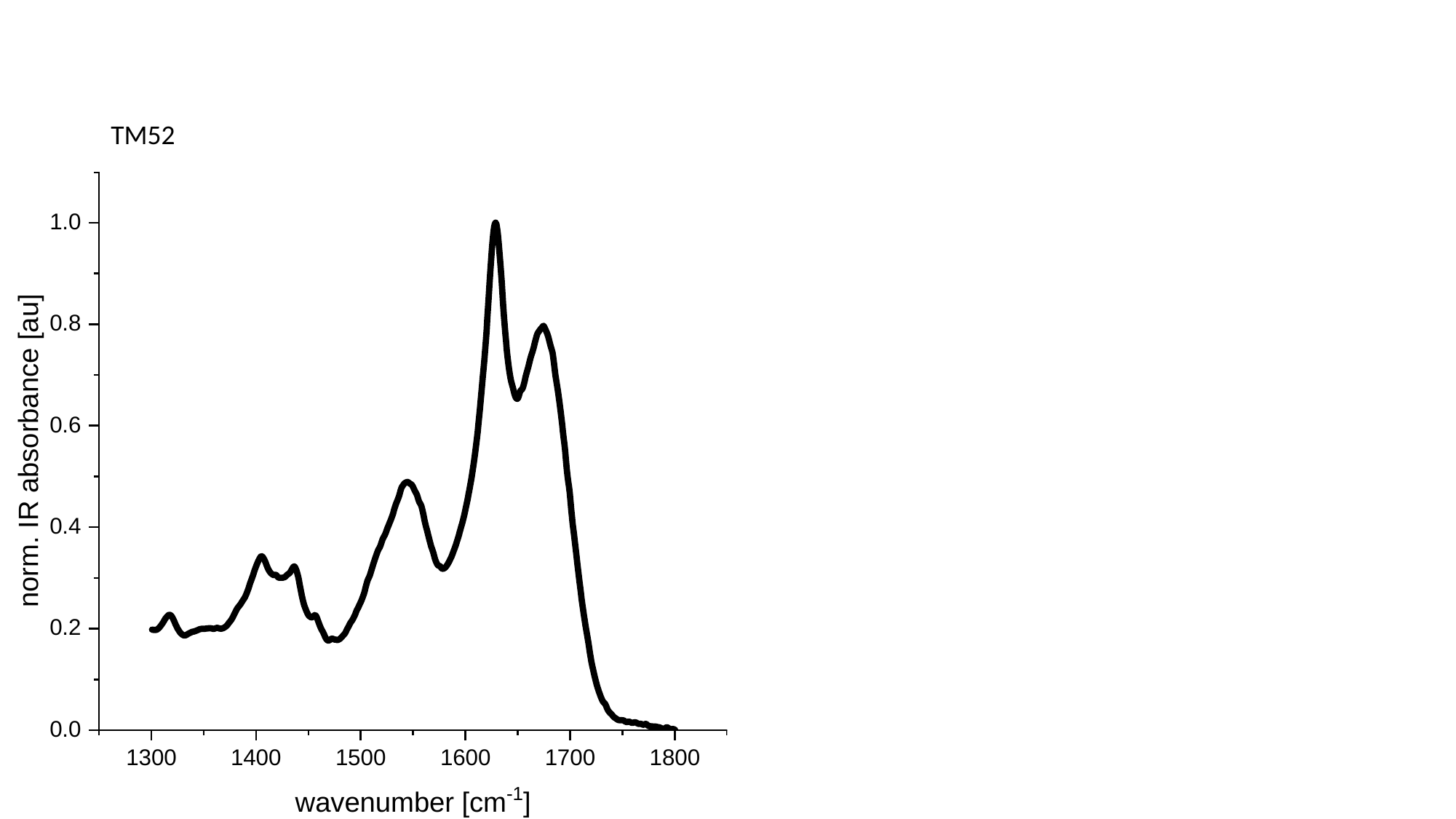

# TM52

## Slide 144
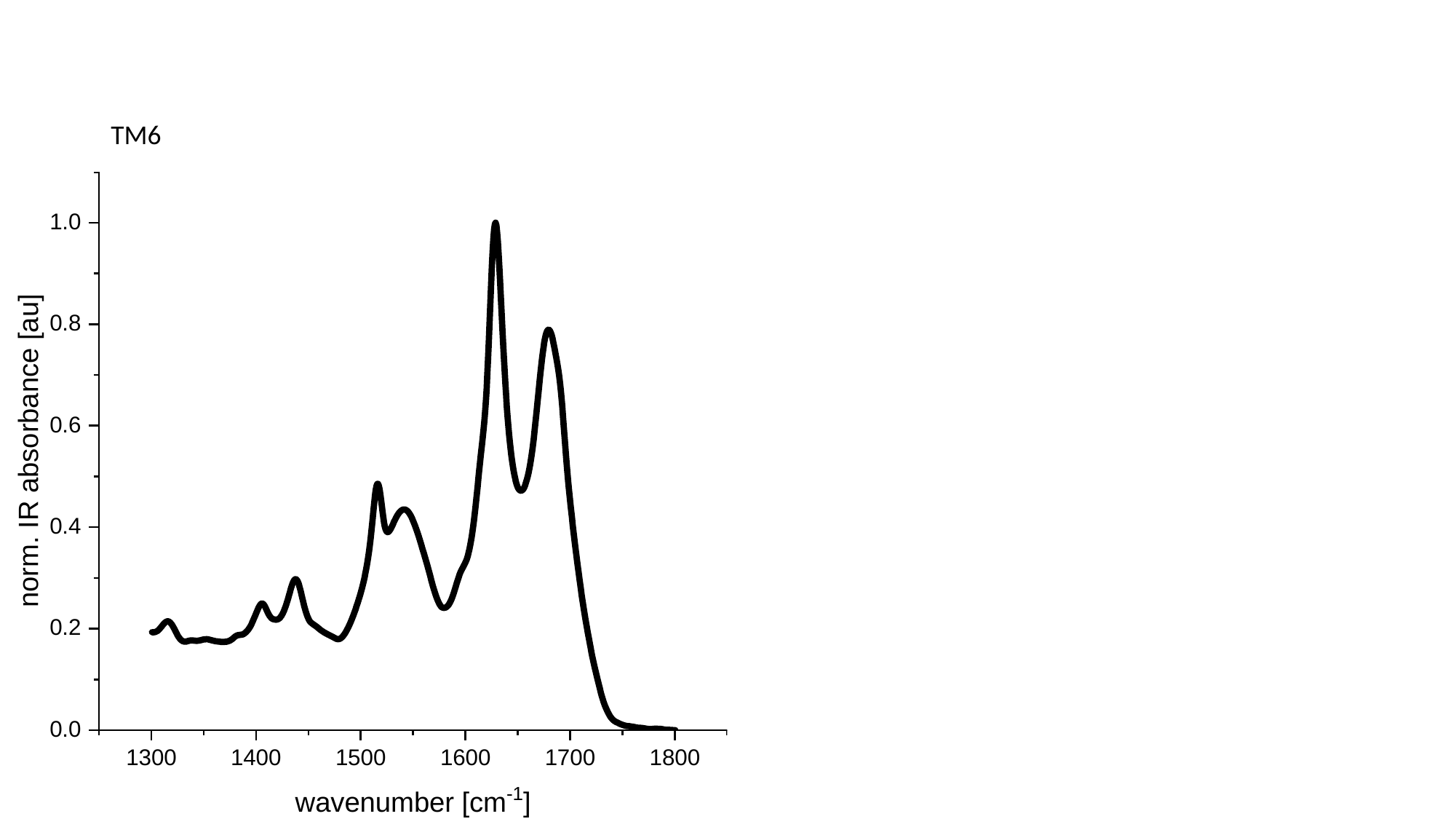

# TM6

## Slide 145
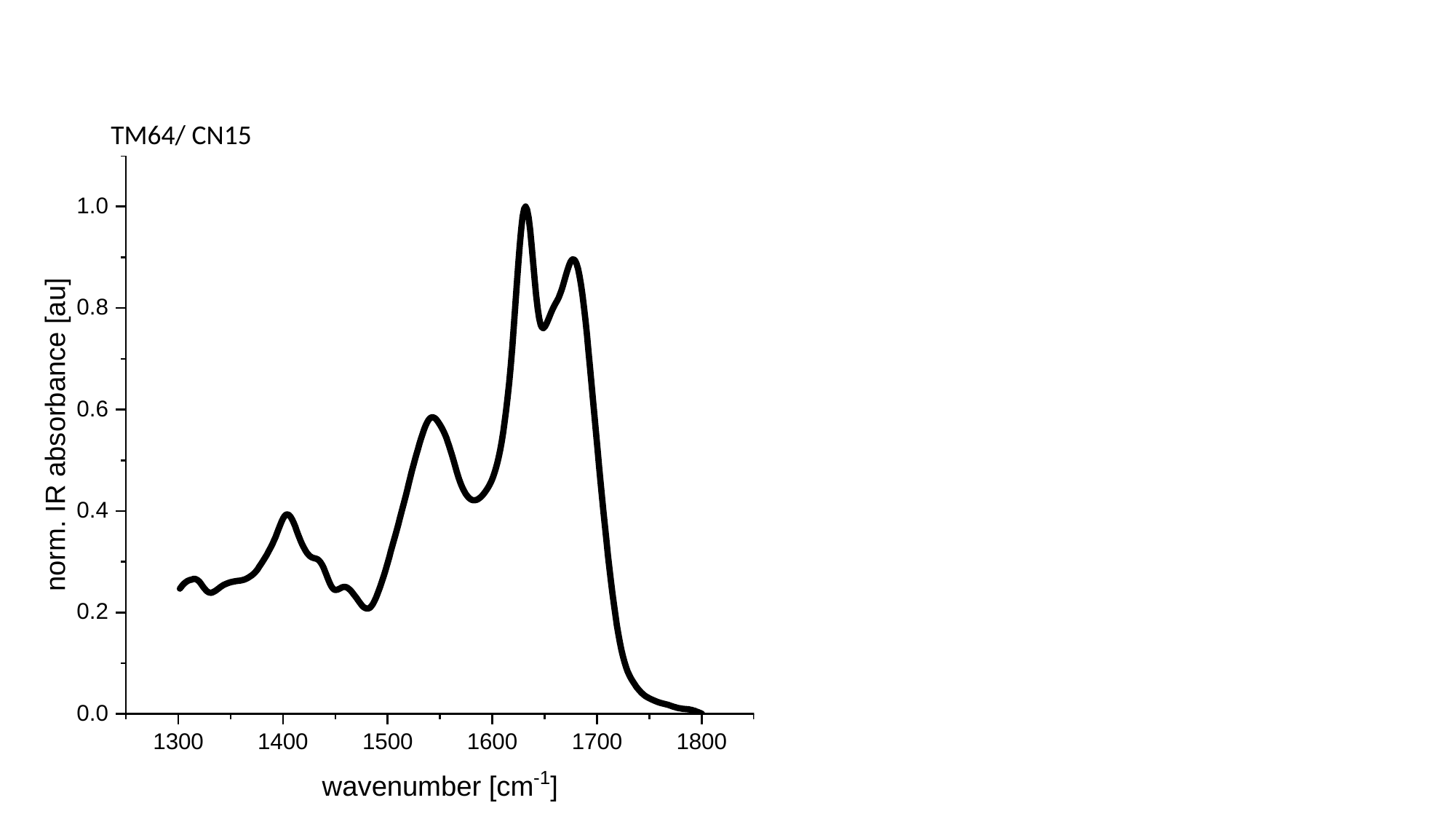

# TM64/ CN15

## Slide 146
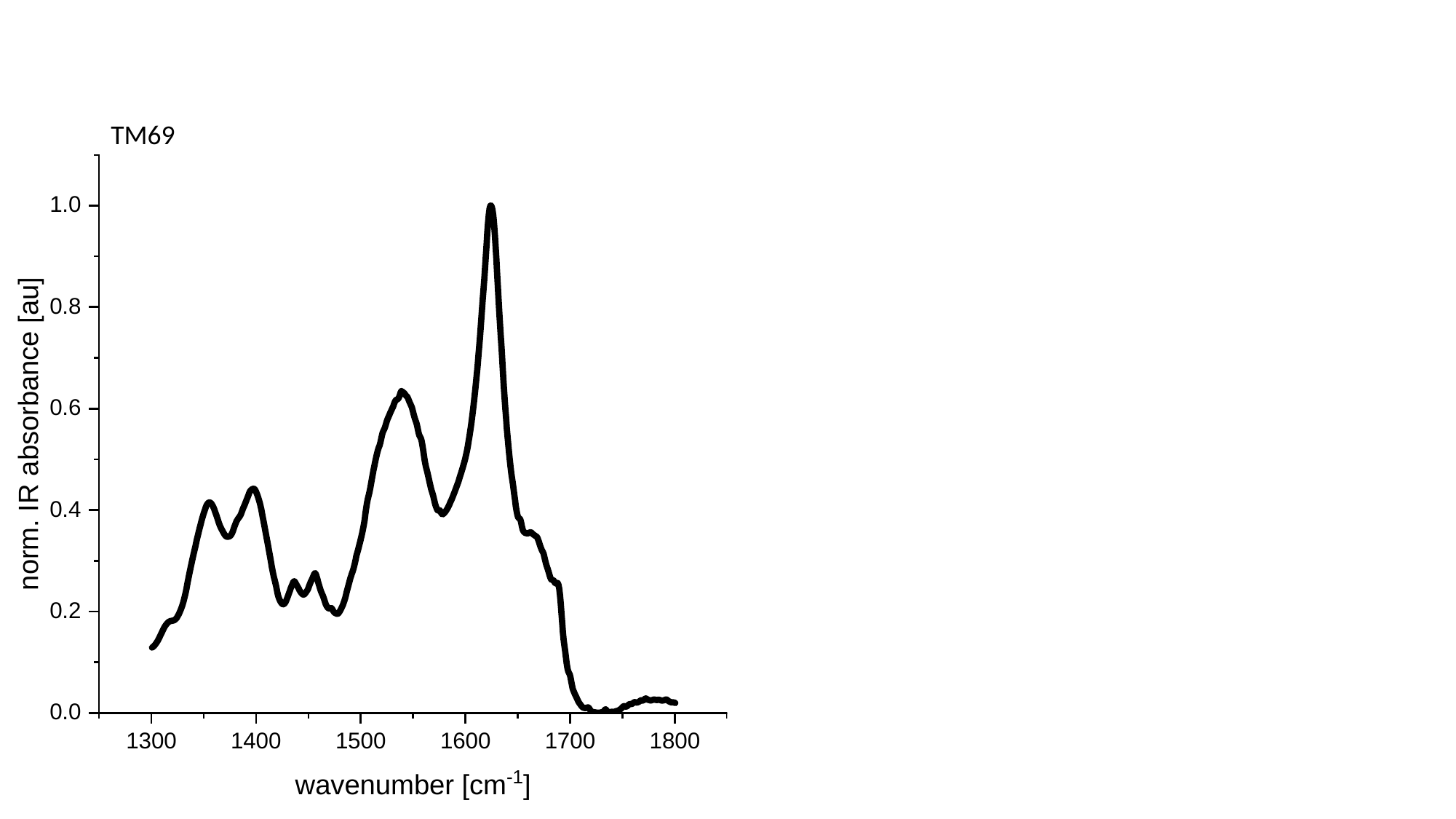

# TM69

## Slide 147
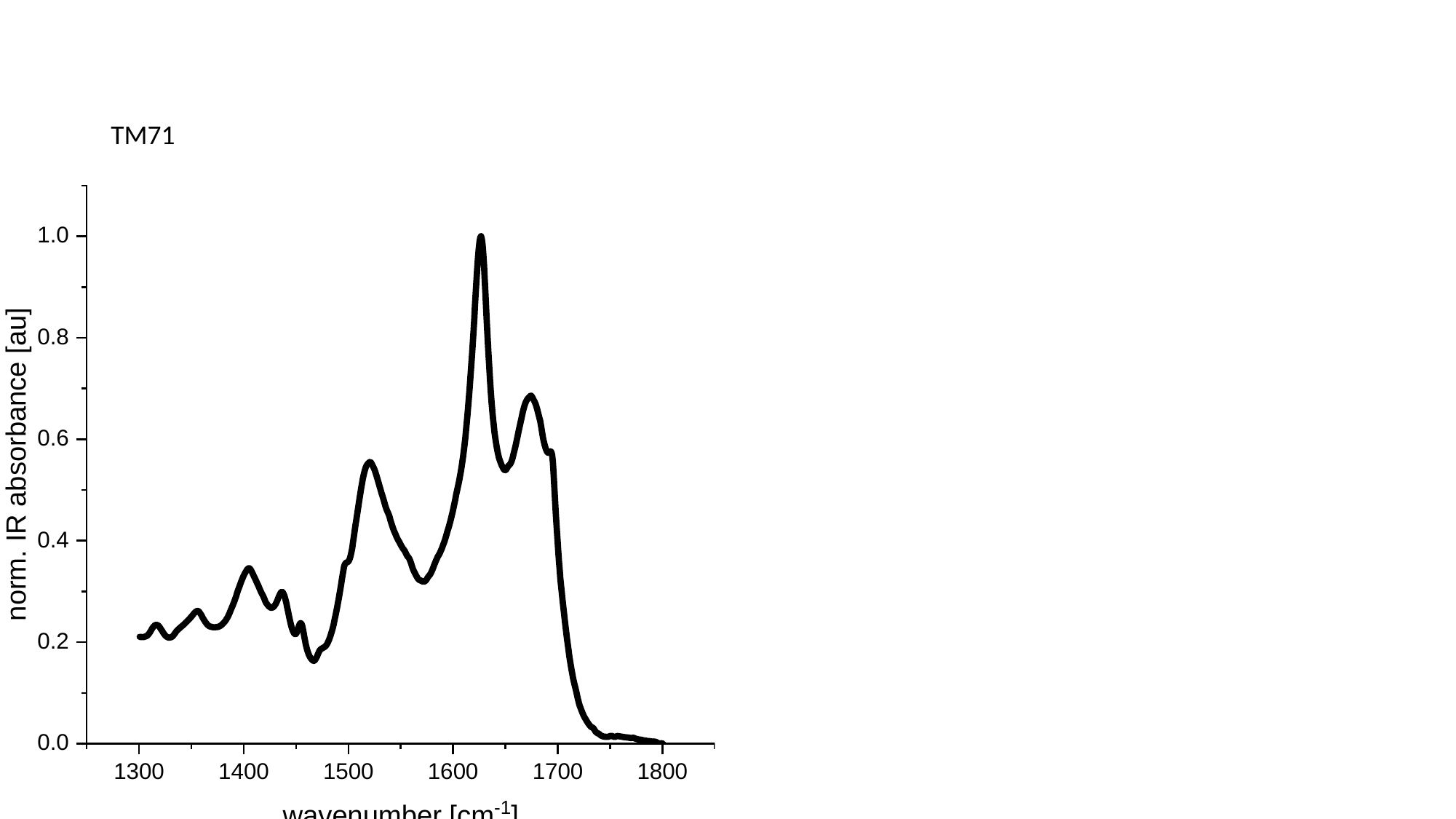

# TM71

## Slide 148
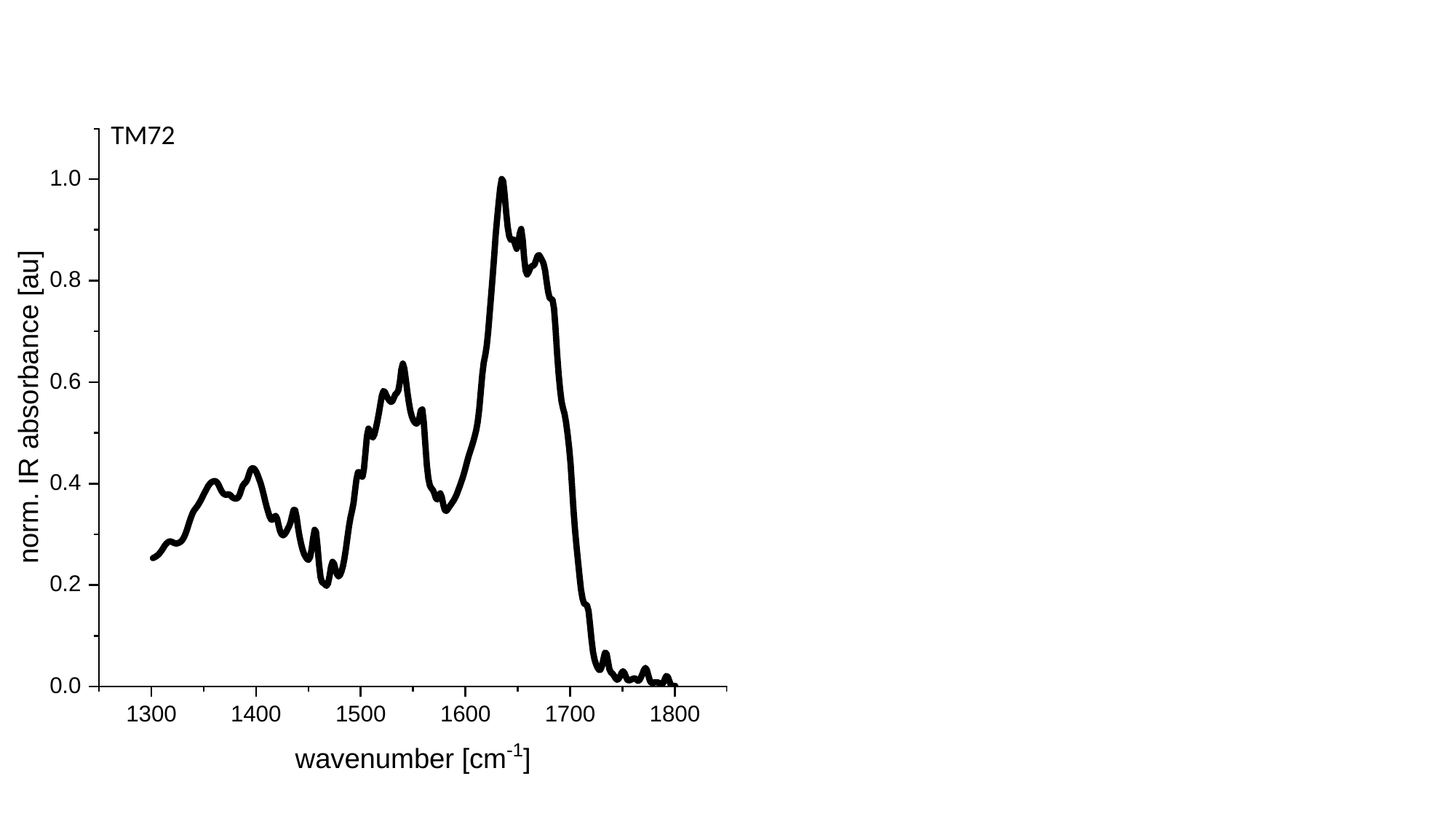

# TM72

## Slide 149
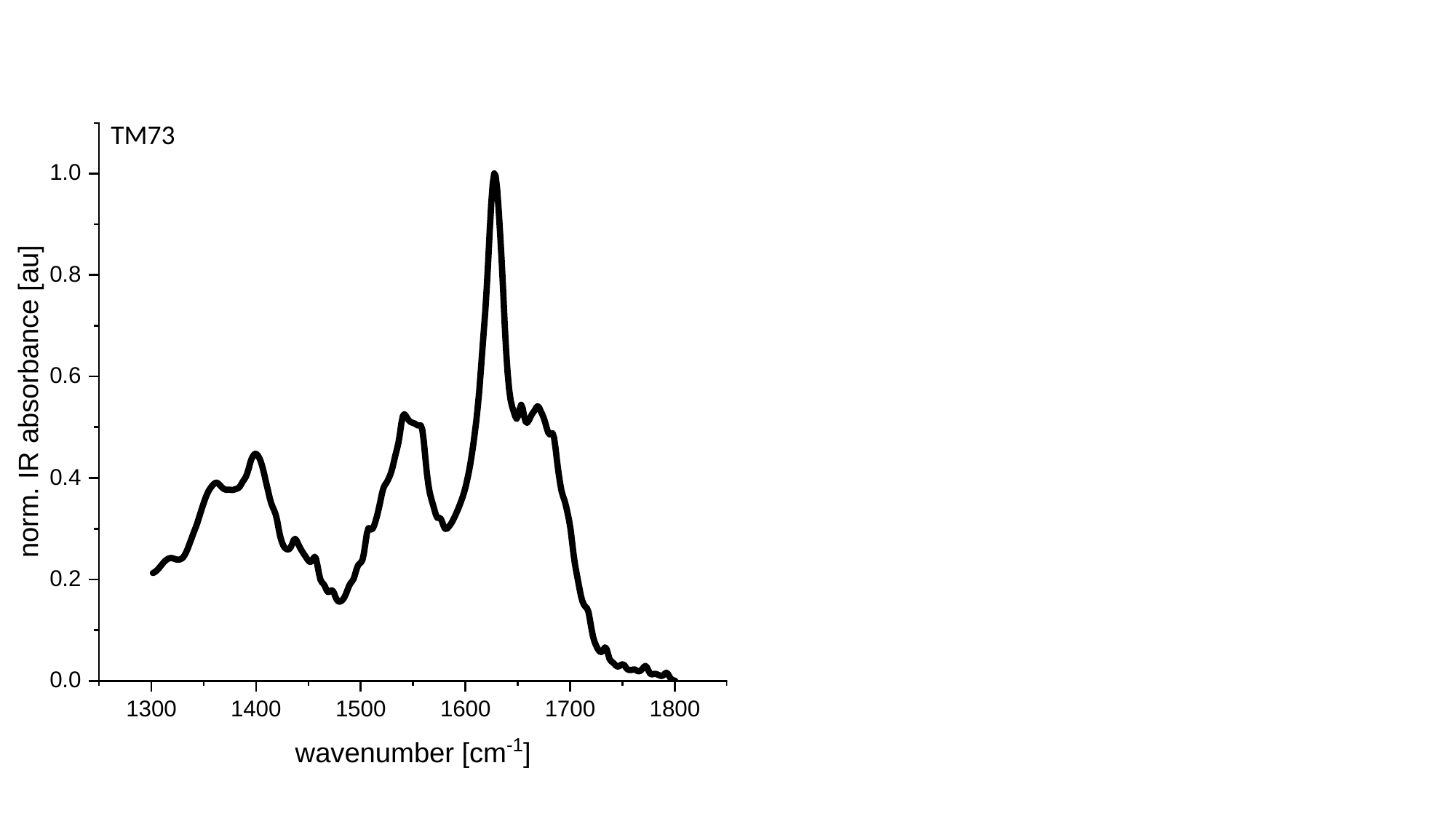

# TM73

## Slide 150
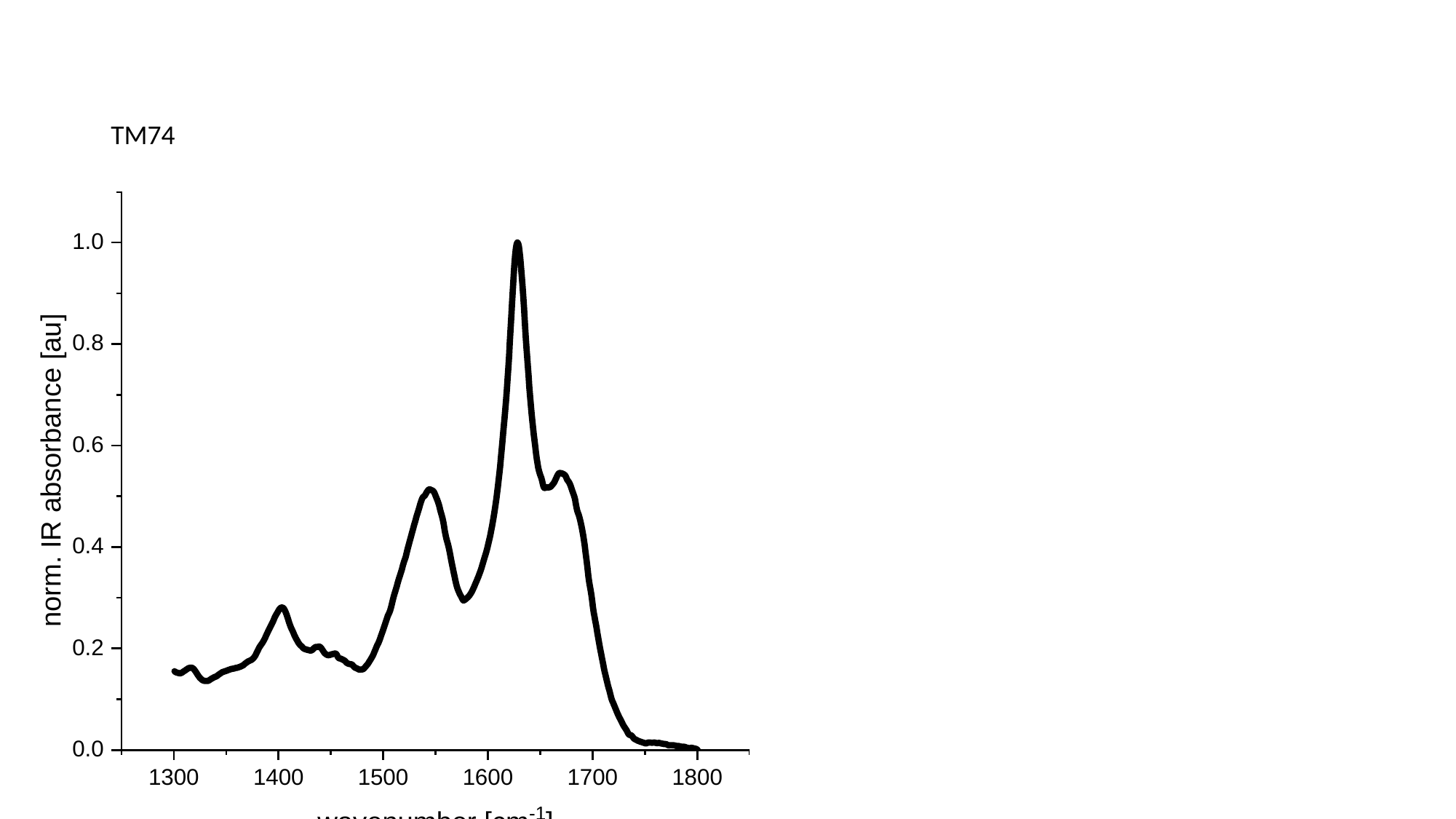

# TM74

## Slide 151
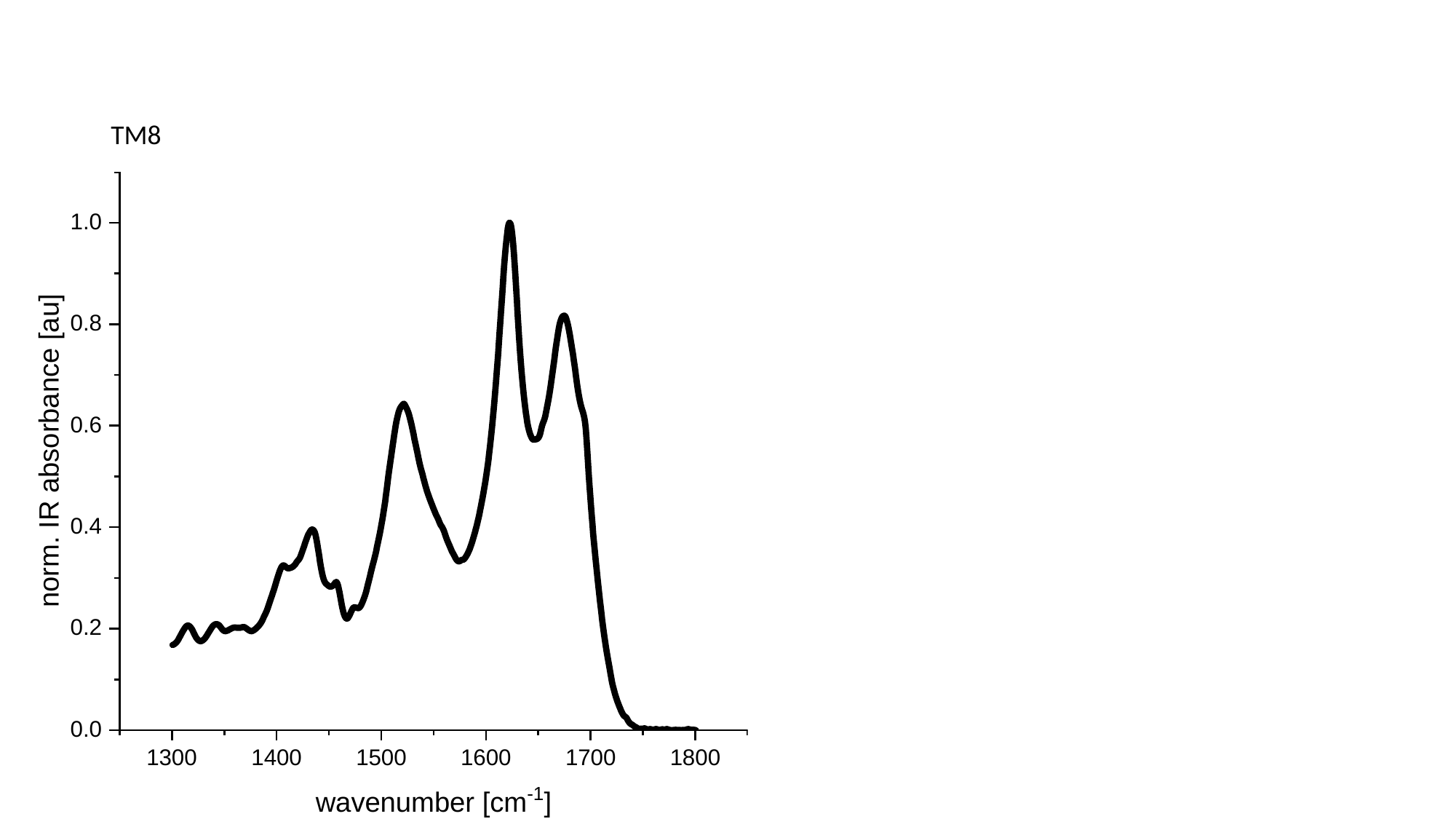

# TM8

## Slide 152
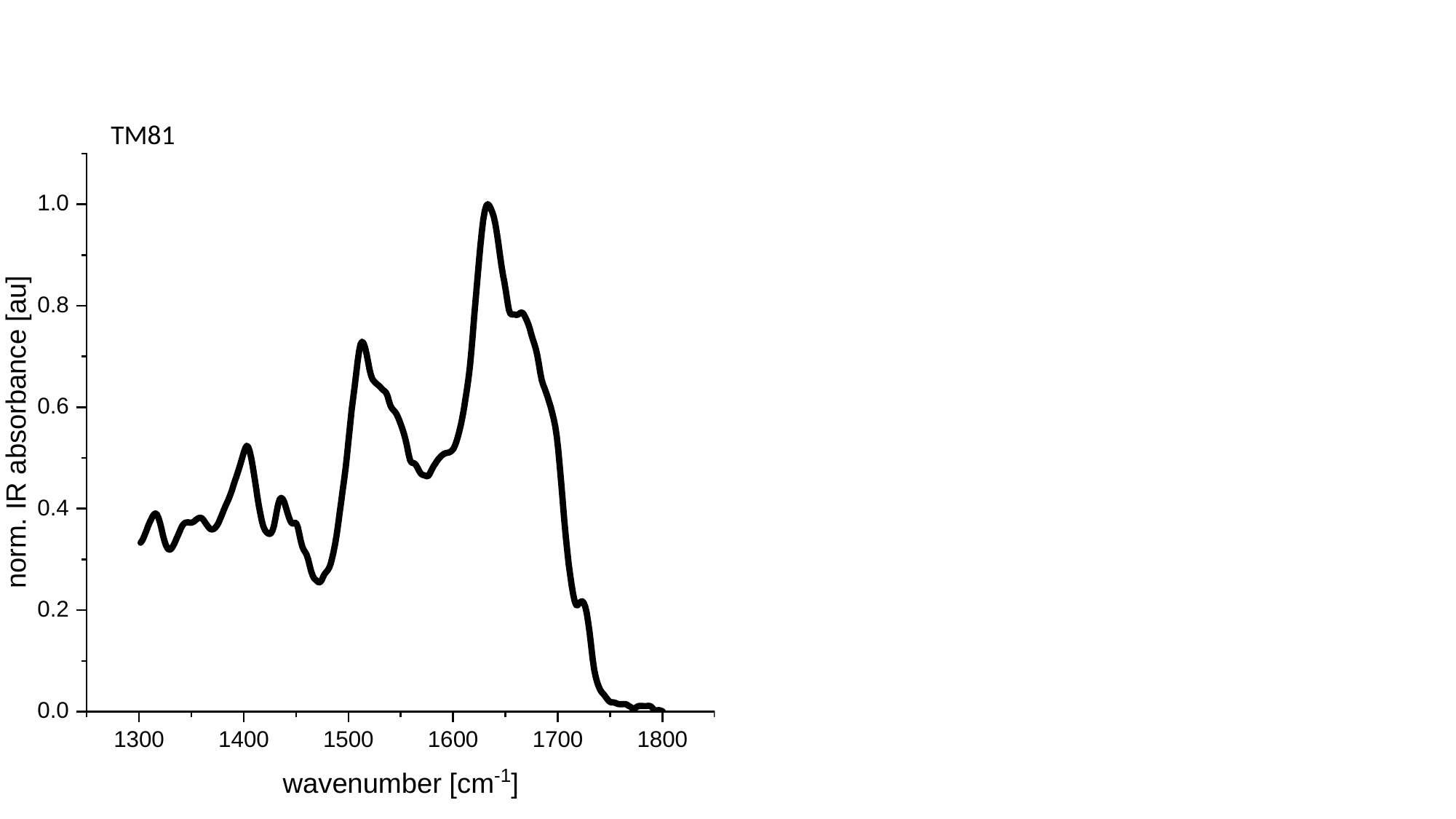

# TM81

## Slide 153
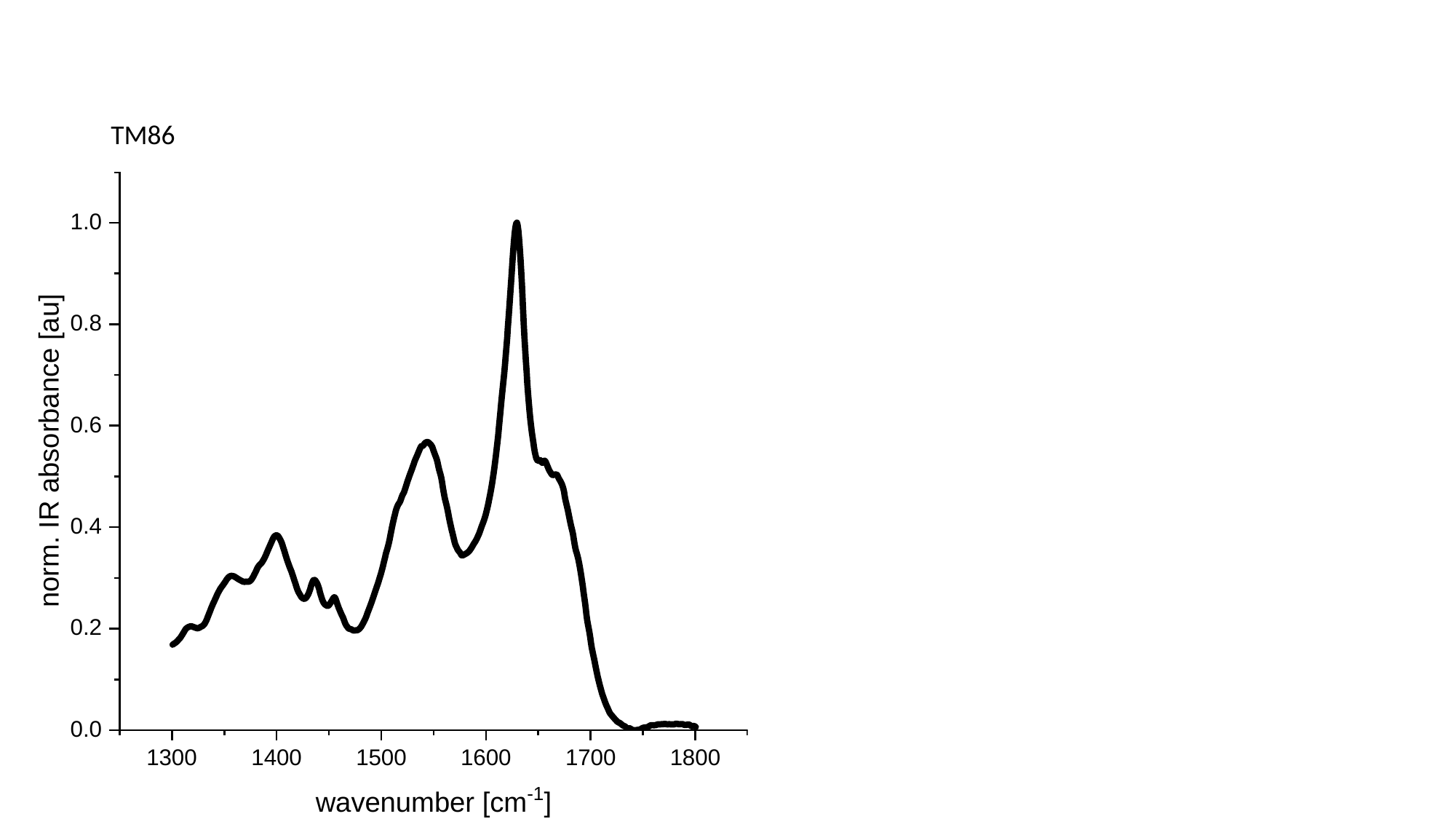

# TM86

## Slide 154
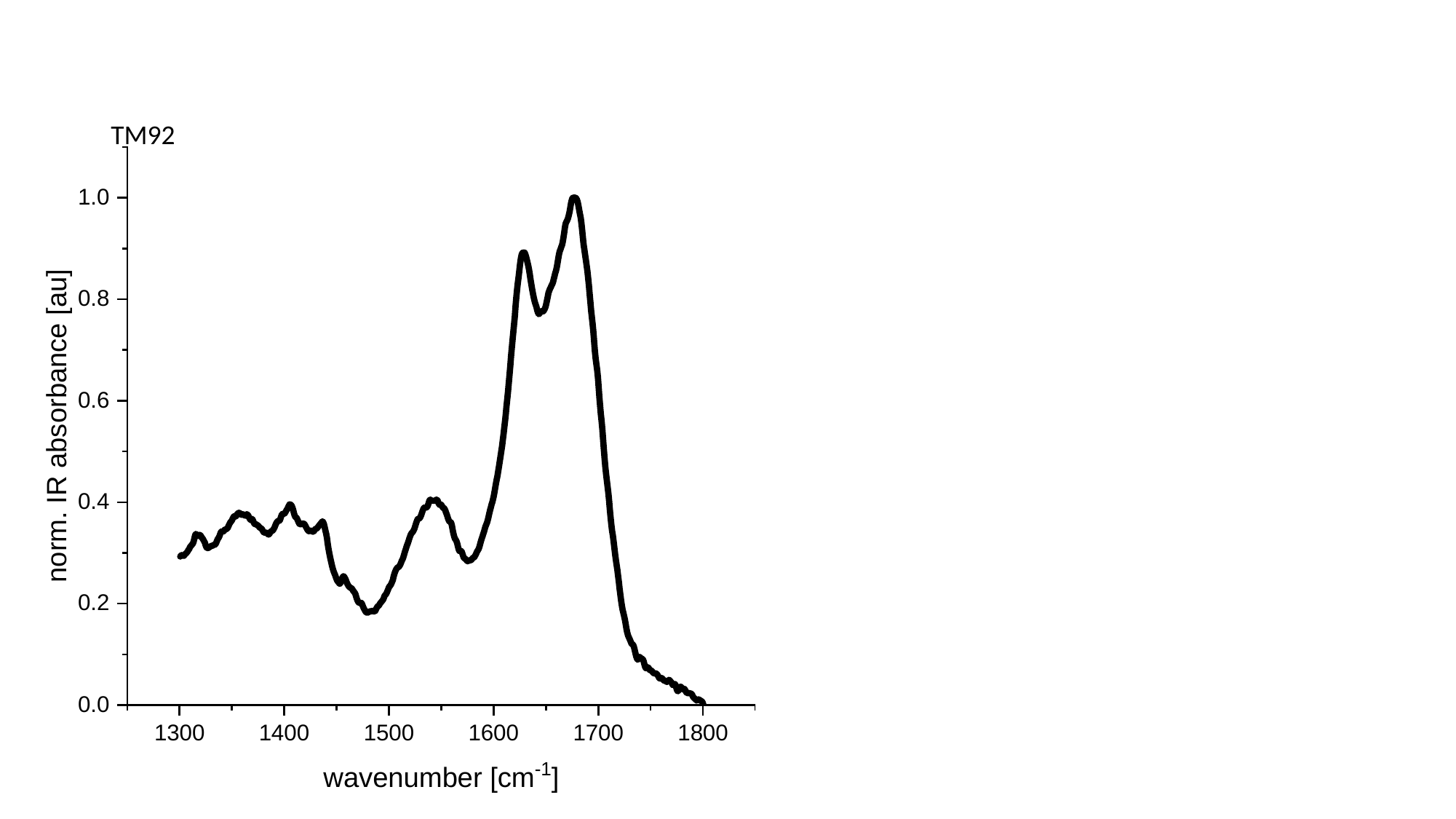

# TM92

## Slide 155
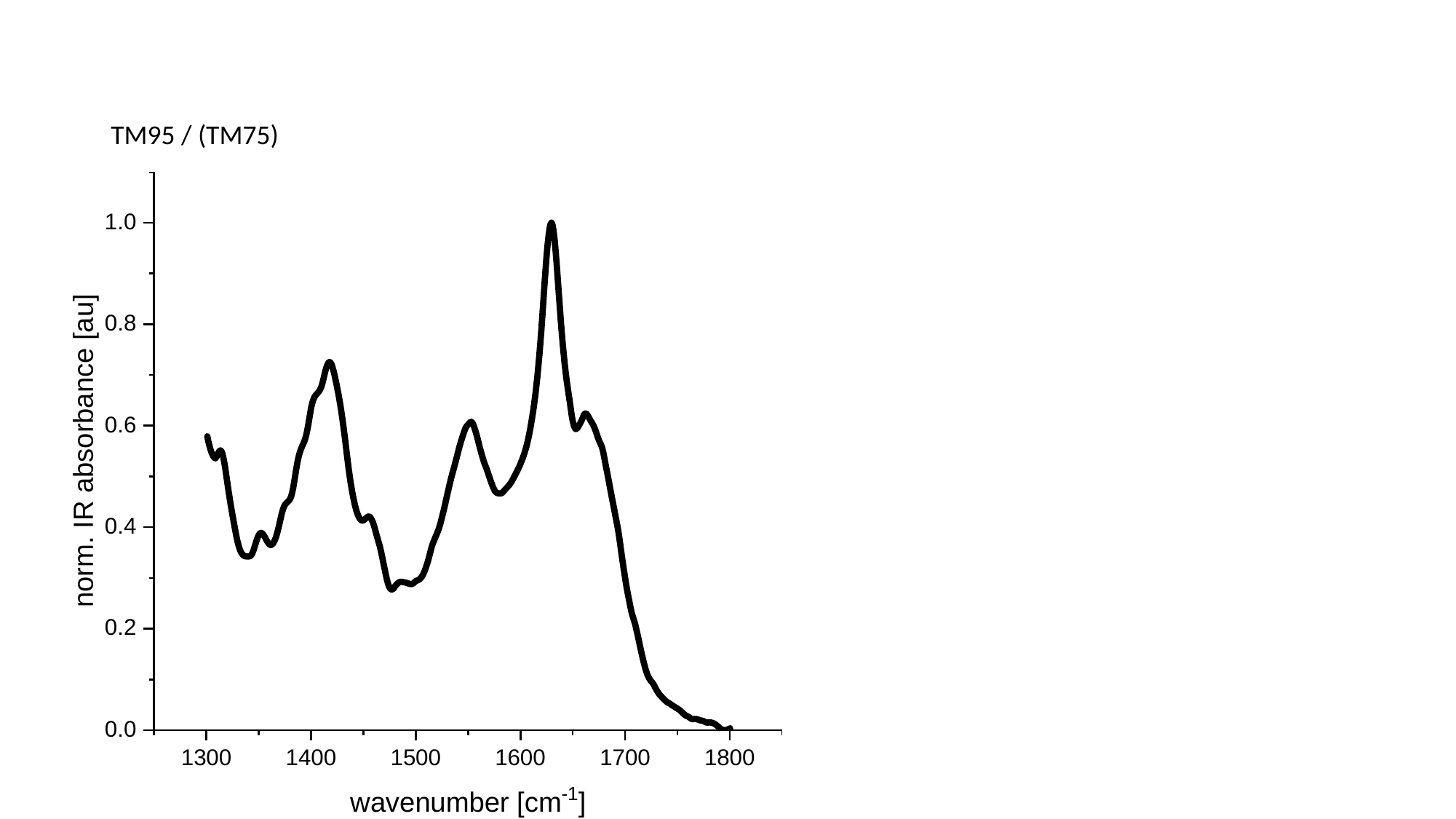

# TM95 / (TM75)

## Slide 156
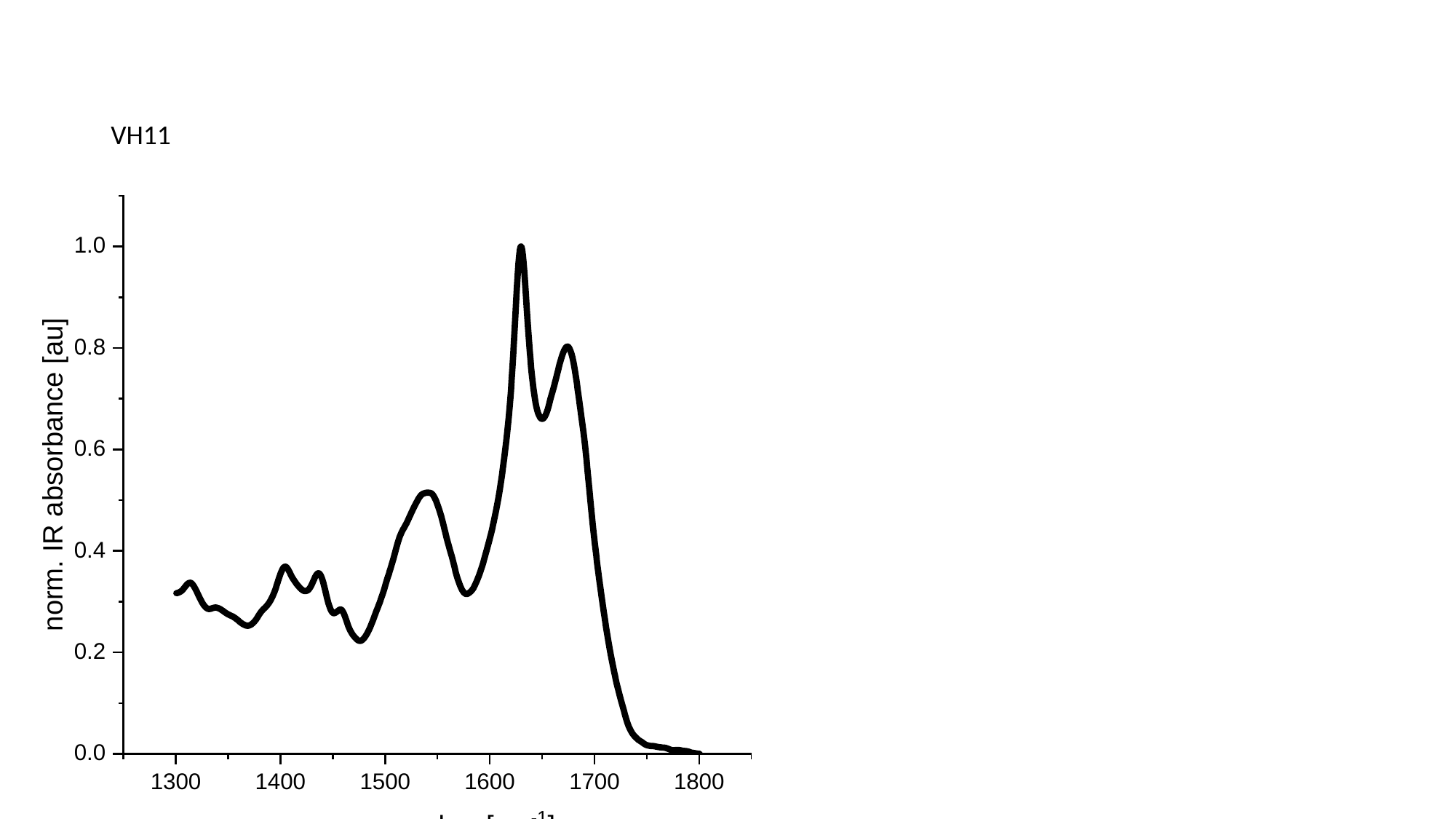

# VH11

## Slide 157
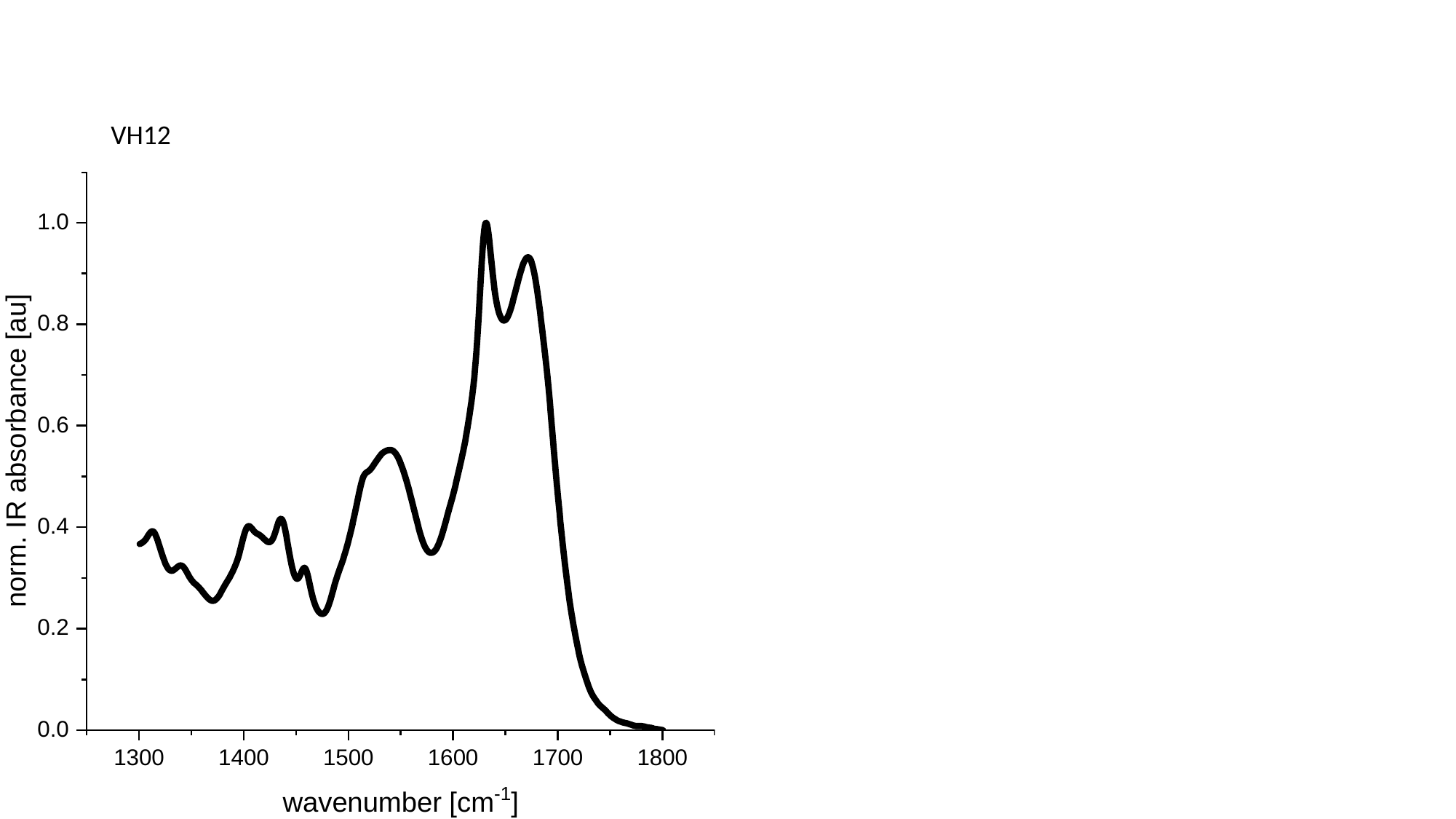

# VH12

## Slide 158
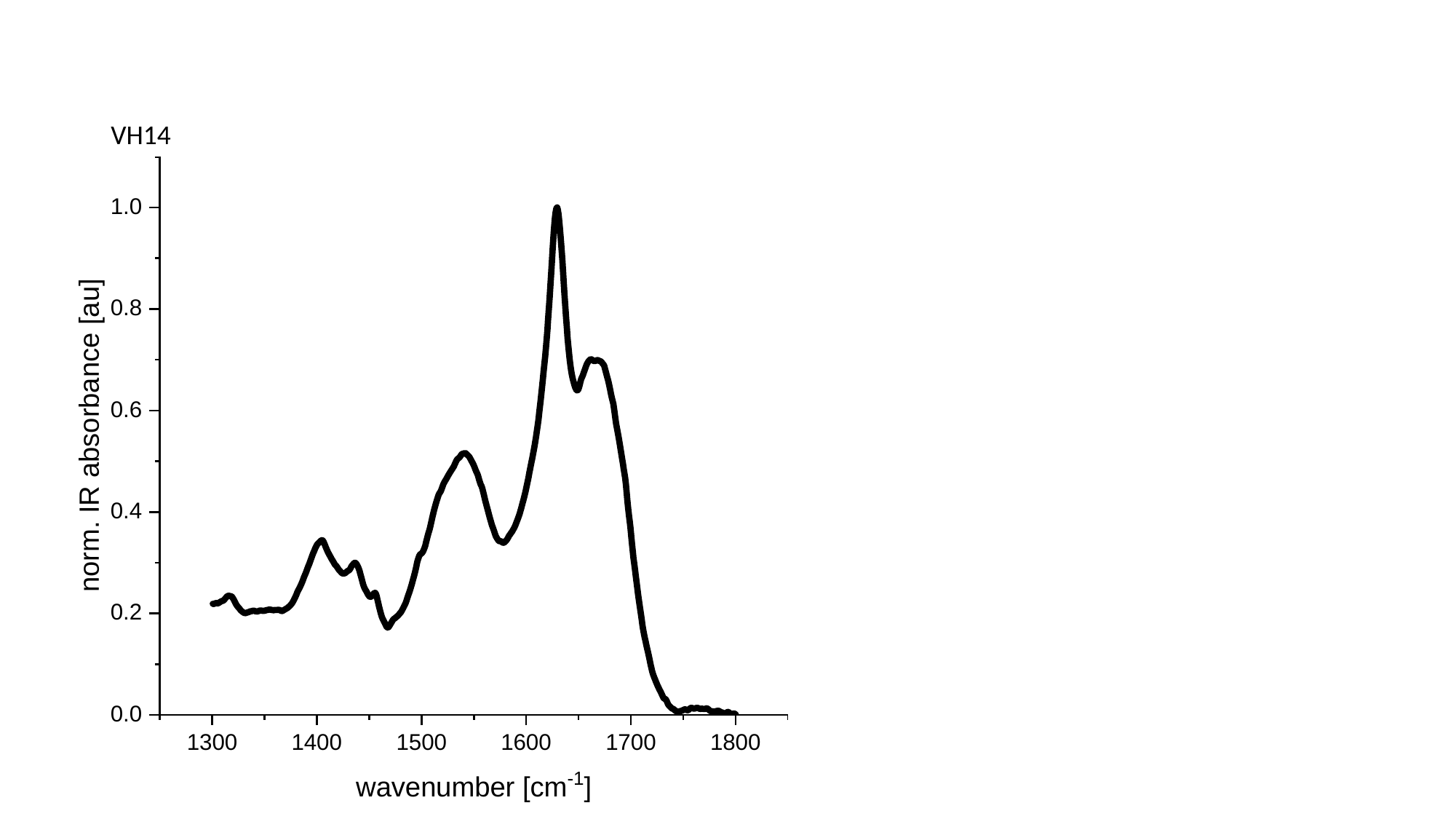

# VH14

## Slide 159
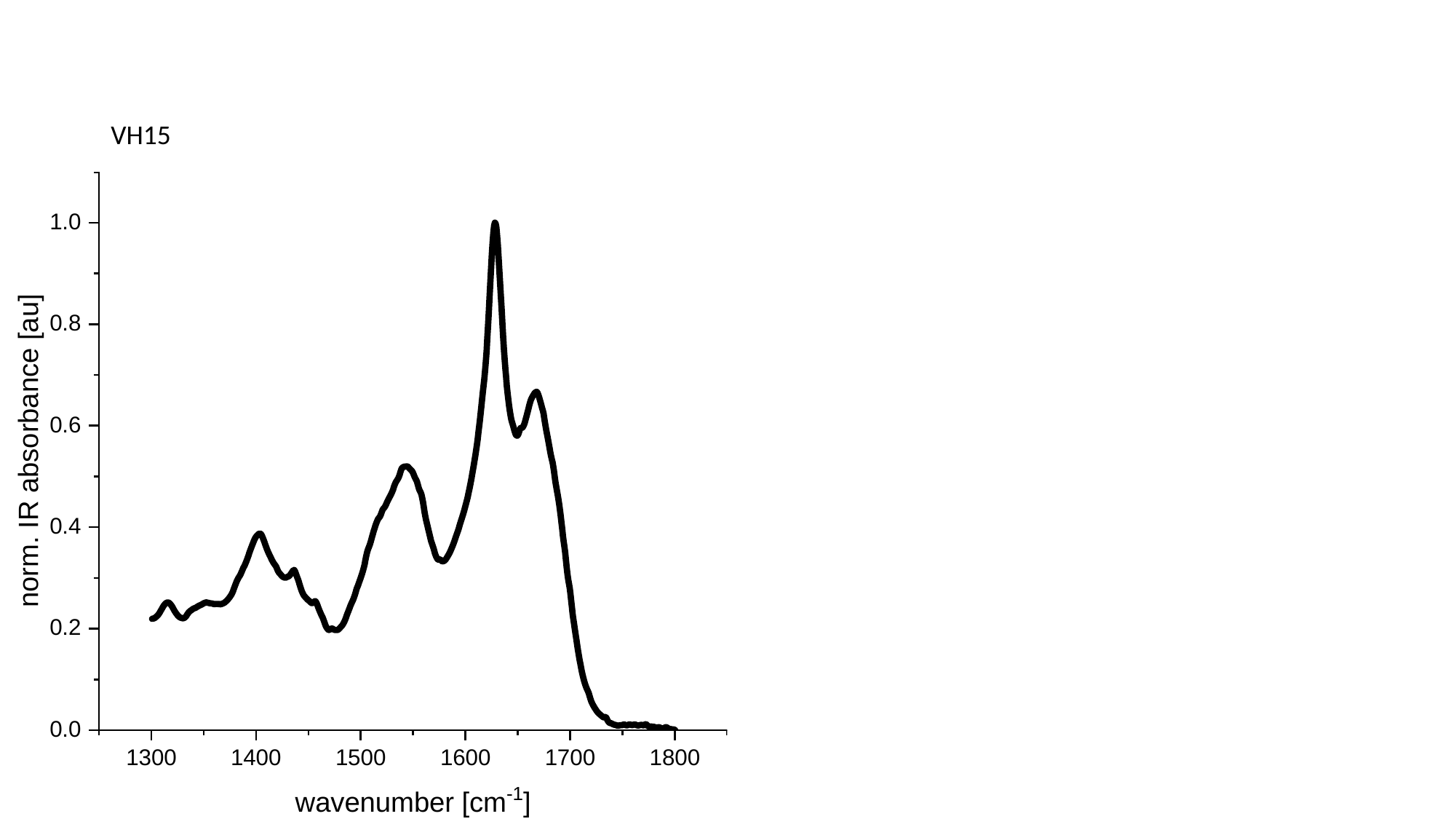

# VH15

## Slide 160
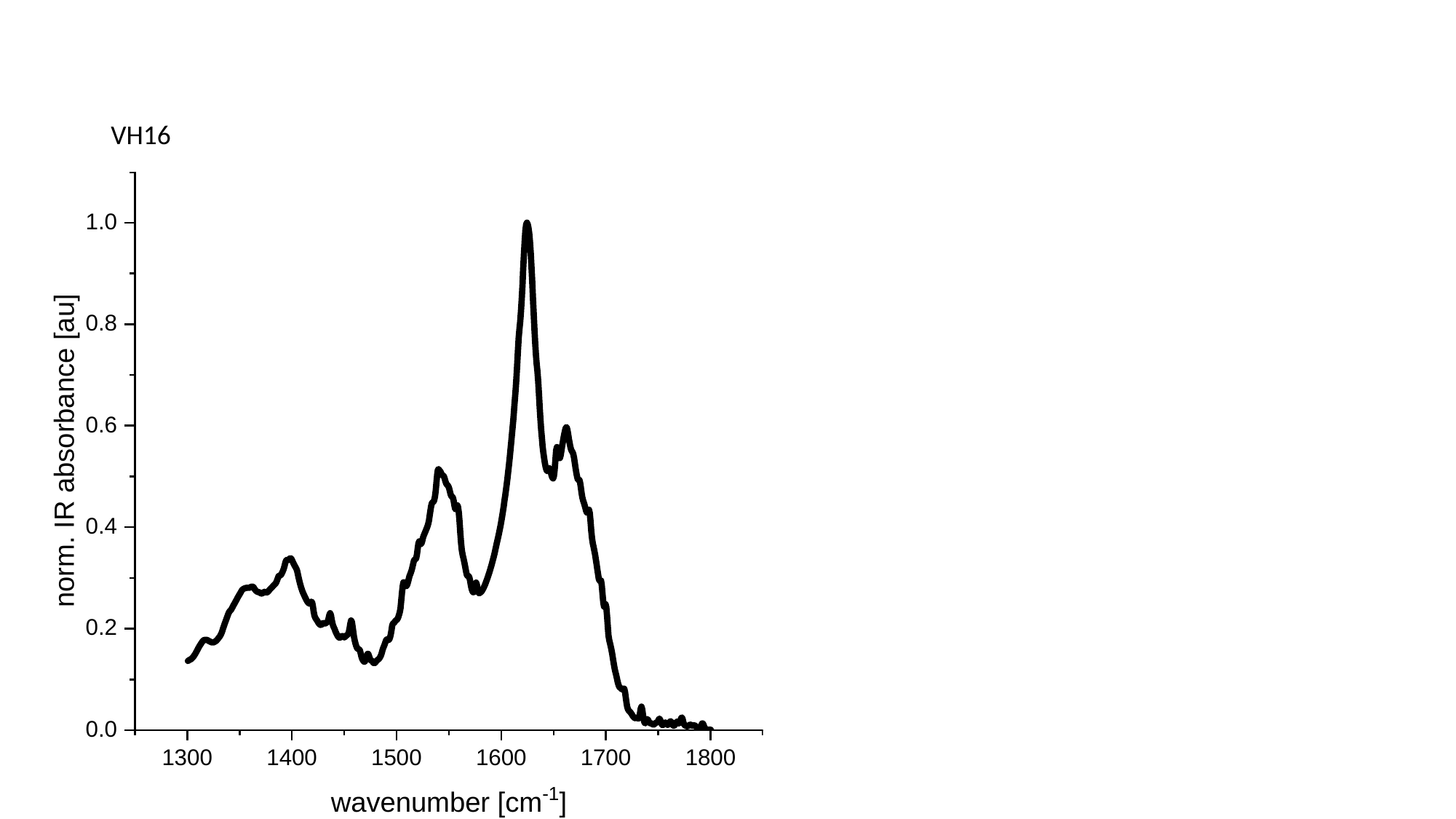

# VH16

## Slide 161
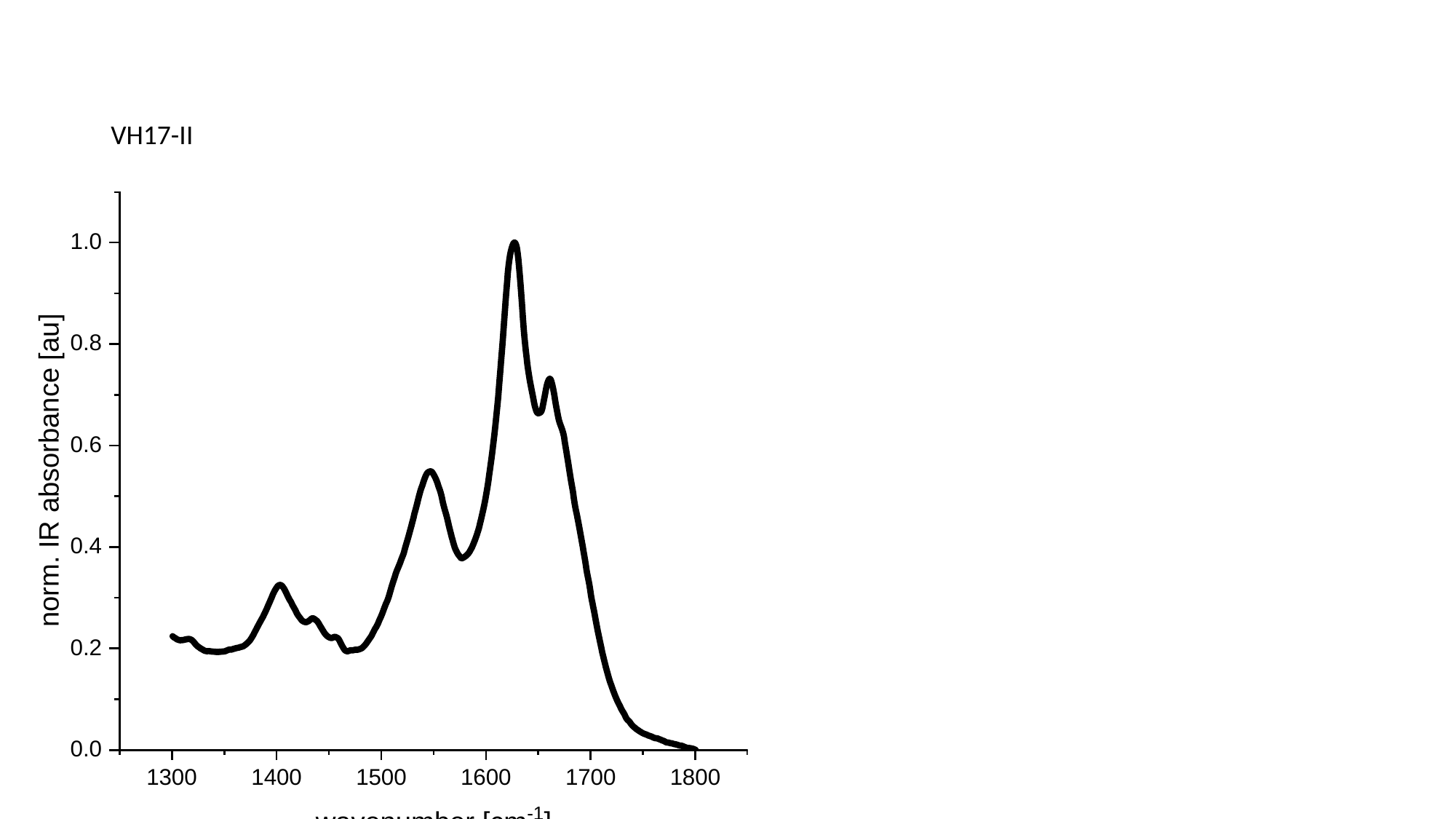

# VH17-II

## Slide 162
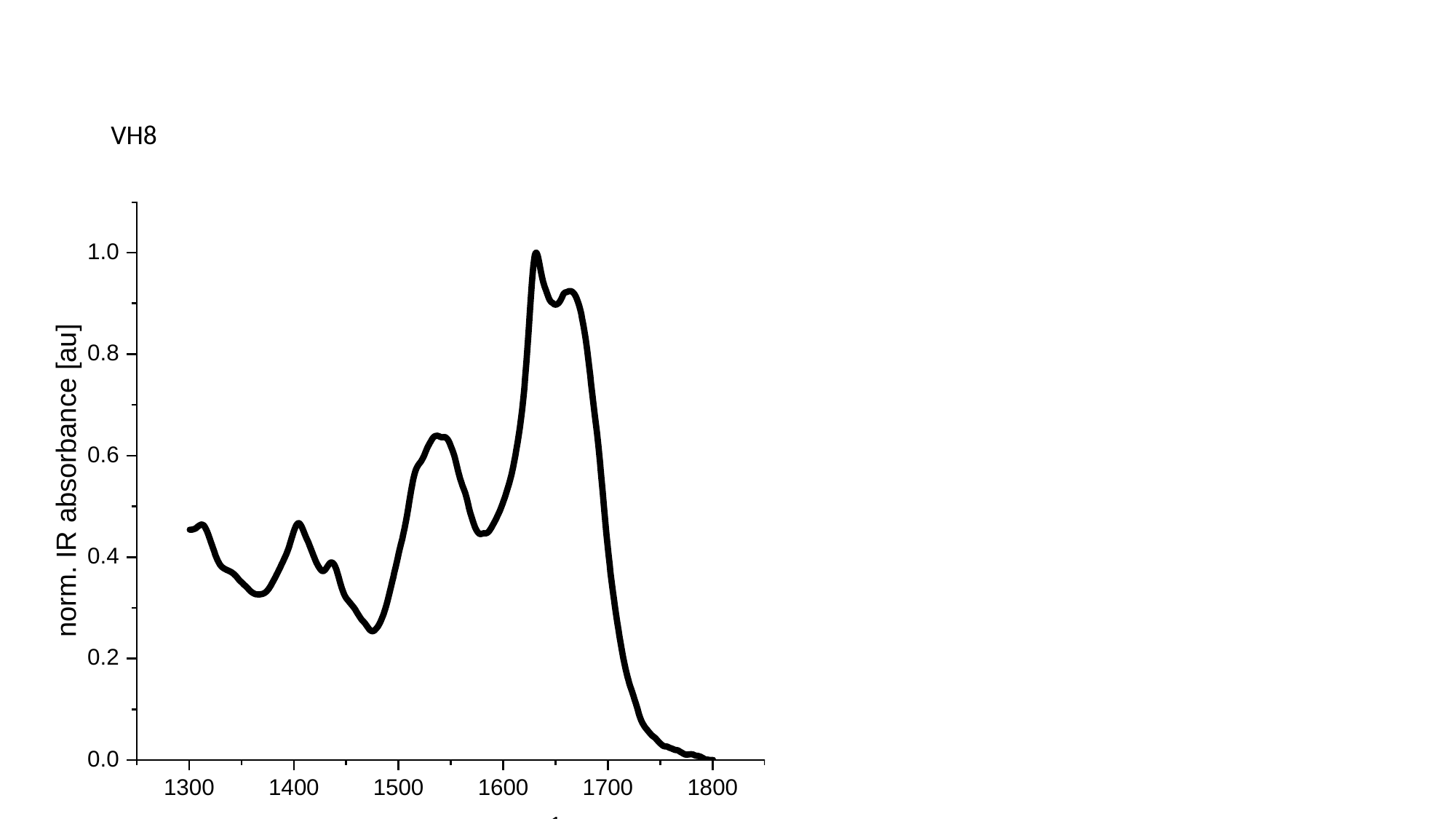

# VH8

## Slide 163
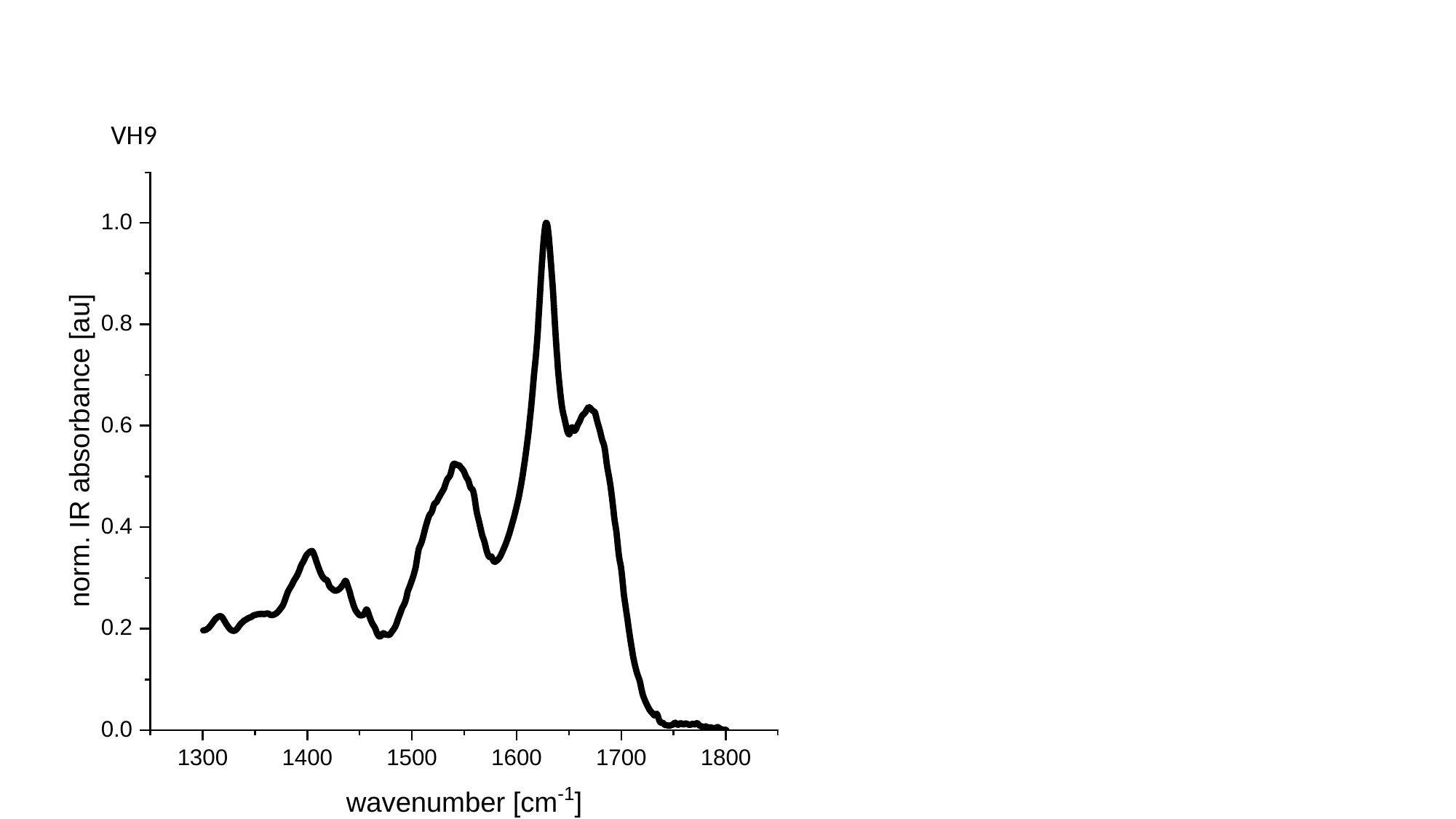

# VH9

## Slide 164
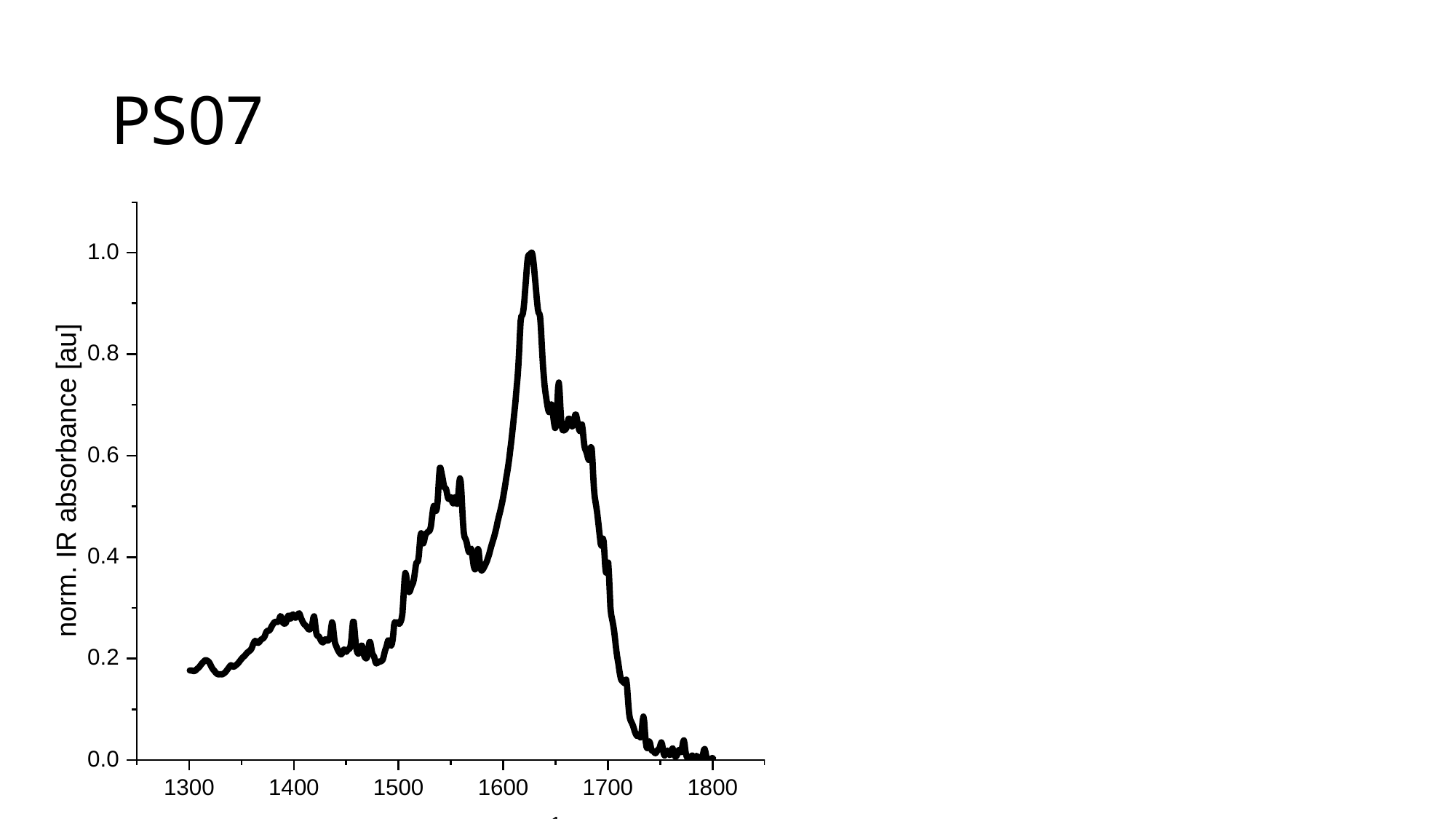

# PS07

## Slide 165
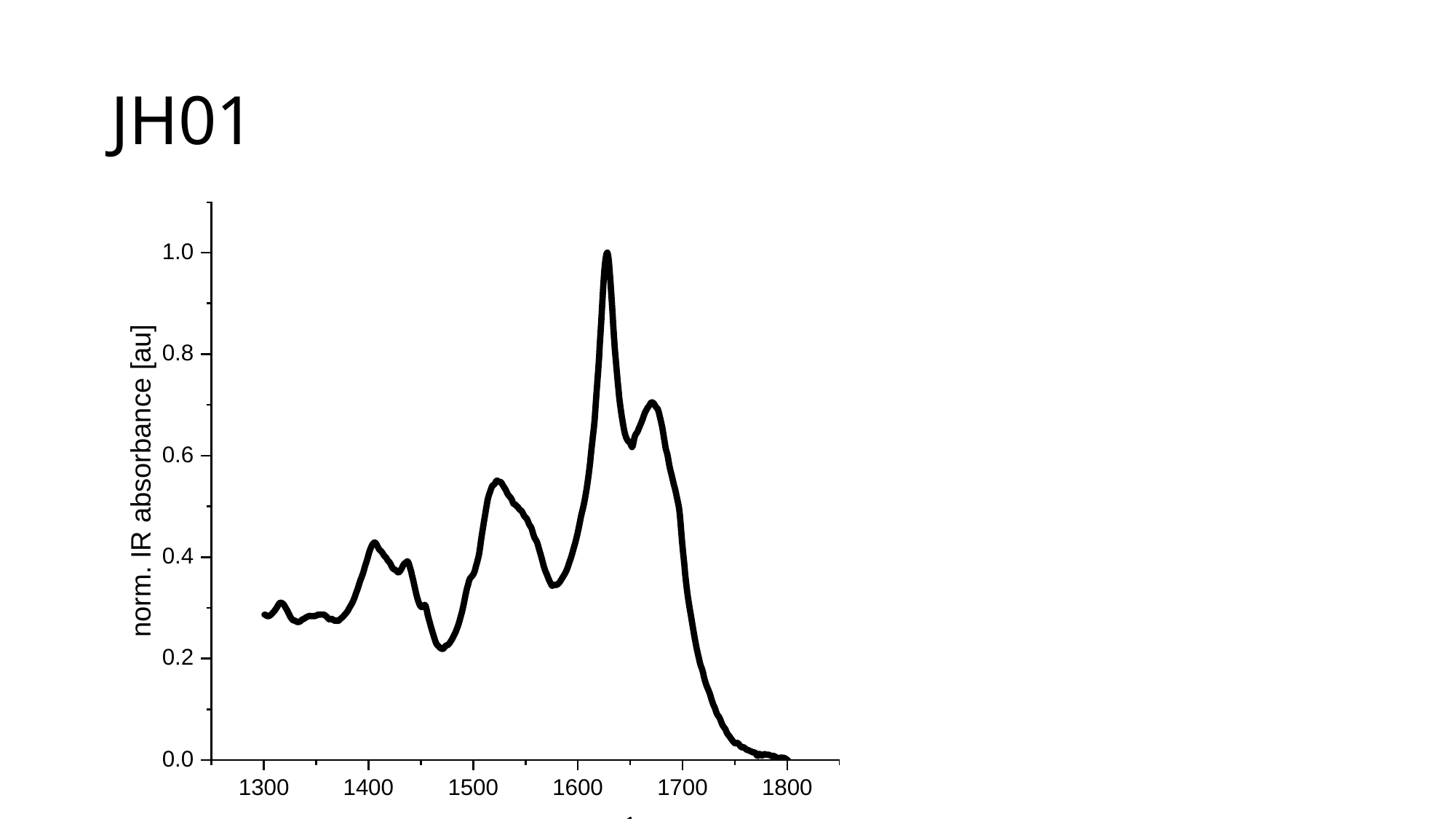

# JH01

## Slide 166
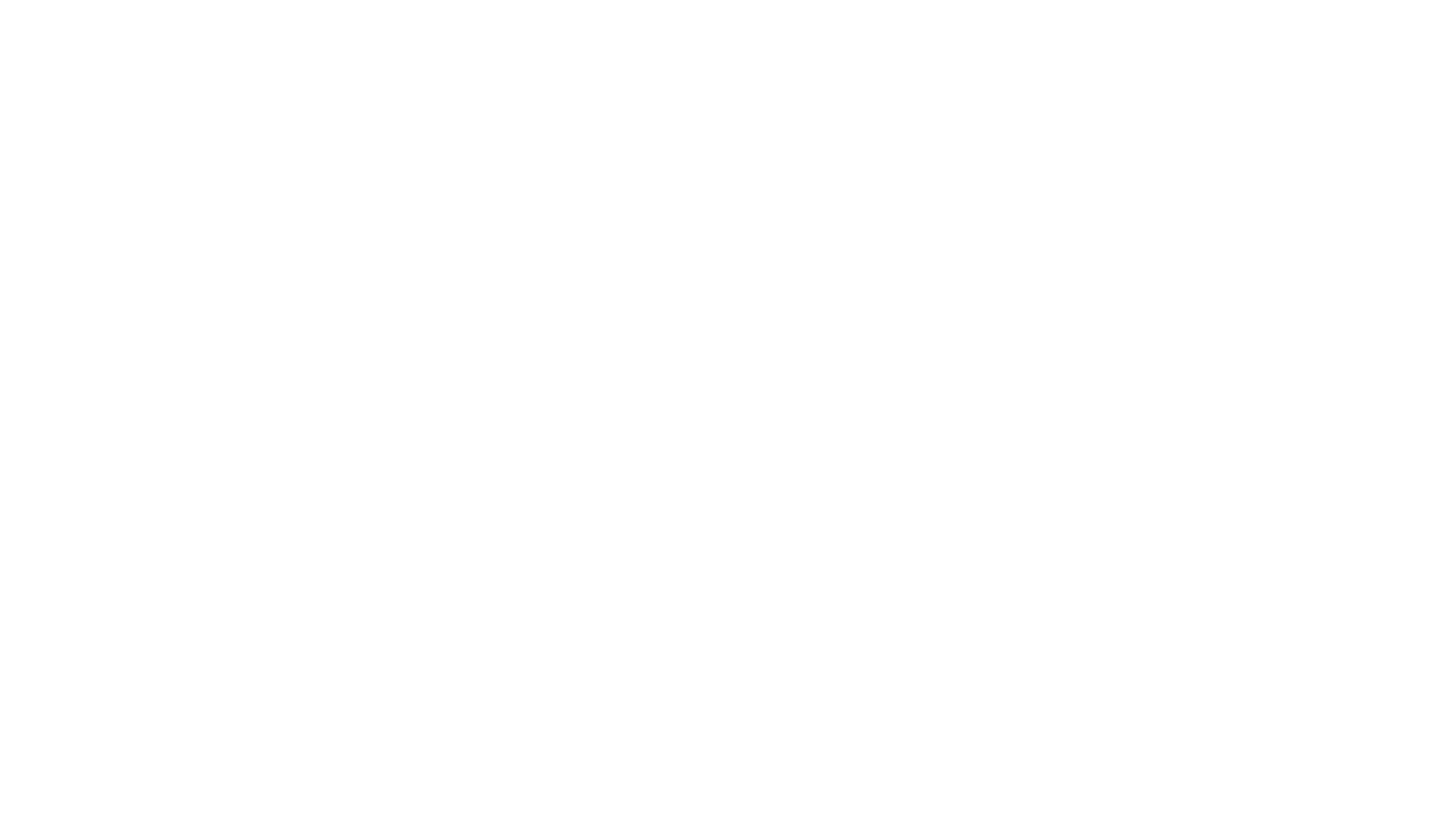

#

## Slide 167
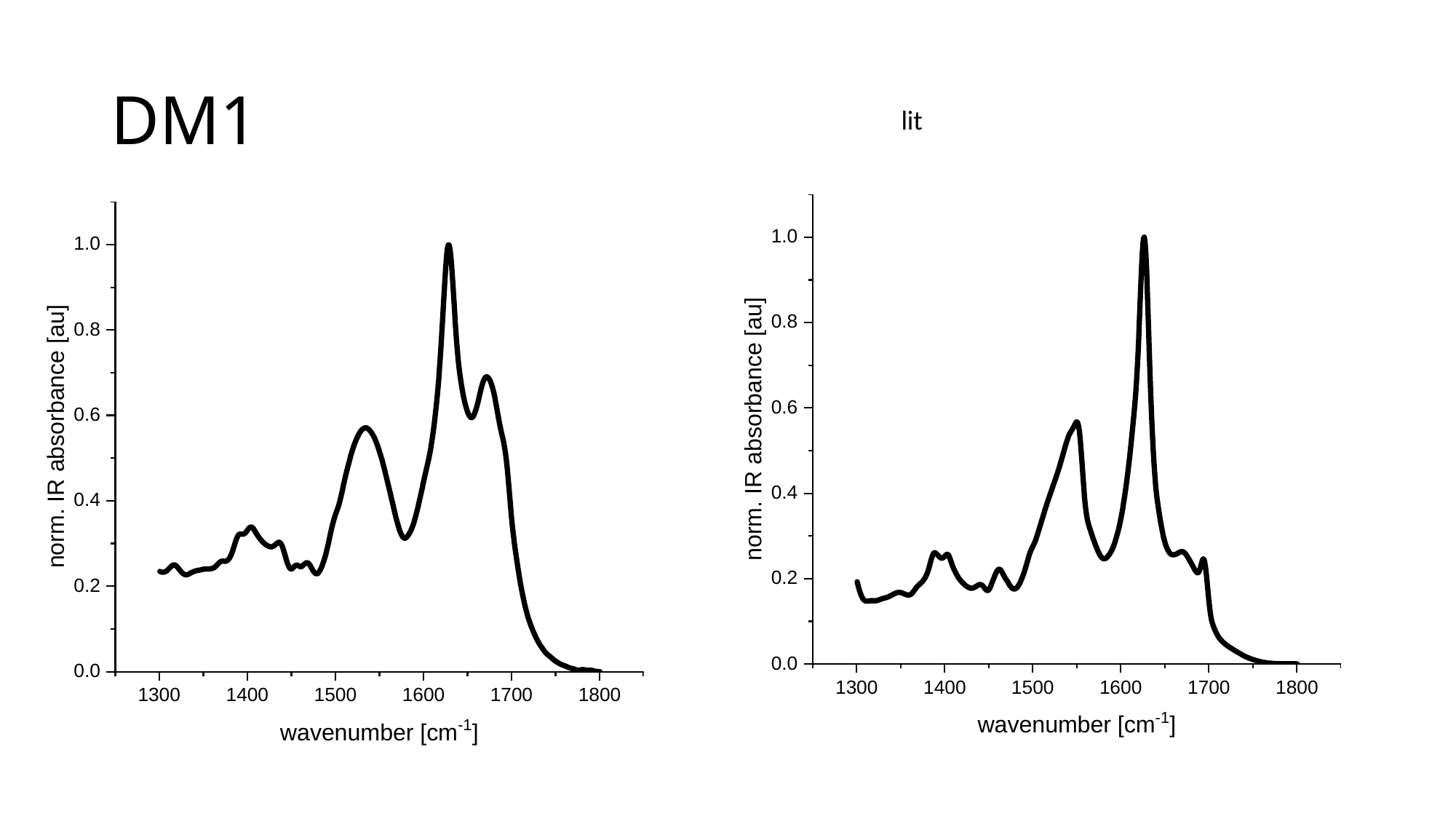

# DM1
lit

## Slide 168
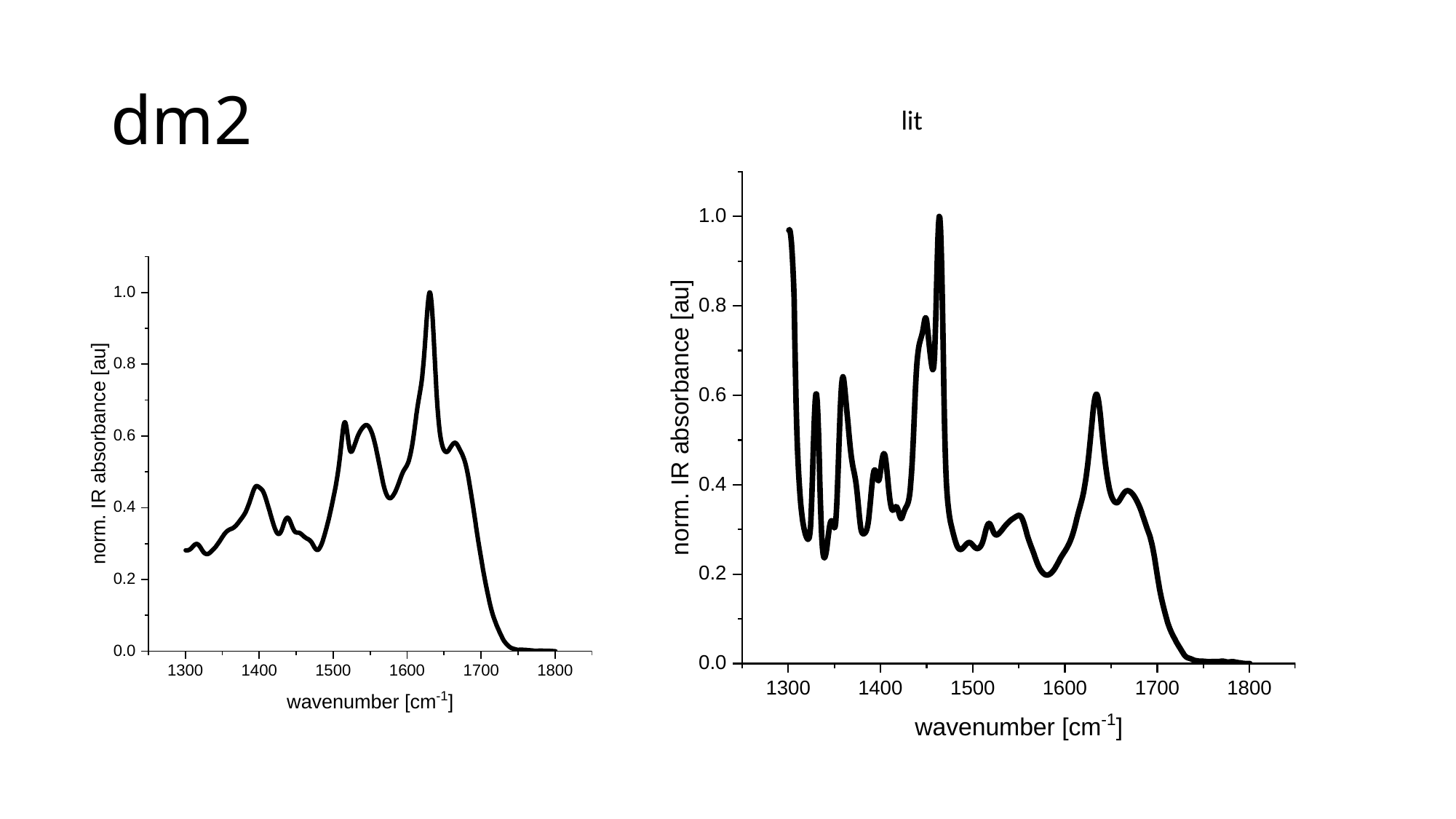

# dm2
lit

## Slide 169
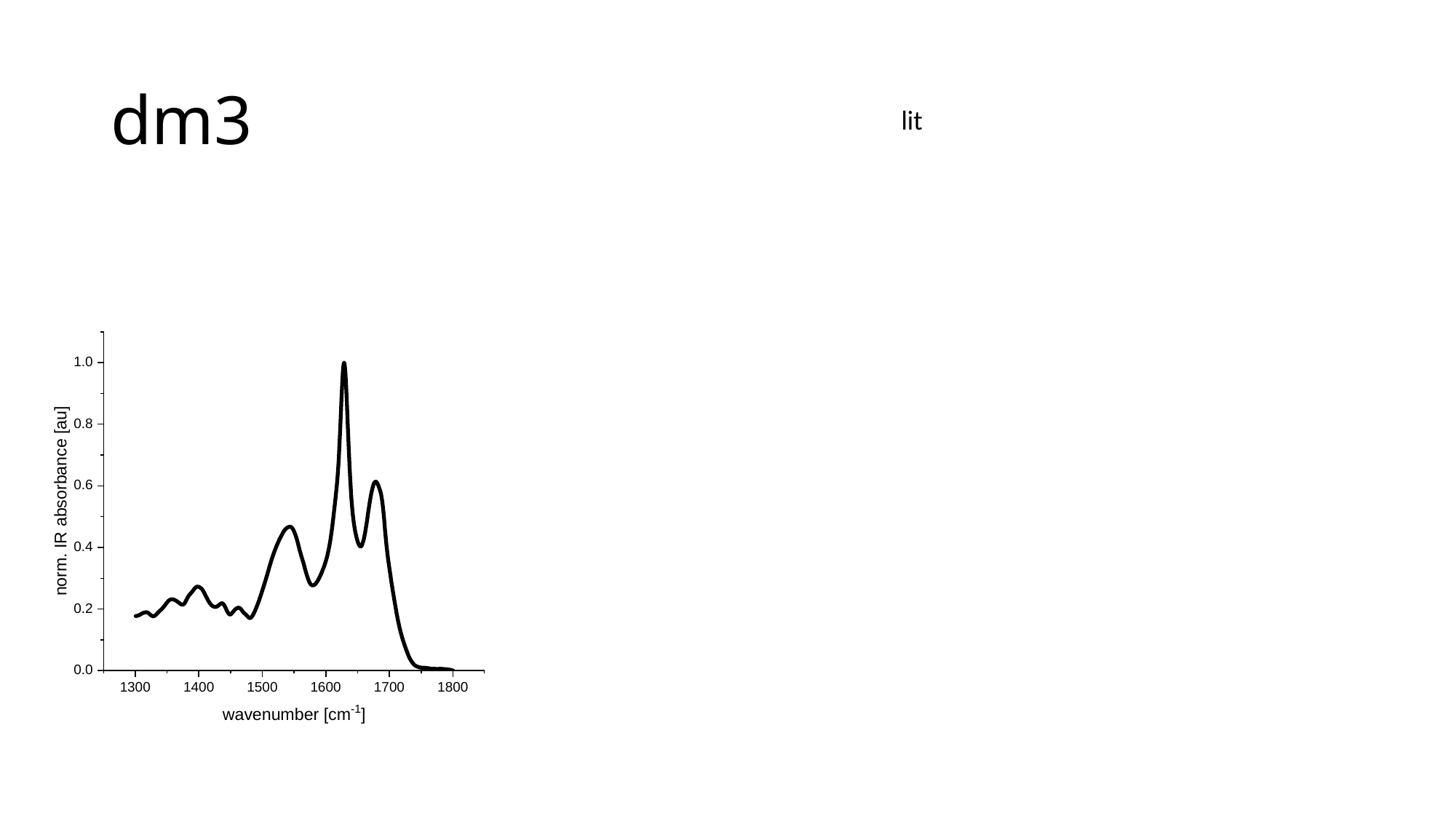

# dm3
lit

## Slide 170
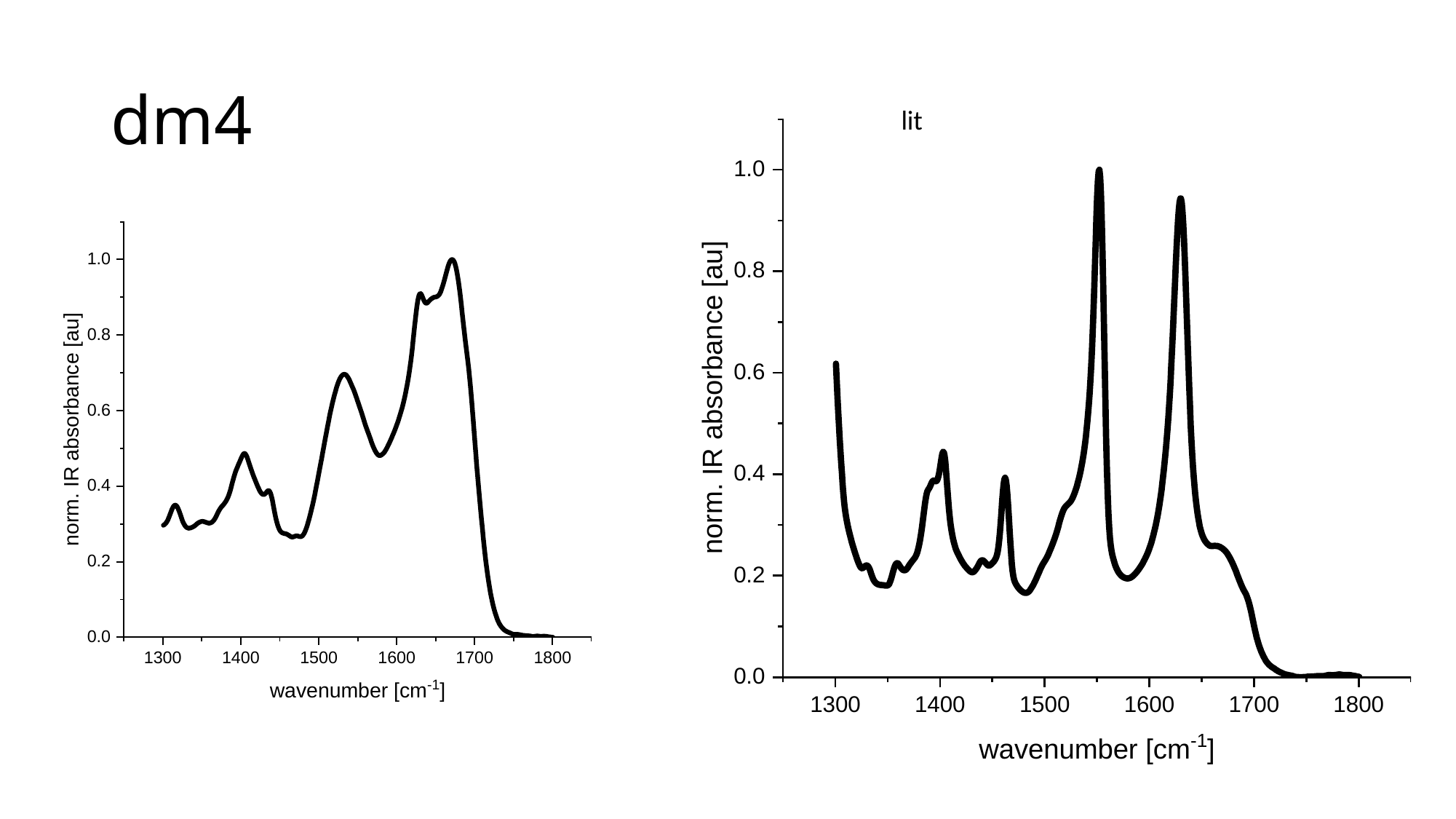

# dm4
lit

## Slide 171
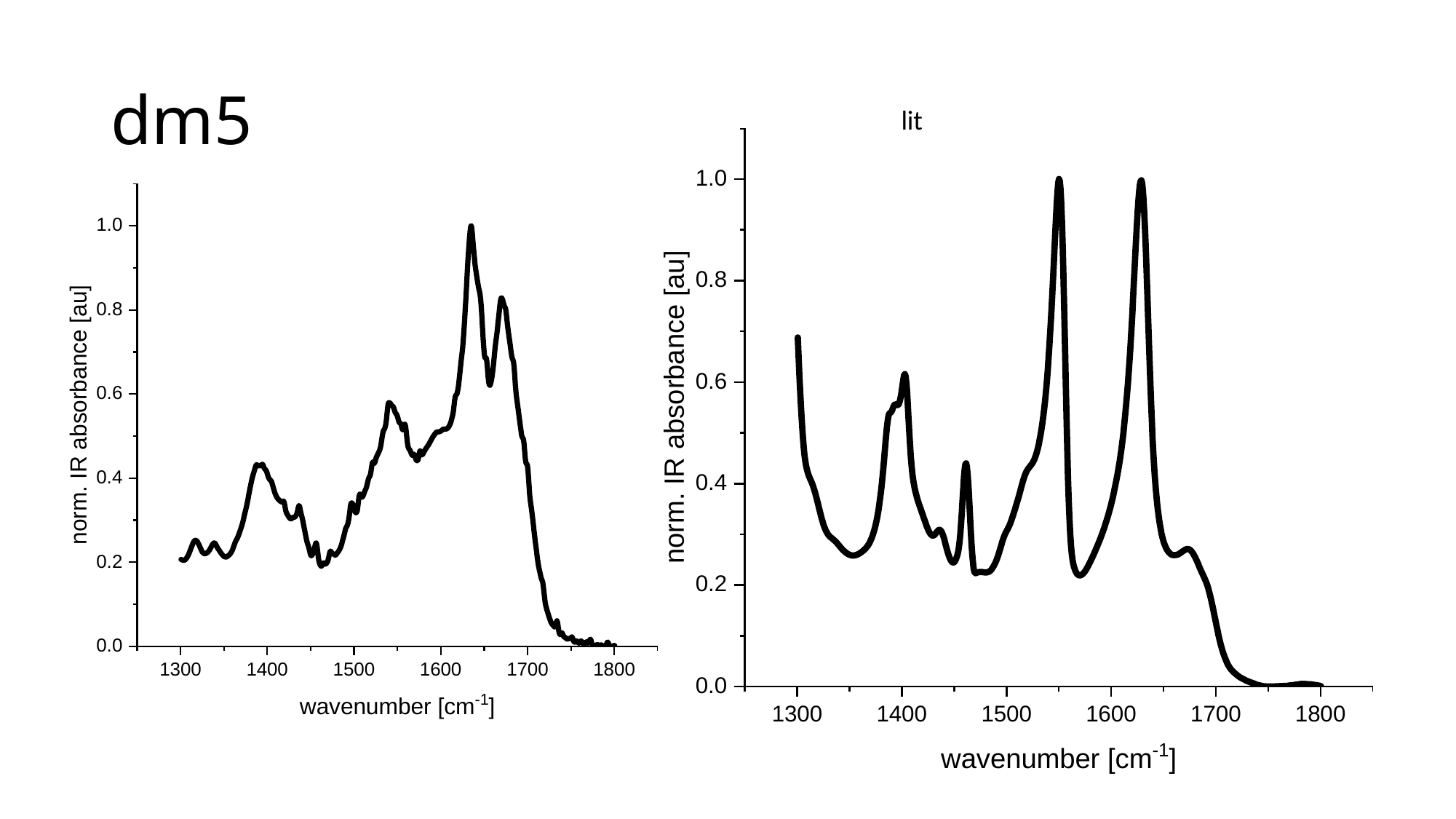

# dm5
lit

## Slide 172
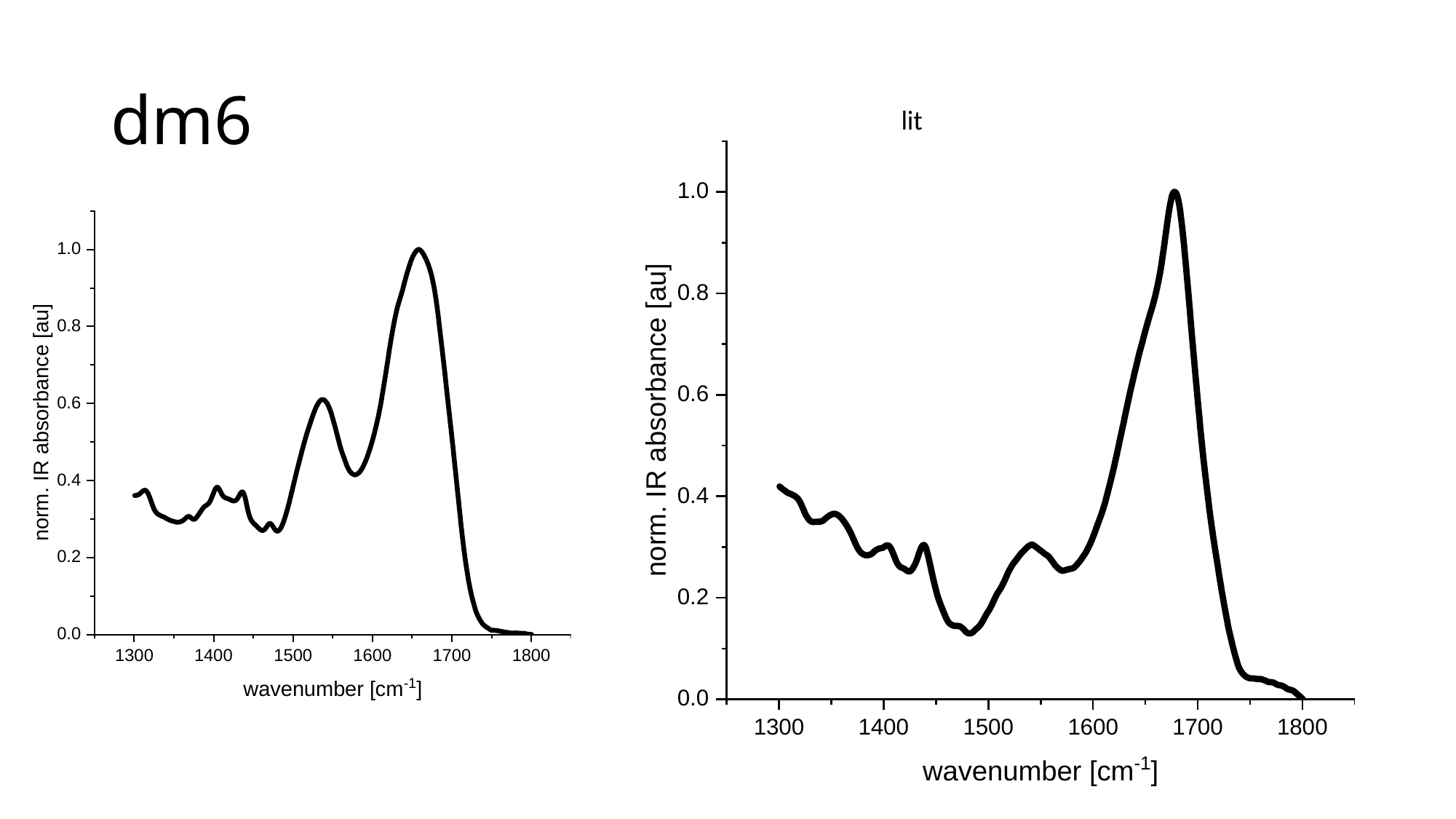

# dm6
lit

## Slide 173
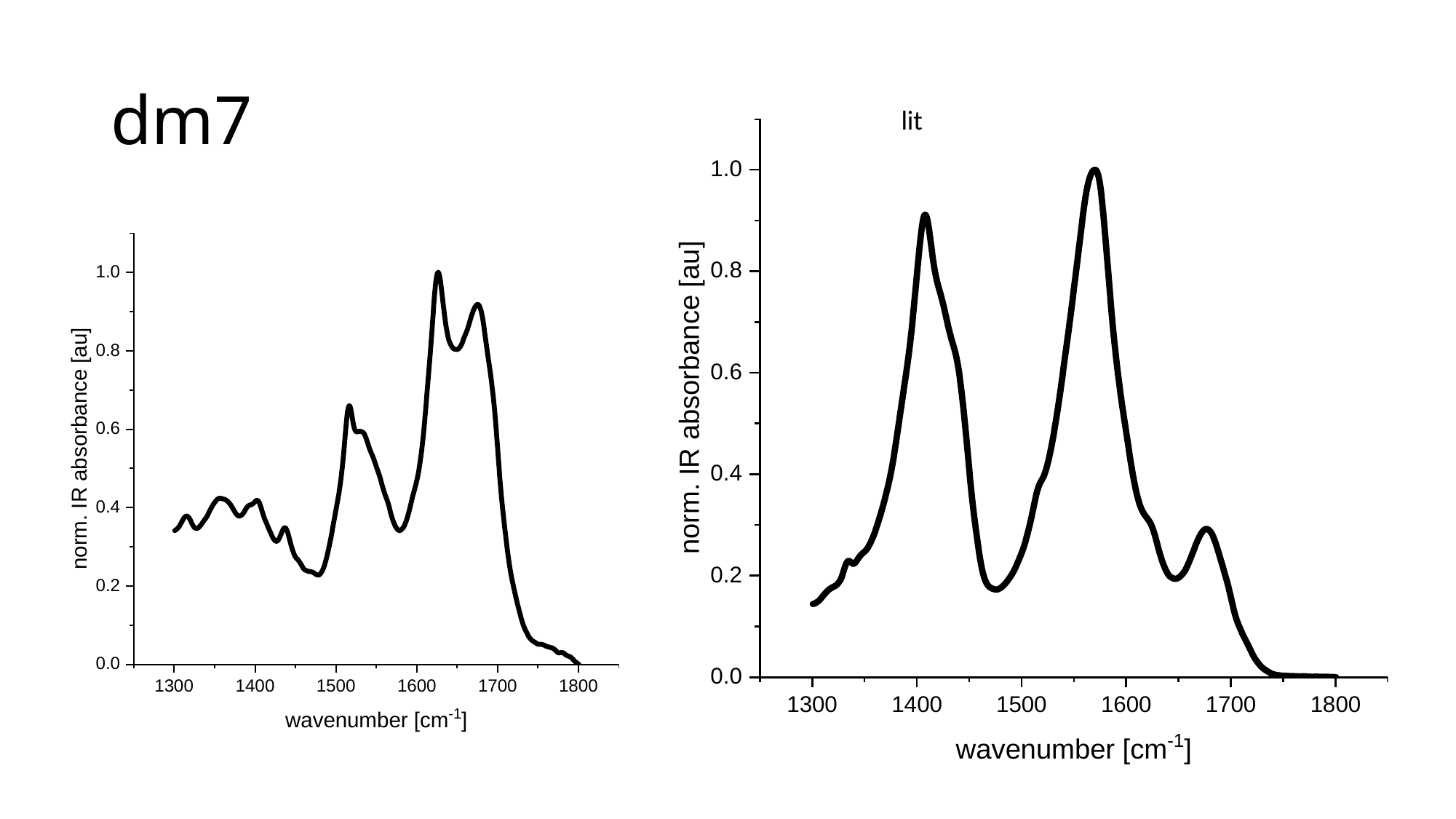

# dm7
lit

## Slide 174
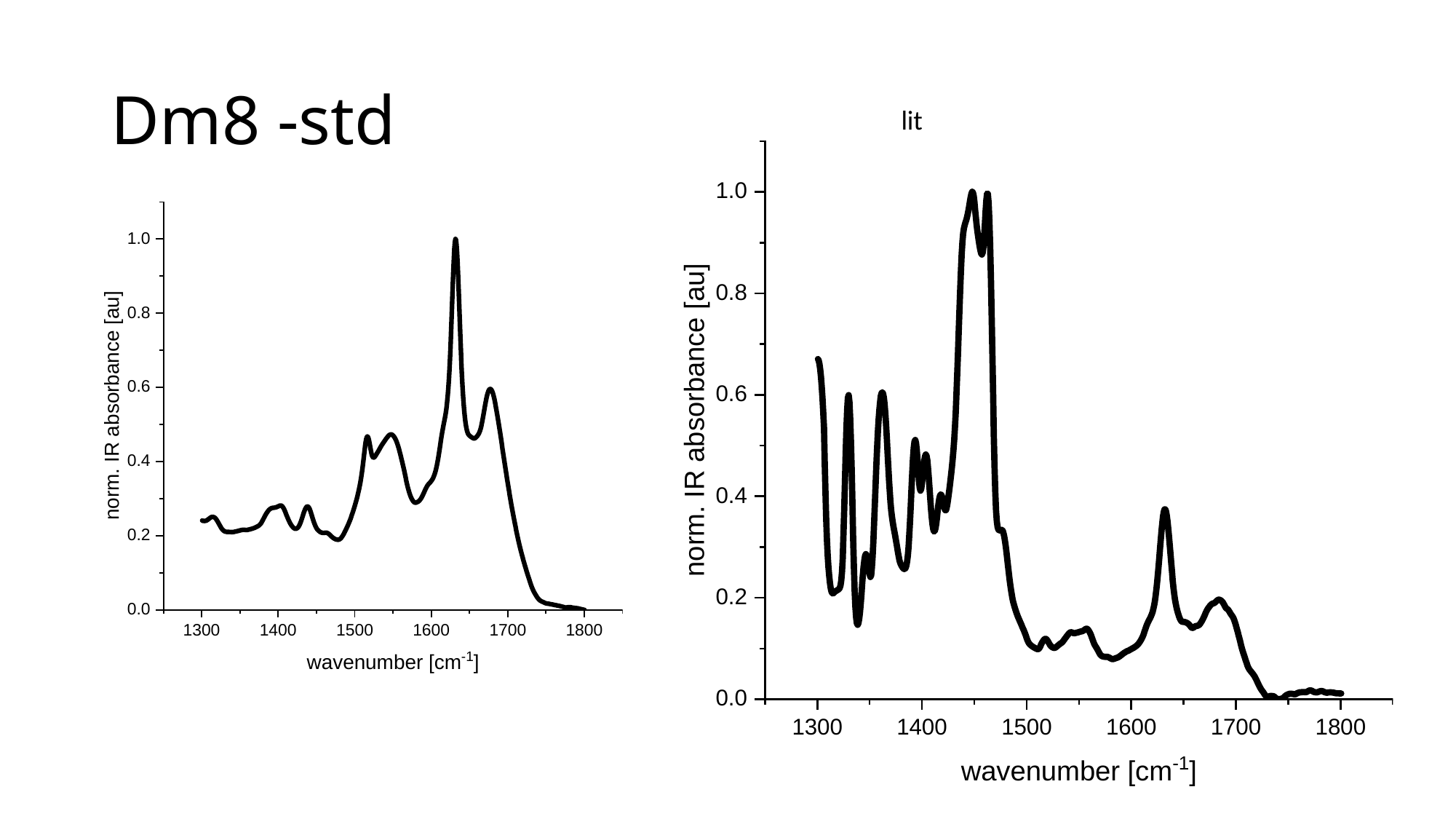

# Dm8 -std
lit

## Slide 175
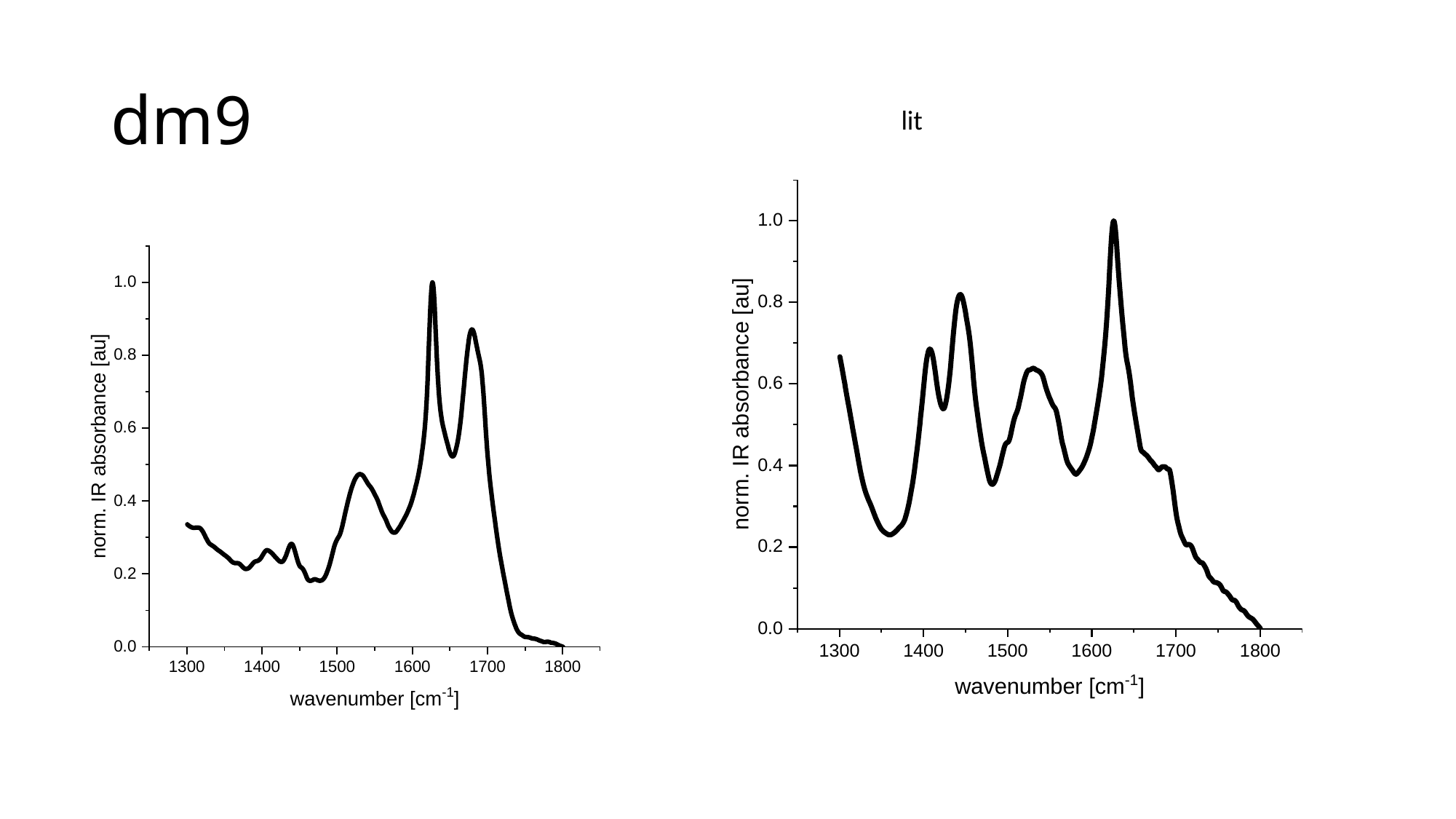

# dm9
lit

## Slide 176
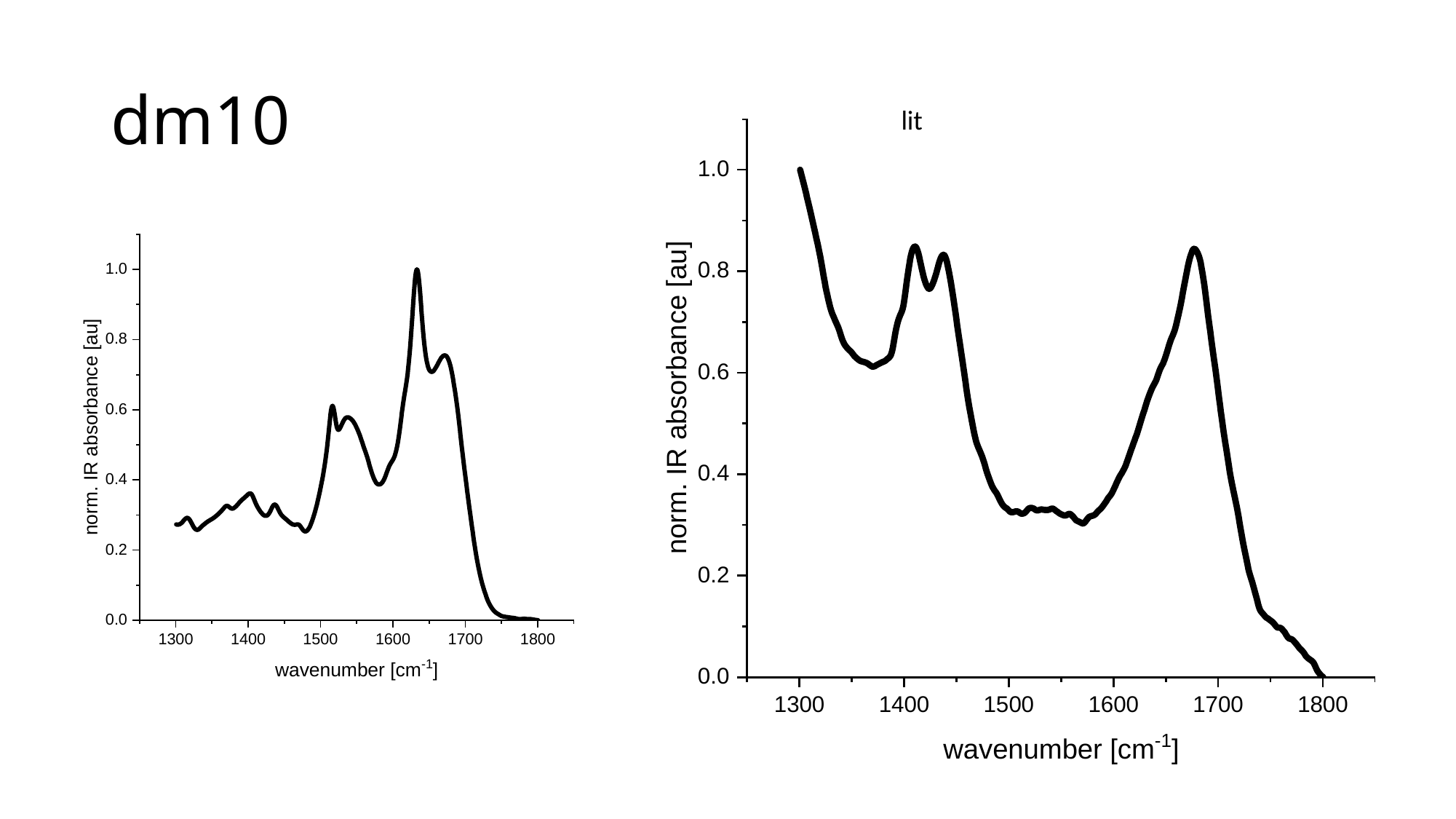

# dm10
lit

## Slide 177
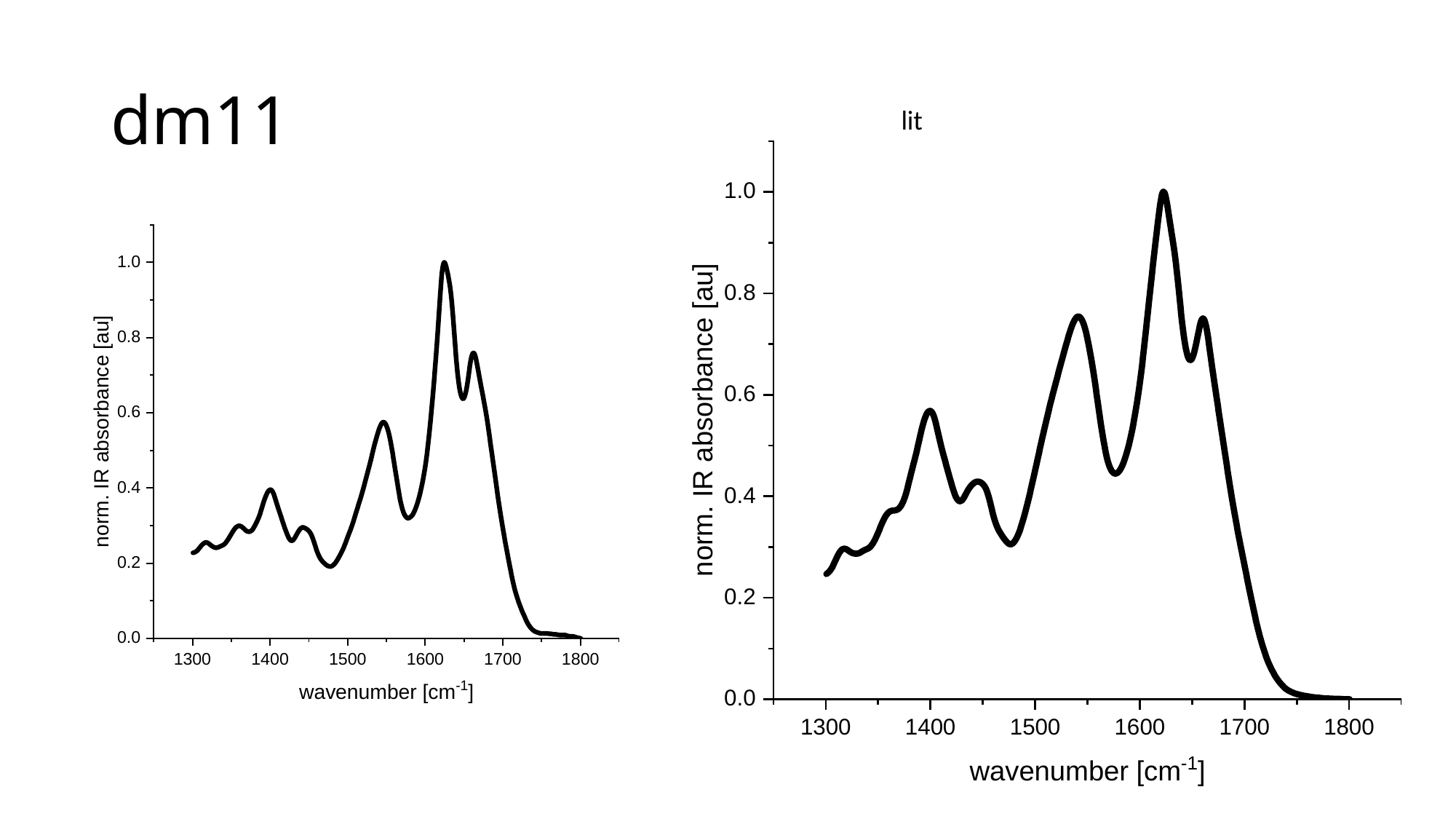

# dm11
lit

## Slide 178
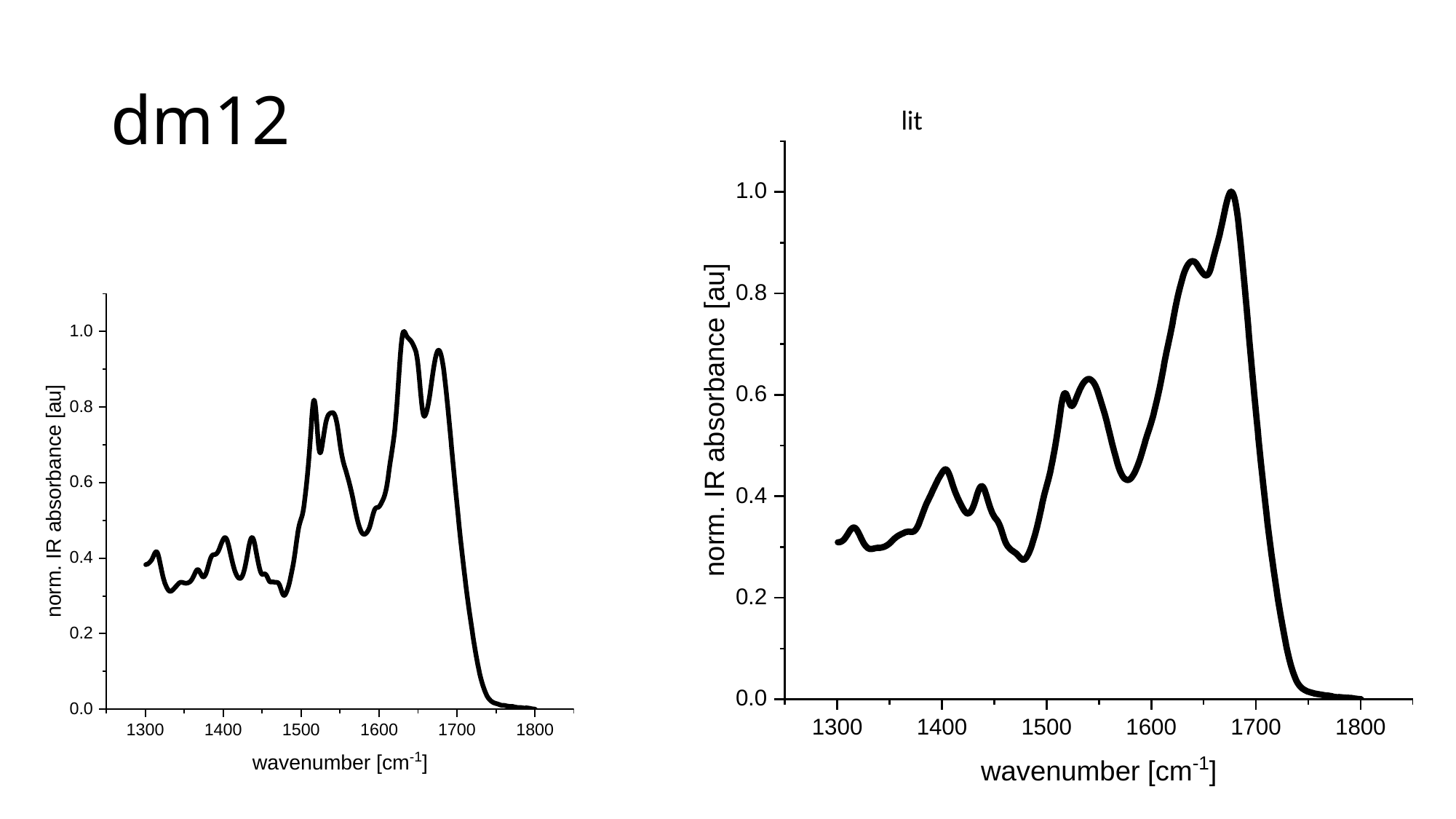

# dm12
lit

## Slide 179
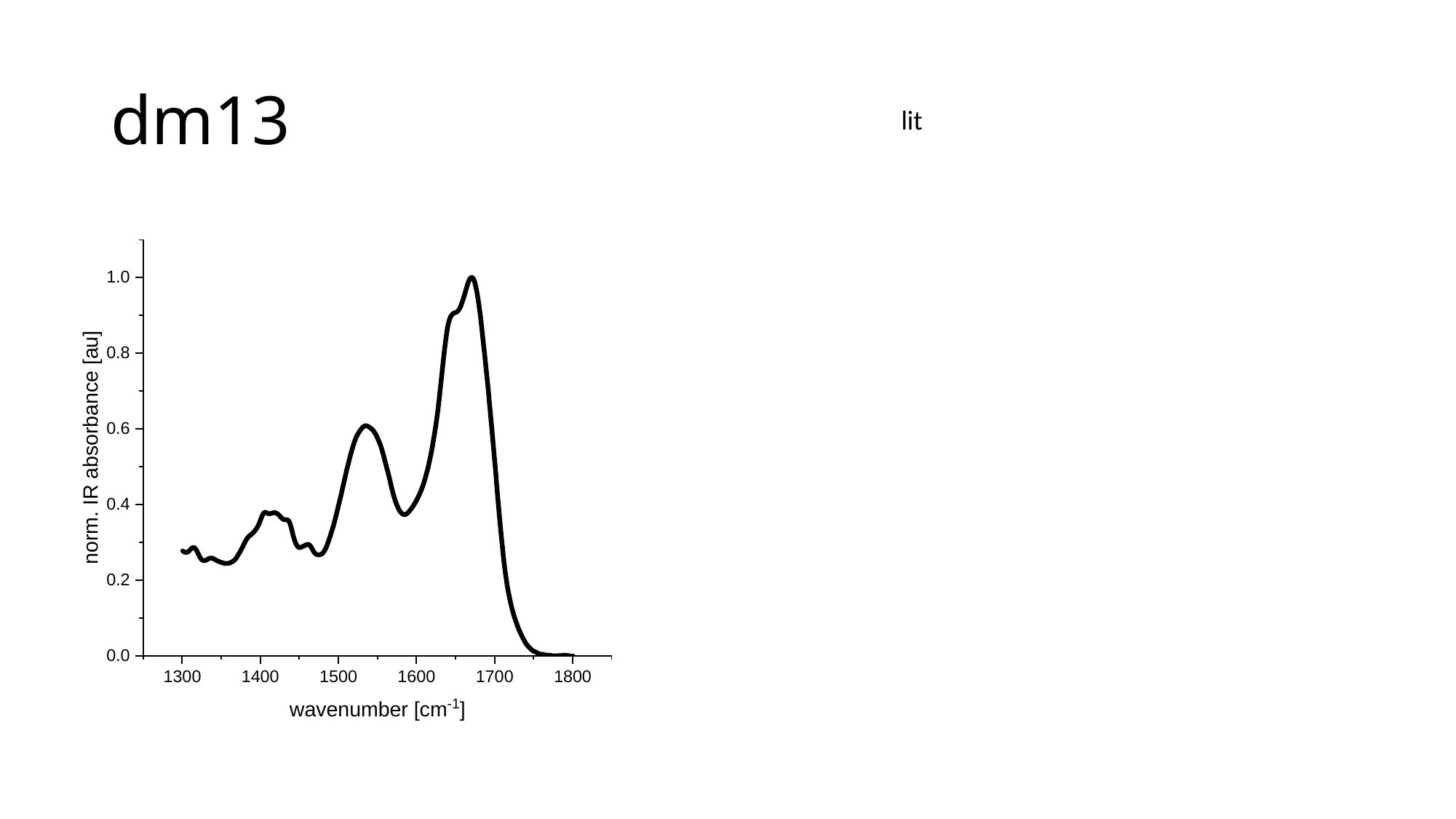

# dm13
lit

## Slide 180
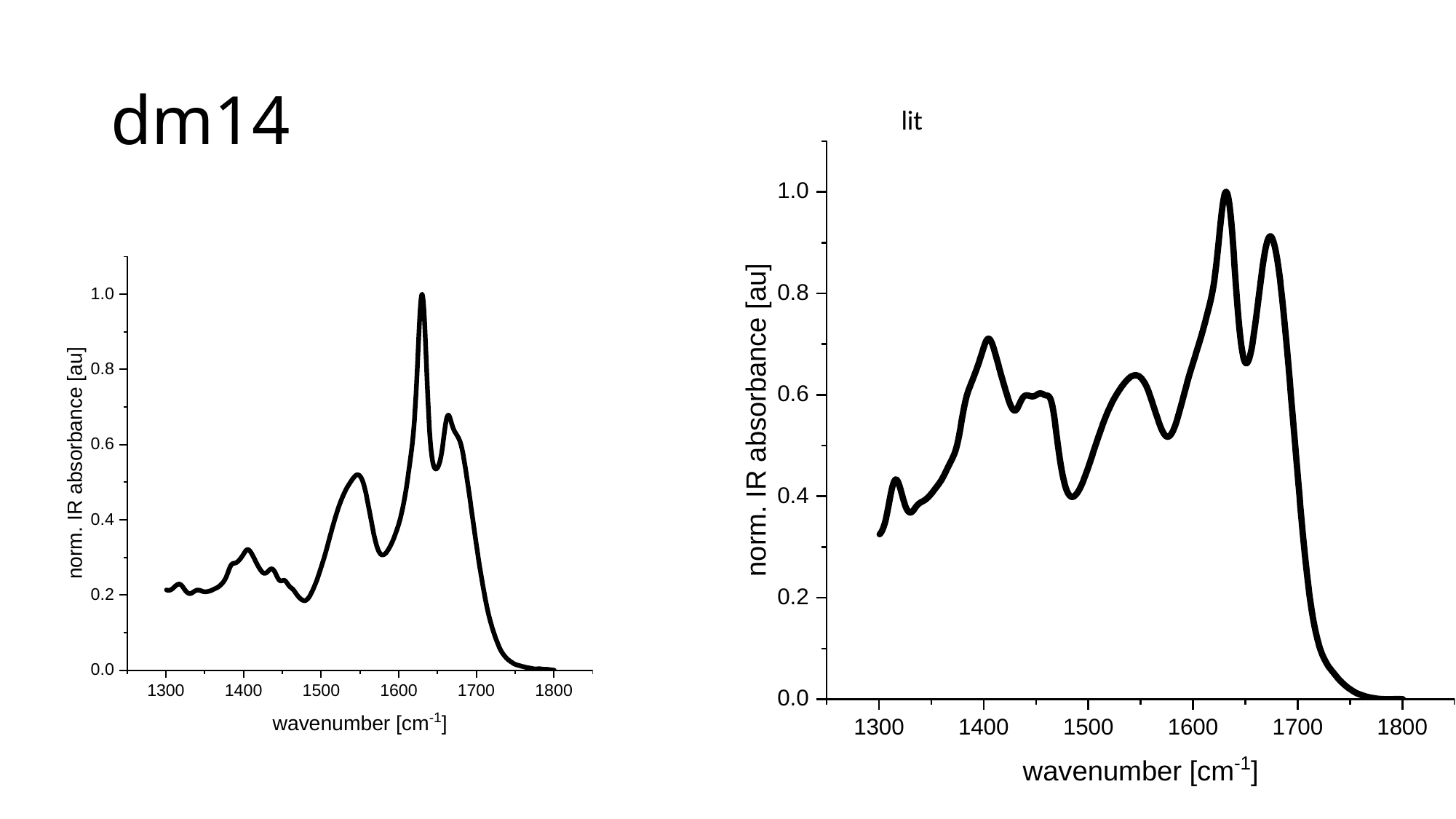

# dm14
lit

## Slide 181
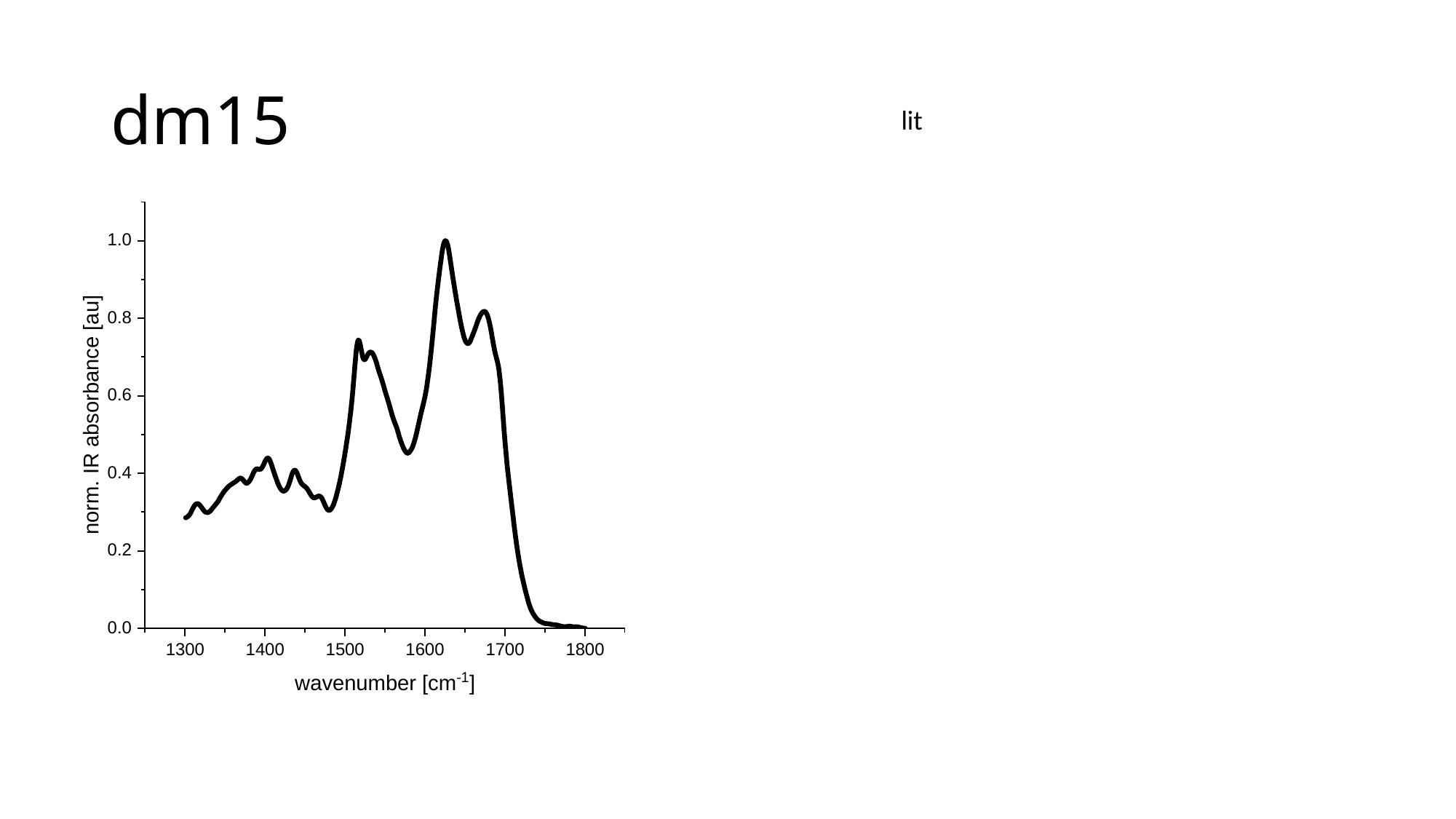

# dm15
lit

## Slide 182
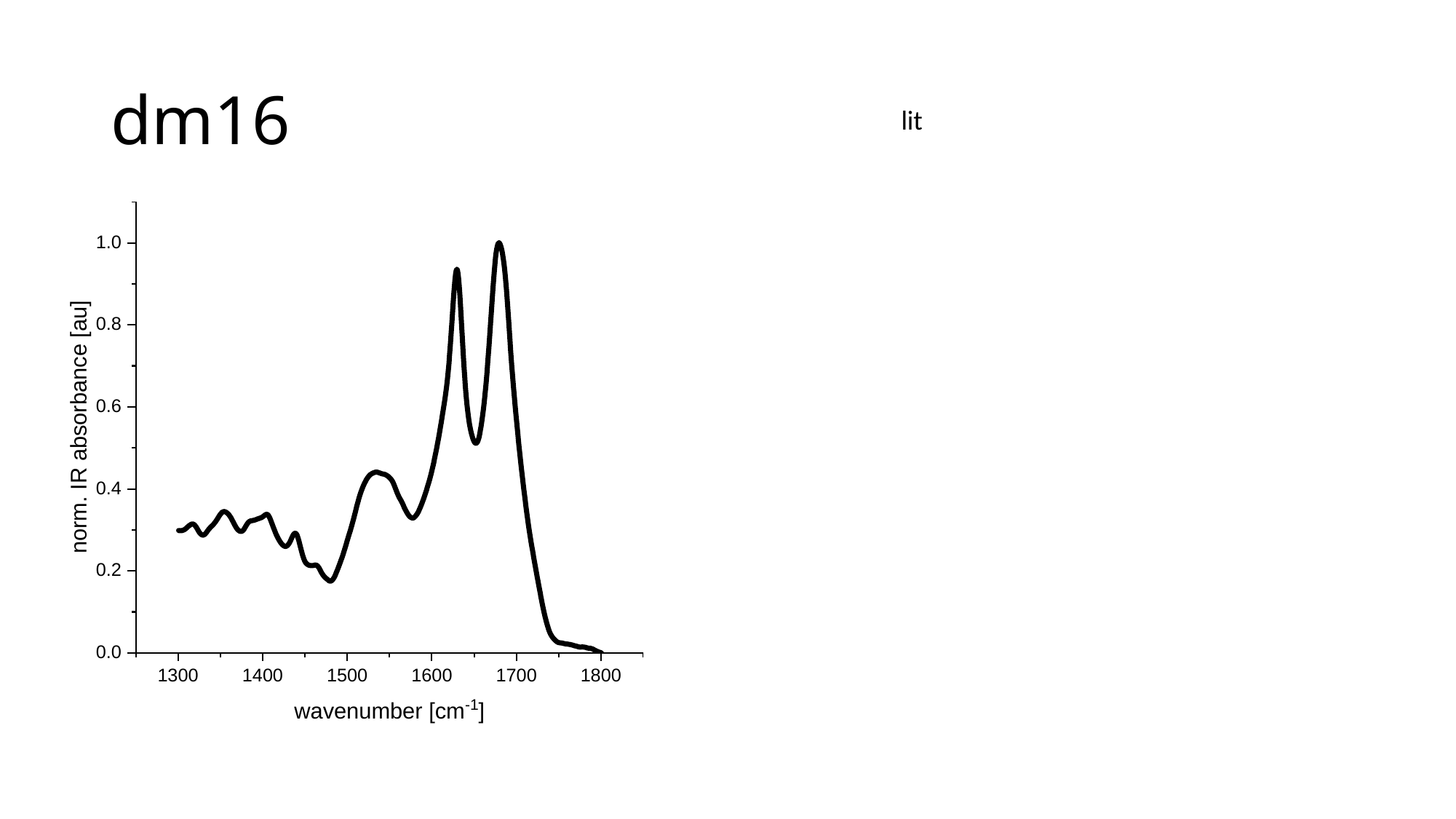

# dm16
lit

## Slide 183
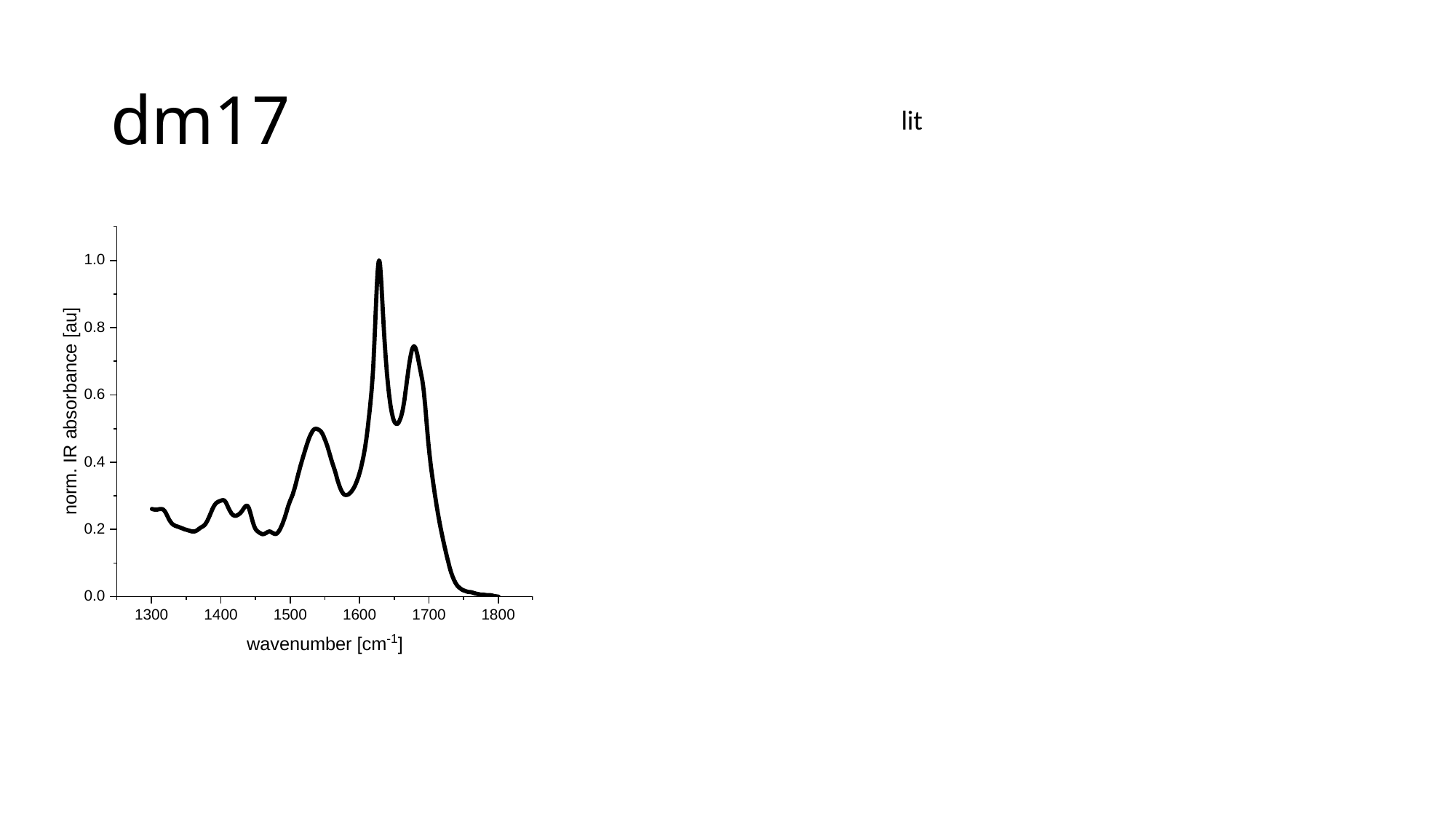

# dm17
lit

## Slide 184
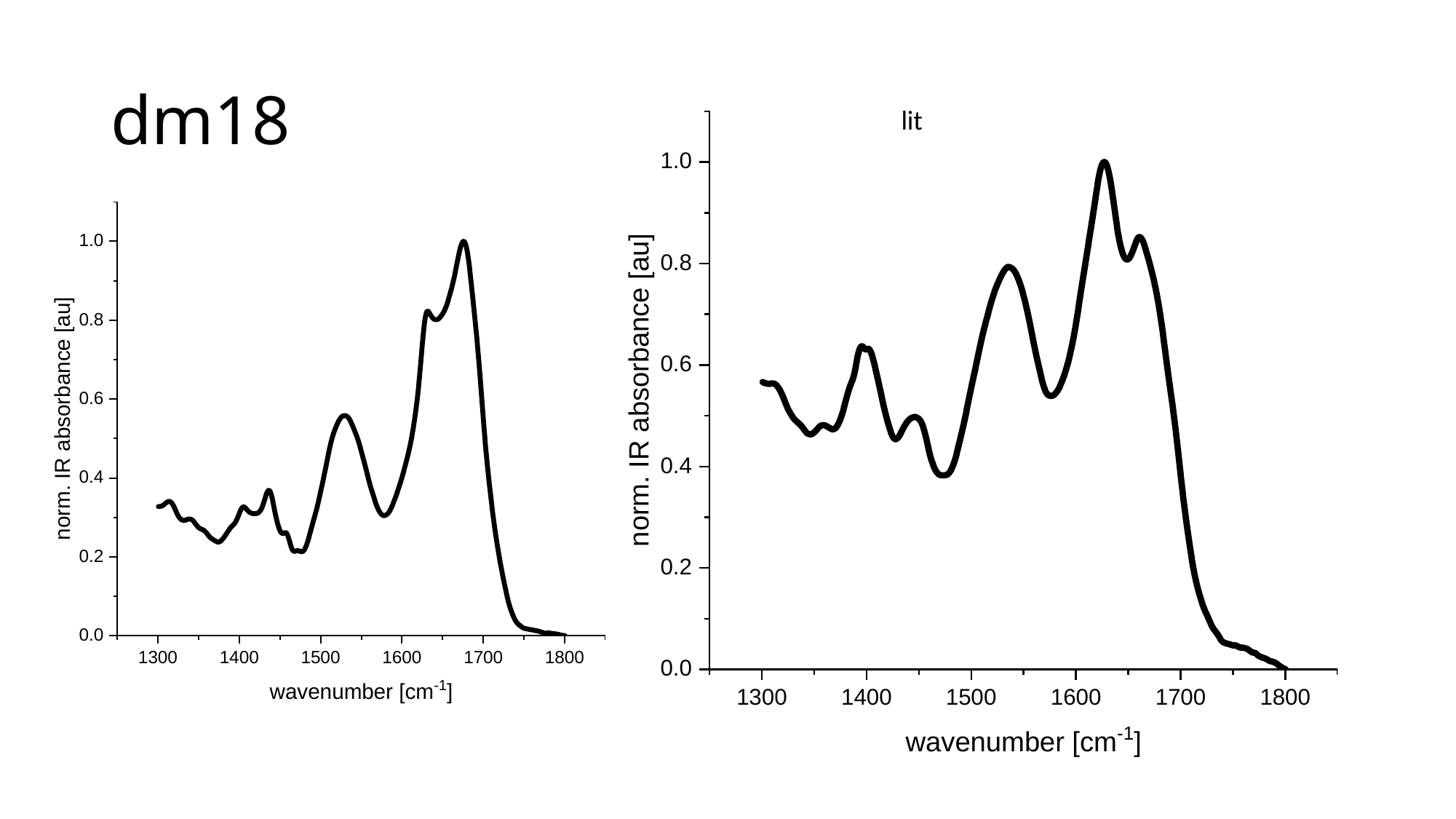

# dm18
lit

## Slide 185
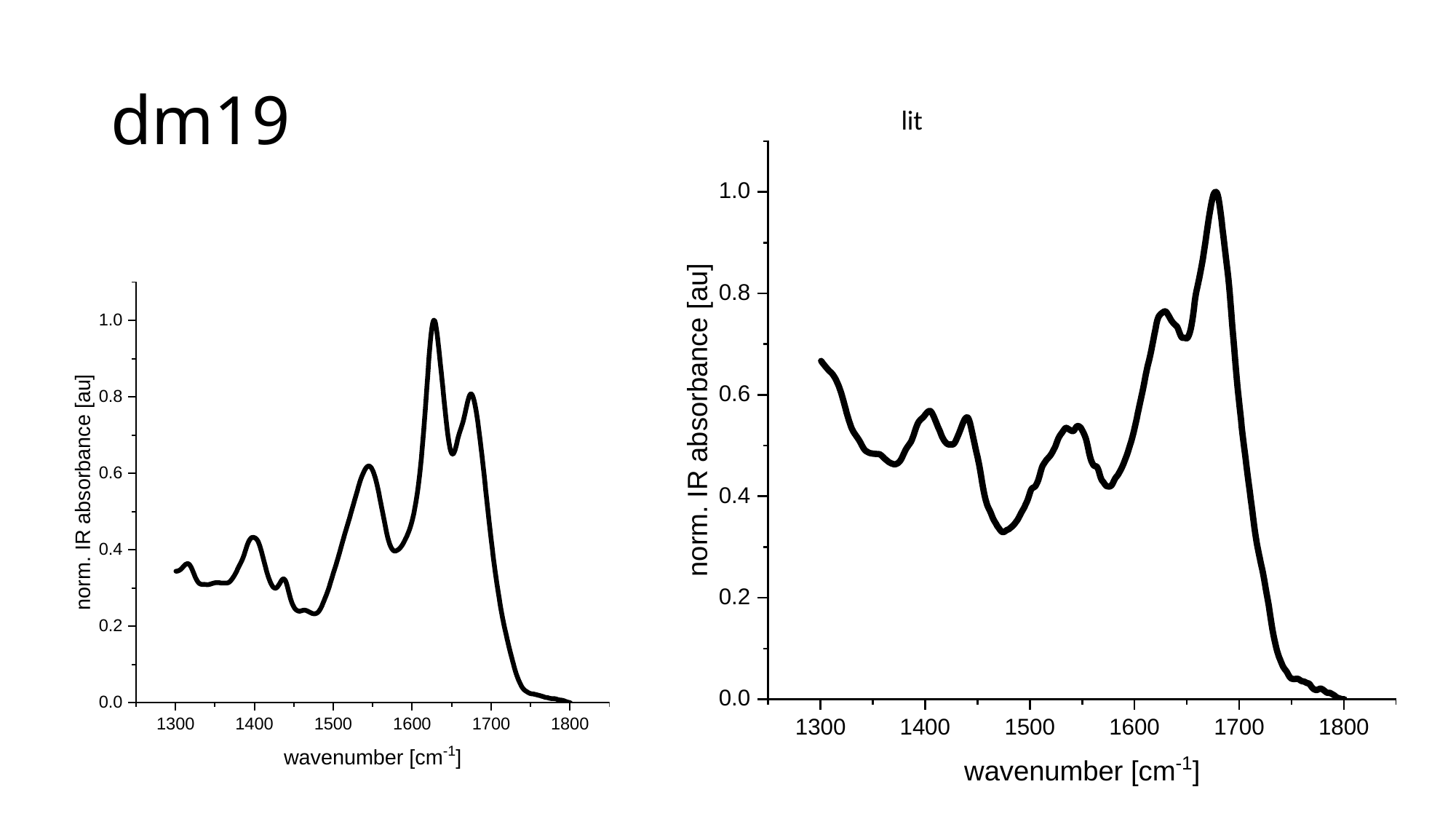

# dm19
lit

## Slide 186
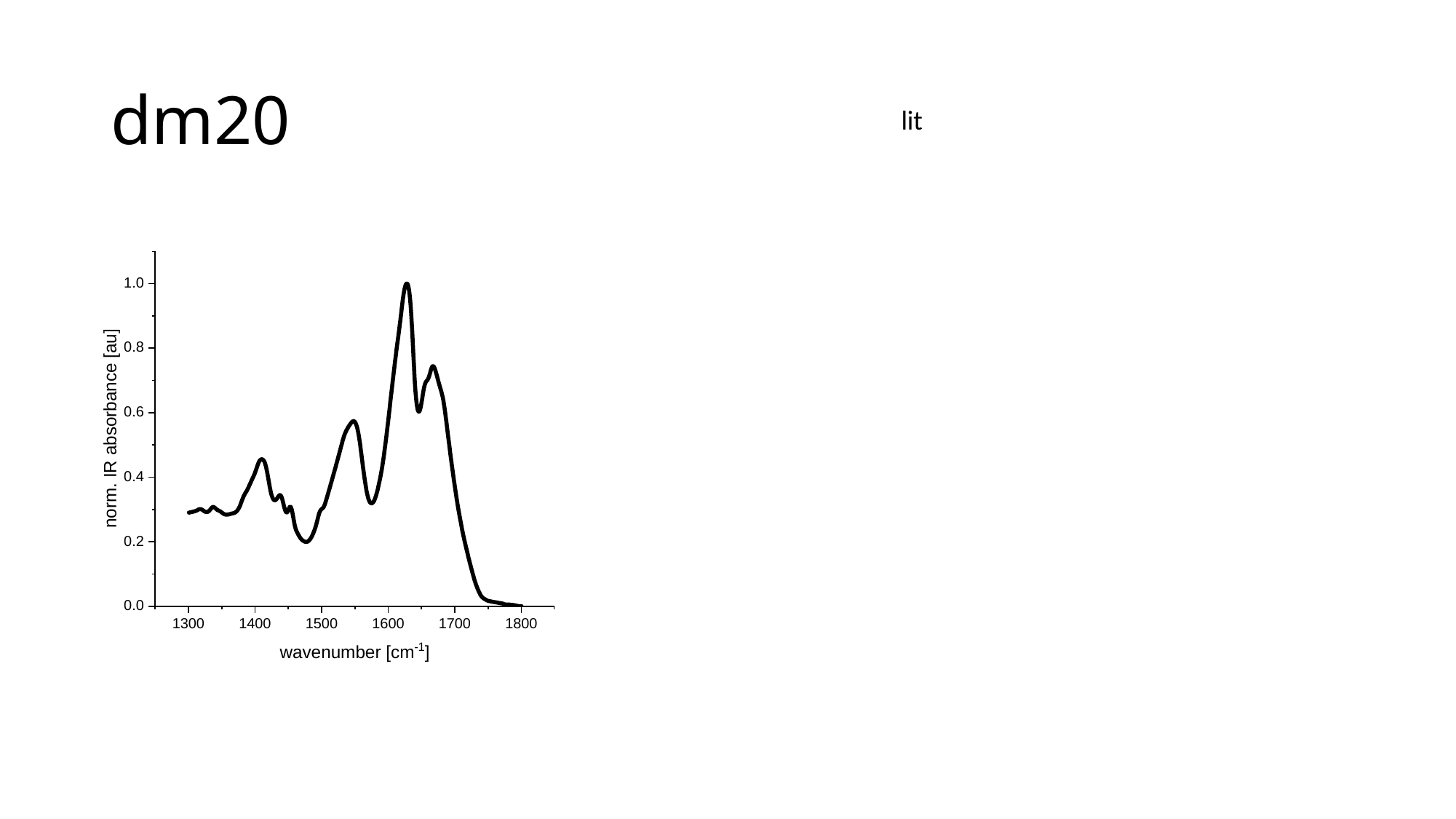

# dm20
lit

## Slide 187
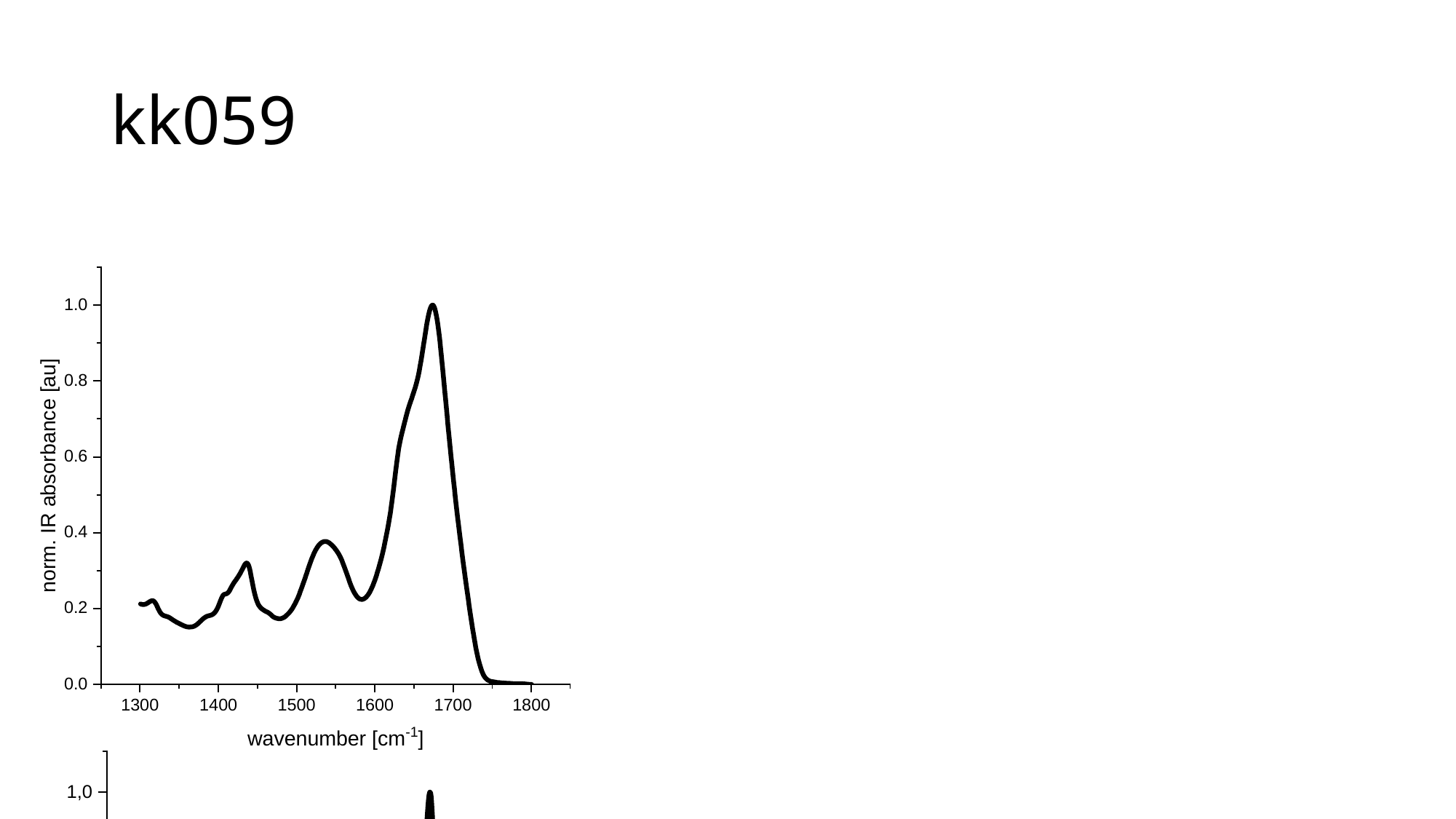

# kk059

## Slide 188
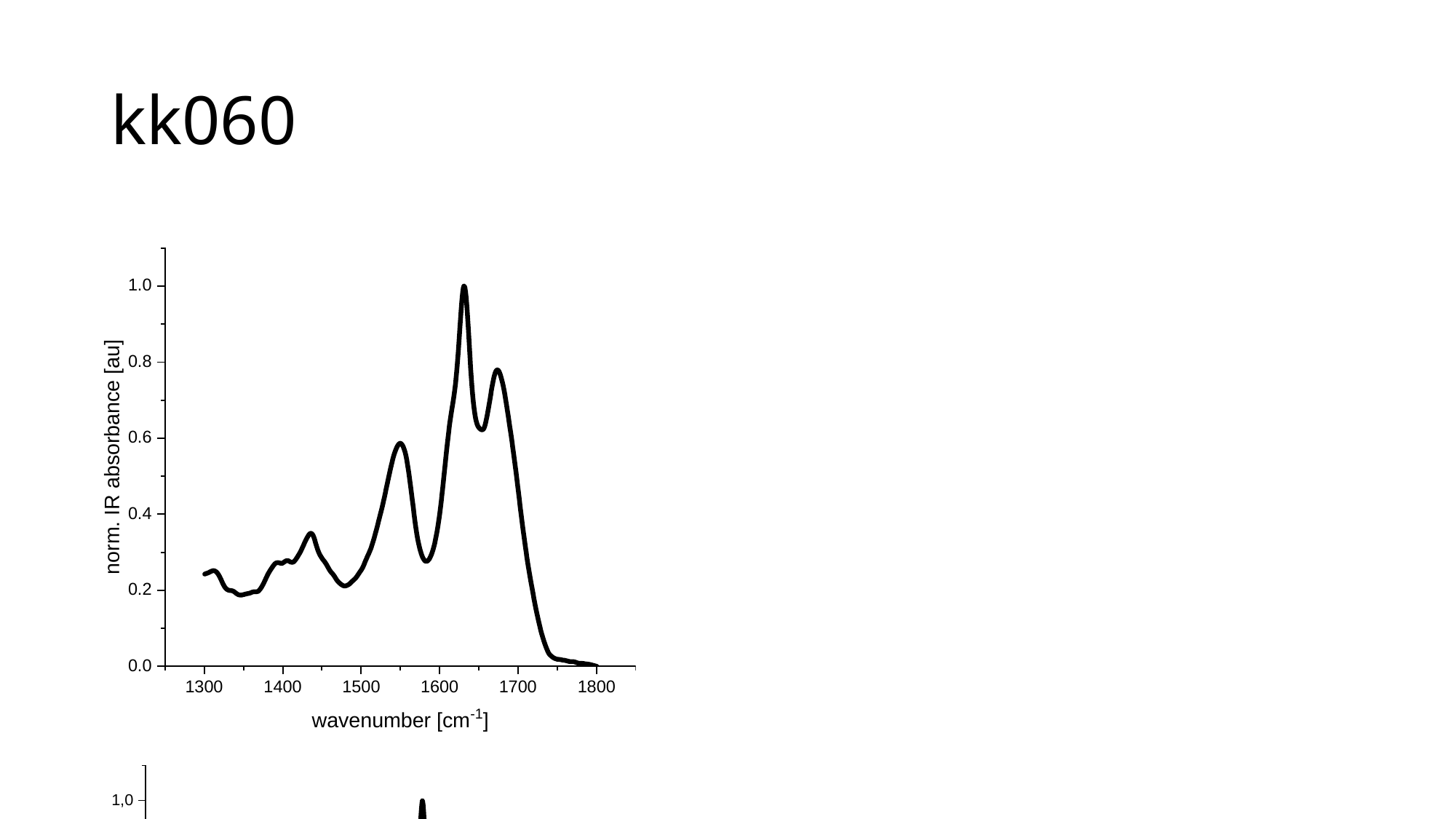

# kk060

## Slide 189
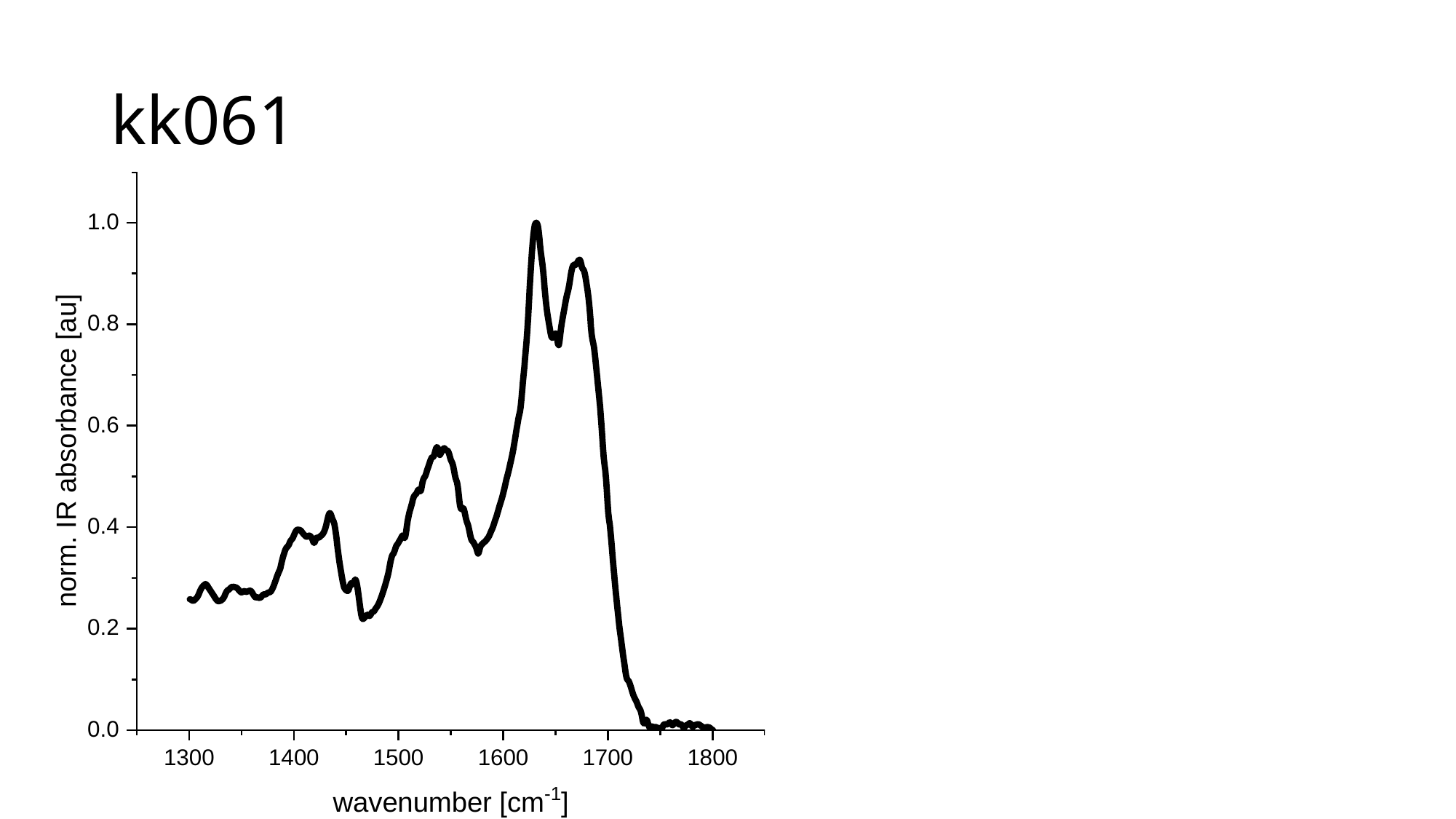

# kk061

## Slide 190
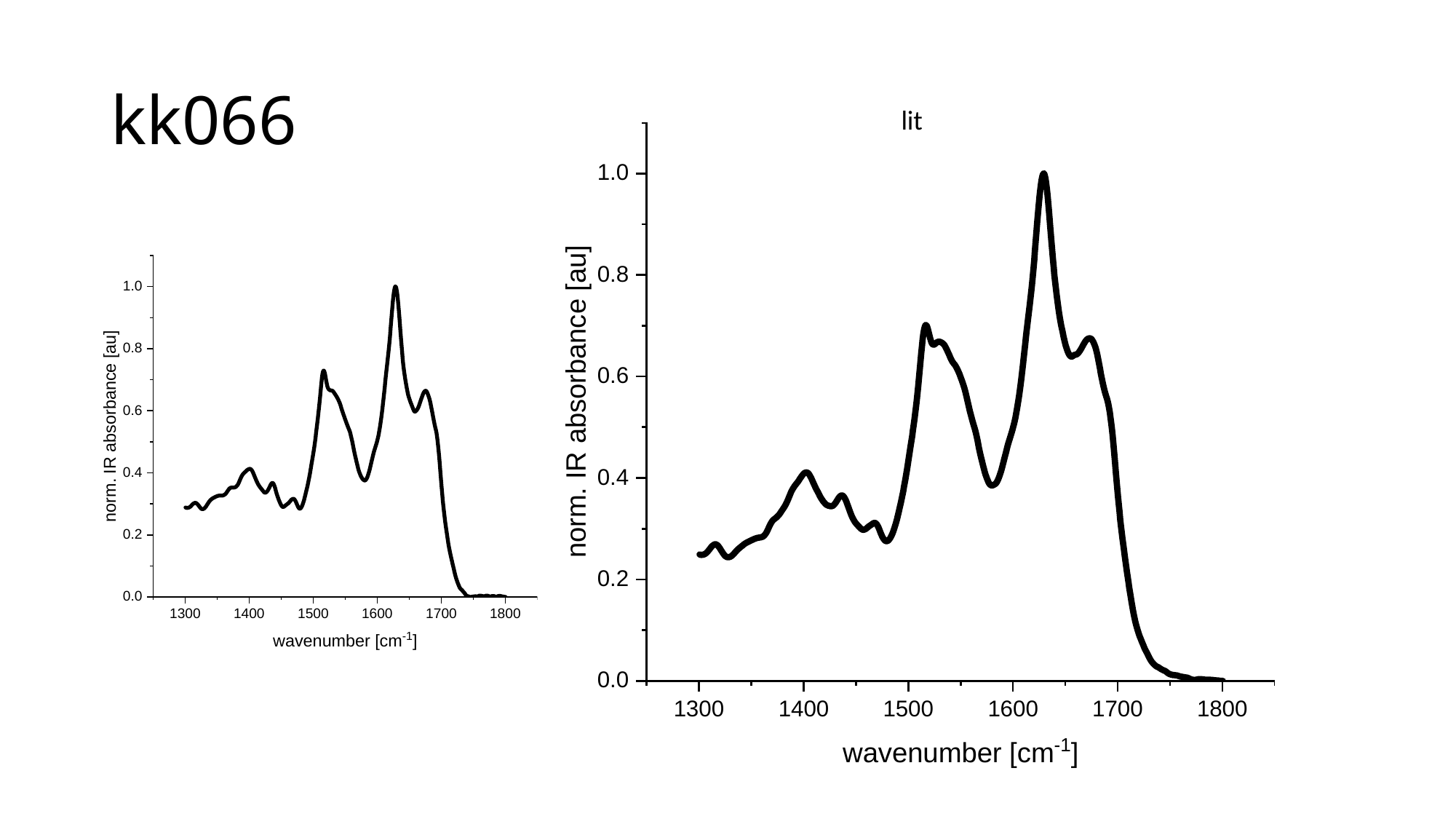

# kk066
lit

## Slide 191
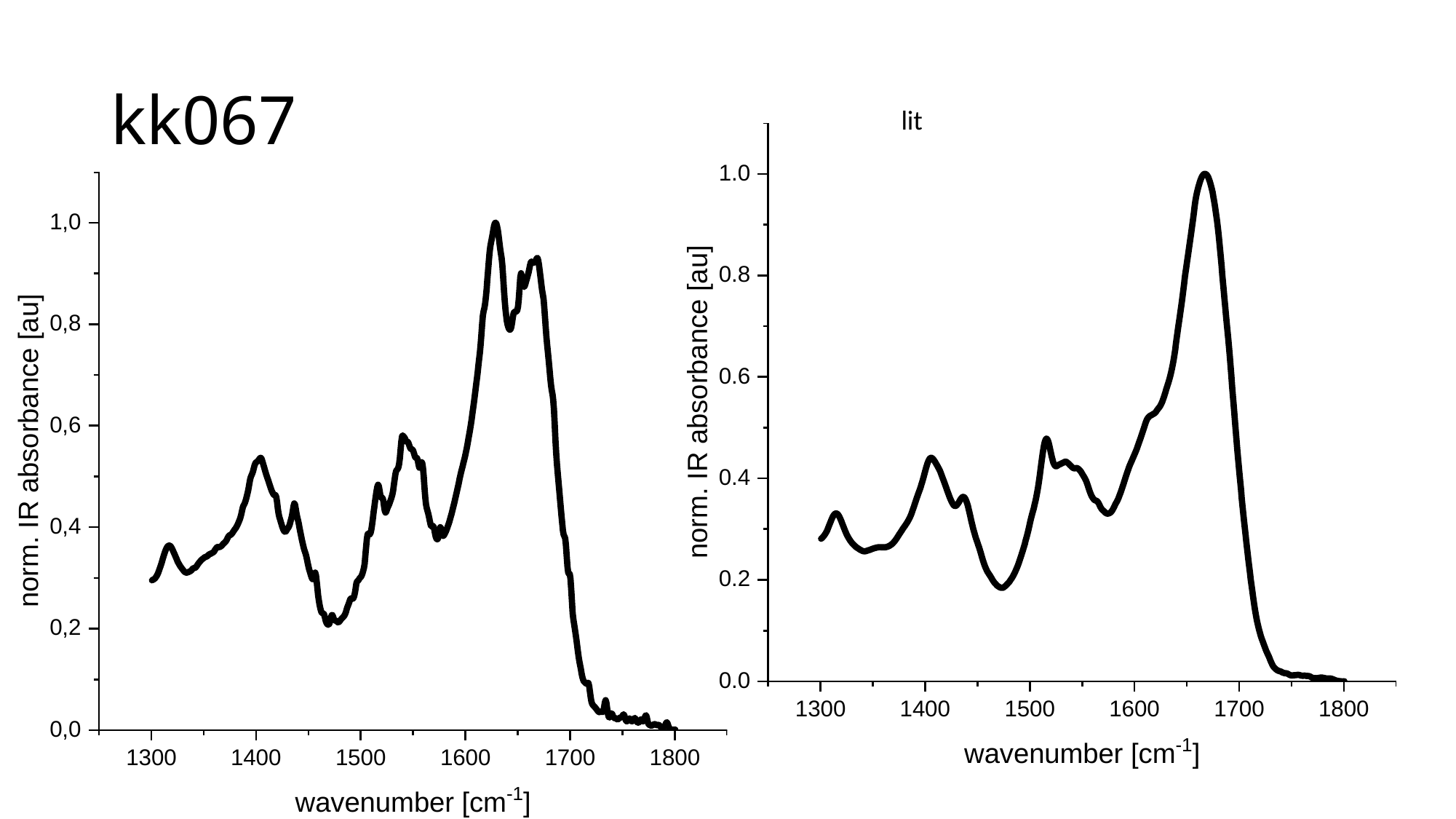

# kk067
lit

## Slide 192
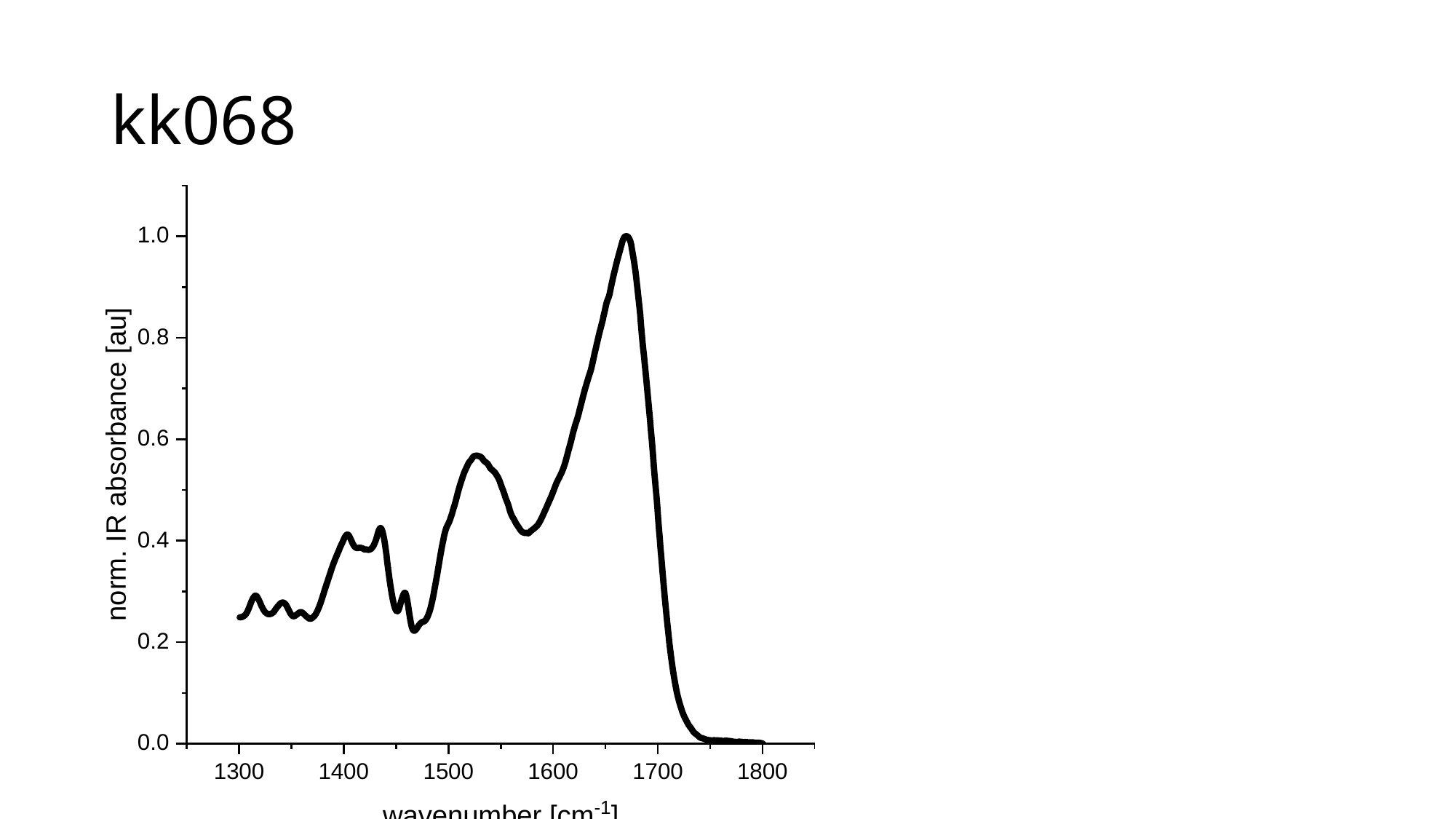

# kk068

## Slide 193
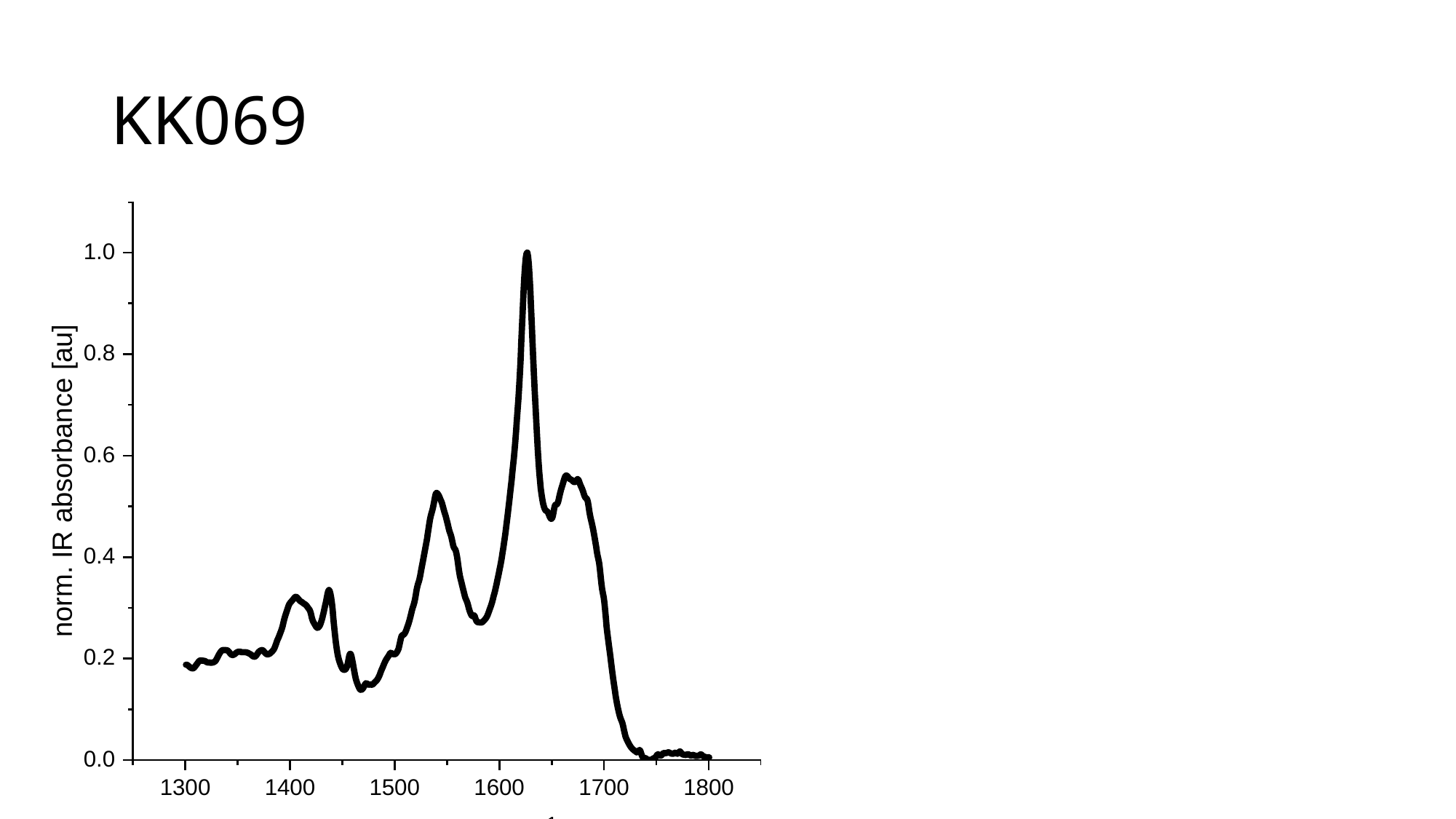

# KK069

## Slide 194
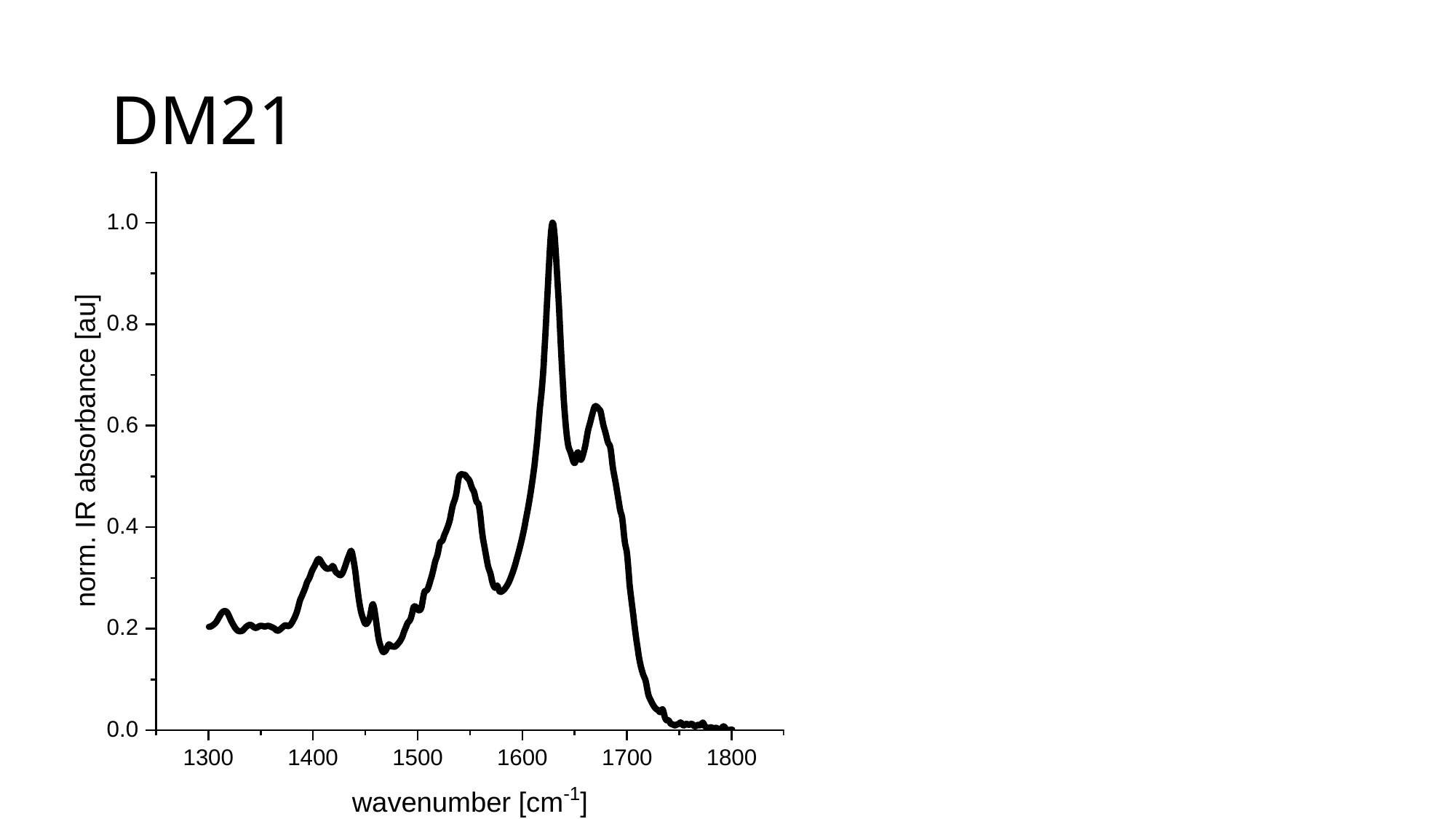

# DM21

## Slide 195
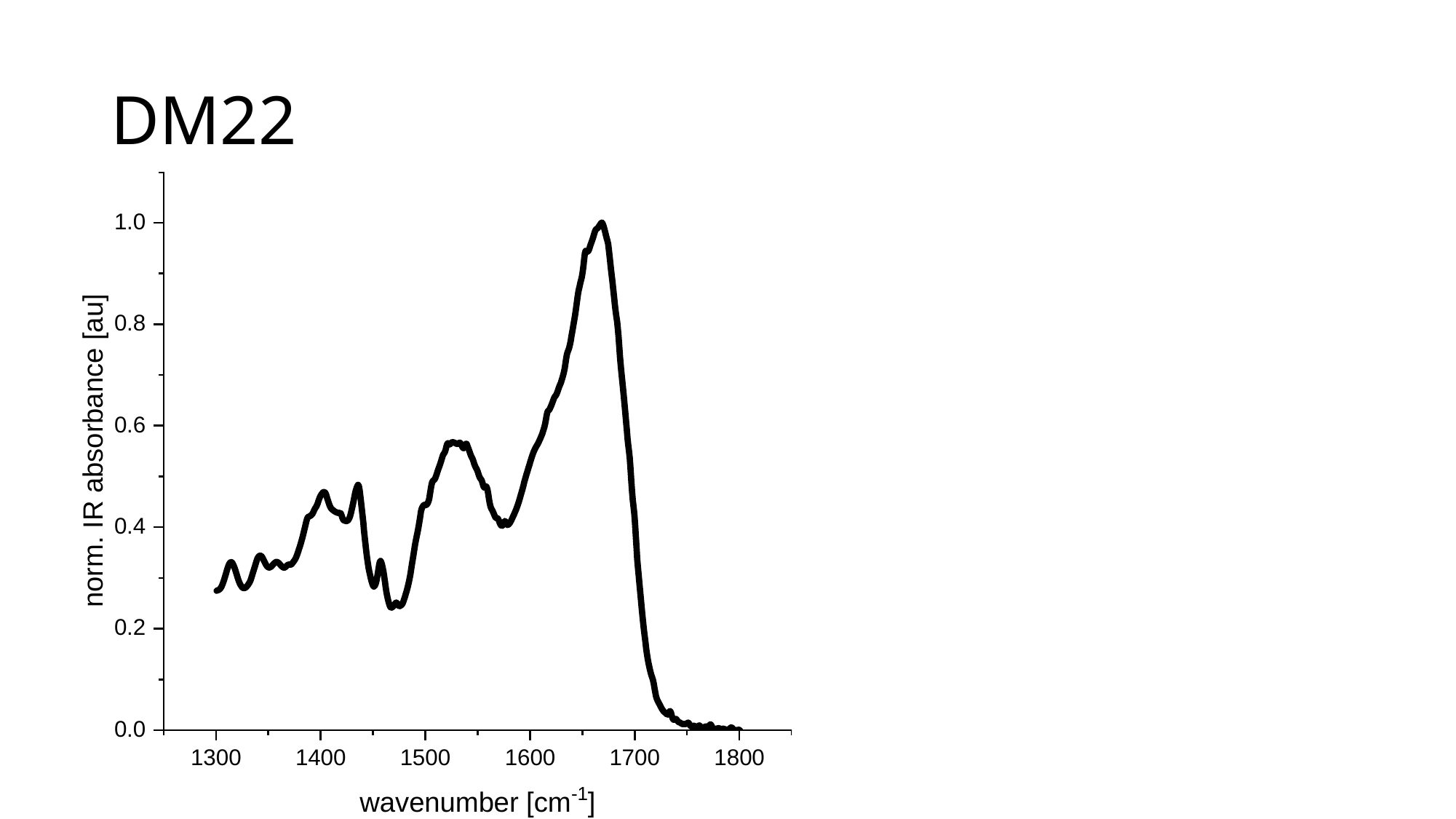

# DM22

## Slide 196
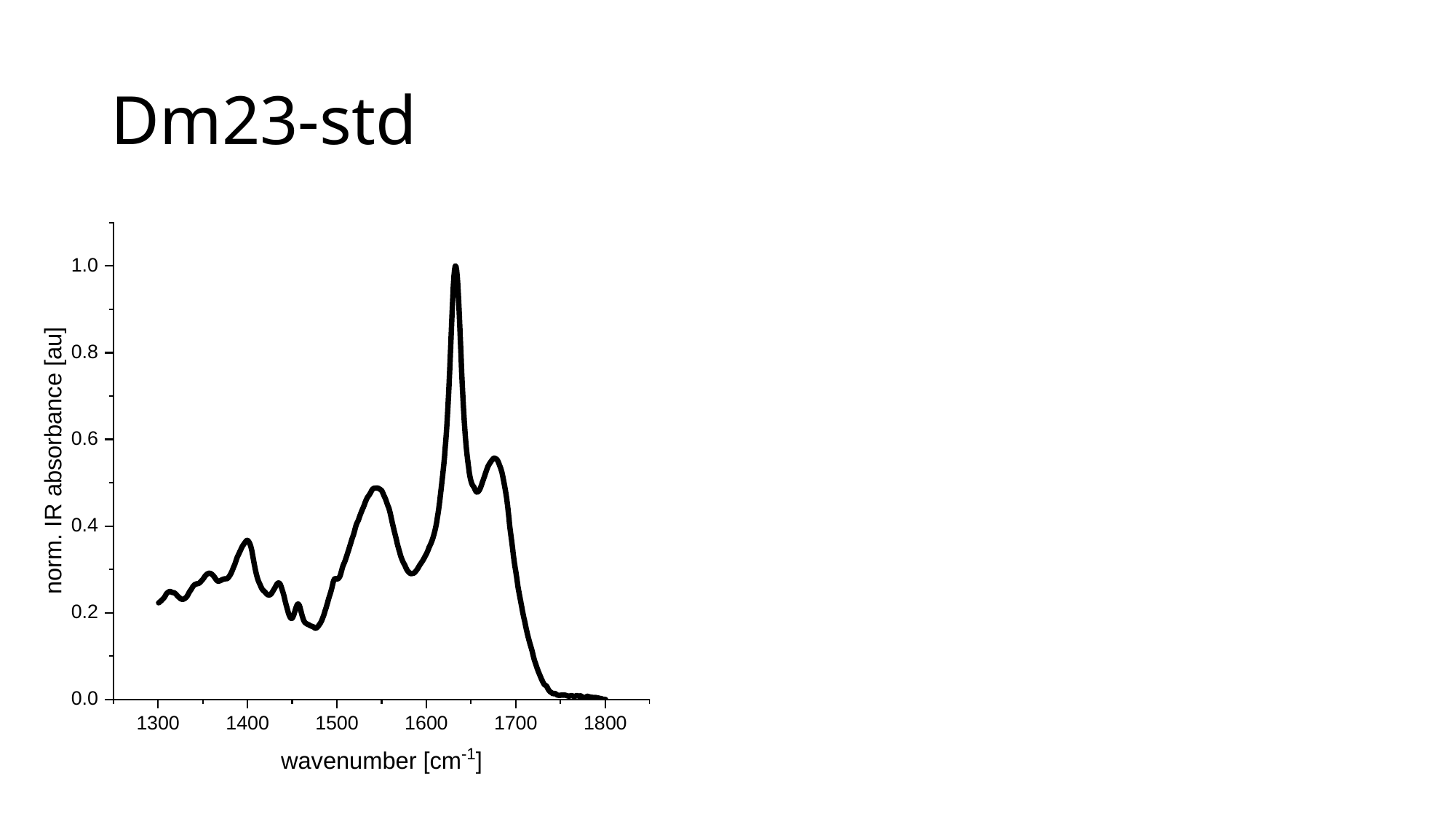

# Dm23-std

## Slide 197
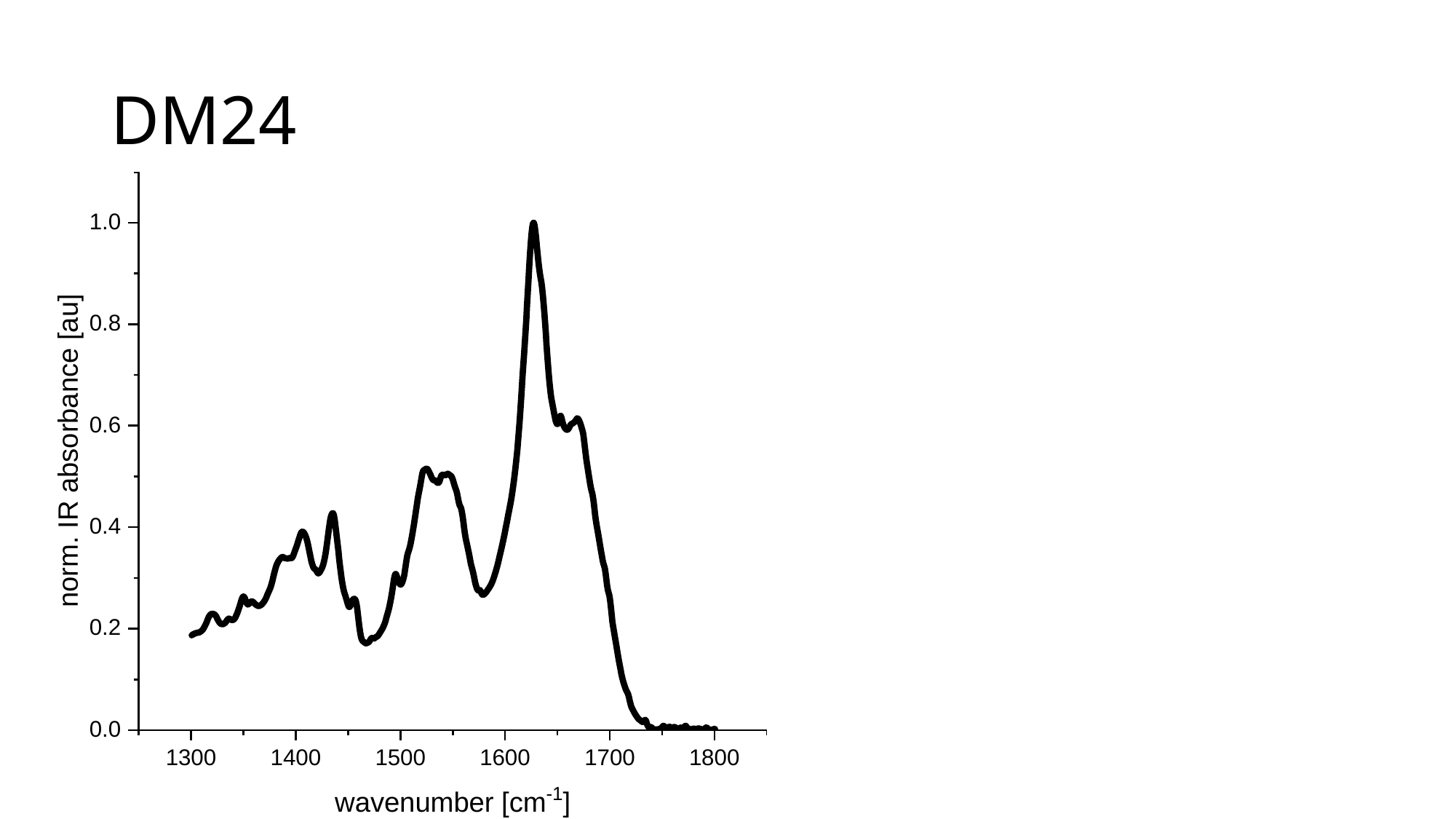

# DM24

## Slide 198
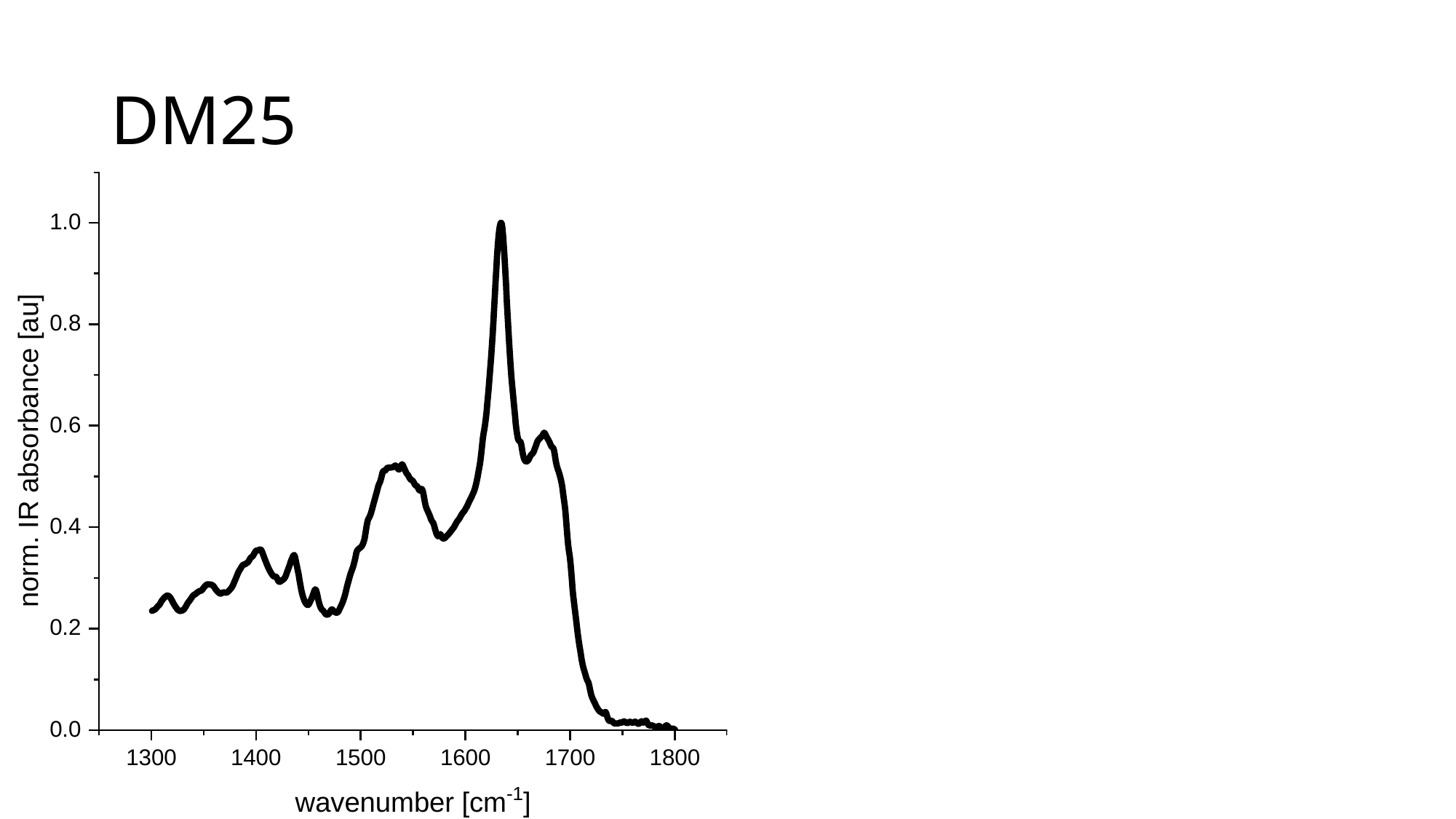

# DM25

## Slide 199
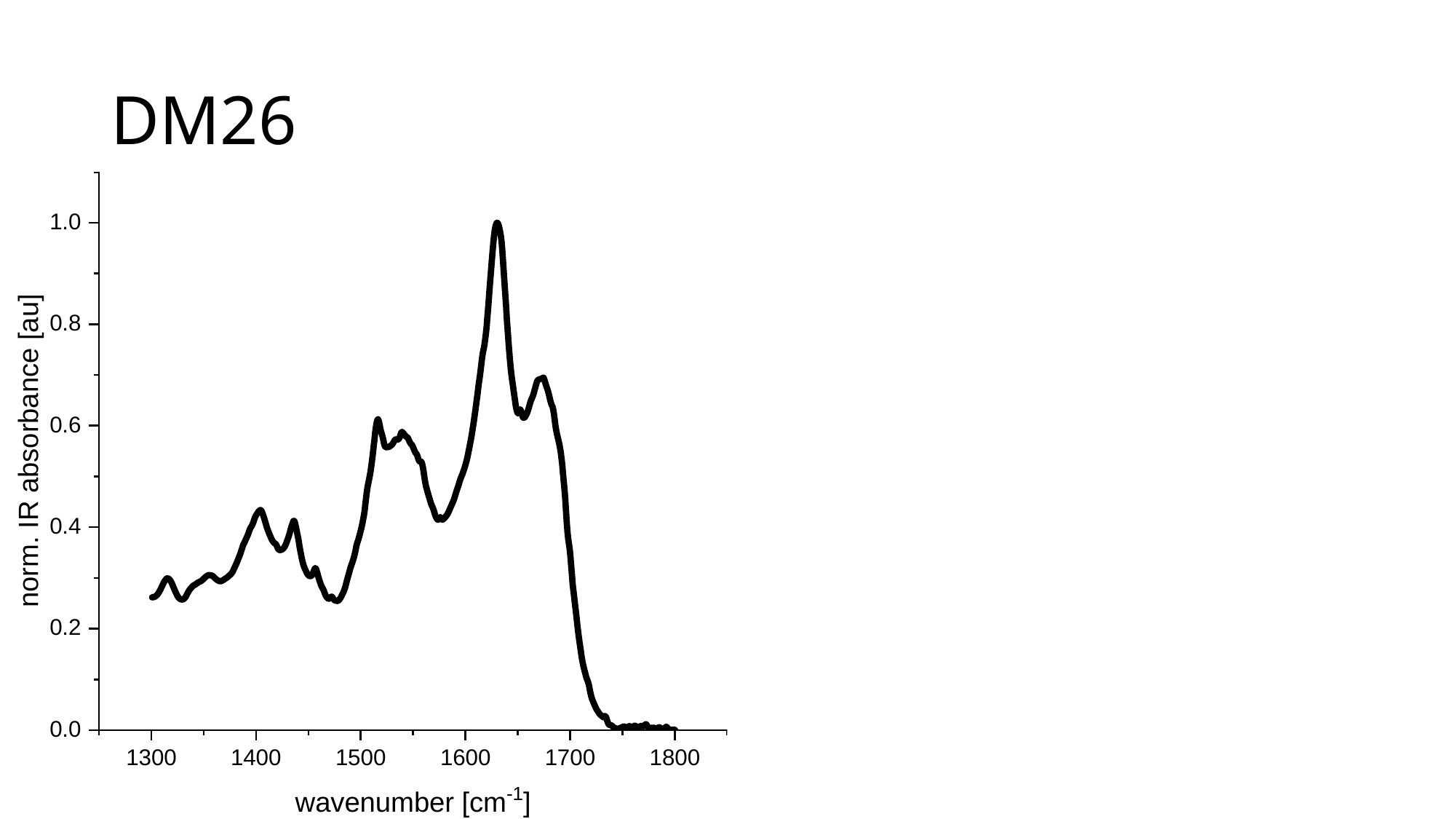

# DM26

## Slide 200
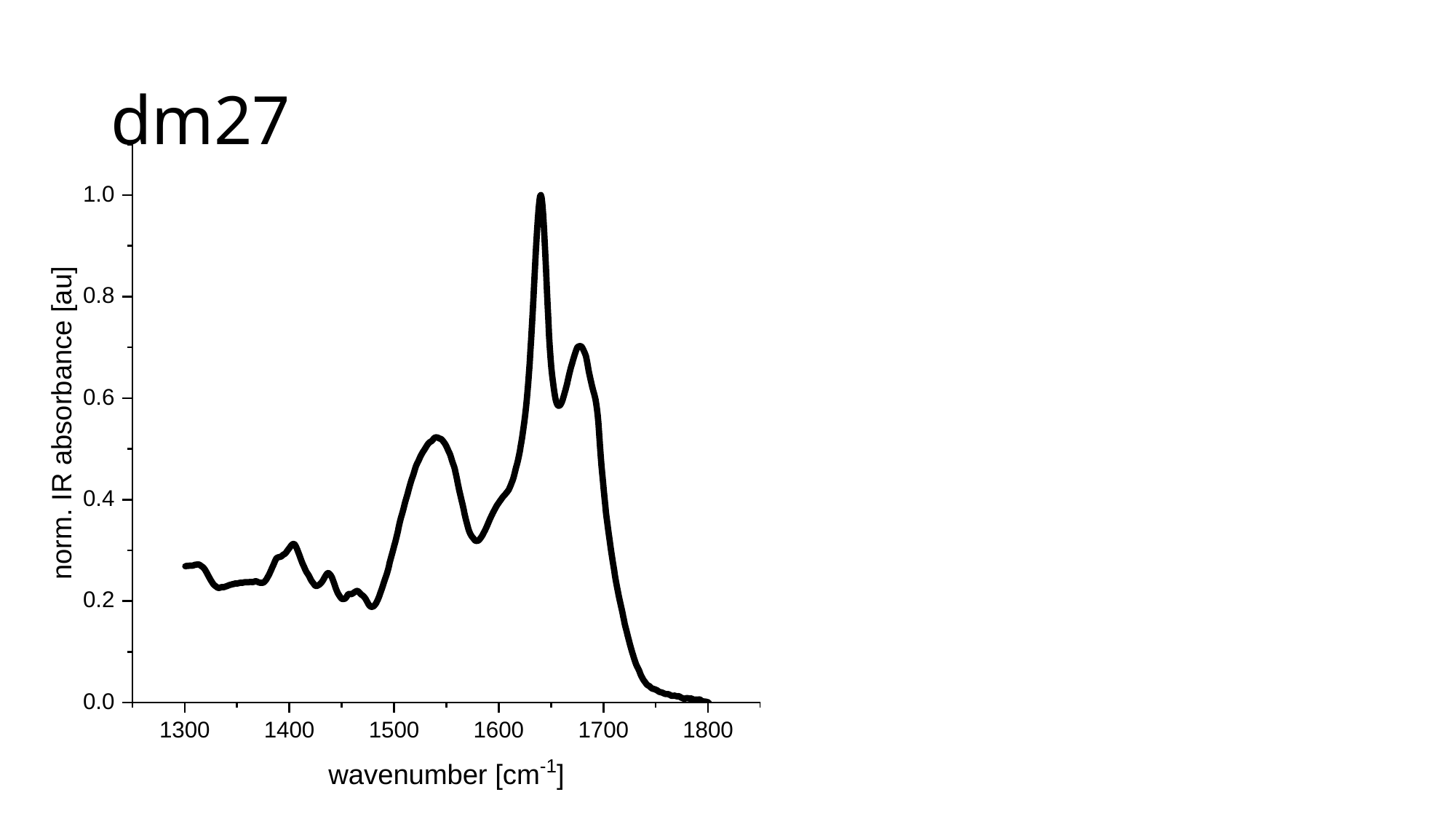

# dm27

## Slide 201
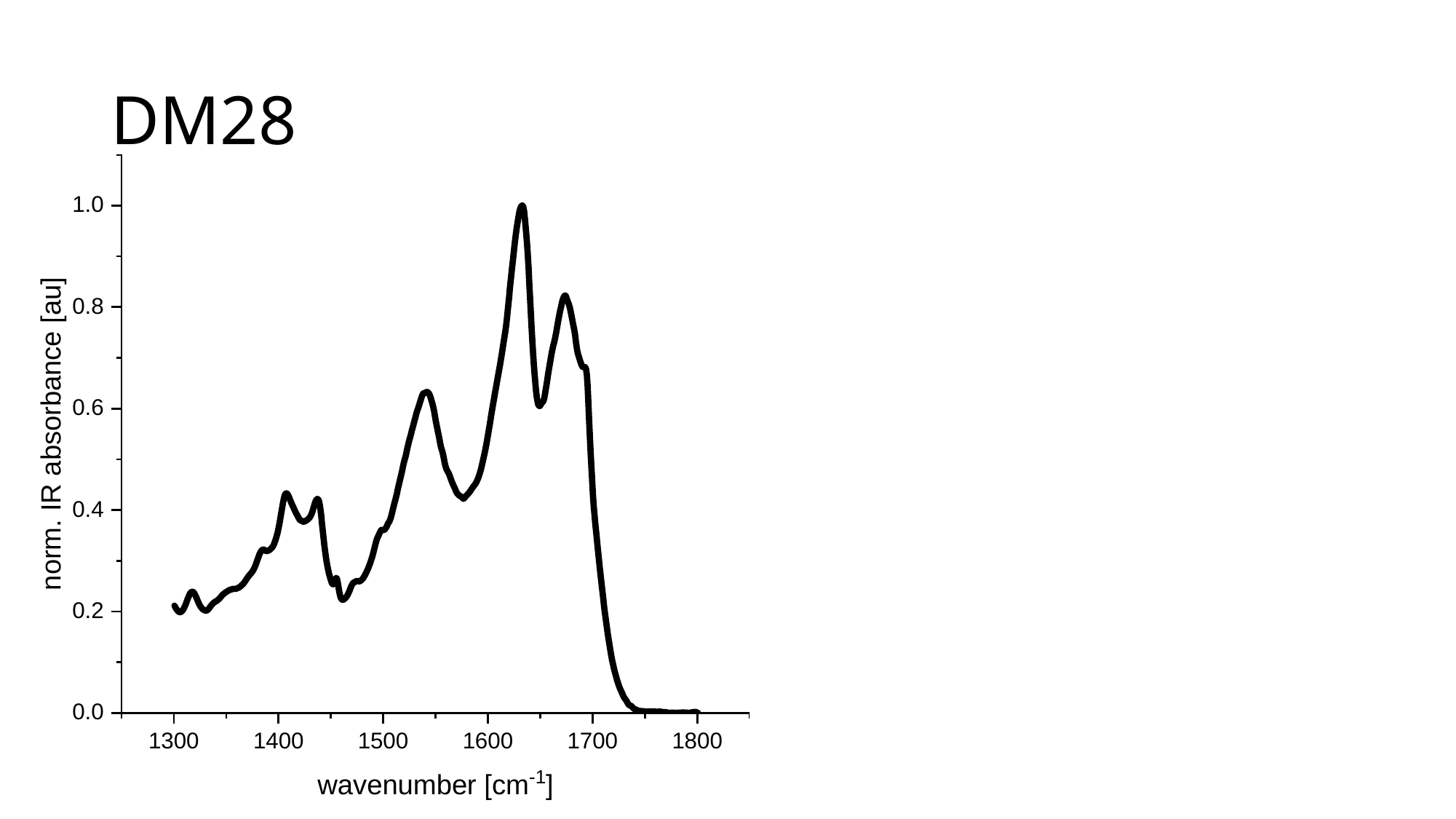

# DM28

## Slide 202
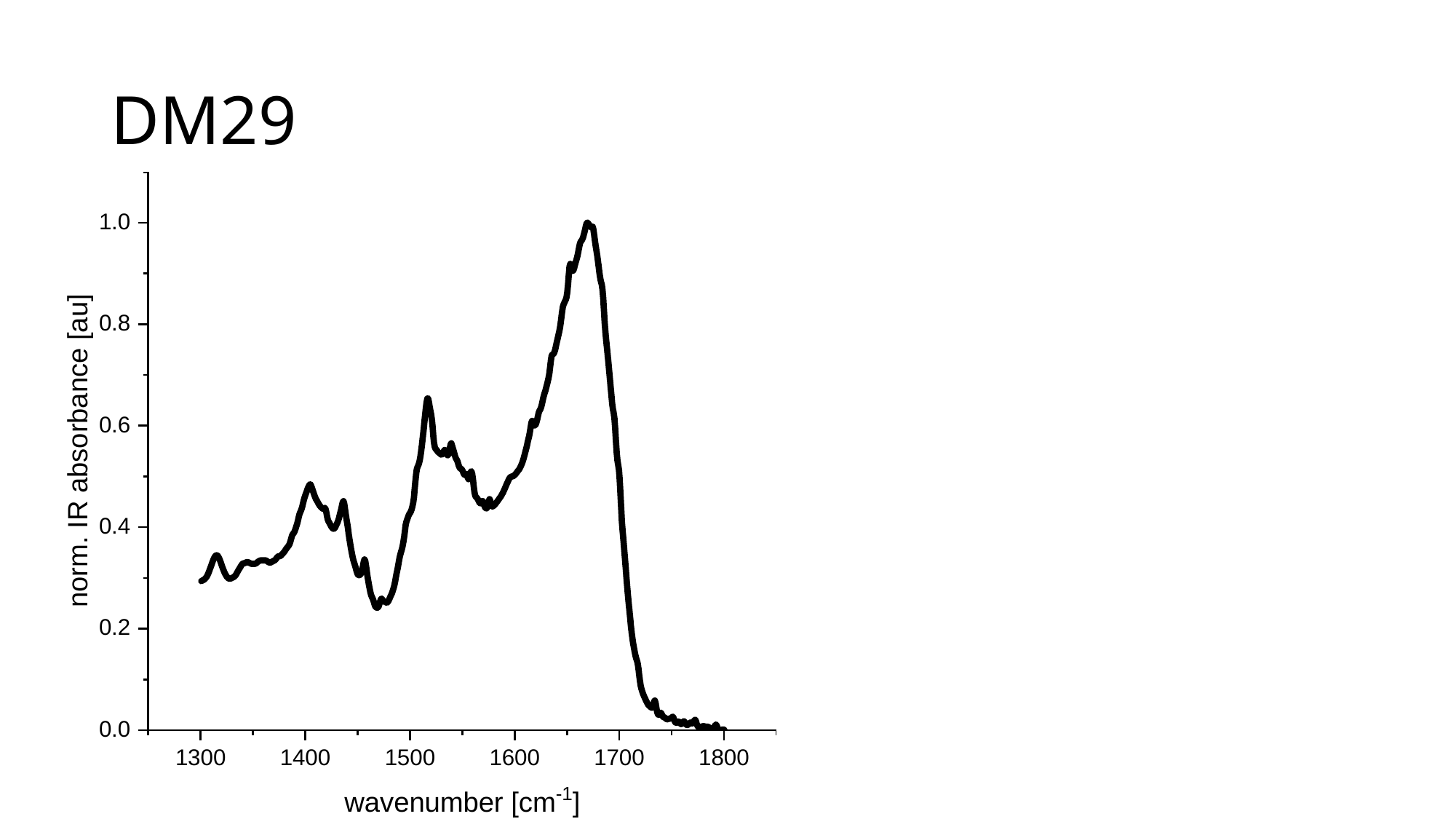

# DM29

## Slide 203
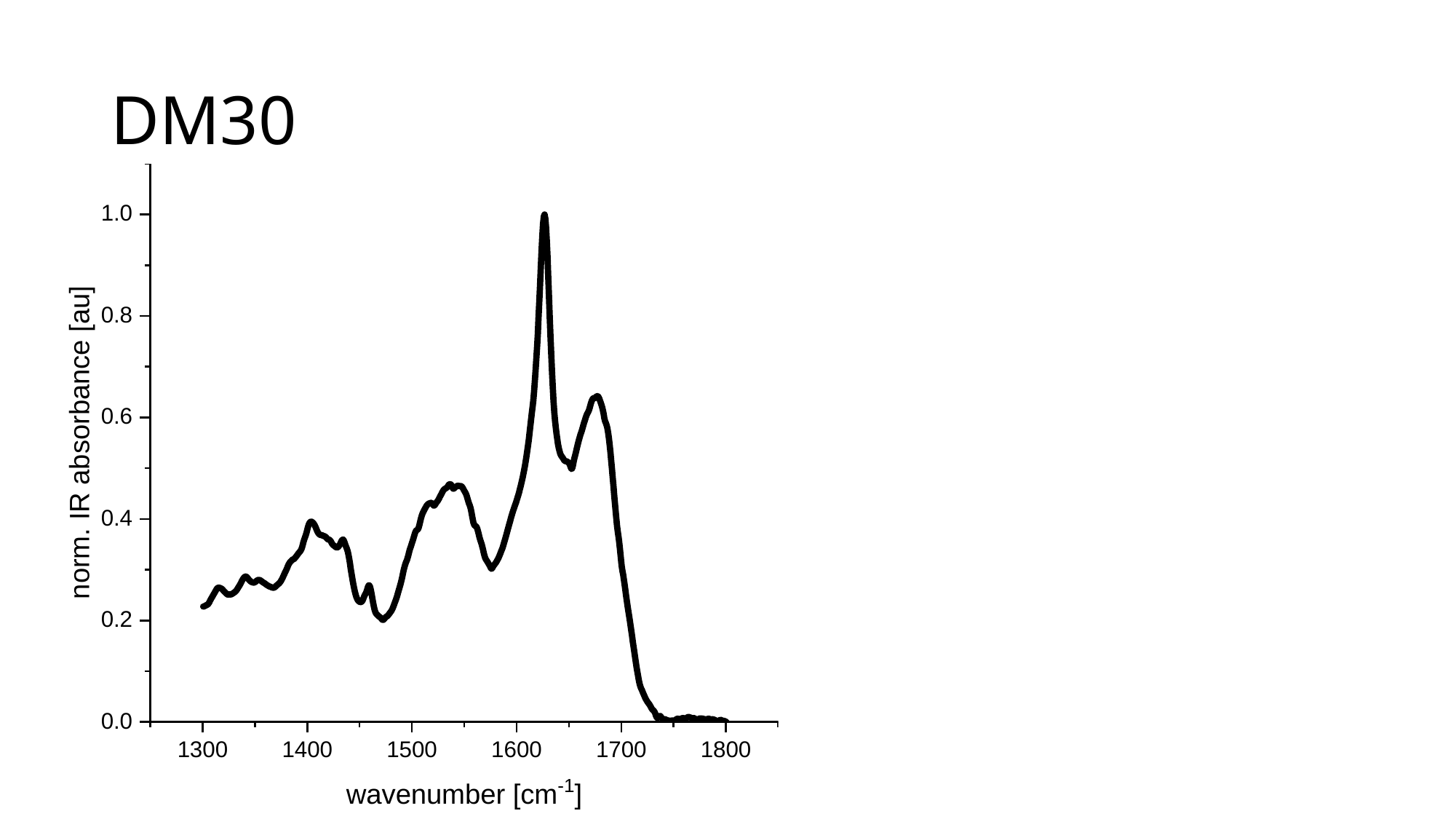

# DM30

## Slide 204
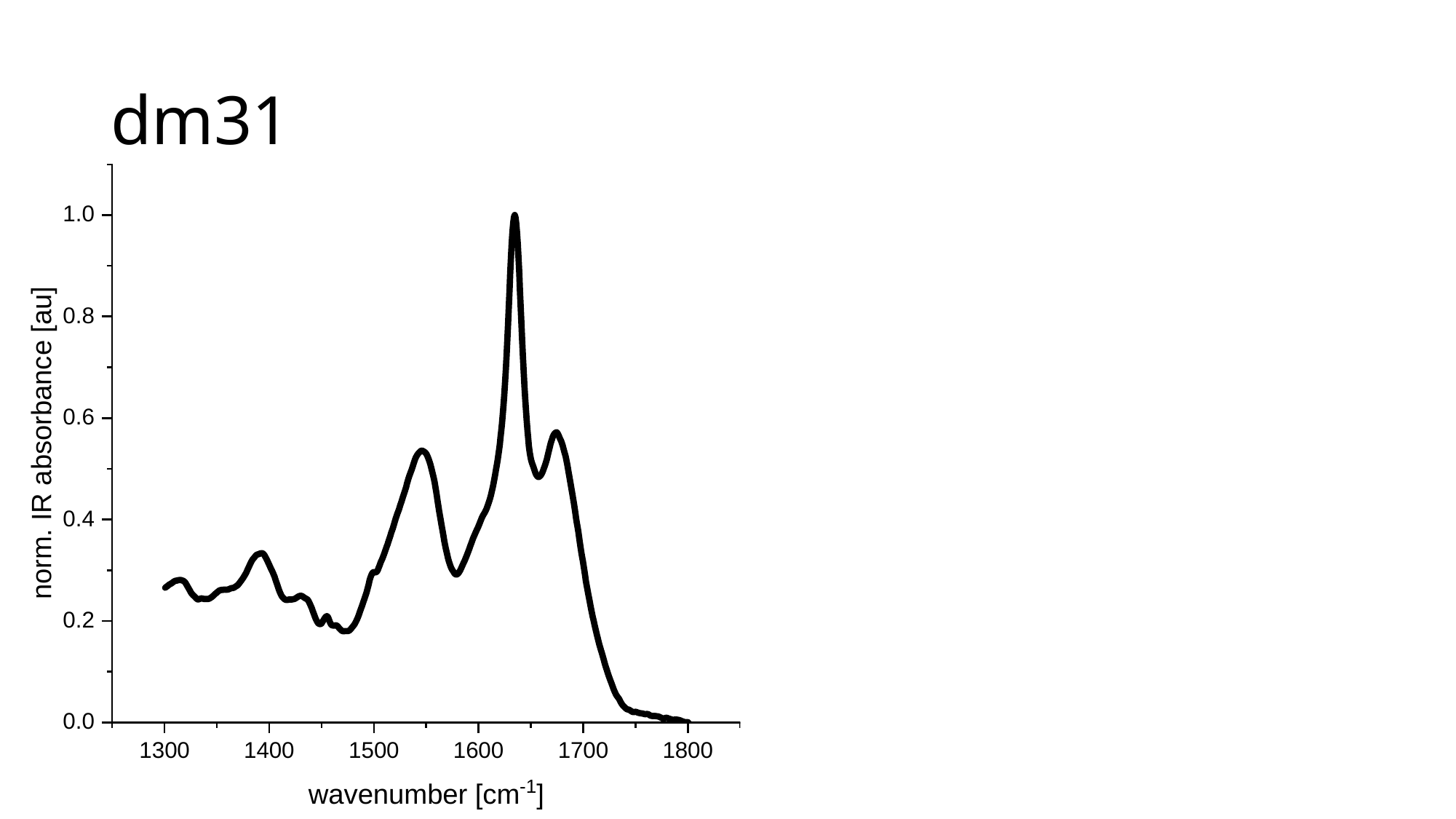

# dm31

## Slide 205
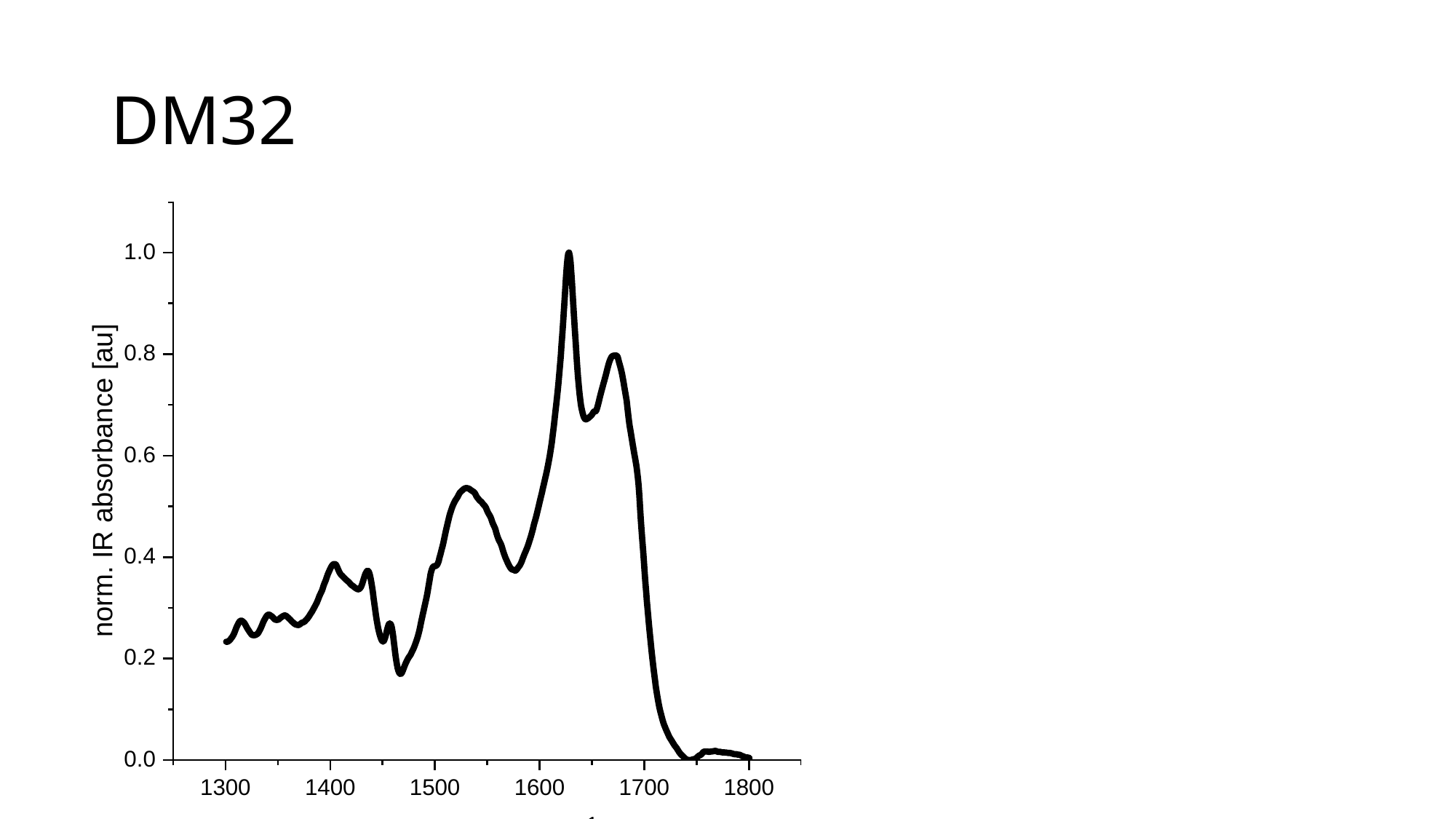

# DM32

## Slide 206
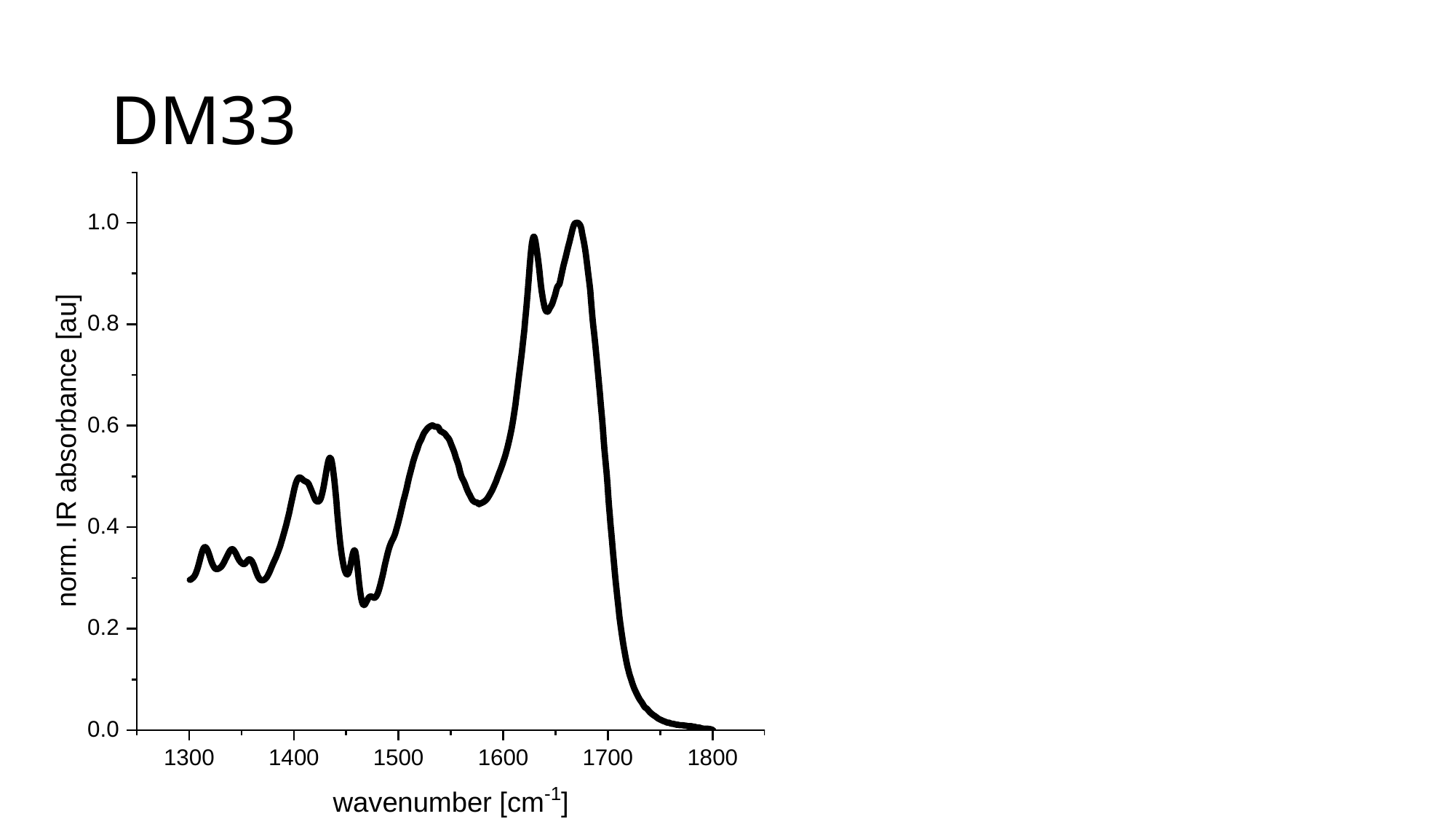

# DM33

## Slide 207
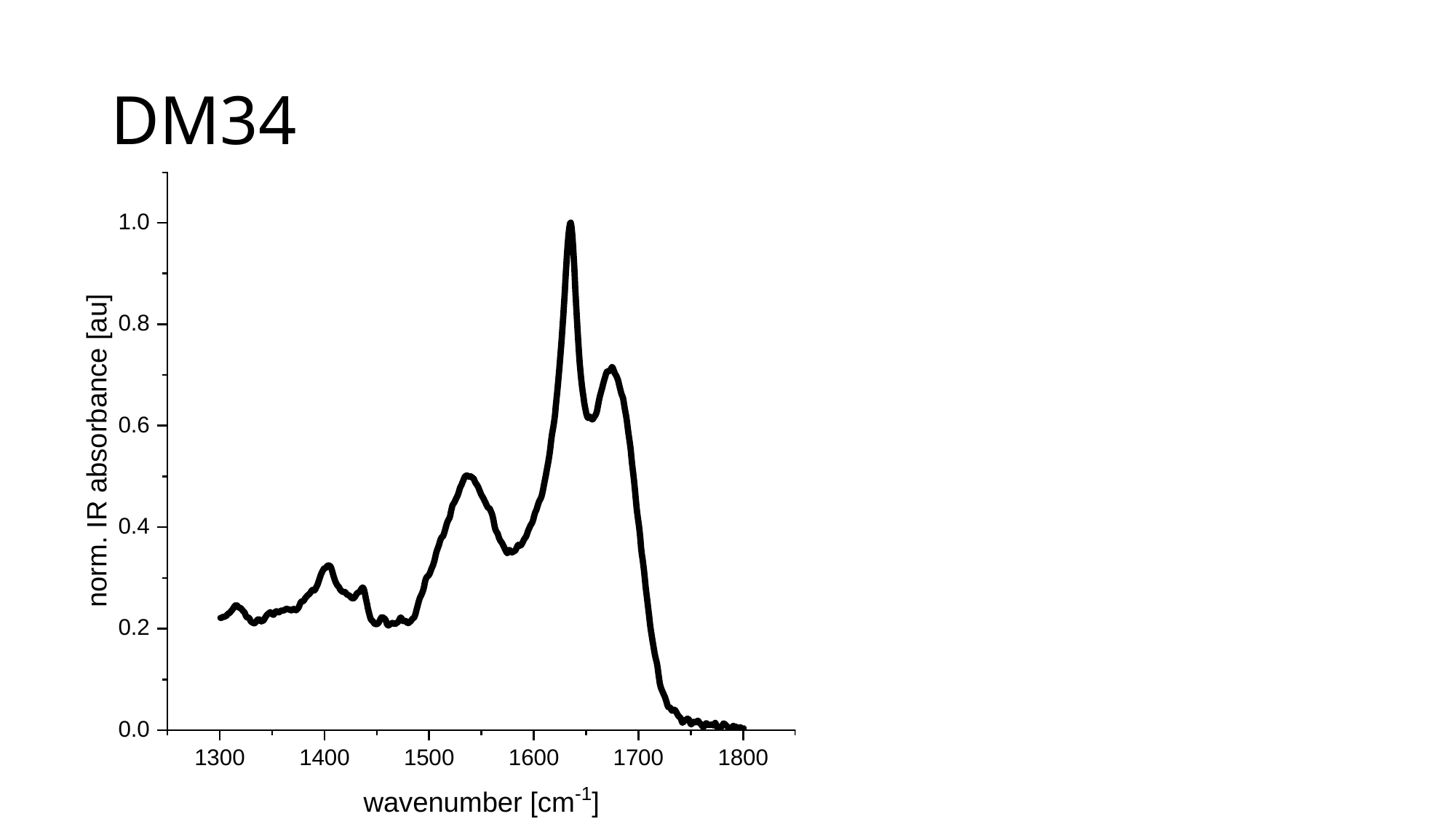

# DM34

## Slide 208
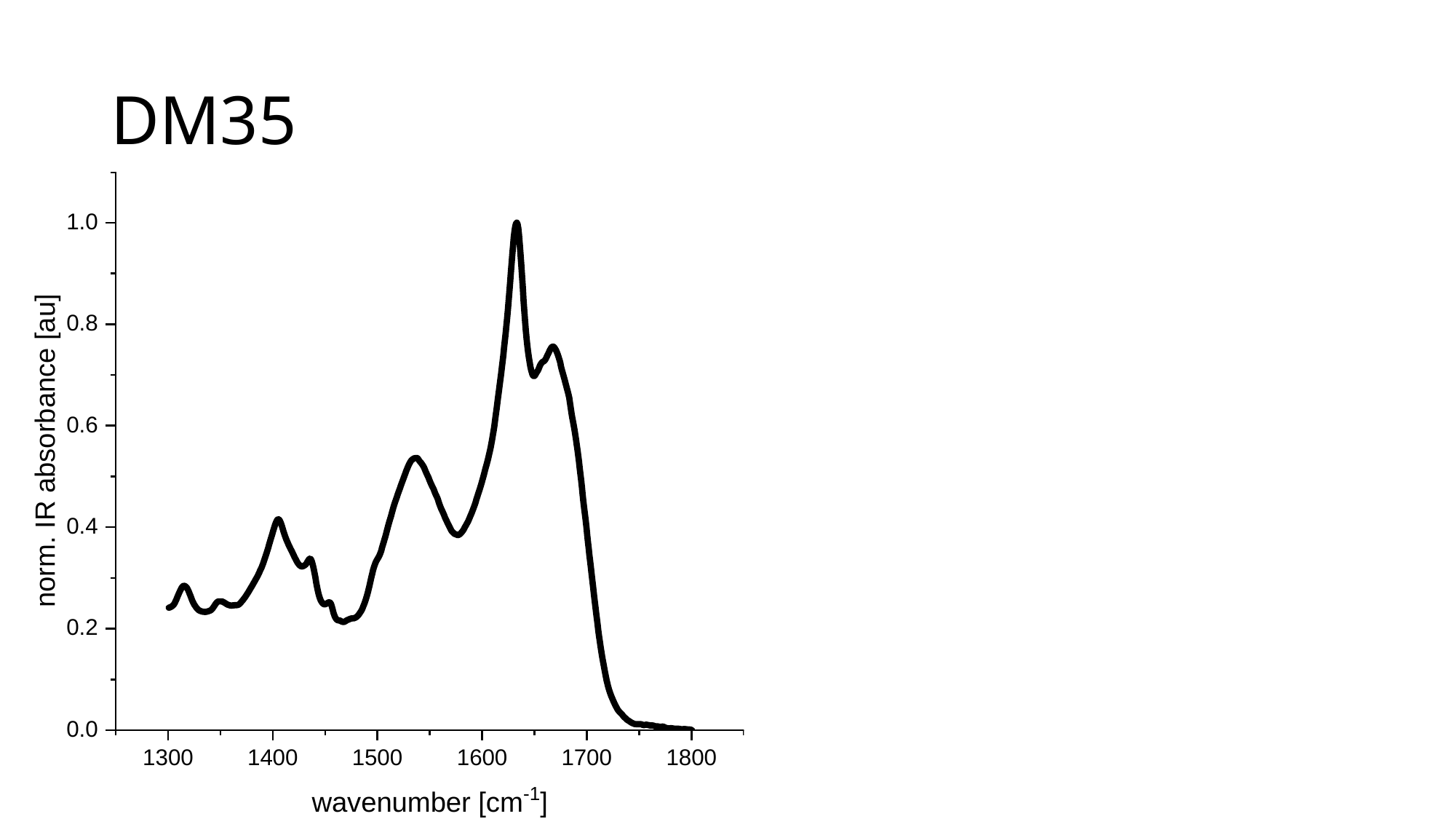

# DM35

## Slide 209
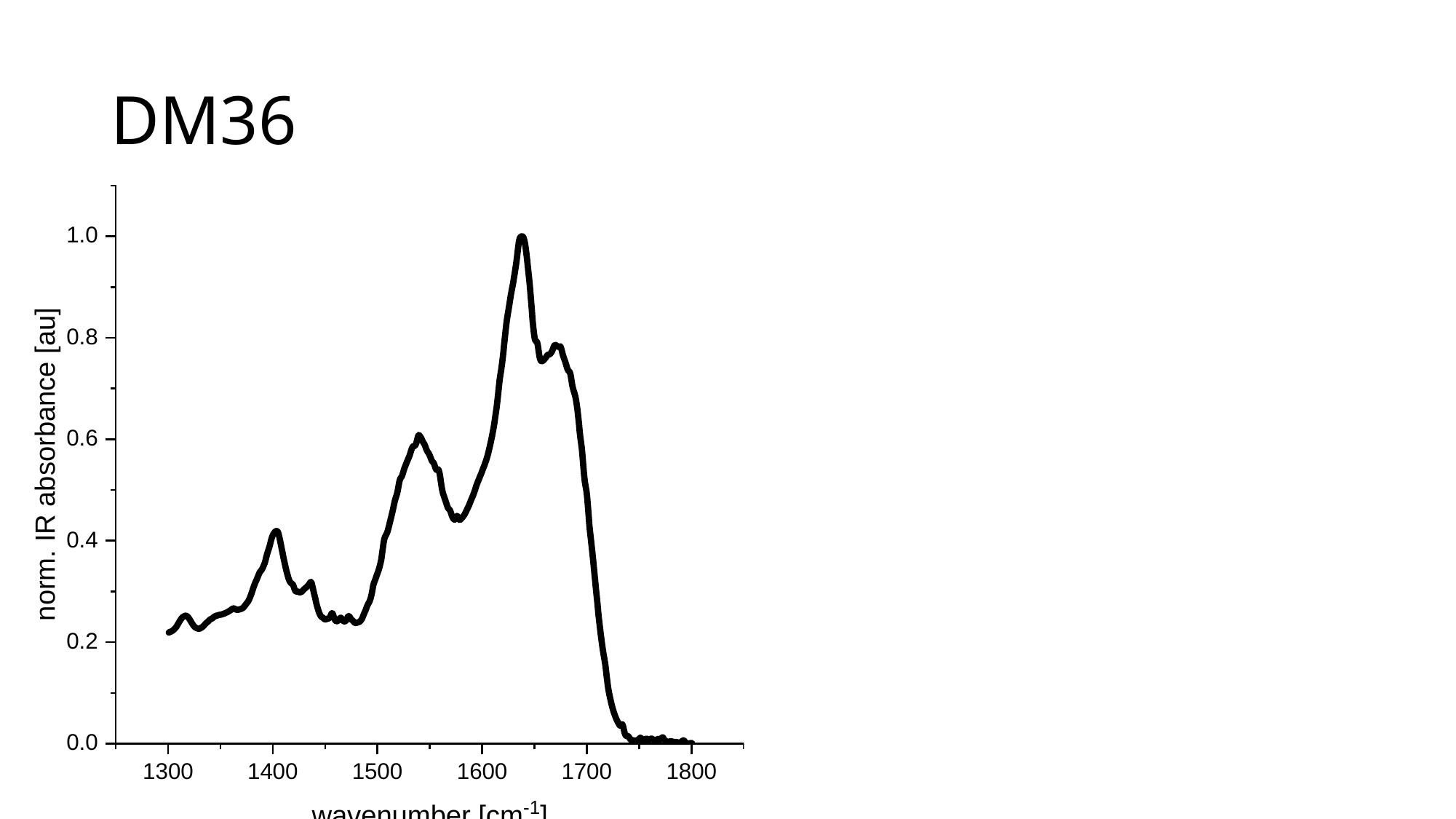

# DM36

## Slide 210
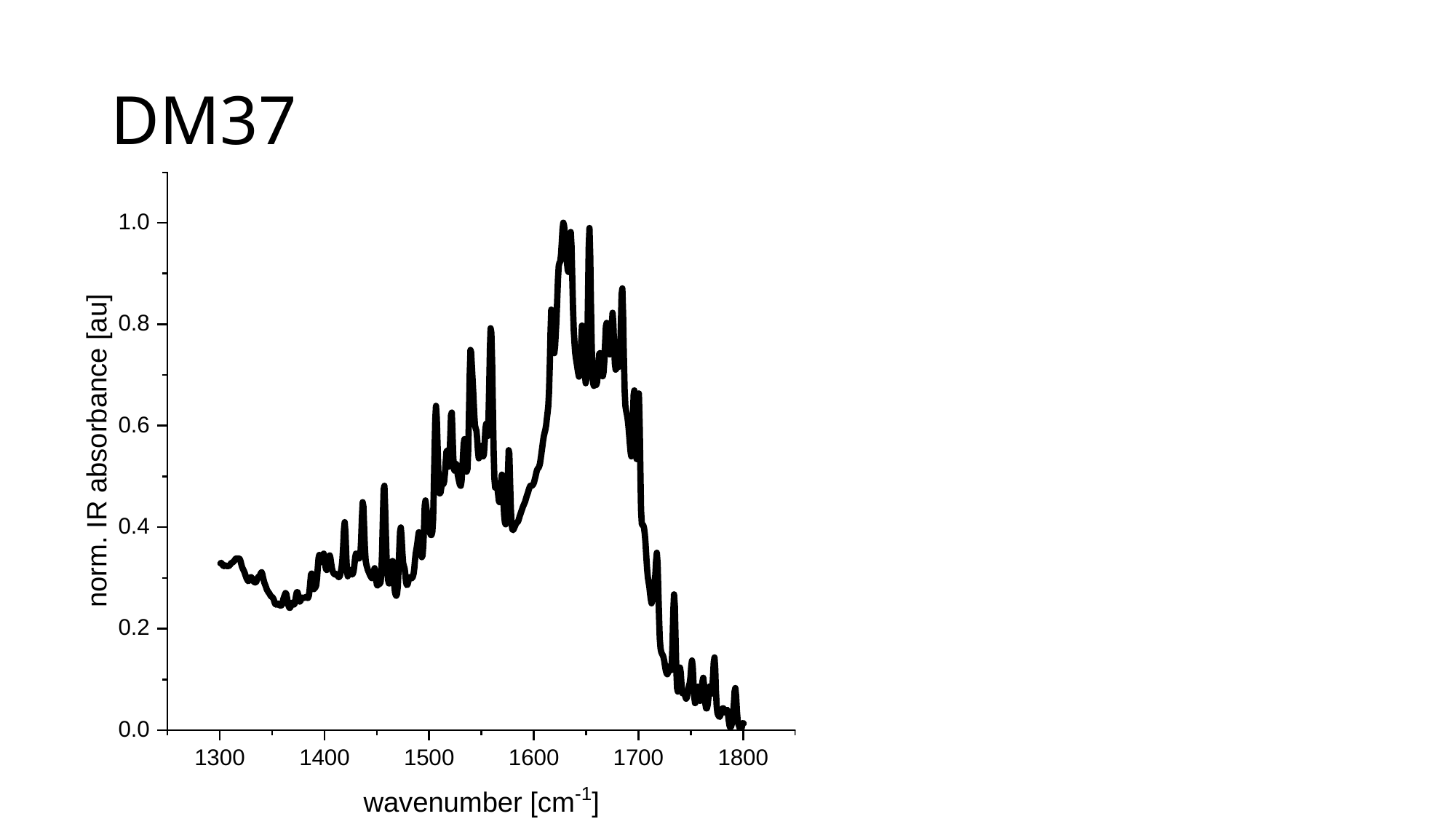

# DM37

## Slide 211
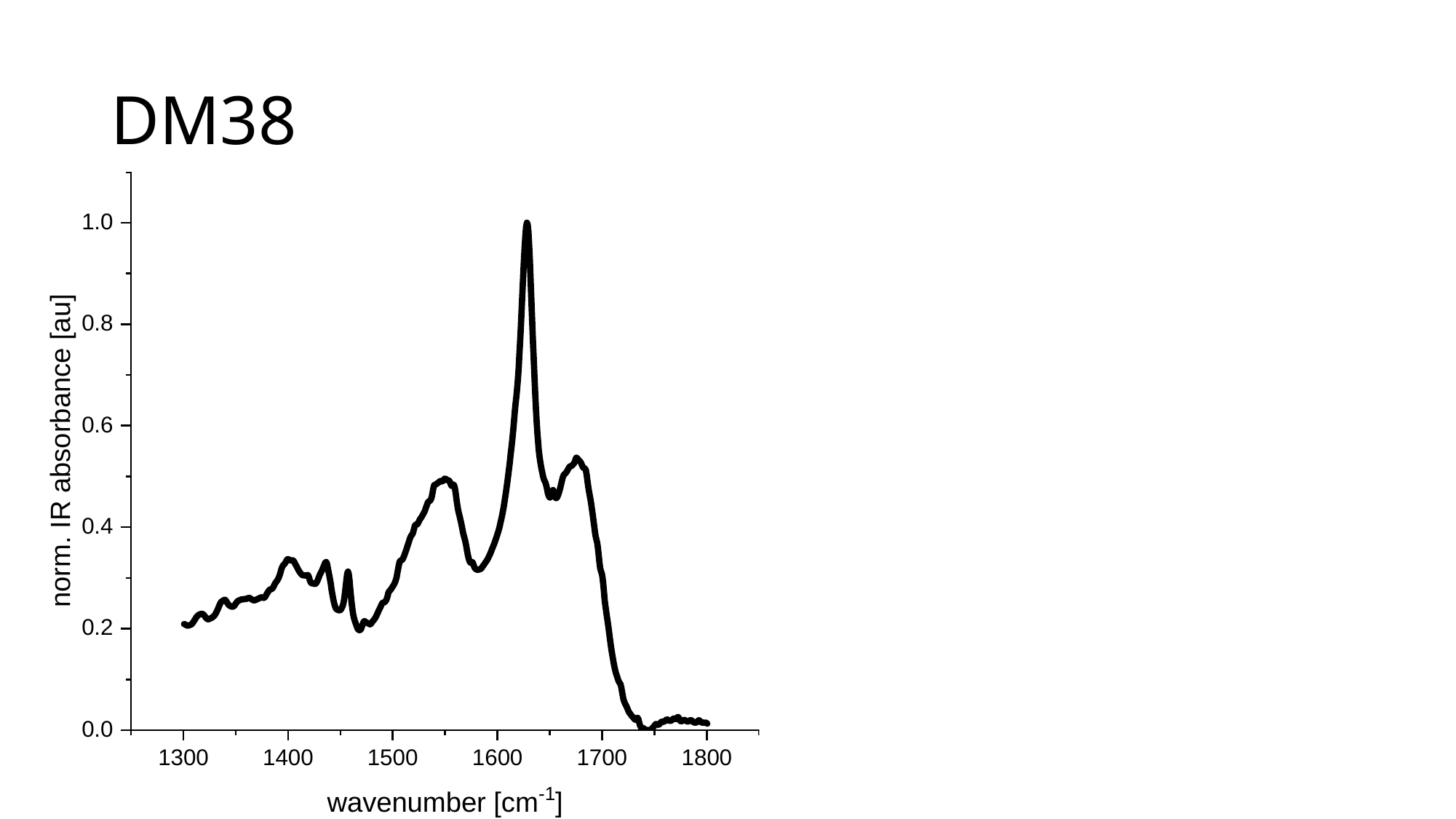

# DM38

## Slide 212
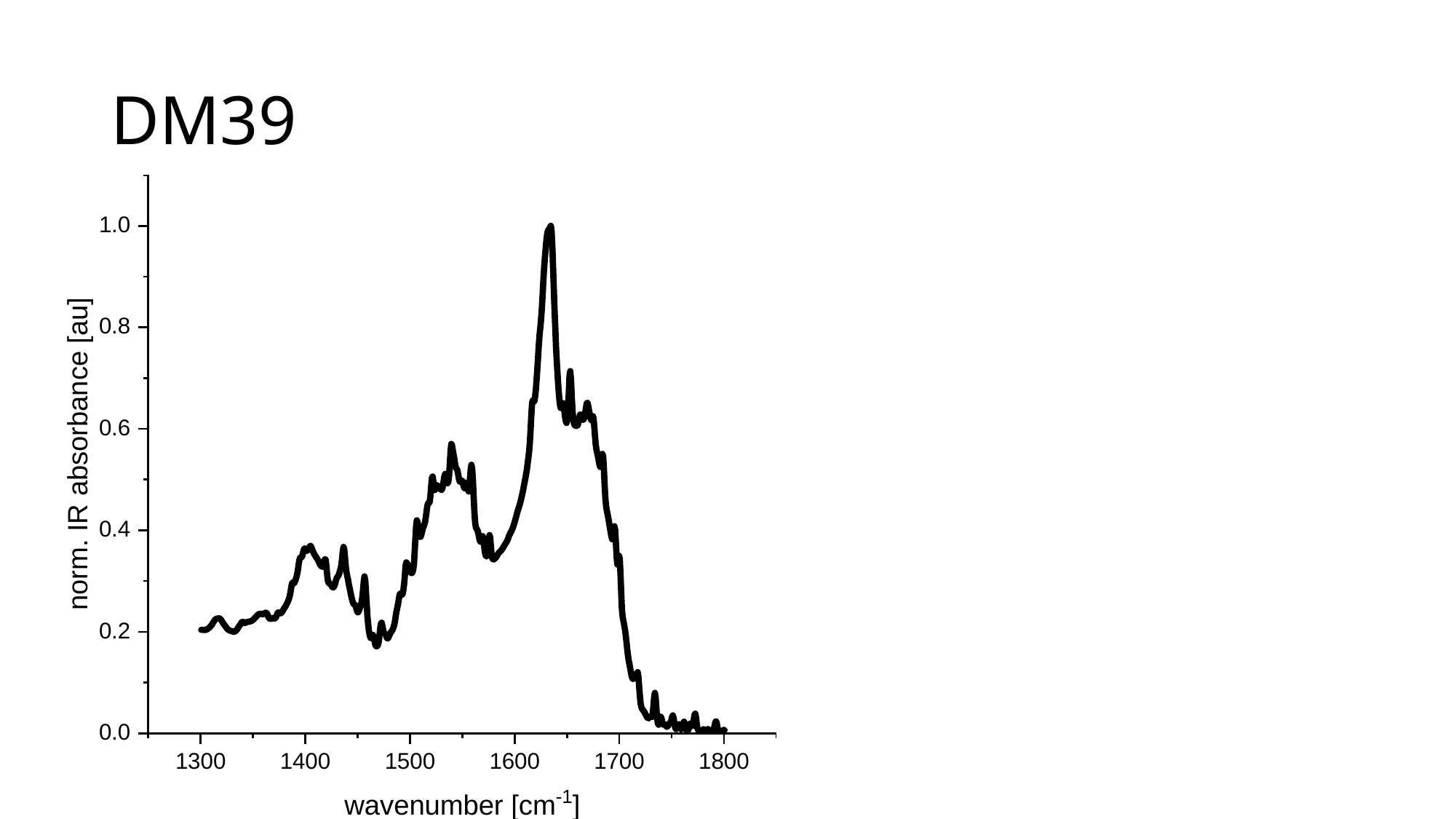

# DM39

## Slide 213
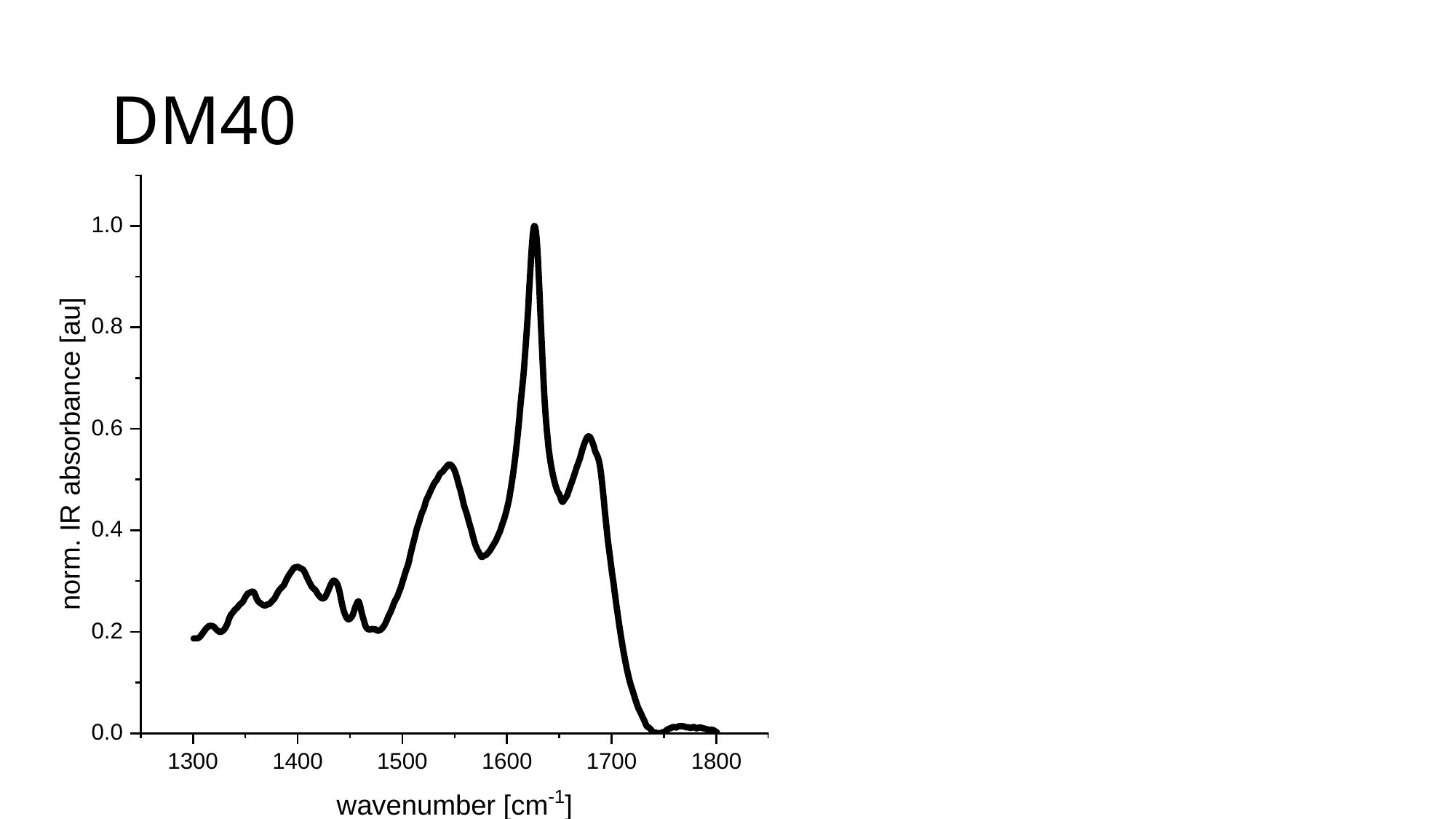

# DM40

## Slide 214
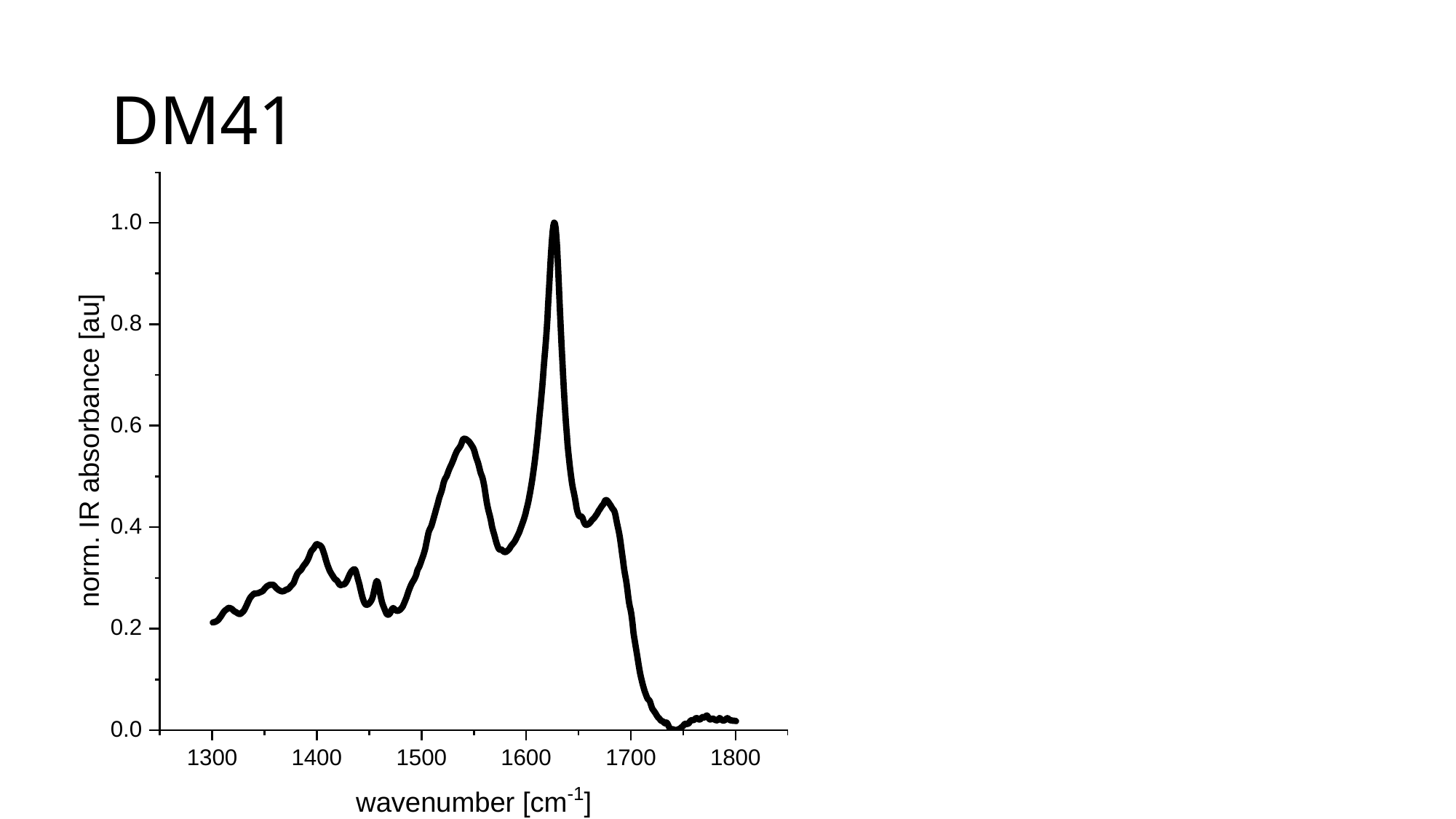

# DM41

## Slide 215
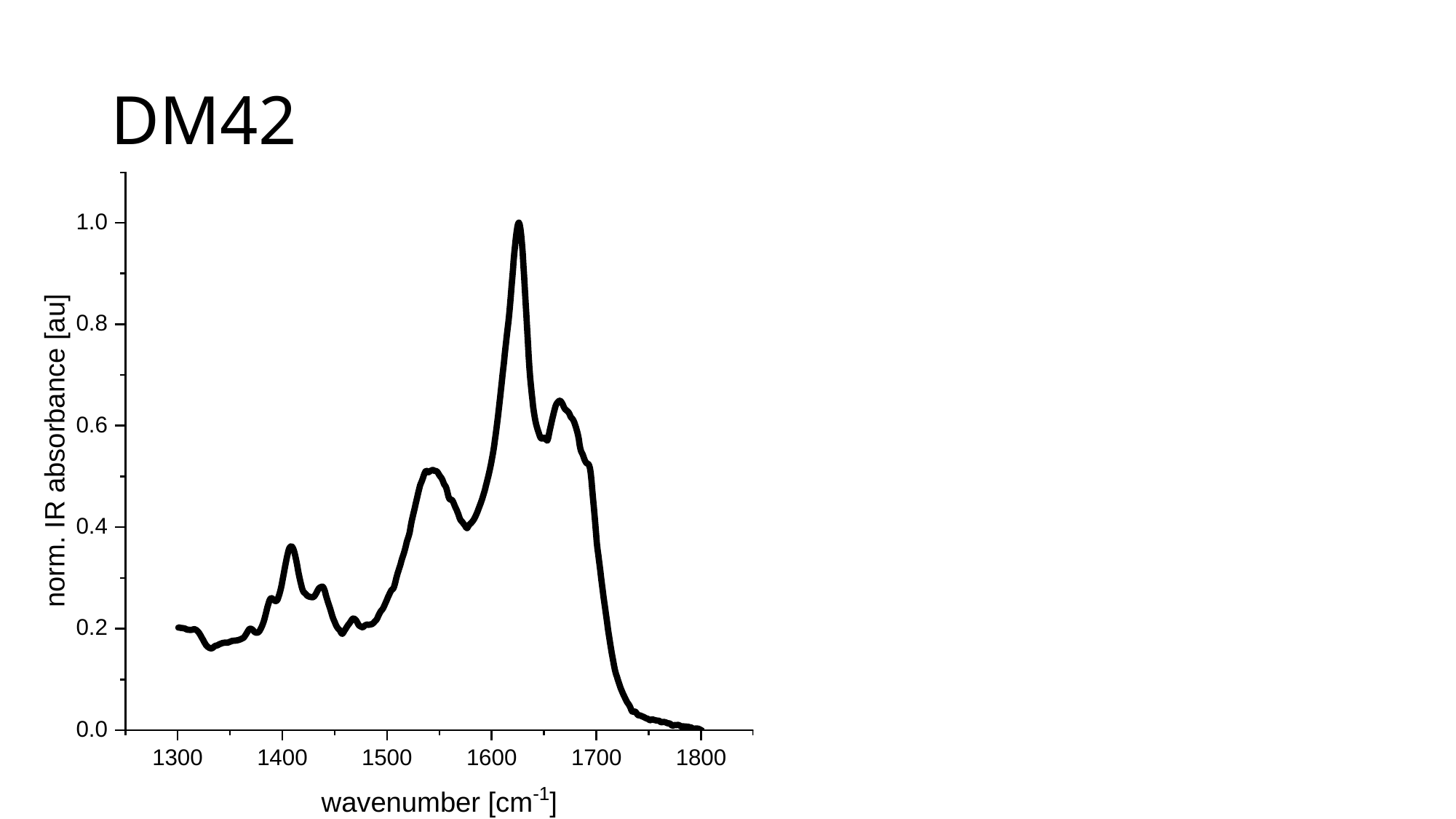

# DM42

## Slide 216
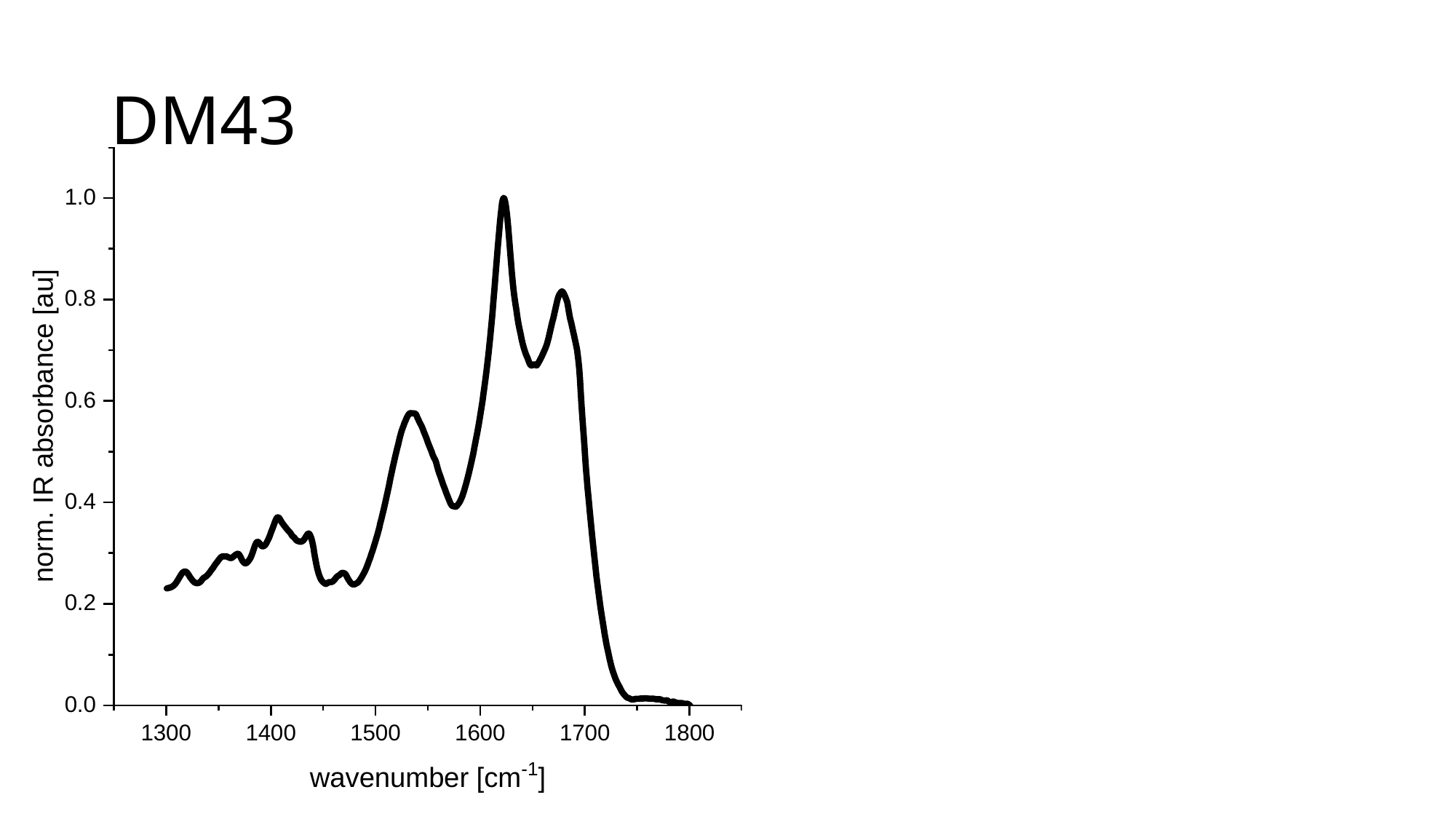

# DM43

## Slide 217
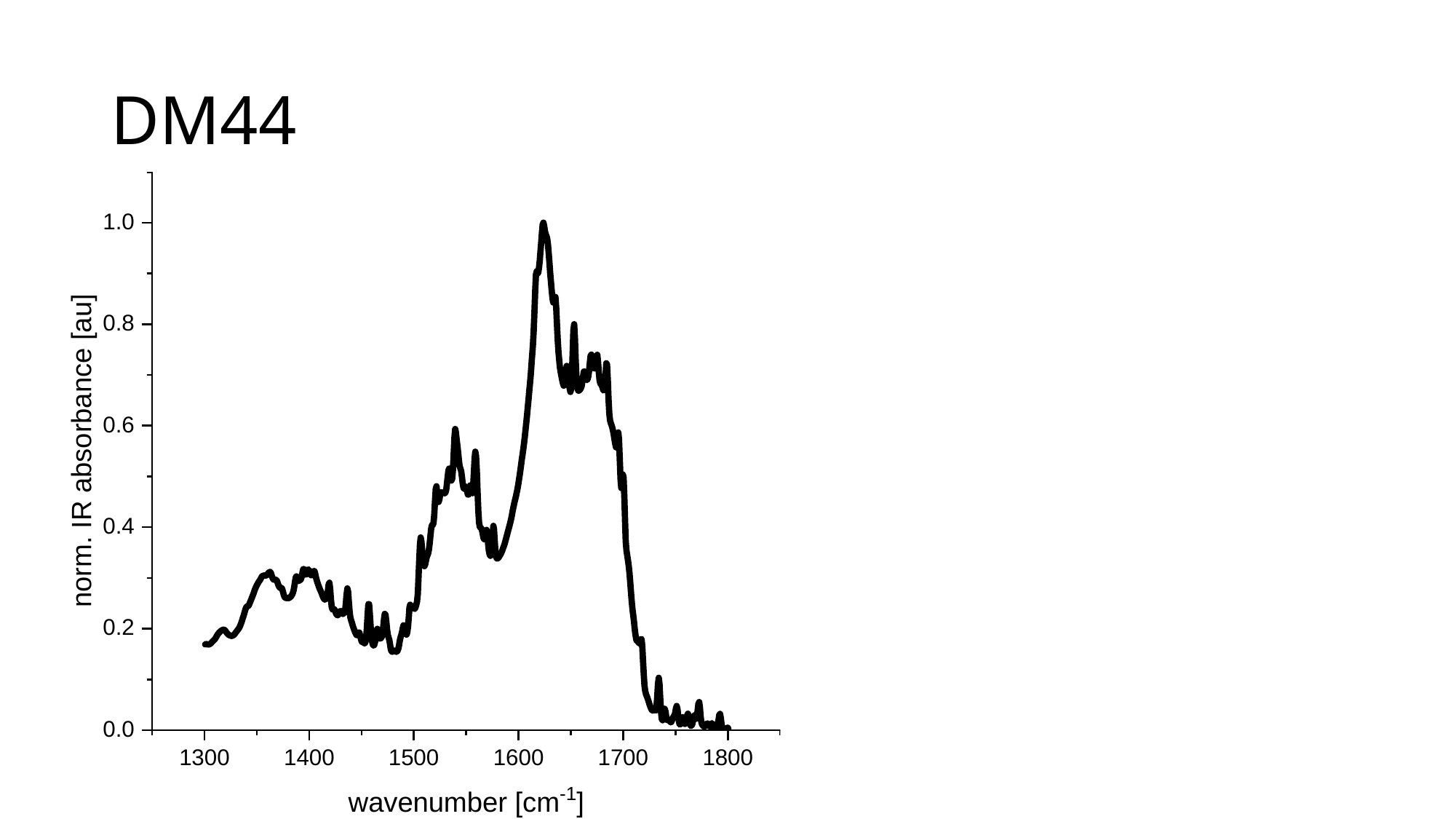

# DM44

## Slide 218
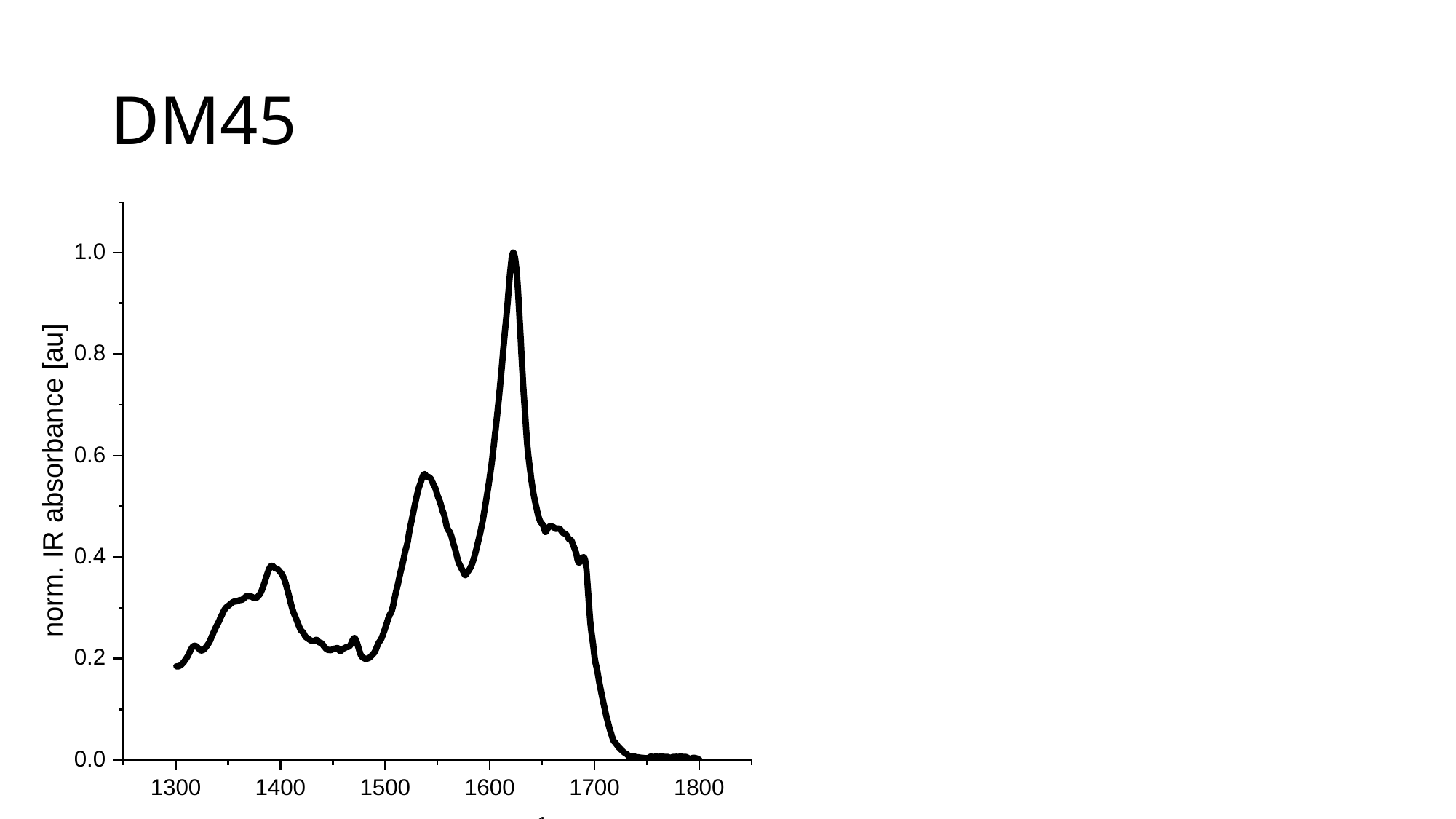

# DM45

## Slide 219
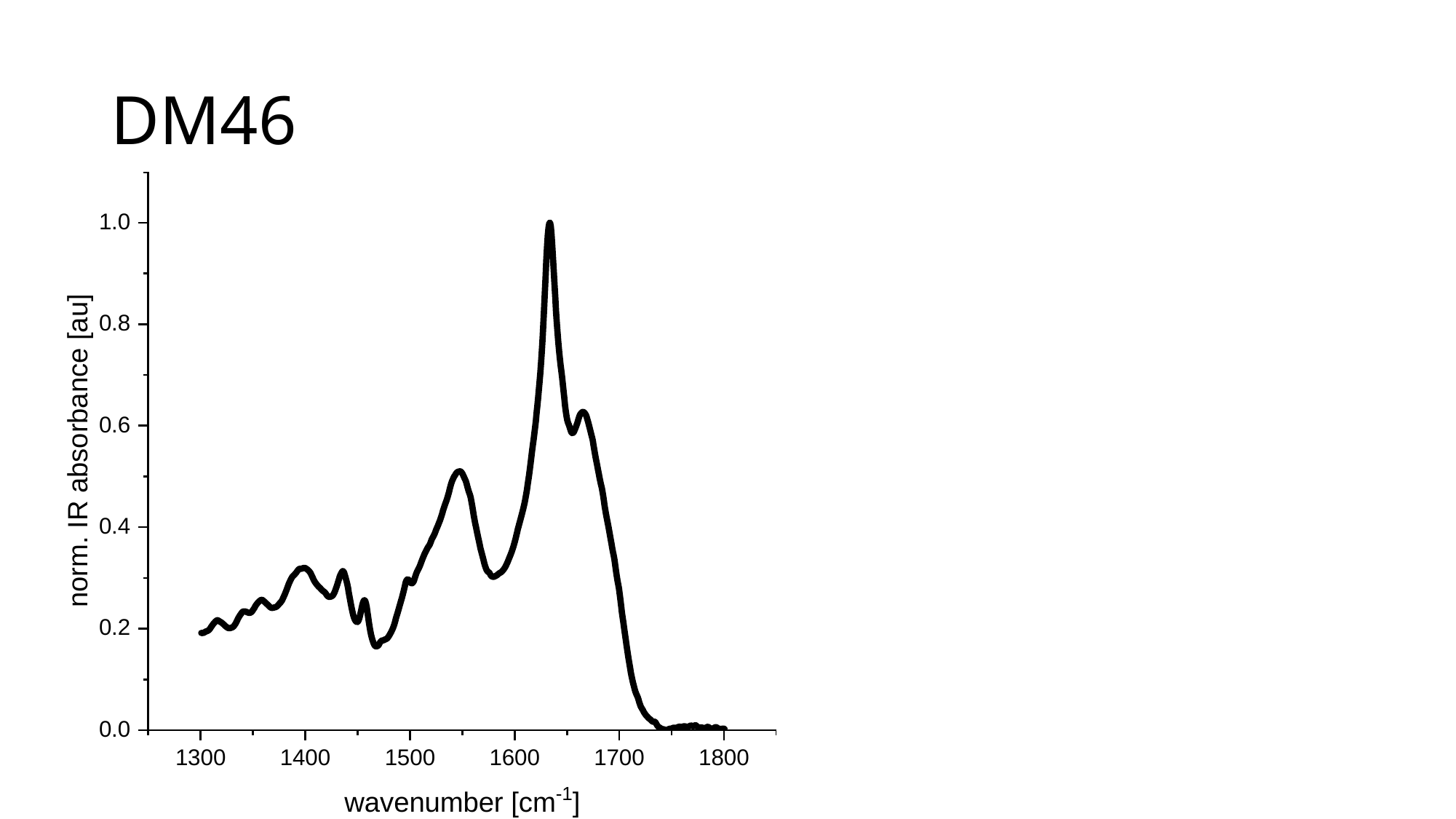

# DM46

## Slide 220
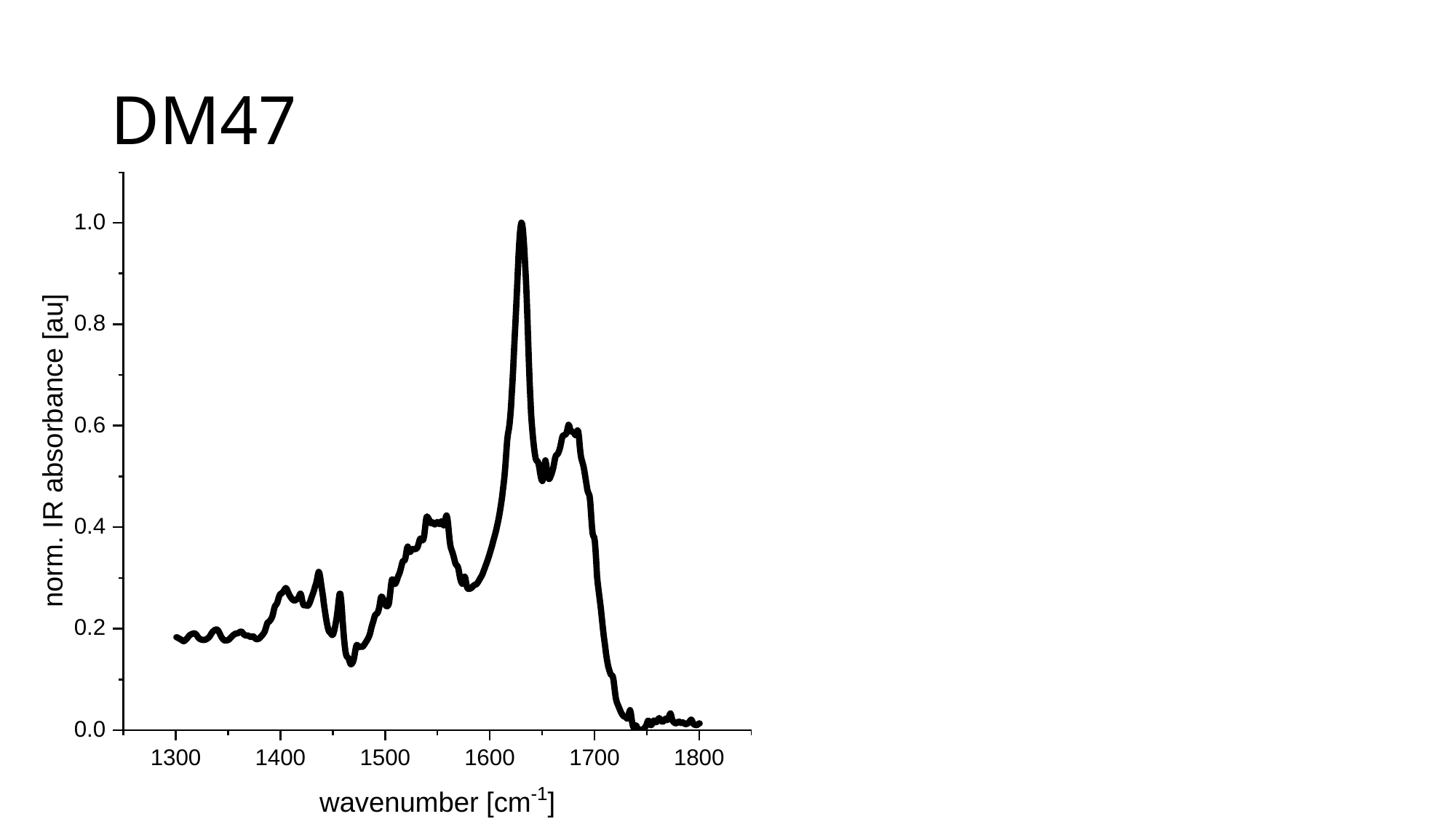

# DM47

## Slide 221
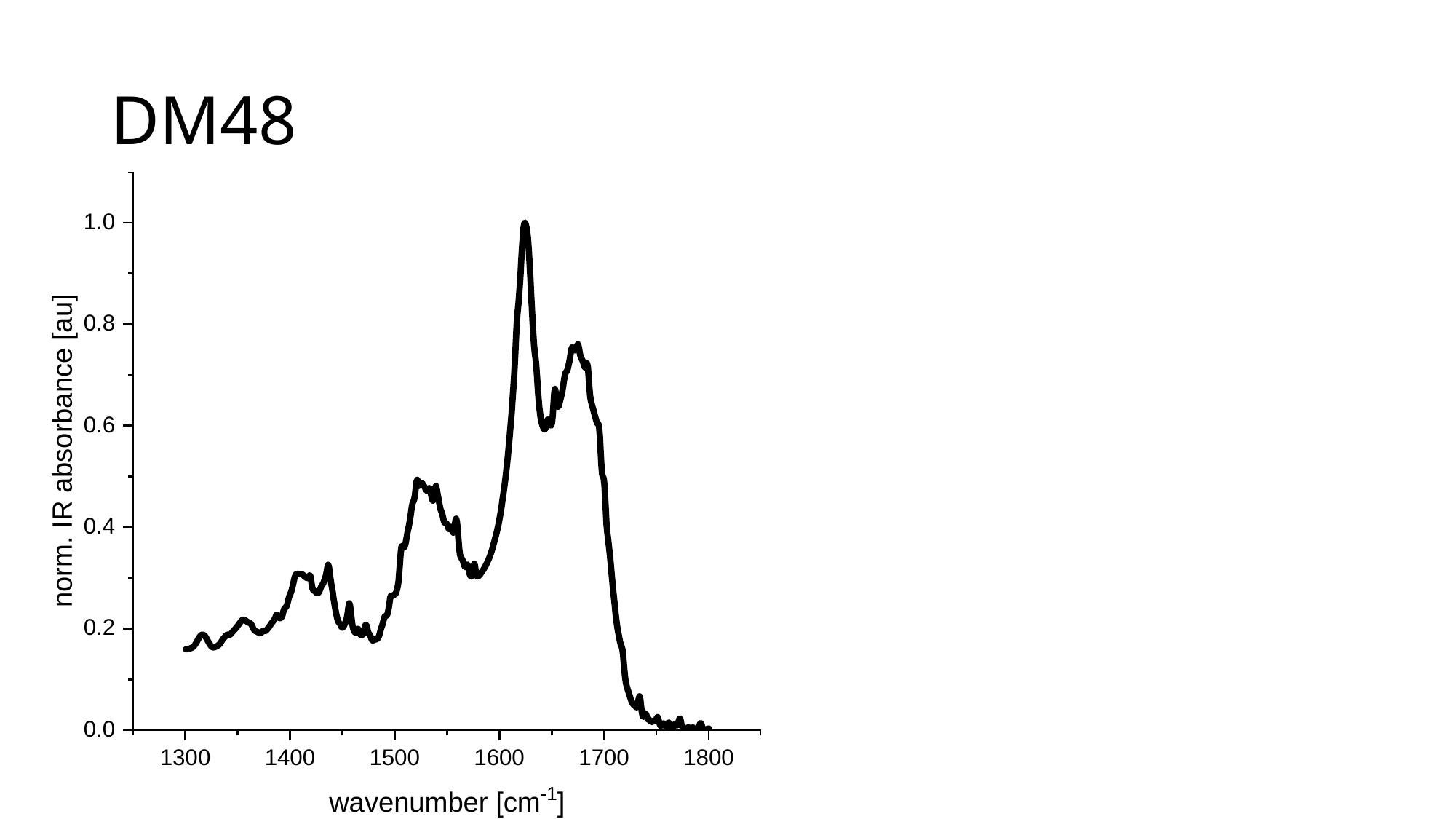

# DM48

## Slide 222
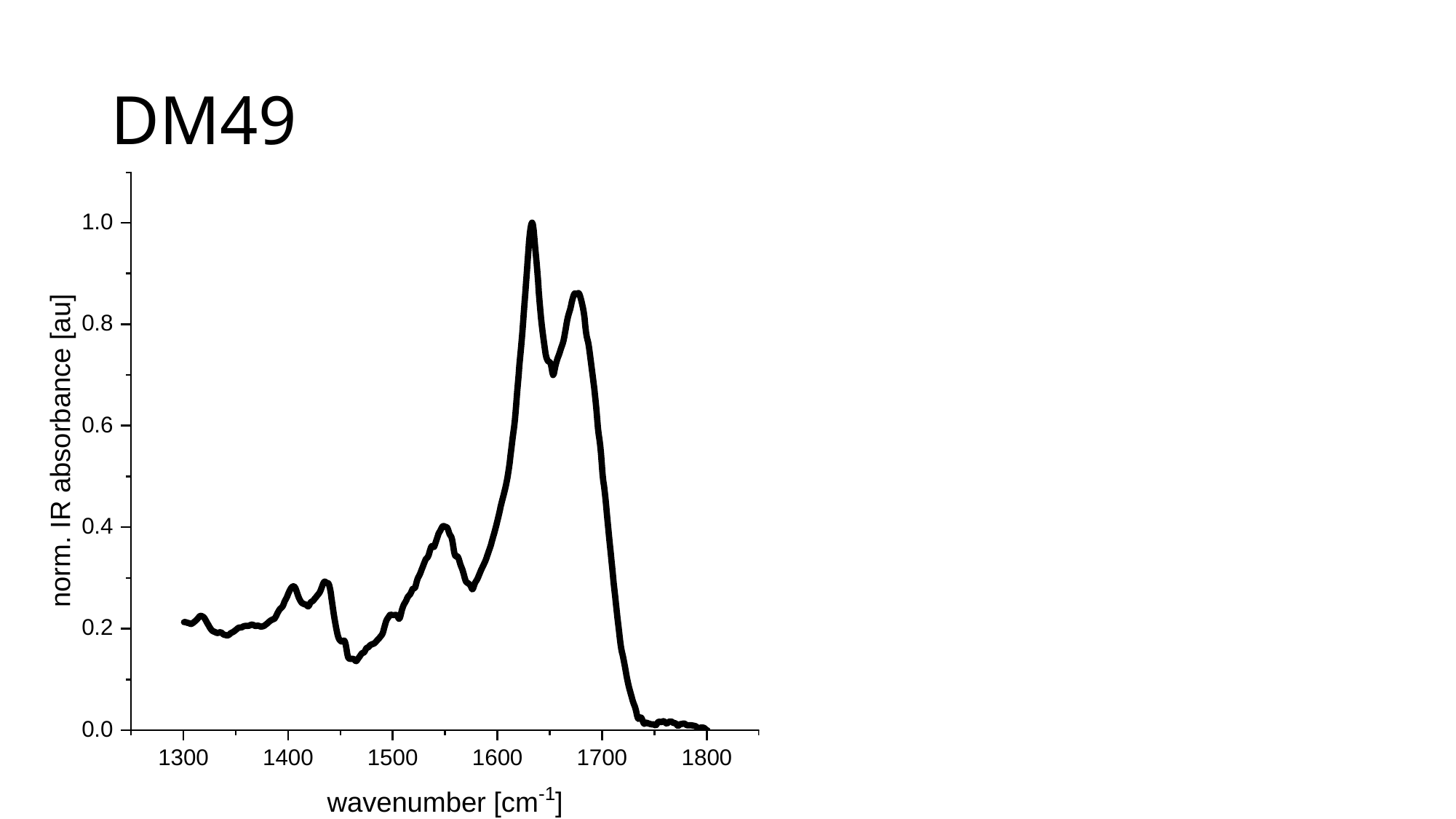

# DM49

## Slide 223
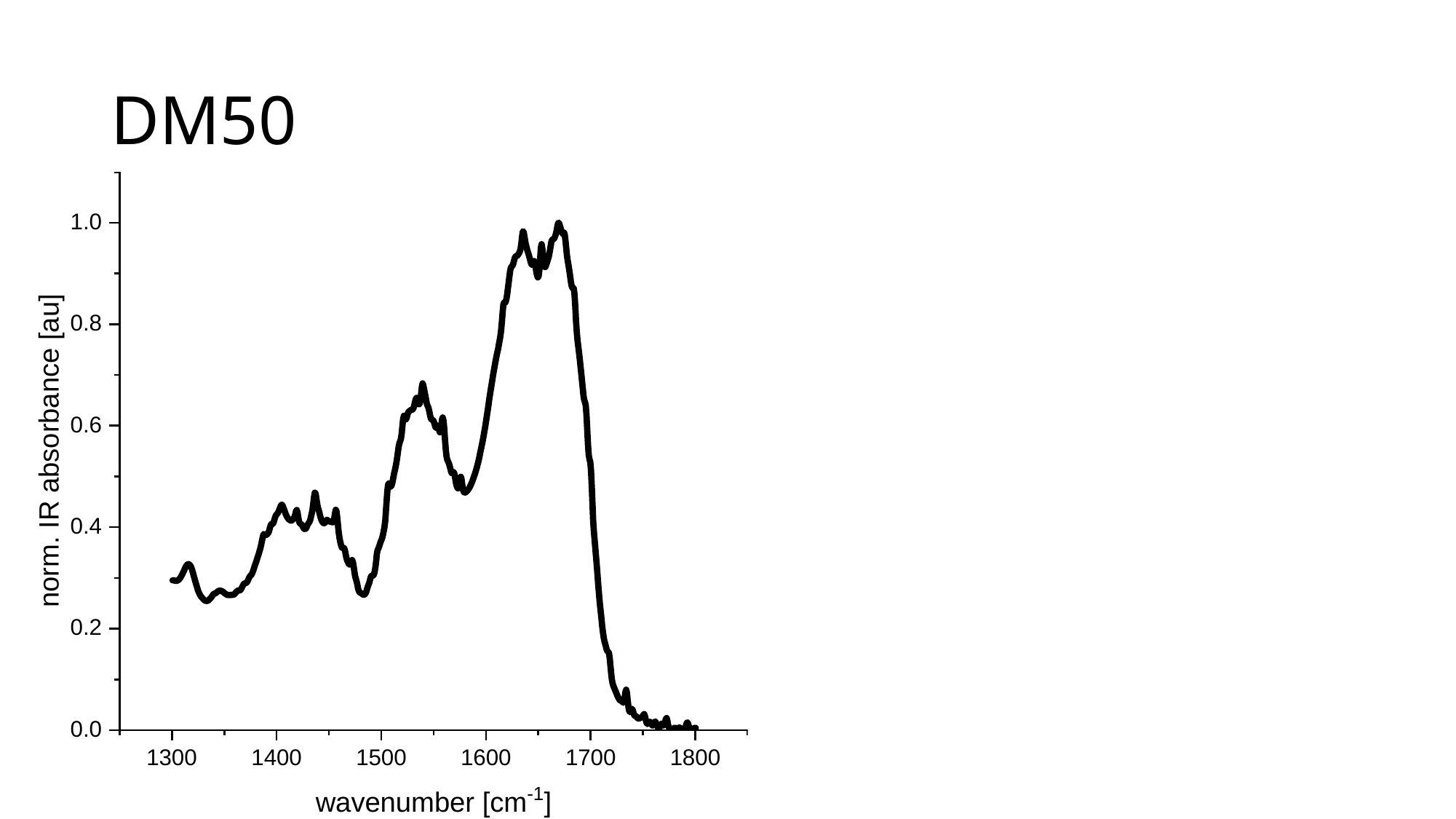

# DM50

## Slide 224
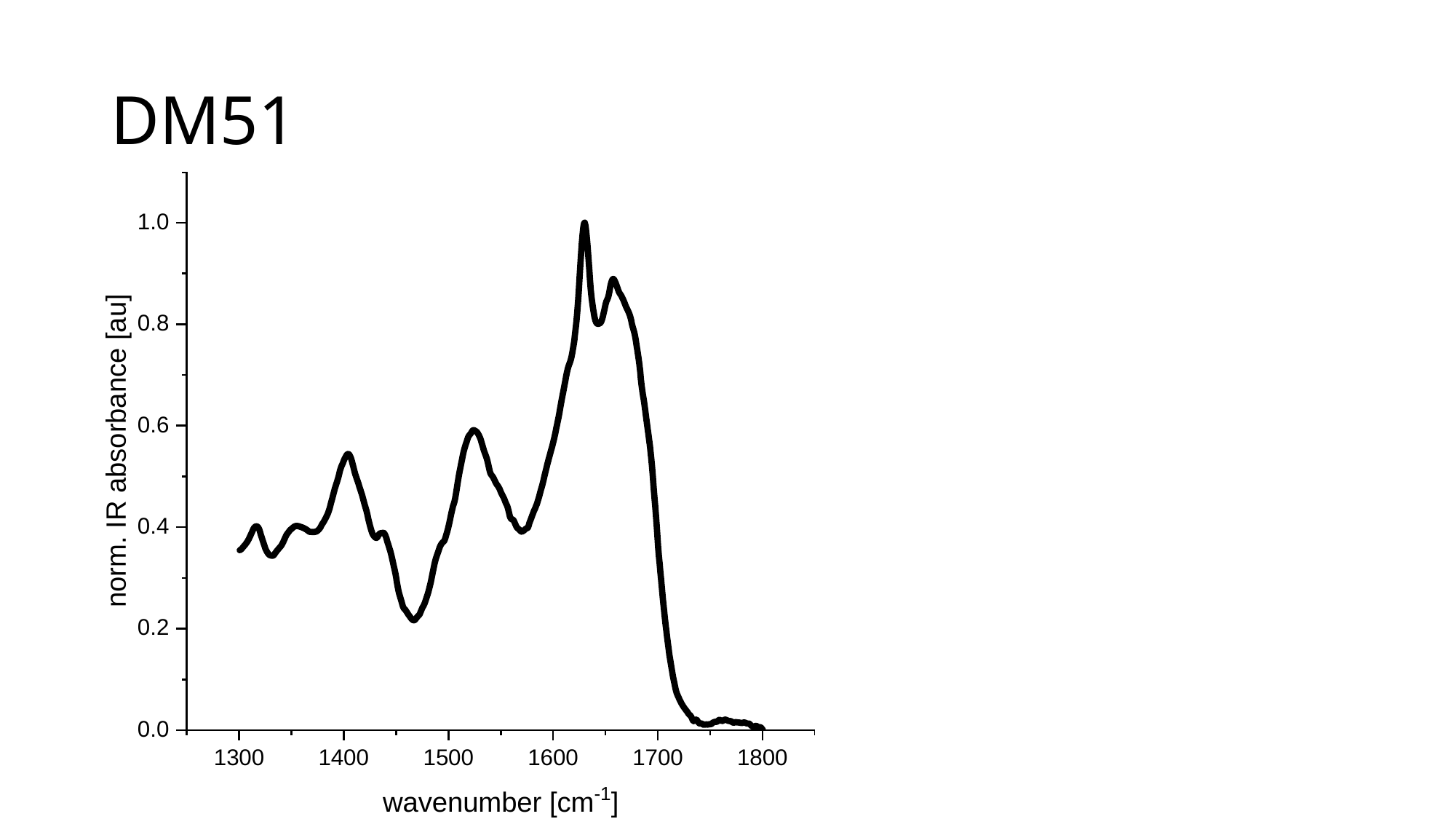

# DM51

## Slide 225
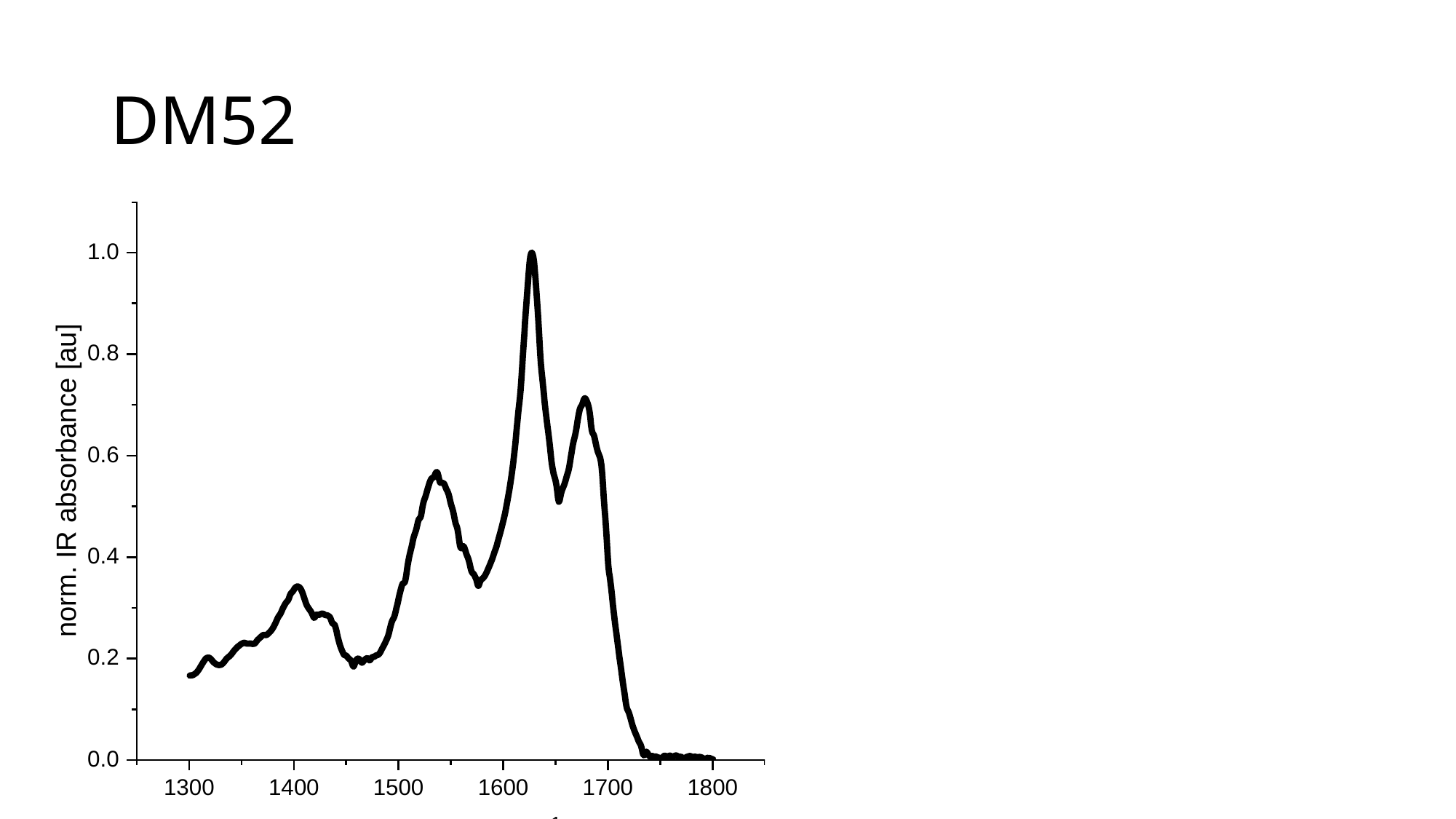

# DM52

## Slide 226
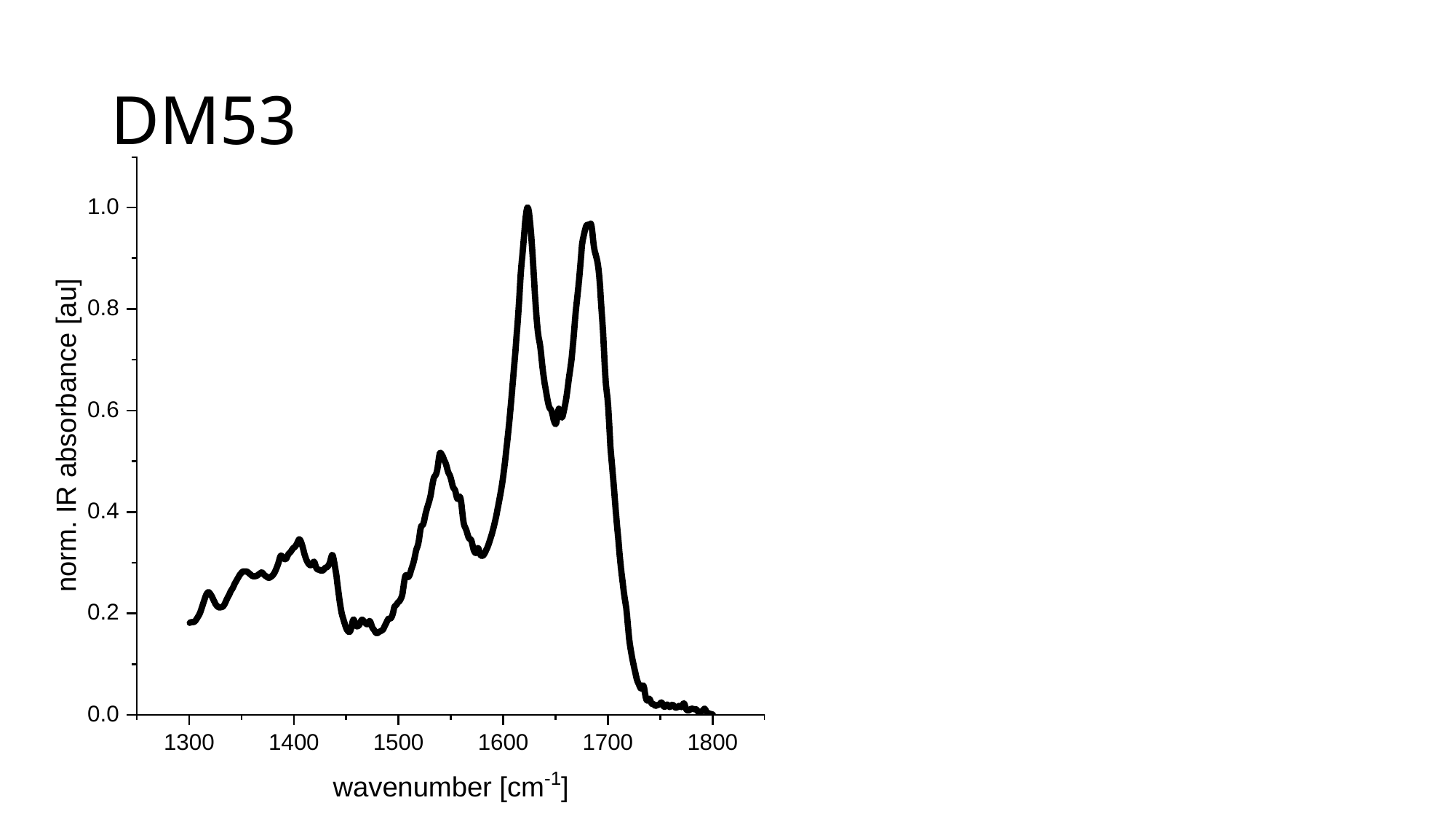

# DM53

## Slide 227
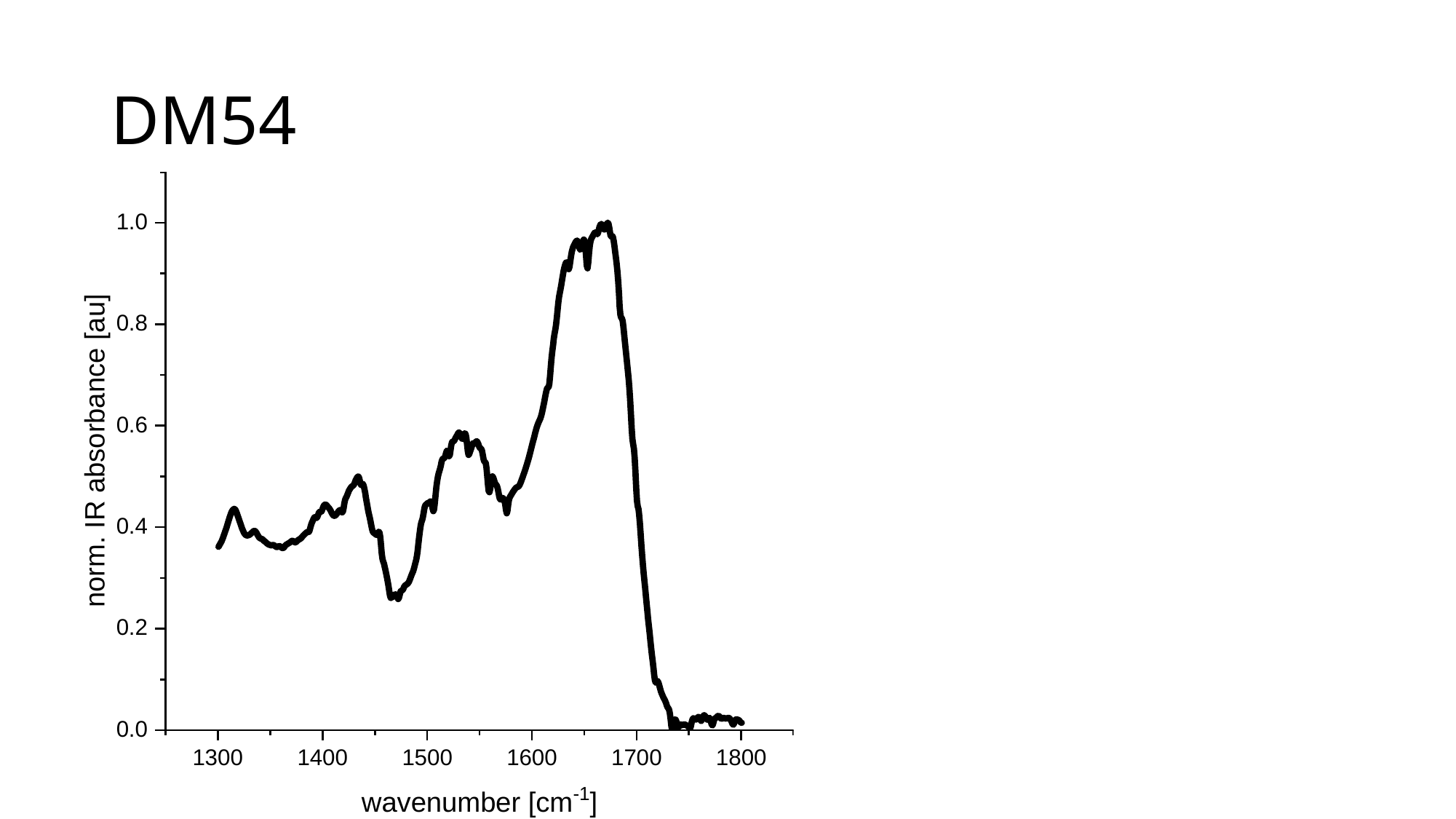

# DM54
